# Single‐cell transcriptomic atlas of taste papilla aging

**DOI:** 10.1111/acel.14308

**Published:** 2024-08-21

**Authors:** Wenwen Ren, Weihao Li, Xudong Cha, Shenglei Wang, Boyu Cai, Tianyu Wang, Fengzhen Li, Tengfei Li, Yingqi Xie, Zengyi Xu, Zhe Wang, Huanhai Liu, Yiqun Yu

**Affiliations:** ^1^ Department of Otolaryngology The Second Affiliated Hospital of the Naval Medical University (Shanghai Changzheng Hospital) Shanghai China; ^2^ ENT Institute and Department of Otorhinolaryngology, Eye & ENT Hospital, Fudan University Shanghai China; ^3^ Olfactory Disorder Diagnosis and Treatment Center Eye & ENT Hospital, Fudan University Shanghai China

**Keywords:** aging, circumvallate papillae, foliate papillae, mature taste cell, single‐cell RNA sequencing

## Abstract

Taste perception is one of the important senses in mammals. Taste dysfunction causes significant inconvenience in daily life, leading to subhealth and even life‐threatening condition. Aging is a major cause to taste dysfunction, while the underlying feature related to gustatory aging is still not known. Using single‐cell RNA Sequencing, differentially expressed genes between aged and young taste papillae are identified, including upregulated *mt‐Nd4l* and *Xist*, as well as downregulated *Hsp90ab1* and *Tmem59*. In the *Tmem59* ^−/−^ circumvallate papillae (CVP), taste mature cell generation is impaired by reduction in the numbers of PLCβ2^+^ and Car4^+^ cells, as well as decreases in expression levels of taste transduction genes. *Tmem59* ^−/−^ mice showed deficits in sensitivities to tastants. Through screening by GenAge and DisGeNET databases, aging‐dependent genes and oral disease‐associated genes are identified in taste papillae. In the CVP, aging promotes intercellular communication reciprocally between (cycling) basal cell and mature taste cell by upregulated *Crlf1*/*Lifr* and *Adam15*/*Itga5* signaling. By transcriptional network analysis, ribosome proteins, *Anxa1*, *Prdx5*, and *Hmgb1/2* are identified as transcriptional hubs in the aged taste papillae. Chronological aging‐associated transcriptional changes throughout taste cell maturation are revealed. Aged taste papillae contain more Muc5b^+^ cells that are not localized in gustatory gland. Collectively, this study shows molecular and cellular features associated with taste papilla aging.

## Graphical abstract

Differentially expressed genes between aged and young taste papillae were identified, including upregulated mt‐Nd4l and Xist, as well as downregulated Hsp90ab1 and Tmem59. Impairing taste receptor cell generation and decreases in expression levels of taste transduction genes were found in the Tmem59^−/−^ circumvallate papillae, and Tmem59^−/−^ mice showed deficits in sensitivities to tastants.

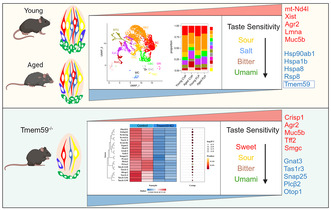

**Abbreviations**
BC: basal cell
CBC: cycling basal cell
CVP: circumvallate papillae
DEG: differentially expressed gene
EnC: endothelial cell
EpC: epithelial cell
ER: endoplasmic reticulum
FFP: fungiform papillae
FLP: foliate papillae
GC: goblet cell
GN: ganglion neuron
GO: gene ontology
IC: immune cell
MC: mesenchymal cell
MTC: mature taste cell
MuC: mucosal cell
SEC: stratified epithelial cell
TF: transcriptional factor
TMC: tongue muscle cell
TPC: taste progenitor cell
UMAP: uniform manifold approximation and projection
UMI: unique molecular identifier
UPR: unfolded protein response

## 1 INTRODUCTION

Gustation, an essential sense for mammals, plays crucial roles in nutrient intake, avoidance of toxicants, and food digestion. Taste disorders significantly and detrimentally impact health and life quality, leading to malnutrition, anorexia, depression, and even life‐threatening conditions. Taste perception is primarily mediated by taste buds located on the tongue. In mammals, taste buds are mainly situated on the tongue's surface and are restricted to taste papillae. Fungiform papillae (FFP) are found in the anterior two‐thirds of tongue, each housing only a single apical taste bud. In contrast, circumvallate papillae (CVP) and foliate papillae (FLP) are located in the posterior region of tongue, and both containing multiple and contiguous taste bud within the papilla epithelial walls (Barlow, 2015). Functionally, taste cells within the taste bud are classified into three subtypes. Glia‐resembling Type I cells, expressing NTPDase2 (Entpd2), have extensive cellular processes that tightly wrap around Type II and III cells (Miura et al., 2014). Type II cells employ G protein‐coupled receptor, expressing PLCβ2, TrpM5, Gustducin, and detect sweet, bitter, and umami tastes (Matsumoto et al., 2009). Type III cells are presynaptic sour detectors, marked by Snap25 and Car4 (Barlow & Klein, 2015). Taste cells undergo consistent renewal in adults. Krt5^+^ and Krt14^+^ basal cells constitute a progenitor population that generates new cells in taste buds. Lineage tracing data have shown that Shh^+^ basal cells derived from Krt5^+^/Krt14^+^ progenitors produce all three types of mature taste cells (Miura et al., 2014).

Although taste disorders are relatively rare in the general population, they are prevalent among cancer patients and elder individuals. Several reports have shown that aging affects taste perception. A decline in the ability to perceive sweet, salt, sour, and bitter tastes in older adults was reported (Braun et al., 2022). Another study highlighted a significant difference in the perception of salty taste among older adults (Alia et al., 2021). At the cellular level, Keita et al. found that aging decreased the voltage‐gated Na^+^ and K^+^ current densities in Type III but not Type II cells (Takeuchi et al., 2021). Our research also indicated that aging affected the generation of taste receptor cells in taste organoids (Ren et al., 2020). Besides, aging‐related reduction in taste function is due to the degradation of gustatory peripheral tissues and is related to alteration in neuronal circuits within the central nervous system (Iannilli et al., 2017). Aging‐induced change in taste perception is resulted from multiple factors, including declines in taste bud numbers and alterations of ion channels (Doty, 2018). Through comparing the response of flies to different appetitive tastants, it was reported that aging impaired the response to sugars but not to medium‐chain fatty acids. This reveals aging‐induced modality‐specific deficits in taste (Brown et al., 2024). However, the specific molecular mechanism underlying aging‐related distortion and loss of taste perception in mammals is still elusive.

Given that the CVP and FLP in the posterior tongue provide sufficient numbers of taste cells as well as taste stem/progenitor cells for analysis, we constructed a transcriptional atlas of young and aged CVP and FLP using scRNA‐Seq. We identified aging‐related differentially expressed genes (DEGs) in multiple cell types. *Tmem59* was downregulated in aged taste papillae, and knocking out this gene impaired taste receptor cell generation and taste sensitivities in mice. Through screening the GenAge and DisGeNET databases, we pinpointed aging‐dependent genes and oral disease‐associated genes in the aged taste papillae. Furthermore, we identified a new cell cluster in aged taste papillae with high Muc5b expression that was named after mucosal cell (MuC). These cells exhibited higher gene set scores related to the unfolded protein response (UPR) and oxidative stress. Additionally, aging enhanced the interactions between basal/progenitor cells and mature taste cells. Core transcriptional factors (TFs) including Anxa1, Prdx5, Hmgb1/2, as well as ribosome proteins such as Rps10, Rpl6, Rps4x, and Rpl35 were identified in various cell types of taste papillae through constructing transcriptional network by SCENIC. We identified a subset of genes such as Krt6a, Mki67, Ptch1, Icam1, and Lgr5 that exhibited differential expression patterns in the process of taste cell maturation established by pseudotime analysis. Collectively, this study reveals aging‐related regulators in taste papillae, providing critical insights into the molecular mechanisms underlying aging‐related gustatory alteration.

## 2 METHODS

### 2.1 Animals

Wide type C57BL/6J mice at 3 months (young) and 24 months (aged) of age were purchased from LEAGENE (Shanghai, China). *Tmem59* ^−/−^ mice were kindly provided by Dr. Tieqiao Wen (Shanghai University). Mice were housed under a 12/12 h light and dark cycle with free access to standard mouse chow and water. The procedures for animal handling and tissue harvesting were approved by the institutional animal care and use committee of the Naval Medical University (Permit Number: SYXK2022‐0011).

### 2.2 Immunostaining

Mice were deeply anesthetized by intraperitoneal injection of ketamine‐xylazine (200 and 15 mg/kg body weight) before decapitation. The CVP tissues were dissected from tongues and fixed in 4% paraformaldehyde (Sigma Aldrich) overnight at 4°C, and infiltrated in a series of sucrose solutions before being embedded in OCT. The frozen tissues were cut into 20 μm sections on a cryostat (Leica CM1950). After rinsing with PBS, the tissue sections were blocked for 1 h in 0.3% Triton X‐100 in phosphate‐buffered saline with 5% bovine serum albumin, and then incubated at 4°C with the primary antibodies overnight, followed by incubation at room temperature with secondary antibodies for 1 h. The primary antibodies used were as follows: goat anti‐Car4 (Abcam, 1:20, #AF2414), rabbit anti‐PLCβ2 (Santa Cruz, 1:100, #SC_515912), rat anti‐Krt8 (Developmental Studies Hybridoma Bank, 1:10, #AB_531826), rabbit anti‐NTPDase2 (Centre de Recherche du CHUL, 1:500, #AB_2314986), goat anti‐ICAM1 (R&D Systems, 1:500, #AF796), rabbit anti‐Krt5 (Abcam, 1:100, #AB_52635), mouse anti‐Muc5b (Abcam, 1:100, #AB_77995), mouse anti‐Txn1 (Santa Cruz, 1:100, #SC_166393), rabbit anti‐Cdkn1a (Proteintech, 1:50, #10355‐1‐AP), rabbit anti‐Apoe (Abcam, 1:300, #ab183597), rat anti‐Cd45 (Ebioscience, 1:200, #14–0451‐81), rat anti‐F4/80 (Bio‐Rad, 1:300, #MCA497GA), mouse anti‐Mki67 (BD Biosciences, 1:100, #550609). The secondary antibodies used in the experiments were as follows: Alexa Fluor 488 Donkey anti‐Rabbit (ThermoFisher, #A21206), Alexa Fluor 647 Donkey anti‐Rabbit (ThermoFisher, #A31573), Alexa Fluor 594 Donkey anti‐Goat (ThermoFisher, #A11058), Alexa Fluor 633 Donkey anti‐Goat (ThermoFisher, #A21082), Alexa Fluor 488 Goat anti‐Mouse IgG (ThermoFisher, #A11001), Alexa Fluor 568 Goat anti‐Mouse IgG (ThermoFisher, #A11004), Alexa Fluor 647 Goat anti‐Rat IgG (ThermoFisher, #A11006), and donkey anti‐rat Alexa Fluor 647 (Abcam, #AB_150155). Tissues were mounted in Vectashield (Vector Laboratories). Fluorescent images were taken under a SP5/Leica confocal microscope with LAS AF Lite software.

### 2.3 RNAscope assay

Tissue sections were sequentially rinsed in 50%, 70% and 100% ethanol, followed by PBS to remove OCT. Sections were then pretreated with RNAscope hydrogen peroxide, RNAscope target retrieval reagent and RNAscope protease III to increase tissue permeability and facilitate the binding of probes to target RNA in tissues. After these pretreatments, 4–6 drops of *Tmem59* (ACD‐Bio, #557001), *Fst* (ACD‐Bio, # 454331), *Ovol3* (ACD‐Bio, #1078571‐C1) or *Lgr5* probe (ACD‐Bio, #312171) was added, and the sections were incubated at 40°C for 2 h in HybEZTM hybridization oven. The sections were then washed twice with fresh RNAscope 1× washing buffer (ACDBio kit, catalog number 323100‐USM). After washing, 4–6 drops of RNAscope multichannel second‐generation fluorescent AMP1 were added, and the sections were incubated at 40°C for 30 min in HybEZTM hybridization oven. The sections were then hybridized with AMP2 and AMP3, followed by signal labeling of C1 channel probe. Then, 4–6 drops of RNAscope multichannel second‐generation fluorescent HRP‐C1 were added, and the sections were incubated at 40 °C for 15 min in HybEZTM hybridization oven. Subsequently, 150–200 μL diluted TSA Plus fluorescent dye was added, and the sections were incubated for 30 min at 40°C. Finally, 4–6 drops of RNAscope multichannel second‐generation fluorescent HRP blocker was added to cover the tissues, and the sections were incubated at 40°C for 15 min. Slides were mounted with Vectashield (Vector Laboratories). Images were captured using a SP8/Leica confocal microscope with LAS AF Lite software.

### 2.4 PinpoRNA in situ hybridization

RNA in situ hybridization for *Xist* was performed using the PinpoRNA™ kit (Shanghai Feldspar Biotechnology). Frozen sections were fixed in 4% PFA for 30 min, and immersed in 70% and 100% ethanol sequentially for 5 min each. After air‐drying the slides at room temperature, 6–8 drops (300–400 μL) of pretreatment solution A were added to completely cover the tissue. The slides were then kept at room temperature for 30 min and washed three times with distilled water. The slides were immersed in the boiled pretreatment solution B for 15 min. Next, 200–400 μL of preheated 1× enzyme working solution was added, and digestion was carried out at 40°C for 15 min. Following this, 200–250 μL of *Xist* probe (2137421‐B1) were added to cover the tissue section, and hybridization was carried out at 40°C for 2 h. The reaction 1 and 2 working solutions were added, and the slides were incubated at 40°C for 25 and 15 min, respectively. Channel I Fluorescent Labeling was then performed by adding the reaction 3 type I working solution for 15 min at 40°C. Finally, 50–150 μL of freshly prepared fluorescent reaction working solution (Opal™520 tyramide fluorescent substrate diluted in reaction solution at 1:300) was added, and the reaction was carried out at room temperature in the dark for 30 min. Nuclear staining and mounting were then performed, and the slides were observed under a fluorescence microscope. Images were captured using a SP8/Leica confocal microscope with LAS AF Lite software.

### 2.5 Single cell isolation

Tongues from wide‐type C57BL/6J mice at 3 months (young) and 22–24 months (aged) of age were injected with approximately 0.5 mL of an enzyme mixture containing elastase (0.1 U/mL, Sigma), protease (2 mg/mL, Sigma), and DNase I (10 mg/mL, Invitrogen) or dispase (2 mg/mL, Roche) and collagenase (1 mg/mL, Roche) in Tyrode's solution (145 mM NaCl, 5 mM KCl, 10 mM Hepes, 5 mM NaHCO3, 10 mM pyruvate, 10 mM glucose) for 15 min at 37°C. Tongue epithelium was gently peeled from the connective tissue underneath, and the regions surrounding the CVP and FLP were dissected out and collected. Samples were minced with scissors into 1 mm^2^ piece in PBS on ice and transferred into 15‐mL centrifuge tubes, and digested with 0.25% trypsin–EDTA at 37°C for 15–30 min. Single‐cell suspension was prepared by pipetting with fire‐polished glass tube, and the suspension was passed through 70‐mm and 40‐mm strainers (BD Falcon). Single cells were collected by centrifugation at 1500 r.p.m. for 5 min at 4°C before washed twice in cold PBS. The cells were resuspended in PBS with 0.04% bovine serum albumin and used for 10× Genomic sequencing. Three independent samples for each group were prepared, and taste tissues in each sample were collected from six mice.

### 2.6 Single‐cell RNA sequencing

Single‐cell suspensions were counted using a Luna fluorometer (Logos Biosystems) wiyh Trypan dye staining, ensuring viability of more than 90%. Cells from young CVP, young FLP, aged CVP, aged FLP were loaded into a Chromium Single Cell 3′ Chip (10x Genomics) and processed following the manufacturer's instructions. Single‐cell complementary DNA (cDNA) libraries were prepared using the Chromium Next GEM Single Cell 3′ Reagent Kit v3.1 according to the manufacturer's instructions by Genergy Bio‐Technology Co., Ltd (Shanghai, China). Samples were run using 10× Genomics based on the manufacturer's protocol. All the libraries were sequenced on the NovaSeq 6000 Sequencing System (Illumina).

### 2.7 scRNA‐seq raw data processing

Sequence data were processed by the 10× Genomics Cell Ranger pipeline (version 5.0.0), by which the quality of FASTQ file was evaluated through alignment to the mouse reference genome (mm10). The digital gene expression matrix was generated, while transcript expression level was determined by the number of unique molecular identifiers (UMIs). In total, 68,608 cells were filtered for scRNA‐Seq analysis, comprising 18,059 cells from young CVP, 15,426 cells from young FLP, 8501 cells from aged CVP, and 26,622 cells from aged FLP. These filtered gene expression matrices were utilized for subsequent analyses.

### 2.8 scRNA‐seq data analysis and cell‐type identification

The data from four samples were analyzed in R (version 4.1.0), using the Seurat package (version 4.0.3). For quality control, cells with fewer than 200 genes or more than 6000 genes detected, and those with more than 10% mitochondrial genes were excluded from the downstream analyses. After quality control, a total of 21,627 cells remained (7240 from young CVP, 7572 from young FLP, 2706 from aged CVP and 4109 from aged FLP) and were used for bioinformatic analyses. Integration and normalization across all samples were conducted through the standard Seurat procedure by using the “IntegrateData” function. Using “NormalizeData” function, normalized UMI values in each cell were obtained through normalizing sequencing reads for each gene to total UMIs. We scaled and centered expression levels in the data set by using the “ScaleData” function for dimensional reduction, which was performed with “RunPCA” function and the principal components (PCs) were then used for downstream dimensional reduction and clustering analyses.

For cell type identification, total cell clustering was performed using “FindClusters” function at a resolution of 0.5 and the first 50 PCs (determined by “Elbow plot”). Dimensionality reduction was performed with “RunUMAP” function and visualized using Uniform Manifold Approximation and Projection (UMAP). Marker genes for each cluster were determined with the “MAST” test by “FindAllMarkers” function implemented in the Seurat Package. For each cell type, we used multiple cell type‐specific marker genes to determine the identity of each cell type. Marker genes for each cluster were shown in Tables S1 and S2. The total number of identified cells was 21,627, classified into the following cell types: 4269 basal cells (BC), 7250 stratified epithelial cells (SEC), 1947 taste progenitor cells (TPC), 1813 cycling basal cells (CBC), 322 mesenchymal cells (MC), 2818 epithelial cells (EpC), 1361 mature taste cells (MTC), 163 endothelial cells (EnC), 27 ganglion neurons (GN), 1189 mucosal cells (MuC), 437 immune cells (IC) and 31 tongue muscle cells (TMC).

### 2.9 Identification of aging‐related DEGs

The function of “FindMarkers” in Seurat was used to identify aging‐related DEGs for each cell type between the aged and young groups. The log fold change (Log2FC) and adjusted *p*‐value of each DEG were determined using the nonparametric two‐sided Wilcoxon rank‐sum test. Aging‐related genes were defined as |“avg_log2FC”| >0.5 and “p_val_adj” <0.05. All DEGs were listed in Tables S3 and S4.

### 2.10 Identification of aging‐dependent DEGs

Aging‐dependent mouse genes were obtained from the GenAge database and categorized based on “lifespan effect” (increase or decrease) and “longevity influence” (anti or pro). Overlapping genes between aging‐related DEGs in the CVP or FLP and GenAge genes were selected and shown in Figure S4.

### 2.11 Identification of aging‐related genes associated with oral diseases

To identify genes associated with oral diseases in aged taste papillae, aging‐related genes were screened by DisGeNET platform (https://www.DisGeNET.org/). Overlapping genes between aging‐related DEGs in the CVP or FLP and genes associated with mouth dryness (C0043352), mouth neoplasm (C0026640), mouth ulcer (C0149745) were identified and shown in Figure S5.

### 2.12 Gene ontology (GO) analysis

GO analysis was performed by clusterProfiler R package (version 3.15, https://bioconductor.org/packages/clusterProfiler/) and visualized with the ggplot2 R package (version 3.3.6, https://github.com/tidyverse/ggplot2). Representative GO terms selected from the top 20 ranked ones (*p* < 0.05) were displayed.

### 2.13 Gene set score analysis

We used the “AddModuleScore” function of the Seurat R package to calculate gene set scores in single cells. First, all the analyzed genes were binned based on average expression levels, and control genes (*n* = 100) were randomly selected from each bin. Then, the average expression value of the gene set at the single‐cell level was calculated by subtracting the aggregated expression of the control gene set. Finally, the group, cell‐type and ModuleScore information for each cell type was extracted, and box plots were drawn using the ggplot2 R package. Differences between groups were determined using the nonparametric two‐sided Wilcoxon rank‐sum test via “stat_compare_means” function implemented in the ggpubr Package (version 0.4.0). All gene sets were obtained from the Molecular Signatures Database (MSigDB) database (https://www.gsea‐msigdb.org/gsea/msigdb/).

### 2.14 Transcriptional regulatory network analysis

To analyze the transcriptional regulatory network through default parameters, we used the GENIE3 (version 1.18.0) and RcisTarget (version 1.14.0) R packages of the SCENIC (version 4.1.3) workflow. Transcriptional factors (TFs) of mm10 were used as reference TFs. GENIE3 was used to infer gene regulatory networks from the gene expression matrix of all CVP and FLP cells. The gene expression matrix of 9946 cells and 5448 aging‐related DEGs from CVP, 11,681 cells and 4291 aging‐related DEGs from FLP were normalized from Seurat as input. For each gene regulatory networks, cis‐regulatory motif enrichment analysis was performed among all potential target genes by RcisTarget, and those enriched with the motifs of the corresponding TFs were defined as direct target genes. Only regulons (TF and its direct target genes) with weight value greater than 1 were displayed in the network. The TF module networks were visualized by Cytoscape (version 3.8.2).

### 2.15 Constructing cell trajectories along the pseudotime

Monocle2 (version 2.22.0) package was applied to construct single‐cell trajectories of each BC, TPC, and MTC type from young to aged stages. We used the “differentialGeneTest” function to calculate pseudo‐temporal dependent DEGs (fullModelFormulaStr = “celltype,” qval <0.01). Pseudo‐temporal dependent DEGs were set as “ordering_genes”. The DDRTree algorithm was used for dimensional reduction. Changes in the expression level of DEGs along the pseudotime were demonstrated by “plot_pseudotime_heatmap” function.

### 2.16 Intercellular communication analysis

To assess cell–cell communication between different cell types, we used CellChat (Version 1.0.0, http://www.cellchat.org/) to analyze intercellular communication networks from scRNA‐Seq data of young and aged taste papilla tissues with default parameters. Only interactions with *p* < 0.05 were considered to be real.

### 2.17 Receptor‐ligand interaction analysis

We performed NicheNet intercellular communication analysis to determine the receptor‐ligand interaction. CBC/BC and MTC were defined as “sender” and “receiver” cells, and vice versa. For upregulating communication analysis, “Receiver” cell genes were upregulated DEGs between aged and young cells. Ligands upregulated in aged cells were defined as genes of “Sender.” The upregulated ligand activity in the aged CVP was plotted by ggplot2. Parameters and significance thresholds for receptors, ligands, and target genes were default parameters of NicheNet.

### 2.18 RNA‐seq analysis

RNA‐Seq analysis was conducted by Personal Biotechnology Co., Ltd (Shanghai, China). Total RNA was isolated using Trizol and assessed for quality with a NanoDrop spectrophotometer. cDNA synthesis and adapter ligation were followed by size selection and purification using the AMPure XP system (Beckman Coulter). The enriched libraries were quantified and sequenced on the NovaSeq 6000 platform. All the sequence data was analyzed by using the online platform Personalbio GenesCloud (https://www.genescloud.cn), and deposited on the NCBI Sequence Read Archive (BioProject ID: PRJNA1127061).

### 2.19 Taste behavior test

We conducted a drinking test using WT‐young (*n* = 6, 3 month), WT‐aged (*n* = 6, 24 month) and *Tmem59* ^−/−^ (*n* = 6, 3 month) mice. Each mouse was individually caged and given two graduated drinking bottles (volume = 40 mL). Two‐bottle preference (TBP) was carried out. Briefly, the mice underwent a two‐day adaptation period with deionized water in both bottles. The test solution was placed in one of bottles, with the bottles exchanged every 12 h to eliminate biases due to taste preference memory of mice. We used sucrose (0.1, 0.3, 1, 3, 10 mM), sodium glutamate (10, 30, 100, 300, 600 mM), sodium chloride (37.5, 75, 150, 300, 600 mM), citric acid (0.3, 1, 3, 10, 30 mM), and quinine (0.003, 0.01, 0.03, 0.1, 0.3 mM) as the representative substances for the taste of sweet, umami, salty, sour, and bitter, respectively. Each concentration of the same taste solution was tested continuously, with a 5–7 days interval between different taste solutions, during which deionized water was provided in both bottles. The volume of solution in each bottle was recorded at the beginning and end of each flavor solution test.

### 2.20 Taste organoid culture

The isolated single cells from CVP of both young and aged mice were cultured in the conditioned medium with matrigel in a low‐attachment 24‐well plate, following the procedure described in our previous report (Ren et al., 2017). Mediums were replaced every 3 days. The size of organoids was measured using ImageJ at Day 3 and Day 7. At Day 14, organoids were collected and subjected to immunostaining to assess their cellular composition.

### 2.21 Analysis of RNAscope and PinpoRNA in situ hybridization data

Fluorescent images of taste papilla tissues were captured under identical exposure and experimental conditions using a Leica SP8 scanning confocal microscope with LAS AF Lite software. Images were converted to 8‐bit greyscale using ImageJ (National Institute of Health, https://imagej.nih.gov/ij/). A “threshold” was set and binarized images were filtered by pixel size to reduce background noise and enhance contrast. The expression levels of *Xist*, *Tmem59*, *Lgr5*, *Fst*, and *Ovol3* in tissue sections were quantified as mean gray value (Mean). Using ImageJ software, positive staining was converted into pixels and the Mean (Integrated Density/Area) was calculated. For the vertical axis of histograms, arbitrary units (a.u.) were used instead of Mean to indicate mRNA expression levels. We used circles, triangles, or squares to represent samples from one of the three mice.

### 2.22 Statistical analysis

Cell counts and fluorescence intensity measurements from the confocal images were performed using ImageJ. The number of positively stained cells and DAPI^+^ cells per organoid, taste bud, and CVP and FLP tissue sections were quantified using ImageJ software. The percentage of positively stained cells was determined by [the number of positively stained cells] / [the number of DAPI^+^ cells]. Data were presented as the mean ± SEM. The statistical differences between two groups were determined by unpaired student's *t* test using GraphPad Prism 8.02 software. The two‐sided nonparametric Wilcoxon's rank‐sum test with Bonferroni's correction was used to determine the significance of all genes and gene set scores between young and aged groups in the dataset. For all statistical analyses, *p*‐values lower than 0.05 were considered statistically significant. *, **, ***, and **** indicated *p* < 0.05, *p* < 0.01, *p* < 0.001 and *p* < 0.0001, respectively.

## 3 RESULTS

### 3.1 Identification of cell types in the circumvallate and foliate papillae

**FIGURE 1:**
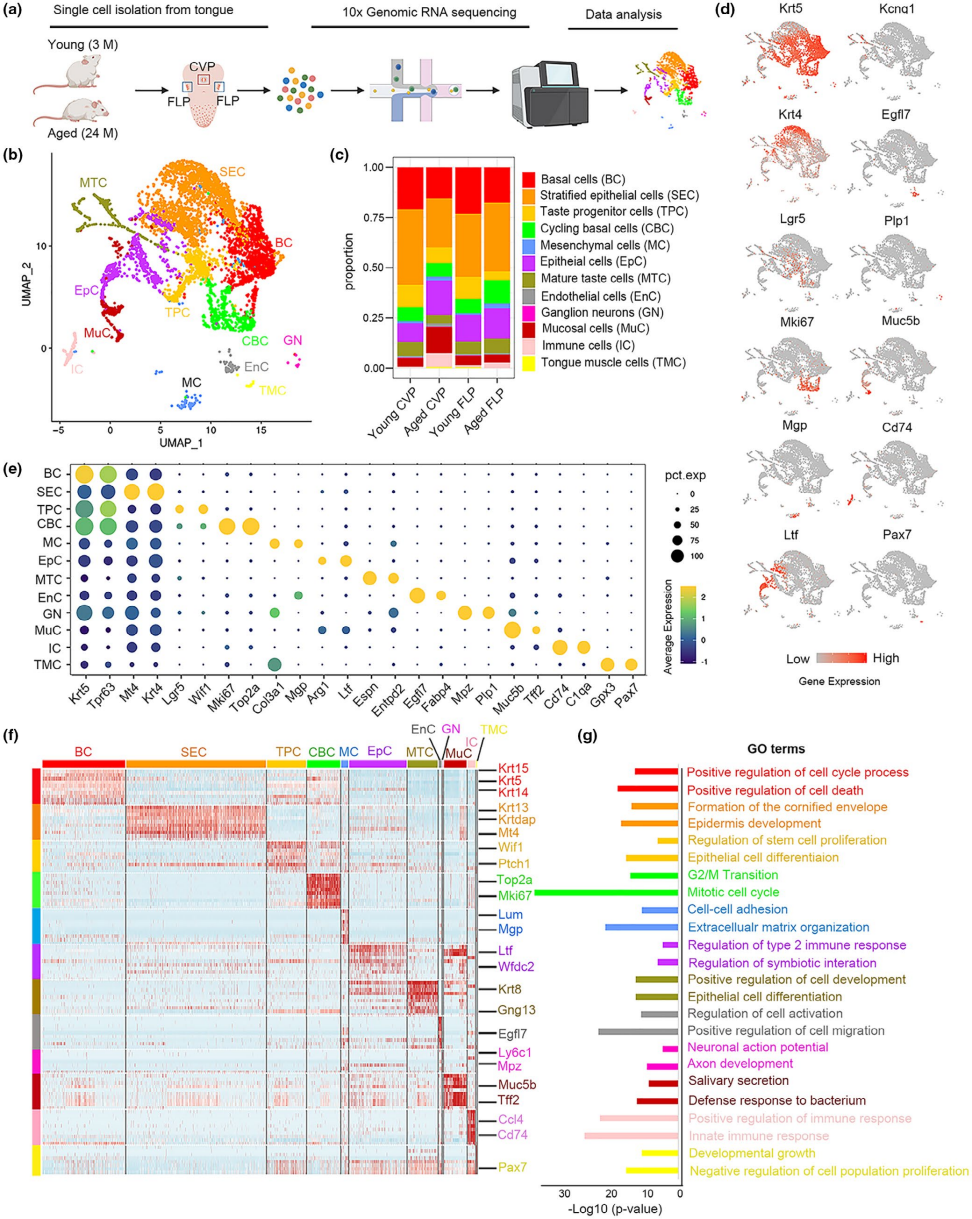
Cell type identification by scRNA‐Seq analysis of young and aged mouse taste papillae. (a) Method flowchart of scRNA‐Seq. (b) UMAP plot showing 12 cell types in taste papillae. (c) Bar graph showing the proportions of cell subtypes. Cells were colored by types and were annotated to the right. (d) Selected gene expression plots demonstrating the molecular markers of representative cell types in taste papillae. The color scale from gray to red represented low to high gene expression levels. (e) Dot plot showing the expression of representative molecular markers for each cell type. Dot size reflected percentage of cells in a cluster expressing each gene, and dot color reflected expression level. (f) Heatmap showing gene expression signatures of each cell type. (g) Enriched GO terms for each cell type.

After filtering, 21,627 cells from CVP and FLP tissues of mice at ages of 3‐ and 24‐month‐old were subjected to further analysis (7240, 2706, 7572, 4109 cells from the young CVP, aged CVP, young FLP, aged FLP) (Figure 1a). Genes with at least three UMIs in at least five cells were used for downstream clustering and cell‐type identification (Figure S1a–c). Two dimensions via UMAP were used to analyze cellular heterogeneity (Figures 1b and S1d). Cell subtypes were assigned using multiple known molecular markers: BC (Krt5^+^/Trp63^+^), SEC (Krt4^+^/Mt4^+^), TPC (Lgr5^+^/Wif1^+^), CBC (Mki67^+^/Top2a^+^), MC (Col3a1^+^/Mgp^+^), EpC (Arg1^+^/Ltf^+^), MTC (Espn^+^/Entpd2^+^), EnC (Egfl7^+^/Fabp4^+^), GN (Mpz^+^/Plp1^+^), MuC (Muc5b^+^/Tff2^+^), IC (Cd74^+^/C1qa^+^) and TMC (Gpx3^+^/Pax7^+^). We mapped the gene expression profiles of well‐defined cell type‐specific markers in the UMAP plot (Figure 1d). Dot plot showed the distribution of expression levels of two representative marker genes across all 12 cell types (Figure 1e). Aging‐induced alterations in cellular proportions were more prominent in the CVP than FLP tissue, with apparent increases in MuC and IC, as well as decreases in BC, TPC and MTC (Figure 1c). Gene ontology analysis of the top 20 marker genes for each cell type revealed unique biological functions (Figure 1f,g). For instance, terms related to stem cell proliferation and epithelial cell proliferation were enriched for TPC, while salivary secretion and defense response to bacteria were enriched for MuC. Thus, this initial analysis of taste papillae from young and aged mice captures the range of cell populations present in original tissues.

### 3.2 Aging‐related DEGs in the circumvallate and foliate papillae at the single‐cell resolution

**FIGURE 2:**
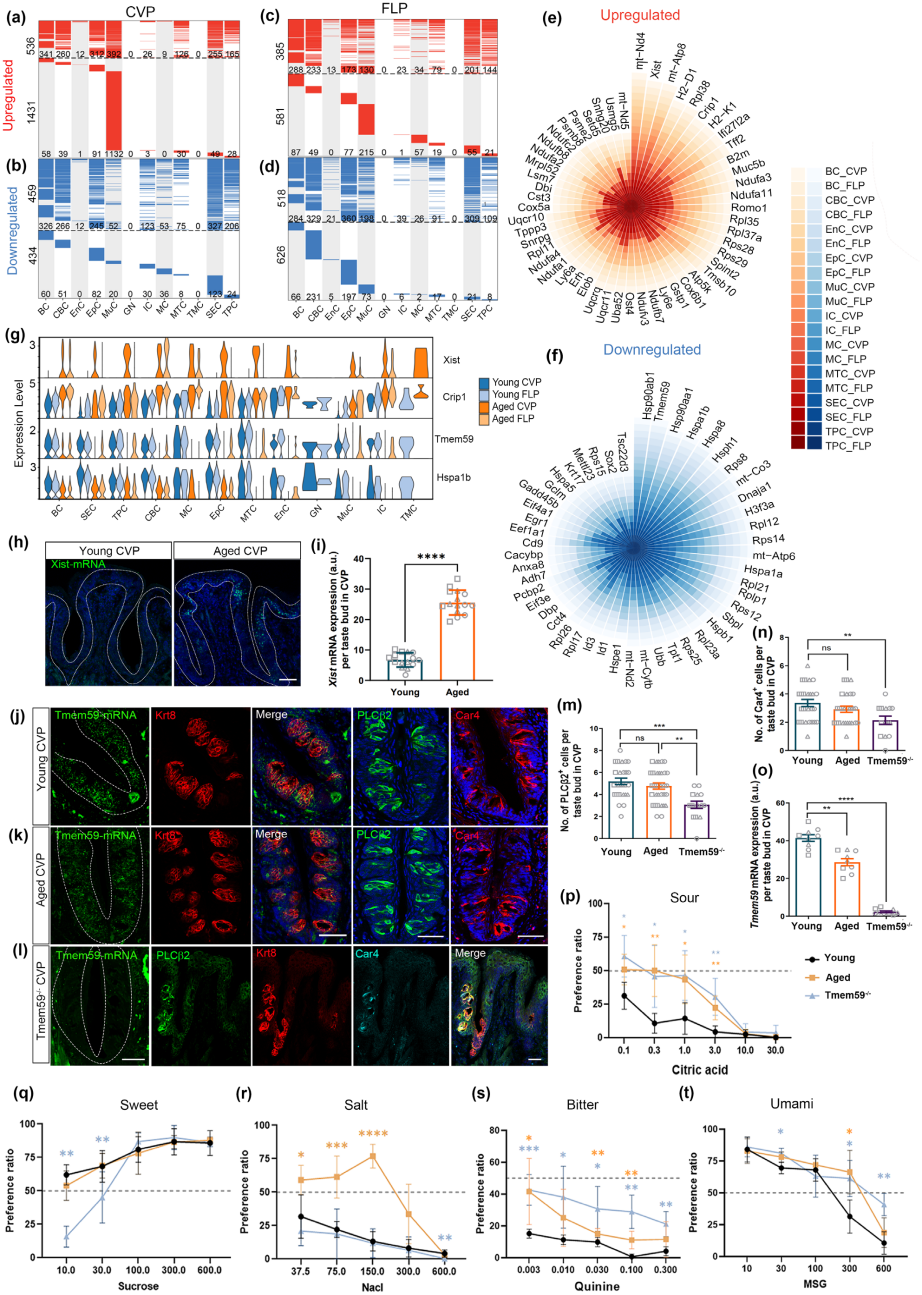
Identification of aging‐related genes in taste papillae. (a–d) Heatmaps showing the distribution of upregulated (red) and downregulated (blue) genes for each cell type between the aged and young CVP (a, b) and FLP (c, d). The upper part above dotted lines demonstrated the DEGs shared across at least two cell types, while the lower panel indicated the unique DEGs in each cell type. (e, f) Plots showing the upregulated DEGs shared by at least nine cell types (e) and downregulated DEGs shared by at least ten cell types (f). (g) Violin plots showing differential expression of upregulated *Xist* and *Crip1*, as well as downregulated *Tmem59* and *Hspa1b* in cell types of aged taste papillae. (h, i) Confocal images and quantification of *Xist*‐mRNA^+^ cells in the young and aged CVP. *n* = 15 section in each group. (j–l) Confocal images of *Tmem59*‐mRNA^+^, PLCβ2^+^, Car4^+^ cells in the CVP of young (j), aged (k), and *Tmem59* ^−/−^ mice (l). (m–o) Quantification of PLCβ2^+^ (m), Car4^+^ (n) cells and *Tmem59*‐mRNA^+^ signal intensity (o). PLCβ2: *n* = 26, 31, 15 sections for young, aged, Tmem59^−/−^ group. Car4: *n* = 25, 25, 14 sections for young, aged, Tmem59^−/−^ group. Tmem59: *n* = 9 sections for each group. (p–t) Two bottle preference assay data showing sensitivities to citric acid (p), sucrose (q), NaCl (r), quinine (s), MSG (t) in young, aged, and Tmem59^−/−^ mice. *n* = 6 mice in each group. *p < 0.05, **p < 0.01, ***p < 0.001, ****p < 0.0001; ns, not significant (by unpaired *t* test). Scale bars, 50 μm.

We then identified DEGs between the aged and young taste papillae. In the aged CVP, MuC subtype exhibited the highest number of upregulated genes including both globally shared and type‐specific ones, compared to young tissue (Figure 2a). The type‐specific upregulated genes were also enriched in BC, EpC and SEC, while globally shared upregulated genes were present in multiple cell types except for GN and TMC due to their limited cell number (Figure 2a). SEC in the aged CVP had the highest number of downregulated genes. The type‐specific downregulated genes were mainly distributed in BC, CBC, EpC, and SEC (Figure 2b). Similar to the upregulated genes in the aged CVP, globally shared downregulated genes were identified across multiple cell subtypes, except for EnC, GN, and TMC (Figure 2b). In the FLP, aging led to globally shared upregulated expression in the most of cell subtypes, except for EnC, GN, and TMC. Cell type‐specific upregulated genes were mainly enriched in BC, CBC, EpC, MuC, MC, and SEC (Figure 2c). Besides, CBC showed the highest number of downregulated genes in the aged FLP. The cell type‐specific downregulated genes were enriched in BC, CBC, EpC, and MuC of the aged FLP, while globally shared downregulated genes were found in most of cell subtypes except for GN and TMC (Figure 2d). Collectively, aging significantly affects multiple cell types such as BC, CBC, EpC, MuC, and SEC in the taste papillae. GO analysis revealed that upregulated genes in the aged CVP and FLP were associated with ribosome biogenesis and assembly, and with oxidative phosphorylation in the aged FLP (Figure S2a,c), while downregulated genes in the aged CVP and FLP were mainly related to response to unfolded protein and protein folding (Figure S2b,d).

To further delineate differential gene expression across all cell types during the aging process, we identified 91 upregulated genes and each of them was upregulated in at least nine cell types of the aged taste papillae compared to young ones, along with 80 downregulated genes in at least ten cell types (Figure 2e,f). The top 11 frequently upregulated genes included *mt‐Nd4l*, *Xist*, *mt‐Atp8*, *H2‐D1*, *Rpl38*, *Crip1*, *H2‐K1*, *Ifi27L2a*, *Tff2*, *B2m*, *Muc5b*, while the top 10 shared downregulated genes were *Hsp90ab1*, *Tmem59*, *Hsp90aa1*, *Hspa1b*, *Hspa8*, *Hsph1*, *Rps8*, *mt‐Co3*, *Dnaja1*, *H3f3a* (Figure 2e,f). Notably, *Xist* was significantly upregulated across all cell types in aged taste papillae, with particularly high expression levels in BC, CBC, EnC, EpC, IC, MTC, SEC, TPC in the aged CVP and BC, CBC, EnC, IC, SEC, TPC of the aged FLP compared to young tissues (Figure S2e–g). RNA in situ hybridization confirmed a 2.9 ± 0.2 folds increase in *Xist* expression in the aged CVP (*p* < 0.0001, Figure 2h,i). *Xist* was reported to function in inflammatory and apoptotic progression (Cao et al., 2022; H. Wang et al., 2024; Xia et al., 2021; Zhang, Wang, et al., 2022). A recent study reported that *Xist* was a female‐specific feature of hypothalamic aging (Hajdarovic et al., 2022). These reports suggest that *Xist* potentially functions as a marker in aging of taste papillae, and we hypothesized that aging‐related *Xist* upregulation in the aged CVP may play a role in apoptosis and development of inflammation. In the aged CVP and FLP, MuC showed elevated levels of *Muc5b* and *Tff2* (Figure S2e,f). *Muc5b* expression was augmented in airway epithelial cells in response to *Saccharopolyspora rectivirgula* antigen and caused inflammatory aggravation (Okamoto et al., 2022). Increased *Tff2* expression resulted in MuC metaplasia and bronchial epithelium apoptosis (Royce et al., 2013). Thus, significant elevation in *Muc5b* and *Tff2* expression in MuC of the aged taste papillae is likely to cause inflammatory activation and apoptosis. Additionally, aged FLP exhibited higher expression of inflammatory markers *Ptprc* (*CD45*) and *Adgre1* (*F4/80*) in IC, compared to young one (Figure S2g). Immunostaining data showed 218% ± 25% and 119% ± 18% more Ptprc ^+^ and Adgre1^+^ inflammatory cells in the aged FLP (*p* < 0.0001, Figure S2h,i). This indicates an inflammatory environment in the aged taste papillae. Aging also led to upregulation of *Lyz2* (a microglial activation gene) in EpC and MTC of the aged FLP, suggesting the potential activation of inflammatory microenvironment (Figure S2f). *Gng13*, a taste‐signaling molecule (Huang et al., 1999), was upregulated in MTC of aged CVP, suggesting that the bitter and sweet perception may be affected by aging (Figure S2e). A subset of transcriptional factors including *Jun*, *Atf3*, *Fos* in BC of the aged CVP and *Egr1*, *Cebpd*, *Fos*, *Jun*, *Junb* in TPC of the aged FLP were apparently downregulated. In several cell subtypes including CBC, EnC, SEC, TPC of the aged CVP and CBC, IC, MTC, SEC of the aged FLP, heat shock protein *Hsp90aa1* was one of the mostly downregulated genes (Figure S2e,f). Previous work indicated that upregulation of *Hsp90aa1* alleviated the ER stress damages in mouse cochlea (Sun et al., 2023). *Hsp90aa1* is a stress inducible‐protein that aids protein folding to maintain cell homeostasis. Therefore, downregulation of this gene in several cell types of the aged taste papillae may cause incorrect folding and aggravate ER stress damage. Other Hsp types such as *Hspa1b* were also significantly downregulated across most of taste papilla cell types (Figure 2g).

*Tmem59* was one of the significantly downregulated genes in EnC, MTC and TPC of aged CVP (Figure 2f,g). *Tmem59* deletion impairs olfactory function, neuronal homeostasis and regeneration in the olfactory epithelium (Ma et al., 2023), indicating a essential role in sensory perception. Our scRNA‐Seq data showed widespread downregulation of *Tmem59* across all cell types in the aged CVP and FLP compared to young tissues (Figure 2g). RNAscope analysis confirmed the lower expression of *Tmem59* in the aged CVP than in young tissue, with a reduction in intensity of *Tmem59*‐mRNA^+^ signals by 30% ± 3% (*p* = 0.002, Figure 2j,k,o). To further elucidate the role of *Tmem59*, we examined the alteration in the number of taste receptor cells using *Tmem59* ^−/−^ mice, and found a significant decrease by 35% ± 5% in the number of PLCβ2^+^ Type II cells and by 25% ± 7% in the number of Car4^+^ Type III cells of the *Tmem59* ^−/−^ CVP compared to WT tissue (*p* < 0.001 for PLCβ2^+^ cells, *p* = 0.0051 for Car4^+^ cells, Figure 2j,l,m,n).

To further investigate whether aging and *Tmem59* knockout impaired the gustatory function, we performed taste behavior test using TBP assay. As shown in Figure 2p–t and S3a, aged mice showed higher preferences to salt, bitter, sour and high concentration of umami tastants, compared to young mice. *Tmem59* ^−/−^ mice showed less preference to low concentration of sucrose, and more preferable to quinine, low concentration of critic acid and high concentration of MSG, compared to young mice. Thus, *Tmem59* deletion alters taste sensitivities to different tastants.

To better explain whether *Tmem59* knockout in CVP affected the generation of mature taste receptor cells, we conducted bulk RNA‐Seq on CVP dissected from young WT (Control) and *Temem59* ^−/−^ mice. The gene expression profiles between *Tmem59* ^−/−^ and control groups displayed obvious differences, with the top 20 DEGs between the two groups shown in Figure S3b. Volcano plot indicated representative upregulated genes such as Crisp1, Muc5b, Agr2, Bpifb2, Smgc, Acta2, Ly6c1, Sval2, Tff2, Bpifa2, and downregulated genes including Ly6d, Krt75, Tmem59, Krt14, Krt6, Chil4, Krt6a, Gnat3 in the *Tmem59* ^−/−^ CVP, compared to WT group (Figure S3c). KEGG enrichment analysis of the upregulated genes in the *Tmem59* ^−/−^ group indicated significant enrichment in pathways like regulation of actin cytoskeleton, PI3k‐Akt signaling pathway and salivary secretion, while the downregulated genes were mainly enriched in pathways such as taste transduction, tight/gap junction, Hippo/Wnt/Rap1 signaling pathway (Figure S3d,e). Additionally, many genes related to taste transduction, including Tas1r1, Tas1r2, Tas1r3, Plcβ2, Snap25, Otop1, were downregulated in the *Tmem59* ^−/−^ CVP (Figure S3f). GSEA enrichment plot revealed upregulation of pathways like epidermis development and downregulation of pathways such as epidermal cell differentiation, keratinization, keratinocyte differentiation, and G protein‐coupled receptor activity in the *Tmem59* ^−/−^ group compared to controls (Figure S3g). These data further confirm the importance of *Tmem59* in gustatory function, and suggest that aging‐related downregulation of *Tmem59* in CVP impairs generation of mature taste receptor cells and diminished the expression of taste transduction genes.

Collectively, we identified a subset of DEGs, including *Tmem59*, between aged and young taste papillae, potentially involved in inflammation, ER stress, taste cell generation, and gustatory function.

### 3.3 Elevated unfolded protein response in aged mucosal cells

**FIGURE 3:**
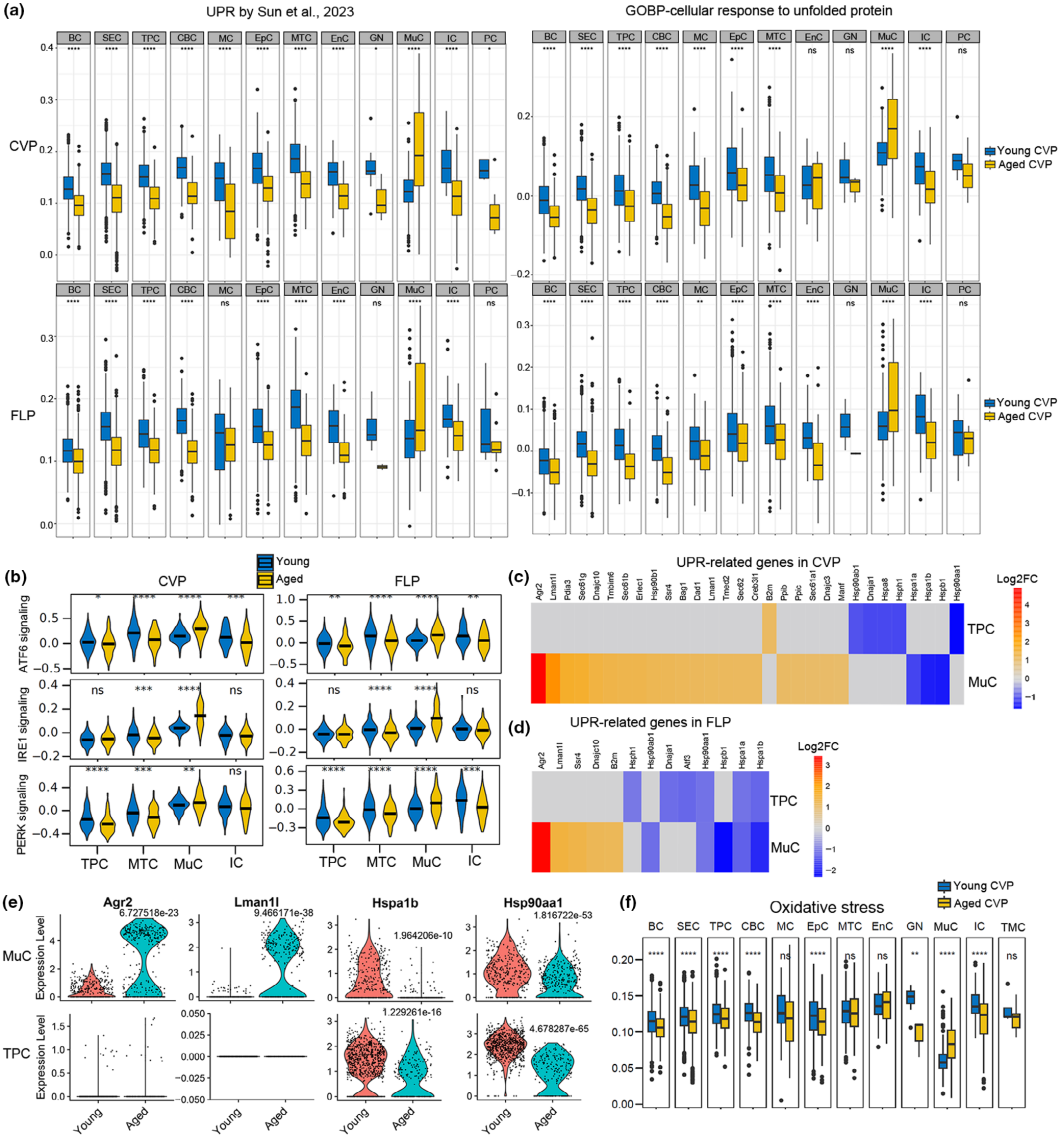
Aging elevates unfolded protein response in mucosal cells. (a) Gene set score analysis showed higher UPR scores in aged mucosal cell (MuC) but not in other cell types of CVP and FLP. (b) Violin plots showing the gene set scores of ATF6, IRE1, PERK signaling pathway in TPC, MTC, MuC, and IC of the young and aged CVP and FLP. (c, d) Heatmaps showing expression levels of UPR‐related genes in aged MuC and TPC of CVP (c) and FLP (d), compared to respective young cell type. (e) Violin plots showing expression of UPR‐related genes *Agr2*, *Lman1l*, *Hspa1b*, *Hsp90aa1* in MuC and TPC of the young and aged CVP. (f) Gene set score analysis of oxidative stress pathways in various cell types of the young and aged CVP. *p < 0.05; **p < 0.01; ***p < 0.001; ****p < 0.0001; ns, not significant (by two‐sided Wilcoxon rank‐sum tests).

Unfolded protein response (UPR) is a homeostatic signaling network that mediates the recovery of endoplasmic reticulum (ER) function, and induces cell apoptosis if adaptation to ER stress fails. In our study, the UPR gene set score was significantly increased in MuC of the aged CVP and FLP compared to young cells, using database either reported by Sun et al., 2023 or GOBP‐Cellular response to unfolded protein (MSigDB: MM6988) (Figure 3a). By contrast, other cell types did not show higher UPR gene set score. We then determined if three branches of the UPR were affected by aging. The ATF6, IRE1, and PERK signaling components were upregulated in MuC of the aged taste papillae (Figure 3b). However, upregulation of UPR branch genes was not observed in MTC, TPC or IC (Figure 3b). In MuC of the aged CVP and FLP, most of the UPR‐related genes were upregulated, while some heat shock proteins were downregulated (Figure 3c,d). Conversely, the expression of most UPR‐related genes was not significantly changed in aged TPC compared to young cells, although heat shock proteins were also downregulated in aged TPC (Figure 3c). Interestingly, the heat shock proteins downregulated in MuC (*Hspa1a*, *Hspa1b*, *Hspb1*) were different from those in TPC (*Hspa8*, *Hsph1*, *Hsp90aa1*) of aged CVP (Figure 3c). In the aged CVP, the expression levels of *Agr2* and *Lman1l* were significantly elevated in MuC compared to young cells, while *Hspa1b* and *Hsp90aa1* were apparently downregulated in aged MuC (Figure 3e). However, no apparent expression changes of *Agr2* or *Lman1l* were found in TPC of the aged CVP compared to young tissue, although *Hspa1b* and *Hsp90aa1* were downregulated (Figure 3e). Hypoxia led to elevated expression of *Agr2* in bronchial epithelial cells, and increased airway MUC5AC hypersecretion (Xu et al., 2019), implying that aged MuC with a higher expression level of *Agr2* may secrete more mucus protein. Meanwhile, aged MuC but not the other cell types showed a higher gene set score of oxidative stress (Figure 3f). Collectively, these data indicate that aging enhances UPR in MuC but not in the other cell types of taste papillae.

### 3.4 Aging‐dependent gene atlas in the circumvallate and foliate papillae

To further identify aging‐dependent genes in the taste papillae, we screened DEGs between the aged and young taste papillae within the GenAge database, and identified a few aging‐dependent genes (Figure S4a–d). In the aged CVP, *Gsta4*, a gene critical for protection against cisplatin ototoxicity (Park et al., 2019), was upregulated in MuC, but downregulated in BC, CBC, EpC, IC, SEC, and TPC (Figure S4a,b,e). This suggests that cell subtypes with *Gsta4* downregulation may be more vulnerable to toxic exposure, while MuC with upregulated *Gsta4* may exert an anti‐toxic function. *Prdx1*, controlling antioxidant function and inflammatory activation (Kim et al., 2022), was upregulated in MuC, and downregulated in BC, CBC, MTC, SEC, TPC (Figure S4a,b,e). Aging‐related *Prdx1* downregulation may increase inflammatory response and decrease resistance to ROS damage in the CVP, while *Prdx1* upregulation in MuC is likely to exert a protective effect against aging. The antioxidant gene *Txn1* was mostly elevated in MuC, and mildly increased in CBC and EC, while *Mt1* was upregulated in BC, CBC, and SEC of the aged CVP. MuC in the aged CVP expressed the highest number of aging‐dependent genes, including 13 upregulated (*Txn1*, *Akt1*, *Cdkn1a*, *Arhgap1*, *Coq7*, *Gpx4*, *Grn*, *Gsta4*, *Insr*, *Mgat5*, *Prdx1*, *Sqstm1*, *Stub1*) and 2 downregulated (*Socs2*, *Gdf15*) ones. This suggests a significant and unique aging‐related influence on MuC. *Mgat5*, a recently reported critical factor in stem cell differentiation (Yale et al., 2023), was mostly upregulated in MuC. Besides, BC and SEC in the aged CVP had 14 (5 upregulated and 9 downregulated) and 12 (6 upregulated and 6 downregulated) aging‐dependent DEGs (Figure S3a,b). In term of the number of aging‐dependent genes, BC, MuC, and SEC are cell subtypes in the CVP that are mostly affected by aging.

In the FLP, BC expressed 8 upregulated (*Mif*, *Txn1*, *Lmna*, *Mt1*, *Gpx4*, *Prdx1*, *Jund*, *Slc25a4*) and 6 downregulated (*Cebpb*, *Trp63*, *Apoe*, *Cisd2*, *Efemp1*, *Myc*) aging‐dependent genes (Figure S4c,d,f). Moreover, CBC and SEC in the aged FLP had respective 11 aging‐dependent genes (Figure S4c,d). These three cell types possessed the most aging‐dependent genes in the aged FLP. MuC in the aged FLP had 1 upregulated (*Mgat5*) and 4 downregulated (*Cebpb*, *Trp63*, *Xpa*, *Apoe*) aging‐dependent genes (Figure S4c,d). In either CVP or FLP, some aging‐dependent genes including *Mgat5*, *Ucp2*, *Myc*, *Pten*, *Trp53* were cell‐type specific, while a few genes such as *Lmna*, *Gsta4*, *Prdx1*, *Cebpb* in the aged CVP, and *Mif*, *Txn1*, *Lmna*, *Cebpb* in the aged FLP were shared by at least four cell subtypes.

To confirm the differential expression of aging‐dependent genes in taste papillae, we immunostained circumvallate sections with antibodies against Lmna and Krt5. Consistent with our scRNA‐Seq data showing upregulation of *Lmna* in aged BC (Figure S4e), Lmna was colocalized with Krt5 and the number of Lmna^+^ cells was higher by 18% ± 4% in the aged CVP compared to young tissue (*p* = 0.0067, Figure S4g,h). Our scRNA‐Seq data also indicated that *Cdkn1a* was upregulated in the aged CVP (Figure S4e), consistent with an increased number of Cdkn1a^+^ cells by 92% ± 15% in the aged CVP compared to young tissue (*p* < 0.0001, Figure S4i,j).

Collectively, we identified aging‐dependent DEGs in the taste papillae, revealing that MuC, BC, CBC, and SEC are significantly influenced by aging.

### 3.5 Aging‐related DEGs in taste papillae are associated with oral diseases

Next, we investigated whether DEGs between the aged and young taste papillae were related to oral diseases by screening the DisGeNET database. We found that *B2m*, a gene related to mouth dryness, was upregulated in multiple cell types of the aged CVP and FLP (Figure S5a). Elevated salivary *B2m* levels have been observed in patients with oral lichen planus compared to healthy group (Nosratzehi et al., 2020), and increased B2m secretion activated fibroblast, contributing to the cardiac repair postischemia (Molenaar et al., 2021). Thus, aging‐induced *B2m* upregulation in BC, CBC, EpC, MuC, SEC, and TPC in both CVP and FLP may affect salivary function and stem cell differentiation. Two heat shock proteins *Hspa8* and *Hspb1* were globally downregulated in most cell types of the aged taste papillae (Figure S5b). Stabilizing *Hspa8* expression delays cell senescence (Zhang, Qiao, et al., 2022). This suggests that global downregulation of *Hspa8* and *Hspb1* in taste papilla cells is potentially associated with cell senescence and death, affecting both basal/progenitor cells and taste receptor cells. We also observed widespread upregulation of *H2‐D* /*H2‐K* (orthologous to human genes *HLA‐A*, *HLA‐B*) in six and seven cell subtypes of the aged CVP and FLP, respectively (Figure S5c). *H2‐K* overexpression decreased proliferation of neural stem cells and impaired neurogenesis (Lin et al., 2021). Therefore, upregulation of *H2‐D/K* in BC, CBC, and TPC may affect their differentiation into taste cells. Moreover, MuC possessed the largest number of DEGs associated with oral diseases, further highlighting the important role of MuC in taste papilla aging. Collectively, screening with DisGeNET database identifies several aging‐related DEGs in the taste papillae that are associated with oral diseases.

### 3.6 Aging promotes the cellular interaction between basal cell and mature taste cell

**FIGURE 4:**
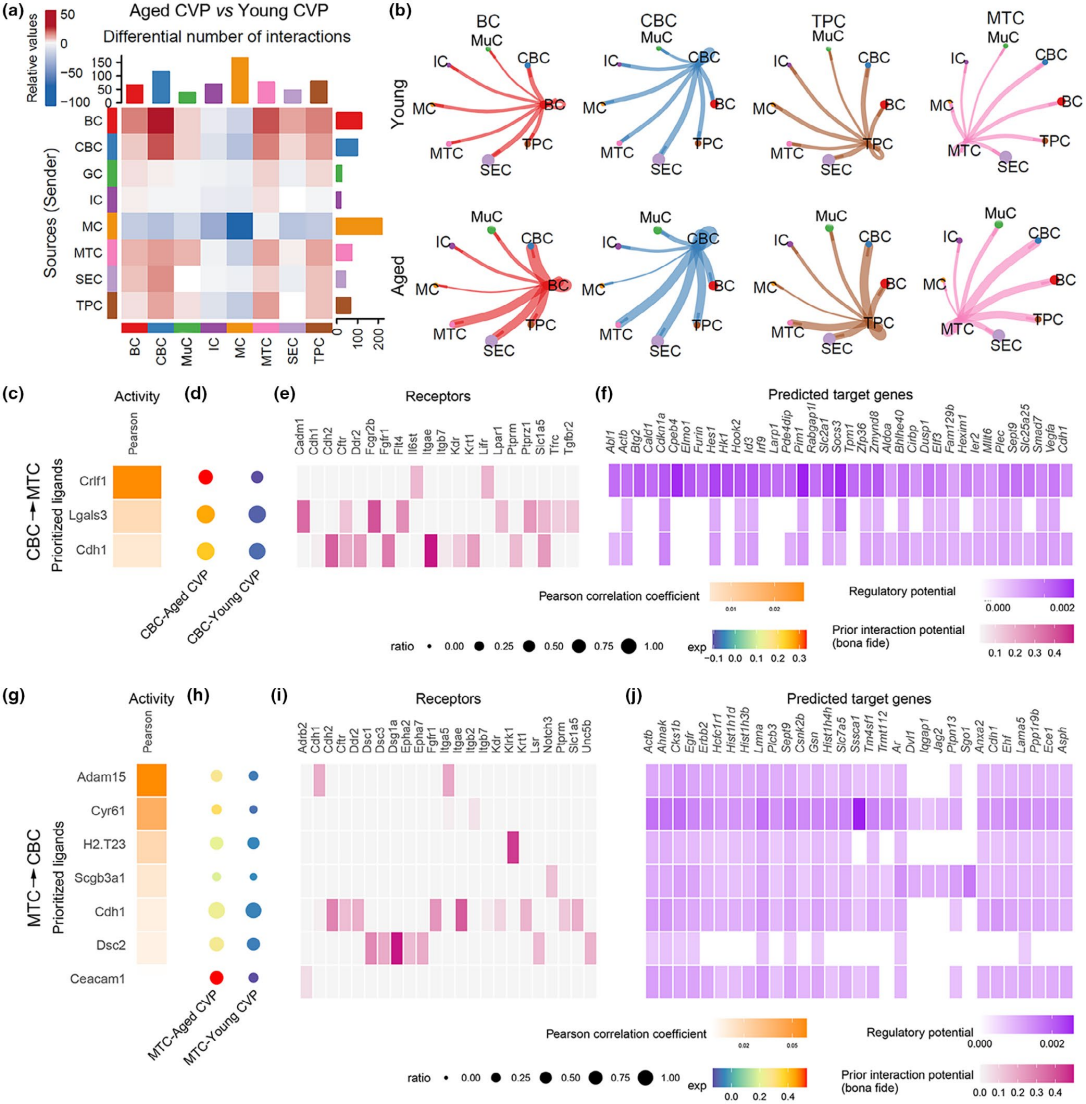
Aging promotes intercellular communication between basal/progenitor cell and mature taste cell. (a) CellChat showing changes in intercellular communications between aged and young cell types in the CVP. (b) Circle plot by CellChat showing the interaction strength outgoing from BC, CBC, TPC, and MTC in the young and aged CVP. The edge width represented the communication weight, and circle size was proportional to the number of cells in each cell group. (c) Upregulated top‐ranked ligands inferred to regulate MTC by CBC in the aged CVP according to NicheNet. (d) Dot plot showing the expression percentage (dot size) and intensity (dot intensity) of top‐ranked ligands in young and aged CBC. (e) Ligand‐receptor pairs showing interaction between aged MTC and CBC ordered by ligand activity (c). (f) Heatmap showing regulatory potential of upregulated top ranked ligands (c) to the downstream target genes upregulated in aged MTC. (g–j) Heatmap and dot plot showing the activity (g) and expression (h) of the top ranked ligands upregulated in MTC of the aged CVP, interaction potential of ligands expressed by aged MTC to receptors in aged CBC (i) and regulatory target genes in aged CBC (j).

To elucidate whether intercellular communication was affected by aging, we used CellChat to analyze the interactions among different cell types in young and aged taste papillae. The analysis revealed that aging enhanced intercellular communication between basal/progenitor cell and mature taste cell of the aged CVP, including interactions between BC/CBC/TPC to MTC, and vice versa (Figure 4a,b). NicheNet analysis showed that CBC in the aged CVP had elevated ligand activity of *Crlf1*, with higher expression levels compared to that in young CVP (Figure 4c,d). *Crlf1* bound to *Lifr*, which was expressed in MTC (Figure 4e). The downstream target genes upregulated in aged MTC to *Crlf1*/*Lifr* pair functioned in inflammatory response (*Abl1*, *Elmo1*, *Tpm1*, *Aldoa*, *Bhlhe40*, *Cirbp*, *Elf3*, *Smad7*), cell cycle arrest (*Btg2*, *Cdkn1a*, *Dusp1*), cellular senescence (*Cpeb4*, *Ier2*), maintenance of stem cell quiescence (*Hes1*), cell death (*Slc2a1*, *Zfp36*, *Dusp1*), and innate immunity (*Hk1*, *Irf9*, *Pim1*) (Figure 4f). Reciprocally, the active ligand *Adam15* upregulated in aged MTC bound to *Itga5* expressed in CBC (Figure 4g–i). In aged CBC, the downstream target genes associated with this ligand/receptor pair functioned in cell proliferation (*Cks1b*, *Egfr*, *Csnk2b*, *Ptpn13*), inflammation (*Erbb2*, *Anxa2*), and epithelial homeostasis (*Ehf*) (Figure 4j). Furthermore, the ligand/receptor pairs and target genes in BC/MTC and TPC/MTC showed similar patterns as in CBC/MTC (Figure S6). Collectively, these data indicate that aging enhances the intercellular communication between basal/progenitor cell and mature taste cell, predominantly influencing cell proliferation and death, inflammation, and immune response.

### 3.7 Ribosome proteins are hubs in the transcriptional factor network

**FIGURE 5:**
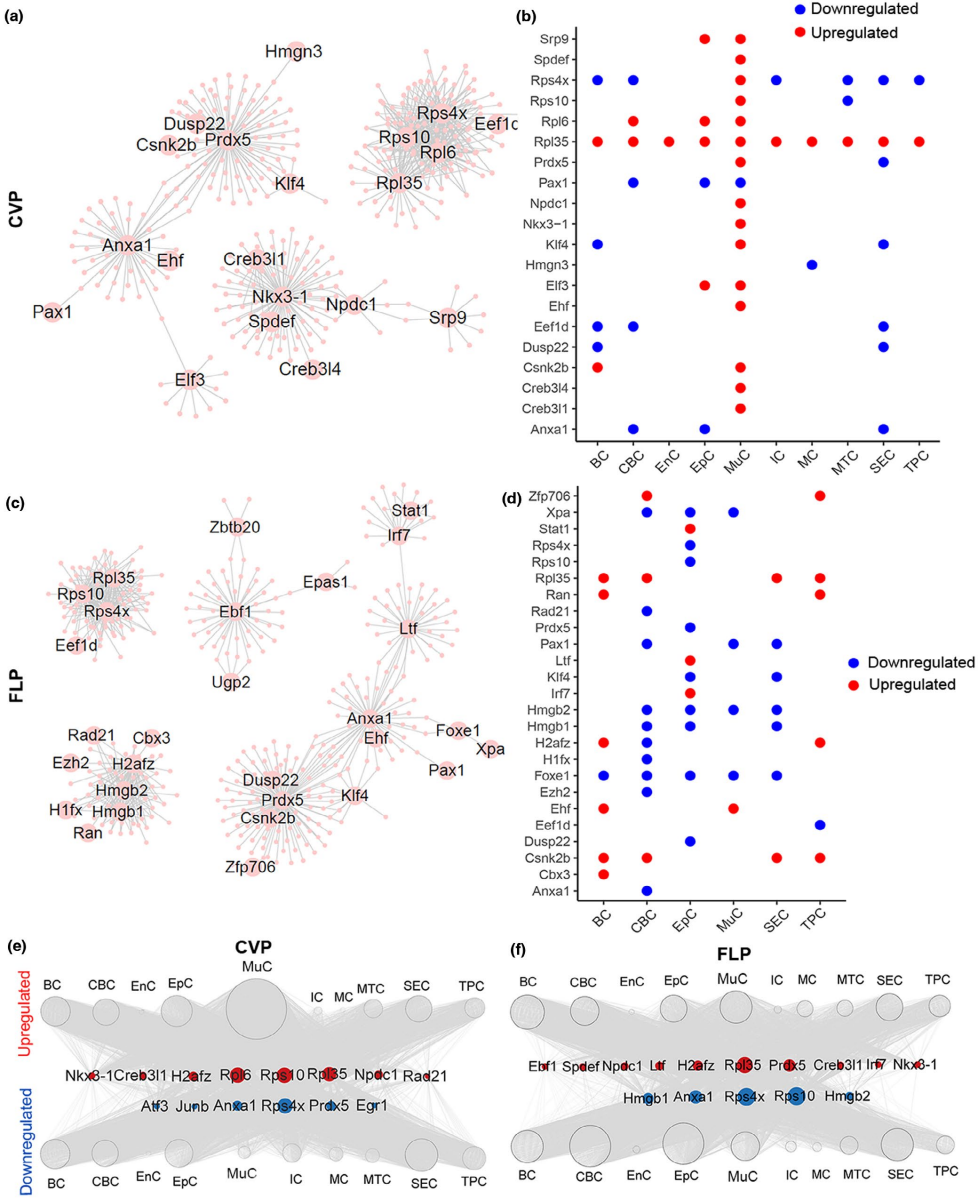
Master regulator related to taste papilla aging in the transcriptional network. (a, c) Network visualization of potential transcriptional regulation in the CVP (a) and FLP (c). Node size was proportional to the number of directed edges. (b, d) Dot plot showing upregulated (red) and downregulated (blue) core regulatory TFs shared by at least two cell types between the aged and young CVP (b) and FLP (d). (e, f) Network visualization of upregulated and downregulated core regulatory TFs in various cell types between aged and young CVP (e), and FLP (f). Node size was positively correlated with the number of edges. Red nodes, upregulated TFs; blue nodes, downregulated TFs.

We then determined the differentially expressed core regulatory transcription factors (TFs) in aged taste papillae compared to young ones. Using SCENIC analysis, we constructed transcriptional network and identified several core TF hubs potentially contributing to taste papilla aging (Figure 5a,c). These regulatory hubs included a set of ribosome proteins including *Rps10*, *Rps4x*, *Rpl35*, *Rpl6*, and other genes such as *Prdx5*, *Anxa1*, *Hmgb1/Hmgb2*. In the aged CVP, *Rps4x* was downregulated in BC, CBC, IC, MTC, SEC, and TPC, while *Rpl35* was upregulated in all cell subtypes (Figure 5b). The majority of core TFs were upregulated in aged MuC (Figure 5b). In the aged FLP, *Rps4x*, and *Rps10* were downregulated only in EpC, whereas *Rpl35* was upregulated in BC, CBC, SEC, TPC (Figure 5d). Transcriptional regulatory network analysis demonstrated preferential transcriptional regulons in each cell type of CVP and FLP (Figure 5e,f), further supporting massive regulons by TF hubs in different cell types. Besides ribosome proteins, downregulated *Pax1* in CBC, EpC, MuC, *Anxa1* in CBC, EpC, upregulated *Prdx5* in MuC of aged CVP, and downregulated *Hmgb1/2* in CBC, EpC, SEC, *Prdx5* in EpC, *Foxe1* in BC, CBC, EpC, MuC, SEC, upregulated *Csnk2b* in BC, CBC, SEC, TPC of the aged FLP were hubs in the TF network (Figure 5b, d–f). *Rps10* was known to be a binding site for scanning of cell cycle genes (Sehrawat et al., 2019). Ribosomal family protein *Rps4x* was associated with CD4^+^ T cell activation, and potentially served as a biomarker for the diagnosis and treatment of Alzheimer's Disease (Y. Wang et al., 2023). *Rpl35* was associated with oxidative phosphorylation and mitochondrial dysfunction (Syn et al., 2017). Thus, the differential expression of these ribosome proteins in taste papillae may cause some disease phenotype and affect immune cell response and mitochondrial function. *Hmgb1* deletion exaggerates inflammatory response, including inflammatory cell infiltration and higher levels of inflammatory mediators (Mao et al., 2022). *Hmgb2* contributes to the transition from the quiescent to the proliferative state of stem cells (Kimura et al., 2018). Therefore, downregulation of *Hmgb1/2* in the aged FLP may correlate with inflammatory activation and maintenance of stem cell's quiescent state. Collectively, our data identify critical TFs potentially regulating taste papilla aging.

### 3.8 Aging‐associated transcriptional changes throughout taste cell maturation

**FIGURE 6:**
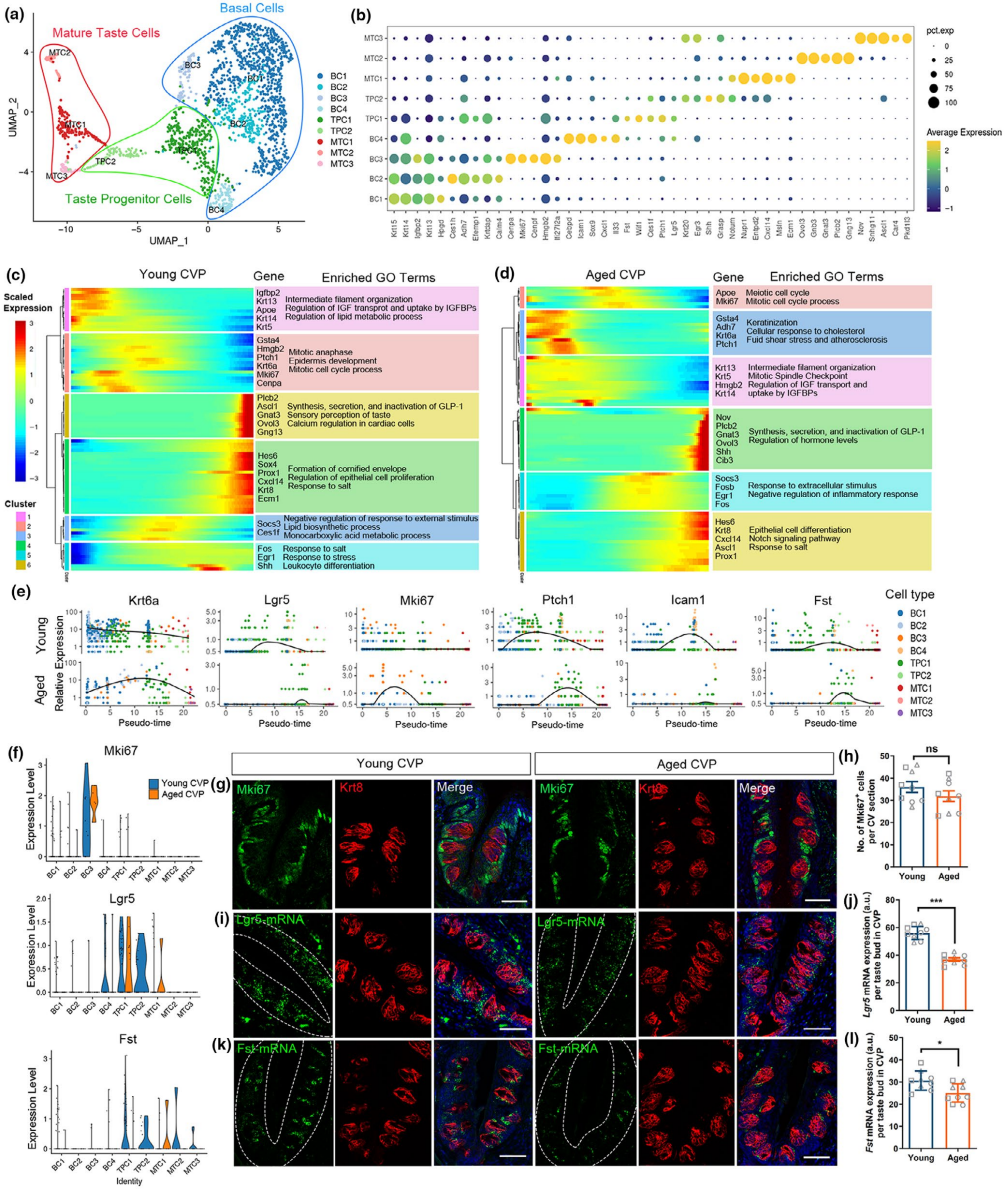
Differential gene expression in taste cell maturation between the aged and young CVP. (a) UMAP plot showing the subclustering of BC, TPC, and MTC. (b) Dot plot showing the expression of five molecular markers for BC, TPC and MTC subclusters. Dot size reflected percentage of cells in a cluster expressing each gene, and dot color represented expression level. (c, d) Six clusters of pseudotime‐dependent genes with dynamic expression patterns from BC to TPC and MTC in the young (c) and aged CVP (d), plotted across pseudotime as heatmap. Blue indicated low level and red indicated high level of expression. Representative genes in each cluster and enriched gene ontology (GO) terms were shown on the right. (e) Differential expression patterns of gene selected from subclusters along pseudotime trajectory in the young and aged CVP. (f) Violin plots of *Mki67*, *Lgr5*, and *Fst* expression levels in various types of BC, TPC, MTC from the young and aged CVP. (g–l) Confocal images and quantification of Mki67^+^ cells (g, h), and Lgr5‐mRNA^+^ (i, j), Fst‐mRNA^+^ (k, l) signal intensity in the young and aged CVP. *n* = 9 sections for each group. The statistical significance was determined by unpaired *t* test. ns, not significant, *p < 0.05, ****p < 0.0001. Scale bars, 50 μm.

To investigate how aging altered transcriptomic features in taste cell maturation from basal and progenitor cells, we next examined transcriptional changes in mature taste cells throughout their developmental progression. We first reclustered BC, TPC and MTC in the young and aged taste papillae (Figures 6a, S7a). UMAP plots showed *Krt14* ^+^ BC1, *Efemp1* ^+^ BC2, *Mki67* ^+^ BC3, *Sox9* ^+^ BC4, *Lgr5* ^+^ TPC1, and *Shh* ^+^ TPC2 as well as *Entpd2* ^+^ MTC1, *Gnat3* ^+^ MTC2, and *Snap25* ^+^ MTC3 in taste papillae (Figure S7c). We identified previously unknown markers, such as *Hpgd*, *Ces1h*, *Hmgb2*, *Cebpd* for BC, *Serpine2*, *Sox4* for TPC, and *Ecm1*, *Ovol3*, *Nov* for MTC (Figures 6b, S7b). Peudotime analysis by Monocle2 showed two developmental axes in the maturation of taste cells in the young CVP, while all three types of MTCs followed the same trajectory in the aged CVP (Figure S7d). To determine which genes regulated the progression of taste cell maturation along pseudo‐timeline, we performed hierarchical clustering of the genes whose expression varied with pseudotime between the aged and young CVP. In either young or aged CVP, a subset of genes, including *Mki67*, were transiently upregulated and afterwards downregulated, implicating the involvement in the cell cycle process (Figure 6c,d). Genes downregulated as the pseudo‐timeline progressed were related to intermediate filament organization in either young or aged CVP, while lipid metabolic process were unique to the young CVP and mitotic spindle checkpoint was relevant in the aged CVP (Figure 6c,d). Genes upregulated along the pseudotime in the young CVP were associated with sensory perception of taste and regulation of epithelial cell proliferation, whereas in the aged CVP, they were related to epithelial cell differentiation and Notch signaling (Figure 6c,d). Genes associated with cell cycle process, such as *Krt6a*, *Mki67*, *Ptch1*, and progenitor cell marker like *Icam1* and *Lgr5*, showed differential expression pattern along pseudo‐timeline of taste cell maturation (Figure 6e). *Mki67*, *Lgr5*, *Fst* were enriched in BC, TPC and MTC, and were differentially expressed between the aged and young CVP according to scRNA‐Seq data (Figure 6f). Immunostaining data showed that the number of Mki67^+^ cells per CVP section was decreased by 11% ± 6% in aged tissue compared to young one (*p* = 0.2443, Figure 6g,h). Meanwhile, RNAscope data indicated that intensity of *Lgr5*‐mRNA^+^ and *Fst*‐mRNA^+^ signal was reduced by 34% ± 2% (*p* < 0.001) and 18% ± 4% (*p* = 0.0125) in the aged CVP, respectively (Figure 6i–l). *Lgr5* marks TPCs in posterior tongue (Yee et al., 2013), and *Fst* is important for the maintenance of taste buds and their stem/progenitor cells (Beites et al., 2009). These potentially suggest that downregulation of *Lgr5* and *Fst* in TPC of the aged CVP may impair the differentiation of taste stem/progenitor cells.

Then, we cultured taste organoids from single cells isolated from young and aged CVP, following our established protocol (Ren et al., 2017). The growth rate and differentiation conditions in organoids reflected the stemness of taste stem/progenitor cells and maturation of taste cells. At Day 3 and Day 7 post culture under identical conditions, the size of organoids from the aged CVP was significantly reduced by 60% ± 3% (*p* < 0.001) and 42% ± 3% (*p* < 0.0001), compared to those from young tissue (Figure S7e,f). At Day 14, organoids were collected for immunostaining with antibodies against mature cell markers, including PLCβ2, Car4, and pan‐taste cell marker Krt8. The number of PLCβ2^+^ Type II, Krt8^+^, Car4^+^ Type III taste cells, were significantly reduced by 75% ± 2% (*p* < 0.0001), 35% ± 9% (*p* = 0.0027), 59% ± 4% (*p* = 0.0002) in aged organoids, respectively (Figure S7g,h). This reduction was potentially related to decreased expression of Lgr5 and other TPC markers in the aged CVP.

Collectively, we identified critical genes potentially contributing to the aging‐related discrepancy in taste cell maturation.

### 3.9 Elevated expression of Muc5b in aged taste papillae

To further explore the role of MuC in the aged CVP, we subdivided the MuCs into three major subtypes: *Sval2* ^+^, *Muc5b* ^+^, and *Crabp2* ^+^ cells (Figure S8a). Since Crabp2 was primarily expressed in FLP but not CVP tissue, we excluded this cell subcluster from the following analysis. The expression level of *Muc5b* was obviously increased in aged taste papillae (Figure S8b). We then conducted PAS staining to locate Von Ebner's glands, also known as gustatory glands, which reside in the moats surrounding the CVP and FLP. PAS staining data indicated a reduction in the number of Von Ebner's glands in aged CVP sections, suggesting fewer gustatory glands in aged mice (Figure S8c). This finding was corroborated by our analysis data in Figure S5, which showed an increase in expression levels of genes associated with mouth dryness in aged mice. Immunostaining data showed that Muc5b^+^ cells were located in the lamina propria near the CVP and FLP, not consistent with the PAS staining positive region (Figure S8c,d). The number of Muc5b^+^ cells was notably higher in aged taste papillae (Figure S8d,e). To better understand the role of MuC in taste cell generation, we combined the *Mub5b* ^+^ and *Sval2* ^+^ MuCs with MTC, TPC, and BC, and analyzed intercellular communication using CellChat. The data revealed that Thbs1 signaling from *Muc5b* ^+^ cells to basal and progenitor cells was most abundant in aged taste papillae (Figure S8f,g). Among the ligand‐receptor pairs involved in Thbs1 signaling, Thbs1‐Sdc4/Sdc1 pairs exhibited the highest communication probability, particularly in *Muc5b* ^+^ cell to BC1 communication (Figure S8f,g). Thus, aging increased Muc5b expression in taste papillae, and intercellular communication between *Muc5b* ^+^ cell and basal cell is potentially mediated by Thbs1 signaling.

### 3.10 *Ovol3* marks type II taste cell

For a precise analysis of mature taste receptor cells, we classified Type II and III taste receptor cells into 7 subclusters (Figure S9a). Type III MTCs contains two subclusters, and Cluster 2 cells expressed sour receptors *Otop1* and *Pkd1l3* (Figure S9b). GO enrichment analysis revealed that genes highly enriched in Cluster 2 cells were involved in sensory perception of sour taste and synaptic vesicle cycle (Figure S9c). Cluster 0 cells belonging to Type III cells highly expressed *Miat*, and were involved in neuronal differentiation and MAPK cascade (Figure S9a–c). Type II taste cells included five subclusters, and Cluster 1 cells expressed sweet and umami receptors, such as *Tas1r1*, *Tas1r2*, *Tas1r3*. We also found that Cluster 1 cells functioning in sensory perception of sweet, bitter and umami taste expressed *Ovol3* and *PLCβ2*, and the latter is typical marker for Type II taste cell (Figure S9c). FeaturePlot blend indicated that *Ovol3* was coexpressed with *PLCβ2*, but not *Car4* (Figure S9d), suggesting *Ovol3* marks Type II but not Type III taste cells. RNAscope and immunostaining data further confirmed the colocalization of *Ovol3* and PLCβ2 in the CVP, while Car4^+^ Type III taste cells did not express *Ovol3* (Figure S9e,f). Cluster 5 cells expressed *Trpm5*, *Gnat3*, and bitter receptor *Tas2r*, indicating their involvement in sensory perception of bitter taste (Figure S9c). GO enrichment analysis also suggested that Cluster 6 cells contributed to cellular response to salt, suggesting these cells expressed salt receptor (Figure S9c). Collectively, we set up new subclusters of Type II and III taste cells, and identify *Ovol3* as a new marker for Type II taste cell.

## 4 DISCUSSION

In this study, we constructed a single‐cell transcriptomic atlas of mouse taste papilla aging. We identified aging‐regulated DEGs in multiple cell subtypes of taste papillae, and confirmed that loss of *Tmem59* impaired generation of taste receptor cells and reduced expression of taste transduction genes in the CVP, and reduced gustatory sensitivities to tastants. Differential expression pattern of several genes such as *Lgr5*, *Krt6a*, *Icam1* potentially led to aging‐related difference in taste cell maturation. Collectively, this work displays critical regulators for taste papilla aging, providing ideal candidates contributing to aging‐related gustatory alteration.

A recent work identified aging‐related DEGs on the labellar tissue of young (10 day) and aged (40 day) flies (Brown et al., 2024). Among these DEGs, upregulated genes were associated with translation and ribosome biogenesis, while downregulated genes were involved in the perception of chemical stimuli. Accordingly, authors suggested that aging‐related increase in translation possibly functioned as a compensatory mechanism for reduced chemosensory expression. In sweet taste neurons, several OBPs were downregulated with age, possibly suggesting a direct involvement in diminished taste, while aging‐upregulated gene including Arc1 involved in synaptic communication, as well as Listericin functioned in the innate immunity were identified. Our scRNA‐Seq data showed that upregulated genes in aged taste papillae functioned in ribosome biogenesis and assembly (Figure S2), consistent with the functions of upregulated genes in labellar tissue of aged flies. However, the downregulated genes in aged taste papillae were associated with protein folding (Figure S2), which was not identified in aged labellar tissues. This is probably due to the differential taste tissues from different species used for transcriptional analysis.

A few DEGs were shared by multiple cell subtypes in the aged taste CVP. Among upregulated genes, *mt‐Nd4l*, *Xist*, *mt‐Atp8* were three top genes mostly shared by different cell types. Both *mt‐Nd4l* and *mt‐Atp8* are located in mitochondria, and involved in ATP synthesis coupled electron transport. Other mitochondrial genes such as *mtNd5*, *mt‐Co1* were also upregulated in multiple cell types. By contrast, several mitochondrial genes such as *mt‐Co3*, *mt‐Atp6*, *mt‐Cytb*, *mt‐Nd2* were downregulated in the aged CVP. Previous report showed that mitochondrial dysfunction was involved in oocyte aging, with decreased expression of *mt‐Nd2*, *mt‐Nd3*, *mt‐Nd4*, *mt‐Nd4l* and *mt‐Nd5* (Zhang et al., 2019). However, *mt‐Nd4l* was apparently upregulated in the aged taste papillae, suggesting the potential mitochondrial dysfunction by aging in the taste papillae is not associated with *mt‐Nd4l*. *Mt‐Co3* (*Cox3*), a globally downregulated gene in multiple cell types of the aged CVP, is critical in mitochondrial electron transport. *Mt‐Co3* as well as other mitochondrial genes such as *mt‐Co1*, *mt‐Co2*, *mt‐Nd1* and *mt‐Nd3* were found to be involved in senescence of islet β‐cells and highly expressed in the pancreas of aged mice (Zheng et al., 2021). This is consistent with our finding that a few mitochondria genes were upregulated in the aged taste papillae. Since function of upregulated genes in the aged taste papillae was associated with oxidative phosphorylation and aerobic respiration, the byproducts from these processes such as ROS may accumulate in aged tissues (Jang et al., 2018). The higher expression of *mt‐Nd4l* in the aged CVP may enhance the activity of the aerobic oxidative respiratory chain complexes I and III, and then increase ROS production. Direct exposure to ROS intensifies oxidative stress, and ultimately damages cells in the aged taste papillae. On the other hand, downregulation of *mt‐Nd2* and *mt‐Co3* may lead to dysfunction of mitochondrial respiratory chain complex I and IV, which may reduce the rate of ATP production (Herst et al., 2017). ATP is a critical neurotransmitter in taste perception (Kinnamon & Finger, 2022). Thus, downregulation of mitochondrial genes in the aged taste papillae may impair taste function. However, the specific function of mitochondrial genes in taste papilla aging needs further investigation.

Goblet cell (GC) is a specialized epithelial cell type that line multiple mucosal surfaces including small intestine and colon, and have a role in barrier maintenance through the secretion of mucus (Knoop & Newberry, 2018). Besides, GC functions in innate immunity by secreting chemokines and cytokines (Yang & Yu, 2021). In the current study, we identified a group of Muc5b^+^/Tff2^+^ cells that are analogous to GCs, and we named this cell cluster as mucosal cell (MuC). Interestingly, MuC showed higher UPR gene set score with aging, contrary to other cell types in the taste papillae (Figure 3). The expression levels of UPR genes in aged versus young MuC were reverse compared to those in other cell types. All these data imply that MuC may exert a unique role in taste papilla aging. The proportion of MuC in the aged CVP was higher than in young tissue. Meanwhile, the gene set score of either chemokine or cytokine was significantly higher in aged MuC compared to young cell (data not shown). These data suggest that aging may aggravate immune response in MuC. Protein folding process produces reactive oxygen species, and causes oxidative stress, which disrupts the correct protein folding and leads to ER stress (Chong et al., 2017). This is validated by our findings that gene set score of oxidative stress was significantly increased exclusively in aged MuC but not in other cell types, compared to young cells (Figure 3). CellChat showed that *Muc5b* ^+^ cells communicated with basal and progenitor cells in aged taste papillae by Thbs1 signaling. However, the specific functions of *Muc5b* ^+^ cells in taste papilla aging required further investigation. Further experiments using transgenic animal models will be conducted to explore the role of *Muc5b* ^+^ cells in taste cell maturation and gustatory function.

In summary, this work shows the molecular and cellular features for taste papilla aging, potentially facilitating to reveal pathogenesis of aging‐related alteration in taste perception.

## AUTHOR CONTRIBUTIONS

Conceptualization, W.R., H.L. and Y.Y.; data curation, W.R., W.L., X.C., S.W., B.C., T.W., F.L., T.L., Y.X., Z.X. and Z.W.; formal analysis, W.R., W.L., X.C. and S.W.; funding acquisition, W.R. and Y.Y.; investigation, W.R. and Y.Y.; methodology, W.R., W.L., X.C. and S.W.; project administration, W.R., H.L. and Y.Y.; software, W.R., W.L., X.C. and S.W.; supervision, W.R., H.L. and Y.Y.; validation, W.R. and Y.Y.; writing–original draft, Y.Y.; writing–review and editing, W.R. and Y.Y. All authors have read and agreed to the published version of the manuscript.

## FUNDING INFORMATION

This work was supported by National Natural Science Foundation of China Grants (31,900,714 and 82,271,136 to W.R., 32,070,996 and 32,271,044 to Y.Y.); Science and Technology Commission of Shanghai Municipality (23ZR1409600 and 21,140,900,600 to Y.Y.); Shanghai Municipal Education Commission, the Shanghai Eastern Scholar Program (GZ2022006/SSF158007 to Y.Y.); Shanghai Municipal Health Commission (GWVI‐11.2‐XD09 to Y.Y.); Fudan University, Shanghai Medical College, Young investigator for clinical and scientific research team (to Y.Y.).

## CONFLICT OF INTEREST STATEMENT

None declared.

## Supporting information


Figure S1‐S9.

Table S1.

Table S2.

Table S3.

Table S4.

## Data Availability

All scRNA‐Seq data were deposited in China National GeneBank DataBase (CNGBdb). The deposit number was CNP0005003. All data were available upon request to the lead contact (Y. Yu).
