# Supplementary material for: Single‐cell transcriptomic atlas of taste papilla aging
Source: Aging Cell. 2024 Aug 21;23(12):e14308. doi: 10.1111/acel.14308 (PMC11634696; doi:10.1111/acel.14308)
Supplement: Supplementary file 1 — Figure S1‐S9. [file ACEL-23-e14308-s001.pdf]

Supplementary Figures for

Single-cell transcriptomic atlas of taste papilla aging

Wenwen Ren[1,#,*], Weihao Li[2, 3,#], Xudong Cha[1,#], Shenglei Wang[1,#], Boyu Cai[1], Tianyu Wang[1], Fengzhen Li[1], Tengfei Li[1], Yingqi Xie[1], Zengyi Xu[1], Zhe Wang[1], Huanhai Liu[1,*], Yiqun Yu[2,3,*,†]

[1] Department of Otolaryngology, the Second Affiliated Hospital of the Naval Medical University (Shanghai Changzheng Hospital), Shanghai, China.
[2] ENT Institute and Department of Otorhinolaryngology, Eye & ENT Hospital, Fudan University, Shanghai, 200031, China.
[3] Olfactory Disorder Diagnosis and Treatment Center, Eye & ENT Hospital, Fudan University, Shanghai 200031, China.

[*] Correspondence may be addressed to:
Wenwen Ren: wenwenren@smmu.edu.cn
Huanhai Liu: liuhuanhaiok@smmu.edu.cn
Department of Otolaryngology,
the Second Affiliated Hospital of the Naval Medical University
(Shanghai Changzheng Hospital), Shanghai, China.
and
Yiqun Yu: yu_yiqun@fudan.edu.cn
ENT Institute and Department of Otorhinolaryngology,
Eye & ENT Hospital, Fudan University,
83 Fen Yang Road, Shanghai, CHINA. 200031
Tel: (021) 64377134, Fax: (021) 64377151.

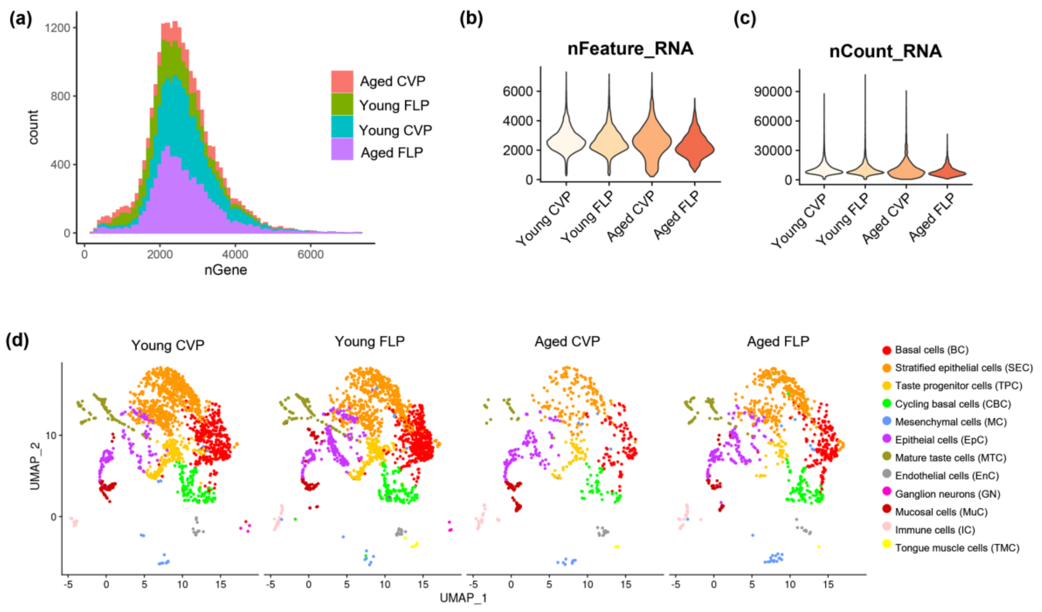

Figure S1. Single cell RNA-Seq of young and aged taste papillae. (a) The number of detected genes and cells in each sample. (b, c) Counts of the number of UMIs and genes detected in each cell. (d) UMAP plot showing integrated data set split into separate plots by tissue type and age. Color note for various cell subtypes were shown on the right.

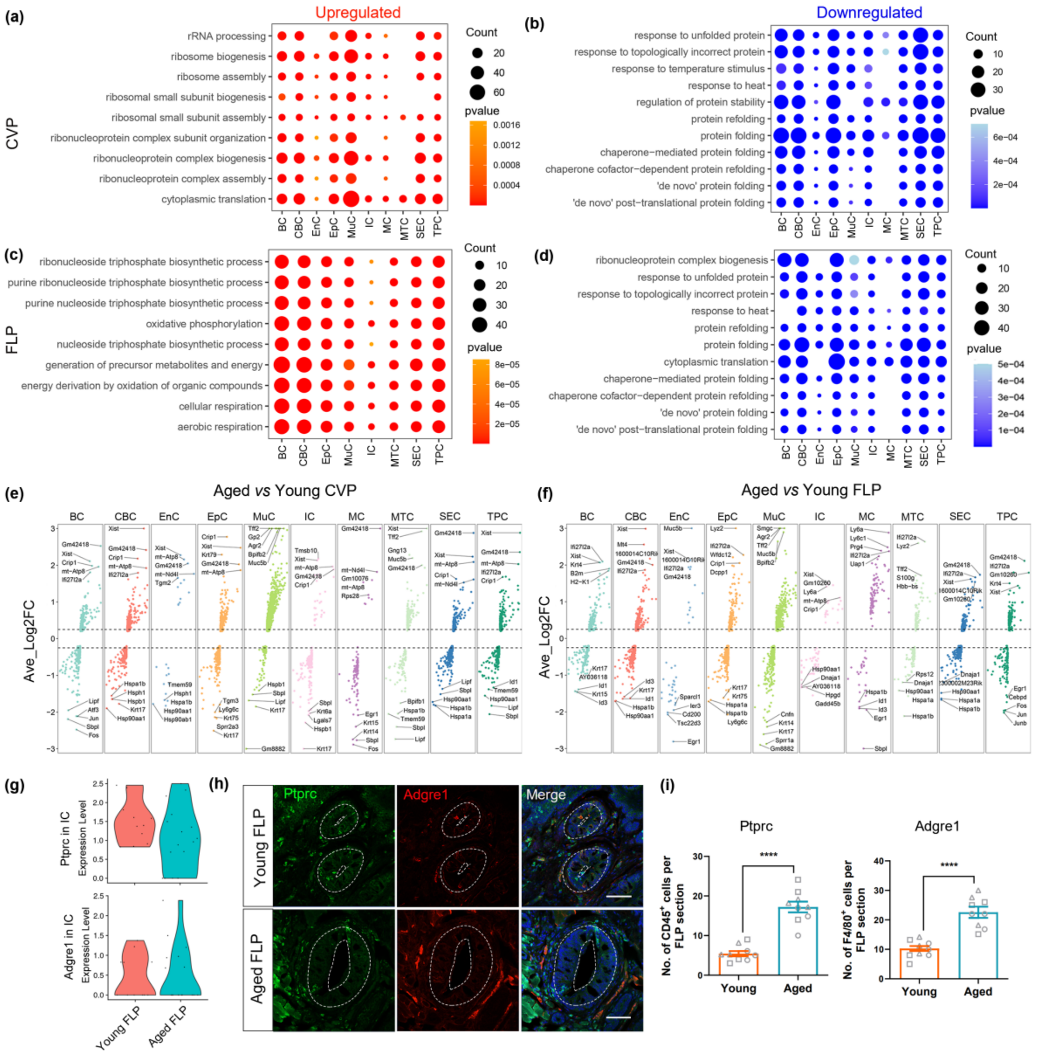

Figure S2. The differentially expressed genes between aged and young taste papilla. (a-d) Representative GO terms of aging-upregulated (red) and -downregulated (blue) genes in different cell types of aged CVP (a, b) and FLP (c, d). “Count” indicated the gene number. The color scales represented the range of p value. (e, f) Plots showing the top-ranked up- and down-regulated genes in different cell types of the aged CVP (e) and FLP (f). (g) Violin plots showing expression of *Ptprc* and *Adgre1* in IC. (h) Confocal images of Ptprc/Adgre1 positive cells in the young and aged FLP. (i) Quantification of CD45 (Ptprc)$^{+}$, F4/80 (Adgre1)$^{+}$ cells in each FLP section. n = 9 sections for each group. The statistical significance was determined by unpaired t test. **** $p < 0.0001$. Scale bars, 50 μm.

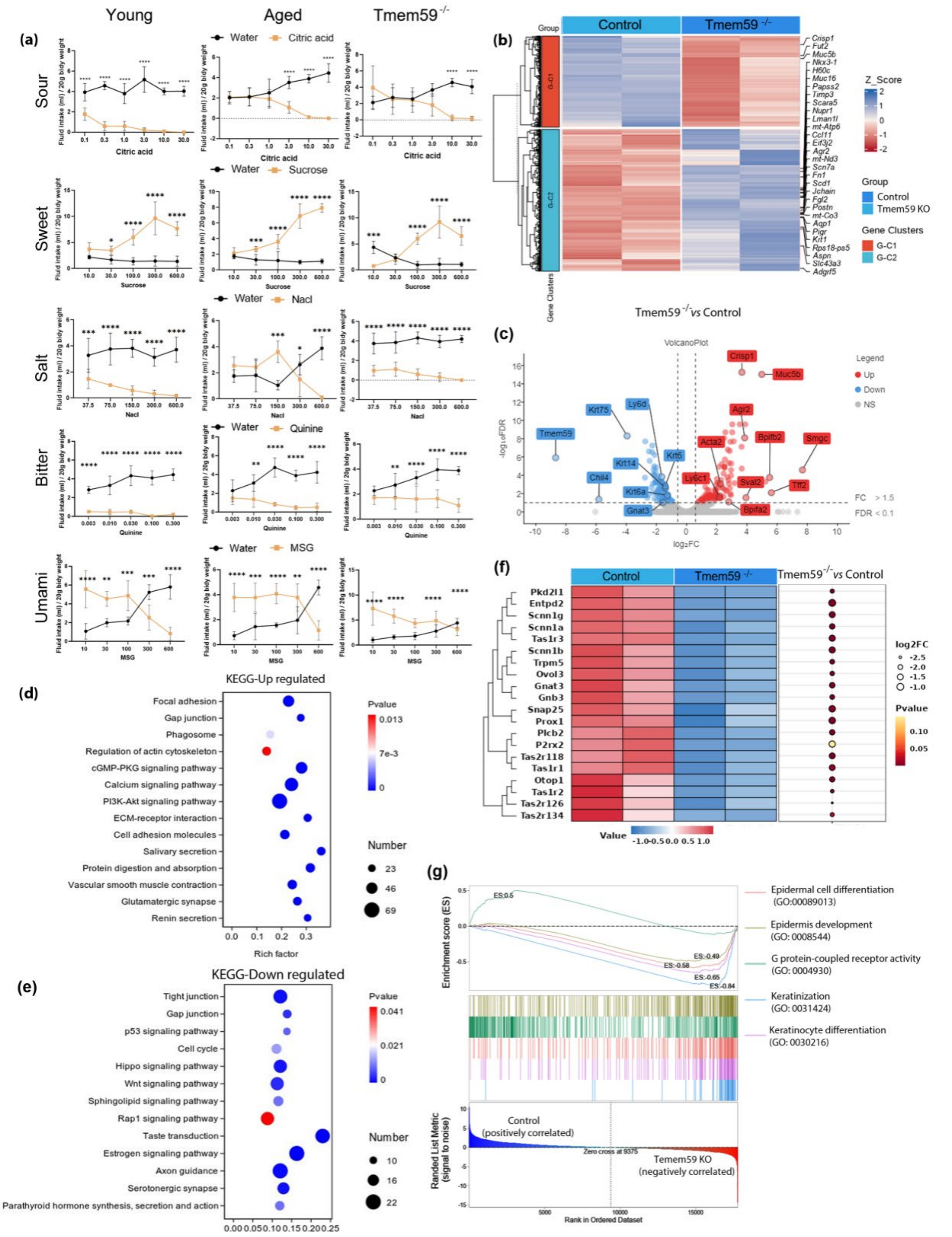

Figure S3. Transcriptional alteration in Tmem59$^{-/-}$ CVP compared to WT tissue. (a) Two bottle preference assay data showing sensitivities to citric acid, sucrose, NaCl, quinine, MSG in young, aged, and Tmem59$^{-/-}$ mice. (b) Heatmap showing the top 20 DEGs between Tmem59$^{-/-}$ and WT CVP. (c) Volcano plot showing representative upregulated and downregulated genes in Tmem59$^{-/-}$ CVP compared to WT tissue. (d, e) KEGG analysis on upregulated (d) and downregulated (e) genes in Tmem59$^{-/-}$ CVP. (f) Heatmap showing significant downregulation of taste transduction genes in Tmem59$^{-/-}$ CVP compared to WT tissue. (g) GSEA indicating upregulated and downregulated GO terms in Tmem59$^{-/-}$ CVP. * $p < 0.05$, ** $p < 0.01$, *** $p < 0.001$, **** $p < 0.0001$; ns, not significant (by unpaired t test).

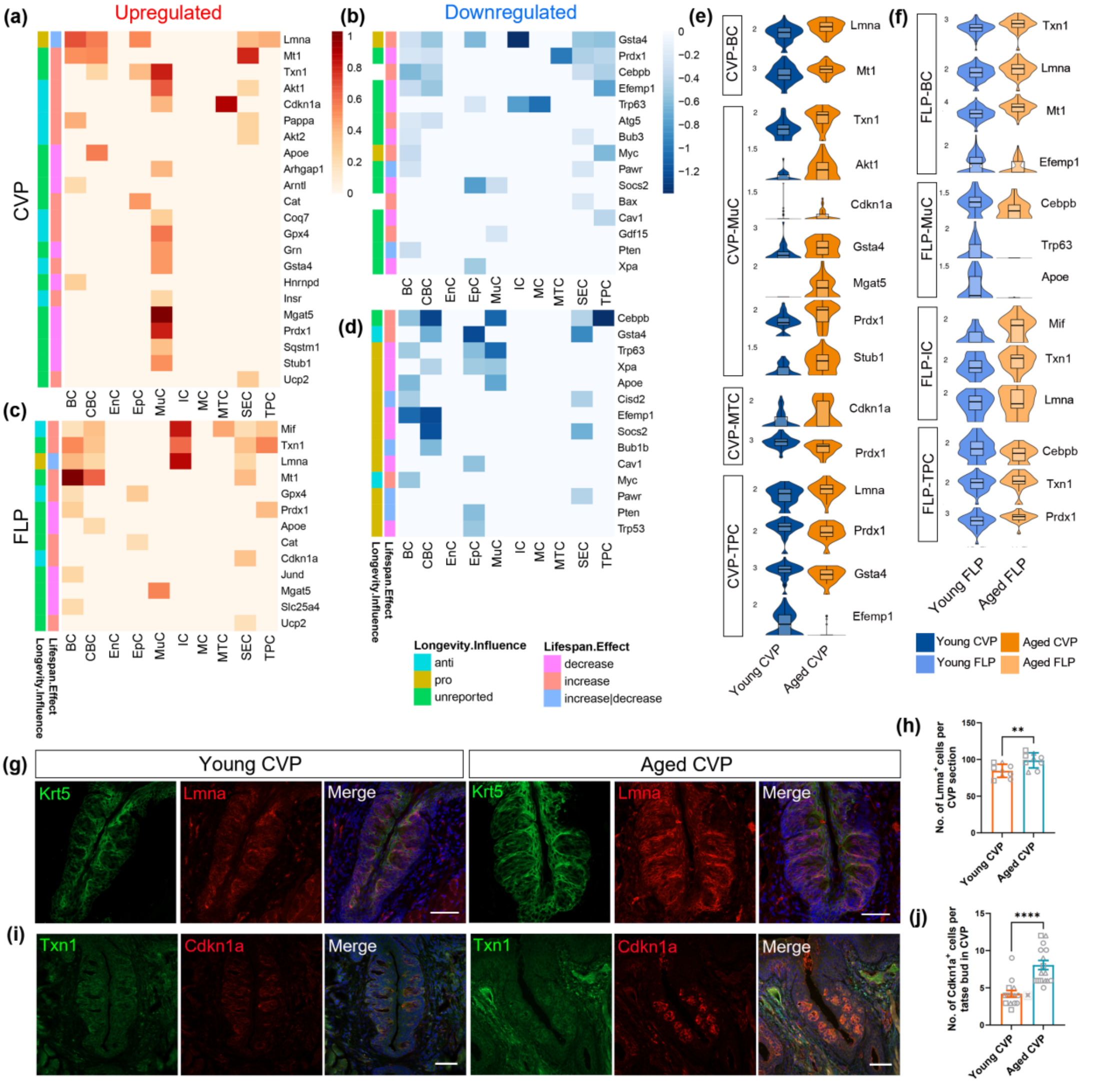

Figure S4. Aging-dependent genes in taste papillae. (a-d) Heatmaps showing aging-dependent genes in CVP (a, b) and FLP (c, d), identified through comparison of DEGs in aged taste papillae with genes in GenAge database. Color scales on the right represented expression level change as Ave_Log2FC(Aged/Young). (e, f) Violin plots showing expression of aging-dependent genes in the young and aged CVP and FLP. (g, i) Confocal images of Krt5, Lmna (g), Txn1, Cdkn1a (i) positive cells in the young and aged CVP. (h, j) Quantification of Lmna^{+}, and Cdkn1a^{+} cells per CVP section in the young and aged tissues. Lmna: n = 9 sections for each group, Cdkn1a: n = 15 sections for each group. The statistical significance was determined by unpaired t test. ns, not significant, *p < 0.05, *** p < 0.001. Scale bars, 50 μm.

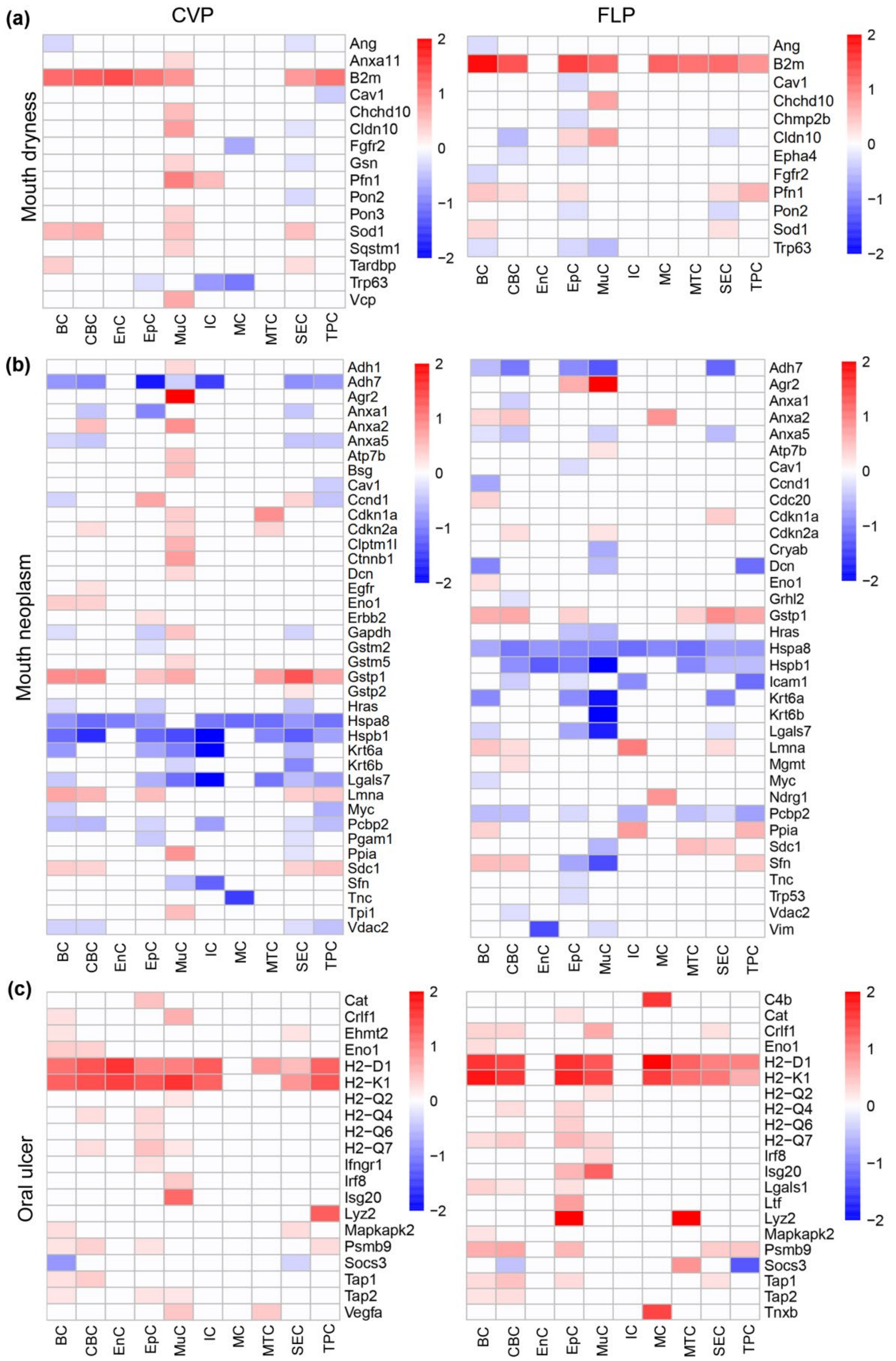

Figure S5. Aging-related DEGs in the taste papillae are associated with oral diseases. Heatmaps of DEGs in the aged taste papillae related with mouth dryness (a), mouth neoplasm (b), and oral ulcer (c). These genes were identified by comparison of DEGs with genes in DisGeNET database. Color scales on the right represented expression level change as Ave_Log$_2$FC(Aged/Young).

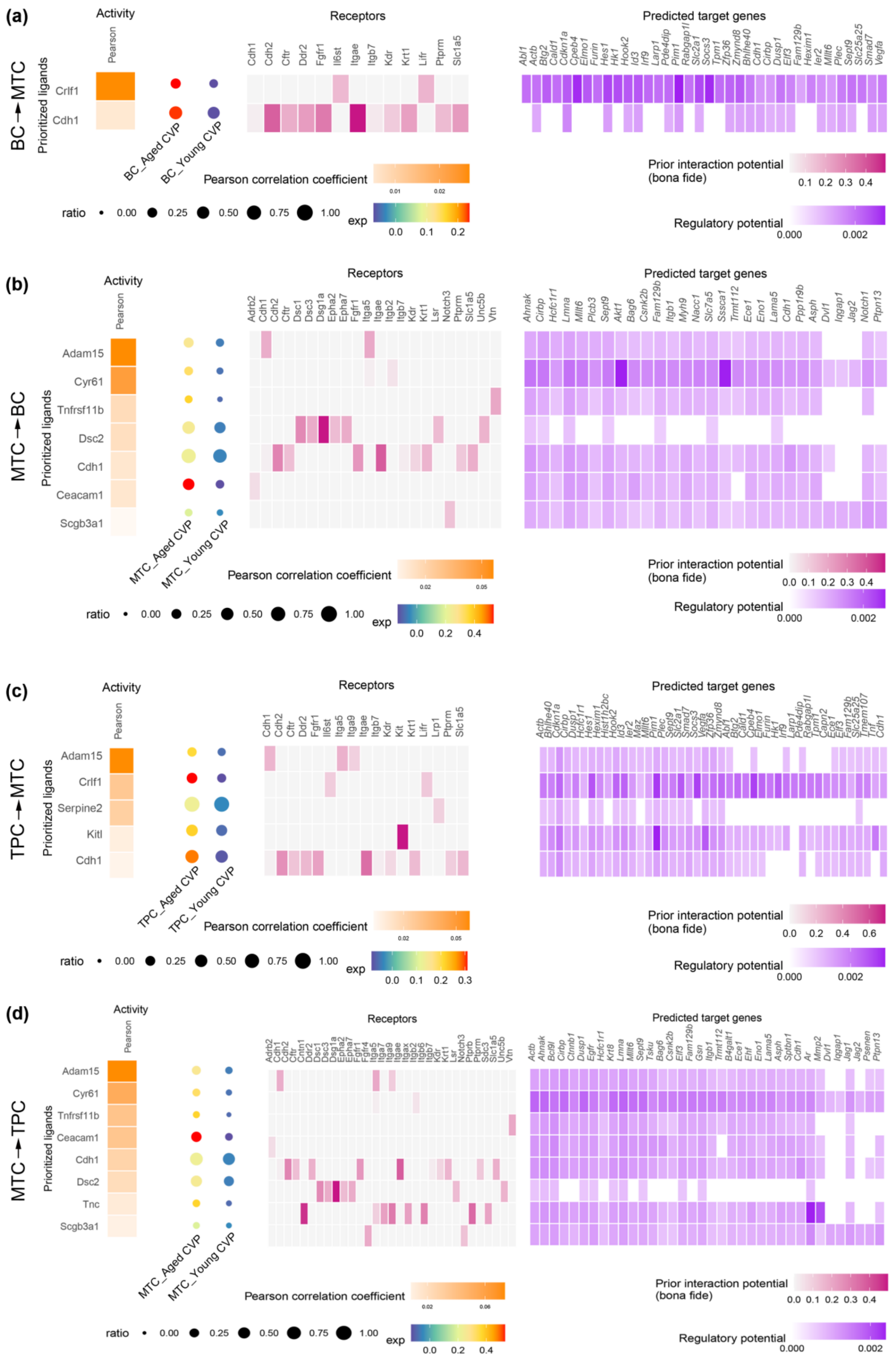

Figure S6. Intercellular communication between BC/TPC and MTC in aged CVP. (a) Upregulated top-ranked ligands to regulate MTCs by aged BCs according to NicheNet, and dot plot showing the expression percentage (dot size) and intensity (dot intensity) of top-ranked ligands in young and aged BCs (left). Ligand-receptor pairs showing interaction between aged BCs and MTCs ordered

by ligand activity (middle). Heatmap showing regulatory potential of upregulated top ranked ligands in aged BCs and the downstream target genes upregulated in aged MTCs (right). (b-d) Same output as (a), showing communication between MTC and BC (b), TPC and MTC (c), MTC and TPC (d).

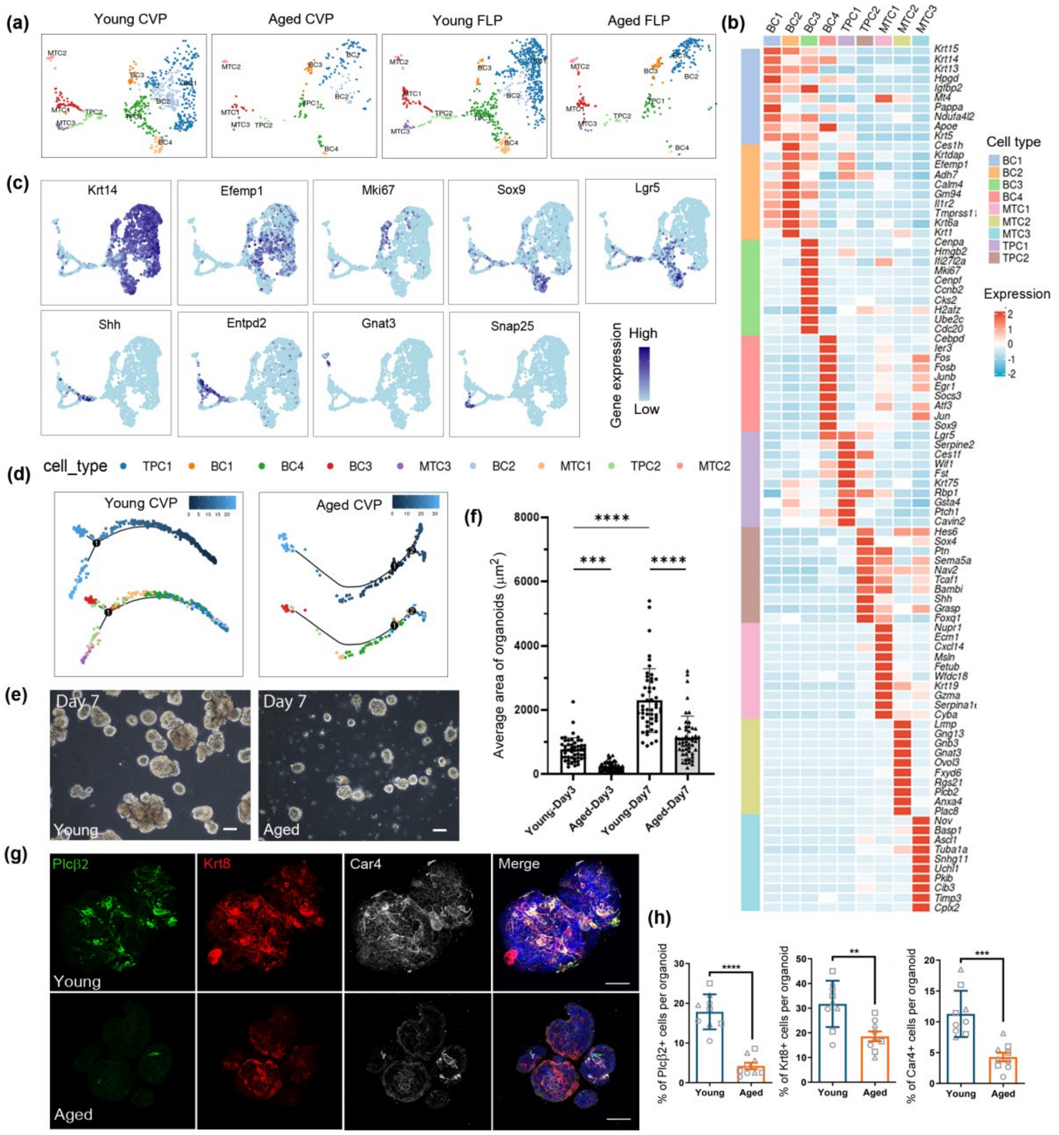

Figure S7. Subclustering of BC, TPC, and MTC in the young and aged CVP. (a) UMAP plots showing subclusters of BC, TPC and MTC in young and aged taste papillae. (b) Enriched highly expressed genes for each subcluster of BC, TPC, and MTC. (c) UMAP plots showing expression of molecular markers for BC, TPC, and MTC in the CVP. (d) Pseudotime trajectory of taste cell maturation from BC and TPC in the young and aged CVP by Monocle2. (e) Images of organoids derived from young and aged CVP at Day 7 post culture. (f) Quantification of organoid size at Day 3 and Day 7 post culture, n = 50 organoids for each group. (g, h) Confocal images (g) and quantification (h) of PLCβ2^{+}, Krt8^{+}, Car4^{+} cells in young and aged CVP organoids. n = 9 preparations for each group. The statistical significance was determined by unpaired t test. ** $p < 0.01$, *** $p < 0.001$, **** $p < 0.0001$. Scale bars, 100 μm in (e), 50 μm in (g).

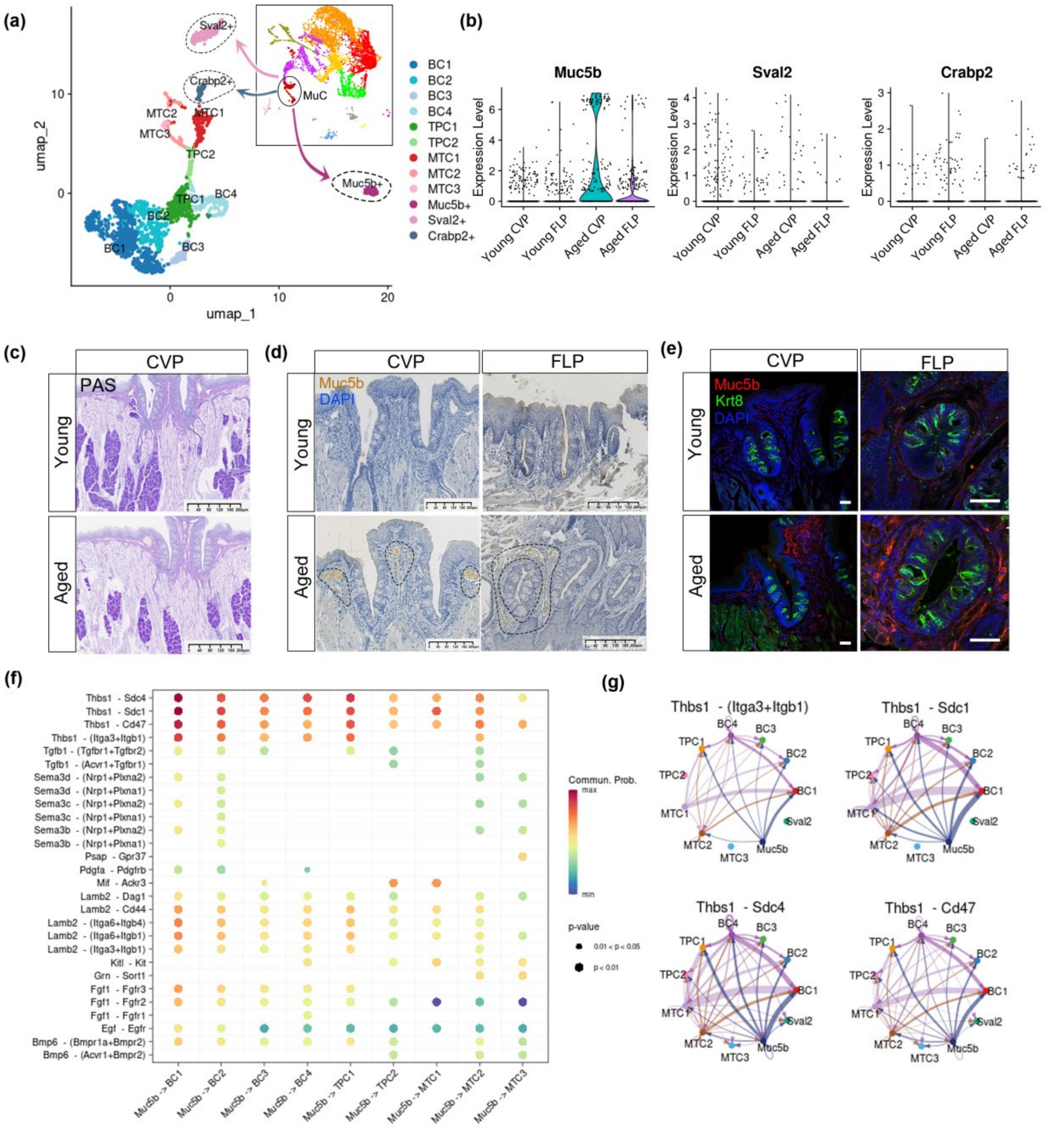

Figure S8. Intracellular communication between Muc5b^{+} cell and basal/progenitor cells. (a) UMAP plot showing subclusters of MuCs. (b) Violin plots showing expression of *Muc5b*, *Sval2*, *Crabp2* in young and aged taste papillae. (c) Images of PAS staining of young and aged CVP. (d, e) Immunostaining images of Muc5b^{+} and Muc5b^{+} Krt8^{+} cells in young and aged taste papillae. (f) Ligand/receptor pairs between Muc5b^{+} cells and BC/TPC/MTC in aged CVP by CellChat. (g) Intercellular communication by Thbs1 among Muc5b^{+} cell, Sval2^{+} cell, BC, TPC and MTC. Scale bars: 200 µm in (c) and (d), 50 µm in (e).

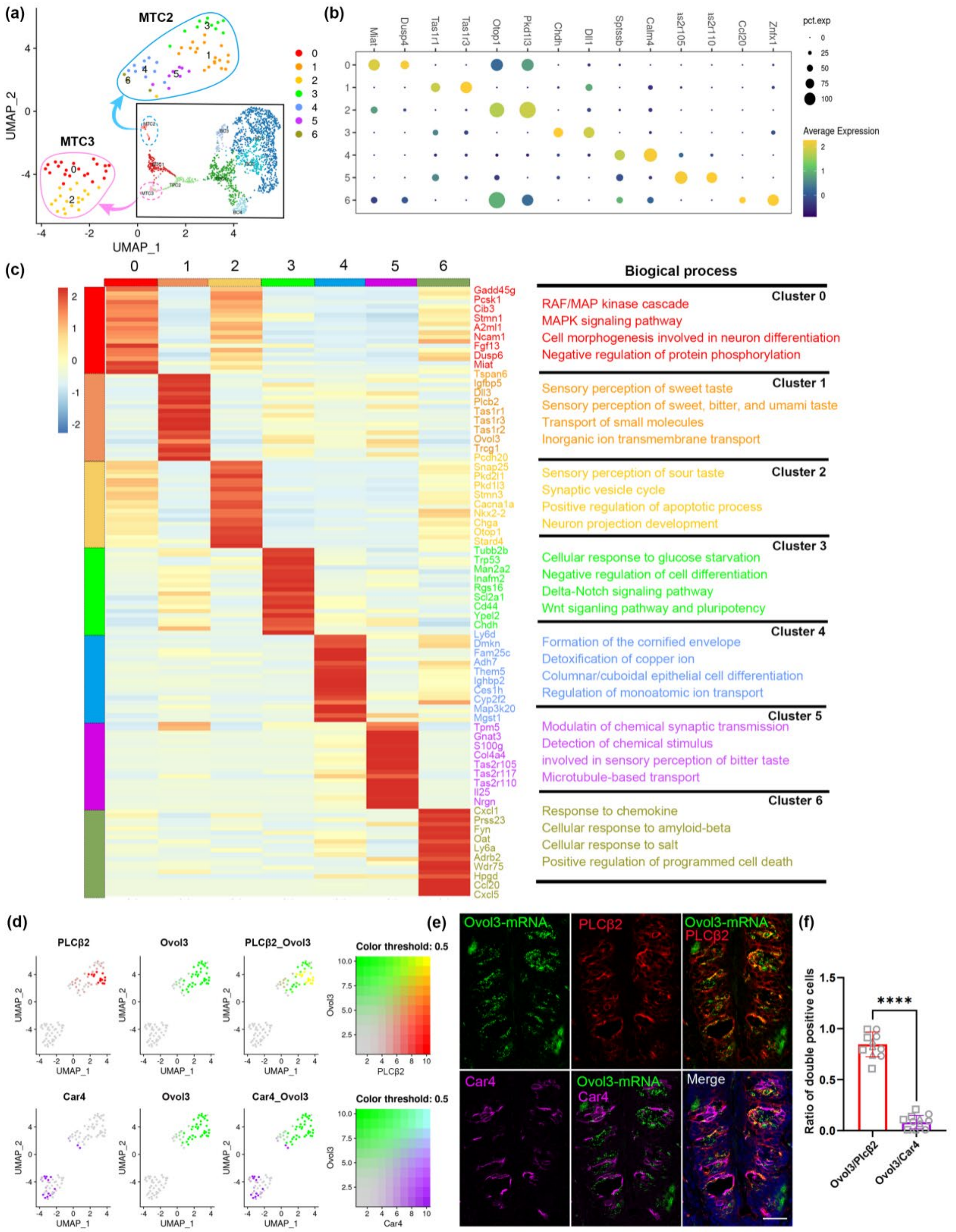

Figure S9. Subclusters of Type II and Type III taste cells in the CVP. (a) UMAP plot showing seven subclusters of Type II and Type III MTCs. (b) Dot plot showing two typical markers for each MTC subtype. (c) Heatmap of top enriched genes for seven subclusters, and biological processes of enriched genes by GO analysis. (d) FeaturePlot blend showing expression of *PLCβ2* and *Ovol3*, *Car4* and *Ovol3* in the CVP. (e) Confocal images of *Ovol3*-mRNA^{+}, PLCβ2^{+}, Car4^{+} cells in the CVP. (f) Quantification of *Ovol3*-mRNA^{+}/PLCβ2^{+} and *Ovol3*-mRNA^{+}/Car4^{+} cells. n = 11 sections for each group. Statistical significance was determined by unpaired t test. Scale bar, 50 μm.
