# Supplementary material for: Single‐cell transcriptomic atlas of taste papilla aging
Source: Aging Cell. 2024 Aug 21;23(12):e14308. doi: 10.1111/acel.14308 (PMC11634696; doi:10.1111/acel.14308)
Supplement: Supplementary file 2 — Table S1. [file ACEL-23-e14308-s004.pdf]

Table S1. Marker genes for each cell type in the CVP.

p_val	avg_log2FC	pct.1	pct.2	p_val_adj	cluster	gene
0	2.5151682	0.953	0.504	0	BC	Krt15
0	2.0616747	0.965	0.509	0	BC	Krt14
0	1.7600217	0.976	0.587	0	BC	Dst
0	1.5458684	0.905	0.368	0	BC	Igfbp2
0	1.5382247	0.998	0.721	0	BC	Krt5
0	1.4938211	0.981	0.51	0	BC	Col17a1
4.68E-142	1.2191714	0.535	0.281	1.12E-137	BC	Hpgd
0	1.1701546	0.927	0.636	0	BC	Wnt4
1.43E-291	1.0753727	0.872	0.468	3.44E-287	BC	Ccnd2
2.91E-279	1.001019	0.798	0.426	6.97E-275	BC	Ccnd1
0	4.4396558	0.909	0.582	0	SEC	Krt4
0	4.1385803	0.857	0.539	0	SEC	Mt4
0	3.6500058	0.987	0.802	0	SEC	Krt13
0	3.3753334	0.998	0.823	0	SEC	Fabp5
0	3.2738213	0.537	0.119	0	SEC	Krt6b
0	3.2538441	0.537	0.156	0	SEC	Sprr1a
0	3.1530885	0.971	0.69	0	SEC	Krtdap
0	3.1297721	0.925	0.309	0	SEC	Fam25c
0	2.8639833	0.956	0.443	0	SEC	Calm4
0	2.8222289	0.895	0.36	0	SEC	Sbsn
0	1.7450416	0.861	0.279	0	TPC	Serpine2
0	1.4971343	0.503	0.074	0	TPC	Wif1
1.49E-231	1.3946407	0.977	0.721	3.58E-227	TPC	Ifitm3
2.63E-223	1.3351004	0.915	0.512	6.32E-219	TPC	Ccnd2
2.19E-39	1.3309082	0.519	0.379	5.25E-35	TPC	Ier3
1.99E-147	1.2894503	0.558	0.221	4.77E-143	TPC	Htra1
2.67E-298	1.2638387	0.628	0.154	6.40E-294	TPC	Tnc
3.23E-28	1.2426799	0.599	0.498	7.74E-24	TPC	Fos
6.42E-157	1.2140411	0.628	0.278	1.54E-152	TPC	Ptch1
7.74E-142	1.1769022	0.879	0.63	1.86E-137	TPC	Id3
0	3.4629628	0.732	0.081	0	CBC	Hist1h2ap
0	2.996454	0.933	0.076	0	CBC	Top2a
0	2.9942803	0.999	0.733	0	CBC	Hmgb2
0	2.9334251	0.799	0.055	0	CBC	Ube2c
0	2.871114	0.676	0.08	0	CBC	Hist1h2ae
0	2.7362144	0.928	0.053	0	CBC	Pclaf
0	2.707384	0.852	0.063	0	CBC	Cenpf
0	2.6586604	0.995	0.438	0	CBC	Stmn1
0	2.480649	0.931	0.083	0	CBC	Mki67
0	2.396846	0.991	0.648	0	CBC	Tubb5
3.09E-35	4.0953199	0.621	0.26	7.41E-31	MC	Dcn
4.87E-13	3.9395698	0.652	0.578	1.17E-08	MC	Gsn
3.73E-88	3.549332	0.591	0.101	8.94E-84	MC	Sparc
0	3.1621316	0.568	0.018	0	MC	Bgn
7.92E-69	2.9454441	0.515	0.093	1.90E-64	MC	Lgals1
5.81E-169	2.7700367	0.568	0.043	1.39E-164	MC	Vim
4.65E-38	2.6430834	0.568	0.19	1.11E-33	MC	Igfbp4
3.85E-91	2.4953666	0.545	0.078	9.22E-87	MC	Timp3
4.68E-35	2.4773959	0.508	0.166	1.12E-30	MC	Plpp3
4.03E-67	2.4179121	0.576	0.123	9.67E-63	MC	Timp2
1.77E-210	5.5008769	0.528	0.115	4.24E-206	MTC	2300002M23Rik
0	3.9128033	0.647	0.08	0	MTC	Cxcl14
0	3.5884278	0.938	0.166	0	MTC	Krt8
0	3.415227	0.707	0.079	0	MTC	Basp1

0	3.1517165	0.93	0.199	0	MTC	Krt18
0	3.1471372	0.69	0.055	0	MTC	Krt19
1.47E-116	2.990121	0.725	0.451	3.53E-112	MTC	Ptn
0	2.9837096	0.823	0.053	0	MTC	Krt7
8.17E-57	2.8241159	0.508	0.286	1.96E-52	MTC	Ecm1
2.18E-106	2.5234195	0.927	0.851	5.22E-102	MTC	S100a6
0	7.6184172	0.956	0.07	0	MuC	Muc5b
1.10E-150	7.0476256	0.952	0.914	2.63E-146	MuC	Lipf
0	6.5621052	0.995	0.921	0	MuC	Sbpl
1.89E-130	6.5492344	0.892	0.843	4.54E-126	MuC	Bpifb1
4.89E-155	6.511587	0.785	0.542	1.17E-150	MuC	Amy1
1.72E-171	6.4381883	0.788	0.496	4.13E-167	MuC	2310057J18Rik
0	4.8444569	0.684	0.031	0	MuC	Agr2
3.43E-195	4.8103955	0.58	0.161	8.22E-191	MuC	Bpifa2
0	4.7493614	0.701	0.167	0	MuC	Prb1
6.67E-205	4.6414456	0.974	0.872	1.60E-200	MuC	Wfdc18
0	7.2882638	0.75	0.049	0	IC	Cd74
0	5.7277757	0.721	0.031	0	IC	H2-Eb1
0	5.6601834	0.725	0.02	0	IC	H2-Aa
2.53E-124	5.5949874	0.733	0.225	6.08E-120	IC	H2-Ab1
0	4.8121711	0.629	0.012	0	IC	C1qa
0	4.7981438	0.662	0.018	0	IC	Cxcl2
2.09E-18	4.550218	0.546	0.45	5.02E-14	IC	Apoe
3.41E-90	4.4760197	0.912	0.774	8.18E-86	IC	Cst3
0	4.3778529	0.758	0.008	0	IC	Tyrobp
0	4.3629254	0.554	0.008	0	IC	C1qb
