# Supplementary material for: Single‐cell transcriptomic atlas of taste papilla aging
Source: Aging Cell. 2024 Aug 21;23(12):e14308. doi: 10.1111/acel.14308 (PMC11634696; doi:10.1111/acel.14308)
Supplement: Supplementary file 3 — Table S2. [file ACEL-23-e14308-s003.pdf]

Table S2. Marker genes for each cell type in the FLP.

p_val	avg_log2FC	pct.1	pct.2	p_val_adj	cluster	gene
0	2.2363041	0.954	0.541	0	BC	Krt15
0	1.8569008	0.996	0.653	0	BC	Krt14
0	1.5766675	0.954	0.584	0	BC	Dst
0	1.4051851	0.998	0.759	0	BC	Krt5
9.07E-199	1.3893514	0.617	0.362	2.17E-194	BC	Hpgd
0	1.3852273	0.974	0.554	0	BC	Col17a1
0	1.3580143	0.938	0.465	0	BC	Igfbp2
0	1.1508308	0.893	0.635	0	BC	Wnt4
0	1.0733092	0.662	0.214	0	BC	Fam213a
0	1.0202763	0.875	0.506	0	BC	Ccnd2
0	3.7212665	0.928	0.596	0	SEC	Krt4
0	3.2256197	0.859	0.594	0	SEC	Mt4
0	3.179733	0.997	0.833	0	SEC	Fabp5
0	3.1199303	0.93	0.597	0	SEC	Krtdap
0	2.9963021	0.986	0.815	0	SEC	Krt13
0	2.6074143	0.901	0.339	0	SEC	Fam25c
0	2.5728118	0.894	0.382	0	SEC	Calm4
0	2.3593117	0.866	0.393	0	SEC	Sbsn
0	2.3083108	0.939	0.766	0	SEC	Krt6a
0	2.2765191	0.871	0.3	0	SEC	Calml3
0	1.7206437	0.801	0.195	0	TPC	Serpine2
9.68E-30	1.6313024	0.589	0.515	2.32E-25	TPC	Fos
5.21E-184	1.5502799	0.628	0.27	1.25E-179	TPC	Htra1
4.06E-252	1.4949886	0.938	0.627	9.73E-248	TPC	Id3
9.52E-243	1.4802754	0.716	0.275	2.28E-238	TPC	Cldn10
1.06E-110	1.4460427	0.503	0.235	2.55E-106	TPC	Socs3
0	1.4407446	0.64	0.107	0	TPC	Tnc
1.88E-65	1.4123177	0.541	0.349	4.51E-61	TPC	Ier3
1.59E-34	1.4011355	0.754	0.717	3.81E-30	TPC	Junb
1.31E-200	1.3864381	0.841	0.528	3.14E-196	TPC	Ces1d
0	3.3159378	0.658	0.087	0	CBC	Hist1h2ap
0	3.2540601	0.859	0.068	0	CBC	Ube2c
0	3.049456	1	0.731	0	CBC	Hmgb2
0	3.0109767	0.933	0.069	0	CBC	Top2a
0	2.8283453	0.562	0.074	0	CBC	Hist1h2ae
0	2.8157371	0.867	0.08	0	CBC	Cenpf
0	2.708209	0.997	0.466	0	CBC	Stmn1
0	2.6061175	0.929	0.051	0	CBC	Birc5
0	2.4442611	0.888	0.045	0	CBC	Pclaf
0	2.4303648	0.905	0.079	0	CBC	Mki67
0	4.1094674	0.505	0.019	0	MC	Col3a1
4.70E-62	4.0975199	0.653	0.248	1.13E-57	MC	Dcn
3.60E-64	4.065631	0.542	0.156	8.64E-60	MC	Igfbp5
8.12E-42	3.8229429	0.774	0.539	1.95E-37	MC	Gsn
0	3.7088034	0.589	0.021	0	MC	Igfbp6
0	3.6912254	0.547	0.009	0	MC	Mfap5
2.15E-114	3.4890058	0.658	0.14	5.15E-110	MC	Sparc
2.18E-262	3.477847	0.663	0.055	5.22E-258	MC	Vim
1.65E-126	3.4220207	0.653	0.123	3.95E-122	MC	Lgals1
0	3.2699201	0.568	0.012	0	MC	Col1a2
8.95E-289	5.5430399	0.611	0.149	2.15E-284	MTC	2300002M23Rik
0	4.2213882	0.665	0.092	0	MTC	Cxcl14
0	3.7237905	0.936	0.144	0	MTC	Krt8
0	3.4892711	0.71	0.087	0	MTC	Basp1

0	3.4211066	0.937	0.185	0	MTC	Krt18
2.11E-212	3.2317563	0.632	0.217	5.05E-208	MTC	Nupr1
0	3.134401	0.737	0.065	0	MTC	Krt19
0	3.0092626	0.841	0.075	0	MTC	Krt7
2.06E-218	2.9875022	0.518	0.135	4.95E-214	MTC	Fetub
1.59E-100	2.9225475	0.593	0.337	3.81E-96	MTC	Ecm1
2.28E-217	7.8773168	0.943	0.738	5.47E-213	MuC	Lipf
6.95E-250	7.4752708	0.971	0.756	1.67E-245	MuC	Sbpl
2.29E-233	6.9930613	0.916	0.53	5.48E-229	MuC	Bpifb1
0	6.5194384	0.855	0.228	0	MuC	2310057J18Rik
1.88E-280	6.4939964	0.838	0.279	4.52E-276	MuC	Amy1
0	6.0665994	0.84	0.099	0	MuC	Muc5b
7.71E-245	5.4214125	0.983	0.748	1.85E-240	MuC	Wfdc18
4.61E-269	4.8178283	0.626	0.117	1.10E-264	MuC	Bpifa2
0	4.2882921	0.851	0.071	0	MuC	Dmbt1
1.17E-185	4.1842272	0.697	0.206	2.80E-181	MuC	Wfdc12
0	7.7663225	0.919	0.059	0	IC	Cd74
0	6.1650334	0.893	0.033	0	IC	H2-Eb1
1.03E-214	6.1545423	0.914	0.179	2.46E-210	IC	H2-Ab1
0	5.9722359	0.893	0.025	0	IC	H2-Aa
0	5.1563454	0.751	0.012	0	IC	C1qa
0	5.0223956	0.695	0.019	0	IC	Cxcl2
0	4.7908801	0.685	0.011	0	IC	C1qb
3.74E-104	4.6027836	0.98	0.828	8.97E-100	IC	Cst3
0	4.563755	0.929	0.013	0	IC	Tyrobp
0	4.3712645	0.98	0.013	0	IC	Cd52
