# Supplementary material for: Single‐cell transcriptomic atlas of taste papilla aging
Source: Aging Cell. 2024 Aug 21;23(12):e14308. doi: 10.1111/acel.14308 (PMC11634696; doi:10.1111/acel.14308)
Supplement: Supplementary file 4 — Table S3. [file ACEL-23-e14308-s002.pdf]

Table S3. DEGs between the aged and young CVP.

p_val	avg_log2FC	pct.1	pct.2	p_val_adj	gene	condition	cluster
4.78E-196	2.5250713	1	0.999	1.15E-191	Gm42418	BC_Agedup	BC
4.47E-93	2.1091968	0.277	0.001	1.07E-88	Xist	BC_Agedup	BC
2.61E-114	1.9844678	0.979	0.854	6.26E-110	Crip1	BC_Agedup	BC
2.42E-175	1.9599177	0.979	0.807	5.81E-171	mt-Atp8	BC_Agedup	BC
2.66E-96	1.8073229	0.616	0.152	6.38E-92	Ifi27l2a	BC_Agedup	BC
4.59E-79	1.558624	0.272	0.01	1.10E-74	Gm10260	BC_Agedup	BC
7.59E-182	1.458393	1	0.999	1.82E-177	mt-Nd4l	BC_Agedup	BC
2.26E-189	1.3678038	1	0.998	5.41E-185	Gm10076	BC_Agedup	BC
1.72E-75	1.2374409	0.767	0.38	4.12E-71	H2-K1	BC_Agedup	BC
2.52E-53	1.1611278	0.593	0.257	6.03E-49	B2m	BC_Agedup	BC
5.66E-48	1.150022	0.34	0.079	1.36E-43	1600014C10Rik	BC_Agedup	BC
6.90E-48	1.1224311	0.791	0.552	1.65E-43	H2-D1	BC_Agedup	BC
4.71E-196	0.9775658	1	1	1.13E-191	Rps28	BC_Agedup	BC
3.65E-61	0.9114925	0.698	0.313	8.75E-57	Gstp1	BC_Agedup	BC
1.35E-32	0.888399	0.86	0.668	3.23E-28	4631405K08Rik	BC_Agedup	BC
1.12E-66	0.8850117	0.658	0.254	2.68E-62	Snhg20	BC_Agedup	BC
5.99E-48	0.8841193	0.933	0.754	1.44E-43	Ifitm3	BC_Agedup	BC
8.59E-57	0.8427812	0.798	0.544	2.06E-52	Erh	BC_Agedup	BC
3.03E-164	0.8360633	1	0.999	7.26E-160	Rpl38	BC_Agedup	BC
4.61E-65	0.8328002	0.909	0.774	1.11E-60	Snrpg	BC_Agedup	BC
1.08E-26	0.83005	0.94	0.849	2.59E-22	Mt2	BC_Agedup	BC
7.38E-67	0.8172153	0.972	0.857	1.77E-62	Atp5k	BC_Agedup	BC
8.14E-59	0.8028341	0.388	0.077	1.95E-54	Gm26870	BC_Agedup	BC
7.34E-34	0.7944372	0.607	0.357	1.76E-29	Car12	BC_Agedup	BC
7.15E-136	0.7839598	1	1	1.72E-131	Rpl35	BC_Agedup	BC
1.32E-95	0.7833872	0.974	0.983	3.16E-91	Uba52	BC_Agedup	BC
5.85E-08	0.7728273	0.426	0.346	0.001403	Apoc1	BC_Agedup	BC
1.18E-25	0.7611629	0.665	0.455	2.84E-21	Tppp3	BC_Agedup	BC
2.37E-177	0.7464409	1	1	5.68E-173	Rps29	BC_Agedup	BC
2.79E-156	0.7344852	1	1	6.68E-152	Rpl37a	BC_Agedup	BC
5.12E-62	0.7304578	0.947	0.903	1.23E-57	Mrpl52	BC_Agedup	BC
3.68E-30	0.7185119	0.681	0.436	8.82E-26	Lars2	BC_Agedup	BC
2.73E-60	0.7137248	0.981	0.949	6.54E-56	Ndufa4	BC_Agedup	BC
1.95E-105	0.7084462	0.312	0.001	4.67E-101	Tff2	BC_Agedup	BC
4.52E-63	0.7016055	0.416	0.086	1.08E-58	Snhg9	BC_Agedup	BC
2.07E-76	0.697407	0.984	0.974	4.96E-72	Rpl27	BC_Agedup	BC
4.53E-44	0.6970396	0.991	0.95	1.09E-39	Lmna	BC_Agedup	BC
4.08E-52	0.6966424	0.909	0.758	9.79E-48	Ndufa1	BC_Agedup	BC
2.34E-30	0.6930866	0.595	0.321	5.61E-26	Ly6e	BC_Agedup	BC
1.91E-28	0.681388	0.793	0.655	4.58E-24	Tmsb10	BC_Agedup	BC
5.06E-40	0.6797851	0.851	0.64	1.21E-35	Ptprs	BC_Agedup	BC
7.21E-41	0.6782729	0.786	0.517	1.73E-36	Ndufa3	BC_Agedup	BC
3.24E-62	0.6753052	0.993	0.97	7.76E-58	Uqcr11	BC_Agedup	BC
1.30E-40	0.651481	0.87	0.668	3.11E-36	Romo1	BC_Agedup	BC
1.41E-14	0.6499803	0.702	0.533	3.37E-10	Wfdc2	BC_Agedup	BC
2.06E-29	0.6345073	0.767	0.528	4.93E-25	Rsrp1	BC_Agedup	BC
1.04E-30	0.6299624	0.937	0.905	2.48E-26	Dbi	BC_Agedup	BC
4.52E-16	0.6271005	0.221	0.083	1.08E-11	H19	BC_Agedup	BC
1.12E-102	0.6083695	1	1	2.68E-98	Rps27	BC_Agedup	BC
1.09E-26	0.6004085	0.805	0.617	2.62E-22	Ktn1	BC_Agedup	BC
3.66E-37	0.5979882	0.909	0.78	8.79E-33	Usmg5	BC_Agedup	BC
1.19E-37	0.5972909	0.87	0.709	2.86E-33	Bola2	BC_Agedup	BC
1.37E-143	0.5855915	1	1	3.28E-139	Rpl41	BC_Agedup	BC
3.40E-13	0.5854728	0.726	0.632	8.16E-09	Krt4	BC_Agedup	BC
8.35E-57	0.5787654	0.974	0.954	2.00E-52	Tomm7	BC_Agedup	BC
7.28E-29	0.5778956	0.795	0.578	1.75E-24	Ewsr1	BC_Agedup	BC
2.07E-116	0.5764341	1	1	4.97E-112	Rpl37	BC_Agedup	BC

7.06E-29	0.5729414	0.898	0.763	1.69E-24	Itgb4	BC_Agedup	BC
1.84E-32	0.5711542	0.923	0.818	4.41E-28	Cox6a1	BC_Agedup	BC
2.04E-22	0.5688939	0.649	0.425	4.88E-18	Gm47283	BC_Agedup	BC
1.47E-22	0.5672941	0.791	0.621	3.54E-18	Sod1	BC_Agedup	BC
4.65E-23	0.5670458	0.879	0.802	1.12E-18	Cstb	BC_Agedup	BC
2.87E-26	0.5664874	0.847	0.721	6.89E-22	Cavin1	BC_Agedup	BC
1.84E-29	0.5646577	0.777	0.549	4.41E-25	Lsm7	BC_Agedup	BC
2.68E-08	0.5582732	0.914	0.902	0.000642	Igfbp2	BC_Agedup	BC
3.29E-19	0.5517371	0.665	0.479	7.90E-15	Igfbp7	BC_Agedup	BC
2.50E-29	0.5484608	0.414	0.166	5.98E-25	Slc7a5	BC_Agedup	BC
1.57E-20	0.5476735	0.712	0.526	3.77E-16	Taf1d	BC_Agedup	BC
3.01E-115	0.5475432	1	1	7.21E-111	Rpl36	BC_Agedup	BC
9.65E-20	0.5295659	0.337	0.153	2.32E-15	Tfcp2l1	BC_Agedup	BC
9.83E-31	0.5294316	0.614	0.322	2.36E-26	Pet100	BC_Agedup	BC
2.78E-25	0.5293574	0.384	0.157	6.67E-21	Calml3	BC_Agedup	BC
2.14E-25	0.526582	0.998	0.997	5.14E-21	S100a6	BC_Agedup	BC
8.16E-44	0.5233834	0.214	0.023	1.96E-39	Muc5b	BC_Agedup	BC
1.37E-25	0.517474	0.814	0.63	3.28E-21	Hnrnpl	BC_Agedup	BC
2.60E-20	0.511467	0.991	0.964	6.23E-16	Mt1	BC_Agedup	BC
4.90E-21	0.5055296	0.767	0.579	1.17E-16	Jag2	BC_Agedup	BC
1.87E-22	0.5045187	0.914	0.829	4.49E-18	Son	BC_Agedup	BC
6.38E-49	0.5035284	0.972	0.977	1.53E-44	Cox6b1	BC_Agedup	BC
7.66E-33	0.4925169	0.937	0.916	1.84E-28	Rpl36al	BC_Agedup	BC
1.81E-22	0.4920027	0.867	0.79	4.35E-18	Ndufb8	BC_Agedup	BC
4.73E-14	0.4909085	0.809	0.682	1.13E-09	Slc6a6	BC_Agedup	BC
4.50E-48	0.487562	0.991	0.996	1.08E-43	Cox6c	BC_Agedup	BC
6.10E-27	0.4816215	0.337	0.127	1.46E-22	Slc16a11	BC_Agedup	BC
8.73E-23	0.4779242	0.786	0.606	2.09E-18	Ddb1	BC_Agedup	BC
8.81E-12	0.4755978	0.521	0.35	2.11E-07	Fam25c	BC_Agedup	BC
9.62E-18	0.4716543	0.593	0.405	2.31E-13	Flna	BC_Agedup	BC
2.67E-27	0.4709051	0.949	0.928	6.41E-23	Uqcrq	BC_Agedup	BC
1.07E-19	0.4707237	0.649	0.454	2.56E-15	Ociad2	BC_Agedup	BC
3.18E-22	0.4620871	0.842	0.714	7.63E-18	Ndufb2	BC_Agedup	BC
7.60E-38	0.4608781	0.979	0.985	1.82E-33	Cox7c	BC_Agedup	BC
1.62E-23	0.4596879	0.86	0.744	3.89E-19	Ost4	BC_Agedup	BC
1.17E-86	0.4577162	1	1	2.81E-82	Rps19	BC_Agedup	BC
1.11E-18	0.4525549	0.786	0.643	2.65E-14	Eif4g1	BC_Agedup	BC
1.57E-16	0.4519863	0.677	0.514	3.76E-12	Dpm3	BC_Agedup	BC
1.70E-17	0.4512105	0.505	0.301	4.07E-13	Itga3	BC_Agedup	BC
9.64E-19	0.4506425	0.779	0.64	2.31E-14	Naa38	BC_Agedup	BC
6.15E-33	0.4494284	0.993	0.994	1.47E-28	mt-Nd5	BC_Agedup	BC
1.31E-21	0.4455957	0.886	0.805	3.13E-17	Ndufa11	BC_Agedup	BC
1.08E-21	0.4454081	0.481	0.256	2.60E-17	Grcc10	BC_Agedup	BC
8.92E-16	0.4440365	0.556	0.377	2.14E-11	Fat2	BC_Agedup	BC
4.08E-19	0.4433864	0.665	0.462	9.79E-15	Gas6	BC_Agedup	BC
4.34E-25	0.4405574	0.933	0.92	1.04E-20	Tspo	BC_Agedup	BC
1.43E-21	0.4383239	0.605	0.357	3.44E-17	Csrp1	BC_Agedup	BC
1.43E-16	0.4371519	0.505	0.313	3.43E-12	Gpt	BC_Agedup	BC
1.24E-17	0.4350616	0.84	0.766	2.98E-13	Ndufc2	BC_Agedup	BC
6.17E-22	0.434241	0.919	0.896	1.48E-17	2010107E04Rik	BC_Agedup	BC
1.33E-20	0.4304532	0.826	0.704	3.19E-16	Ndufa5	BC_Agedup	BC
1.28E-13	0.4292849	0.753	0.625	3.08E-09	Cbx3	BC_Agedup	BC
1.67E-17	0.4255221	0.914	0.88	4.01E-13	Crip2	BC_Agedup	BC
2.17E-27	0.4251009	0.951	0.94	5.19E-23	Uqcr10	BC_Agedup	BC
7.57E-10	0.422168	0.716	0.619	1.82E-05	Notch1	BC_Agedup	BC
3.07E-14	0.4202916	0.772	0.627	7.37E-10	Ly6a	BC_Agedup	BC
6.53E-19	0.4193685	0.83	0.681	1.57E-14	Psap	BC_Agedup	BC
9.41E-18	0.4182733	0.821	0.701	2.26E-13	Mrpl33	BC_Agedup	BC
5.10E-21	0.4177474	0.849	0.736	1.22E-16	Arpp19	BC_Agedup	BC

9.33E-75	0.4169913	0.998	1	2.24E-70	Rpl39	BC_Agedup	BC
9.21E-25	0.4154694	0.912	0.882	2.21E-20	Ndufa2	BC_Agedup	BC
2.69E-07	0.4152757	0.463	0.336	0.006445	Pappa	BC_Agedup	BC
2.07E-18	0.4152039	0.795	0.627	4.95E-14	Mrps21	BC_Agedup	BC
3.04E-70	0.414646	1	1	7.29E-66	Rps20	BC_Agedup	BC
1.19E-10	0.4119634	0.856	0.763	2.86E-06	Sdc1	BC_Agedup	BC
5.65E-21	0.4109587	0.933	0.937	1.35E-16	Sec61g	BC_Agedup	BC
1.70E-16	0.4106601	0.586	0.402	4.08E-12	Dnajc19	BC_Agedup	BC
9.35E-17	0.4043208	0.86	0.728	2.24E-12	Ctsd	BC_Agedup	BC
2.35E-15	0.4021221	0.505	0.311	5.64E-11	Sfrp1	BC_Agedup	BC
1.39E-26	0.3983039	0.979	0.986	3.34E-22	Cox5b	BC_Agedup	BC
3.70E-09	0.3981266	0.798	0.749	8.86E-05	Cst3	BC_Agedup	BC
2.15E-63	0.3968731	1	1	5.16E-59	Rps2	BC_Agedup	BC
4.68E-13	0.3967816	0.616	0.457	1.12E-08	Plxnb1	BC_Agedup	BC
2.48E-11	0.3965652	0.34	0.194	5.96E-07	Igfbp4	BC_Agedup	BC
5.04E-12	0.3958634	0.763	0.643	1.21E-07	Tardbp	BC_Agedup	BC
1.85E-12	0.3954466	0.707	0.592	4.43E-08	Eno1	BC_Agedup	BC
5.74E-16	0.3944615	0.581	0.381	1.38E-11	Hk1	BC_Agedup	BC
5.60E-11	0.3928931	0.656	0.512	1.34E-06	Ptgfrn	BC_Agedup	BC
1.19E-13	0.3909709	0.619	0.462	2.85E-09	Gm11808	BC_Agedup	BC
1.87E-19	0.390814	0.984	0.97	4.49E-15	Ncl	BC_Agedup	BC
3.53E-15	0.3865983	0.165	0.05	8.47E-11	Cenpa	BC_Agedup	BC
4.81E-14	0.3864593	0.488	0.311	1.15E-09	Cluh	BC_Agedup	BC
5.44E-28	0.3857691	0.986	0.997	1.31E-23	Atp5e	BC_Agedup	BC
1.11E-24	0.3826636	0.984	0.978	2.66E-20	Pabpc1	BC_Agedup	BC
4.65E-11	0.3815924	0.609	0.477	1.12E-06	Psme2	BC_Agedup	BC
3.03E-26	0.3787841	0.309	0.104	7.26E-22	Gm10036	BC_Agedup	BC
5.92E-11	0.3786461	0.644	0.507	1.42E-06	Prrc2a	BC_Agedup	BC
8.77E-15	0.3761261	0.549	0.364	2.10E-10	Prpf8	BC_Agedup	BC
6.02E-16	0.3755959	0.835	0.71	1.44E-11	Srsf5	BC_Agedup	BC
9.45E-12	0.3737541	0.56	0.395	2.27E-07	Mink1	BC_Agedup	BC
9.26E-14	0.3719105	0.714	0.576	2.22E-09	Ndufv3	BC_Agedup	BC
2.65E-19	0.3691089	0.465	0.247	6.36E-15	Cox17	BC_Agedup	BC
8.16E-12	0.367593	0.653	0.474	1.96E-07	Ccnl2	BC_Agedup	BC
3.06E-22	0.367534	0.944	0.899	7.35E-18	Atp5g3	BC_Agedup	BC
2.14E-14	0.367409	0.867	0.765	5.14E-10	Nop53	BC_Agedup	BC
2.33E-19	0.3669497	0.944	0.951	5.59E-15	Elob	BC_Agedup	BC
2.28E-13	0.3669484	0.714	0.549	5.47E-09	Rcc2	BC_Agedup	BC
8.00E-13	0.366343	0.833	0.753	1.92E-08	Prelid1	BC_Agedup	BC
2.48E-12	0.3626187	0.7	0.566	5.95E-08	Tmem256	BC_Agedup	BC
6.09E-18	0.3623746	0.36	0.167	1.46E-13	Gm8186	BC_Agedup	BC
1.63E-13	0.3615104	0.36	0.198	3.90E-09	Srebf2	BC_Agedup	BC
8.15E-29	0.3610752	0.991	0.98	1.95E-24	Sem1	BC_Agedup	BC
1.08E-16	0.3550637	0.96	0.903	2.60E-12	H2afj	BC_Agedup	BC
7.29E-12	0.3545527	0.605	0.455	1.75E-07	1810022K09Rik	BC_Agedup	BC
2.09E-12	0.3544955	0.714	0.588	5.01E-08	Chchd1	BC_Agedup	BC
3.40E-14	0.3533835	0.502	0.315	8.15E-10	Tnfrsf1a	BC_Agedup	BC
5.70E-11	0.3530412	0.612	0.446	1.37E-06	Moxd1	BC_Agedup	BC
2.92E-13	0.3516807	0.812	0.719	7.01E-09	Spint2	BC_Agedup	BC
2.73E-07	0.351615	0.644	0.496	0.006551	Gm26917	BC_Agedup	BC
8.73E-09	0.3515451	0.602	0.452	0.000209	H1f0	BC_Agedup	BC
6.29E-13	0.3504506	0.872	0.838	1.51E-08	Ndufc1	BC_Agedup	BC
1.29E-10	0.3493433	0.73	0.6	3.08E-06	Cops9	BC_Agedup	BC
2.72E-12	0.3482713	0.923	0.866	6.53E-08	Pkp1	BC_Agedup	BC
3.87E-08	0.3469152	0.507	0.384	0.000927	Hopx	BC_Agedup	BC
1.75E-12	0.3468002	0.474	0.304	4.19E-08	Cep170b	BC_Agedup	BC
1.17E-13	0.3434394	0.823	0.742	2.80E-09	Trmt112	BC_Agedup	BC
1.78E-08	0.3426436	0.402	0.276	0.000428	Vsig8	BC_Agedup	BC
1.18E-11	0.3419438	0.442	0.283	2.84E-07	Nfx1	BC_Agedup	BC

2.52E-11	0.3412293	0.47	0.311	6.05E-07	Asph	BC_Agedup	BC
5.70E-15	0.3401416	0.947	0.963	1.37E-10	Cox5a	BC_Agedup	BC
1.78E-10	0.3398055	0.621	0.486	4.27E-06	Ier3ip1	BC_Agedup	BC
1.97E-09	0.3378354	0.498	0.354	4.72E-05	Ece1	BC_Agedup	BC
1.82E-28	0.3366749	0.984	0.992	4.36E-24	Atp5l	BC_Agedup	BC
1.12E-09	0.3366099	0.502	0.35	2.69E-05	9-Sep	BC_Agedup	BC
1.28E-09	0.336462	0.647	0.526	3.07E-05	Csnk2b	BC_Agedup	BC
4.93E-08	0.3348042	0.667	0.579	0.001182	Hnrnpdl	BC_Agedup	BC
6.69E-11	0.3343111	0.463	0.305	1.60E-06	Coro1c	BC_Agedup	BC
4.81E-09	0.3340035	0.34	0.211	0.000115	Gdpd2	BC_Agedup	BC
1.07E-17	0.3336235	0.977	0.976	2.56E-13	Ndufb9	BC_Agedup	BC
1.31E-11	0.3334777	0.46	0.286	3.15E-07	Lama5	BC_Agedup	BC
2.75E-10	0.3330453	0.784	0.717	6.61E-06	Ndufb7	BC_Agedup	BC
3.54E-21	0.3329865	0.34	0.136	8.49E-17	Sparc	BC_Agedup	BC
1.20E-20	0.3316036	0.956	0.951	2.87E-16	Snrpe	BC_Agedup	BC
6.45E-15	0.3298529	0.947	0.952	1.55E-10	Atp5j	BC_Agedup	BC
2.66E-16	0.328312	0.363	0.184	6.38E-12	Polr2l	BC_Agedup	BC
1.36E-12	0.3282906	0.963	0.952	3.27E-08	Atp1a1	BC_Agedup	BC
1.12E-11	0.3272681	0.579	0.428	2.69E-07	Mrps28	BC_Agedup	BC
1.39E-13	0.3266706	0.933	0.879	3.33E-09	Ndufa13	BC_Agedup	BC
1.31E-11	0.3257277	0.558	0.399	3.14E-07	Setd5	BC_Agedup	BC
1.48E-09	0.3252624	0.423	0.283	3.56E-05	Lasp1	BC_Agedup	BC
3.72E-13	0.3243866	0.363	0.196	8.93E-09	Furin	BC_Agedup	BC
2.50E-09	0.3243071	0.614	0.485	5.99E-05	Rpn1	BC_Agedup	BC
2.22E-09	0.3218927	0.66	0.538	5.33E-05	Eif3b	BC_Agedup	BC
2.81E-29	0.3205152	0.247	0.059	6.75E-25	Adat2	BC_Agedup	BC
2.67E-10	0.320312	0.642	0.496	6.40E-06	Larp1	BC_Agedup	BC
1.16E-19	0.3197401	0.272	0.1	2.79E-15	Nme2	BC_Agedup	BC
3.87E-10	0.3195989	0.684	0.536	9.27E-06	Ifi27	BC_Agedup	BC
1.99E-13	0.3195295	0.316	0.161	4.76E-09	Lamb2	BC_Agedup	BC
9.56E-11	0.3181127	0.379	0.236	2.29E-06	Vars	BC_Agedup	BC
6.37E-11	0.3164111	0.702	0.545	1.53E-06	Ltbp4	BC_Agedup	BC
2.44E-14	0.3163721	0.323	0.164	5.86E-10	Dvl1	BC_Agedup	BC
1.15E-11	0.3157729	0.391	0.227	2.76E-07	Shmt2	BC_Agedup	BC
1.80E-07	0.315129	0.516	0.39	0.004308	Lmo1	BC_Agedup	BC
7.66E-08	0.3149343	0.288	0.175	0.001836	Smoc2	BC_Agedup	BC
3.00E-12	0.3139882	0.902	0.882	7.20E-08	Sec61b	BC_Agedup	BC
5.37E-07	0.3127795	0.698	0.609	0.01288	Ddx3x	BC_Agedup	BC
2.44E-09	0.3119493	0.805	0.686	5.86E-05	Actn4	BC_Agedup	BC
2.68E-09	0.3116069	0.465	0.33	6.42E-05	Atxn2l	BC_Agedup	BC
5.54E-17	0.310581	0.963	0.982	1.33E-12	Atp5j2	BC_Agedup	BC
1.08E-07	0.3100979	0.647	0.558	0.002599	Selenoh	BC_Agedup	BC
4.36E-09	0.3100932	0.558	0.412	0.000105	Clstn1	BC_Agedup	BC
2.12E-10	0.3100507	0.43	0.279	5.09E-06	Susd6	BC_Agedup	BC
5.51E-08	0.3098244	0.36	0.238	0.001321	Kctd15	BC_Agedup	BC
2.95E-10	0.3097547	0.747	0.669	7.07E-06	Nenf	BC_Agedup	BC
6.18E-09	0.3097177	0.477	0.329	0.000148	Sema3f	BC_Agedup	BC
4.94E-44	0.3093624	1	1	1.18E-39	Rpl35a	BC_Agedup	BC
2.58E-14	0.3089506	0.388	0.209	6.18E-10	Rpl27-ps3	BC_Agedup	BC
5.46E-10	0.3081925	0.402	0.256	1.31E-05	Hs6st1	BC_Agedup	BC
8.16E-13	0.3078846	0.902	0.845	1.96E-08	Ndufs6	BC_Agedup	BC
5.49E-08	0.3071261	0.688	0.582	0.001317	Nop58	BC_Agedup	BC
5.11E-21	0.3067116	0.993	0.996	1.22E-16	Atp5g2	BC_Agedup	BC
4.50E-51	0.3062306	1	1	1.08E-46	Rpl30	BC_Agedup	BC
1.00E-08	0.3052055	0.367	0.241	0.00024	Lars	BC_Agedup	BC
1.22E-07	0.3038047	0.795	0.732	0.002925	Snrnp70	BC_Agedup	BC
8.32E-07	0.3021507	0.7	0.6	0.019955	Tmem132a	BC_Agedup	BC
1.20E-11	0.3007849	0.472	0.294	2.88E-07	Tmem259	BC_Agedup	BC
6.22E-12	0.300683	0.391	0.23	1.49E-07	Zfp219	BC_Agedup	BC

5.48E-16	0.2989298	0.96	0.975	1.32E-11	Cox7a2	BC_Agedup	BC
1.02E-15	0.2979607	0.96	0.961	2.44E-11	Rps27l	BC_Agedup	BC
3.18E-09	0.2977914	0.488	0.343	7.64E-05	Ccdc3	BC_Agedup	BC
3.39E-15	0.2977891	0.288	0.127	8.12E-11	Apobec1	BC_Agedup	BC
9.14E-28	0.2967645	0.991	1	2.19E-23	Rpl36a	BC_Agedup	BC
2.19E-10	0.2965606	0.423	0.266	5.26E-06	Taf6l	BC_Agedup	BC
6.02E-07	0.2956168	0.293	0.191	0.014448	Il6st	BC_Agedup	BC
1.88E-13	0.2955251	0.384	0.21	4.51E-09	Dynlt1f	BC_Agedup	BC
8.25E-10	0.2949855	0.407	0.265	1.98E-05	Stk11	BC_Agedup	BC
2.57E-09	0.2946028	0.435	0.283	6.17E-05	Bag6	BC_Agedup	BC
1.98E-17	0.2944904	0.184	0.056	4.76E-13	Adam8	BC_Agedup	BC
3.79E-08	0.294347	0.46	0.331	0.000908	Hr	BC_Agedup	BC
4.02E-16	0.292906	0.986	0.994	9.65E-12	Oaz1	BC_Agedup	BC
7.27E-08	0.291168	0.435	0.304	0.001744	Ldb1	BC_Agedup	BC
7.01E-07	0.2907434	0.665	0.577	0.016801	Lad1	BC_Agedup	BC
1.60E-09	0.2906407	0.421	0.279	3.85E-05	Ndufs1	BC_Agedup	BC
2.05E-09	0.2905683	0.484	0.336	4.92E-05	Hypk	BC_Agedup	BC
1.46E-06	0.2896759	0.572	0.466	0.035083	Tmem176b	BC_Agedup	BC
5.11E-13	0.2889198	0.414	0.238	1.23E-08	Atad3a	BC_Agedup	BC
5.90E-08	0.2882174	0.577	0.442	0.001415	Pin4	BC_Agedup	BC
2.30E-10	0.2875285	0.342	0.198	5.50E-06	Smim4	BC_Agedup	BC
8.60E-07	0.2874955	0.477	0.366	0.020632	Wdr1	BC_Agedup	BC
2.19E-11	0.2865944	0.491	0.318	5.25E-07	Fmc1	BC_Agedup	BC
1.55E-06	0.2861116	0.412	0.305	0.037093	Sidt2	BC_Agedup	BC
1.47E-08	0.2853861	0.519	0.367	0.000353	Ptk7	BC_Agedup	BC
1.40E-07	0.2853568	0.791	0.727	0.003346	Itgb1	BC_Agedup	BC
1.98E-08	0.2853496	0.57	0.432	0.000475	Sap18	BC_Agedup	BC
1.65E-12	0.2845484	0.253	0.118	3.96E-08	0610010K14Rik	BC_Agedup	BC
2.79E-07	0.2843519	0.888	0.816	0.006687	Dsc3	BC_Agedup	BC
2.58E-07	0.2842368	0.581	0.457	0.006179	Kank1	BC_Agedup	BC
1.46E-06	0.2840937	0.174	0.093	0.035046	Cenpf	BC_Agedup	BC
1.62E-10	0.2835867	0.263	0.137	3.89E-06	Bcap29	BC_Agedup	BC
1.89E-08	0.280764	0.749	0.66	0.000454	Cox14	BC_Agedup	BC
1.80E-07	0.2795938	0.705	0.614	0.004323	Gtf2i	BC_Agedup	BC
1.77E-08	0.2782466	0.721	0.641	0.000424	Cycs	BC_Agedup	BC
2.64E-11	0.2781419	0.233	0.109	6.33E-07	Idi1	BC_Agedup	BC
2.11E-07	0.2775836	0.579	0.463	0.00505	Cdh1	BC_Agedup	BC
6.54E-45	0.2773821	0.998	1	1.57E-40	Rpl11	BC_Agedup	BC
1.83E-10	0.2771399	0.307	0.17	4.39E-06	Fam193b	BC_Agedup	BC
3.64E-08	0.2768397	0.4	0.26	0.000873	2310039H08Rik	BC_Agedup	BC
6.32E-09	0.2764875	0.477	0.335	0.000152	Cmc1	BC_Agedup	BC
5.54E-08	0.2748236	0.347	0.227	0.00133	Slc39a1	BC_Agedup	BC
5.54E-11	0.2739918	0.381	0.222	1.33E-06	Slc35a4	BC_Agedup	BC
1.37E-09	0.2739724	0.863	0.796	3.29E-05	Ndufa6	BC_Agedup	BC
4.38E-07	0.2733028	0.451	0.331	0.010512	AC149090.1	BC_Agedup	BC
7.85E-17	0.2723377	0.186	0.058	1.88E-12	Cyp7b1	BC_Agedup	BC
4.65E-07	0.2721404	0.8	0.762	0.011156	D8Ertd738e	BC_Agedup	BC
1.86E-11	0.2718431	0.272	0.134	4.46E-07	Slc1a3	BC_Agedup	BC
1.06E-06	0.271657	0.644	0.532	0.025452	Hnrnpd	BC_Agedup	BC
3.19E-07	0.2714573	0.463	0.336	0.007654	Tnpo2	BC_Agedup	BC
4.05E-10	0.2711719	0.407	0.252	9.72E-06	Ncaph2	BC_Agedup	BC
1.08E-08	0.2709615	0.284	0.167	0.000258	Afg3l1	BC_Agedup	BC
5.02E-11	0.2706471	0.351	0.199	1.20E-06	Zfc3h1	BC_Agedup	BC
1.07E-10	0.2698949	0.921	0.877	2.56E-06	Ndufa7	BC_Agedup	BC
6.65E-11	0.2693875	0.326	0.183	1.59E-06	Ttc37	BC_Agedup	BC
2.40E-07	0.2677691	0.888	0.889	0.005746	Reep5	BC_Agedup	BC
4.64E-08	0.267434	0.367	0.237	0.001113	Mapkapk2	BC_Agedup	BC
5.21E-07	0.2670446	0.84	0.79	0.012486	Lsm4	BC_Agedup	BC
3.95E-09	0.2665274	0.847	0.806	9.48E-05	Tma7	BC_Agedup	BC

1.06E-06	0.2658183	0.472	0.344	0.025418	Itm2c	BC_Agedup	BC
6.04E-10	0.2657218	0.263	0.139	1.45E-05	Lrrc45	BC_Agedup	BC
3.22E-07	0.2654072	0.433	0.309	0.007714	Mgea5	BC_Agedup	BC
6.38E-11	0.264893	0.247	0.122	1.53E-06	Slc6a9	BC_Agedup	BC
8.35E-14	0.264833	0.284	0.13	2.00E-09	Rgl2	BC_Agedup	BC
3.65E-07	0.2642121	0.453	0.331	0.008743	Sytl1	BC_Agedup	BC
5.93E-09	0.2636826	0.858	0.81	0.000142	Nop10	BC_Agedup	BC
2.91E-10	0.2633742	0.9	0.835	6.97E-06	Uqcc2	BC_Agedup	BC
3.50E-08	0.2629799	0.351	0.22	0.000838	Gtf3c2	BC_Agedup	BC
3.11E-07	0.2626126	0.721	0.597	0.007456	Chd4	BC_Agedup	BC
1.88E-11	0.2624487	0.249	0.119	4.50E-07	Cc2d1b	BC_Agedup	BC
1.11E-07	0.2624444	0.588	0.481	0.002661	Mybbp1a	BC_Agedup	BC
1.31E-12	0.2613156	0.188	0.072	3.14E-08	Cyp51	BC_Agedup	BC
2.78E-10	0.261129	0.256	0.132	6.67E-06	Fads1	BC_Agedup	BC
6.41E-07	0.2598095	0.749	0.652	0.015382	Prpf4b	BC_Agedup	BC
4.30E-11	0.2587519	0.314	0.171	1.03E-06	Rassf7	BC_Agedup	BC
3.40E-08	0.2572159	0.528	0.386	0.000815	Nars	BC_Agedup	BC
7.37E-08	0.2571189	0.377	0.25	0.001768	Ilf3	BC_Agedup	BC
2.50E-07	0.2569833	0.428	0.313	0.005985	Sec61a1	BC_Agedup	BC
7.42E-11	0.2566521	0.286	0.151	1.78E-06	Mllt6	BC_Agedup	BC
4.08E-07	0.2552006	0.653	0.556	0.009777	Churc1	BC_Agedup	BC
1.07E-06	0.2539959	0.74	0.688	0.025722	Tmem258	BC_Agedup	BC
5.85E-08	0.2536808	0.807	0.756	0.001403	Ndufb4	BC_Agedup	BC
1.35E-07	0.2514352	0.577	0.434	0.00323	Cdc42se1	BC_Agedup	BC
1.83E-06	0.2509556	0.7	0.593	0.043809	Mast4	BC_Agedup	BC
3.20E-11	0.2507784	0.914	0.904	7.67E-07	Timm13	BC_Agedup	BC
4.54E-08	0.2475794	0.274	0.16	0.001088	Crlf1	BC_Agedup	BC
1.50E-10	0.2464538	0.363	0.211	3.61E-06	Scap	BC_Agedup	BC
4.80E-08	0.2464128	0.879	0.866	0.00115	Nme1	BC_Agedup	BC
4.61E-08	0.2460442	0.923	0.912	0.001105	Minos1	BC_Agedup	BC
4.01E-07	0.2455018	0.684	0.56	0.009612	Ptbp1	BC_Agedup	BC
6.42E-07	0.2454485	0.33	0.216	0.015386	Gigyf1	BC_Agedup	BC
1.71E-07	0.2443844	0.688	0.598	0.004091	Ndufb6	BC_Agedup	BC
7.83E-07	0.2442413	0.335	0.225	0.018772	Arhgef1	BC_Agedup	BC
2.42E-07	0.244096	0.388	0.265	0.005796	Plxna1	BC_Agedup	BC
5.34E-07	0.2433255	0.777	0.7	0.012812	Cyc1	BC_Agedup	BC
1.19E-39	0.2429346	1	1	2.86E-35	Rpl18a	BC_Agedup	BC
1.39E-06	0.2424511	0.779	0.71	0.033214	Iqgap1	BC_Agedup	BC
3.20E-08	0.2424146	0.337	0.212	0.000766	Celf4	BC_Agedup	BC
4.96E-08	0.2423341	0.23	0.127	0.001188	Ptpre	BC_Agedup	BC
4.35E-10	0.2413384	0.956	0.99	1.04E-05	Selenow	BC_Agedup	BC
4.30E-08	0.2396357	0.344	0.211	0.001031	Iars2	BC_Agedup	BC
7.41E-07	0.2390657	0.377	0.262	0.017768	Raf1	BC_Agedup	BC
1.65E-06	0.238807	0.481	0.353	0.039631	Tomm70a	BC_Agedup	BC
9.44E-07	0.2377593	0.34	0.227	0.022627	Ggh	BC_Agedup	BC
1.52E-07	0.237121	0.314	0.198	0.00364	Actr1b	BC_Agedup	BC
5.45E-07	0.2363337	0.344	0.229	0.013074	Psme3	BC_Agedup	BC
1.56E-07	0.2341137	0.202	0.107	0.003737	Trpm4	BC_Agedup	BC
5.59E-07	0.2337848	0.286	0.18	0.013407	Lonp1	BC_Agedup	BC
7.43E-08	0.2337638	0.258	0.149	0.001783	Trpv4	BC_Agedup	BC
4.70E-08	0.2320884	0.179	0.089	0.001127	Tap1	BC_Agedup	BC
8.92E-08	0.2317543	0.305	0.188	0.002139	Nomo1	BC_Agedup	BC
4.48E-08	0.2313164	0.284	0.167	0.001074	Zfp395	BC_Agedup	BC
8.18E-08	0.2302725	0.319	0.195	0.001961	Serhl	BC_Agedup	BC
5.51E-17	0.229927	0.191	0.059	1.32E-12	Akr1b8	BC_Agedup	BC
2.07E-06	0.2289634	0.556	0.437	0.049643	1500011K16Rik	BC_Agedup	BC
2.32E-08	0.22895	0.886	0.844	0.000557	Ndufab1	BC_Agedup	BC
5.72E-07	0.2287089	0.774	0.698	0.013723	Ndufb3	BC_Agedup	BC
1.51E-12	0.2281848	0.972	0.994	3.62E-08	Hint1	BC_Agedup	BC

4.18E-13	0.2281298	0.184	0.068	1.00E-08	Cpxm2	BC_Agedup	BC
2.08E-07	0.2280452	0.23	0.134	0.004997	Itpr2	BC_Agedup	BC
3.08E-07	0.2271637	0.326	0.21	0.007378	Kansl2	BC_Agedup	BC
5.39E-09	0.2259291	0.256	0.139	0.000129	Nacc1	BC_Agedup	BC
1.73E-07	0.2258649	0.374	0.249	0.004154	Ehmt2	BC_Agedup	BC
5.71E-07	0.2244252	0.235	0.137	0.013687	Ube3b	BC_Agedup	BC
4.54E-08	0.2238559	0.319	0.194	0.00109	Ick	BC_Agedup	BC
6.82E-08	0.2235974	0.265	0.153	0.001636	Spg7	BC_Agedup	BC
1.42E-25	0.2223273	0.142	0.019	3.41E-21	Psmb9	BC_Agedup	BC
3.22E-08	0.2212029	0.277	0.16	0.000773	Anpep	BC_Agedup	BC
3.37E-10	0.2212008	0.26	0.132	8.08E-06	Tomm6	BC_Agedup	BC
1.02E-06	0.2208467	0.37	0.256	0.02458	Btbd3	BC_Agedup	BC
2.13E-08	0.2193965	0.209	0.107	0.000511	Oplah	BC_Agedup	BC
1.38E-06	0.2192657	0.519	0.377	0.032985	Hnrnpul1	BC_Agedup	BC
2.05E-15	0.2191029	0.123	0.029	4.91E-11	Col23a1	BC_Agedup	BC
5.78E-11	0.2188657	0.195	0.084	1.39E-06	Myef2	BC_Agedup	BC
3.35E-08	0.2181878	0.172	0.082	0.000804	Ckap2l	BC_Agedup	BC
1.89E-18	0.2176967	0.172	0.045	4.52E-14	Arntl	BC_Agedup	BC
2.11E-10	0.2172937	0.986	0.994	5.05E-06	Atp5h	BC_Agedup	BC
3.74E-08	0.2171246	0.351	0.222	0.000896	Strn4	BC_Agedup	BC
1.25E-11	0.2168203	0.179	0.07	2.99E-07	Als2cl	BC_Agedup	BC
1.39E-10	0.216691	0.235	0.111	3.34E-06	Ccdc85b	BC_Agedup	BC
1.04E-07	0.2157984	0.298	0.179	0.00249	Plcb3	BC_Agedup	BC
4.99E-09	0.2146539	0.2	0.098	0.00012	Bloc1s1	BC_Agedup	BC
6.52E-08	0.2141309	0.935	0.923	0.001563	Ndufb11	BC_Agedup	BC
4.96E-07	0.2132513	0.347	0.23	0.011883	Paxbp1	BC_Agedup	BC
7.52E-07	0.2132215	0.374	0.251	0.018044	2310009B15Rik	BC_Agedup	BC
6.78E-07	0.2131413	0.793	0.766	0.016247	Ndufs7	BC_Agedup	BC
2.41E-07	0.212944	0.237	0.136	0.005768	Slc1a5	BC_Agedup	BC
9.00E-08	0.2115467	0.319	0.196	0.002158	Scaf1	BC_Agedup	BC
2.59E-09	0.2092488	0.274	0.15	6.20E-05	Man2a2	BC_Agedup	BC
7.60E-08	0.2090017	0.288	0.17	0.001824	Copg1	BC_Agedup	BC
4.23E-08	0.2086945	0.247	0.136	0.001014	Aagab	BC_Agedup	BC
1.60E-06	0.208307	0.3	0.193	0.0383	Lrp5	BC_Agedup	BC
5.57E-13	0.207975	0.191	0.072	1.34E-08	Dhcr24	BC_Agedup	BC
4.67E-12	0.2078569	0.147	0.049	1.12E-07	Tap2	BC_Agedup	BC
5.63E-07	0.2077415	0.186	0.098	0.013491	Col16a1	BC_Agedup	BC
2.43E-07	0.2064206	0.3	0.184	0.005837	Arhgef4	BC_Agedup	BC
2.14E-09	0.2061339	0.226	0.113	5.14E-05	Gpc1	BC_Agedup	BC
4.96E-11	0.2043966	0.981	0.994	1.19E-06	Atpif1	BC_Agedup	BC
9.53E-23	0.2043614	1	1	2.28E-18	Rps26	BC_Agedup	BC
1.66E-07	0.2034542	0.163	0.079	0.003974	Pcdh19	BC_Agedup	BC
1.19E-13	0.2031988	0.109	0.026	2.85E-09	Nptx1	BC_Agedup	BC
8.61E-08	0.2025465	0.953	0.973	0.002065	Atp5g1	BC_Agedup	BC
3.34E-07	0.2024384	0.288	0.177	0.008006	Mreg	BC_Agedup	BC
1.59E-07	0.202072	0.258	0.149	0.003808	Srebf1	BC_Agedup	BC
8.81E-09	0.2006927	0.212	0.106	0.000211	Ikbke	BC_Agedup	BC
1.03E-07	-0.200312	0.102	0.215	0.002469	Rbm4b	BC_Ageddown	BC
6.71E-08	-0.201335	0.109	0.23	0.001608	Pgap1	BC_Ageddown	BC
6.33E-07	-0.201791	0.123	0.227	0.015183	Ddit3	BC_Ageddown	BC
1.04E-07	-0.202772	0.537	0.662	0.002489	Spcs2	BC_Ageddown	BC
1.03E-06	-0.204754	0.214	0.324	0.024635	Lsm1	BC_Ageddown	BC
1.34E-06	-0.205335	0.156	0.265	0.032168	Ssfa2	BC_Ageddown	BC
1.21E-07	-0.207917	0.988	0.994	0.002901	Fxyd3	BC_Ageddown	BC
1.36E-06	-0.208016	0.614	0.713	0.032504	Atp6v0b	BC_Ageddown	BC
2.58E-07	-0.208063	0.247	0.373	0.006178	Vamp3	BC_Ageddown	BC
4.47E-08	-0.209014	0.023	0.109	0.001072	2300002M23Rik	BC_Ageddown	BC
2.32E-07	-0.209452	0.535	0.665	0.005573	Magoh	BC_Ageddown	BC
9.81E-08	-0.21018	0.953	0.988	0.002353	Eif5a	BC_Ageddown	BC

1.66E-06	-0.210248	0.316	0.434	0.039856	C1d	BC_Ageddown	BC
1.31E-07	-0.210311	0.558	0.687	0.003153	Psmd8	BC_Ageddown	BC
6.50E-07	-0.210346	0.112	0.218	0.015598	Gt(ROSA)26Sor	BC_Ageddown	BC
3.85E-13	-0.210677	1	1	9.22E-09	Npm1	BC_Ageddown	BC
2.17E-07	-0.210683	0.967	0.989	0.005192	Hspe1	BC_Ageddown	BC
7.06E-30	-0.210925	1	1	1.69E-25	Rpl23	BC_Ageddown	BC
1.18E-08	-0.212308	0.621	0.751	0.000282	Scp2	BC_Ageddown	BC
1.05E-20	-0.212345	0.998	1	2.52E-16	Rps18	BC_Ageddown	BC
2.00E-06	-0.212635	0.221	0.33	0.047937	2610001J05Rik	BC_Ageddown	BC
4.46E-08	-0.212704	0.081	0.192	0.00107	Otud1	BC_Ageddown	BC
1.93E-07	-0.213749	0.414	0.547	0.004631	Psmd12	BC_Ageddown	BC
5.77E-08	-0.213762	0.372	0.519	0.001385	Syf2	BC_Ageddown	BC
9.68E-08	-0.215073	0.663	0.77	0.002322	Paip2	BC_Ageddown	BC
5.89E-07	-0.215324	0.719	0.803	0.01412	Snu13	BC_Ageddown	BC
5.04E-08	-0.215926	0.842	0.907	0.001207	Clta	BC_Ageddown	BC
3.34E-07	-0.216218	0.142	0.251	0.008018	Mrps6	BC_Ageddown	BC
1.31E-06	-0.21651	0.186	0.297	0.031476	Atg5	BC_Ageddown	BC
2.68E-08	-0.21651	0.137	0.267	0.000644	Nkx2-3	BC_Ageddown	BC
1.64E-08	-0.217228	0.177	0.317	0.000394	Rnf11	BC_Ageddown	BC
2.05E-06	-0.217995	0.195	0.297	0.049139	Irf2	BC_Ageddown	BC
2.96E-07	-0.218144	0.128	0.24	0.007107	Errfi1	BC_Ageddown	BC
2.85E-07	-0.218322	0.784	0.868	0.006832	Ptges3	BC_Ageddown	BC
7.89E-07	-0.219196	0.547	0.675	0.018917	Polr2j	BC_Ageddown	BC
7.22E-08	-0.219844	0.458	0.598	0.001732	Psmc6	BC_Ageddown	BC
1.39E-09	-0.22021	0.963	0.988	3.33E-05	Serbp1	BC_Ageddown	BC
1.11E-06	-0.220395	0.614	0.7	0.026703	Tmed9	BC_Ageddown	BC
7.55E-08	-0.222528	0.205	0.333	0.00181	Desi2	BC_Ageddown	BC
1.31E-06	-0.223087	0.437	0.55	0.031411	Stip1	BC_Ageddown	BC
8.51E-09	-0.223357	0.867	0.929	0.000204	Ppib	BC_Ageddown	BC
9.84E-09	-0.224122	0.177	0.32	0.000236	Mpst	BC_Ageddown	BC
9.22E-08	-0.224958	0.526	0.641	0.002211	Ppig	BC_Ageddown	BC
5.79E-09	-0.225155	0.281	0.432	0.000139	Casz1	BC_Ageddown	BC
1.06E-07	-0.225162	0.153	0.272	0.002548	Wee1	BC_Ageddown	BC
1.58E-08	-0.225463	0.158	0.295	0.000378	Slit3	BC_Ageddown	BC
1.02E-06	-0.225763	0.079	0.173	0.024495	Frem2	BC_Ageddown	BC
1.03E-06	-0.227395	0.172	0.29	0.024791	Smad7	BC_Ageddown	BC
5.68E-09	-0.228404	0.972	0.994	0.000136	Prdx1	BC_Ageddown	BC
1.11E-07	-0.228736	0.277	0.408	0.002651	Wdr89	BC_Ageddown	BC
4.73E-07	-0.229147	0.763	0.847	0.011354	Tax1bp1	BC_Ageddown	BC
1.44E-06	-0.229837	0.472	0.582	0.034549	Pura	BC_Ageddown	BC
2.66E-07	-0.23001	0.495	0.616	0.006379	Dnajb6	BC_Ageddown	BC
1.18E-09	-0.230757	0.937	0.95	2.82E-05	Klf5	BC_Ageddown	BC
4.51E-08	-0.231018	0.07	0.174	0.001082	Bdnf	BC_Ageddown	BC
3.62E-07	-0.231481	0.335	0.459	0.008675	Yipf4	BC_Ageddown	BC
5.32E-07	-0.231662	0.484	0.608	0.01277	Bcap31	BC_Ageddown	BC
4.55E-07	-0.232413	0.179	0.296	0.010919	Dusp22	BC_Ageddown	BC
6.01E-07	-0.232493	0.558	0.653	0.014417	Klf3	BC_Ageddown	BC
1.30E-07	-0.233015	0.27	0.404	0.003106	Bub3	BC_Ageddown	BC
4.94E-08	-0.233104	0.447	0.6	0.001186	Nxn	BC_Ageddown	BC
5.08E-07	-0.233367	0.872	0.948	0.012176	Anp32b	BC_Ageddown	BC
6.30E-08	-0.233718	0.912	0.964	0.001512	Hmgn1	BC_Ageddown	BC
5.47E-09	-0.234634	0.365	0.506	0.000131	Tmem65	BC_Ageddown	BC
4.49E-09	-0.235101	0.405	0.543	0.000108	Mtpn	BC_Ageddown	BC
4.21E-07	-0.235479	0.33	0.452	0.010096	Ahsa1	BC_Ageddown	BC
1.01E-06	-0.236116	0.633	0.736	0.024338	Serpinb6a	BC_Ageddown	BC
2.75E-08	-0.236872	0.602	0.751	0.000661	Stmn1	BC_Ageddown	BC
3.10E-16	-0.237828	0.995	1	7.43E-12	Btf3	BC_Ageddown	BC
8.91E-11	-0.238128	0.956	0.99	2.14E-06	Gapdh	BC_Ageddown	BC
1.78E-07	-0.239255	0.351	0.468	0.004258	Polr2h	BC_Ageddown	BC

1.84E-32	-0.240744	1	1	4.42E-28	Rplp0	BC_Ageddown	BC
8.79E-08	-0.241842	0.216	0.348	0.002108	Rbms3	BC_Ageddown	BC
1.08E-21	-0.24212	1	1	2.59E-17	mt-Nd1	BC_Ageddown	BC
1.17E-08	-0.24242	0.733	0.813	0.000281	Cct5	BC_Ageddown	BC
2.28E-30	-0.243604	1	1	5.47E-26	Rpl15	BC_Ageddown	BC
3.73E-08	-0.246476	0.328	0.46	0.000894	Cfdp1	BC_Ageddown	BC
1.18E-08	-0.247667	0.647	0.764	0.000283	Cct7	BC_Ageddown	BC
3.11E-08	-0.248075	0.312	0.453	0.000746	Dnajc21	BC_Ageddown	BC
6.28E-08	-0.248611	0.226	0.357	0.001505	Gpatch4	BC_Ageddown	BC
9.87E-16	-0.249303	0.995	1	2.37E-11	Rps17	BC_Ageddown	BC
6.73E-09	-0.250153	0.316	0.461	0.000161	Tprgl	BC_Ageddown	BC
5.09E-09	-0.250253	0.388	0.527	0.000122	Elavl1	BC_Ageddown	BC
3.20E-23	-0.250281	0.991	1	7.68E-19	Naca	BC_Ageddown	BC
1.90E-08	-0.25114	0.098	0.215	0.000456	Dnajb9	BC_Ageddown	BC
6.19E-14	-0.25173	0.956	0.992	1.48E-09	mt-Nd3	BC_Ageddown	BC
2.04E-09	-0.252094	0.814	0.885	4.90E-05	Hnrnpa0	BC_Ageddown	BC
7.44E-08	-0.252141	0.488	0.608	0.001783	Dynlt3	BC_Ageddown	BC
2.45E-07	-0.252184	0.365	0.476	0.00587	Eif4e2	BC_Ageddown	BC
2.16E-07	-0.252305	0.633	0.71	0.005169	Spop	BC_Ageddown	BC
5.98E-09	-0.252688	0.212	0.353	0.000143	Med19	BC_Ageddown	BC
8.46E-09	-0.253198	0.442	0.577	0.000203	Txnl1	BC_Ageddown	BC
3.76E-32	-0.254559	1	1	9.01E-28	Rps3a1	BC_Ageddown	BC
5.97E-09	-0.254823	0.123	0.248	0.000143	Pawr	BC_Ageddown	BC
9.39E-09	-0.255002	0.477	0.602	0.000225	Map2k2	BC_Ageddown	BC
1.27E-07	-0.255714	0.426	0.565	0.00305	Cux1	BC_Ageddown	BC
2.30E-08	-0.256672	0.73	0.821	0.000551	Rab10	BC_Ageddown	BC
1.58E-07	-0.257	0.147	0.263	0.003788	Rhob	BC_Ageddown	BC
1.53E-12	-0.257453	0.951	0.981	3.68E-08	Eif3f	BC_Ageddown	BC
4.83E-10	-0.25761	0.165	0.315	1.16E-05	Gm9493	BC_Ageddown	BC
6.68E-09	-0.258198	0.47	0.602	0.00016	Bcas2	BC_Ageddown	BC
4.36E-08	-0.259716	0.447	0.579	0.001045	Arf4	BC_Ageddown	BC
1.40E-08	-0.260001	0.5	0.624	0.000335	Pop5	BC_Ageddown	BC
1.86E-08	-0.260495	0.367	0.511	0.000446	Cd24a	BC_Ageddown	BC
1.80E-07	-0.260568	0.635	0.731	0.004318	Ube2i	BC_Ageddown	BC
2.25E-08	-0.260774	0.456	0.578	0.000539	Arpc5	BC_Ageddown	BC
9.41E-30	-0.261092	0.998	1	2.26E-25	Rpl3	BC_Ageddown	BC
1.03E-08	-0.261322	0.465	0.613	0.000248	2900026A02Rik	BC_Ageddown	BC
8.45E-09	-0.261392	0.428	0.595	0.000203	Aqp3	BC_Ageddown	BC
9.00E-09	-0.263172	0.605	0.747	0.000216	Taldo1	BC_Ageddown	BC
1.18E-08	-0.263649	0.635	0.732	0.000283	Rp9	BC_Ageddown	BC
1.54E-08	-0.265043	0.33	0.468	0.000369	Lancl1	BC_Ageddown	BC
5.70E-10	-0.266766	0.847	0.901	1.37E-05	Hras	BC_Ageddown	BC
4.09E-08	-0.267872	0.435	0.556	0.000982	Mrpl42	BC_Ageddown	BC
5.61E-09	-0.268353	0.788	0.855	0.000134	Gabarap	BC_Ageddown	BC
1.43E-08	-0.268947	0.258	0.406	0.000343	Sox21	BC_Ageddown	BC
1.25E-24	-0.27048	1	1	2.99E-20	Ptma	BC_Ageddown	BC
2.85E-12	-0.270658	0.126	0.289	6.83E-08	Ahsa2	BC_Ageddown	BC
1.31E-10	-0.27119	0.009	0.108	3.14E-06	Klf2	BC_Ageddown	BC
1.11E-09	-0.271598	0.6	0.709	2.66E-05	Dctn3	BC_Ageddown	BC
2.76E-13	-0.272722	0.93	0.981	6.62E-09	Pfdn5	BC_Ageddown	BC
1.72E-06	-0.274292	0.442	0.549	0.041147	Hspa4l	BC_Ageddown	BC
2.17E-34	-0.275025	1	1	5.21E-30	Rpsa	BC_Ageddown	BC
1.25E-11	-0.276761	0.409	0.59	2.99E-07	Tnrc6c	BC_Ageddown	BC
1.87E-09	-0.277247	0.433	0.581	4.50E-05	Srpk2	BC_Ageddown	BC
1.31E-12	-0.278129	0.056	0.197	3.14E-08	Sertad1	BC_Ageddown	BC
6.25E-10	-0.278588	0.651	0.737	1.50E-05	Ncor1	BC_Ageddown	BC
1.12E-10	-0.278727	0.656	0.776	2.69E-06	Srsf11	BC_Ageddown	BC
4.35E-10	-0.278974	0.149	0.292	1.04E-05	Acsl1	BC_Ageddown	BC
2.28E-09	-0.278984	0.058	0.172	5.47E-05	Nr4a2	BC_Ageddown	BC

2.03E-06	-0.279447	0.386	0.501	0.048734	Mmp2	BC_Ageddown	BC
2.91E-08	-0.280324	0.547	0.661	0.000699	Tacstd2	BC_Ageddown	BC
3.03E-10	-0.28099	0.058	0.18	7.27E-06	Ntf3	BC_Ageddown	BC
1.80E-09	-0.282189	0.377	0.529	4.31E-05	Gadd45gip1	BC_Ageddown	BC
1.64E-08	-0.282416	0.456	0.588	0.000392	Cd81	BC_Ageddown	BC
8.67E-10	-0.283676	0.328	0.478	2.08E-05	Rap1b	BC_Ageddown	BC
1.68E-07	-0.285195	0.091	0.194	0.004036	Cldn1	BC_Ageddown	BC
1.50E-14	-0.285344	0.912	0.974	3.60E-10	Eif3k	BC_Ageddown	BC
4.08E-09	-0.286732	0.051	0.162	9.77E-05	Plk2	BC_Ageddown	BC
2.00E-10	-0.287014	0.586	0.725	4.79E-06	Glrx3	BC_Ageddown	BC
4.14E-10	-0.287115	0.774	0.869	9.92E-06	Rtraf	BC_Ageddown	BC
1.14E-09	-0.287402	0.802	0.88	2.73E-05	MuClm	BC_Ageddown	BC
1.03E-09	-0.287978	0.298	0.445	2.48E-05	Chordc1	BC_Ageddown	BC
9.16E-08	-0.28835	0.363	0.49	0.002197	Mapk6	BC_Ageddown	BC
2.98E-08	-0.288363	0.493	0.608	0.000715	Eid1	BC_Ageddown	BC
1.45E-09	-0.288743	0.498	0.66	3.49E-05	Prmt1	BC_Ageddown	BC
8.70E-11	-0.289194	0.084	0.221	2.09E-06	Cited2	BC_Ageddown	BC
6.22E-12	-0.289307	0.386	0.552	1.49E-07	Sri	BC_Ageddown	BC
1.50E-09	-0.2894	0.281	0.426	3.61E-05	Bcl10	BC_Ageddown	BC
1.26E-12	-0.289812	0.823	0.915	3.02E-08	Atp5c1	BC_Ageddown	BC
8.06E-09	-0.291765	0.974	0.983	0.000193	Gsta4	BC_Ageddown	BC
1.87E-10	-0.292632	0.528	0.671	4.49E-06	Actr3	BC_Ageddown	BC
7.90E-10	-0.293308	0.123	0.257	1.90E-05	Sdf2l1	BC_Ageddown	BC
1.53E-37	-0.294221	0.998	1	3.66E-33	Rps6	BC_Ageddown	BC
2.99E-08	-0.295125	0.379	0.507	0.000716	Carnmt1	BC_Ageddown	BC
6.49E-11	-0.295356	0.263	0.441	1.56E-06	Herpud1	BC_Ageddown	BC
3.12E-13	-0.296379	0.263	0.464	7.49E-09	Nr1d2	BC_Ageddown	BC
1.17E-10	-0.296851	0.44	0.609	2.80E-06	Ssbp2	BC_Ageddown	BC
5.62E-14	-0.296854	0.13	0.32	1.35E-09	Banp	BC_Ageddown	BC
3.20E-11	-0.296866	0.547	0.677	7.68E-07	Psmd4	BC_Ageddown	BC
2.24E-16	-0.296976	0.047	0.221	5.38E-12	Ang	BC_Ageddown	BC
1.51E-10	-0.297585	0.274	0.432	3.62E-06	Actr10	BC_Ageddown	BC
8.60E-09	-0.299122	0.537	0.656	0.000206	Ythdc1	BC_Ageddown	BC
2.90E-13	-0.299387	0.937	0.969	6.95E-09	Ddx5	BC_Ageddown	BC
5.98E-08	-0.299433	0.521	0.64	0.001433	Gltp	BC_Ageddown	BC
2.25E-11	-0.300127	0.43	0.576	5.39E-07	Rheb	BC_Ageddown	BC
3.95E-10	-0.30088	0.421	0.561	9.47E-06	Smap1	BC_Ageddown	BC
1.41E-11	-0.301286	0.214	0.384	3.39E-07	Tmem33	BC_Ageddown	BC
1.49E-06	-0.301937	0.453	0.543	0.035806	Mcl1	BC_Ageddown	BC
5.81E-09	-0.30251	0.305	0.444	0.000139	Ankrd12	BC_Ageddown	BC
1.79E-11	-0.302557	0.784	0.859	4.30E-07	Anxa5	BC_Ageddown	BC
3.00E-08	-0.303053	0.698	0.763	0.000718	Ankrd11	BC_Ageddown	BC
4.35E-10	-0.303493	0.126	0.265	1.04E-05	Sema3c	BC_Ageddown	BC
4.99E-14	-0.304259	0.73	0.828	1.20E-09	Pcbp1	BC_Ageddown	BC
9.06E-12	-0.304322	0.826	0.907	2.17E-07	Eif3i	BC_Ageddown	BC
3.06E-11	-0.304835	0.307	0.466	7.33E-07	Arpc1a	BC_Ageddown	BC
9.38E-13	-0.305936	0.021	0.147	2.25E-08	Csrnp1	BC_Ageddown	BC
6.72E-11	-0.30661	0.656	0.773	1.61E-06	Psmb2	BC_Ageddown	BC
1.06E-07	-0.307927	0.021	0.102	0.002536	Krt76	BC_Ageddown	BC
9.04E-39	-0.308124	1	1	2.17E-34	Rpl7	BC_Ageddown	BC
1.54E-11	-0.308777	0.863	0.92	3.70E-07	Hnrnpa1	BC_Ageddown	BC
1.86E-12	-0.312455	0.574	0.706	4.46E-08	Cacybp	BC_Ageddown	BC
1.78E-10	-0.312943	0.812	0.875	4.26E-06	Prdx6	BC_Ageddown	BC
1.86E-13	-0.314201	0.595	0.747	4.45E-09	Ube2b	BC_Ageddown	BC
6.13E-12	-0.316254	0.458	0.635	1.47E-07	Pten	BC_Ageddown	BC
7.02E-17	-0.316526	0.972	0.994	1.68E-12	Dynll1	BC_Ageddown	BC
2.34E-12	-0.317227	0.488	0.669	5.60E-08	Vapa	BC_Ageddown	BC
6.04E-16	-0.317433	0.853	0.929	1.45E-11	Psmb1	BC_Ageddown	BC
2.91E-13	-0.317536	0.209	0.394	6.99E-09	Spg21	BC_Ageddown	BC

7.67E-15	-0.318448	0.763	0.873	1.84E-10	Ube2d3	BC_Ageddown	BC
4.33E-11	-0.319425	0.456	0.607	1.04E-06	Vdac3	BC_Ageddown	BC
3.54E-11	-0.320283	0.695	0.829	8.48E-07	Ralbp1	BC_Ageddown	BC
2.65E-08	-0.321322	0.358	0.483	0.000636	Crlf3	BC_Ageddown	BC
6.67E-18	-0.322689	0.944	0.98	1.60E-13	Gsto1	BC_Ageddown	BC
4.97E-08	-0.323158	0.563	0.679	0.001192	Emp1	BC_Ageddown	BC
3.60E-13	-0.323667	0.174	0.363	8.64E-09	Nr1d1	BC_Ageddown	BC
6.99E-10	-0.323813	0.653	0.767	1.68E-05	Txnip	BC_Ageddown	BC
1.58E-10	-0.324155	0.572	0.689	3.79E-06	Marcks	BC_Ageddown	BC
4.69E-11	-0.324999	0.309	0.463	1.13E-06	Lsm2	BC_Ageddown	BC
2.75E-31	-0.325976	0.995	0.999	6.59E-27	Rpl29	BC_Ageddown	BC
3.27E-10	-0.326175	0.716	0.823	7.85E-06	Ccnd1	BC_Ageddown	BC
3.68E-09	-0.327238	0.577	0.698	8.81E-05	Tubb4b	BC_Ageddown	BC
4.14E-47	-0.328359	1	1	9.92E-43	Rps4x	BC_Ageddown	BC
9.21E-13	-0.328863	0.277	0.464	2.21E-08	Zfhx3	BC_Ageddown	BC
8.94E-10	-0.329669	0.121	0.258	2.14E-05	Bhlhe40	BC_Ageddown	BC
4.22E-10	-0.33032	0.621	0.719	1.01E-05	Btg1	BC_Ageddown	BC
1.49E-13	-0.330679	0.514	0.675	3.57E-09	Rbm8a	BC_Ageddown	BC
3.59E-09	-0.331619	0.97	0.978	8.62E-05	Dst	BC_Ageddown	BC
2.03E-13	-0.332626	0.781	0.88	4.88E-09	Top1	BC_Ageddown	BC
2.25E-53	-0.333412	1	1	5.40E-49	Rplp1	BC_Ageddown	BC
4.58E-17	-0.333911	0.921	0.971	1.10E-12	Prdx2	BC_Ageddown	BC
6.32E-13	-0.33483	0.76	0.854	1.52E-08	Sfr1	BC_Ageddown	BC
1.14E-10	-0.335023	0.551	0.675	2.74E-06	Tst	BC_Ageddown	BC
5.26E-11	-0.336673	0.502	0.624	1.26E-06	Atp6v1e1	BC_Ageddown	BC
8.99E-16	-0.337562	0.351	0.558	2.16E-11	Snrnp48	BC_Ageddown	BC
2.03E-08	-0.338399	0.414	0.552	0.000487	Efemp1	BC_Ageddown	BC
2.78E-15	-0.344212	0.04	0.198	6.66E-11	Prb1	BC_Ageddown	BC
3.08E-10	-0.348298	0.667	0.767	7.39E-06	Cltb	BC_Ageddown	BC
9.26E-14	-0.350278	0.607	0.757	2.22E-09	Vdac2	BC_Ageddown	BC
2.63E-12	-0.350578	0.342	0.528	6.30E-08	Pdzd2	BC_Ageddown	BC
2.05E-19	-0.3509	0.995	0.999	4.92E-15	Fth1	BC_Ageddown	BC
3.59E-13	-0.351612	0.395	0.575	8.61E-09	Snx3	BC_Ageddown	BC
8.64E-07	-0.352975	0.465	0.561	0.020728	Ccnl1	BC_Ageddown	BC
9.08E-15	-0.353863	0.751	0.852	2.18E-10	Eif5	BC_Ageddown	BC
1.73E-16	-0.355379	0.688	0.834	4.14E-12	Calr	BC_Ageddown	BC
1.86E-06	-0.355774	0.353	0.455	0.044637	Myc	BC_Ageddown	BC
1.30E-08	-0.358145	0.435	0.552	0.000312	Klf4	BC_Ageddown	BC
1.86E-13	-0.360301	0.163	0.338	4.46E-09	Tgif1	BC_Ageddown	BC
1.17E-11	-0.360883	0.14	0.298	2.80E-07	Pim3	BC_Ageddown	BC
8.47E-13	-0.36112	0.377	0.537	2.03E-08	Mettl23	BC_Ageddown	BC
1.24E-07	-0.364319	0.205	0.334	0.002966	Tgm3	BC_Ageddown	BC
2.08E-22	-0.368384	0.935	0.98	5.00E-18	Eef1d	BC_Ageddown	BC
9.53E-16	-0.369845	0.149	0.342	2.29E-11	Pnp	BC_Ageddown	BC
1.56E-16	-0.370399	0.693	0.825	3.74E-12	Arpc3	BC_Ageddown	BC
4.24E-11	-0.370465	0.365	0.54	1.02E-06	Hlf	BC_Ageddown	BC
2.85E-16	-0.371107	0.423	0.616	6.83E-12	Tnfaip8	BC_Ageddown	BC
3.34E-16	-0.373307	0.407	0.594	8.01E-12	Nudc	BC_Ageddown	BC
1.75E-78	-0.373965	1	0.999	4.20E-74	Rpl8	BC_Ageddown	BC
1.30E-16	-0.375975	0.1	0.293	3.11E-12	Coq10b	BC_Ageddown	BC
4.14E-08	-0.378913	0.965	0.987	0.000993	Actg1	BC_Ageddown	BC
7.64E-07	-0.380413	0.07	0.159	0.018326	Sostdc1	BC_Ageddown	BC
1.74E-17	-0.382101	0.693	0.833	4.16E-13	Hnrnpc	BC_Ageddown	BC
4.86E-11	-0.383027	0.444	0.586	1.16E-06	Snai2	BC_Ageddown	BC
6.18E-12	-0.383413	0.863	0.903	1.48E-07	Neat1	BC_Ageddown	BC
6.86E-12	-0.385541	0.563	0.696	1.65E-07	Sox2	BC_Ageddown	BC
2.39E-16	-0.386618	0.97	0.997	5.72E-12	Perp	BC_Ageddown	BC
1.89E-14	-0.387672	0.426	0.608	4.54E-10	Serpinh1	BC_Ageddown	BC
7.65E-25	-0.389312	0.995	1	1.83E-20	Tmsb4x	BC_Ageddown	BC

8.01E-79	-0.390771	1	1	1.92E-74	Rpl13	BC_Ageddown	BC
7.49E-13	-0.393244	0.393	0.582	1.80E-08	Foxe1	BC_Ageddown	BC
1.04E-20	-0.394536	0.858	0.932	2.49E-16	Arpc2	BC_Ageddown	BC
9.87E-21	-0.395203	0.728	0.862	2.37E-16	Rhoa	BC_Ageddown	BC
5.00E-20	-0.395222	0.86	0.95	1.20E-15	Ybx1	BC_Ageddown	BC
9.54E-15	-0.395894	0.579	0.724	2.29E-10	Ppp1cb	BC_Ageddown	BC
3.10E-53	-0.397058	1	1	7.43E-49	Rpl10	BC_Ageddown	BC
2.78E-15	-0.398283	0.444	0.617	6.66E-11	Arid5b	BC_Ageddown	BC
2.32E-16	-0.398494	0.53	0.703	5.55E-12	Tmpo	BC_Ageddown	BC
3.92E-15	-0.399495	0.235	0.428	9.41E-11	Gadd45b	BC_Ageddown	BC
3.12E-57	-0.401858	1	1	7.49E-53	Rack1	BC_Ageddown	BC
3.62E-09	-0.404116	0.312	0.443	8.68E-05	Foxq1	BC_Ageddown	BC
4.42E-47	-0.404262	1	0.999	1.06E-42	Lgals7	BC_Ageddown	BC
7.61E-12	-0.406079	0.086	0.24	1.82E-07	Krt75	BC_Ageddown	BC
6.67E-14	-0.409964	0.065	0.22	1.60E-09	Frat2	BC_Ageddown	BC
1.31E-07	-0.413557	0.256	0.375	0.003141	Nfkbiz	BC_Ageddown	BC
3.37E-12	-0.414909	0.607	0.733	8.08E-08	Cbr3	BC_Ageddown	BC
3.80E-20	-0.417228	0.679	0.822	9.12E-16	Laptm4a	BC_Ageddown	BC
1.34E-16	-0.419678	0.26	0.506	3.22E-12	Dbp	BC_Ageddown	BC
7.84E-46	-0.420789	0.998	0.999	1.88E-41	Cox4i1	BC_Ageddown	BC
1.37E-12	-0.420888	0.621	0.772	3.30E-08	Rab25	BC_Ageddown	BC
2.42E-18	-0.422518	0.493	0.68	5.81E-14	Psmd7	BC_Ageddown	BC
3.07E-13	-0.426077	0.021	0.151	7.36E-09	Cyr61	BC_Ageddown	BC
1.21E-27	-0.429736	0.949	0.981	2.90E-23	Eif4a1	BC_Ageddown	BC
2.73E-12	-0.436367	0.286	0.453	6.54E-08	Ctsl	BC_Ageddown	BC
3.78E-90	-0.437027	1	1	9.06E-86	Eef1a1	BC_Ageddown	BC
6.98E-21	-0.437249	0.616	0.808	1.67E-16	Eif3m	BC_Ageddown	BC
6.67E-15	-0.437361	0.167	0.359	1.60E-10	Klf9	BC_Ageddown	BC
3.53E-20	-0.441182	0.556	0.744	8.46E-16	Brk1	BC_Ageddown	BC
3.53E-15	-0.446717	0.521	0.691	8.48E-11	Sox4	BC_Ageddown	BC
5.15E-07	-0.447484	0.402	0.506	0.012342	Sgk1	BC_Ageddown	BC
1.64E-16	-0.453095	0.723	0.866	3.94E-12	Pycard	BC_Ageddown	BC
3.69E-35	-0.456377	0.979	0.999	8.85E-31	S100a11	BC_Ageddown	BC
1.72E-23	-0.456454	0.651	0.806	4.13E-19	Sumo1	BC_Ageddown	BC
3.36E-17	-0.456634	0.865	0.946	8.07E-13	Wnt4	BC_Ageddown	BC
2.10E-13	-0.458738	0.316	0.481	5.05E-09	Lap3	BC_Ageddown	BC
6.20E-44	-0.459003	0.984	0.999	1.49E-39	Cd9	BC_Ageddown	BC
3.58E-21	-0.46009	0.326	0.551	8.59E-17	Cldnd1	BC_Ageddown	BC
3.41E-19	-0.46249	0.481	0.668	8.19E-15	Fkbp4	BC_Ageddown	BC
6.72E-12	-0.464263	0.309	0.478	1.61E-07	Mafb	BC_Ageddown	BC
1.14E-13	-0.464347	0.07	0.225	2.73E-09	Rbp2	BC_Ageddown	BC
8.59E-39	-0.464952	0.984	0.999	2.06E-34	Itm2b	BC_Ageddown	BC
1.43E-30	-0.465715	0.842	0.964	3.42E-26	Eif3h	BC_Ageddown	BC
2.60E-23	-0.466057	0	0.197	6.23E-19	Gm8882	BC_Ageddown	BC
1.49E-21	-0.470802	0.684	0.842	3.57E-17	Cct4	BC_Ageddown	BC
5.22E-13	-0.472311	0.537	0.698	1.25E-08	Glul	BC_Ageddown	BC
6.66E-22	-0.479717	0.453	0.692	1.60E-17	Manf	BC_Ageddown	BC
3.15E-24	-0.47975	0.895	0.964	7.56E-20	S100a14	BC_Ageddown	BC
1.53E-81	-0.482048	0.998	1	3.67E-77	Rpl17	BC_Ageddown	BC
2.18E-15	-0.490742	0.323	0.506	5.22E-11	Arl4a	BC_Ageddown	BC
1.99E-13	-0.493249	0.377	0.561	4.78E-09	Il1r2	BC_Ageddown	BC
3.05E-08	-0.496651	0.07	0.18	0.000732	Krt16	BC_Ageddown	BC
8.33E-14	-0.502306	0.019	0.153	2.00E-09	Egr3	BC_Ageddown	BC
7.39E-109	-0.506554	1	1	1.77E-104	Rpl26	BC_Ageddown	BC
6.14E-21	-0.508885	0.879	0.967	1.47E-16	Dmkn	BC_Ageddown	BC
2.62E-26	-0.517394	0.488	0.721	6.27E-22	Erp29	BC_Ageddown	BC
9.99E-28	-0.519011	0.588	0.794	2.40E-23	Psma4	BC_Ageddown	BC
1.85E-32	-0.524345	0.874	0.964	4.44E-28	Pcbp2	BC_Ageddown	BC
2.42E-07	-0.525409	0.07	0.166	0.005793	Fxyd4	BC_Ageddown	BC

7.96E-38	-0.529983	0.905	0.981	1.91E-33	Slc25a5	BC_Ageddown	BC
3.86E-14	-0.534128	0.674	0.771	9.27E-10	Ubc	BC_Ageddown	BC
1.71E-25	-0.540368	0.298	0.549	4.09E-21	Zkscan3	BC_Ageddown	BC
8.19E-32	-0.550027	0.542	0.795	1.96E-27	U2af1	BC_Ageddown	BC
3.37E-14	-0.550424	0.193	0.375	8.09E-10	Adrb2	BC_Ageddown	BC
2.31E-22	-0.552444	0.5	0.697	5.54E-18	Lmo4	BC_Ageddown	BC
7.12E-21	-0.56523	0.956	0.953	1.71E-16	Krt15	BC_Ageddown	BC
1.14E-50	-0.568811	0.988	0.999	2.73E-46	H3f3b	BC_Ageddown	BC
3.17E-29	-0.573173	0.305	0.584	7.60E-25	Hspb8	BC_Ageddown	BC
1.49E-126	-0.573794	1	1	3.57E-122	Rps14	BC_Ageddown	BC
2.04E-40	-0.578753	0.86	0.967	4.89E-36	Sumo2	BC_Ageddown	BC
4.25E-31	-0.579027	0.779	0.932	1.02E-26	Serpinb5	BC_Ageddown	BC
7.33E-29	-0.581765	0.681	0.885	1.76E-24	Wfdc18	BC_Ageddown	BC
7.94E-27	-0.597895	0.728	0.882	1.90E-22	Zfp36l2	BC_Ageddown	BC
3.10E-28	-0.60199	0.293	0.568	7.44E-24	Tsc22d3	BC_Ageddown	BC
6.67E-32	-0.606395	0.093	0.393	1.60E-27	Ifi202b	BC_Ageddown	BC
8.98E-13	-0.61005	0.444	0.57	2.15E-08	Pnrc1	BC_Ageddown	BC
2.11E-19	-0.61692	0.642	0.781	5.06E-15	Sat1	BC_Ageddown	BC
5.93E-44	-0.618147	0.781	0.945	1.42E-39	Cox7a2l	BC_Ageddown	BC
5.85E-87	-0.62514	0.993	0.999	1.40E-82	Rps25	BC_Ageddown	BC
2.80E-28	-0.629521	0.67	0.843	6.72E-24	Hspa5	BC_Ageddown	BC
6.60E-160	-0.631081	1	1	1.58E-155	Rps8	BC_Ageddown	BC
2.55E-28	-0.63423	0.533	0.76	6.12E-24	Cebpb	BC_Ageddown	BC
4.02E-07	-0.639411	0.409	0.527	0.009629	Gm94	BC_Ageddown	BC
8.35E-13	-0.660072	0.051	0.192	2.00E-08	Cebpd	BC_Ageddown	BC
6.12E-55	-0.664158	0.998	0.999	1.47E-50	Rps15	BC_Ageddown	BC
3.16E-50	-0.678177	0.798	0.945	7.57E-46	Eif3e	BC_Ageddown	BC
2.92E-21	-0.681328	0.486	0.673	7.00E-17	Zfp36l1	BC_Ageddown	BC
3.63E-137	-0.704667	0.993	1	8.69E-133	Rpl21	BC_Ageddown	BC
1.09E-36	-0.723769	0.372	0.687	2.61E-32	Anxa8	BC_Ageddown	BC
9.16E-41	-0.737466	0.228	0.594	2.20E-36	Cpn1	BC_Ageddown	BC
2.27E-168	-0.737705	1	1	5.45E-164	mt-Cytb	BC_Ageddown	BC
2.47E-166	-0.754203	1	1	5.93E-162	Tpt1	BC_Ageddown	BC
1.38E-84	-0.773811	0.993	0.999	3.31E-80	Rpl23a	BC_Ageddown	BC
2.85E-19	-0.775721	0.058	0.252	6.83E-15	Ppp1r15a	BC_Ageddown	BC
1.28E-36	-0.777092	0.158	0.484	3.07E-32	Hspa1a	BC_Ageddown	BC
4.73E-140	-0.787082	0.998	1	1.13E-135	mt-Nd2	BC_Ageddown	BC
2.30E-23	-0.795675	0.898	0.93	5.51E-19	Adh7	BC_Ageddown	BC
1.97E-42	-0.79638	0.835	0.979	4.73E-38	Krt6a	BC_Ageddown	BC
1.05E-28	-0.798249	0.249	0.547	2.53E-24	2310057J18Rik	BC_Ageddown	BC
2.81E-21	-0.80965	0.065	0.279	6.73E-17	Socs3	BC_Ageddown	BC
8.69E-33	-0.809739	0.347	0.629	2.08E-28	Capns2	BC_Ageddown	BC
4.30E-99	-0.81598	0.979	1	1.03E-94	Hspa8	BC_Ageddown	BC
1.91E-139	-0.837152	0.995	1	4.57E-135	Rpl12	BC_Ageddown	BC
1.66E-20	-0.846184	0.03	0.222	3.99E-16	Nr4a1	BC_Ageddown	BC
3.12E-183	-0.849043	1	1	7.47E-179	mt-Co3	BC_Ageddown	BC
7.99E-66	-0.871977	0.247	0.742	1.92E-61	Tmem59	BC_Ageddown	BC
6.58E-196	-0.880847	1	1	1.58E-191	mt-Atp6	BC_Ageddown	BC
1.58E-147	-0.886708	0.998	1	3.80E-143	Hsp90ab1	BC_Ageddown	BC
5.60E-60	-0.88724	0.647	0.873	1.34E-55	Dnaja1	BC_Ageddown	BC
1.20E-45	-0.891578	0.586	0.802	2.88E-41	Hspa1b	BC_Ageddown	BC
2.92E-24	-0.892335	0.114	0.359	7.00E-20	Ier3	BC_Ageddown	BC
2.05E-34	-0.897579	0.151	0.464	4.91E-30	Gadd45g	BC_Ageddown	BC
2.30E-170	-0.92812	0.998	1	5.52E-166	Rps12	BC_Ageddown	BC
1.97E-146	-0.94981	0.979	1	4.72E-142	H3f3a	BC_Ageddown	BC
2.15E-28	-0.956099	0.24	0.517	5.16E-24	Nfkbia	BC_Ageddown	BC
1.29E-59	-1.011862	0.23	0.646	3.09E-55	Hsph1	BC_Ageddown	BC
8.77E-111	-1.03684	0.902	0.997	2.10E-106	Ubb	BC_Ageddown	BC
6.73E-24	-1.051319	0.391	0.605	1.61E-19	Dnajb1	BC_Ageddown	BC

1.00E-35	-1.053963	0.263	0.592	2.40E-31	Amy1	BC_Ageddown	BC
1.44E-46	-1.065689	0.647	0.879	3.45E-42	Id3	BC_Ageddown	BC
3.20E-26	-1.077242	0.402	0.616	7.67E-22	Ier2	BC_Ageddown	BC
9.30E-77	-1.141863	0.947	0.99	2.23E-72	Hspb1	BC_Ageddown	BC
9.74E-17	-1.239988	0.344	0.506	2.34E-12	Klf6	BC_Ageddown	BC
2.13E-123	-1.376974	0.849	0.983	5.11E-119	Hsp90aa1	BC_Ageddown	BC
3.62E-28	-1.414393	0.16	0.43	8.68E-24	Dusp1	BC_Ageddown	BC
2.30E-70	-1.435247	0.286	0.741	5.50E-66	Id1	BC_Ageddown	BC
2.08E-80	-1.466083	0.574	0.922	5.00E-76	Bpifb1	BC_Ageddown	BC
9.33E-29	-1.516901	0.147	0.41	2.24E-24	Zfp36	BC_Ageddown	BC
4.02E-28	-1.519378	0.43	0.647	9.64E-24	Btg2	BC_Ageddown	BC
7.75E-41	-1.523145	0.705	0.944	1.86E-36	Krt17	BC_Ageddown	BC
5.82E-15	-1.530102	0.363	0.507	1.40E-10	Egr1	BC_Ageddown	BC
1.79E-34	-1.545679	0.042	0.343	4.29E-30	Fosb	BC_Ageddown	BC
1.42E-30	-1.721249	0.495	0.687	3.41E-26	Junb	BC_Ageddown	BC
1.34E-117	-1.951082	0.681	0.983	3.22E-113	Lipf	BC_Ageddown	BC
6.99E-30	-2.11113	0.063	0.333	1.68E-25	Atf3	BC_Ageddown	BC
1.78E-42	-2.111348	0.221	0.55	4.28E-38	Jun	BC_Ageddown	BC
1.39E-121	-2.190857	0.705	0.983	3.33E-117	Sbpl	BC_Ageddown	BC
7.74E-42	-2.476209	0.109	0.459	1.86E-37	Fos	BC_Ageddown	BC
4.47E-75	3.0374382	0.49	0	1.07E-70	Xist	CBC_Agedup	CBC
2.80E-84	2.4145697	1	1	6.71E-80	Gm42418	CBC_Agedup	CBC
1.61E-49	2.1812227	0.969	0.905	3.85E-45	Crip1	CBC_Agedup	CBC
7.02E-85	1.9223626	1	0.851	1.68E-80	mt-Atp8	CBC_Agedup	CBC
5.12E-51	1.7944684	0.74	0.192	1.23E-46	Ifi27l2a	CBC_Agedup	CBC
1.38E-48	1.6641561	0.385	0.018	3.31E-44	Gm10260	CBC_Agedup	CBC
2.55E-10	1.6163485	0.703	0.938	6.13E-06	Wfdc18	CBC_Agedup	CBC
1.85E-91	1.4570264	1	1	4.44E-87	Gm10076	CBC_Agedup	CBC
2.78E-39	1.3966416	0.901	0.579	6.66E-35	H2-K1	CBC_Agedup	CBC
7.94E-79	1.3611821	1	1	1.90E-74	mt-Nd4l	CBC_Agedup	CBC
4.19E-31	1.3500807	0.911	0.808	1.01E-26	H2-D1	CBC_Agedup	CBC
1.93E-34	1.2712445	0.833	0.452	4.63E-30	B2m	CBC_Agedup	CBC
7.37E-59	1.1810499	0.391	0	1.77E-54	Tff2	CBC_Agedup	CBC
1.43E-29	1.1785311	0.823	0.538	3.44E-25	Tppp3	CBC_Agedup	CBC
7.19E-31	1.0795409	0.396	0.072	1.73E-26	1600014C10Rik	CBC_Agedup	CBC
8.17E-09	1.0745177	0.458	0.287	0.000196	Apoc1	CBC_Agedup	CBC
1.73E-92	1.0517817	1	1	4.15E-88	Rps28	CBC_Agedup	CBC
1.10E-13	1.0405228	0.74	0.564	2.63E-09	Igfbp2	CBC_Agedup	CBC
1.42E-78	0.9656851	1	1	3.39E-74	Rpl35	CBC_Agedup	CBC
4.68E-24	0.9556457	0.615	0.293	1.12E-19	Pttg1	CBC_Agedup	CBC
9.43E-28	0.942666	0.932	0.848	2.26E-23	Tmsb10	CBC_Agedup	CBC
1.42E-32	0.9306069	0.792	0.418	3.40E-28	Gstp1	CBC_Agedup	CBC
4.24E-81	0.9026361	1	1	1.02E-76	Rpl38	CBC_Agedup	CBC
1.65E-57	0.8977259	1	0.998	3.95E-53	Uba52	CBC_Agedup	CBC
3.33E-53	0.890458	0.99	0.936	7.98E-49	Snrpg	CBC_Agedup	CBC
1.44E-17	0.8894152	0.193	0.021	3.46E-13	Muc5b	CBC_Agedup	CBC
1.21E-83	0.8754085	1	1	2.91E-79	Rpl37a	CBC_Agedup	CBC
1.75E-21	0.8584835	0.922	0.849	4.20E-17	Cstb	CBC_Agedup	CBC
3.73E-87	0.8409273	1	1	8.95E-83	Rps29	CBC_Agedup	CBC
5.01E-38	0.8365446	0.943	0.805	1.20E-33	Erh	CBC_Agedup	CBC
7.94E-22	0.7965344	0.802	0.484	1.90E-17	Ly6a	CBC_Agedup	CBC
2.55E-21	0.7935119	0.995	0.997	6.13E-17	S100a6	CBC_Agedup	CBC
2.37E-38	0.7836913	0.969	0.692	5.69E-34	Ndufa3	CBC_Agedup	CBC
1.26E-39	0.7750687	0.953	0.859	3.03E-35	Ndufa1	CBC_Agedup	CBC
3.46E-39	0.7606316	0.99	0.936	8.30E-35	Mrpl52	CBC_Agedup	CBC
2.08E-22	0.7573522	0.958	0.864	4.98E-18	Crip2	CBC_Agedup	CBC
2.56E-18	0.7536316	0.318	0.075	6.15E-14	Gm26870	CBC_Agedup	CBC
2.16E-49	0.7529509	0.99	0.989	5.18E-45	Rpl27	CBC_Agedup	CBC
1.24E-34	0.7518623	0.984	0.949	2.96E-30	Atp5k	CBC_Agedup	CBC

5.68E-15	0.7512199	0.979	0.951	1.36E-10	Mt2	CBC_Agedup	CBC
1.34E-67	0.7275133	1	1	3.21E-63	Rpl37	CBC_Agedup	CBC
3.03E-18	0.7263324	0.365	0.105	7.27E-14	Gm42047	CBC_Agedup	CBC
1.12E-15	0.7232624	0.792	0.523	2.69E-11	4631405K08Rik	CBC_Agedup	CBC
1.28E-42	0.7184599	0.99	0.985	3.07E-38	Uqcr11	CBC_Agedup	CBC
1.04E-80	0.7150412	1	1	2.49E-76	Rpl41	CBC_Agedup	CBC
9.41E-68	0.6979432	1	1	2.26E-63	Rpl36	CBC_Agedup	CBC
4.34E-61	0.6968263	1	1	1.04E-56	Rps27	CBC_Agedup	CBC
7.28E-14	0.6895858	0.828	0.649	1.75E-09	Taf1d	CBC_Agedup	CBC
2.77E-15	0.6741791	0.531	0.254	6.64E-11	Hopx	CBC_Agedup	CBC
3.21E-37	0.673752	0.99	0.993	7.69E-33	Ndufa4	CBC_Agedup	CBC
2.47E-14	0.66095	0.802	0.548	5.92E-10	Ly6e	CBC_Agedup	CBC
1.42E-21	0.6572874	0.979	0.982	3.42E-17	Dbi	CBC_Agedup	CBC
7.92E-13	0.6431382	0.927	0.91	1.90E-08	Fam162a	CBC_Agedup	CBC
7.27E-20	0.6367864	0.766	0.466	1.74E-15	Lars2	CBC_Agedup	CBC
2.22E-16	0.6322485	0.672	0.393	5.33E-12	Igfbp7	CBC_Agedup	CBC
2.85E-15	0.6311696	0.922	0.815	6.83E-11	Cst3	CBC_Agedup	CBC
4.74E-16	0.6224367	0.917	0.759	1.14E-11	Sod1	CBC_Agedup	CBC
1.14E-14	0.6072594	0.625	0.334	2.72E-10	Barx2	CBC_Agedup	CBC
4.65E-07	0.5954381	0.531	0.344	0.011155	Tmem176a	CBC_Agedup	CBC
1.26E-23	0.5914487	0.781	0.451	3.03E-19	Pet100	CBC_Agedup	CBC
1.25E-36	0.5903612	0.984	0.98	3.00E-32	Tomm7	CBC_Agedup	CBC
2.27E-53	0.5844602	1	1	5.44E-49	Rps20	CBC_Agedup	CBC
2.71E-18	0.5833117	0.938	0.767	6.51E-14	Ptprs	CBC_Agedup	CBC
6.14E-14	0.5818474	0.734	0.538	1.47E-09	Flna	CBC_Agedup	CBC
9.02E-22	0.5816321	0.557	0.216	2.16E-17	Snhg20	CBC_Agedup	CBC
6.03E-36	0.5792564	0.995	0.995	1.45E-31	Cox6b1	CBC_Agedup	CBC
1.40E-21	0.578359	0.854	0.6	3.36E-17	Cenpw	CBC_Agedup	CBC
1.45E-42	0.5751543	1	1	3.48E-38	Cox6c	CBC_Agedup	CBC
1.01E-17	0.5745307	0.984	0.987	2.43E-13	Lmna	CBC_Agedup	CBC
1.05E-21	0.5682747	0.938	0.89	2.52E-17	Usmg5	CBC_Agedup	CBC
4.71E-09	0.5674497	0.75	0.557	0.000113	Apoe	CBC_Agedup	CBC
6.78E-24	0.5668845	0.995	0.926	1.63E-19	Cox6a1	CBC_Agedup	CBC
4.10E-15	0.556492	0.88	0.728	9.83E-11	Ktn1	CBC_Agedup	CBC
4.46E-10	0.5546134	0.714	0.557	1.07E-05	Gm47283	CBC_Agedup	CBC
6.49E-19	0.5533916	0.906	0.805	1.56E-14	Bola2	CBC_Agedup	CBC
1.47E-13	0.5518674	0.943	0.885	3.53E-09	Tspo	CBC_Agedup	CBC
3.85E-10	0.5380293	0.771	0.585	9.22E-06	Aspm	CBC_Agedup	CBC
1.03E-16	0.5317031	0.969	0.92	2.46E-12	Mki67	CBC_Agedup	CBC
1.18E-18	0.529619	0.948	0.877	2.84E-14	Spint2	CBC_Agedup	CBC
8.07E-08	0.5295928	0.911	0.911	0.001934	Anxa2	CBC_Agedup	CBC
3.59E-14	0.5267813	0.823	0.597	8.62E-10	Ifi27	CBC_Agedup	CBC
1.10E-13	0.5244741	0.995	0.995	2.65E-09	Mt1	CBC_Agedup	CBC
7.57E-19	0.5208877	0.495	0.184	1.82E-14	Slc7a5	CBC_Agedup	CBC
8.85E-55	0.5196978	1	1	2.12E-50	Rps19	CBC_Agedup	CBC
1.52E-06	0.5171163	0.646	0.489	0.03639	Moxd1	CBC_Agedup	CBC
8.83E-17	0.5112286	0.927	0.815	2.12E-12	Ndufb2	CBC_Agedup	CBC
3.23E-19	0.5100068	0.922	0.884	7.74E-15	Ost4	CBC_Agedup	CBC
9.32E-14	0.5090442	0.734	0.528	2.23E-09	Dynlt1f	CBC_Agedup	CBC
6.76E-14	0.5085485	0.964	0.931	1.62E-09	Reep5	CBC_Agedup	CBC
5.01E-13	0.5060798	0.953	0.818	1.20E-08	Pkp1	CBC_Agedup	CBC
4.79E-49	0.5015908	1	1	1.15E-44	Rpl39	CBC_Agedup	CBC
1.14E-15	0.5000155	0.922	0.759	2.73E-11	Romo1	CBC_Agedup	CBC
2.01E-21	0.4962701	0.979	0.951	4.81E-17	2010107E04Rik	CBC_Agedup	CBC
1.38E-21	0.4916209	0.984	0.984	3.31E-17	Uqcrq	CBC_Agedup	CBC
3.44E-15	0.4894694	0.74	0.48	8.24E-11	1810022K09Rik	CBC_Agedup	CBC
1.38E-09	0.483424	0.823	0.618	3.30E-05	Slc6a6	CBC_Agedup	CBC
6.16E-10	0.4792875	0.526	0.303	1.48E-05	Sfrp1	CBC_Agedup	CBC
4.33E-15	0.4785383	0.958	0.805	1.04E-10	Ewsr1	CBC_Agedup	CBC

5.50E-24	0.4772248	0.995	0.989	1.32E-19	Sec61g	CBC_Agedup	CBC
1.28E-28	0.4762279	0.995	0.998	3.06E-24	Cox7c	CBC_Agedup	CBC
5.02E-16	0.4718676	0.948	0.928	1.20E-11	Ndufc2	CBC_Agedup	CBC
9.86E-08	0.4707645	0.672	0.505	0.002364	Ociad2	CBC_Agedup	CBC
7.88E-10	0.4695713	0.469	0.241	1.89E-05	Iffo2	CBC_Agedup	CBC
3.31E-39	0.4690388	1	1	7.94E-35	Rpl11	CBC_Agedup	CBC
1.31E-07	0.4671347	0.401	0.226	0.003143	Chit1	CBC_Agedup	CBC
2.59E-27	0.4657135	0.995	0.998	6.21E-23	Atp5e	CBC_Agedup	CBC
2.37E-15	0.4560131	0.865	0.695	5.69E-11	Lsm7	CBC_Agedup	CBC
8.28E-11	0.4549388	0.484	0.252	1.99E-06	Car12	CBC_Agedup	CBC
1.04E-09	0.4487822	0.896	0.752	2.50E-05	Anln	CBC_Agedup	CBC
1.40E-11	0.4486843	0.797	0.613	3.36E-07	Dnajc19	CBC_Agedup	CBC
2.63E-16	0.4451459	0.974	0.949	6.31E-12	Rpl36al	CBC_Agedup	CBC
2.37E-15	0.4449596	0.958	0.887	5.68E-11	Ndufb7	CBC_Agedup	CBC
1.17E-13	0.4444097	0.88	0.785	2.81E-09	Naa38	CBC_Agedup	CBC
6.68E-08	0.4396331	0.557	0.385	0.001602	Lmo1	CBC_Agedup	CBC
1.03E-14	0.4393265	0.938	0.852	2.47E-10	Mrpl33	CBC_Agedup	CBC
1.66E-20	0.4381854	0.99	0.982	3.98E-16	Rps27l	CBC_Agedup	CBC
1.19E-34	0.4381368	1	1	2.86E-30	Rps2	CBC_Agedup	CBC
1.20E-44	0.4367178	1	1	2.87E-40	Rpl30	CBC_Agedup	CBC
4.77E-12	0.4366911	0.896	0.774	1.14E-07	Ddb1	CBC_Agedup	CBC
4.26E-08	0.4361826	0.573	0.367	0.001021	Fam213a	CBC_Agedup	CBC
2.12E-11	0.433931	0.917	0.844	5.08E-07	Cycs	CBC_Agedup	CBC
1.36E-07	0.4297328	0.948	0.902	0.003265	Krt14	CBC_Agedup	CBC
2.08E-09	0.4280411	0.849	0.78	4.99E-05	Mrpl54	CBC_Agedup	CBC
2.66E-10	0.4264131	0.917	0.862	6.38E-06	Hnrnpl	CBC_Agedup	CBC
1.42E-19	0.4258996	0.995	0.998	3.40E-15	Cox5b	CBC_Agedup	CBC
1.75E-08	0.4258098	0.776	0.695	0.00042	Churc1	CBC_Agedup	CBC
8.24E-35	0.4255017	1	1	1.98E-30	Rps26	CBC_Agedup	CBC
2.70E-18	0.4248772	0.974	0.967	6.48E-14	Ndufa2	CBC_Agedup	CBC
5.52E-26	0.424576	1	1	1.32E-21	Rpl36a	CBC_Agedup	CBC
9.25E-12	0.4229375	0.911	0.792	2.22E-07	Iqgap1	CBC_Agedup	CBC
2.60E-16	0.4228978	0.938	0.908	6.23E-12	Prelid1	CBC_Agedup	CBC
7.94E-12	0.4217151	0.823	0.707	1.90E-07	Ndufv3	CBC_Agedup	CBC
2.76E-15	0.4208663	0.594	0.287	6.62E-11	Grcc10	CBC_Agedup	CBC
1.11E-11	0.4199879	0.786	0.656	2.66E-07	Dpm3	CBC_Agedup	CBC
1.99E-16	0.4160467	0.573	0.277	4.77E-12	Rpl27-ps3	CBC_Agedup	CBC
8.43E-10	0.4156846	0.875	0.736	2.02E-05	Prrc2a	CBC_Agedup	CBC
1.96E-18	0.4144629	0.969	0.993	4.70E-14	Elob	CBC_Agedup	CBC
6.64E-12	0.4134282	0.948	0.923	1.59E-07	Son	CBC_Agedup	CBC
1.39E-21	0.4133688	1	0.997	3.33E-17	Sem1	CBC_Agedup	CBC
4.10E-12	0.4128747	0.927	0.918	9.82E-08	Ndufa5	CBC_Agedup	CBC
2.64E-14	0.412344	0.969	0.949	6.34E-10	Ndufc1	CBC_Agedup	CBC
7.02E-11	0.4110458	0.849	0.689	1.68E-06	Nop53	CBC_Agedup	CBC
1.45E-12	0.4068013	0.469	0.225	3.47E-08	Espl1	CBC_Agedup	CBC
1.91E-17	0.4053428	0.932	0.93	4.59E-13	Ndufb8	CBC_Agedup	CBC
4.84E-15	0.4046219	0.333	0.102	1.16E-10	Tap1	CBC_Agedup	CBC
6.73E-09	0.4028012	0.693	0.503	0.000161	Setd5	CBC_Agedup	CBC
1.33E-07	0.3997586	0.656	0.439	0.00318	Celsr2	CBC_Agedup	CBC
6.44E-12	0.3982516	0.63	0.354	1.54E-07	C77080	CBC_Agedup	CBC
2.05E-12	0.3966054	0.859	0.762	4.91E-08	Mrps21	CBC_Agedup	CBC
1.24E-19	0.3913102	0.193	0.016	2.97E-15	Stra6	CBC_Agedup	CBC
5.47E-12	0.3885294	0.964	0.952	1.31E-07	Ndufa11	CBC_Agedup	CBC
1.20E-09	0.387916	0.698	0.497	2.89E-05	9-Sep	CBC_Agedup	CBC
2.94E-12	0.3864923	0.922	0.856	7.05E-08	Ndufb4	CBC_Agedup	CBC
6.79E-11	0.3864758	0.719	0.526	1.63E-06	Gm11808	CBC_Agedup	CBC
8.26E-08	0.3824566	0.661	0.472	0.00198	Igsf9	CBC_Agedup	CBC
1.04E-08	0.3809798	0.844	0.68	0.000251	Rsrp1	CBC_Agedup	CBC
2.24E-08	0.3790967	0.964	0.954	0.000537	Cbx3	CBC_Agedup	CBC

8.36E-10	0.3774194	0.641	0.41	2.01E-05	Cep170b	CBC_Agedup	CBC
9.35E-10	0.3769444	0.495	0.267	2.24E-05	Plbd1	CBC_Agedup	CBC
5.47E-09	0.3766927	0.781	0.644	0.000131	Sap18	CBC_Agedup	CBC
2.03E-10	0.3757872	0.62	0.39	4.87E-06	Pam16	CBC_Agedup	CBC
6.66E-16	0.3686178	0.375	0.12	1.60E-11	Psmb9	CBC_Agedup	CBC
5.32E-08	0.3685494	0.828	0.705	0.001277	Kif23	CBC_Agedup	CBC
6.04E-08	0.3683416	0.833	0.734	0.001448	Cops9	CBC_Agedup	CBC
2.91E-08	0.3679676	0.75	0.597	0.000697	Mrps28	CBC_Agedup	CBC
4.04E-08	0.3650313	0.729	0.595	0.000969	Csrp1	CBC_Agedup	CBC
4.20E-07	0.3623142	0.875	0.805	0.01007	Cavin1	CBC_Agedup	CBC
2.00E-10	0.3616561	0.693	0.462	4.81E-06	Znhit1	CBC_Agedup	CBC
1.18E-07	0.3603501	0.786	0.705	0.002826	Eno1	CBC_Agedup	CBC
3.93E-07	0.359956	0.693	0.516	0.009419	Urah	CBC_Agedup	CBC
1.48E-09	0.3574782	0.776	0.646	3.54E-05	Hcfc1r1	CBC_Agedup	CBC
4.11E-08	0.3561887	0.698	0.521	0.000985	Hr	CBC_Agedup	CBC
1.11E-09	0.3561579	1	1	2.66E-05	mt-Nd5	CBC_Agedup	CBC
1.71E-07	0.3545817	0.781	0.649	0.004092	Psme2	CBC_Agedup	CBC
4.86E-18	0.3532543	0.99	1	1.16E-13	Atp5g2	CBC_Agedup	CBC
5.50E-13	0.3512187	0.344	0.121	1.32E-08	Dhcr24	CBC_Agedup	CBC
2.34E-11	0.3511846	0.995	0.989	5.62E-07	Pabpc1	CBC_Agedup	CBC
3.69E-09	0.3506559	0.651	0.446	8.86E-05	Cox17	CBC_Agedup	CBC
7.58E-12	0.3500204	0.99	0.995	1.82E-07	Cox5a	CBC_Agedup	CBC
1.82E-07	0.3491859	0.672	0.498	0.004353	Gas6	CBC_Agedup	CBC
2.46E-07	0.3487027	0.729	0.611	0.005893	Jag2	CBC_Agedup	CBC
1.70E-07	0.347908	0.682	0.523	0.004084	Ilf3	CBC_Agedup	CBC
1.29E-07	0.3438931	0.854	0.77	0.003082	Ckap2l	CBC_Agedup	CBC
1.23E-08	0.343743	0.37	0.189	0.000295	Hist2h2aa1	CBC_Agedup	CBC
2.29E-12	0.3435624	0.974	0.992	5.49E-08	Atp5j	CBC_Agedup	CBC
1.05E-11	0.3421769	0.984	0.989	2.52E-07	Uqcr10	CBC_Agedup	CBC
2.11E-16	0.3413393	0.37	0.113	5.06E-12	Gm10036	CBC_Agedup	CBC
7.61E-07	0.34098	0.88	0.839	0.018254	Sdc1	CBC_Agedup	CBC
8.86E-10	0.3394869	0.906	0.836	2.12E-05	Ndufa6	CBC_Agedup	CBC
3.58E-13	0.3393012	1	0.998	8.57E-09	Atpif1	CBC_Agedup	CBC
1.06E-08	0.3383493	0.87	0.772	0.000254	Chchd1	CBC_Agedup	CBC
1.34E-07	0.337587	0.677	0.502	0.003217	Wdr1	CBC_Agedup	CBC
1.16E-08	0.3352671	0.865	0.8	0.000278	Tmem256	CBC_Agedup	CBC
2.96E-15	0.3328711	0.359	0.111	7.10E-11	Psmb8	CBC_Agedup	CBC
2.39E-10	0.3284659	0.948	0.93	5.74E-06	Tma7	CBC_Agedup	CBC
1.28E-09	0.3275582	0.948	0.943	3.06E-05	Ndufa7	CBC_Agedup	CBC
5.61E-08	0.3259573	0.125	0.028	0.001346	Cldn3	CBC_Agedup	CBC
1.16E-06	0.3242259	0.411	0.238	0.027731	1700097N02Rik	CBC_Agedup	CBC
7.20E-09	0.3240987	0.88	0.843	0.000173	Trmt112	CBC_Agedup	CBC
2.34E-10	0.3236512	0.964	0.984	5.61E-06	Minos1	CBC_Agedup	CBC
1.12E-17	0.3229032	0.995	0.993	2.69E-13	Cox7a2	CBC_Agedup	CBC
1.13E-08	0.3226963	0.891	0.764	0.00027	Srsf5	CBC_Agedup	CBC
6.95E-07	0.3219588	0.786	0.656	0.016673	Syne2	CBC_Agedup	CBC
2.98E-07	0.3216426	0.698	0.513	0.007139	Pax9	CBC_Agedup	CBC
2.17E-08	0.3196833	0.573	0.356	0.000521	Itga3	CBC_Agedup	CBC
3.41E-08	0.3165638	0.578	0.359	0.000818	0610010K14Rik	CBC_Agedup	CBC
2.69E-11	0.3164511	0.516	0.27	6.44E-07	Polr2l	CBC_Agedup	CBC
6.76E-11	0.3157091	0.417	0.185	1.62E-06	Dvl1	CBC_Agedup	CBC
2.40E-07	0.3139691	0.562	0.372	0.005744	Cmc1	CBC_Agedup	CBC
4.50E-07	0.3130862	0.526	0.333	0.010802	Tnfrsf1a	CBC_Agedup	CBC
8.55E-07	0.3113336	0.755	0.57	0.020499	Mgst1	CBC_Agedup	CBC
1.98E-08	0.3093994	0.688	0.516	0.000476	Pin4	CBC_Agedup	CBC
8.50E-20	0.3093012	0.995	1	2.04E-15	Rps7	CBC_Agedup	CBC
6.37E-10	0.3084739	0.958	0.933	1.53E-05	Arpp19	CBC_Agedup	CBC
1.79E-10	0.3078208	0.417	0.182	4.29E-06	Calml3	CBC_Agedup	CBC
3.46E-10	0.3074467	0.37	0.159	8.30E-06	Sparc	CBC_Agedup	CBC

5.94E-22	0.3069676	1	1	1.42E-17	Rpl34	CBC_Agedup	CBC
3.60E-09	0.3066464	0.958	0.966	8.64E-05	Selenoh	CBC_Agedup	CBC
2.90E-08	0.306592	0.74	0.53	0.000697	2-Mar	CBC_Agedup	CBC
7.28E-07	0.3062491	0.365	0.208	0.017448	Notch2	CBC_Agedup	CBC
3.98E-22	0.3059451	1	1	9.54E-18	Rpl18a	CBC_Agedup	CBC
1.30E-06	0.3059346	0.99	0.997	0.031089	Krt5	CBC_Agedup	CBC
1.04E-09	0.3043743	0.99	0.977	2.48E-05	Atp5g3	CBC_Agedup	CBC
9.03E-10	0.3036454	0.995	0.99	2.17E-05	Eif4g2	CBC_Agedup	CBC
2.61E-08	0.3028663	0.943	0.931	0.000627	Ndufa13	CBC_Agedup	CBC
1.86E-06	0.3028132	0.844	0.759	0.044592	Ddx3x	CBC_Agedup	CBC
9.38E-07	0.3026514	0.74	0.608	0.022489	Cenpb	CBC_Agedup	CBC
9.25E-10	0.3011867	0.297	0.115	2.22E-05	Hist1h2br	CBC_Agedup	CBC
2.80E-09	0.3004427	0.896	0.802	6.71E-05	Cox14	CBC_Agedup	CBC
7.31E-07	0.2994015	0.682	0.523	0.017538	Fam173a	CBC_Agedup	CBC
5.24E-07	0.2991316	0.641	0.448	0.01256	Ralgps2	CBC_Agedup	CBC
8.38E-08	0.2987781	0.333	0.157	0.00201	Serpinb2	CBC_Agedup	CBC
4.33E-08	0.2970794	0.438	0.239	0.001039	Lamb2	CBC_Agedup	CBC
1.83E-07	0.2969794	0.708	0.485	0.004383	Nudcd3	CBC_Agedup	CBC
6.09E-11	0.2964048	0.995	0.998	1.46E-06	Snrpe	CBC_Agedup	CBC
9.77E-14	0.296125	1	0.997	2.34E-09	Atp5l	CBC_Agedup	CBC
2.85E-07	0.2943157	0.906	0.821	0.006843	Rex1bd	CBC_Agedup	CBC
2.23E-10	0.2923313	0.37	0.161	5.35E-06	Dynlt1a	CBC_Agedup	CBC
3.30E-08	0.2909567	0.49	0.279	0.000791	Rnf141	CBC_Agedup	CBC
1.81E-06	0.2899458	0.688	0.508	0.043485	Mybbp1a	CBC_Agedup	CBC
6.23E-07	0.2898755	0.927	0.88	0.014933	Fkbp2	CBC_Agedup	CBC
8.93E-07	0.289343	0.589	0.4	0.021413	Diaph1	CBC_Agedup	CBC
1.46E-12	0.2872387	0.198	0.041	3.50E-08	Pdlim4	CBC_Agedup	CBC
3.50E-07	0.2861951	0.505	0.308	0.008389	Lama5	CBC_Agedup	CBC
2.68E-17	0.2861564	1	1	6.42E-13	Rpl10a	CBC_Agedup	CBC
3.32E-07	0.2856296	0.37	0.193	0.007967	Cald1	CBC_Agedup	CBC
1.00E-06	0.2838204	0.448	0.251	0.024092	Sytl1	CBC_Agedup	CBC
5.25E-08	0.2834112	0.672	0.451	0.00126	Kank1	CBC_Agedup	CBC
2.82E-19	0.2829743	1	1	6.75E-15	Rpl35a	CBC_Agedup	CBC
1.78E-13	0.280465	0.969	0.998	4.26E-09	Atp5j2	CBC_Agedup	CBC
1.53E-07	0.2787869	0.37	0.193	0.003663	Gpc1	CBC_Agedup	CBC
3.66E-07	0.2770499	0.427	0.238	0.008769	Ar	CBC_Agedup	CBC
1.64E-13	0.2768572	0.255	0.064	3.94E-09	Akr1b8	CBC_Agedup	CBC
1.88E-08	0.2747904	0.562	0.325	0.000451	Nek2	CBC_Agedup	CBC
1.33E-06	0.2743399	0.599	0.39	0.031919	Iqgap3	CBC_Agedup	CBC
3.93E-07	0.2735934	0.521	0.326	0.009426	Zc3h7a	CBC_Agedup	CBC
3.35E-14	0.2721152	0.318	0.095	8.03E-10	Adat2	CBC_Agedup	CBC
1.20E-07	0.2720232	0.245	0.105	0.002868	Snhg9	CBC_Agedup	CBC
5.35E-19	0.2716716	1	1	1.28E-14	Rps13	CBC_Agedup	CBC
1.60E-12	0.2702521	0.984	1	3.83E-08	Oaz1	CBC_Agedup	CBC
4.90E-09	0.2662499	0.573	0.334	0.000118	Gm8186	CBC_Agedup	CBC
4.78E-07	0.265155	0.422	0.236	0.011465	Pdcd11	CBC_Agedup	CBC
4.24E-12	0.2638241	0.25	0.07	1.02E-07	H2-Q4	CBC_Agedup	CBC
1.08E-07	0.2616947	0.927	0.964	0.002597	Sec61b	CBC_Agedup	CBC
3.70E-12	0.2612206	0.219	0.052	8.88E-08	Cdkn2a	CBC_Agedup	CBC
1.16E-07	0.2596339	0.453	0.254	0.002777	Mnd1	CBC_Agedup	CBC
8.34E-07	0.2586807	0.635	0.439	0.019993	Hypk	CBC_Agedup	CBC
4.09E-09	0.2576648	0.396	0.189	9.81E-05	Tomm6	CBC_Agedup	CBC
9.25E-07	0.2558341	0.87	0.862	0.022176	Lsm6	CBC_Agedup	CBC
1.70E-07	0.2553692	0.385	0.205	0.004071	Myadm	CBC_Agedup	CBC
2.48E-13	0.255368	0.177	0.028	5.95E-09	H2-Q7	CBC_Agedup	CBC
1.27E-06	0.2536782	0.411	0.244	0.030478	Map2k7	CBC_Agedup	CBC
9.54E-07	0.2501254	0.479	0.284	0.022878	Egfr	CBC_Agedup	CBC
1.77E-13	0.2465662	1	1	4.24E-09	Rpl22	CBC_Agedup	CBC
1.02E-06	0.2463788	0.375	0.2	0.024365	Acaca	CBC_Agedup	CBC

1.77E-08	0.2460928	0.25	0.093	0.000423	Il1rn	CBC_Agedup	CBC
6.53E-09	0.2451635	0.995	1	0.000157	Txn1	CBC_Agedup	CBC
9.82E-07	0.2441603	0.964	0.99	0.023557	Ndufb11	CBC_Agedup	CBC
8.41E-08	0.2403796	0.26	0.108	0.002017	Dapk2	CBC_Agedup	CBC
3.28E-09	0.2401779	1	0.998	7.87E-05	Serf2	CBC_Agedup	CBC
1.10E-06	0.2357436	0.943	0.916	0.02633	2410015M20Rik	CBC_Agedup	CBC
1.73E-06	0.2347173	0.281	0.134	0.041553	Atp6v0a1	CBC_Agedup	CBC
3.55E-07	0.2342981	0.964	0.979	0.008505	Ubl5	CBC_Agedup	CBC
4.62E-07	0.2337308	0.964	0.995	0.011071	Ndufb9	CBC_Agedup	CBC
5.39E-09	0.2307774	0.344	0.152	0.000129	Ltbp3	CBC_Agedup	CBC
3.54E-15	0.2250914	1	1	8.50E-11	Rps23	CBC_Agedup	CBC
3.13E-12	0.2250724	0.271	0.079	7.51E-08	Cpxm2	CBC_Agedup	CBC
7.27E-11	0.2239226	0.177	0.038	1.74E-06	Bmp3	CBC_Agedup	CBC
1.77E-06	0.2219793	0.979	0.997	0.04235	Selenow	CBC_Agedup	CBC
1.39E-06	0.2210645	0.198	0.075	0.033385	Slc1a3	CBC_Agedup	CBC
1.84E-09	0.2206089	0.302	0.118	4.42E-05	Nme2	CBC_Agedup	CBC
1.92E-12	0.2197085	1	1	4.59E-08	Rpl6	CBC_Agedup	CBC
6.69E-15	0.213919	0.156	0.016	1.60E-10	Gm11361	CBC_Agedup	CBC
1.64E-09	0.2091901	0.188	0.051	3.94E-05	Recql4	CBC_Agedup	CBC
5.02E-10	0.2079247	0.245	0.079	1.20E-05	Lmtk2	CBC_Agedup	CBC
9.57E-07	0.2072564	0.302	0.144	0.022958	Pif1	CBC_Agedup	CBC
3.06E-09	0.2029117	0.365	0.157	7.34E-05	Phpt1	CBC_Agedup	CBC
2.61E-10	0.2025822	0.255	0.082	6.25E-06	2010300C02Rik	CBC_Agedup	CBC
5.70E-08	0.2002829	0.24	0.09	0.001367	Enpp1	CBC_Agedup	CBC
1.35E-06	-0.202588	0.167	0.356	0.032445	Eif2s3y	CBC_Ageddown	CBC
8.82E-07	-0.206058	0.974	0.99	0.02114	Ywhae	CBC_Ageddown	CBC
1.56E-06	-0.21293	0.125	0.297	0.037346	Tgfa	CBC_Ageddown	CBC
7.70E-07	-0.217929	0.068	0.223	0.018455	Pla2g12a	CBC_Ageddown	CBC
1.63E-06	-0.21861	0.37	0.533	0.039062	2610001J05Rik	CBC_Ageddown	CBC
6.63E-07	-0.220194	0.995	1	0.015911	Rps17	CBC_Ageddown	CBC
6.37E-08	-0.223383	0.036	0.2	0.001528	Aunip	CBC_Ageddown	CBC
1.75E-06	-0.228118	0.453	0.638	0.041883	Rap1b	CBC_Ageddown	CBC
1.63E-07	-0.236913	0.984	0.99	0.003918	Eef1g	CBC_Ageddown	CBC
5.62E-08	-0.238784	0.99	1	0.001348	Atp5b	CBC_Ageddown	CBC
1.82E-14	-0.239395	1	1	4.37E-10	Rps4x	CBC_Ageddown	CBC
1.64E-06	-0.239413	0.943	0.975	0.039321	Gnb2	CBC_Ageddown	CBC
1.73E-06	-0.240181	0.276	0.466	0.041537	Ppm1b	CBC_Ageddown	CBC
6.12E-11	-0.243083	1	1	1.47E-06	Rpl7	CBC_Ageddown	CBC
2.09E-07	-0.24316	0.995	0.998	0.005019	Ran	CBC_Ageddown	CBC
2.04E-06	-0.243929	0.198	0.369	0.048816	Rnf149	CBC_Ageddown	CBC
3.26E-07	-0.245067	0.958	0.989	0.007806	Mrfap1	CBC_Ageddown	CBC
6.98E-12	-0.245828	1	1	1.67E-07	Rpl10	CBC_Ageddown	CBC
1.65E-06	-0.248587	0.172	0.348	0.039585	Pim3	CBC_Ageddown	CBC
5.24E-08	-0.250051	0.896	0.962	0.001257	Hspa5	CBC_Ageddown	CBC
1.68E-06	-0.252061	0.974	0.99	0.040185	Eif5a	CBC_Ageddown	CBC
1.14E-12	-0.254767	1	1	2.73E-08	Rps6	CBC_Ageddown	CBC
1.68E-07	-0.25686	0.922	0.951	0.004027	Ube2d3	CBC_Ageddown	CBC
1.39E-06	-0.256893	0.771	0.854	0.033443	Tmed9	CBC_Ageddown	CBC
9.02E-07	-0.257248	0.516	0.695	0.021625	Eif4e	CBC_Ageddown	CBC
2.04E-06	-0.258261	0.917	0.982	0.048905	Atp5c1	CBC_Ageddown	CBC
1.25E-06	-0.259913	0.542	0.749	0.029888	Cux1	CBC_Ageddown	CBC
1.11E-17	-0.260618	1	1	2.65E-13	Rpl13	CBC_Ageddown	CBC
7.38E-07	-0.262206	0.469	0.621	0.017709	Gadd45gip1	CBC_Ageddown	CBC
1.50E-06	-0.266439	0.37	0.572	0.035909	Atp8b1	CBC_Ageddown	CBC
7.96E-07	-0.269589	0.312	0.513	0.019086	Tgif1	CBC_Ageddown	CBC
1.80E-07	-0.2701	0.37	0.569	0.004305	Carnmt1	CBC_Ageddown	CBC
1.07E-08	-0.270372	0.099	0.293	0.000257	Rbm4b	CBC_Ageddown	CBC
2.15E-07	-0.271434	0.193	0.374	0.005161	D030056L22Rik	CBC_Ageddown	CBC
1.26E-08	-0.273124	0.219	0.482	0.000303	Fosb	CBC_Ageddown	CBC

4.11E-07	-0.274382	0.177	0.359	0.009864	Gm9493	CBC_Ageddown	CBC
2.54E-09	-0.275086	0.984	1	6.08E-05	Serbp1	CBC_Ageddown	CBC
3.19E-08	-0.27533	0.224	0.436	0.000765	Mrps6	CBC_Ageddown	CBC
1.33E-08	-0.276275	0.818	0.895	0.000318	Rbm25	CBC_Ageddown	CBC
1.34E-07	-0.276901	0.151	0.348	0.003214	Per3	CBC_Ageddown	CBC
1.78E-18	-0.278047	1	1	4.26E-14	mt-Nd4	CBC_Ageddown	CBC
5.37E-17	-0.278429	1	1	1.29E-12	Rpsa	CBC_Ageddown	CBC
4.93E-07	-0.280091	0.458	0.611	0.011832	Arap2	CBC_Ageddown	CBC
4.88E-07	-0.282099	0.37	0.533	0.011713	Mphosph10	CBC_Ageddown	CBC
1.27E-06	-0.282432	0.656	0.782	0.030481	Med21	CBC_Ageddown	CBC
1.84E-06	-0.282506	0.635	0.749	0.044208	Auts2	CBC_Ageddown	CBC
2.99E-10	-0.283586	0.052	0.264	7.17E-06	Ogfrl1	CBC_Ageddown	CBC
1.69E-06	-0.284562	0.422	0.605	0.040466	Cdh13	CBC_Ageddown	CBC
8.35E-08	-0.284771	0.776	0.866	0.002004	Cct8	CBC_Ageddown	CBC
3.16E-07	-0.28495	0.359	0.549	0.007569	Gna11	CBC_Ageddown	CBC
8.43E-08	-0.285555	0.99	0.99	0.002021	Calm1	CBC_Ageddown	CBC
2.93E-07	-0.287052	0.229	0.43	0.007024	Arl6ip6	CBC_Ageddown	CBC
3.04E-07	-0.28715	0.714	0.807	0.007291	P4hb	CBC_Ageddown	CBC
1.03E-08	-0.288346	0.948	0.997	0.000247	Arf5	CBC_Ageddown	CBC
1.24E-07	-0.288956	0.297	0.477	0.002965	Atg5	CBC_Ageddown	CBC
4.64E-07	-0.28902	0.422	0.6	0.011128	Ppic	CBC_Ageddown	CBC
4.70E-08	-0.28906	0.99	0.997	0.001128	Stmn1	CBC_Ageddown	CBC
2.84E-08	-0.289305	0.167	0.366	0.000682	Gm16136	CBC_Ageddown	CBC
5.41E-07	-0.290684	0.609	0.748	0.012964	Arpc5	CBC_Ageddown	CBC
4.88E-09	-0.292174	0.682	0.848	0.000117	Pdia6	CBC_Ageddown	CBC
4.07E-09	-0.292387	0.104	0.318	9.75E-05	Pbx3	CBC_Ageddown	CBC
4.24E-07	-0.293665	0.26	0.434	0.010158	Mns1	CBC_Ageddown	CBC
3.60E-07	-0.293679	0.505	0.684	0.008623	Cdc5l	CBC_Ageddown	CBC
4.19E-09	-0.294049	0.115	0.344	0.0001	Nr1d1	CBC_Ageddown	CBC
9.65E-08	-0.294758	0.766	0.857	0.002314	Capza2	CBC_Ageddown	CBC
1.25E-06	-0.296267	0.359	0.518	0.029939	Sox21	CBC_Ageddown	CBC
5.23E-07	-0.296471	0.583	0.744	0.012552	Chd3	CBC_Ageddown	CBC
1.60E-06	-0.296532	0.719	0.811	0.038368	Vdac3	CBC_Ageddown	CBC
7.20E-08	-0.297646	0.49	0.659	0.001726	Chmp5	CBC_Ageddown	CBC
1.18E-06	-0.298407	0.729	0.823	0.028356	Mrpl42	CBC_Ageddown	CBC
8.81E-08	-0.298485	0.76	0.838	0.002112	Glrx3	CBC_Ageddown	CBC
1.44E-11	-0.29907	0.995	1	3.46E-07	Prdx1	CBC_Ageddown	CBC
1.53E-07	-0.299222	0.417	0.602	0.003671	Med19	CBC_Ageddown	CBC
9.84E-07	-0.299471	0.828	0.884	0.023604	St13	CBC_Ageddown	CBC
4.29E-07	-0.299854	0.417	0.595	0.010291	Ammecr1	CBC_Ageddown	CBC
2.95E-08	-0.300288	0.922	0.962	0.000707	Purb	CBC_Ageddown	CBC
1.74E-06	-0.300313	0.521	0.662	0.041725	Snrnp48	CBC_Ageddown	CBC
7.78E-08	-0.30051	0.172	0.364	0.001866	Ddx3y	CBC_Ageddown	CBC
1.49E-08	-0.300995	0.901	0.949	0.000358	Cox7a2l	CBC_Ageddown	CBC
1.93E-06	-0.301349	0.323	0.528	0.046326	Foxq1	CBC_Ageddown	CBC
3.37E-09	-0.302497	0.203	0.426	8.09E-05	Banp	CBC_Ageddown	CBC
3.50E-24	-0.30303	1	1	8.38E-20	Rpl8	CBC_Ageddown	CBC
3.22E-07	-0.303216	0.88	0.931	0.007714	Eif3i	CBC_Ageddown	CBC
2.55E-11	-0.303486	1	1	6.12E-07	H3f3b	CBC_Ageddown	CBC
5.19E-07	-0.304265	0.724	0.83	0.012443	Snx3	CBC_Ageddown	CBC
1.64E-18	-0.304402	1	1	3.94E-14	Rpl17	CBC_Ageddown	CBC
6.91E-08	-0.306077	0.885	0.931	0.001657	Serpinb5	CBC_Ageddown	CBC
1.62E-07	-0.306123	0.865	0.928	0.003878	Prdx6	CBC_Ageddown	CBC
4.62E-07	-0.307746	0.63	0.87	0.011087	Rbp1	CBC_Ageddown	CBC
2.75E-20	-0.307987	1	1	6.60E-16	Rplp1	CBC_Ageddown	CBC
2.60E-07	-0.309125	0.823	0.882	0.006233	Gnai2	CBC_Ageddown	CBC
2.30E-07	-0.309156	0.708	0.811	0.005525	Ppp2ca	CBC_Ageddown	CBC
5.85E-07	-0.310405	0.714	0.838	0.014031	Ncor1	CBC_Ageddown	CBC
2.91E-07	-0.310711	0.839	0.92	0.006986	Pdap1	CBC_Ageddown	CBC

2.64E-08	-0.310835	0.771	0.903	0.000633	Ube2i	CBC_Ageddown	CBC
4.26E-07	-0.311201	0.495	0.639	0.010225	Tmem165	CBC_Ageddown	CBC
4.26E-07	-0.311311	0.839	0.931	0.010219	Rdx	CBC_Ageddown	CBC
3.04E-09	-0.311485	0.932	0.972	7.28E-05	Hnrnpa1	CBC_Ageddown	CBC
1.12E-06	-0.312944	0.807	0.897	0.026928	Txnip	CBC_Ageddown	CBC
1.22E-06	-0.313969	0.578	0.685	0.029212	Exosc7	CBC_Ageddown	CBC
8.08E-08	-0.315234	0.703	0.807	0.001938	Morf4l2	CBC_Ageddown	CBC
1.86E-07	-0.315373	0.875	0.93	0.00447	Top1	CBC_Ageddown	CBC
4.16E-08	-0.316179	0.62	0.787	0.000997	Arf4	CBC_Ageddown	CBC
6.60E-11	-0.316934	0.99	1	1.58E-06	Npm1	CBC_Ageddown	CBC
2.84E-07	-0.317162	0.615	0.762	0.006804	Sri	CBC_Ageddown	CBC
1.19E-07	-0.318748	0.771	0.889	0.002864	Banf1	CBC_Ageddown	CBC
4.55E-09	-0.319586	0.896	0.948	0.000109	Cnbp	CBC_Ageddown	CBC
1.03E-08	-0.319855	0.802	0.889	0.000247	Vdac2	CBC_Ageddown	CBC
1.84E-09	-0.320072	0.318	0.526	4.42E-05	Vamp3	CBC_Ageddown	CBC
3.49E-08	-0.321343	0.661	0.803	0.000836	Psma1	CBC_Ageddown	CBC
3.71E-09	-0.321442	0.797	0.889	8.90E-05	Taldo1	CBC_Ageddown	CBC
2.06E-07	-0.321809	0.849	0.944	0.004937	Golim4	CBC_Ageddown	CBC
1.95E-08	-0.32198	0.307	0.497	0.000466	Pnp	CBC_Ageddown	CBC
4.06E-07	-0.322484	0.745	0.864	0.009737	Phf5a	CBC_Ageddown	CBC
1.47E-07	-0.323679	0.458	0.608	0.003531	Snai2	CBC_Ageddown	CBC
1.66E-08	-0.324215	0.641	0.792	0.000397	Bcap31	CBC_Ageddown	CBC
1.20E-07	-0.325544	0.344	0.557	0.002888	Polr2h	CBC_Ageddown	CBC
2.76E-10	-0.326964	0.12	0.359	6.62E-06	Cited2	CBC_Ageddown	CBC
1.69E-08	-0.327577	0.802	0.905	0.000404	Tceal9	CBC_Ageddown	CBC
5.74E-09	-0.328486	0.193	0.405	0.000138	Desi2	CBC_Ageddown	CBC
3.28E-07	-0.329602	0.385	0.551	0.007865	Zkscan3	CBC_Ageddown	CBC
8.52E-12	-0.33125	0.932	0.987	2.04E-07	Eif3h	CBC_Ageddown	CBC
4.86E-08	-0.332061	0.276	0.467	0.001166	Sdf2l1	CBC_Ageddown	CBC
2.71E-08	-0.334008	0.594	0.721	0.00065	Psmd4	CBC_Ageddown	CBC
1.27E-08	-0.334733	0.828	0.905	0.000304	Arglu1	CBC_Ageddown	CBC
6.74E-10	-0.335872	0.943	0.992	1.62E-05	Eef1d	CBC_Ageddown	CBC
3.08E-08	-0.336308	0.714	0.846	0.00074	Cct3	CBC_Ageddown	CBC
2.85E-08	-0.33687	0.339	0.508	0.000684	Rfc5	CBC_Ageddown	CBC
1.15E-09	-0.338581	0.781	0.902	2.75E-05	Aldh3a1	CBC_Ageddown	CBC
1.44E-10	-0.338992	0.88	0.948	3.45E-06	Rhoa	CBC_Ageddown	CBC
5.33E-08	-0.34015	0.615	0.78	0.001279	Imp3	CBC_Ageddown	CBC
2.17E-11	-0.340649	0.005	0.211	5.20E-07	Gm8882	CBC_Ageddown	CBC
1.66E-09	-0.342138	0.802	0.861	3.98E-05	Erp29	CBC_Ageddown	CBC
9.58E-19	-0.342767	1	1	2.30E-14	Ptma	CBC_Ageddown	CBC
9.13E-08	-0.343505	0.724	0.821	0.00219	Ppig	CBC_Ageddown	CBC
7.45E-08	-0.344358	0.484	0.656	0.001787	Hspb8	CBC_Ageddown	CBC
4.97E-11	-0.344849	0.979	0.99	1.19E-06	Hnrnpab	CBC_Ageddown	CBC
7.30E-09	-0.345086	0.385	0.577	0.000175	Bmpr1a	CBC_Ageddown	CBC
7.47E-09	-0.345087	0.401	0.605	0.000179	Ube2e3	CBC_Ageddown	CBC
7.09E-12	-0.345269	0.776	0.911	1.70E-07	Manf	CBC_Ageddown	CBC
1.13E-07	-0.349321	0.531	0.652	0.002699	Cldnd1	CBC_Ageddown	CBC
5.12E-09	-0.349816	0.49	0.707	0.000123	Dnajc9	CBC_Ageddown	CBC
2.24E-08	-0.349845	0.578	0.749	0.000536	Acp1	CBC_Ageddown	CBC
1.65E-10	-0.350575	0.911	0.97	3.96E-06	Hnrnpa0	CBC_Ageddown	CBC
3.24E-08	-0.355955	0.333	0.536	0.000777	Frmd4a	CBC_Ageddown	CBC
2.75E-10	-0.356382	0.938	0.997	6.59E-06	Prdx2	CBC_Ageddown	CBC
1.01E-07	-0.356479	0.458	0.61	0.00243	Mapk6	CBC_Ageddown	CBC
1.73E-10	-0.356492	0.734	0.869	4.14E-06	Eif3m	CBC_Ageddown	CBC
8.21E-07	-0.357623	0.375	0.541	0.019685	Tceal8	CBC_Ageddown	CBC
5.13E-07	-0.357749	0.911	0.961	0.012291	Ccdc34	CBC_Ageddown	CBC
7.41E-08	-0.360392	0.573	0.73	0.001776	Btg1	CBC_Ageddown	CBC
4.34E-10	-0.360872	0.974	0.989	1.04E-05	Gsto1	CBC_Ageddown	CBC
1.05E-30	-0.361076	0.995	1	2.51E-26	Rpl26	CBC_Ageddown	CBC

9.59E-07	-0.361226	0.552	0.716	0.022988	Tshz2	CBC_Ageddown	CBC
7.35E-09	-0.361684	0.875	0.944	0.000176	Calr	CBC_Ageddown	CBC
2.24E-13	-0.361739	0.979	0.992	5.38E-09	mt-Nd3	CBC_Ageddown	CBC
1.76E-07	-0.36217	0.432	0.585	0.004225	Dnajc21	CBC_Ageddown	CBC
2.73E-09	-0.362813	0.818	0.931	6.55E-05	Sf3b1	CBC_Ageddown	CBC
2.41E-07	-0.364098	0.547	0.702	0.005784	Tst	CBC_Ageddown	CBC
4.12E-14	-0.36524	0.276	0.58	9.89E-10	2310057J18Rik	CBC_Ageddown	CBC
7.80E-11	-0.36535	0.443	0.651	1.87E-06	Hdac1	CBC_Ageddown	CBC
1.29E-10	-0.365668	0.385	0.602	3.09E-06	Adh5	CBC_Ageddown	CBC
1.47E-08	-0.367169	0.891	0.959	0.000352	Mdh1	CBC_Ageddown	CBC
1.05E-14	-0.367657	0.99	0.998	2.51E-10	Hmgn1	CBC_Ageddown	CBC
2.08E-12	-0.368697	0.87	0.944	4.99E-08	Pcbp1	CBC_Ageddown	CBC
1.85E-09	-0.36898	0.729	0.869	4.44E-05	Anp32a	CBC_Ageddown	CBC
2.49E-08	-0.369734	0.682	0.808	0.000598	Rbbp4	CBC_Ageddown	CBC
1.01E-07	-0.371988	0.224	0.416	0.002419	Sema5a	CBC_Ageddown	CBC
6.60E-10	-0.372579	0.797	0.915	1.58E-05	Srsf11	CBC_Ageddown	CBC
4.00E-07	-0.379222	0.526	0.644	0.009599	Fzd1	CBC_Ageddown	CBC
8.47E-09	-0.379298	0.422	0.615	0.000203	Zfhx3	CBC_Ageddown	CBC
2.15E-12	-0.380209	0.969	0.99	5.16E-08	Hnrnpf	CBC_Ageddown	CBC
4.56E-09	-0.380928	0.745	0.848	0.000109	Tra2a	CBC_Ageddown	CBC
6.43E-11	-0.381044	0.885	0.961	1.54E-06	Sfr1	CBC_Ageddown	CBC
1.43E-08	-0.38111	0.562	0.726	0.000344	Arid5b	CBC_Ageddown	CBC
8.32E-10	-0.381922	0.297	0.523	1.99E-05	Rassf9	CBC_Ageddown	CBC
3.45E-11	-0.386753	0.562	0.767	8.28E-07	Lsm12	CBC_Ageddown	CBC
1.02E-15	-0.389067	0.974	0.998	2.44E-11	Itm2b	CBC_Ageddown	CBC
1.52E-08	-0.389377	0.495	0.67	0.000363	Prnp	CBC_Ageddown	CBC
8.46E-07	-0.390105	0.682	0.767	0.0203	Sox4	CBC_Ageddown	CBC
4.34E-09	-0.390222	0.656	0.774	0.000104	Rheb	CBC_Ageddown	CBC
3.47E-08	-0.39093	0.688	0.816	0.000833	Ythdc1	CBC_Ageddown	CBC
2.67E-10	-0.39816	0.911	0.98	6.40E-06	Nap1l1	CBC_Ageddown	CBC
3.26E-09	-0.398178	0.292	0.511	7.82E-05	Mpp6	CBC_Ageddown	CBC
3.23E-12	-0.398187	0.328	0.57	7.74E-08	Mettl23	CBC_Ageddown	CBC
3.29E-27	-0.399545	1	1	7.90E-23	Rack1	CBC_Ageddown	CBC
3.04E-07	-0.399647	0.859	0.898	0.007282	Lmnb1	CBC_Ageddown	CBC
2.06E-10	-0.406546	0.354	0.6	4.94E-06	Hlf	CBC_Ageddown	CBC
6.11E-13	-0.411886	0.583	0.766	1.47E-08	Elavl1	CBC_Ageddown	CBC
1.67E-10	-0.416244	0.536	0.715	4.00E-06	Pura	CBC_Ageddown	CBC
2.33E-13	-0.41707	0.312	0.559	5.59E-09	Tmem33	CBC_Ageddown	CBC
3.00E-12	-0.41787	0.906	0.954	7.19E-08	Anxa5	CBC_Ageddown	CBC
9.52E-15	-0.41842	0.974	0.997	2.28E-10	Anp32b	CBC_Ageddown	CBC
1.09E-06	-0.418431	0.234	0.402	0.026129	Frat2	CBC_Ageddown	CBC
7.88E-12	-0.42484	0.849	0.946	1.89E-07	Arpc2	CBC_Ageddown	CBC
6.14E-09	-0.431886	0.161	0.372	0.000147	Bdnf	CBC_Ageddown	CBC
2.37E-42	-0.433565	1	1	5.69E-38	Eef1a1	CBC_Ageddown	CBC
5.33E-10	-0.435707	0.38	0.579	1.28E-05	Hat1	CBC_Ageddown	CBC
2.43E-14	-0.438296	0.745	0.907	5.82E-10	Magoh	CBC_Ageddown	CBC
4.22E-11	-0.439083	0.609	0.8	1.01E-06	Zfp36l1	CBC_Ageddown	CBC
1.53E-06	-0.440368	0.891	0.939	0.036592	Anxa1	CBC_Ageddown	CBC
2.13E-07	-0.442288	0.609	0.748	0.005116	Tyms	CBC_Ageddown	CBC
2.44E-13	-0.443338	0.828	0.91	5.85E-09	Cct7	CBC_Ageddown	CBC
3.90E-13	-0.44721	0.214	0.498	9.35E-09	Gadd45b	CBC_Ageddown	CBC
4.64E-13	-0.448696	0.531	0.761	1.11E-08	Tnrc6c	CBC_Ageddown	CBC
6.39E-23	-0.450122	1	1	1.53E-18	mt-Nd1	CBC_Ageddown	CBC
1.29E-09	-0.450178	0.688	0.821	3.09E-05	Cebpb	CBC_Ageddown	CBC
6.13E-13	-0.450706	0.911	0.98	1.47E-08	Skp1a	CBC_Ageddown	CBC
2.29E-11	-0.455537	0.844	0.925	5.48E-07	Marcks	CBC_Ageddown	CBC
1.03E-15	-0.45689	0.182	0.497	2.46E-11	Nr1d2	CBC_Ageddown	CBC
3.65E-12	-0.459336	0.552	0.769	8.75E-08	Tnfaip8	CBC_Ageddown	CBC
7.00E-10	-0.463679	0.526	0.741	1.68E-05	Glul	CBC_Ageddown	CBC

1.24E-11	-0.463974	0.557	0.741	2.98E-07	Irf2bp2	CBC_Ageddown	CBC
2.29E-15	-0.464245	0.776	0.946	5.49E-11	Hnrnpc	CBC_Ageddown	CBC
7.80E-15	-0.465323	0.76	0.89	1.87E-10	Cct5	CBC_Ageddown	CBC
1.33E-06	-0.469873	0.198	0.382	0.031991	Ppp1r15a	CBC_Ageddown	CBC
1.48E-16	-0.474562	0.896	0.969	3.55E-12	Srsf2	CBC_Ageddown	CBC
4.40E-13	-0.475497	0.12	0.416	1.06E-08	Dbp	CBC_Ageddown	CBC
4.21E-20	-0.481872	0.984	0.997	1.01E-15	Eif4a1	CBC_Ageddown	CBC
3.17E-16	-0.48358	0.714	0.889	7.61E-12	Cct4	CBC_Ageddown	CBC
1.70E-06	-0.484482	0.516	0.659	0.04069	Pnrc1	CBC_Ageddown	CBC
1.14E-14	-0.485855	0.76	0.884	2.74E-10	Psma4	CBC_Ageddown	CBC
2.03E-13	-0.48632	0.635	0.807	4.86E-09	Prmt1	CBC_Ageddown	CBC
1.51E-16	-0.49059	0.693	0.875	3.61E-12	Brk1	CBC_Ageddown	CBC
4.24E-18	-0.492109	0.911	0.982	1.02E-13	Ptges3	CBC_Ageddown	CBC
5.73E-10	-0.492323	0.37	0.566	1.37E-05	Capns2	CBC_Ageddown	CBC
3.39E-15	-0.493233	0.781	0.902	8.12E-11	U2af1	CBC_Ageddown	CBC
9.13E-16	-0.495151	0.193	0.505	2.19E-11	Ahsa2	CBC_Ageddown	CBC
1.03E-16	-0.496596	0.734	0.902	2.48E-12	Rbm8a	CBC_Ageddown	CBC
2.66E-14	-0.498923	0.656	0.859	6.37E-10	Psmd7	CBC_Ageddown	CBC
1.45E-09	-0.500159	0.844	0.954	3.48E-05	Pclaf	CBC_Ageddown	CBC
7.83E-16	-0.500988	0.672	0.874	1.88E-11	Stip1	CBC_Ageddown	CBC
8.57E-12	-0.503199	0.339	0.579	2.05E-07	Lap3	CBC_Ageddown	CBC
4.93E-17	-0.503223	0.318	0.684	1.18E-12	Tsc22d3	CBC_Ageddown	CBC
3.77E-52	-0.506339	1	1	9.03E-48	Rps14	CBC_Ageddown	CBC
5.43E-16	-0.509669	0.682	0.895	1.30E-11	Lsm2	CBC_Ageddown	CBC
5.52E-07	-0.513875	0.245	0.439	0.013247	Ier3	CBC_Ageddown	CBC
5.95E-10	-0.516192	0.276	0.516	1.43E-05	Pax1	CBC_Ageddown	CBC
4.00E-08	-0.519226	0.411	0.608	0.00096	Ptch1	CBC_Ageddown	CBC
6.17E-19	-0.519443	0.854	0.961	1.48E-14	Eif5	CBC_Ageddown	CBC
3.48E-22	-0.520568	0.99	0.997	8.35E-18	Hspe1	CBC_Ageddown	CBC
1.73E-18	-0.523992	0.906	0.979	4.14E-14	Tmpo	CBC_Ageddown	CBC
2.48E-10	-0.525868	0.271	0.513	5.94E-06	Lxn	CBC_Ageddown	CBC
1.12E-12	-0.529965	0.38	0.633	2.68E-08	Epha7	CBC_Ageddown	CBC
5.71E-18	-0.533587	0.745	0.892	1.37E-13	Spop	CBC_Ageddown	CBC
2.79E-22	-0.53635	0.781	0.93	6.70E-18	Laptm4a	CBC_Ageddown	CBC
1.62E-14	-0.539364	0.906	0.97	3.88E-10	MuClm	CBC_Ageddown	CBC
7.50E-18	-0.539989	0.911	0.966	1.80E-13	Hspd1	CBC_Ageddown	CBC
9.47E-15	-0.541409	0.958	0.985	2.27E-10	Gsta4	CBC_Ageddown	CBC
6.27E-37	-0.542992	1	1	1.50E-32	Cox4i1	CBC_Ageddown	CBC
9.37E-10	-0.54617	0.453	0.634	2.25E-05	Efemp1	CBC_Ageddown	CBC
1.61E-19	-0.549392	0.734	0.898	3.87E-15	Sumo1	CBC_Ageddown	CBC
6.43E-27	-0.549481	0.964	0.997	1.54E-22	Slc25a5	CBC_Ageddown	CBC
1.32E-24	-0.551096	0.943	0.99	3.16E-20	Pcbp2	CBC_Ageddown	CBC
7.96E-15	-0.558788	0.115	0.41	1.91E-10	Lgr5	CBC_Ageddown	CBC
7.25E-36	-0.565944	0.995	1	1.74E-31	Rps25	CBC_Ageddown	CBC
1.11E-30	-0.577823	0.984	1	2.65E-26	Cd9	CBC_Ageddown	CBC
9.30E-21	-0.598254	0.708	0.884	2.23E-16	Nudc	CBC_Ageddown	CBC
8.77E-29	-0.598266	0.974	0.998	2.10E-24	Ybx1	CBC_Ageddown	CBC
1.72E-71	-0.601633	1	1	4.12E-67	Rps8	CBC_Ageddown	CBC
1.99E-23	-0.60823	0.802	0.961	4.77E-19	Eif3e	CBC_Ageddown	CBC
7.79E-14	-0.610077	0.786	0.89	1.87E-09	Tcf4	CBC_Ageddown	CBC
8.52E-15	-0.623153	0.818	0.916	2.04E-10	Ubc	CBC_Ageddown	CBC
1.68E-18	-0.630341	0.599	0.841	4.03E-14	Dnajb1	CBC_Ageddown	CBC
7.98E-20	-0.630526	0.547	0.782	1.91E-15	Serpinh1	CBC_Ageddown	CBC
3.33E-58	-0.635955	0.995	1	7.99E-54	Rpl21	CBC_Ageddown	CBC
1.84E-41	-0.637571	0.552	0.939	4.42E-37	Bpifb1	CBC_Ageddown	CBC
1.06E-16	-0.637686	0.536	0.766	2.54E-12	Anxa8	CBC_Ageddown	CBC
4.25E-14	-0.645151	0.76	0.877	1.02E-09	Sox2	CBC_Ageddown	CBC
7.76E-17	-0.645348	0.495	0.782	1.86E-12	Foxe1	CBC_Ageddown	CBC
4.03E-71	-0.64847	1	1	9.65E-67	Tpt1	CBC_Ageddown	CBC

3.23E-18	-0.652297	0.208	0.589	7.74E-14	Gadd45g	CBC_Ageddown	CBC
2.69E-23	-0.653665	0.359	0.715	6.45E-19	Chordc1	CBC_Ageddown	CBC
2.38E-18	-0.655217	0.146	0.489	5.71E-14	Ifi202b	CBC_Ageddown	CBC
1.67E-17	-0.657153	0.75	0.934	4.01E-13	Cltb	CBC_Ageddown	CBC
4.73E-08	-0.663112	0.844	0.872	0.001135	Neat1	CBC_Ageddown	CBC
1.26E-33	-0.664155	0.953	1	3.02E-29	Sumo2	CBC_Ageddown	CBC
3.66E-08	-0.667724	0.297	0.507	0.000879	Zfp36	CBC_Ageddown	CBC
1.38E-30	-0.671808	1	1	3.30E-26	Rps15	CBC_Ageddown	CBC
2.13E-09	-0.69837	0.219	0.464	5.10E-05	Sptssb	CBC_Ageddown	CBC
5.32E-22	-0.745745	0.75	0.895	1.28E-17	Lmo4	CBC_Ageddown	CBC
6.23E-16	-0.748375	0.375	0.656	1.50E-11	Nfkbia	CBC_Ageddown	CBC
3.91E-29	-0.75037	0.75	0.913	9.38E-25	Cacybp	CBC_Ageddown	CBC
3.37E-18	-0.751399	0.719	0.887	8.09E-14	Ccnd2	CBC_Ageddown	CBC
1.21E-41	-0.763307	0.99	1	2.90E-37	Rpl23a	CBC_Ageddown	CBC
9.09E-09	-0.773383	0.141	0.362	0.000218	Wif1	CBC_Ageddown	CBC
2.31E-12	-0.775591	0.5	0.743	5.55E-08	Serpine2	CBC_Ageddown	CBC
3.87E-61	-0.780588	1	1	9.28E-57	H3f3a	CBC_Ageddown	CBC
2.81E-15	-0.799943	0.229	0.572	6.75E-11	Krt75	CBC_Ageddown	CBC
1.21E-64	-0.824486	0.995	1	2.91E-60	Rpl12	CBC_Ageddown	CBC
3.24E-80	-0.856082	1	1	7.76E-76	mt-Cytb	CBC_Ageddown	CBC
6.88E-71	-0.863331	0.995	1	1.65E-66	Rps12	CBC_Ageddown	CBC
4.18E-39	-0.891284	0.646	0.938	1.00E-34	Fkbp4	CBC_Ageddown	CBC
2.46E-68	-0.901911	1	1	5.89E-64	mt-Nd2	CBC_Ageddown	CBC
1.39E-83	-0.903836	1	1	3.34E-79	mt-Atp6	CBC_Ageddown	CBC
4.85E-07	-0.937823	0.432	0.595	0.01164	Egr1	CBC_Ageddown	CBC
1.85E-57	-0.948578	0.76	0.995	4.44E-53	Lipf	CBC_Ageddown	CBC
1.87E-26	-0.952671	0.854	0.969	4.48E-22	Adh7	CBC_Ageddown	CBC
1.54E-14	-0.981603	0.312	0.654	3.69E-10	Fos	CBC_Ageddown	CBC
1.76E-11	-0.994503	0.536	0.734	4.22E-07	Junb	CBC_Ageddown	CBC
7.29E-38	-1.019586	0.917	0.987	1.75E-33	Ptn	CBC_Ageddown	CBC
2.50E-80	-1.023551	1	1	6.00E-76	mt-Co3	CBC_Ageddown	CBC
9.21E-31	-1.051867	0.734	0.975	2.21E-26	Id3	CBC_Ageddown	CBC
5.15E-54	-1.112288	0.141	0.813	1.24E-49	Tmem59	CBC_Ageddown	CBC
2.99E-49	-1.123623	0.958	1	7.18E-45	Ubb	CBC_Ageddown	CBC
4.74E-78	-1.151499	1	1	1.14E-73	Hspa8	CBC_Ageddown	CBC
7.91E-90	-1.254609	1	1	1.90E-85	Hsp90ab1	CBC_Ageddown	CBC
1.33E-36	-1.292079	0.531	0.885	3.20E-32	Id1	CBC_Ageddown	CBC
9.65E-61	-1.340272	0.698	0.993	2.32E-56	Sbpl	CBC_Ageddown	CBC
8.00E-66	-1.361455	0.885	0.993	1.92E-61	Dnaja1	CBC_Ageddown	CBC
1.08E-50	-1.369517	0.333	0.849	2.60E-46	Hspa1a	CBC_Ageddown	CBC
7.93E-13	-1.409885	0.458	0.679	1.90E-08	Jun	CBC_Ageddown	CBC
7.61E-69	-1.554447	0.786	0.984	1.82E-64	Hspa1b	CBC_Ageddown	CBC
3.90E-67	-1.64464	0.479	0.926	9.35E-63	Hsph1	CBC_Ageddown	CBC
3.74E-65	-1.649027	0.943	0.998	8.97E-61	Hspb1	CBC_Ageddown	CBC
7.53E-38	-1.772753	0.807	0.992	1.81E-33	Krt17	CBC_Ageddown	CBC
1.04E-88	-1.896949	0.974	1	2.50E-84	Hsp90aa1	CBC_Ageddown	CBC
2.07E-07	2.2731346	0.5	0	0.004972	Xist	EnC_Agedup	EnC
1.56E-08	2.1574231	0.929	0.578	0.000374	mt-Atp8	EnC_Agedup	EnC
8.69E-10	2.0484164	1	0.978	2.08E-05	Gm42418	EnC_Agedup	EnC
8.87E-12	1.7664131	1	1	2.13E-07	mt-Nd4l	EnC_Agedup	EnC
1.71E-06	1.6549553	0.893	0.578	0.040969	Tgm2	EnC_Agedup	EnC
1.55E-08	1.5996657	0.964	0.956	0.000373	H2-D1	EnC_Agedup	EnC
1.74E-07	1.5089127	1	0.889	0.004176	H2-K1	EnC_Agedup	EnC
1.71E-06	1.4061726	0.964	0.844	0.041027	B2m	EnC_Agedup	EnC
3.94E-10	1.2580617	1	1	9.44E-06	Rps29	EnC_Agedup	EnC
2.23E-07	1.0848308	1	1	0.005355	Rpl35	EnC_Agedup	EnC
3.84E-07	0.981005	1	1	0.009209	Rpl38	EnC_Agedup	EnC
1.04E-06	0.9393026	0.964	0.911	0.024853	Gm10076	EnC_Agedup	EnC
1.17E-06	0.9216758	1	1	0.027985	Rps27	EnC_Agedup	EnC

1.75E-07	-0.703591	1	1	0.004195	mt-Co3	EnC_Ageddown	EnC
1.12E-10	-0.771769	1	1	2.69E-06	mt-Atp6	EnC_Ageddown	EnC
8.75E-07	-0.800785	1	1	0.020979	Rps14	EnC_Ageddown	EnC
1.17E-06	-0.962579	1	1	0.027985	Rps8	EnC_Ageddown	EnC
2.23E-07	-0.995562	0.929	1	0.005354	Hspa8	EnC_Ageddown	EnC
1.51E-06	-1.23069	0.25	0.8	0.03625	Ssr4	EnC_Ageddown	EnC
2.86E-08	-1.235246	0.75	1	0.000685	H3f3a	EnC_Ageddown	EnC
1.28E-07	-1.503867	0.107	0.822	0.003081	Tmem59	EnC_Ageddown	EnC
4.77E-08	-1.673429	0.071	0.778	0.001143	Hsph1	EnC_Ageddown	EnC
5.14E-07	-1.694196	0.107	0.778	0.01233	Hspa1b	EnC_Ageddown	EnC
2.45E-12	-1.776674	0.821	1	5.87E-08	Hsp90ab1	EnC_Ageddown	EnC
1.21E-07	-1.827621	0.464	0.933	0.002897	Hsp90aa1	EnC_Ageddown	EnC
9.94E-45	2.7733373	0.798	0.575	2.38E-40	Crip1	EpC_Agedup	EpC
5.52E-144	2.4760001	0.704	0	1.32E-139	Xist	EpC_Agedup	EpC
3.42E-15	2.2882003	0.391	0.217	8.19E-11	Krt79	EpC_Agedup	EpC
5.78E-114	2.0255487	1	1	1.39E-109	Gm42418	EpC_Agedup	EpC
1.81E-130	1.8649464	0.983	0.802	4.33E-126	mt-Atp8	EpC_Agedup	EpC
1.21E-25	1.6943693	0.826	0.731	2.90E-21	S100a6	EpC_Agedup	EpC
5.30E-31	1.5328805	0.483	0.216	1.27E-26	Tppp3	EpC_Agedup	EpC
5.39E-104	1.3487155	0.987	0.968	1.29E-99	Gm10076	EpC_Agedup	EpC
6.56E-48	1.3253607	0.935	0.822	1.57E-43	H2-K1	EpC_Agedup	EpC
5.31E-34	1.3197135	0.4	0.12	1.27E-29	Pigr	EpC_Agedup	EpC
2.09E-17	1.3195323	0.62	0.427	5.01E-13	Ly6a	EpC_Agedup	EpC
2.64E-106	1.2871885	1	0.999	6.33E-102	mt-Nd4l	EpC_Agedup	EpC
8.21E-39	1.2647696	0.846	0.685	1.97E-34	Tmsb10	EpC_Agedup	EpC
2.33E-39	1.2309108	0.972	0.951	5.59E-35	Dbi	EpC_Agedup	EpC
8.23E-18	1.2227415	0.617	0.408	1.97E-13	Krt7	EpC_Agedup	EpC
3.63E-10	1.2102227	0.502	0.355	8.70E-06	Slpi	EpC_Agedup	EpC
8.60E-35	1.1883227	0.626	0.284	2.06E-30	Ifi27l2a	EpC_Agedup	EpC
3.18E-39	1.1148757	0.852	0.622	7.63E-35	B2m	EpC_Agedup	EpC
4.58E-10	1.083749	0.73	0.654	1.10E-05	Hopx	EpC_Agedup	EpC
2.70E-08	1.0702267	0.191	0.085	0.000648	Cxcl5	EpC_Agedup	EpC
9.17E-58	1.045315	0.359	0.013	2.20E-53	Tff2	EpC_Agedup	EpC
2.07E-72	1.0372874	0.996	0.997	4.97E-68	Rps28	EpC_Agedup	EpC
7.85E-81	1.005796	0.989	0.993	1.88E-76	Rpl38	EpC_Agedup	EpC
4.03E-18	1.0029962	0.637	0.443	9.67E-14	Plet1	EpC_Agedup	EpC
1.30E-17	0.9974629	0.837	0.689	3.11E-13	Tmem176b	EpC_Agedup	EpC
3.30E-07	0.9792469	0.47	0.36	0.007908	Krt19	EpC_Agedup	EpC
6.62E-51	0.9535257	0.974	0.986	1.59E-46	Rpl35	EpC_Agedup	EpC
4.14E-27	0.9515346	0.224	0.027	9.93E-23	Alox12e	EpC_Agedup	EpC
7.73E-30	0.9357441	0.926	0.896	1.85E-25	H2-D1	EpC_Agedup	EpC
1.48E-15	0.8742451	0.409	0.194	3.56E-11	Muc5b	EpC_Agedup	EpC
4.00E-45	0.8551721	0.383	0.055	9.59E-41	Gm26870	EpC_Agedup	EpC
1.54E-44	0.8415067	0.941	0.913	3.68E-40	Uba52	EpC_Agedup	EpC
6.01E-50	0.8339338	0.998	0.996	1.44E-45	Rpl37a	EpC_Agedup	EpC
1.25E-21	0.8302821	0.641	0.433	2.99E-17	Plb1	EpC_Agedup	EpC
1.10E-48	0.8051375	0.95	0.891	2.63E-44	Rpl27	EpC_Agedup	EpC
8.00E-50	0.7964092	0.998	0.996	1.92E-45	Rps29	EpC_Agedup	EpC
3.07E-37	0.7697566	0.824	0.605	7.37E-33	Snrpg	EpC_Agedup	EpC
5.33E-14	0.7624281	0.841	0.829	1.28E-09	Lgals3	EpC_Agedup	EpC
5.42E-33	0.7451901	0.215	0.009	1.30E-28	Gm10260	EpC_Agedup	EpC
4.13E-46	0.7397956	0.948	0.852	9.90E-42	Atp5k	EpC_Agedup	EpC
1.44E-17	0.7302657	0.787	0.666	3.46E-13	Cst3	EpC_Agedup	EpC
7.23E-44	0.7216105	0.846	0.687	1.73E-39	Ndufa3	EpC_Agedup	EpC
1.33E-26	0.7182917	0.389	0.127	3.19E-22	Stra6	EpC_Agedup	EpC
5.72E-19	0.7177975	0.913	0.857	1.37E-14	Crip2	EpC_Agedup	EpC
1.25E-12	0.7116603	0.802	0.725	3.00E-08	Hes1	EpC_Agedup	EpC
1.57E-42	0.7052351	0.998	1	3.75E-38	Rpl37	EpC_Agedup	EpC
1.10E-10	0.7028411	0.778	0.637	2.65E-06	Tmem176a	EpC_Agedup	EpC

5.91E-12	0.7028236	0.678	0.517	1.42E-07	Ccnd1	EpC_Agedup	EpC
6.93E-08	0.7014288	0.633	0.527	0.001662	Lgalsl	EpC_Agedup	EpC
5.07E-11	0.6951193	0.702	0.573	1.22E-06	Mfge8	EpC_Agedup	EpC
2.54E-14	0.6941961	0.67	0.505	6.09E-10	Elf3	EpC_Agedup	EpC
4.09E-32	0.6812148	0.998	0.999	9.82E-28	Rps27	EpC_Agedup	EpC
9.45E-37	0.6712393	0.996	0.997	2.27E-32	Rpl36	EpC_Agedup	EpC
1.79E-45	0.6611146	0.961	0.949	4.30E-41	Uqcr11	EpC_Agedup	EpC
3.62E-12	0.6602478	0.185	0.059	8.68E-08	Alox15	EpC_Agedup	EpC
5.83E-40	0.6537644	0.924	0.815	1.40E-35	Mrpl52	EpC_Agedup	EpC
2.83E-17	0.647559	0.493	0.26	6.78E-13	Nkd2	EpC_Agedup	EpC
1.86E-34	0.6412648	0.889	0.792	4.47E-30	Usmg5	EpC_Agedup	EpC
1.23E-32	0.6379679	0.757	0.486	2.95E-28	Erh	EpC_Agedup	EpC
4.01E-12	0.6374745	0.32	0.162	9.60E-08	Moxd1	EpC_Agedup	EpC
3.47E-13	0.6232459	0.622	0.449	8.33E-09	Cyba	EpC_Agedup	EpC
3.36E-16	0.6199854	0.515	0.291	8.07E-12	Tnfaip2	EpC_Agedup	EpC
2.99E-30	0.6161186	0.978	0.957	7.16E-26	Atpif1	EpC_Agedup	EpC
4.51E-15	0.6150518	0.42	0.221	1.08E-10	Gabrp	EpC_Agedup	EpC
3.39E-08	0.6115832	0.413	0.282	0.000814	Spns2	EpC_Agedup	EpC
3.42E-13	0.6115072	0.615	0.431	8.20E-09	S100a1	EpC_Agedup	EpC
1.35E-36	0.6104273	1	1	3.24E-32	Rpl41	EpC_Agedup	EpC
8.69E-35	0.6004241	0.889	0.757	2.08E-30	Romo1	EpC_Agedup	EpC
5.39E-25	0.5935725	0.996	0.997	1.29E-20	Rps20	EpC_Agedup	EpC
1.00E-33	0.5885302	0.93	0.926	2.41E-29	Tomm7	EpC_Agedup	EpC
5.54E-22	0.5873466	0.726	0.559	1.33E-17	Psme2	EpC_Agedup	EpC
1.34E-16	0.5830844	0.893	0.799	3.21E-12	Ptms	EpC_Agedup	EpC
2.46E-11	0.5763176	0.707	0.593	5.89E-07	Krt14	EpC_Agedup	EpC
1.14E-12	0.5729799	0.287	0.133	2.73E-08	1700016C15Rik	EpC_Agedup	EpC
1.80E-34	0.5722988	0.891	0.784	4.33E-30	Ndufa1	EpC_Agedup	EpC
1.15E-28	0.5721499	0.452	0.168	2.77E-24	Psmb8	EpC_Agedup	EpC
2.27E-27	0.5696204	0.752	0.53	5.45E-23	Pet100	EpC_Agedup	EpC
2.93E-07	0.5682108	0.17	0.072	0.007015	AA467197	EpC_Agedup	EpC
5.58E-42	0.566462	0.963	0.961	1.34E-37	Sec61g	EpC_Agedup	EpC
9.45E-20	0.5637226	0.707	0.522	2.27E-15	Psme1	EpC_Agedup	EpC
6.70E-19	0.5600659	0.304	0.104	1.61E-14	Cwh43	EpC_Agedup	EpC
3.57E-38	0.5592184	0.974	0.975	8.57E-34	Spint2	EpC_Agedup	EpC
2.52E-29	0.5585763	0.739	0.538	6.04E-25	Dnajc19	EpC_Agedup	EpC
8.77E-31	0.5580166	0.665	0.366	2.10E-26	Setd5	EpC_Agedup	EpC
1.38E-07	0.5552701	0.309	0.188	0.0033	Klf2	EpC_Agedup	EpC
2.28E-13	0.5506394	0.613	0.476	5.46E-09	Furin	EpC_Agedup	EpC
2.64E-17	0.5479104	0.604	0.401	6.33E-13	Prr15l	EpC_Agedup	EpC
7.88E-12	0.5470519	0.67	0.546	1.89E-07	Acadm	EpC_Agedup	EpC
3.80E-12	0.5460797	0.263	0.114	9.11E-08	F5	EpC_Agedup	EpC
1.20E-17	0.5459918	0.993	0.999	2.87E-13	Rps19	EpC_Agedup	EpC
3.08E-17	0.5432977	0.567	0.349	7.39E-13	Gm47283	EpC_Agedup	EpC
1.33E-23	0.535126	0.88	0.847	3.18E-19	Ndufc2	EpC_Agedup	EpC
6.84E-41	0.5327943	0.396	0.078	1.64E-36	Samd9l	EpC_Agedup	EpC
1.96E-23	0.5325317	0.702	0.945	4.69E-19	Bpifb1	EpC_Agedup	EpC
1.07E-29	0.5319538	0.883	0.851	2.56E-25	Ost4	EpC_Agedup	EpC
4.72E-31	0.5296664	0.978	0.977	1.13E-26	Atp5l	EpC_Agedup	EpC
3.79E-12	0.5279983	0.672	0.504	9.10E-08	Mgst1	EpC_Agedup	EpC
3.62E-27	0.527789	0.965	0.97	8.68E-23	Cox6b1	EpC_Agedup	EpC
1.41E-16	0.5239836	0.452	0.237	3.39E-12	Dhcr24	EpC_Agedup	EpC
6.16E-09	0.523969	0.689	0.537	0.000148	Cldn3	EpC_Agedup	EpC
3.98E-11	0.5233563	0.38	0.226	9.54E-07	Nt5e	EpC_Agedup	EpC
4.38E-26	0.5216717	0.707	0.466	1.05E-21	1810022K09Rik	EpC_Agedup	EpC
4.07E-19	0.5208232	0.55	0.314	9.75E-15	Flna	EpC_Agedup	EpC
1.52E-09	0.5194592	0.865	0.86	3.64E-05	Lmna	EpC_Agedup	EpC
4.87E-16	0.5158686	0.928	0.74	1.17E-11	Ly6e	EpC_Agedup	EpC
8.11E-26	0.5156124	0.865	0.754	1.95E-21	Mrpl33	EpC_Agedup	EpC

8.73E-12	0.5149401	0.554	0.375	2.09E-07	Sorbs2	EpC_Agedup	EpC
1.67E-28	0.5139902	0.98	0.986	4.01E-24	Cox6c	EpC_Agedup	EpC
1.63E-08	0.513217	0.591	0.818	0.00039	Krtdap	EpC_Agedup	EpC
5.86E-11	0.5108735	0.843	0.829	1.40E-06	Tagln2	EpC_Agedup	EpC
4.66E-24	0.5053109	0.911	0.854	1.12E-19	2010107E04Rik	EpC_Agedup	EpC
8.45E-19	0.5043175	0.246	0.068	2.03E-14	1600014C10Rik	EpC_Agedup	EpC
5.40E-31	0.5004232	0.965	0.975	1.29E-26	Atp5e	EpC_Agedup	EpC
1.21E-06	0.4941344	0.704	0.602	0.028947	Tfcp2l1	EpC_Agedup	EpC
5.74E-21	0.4939684	0.787	0.622	1.38E-16	Bola2	EpC_Agedup	EpC
5.63E-13	0.4913542	0.663	0.493	1.35E-08	Ece1	EpC_Agedup	EpC
6.05E-13	0.4889129	0.598	0.42	1.45E-08	Scd2	EpC_Agedup	EpC
1.52E-12	0.487478	0.624	0.452	3.65E-08	Lars2	EpC_Agedup	EpC
1.16E-08	0.4873916	0.767	0.687	0.000279	Tsc22d1	EpC_Agedup	EpC
1.80E-09	0.4865195	0.887	0.848	4.32E-05	Cstb	EpC_Agedup	EpC
1.17E-30	0.4851557	0.972	0.974	2.80E-26	Elob	EpC_Agedup	EpC
5.60E-10	0.4824939	0.25	0.114	1.34E-05	Prss22	EpC_Agedup	EpC
6.66E-30	0.4823644	0.248	0.029	1.60E-25	H2-Q7	EpC_Agedup	EpC
2.92E-28	0.4812133	0.92	0.925	7.01E-24	Rpl36al	EpC_Agedup	EpC
2.20E-13	0.4811346	0.917	0.907	5.27E-09	Ctsd	EpC_Agedup	EpC
2.64E-19	0.4809591	0.424	0.195	6.34E-15	Meis2	EpC_Agedup	EpC
3.00E-12	0.4776608	0.228	0.087	7.19E-08	Arc	EpC_Agedup	EpC
4.88E-09	0.4749183	0.702	0.599	0.000117	Cat	EpC_Agedup	EpC
1.70E-15	0.4747176	0.672	0.47	4.08E-11	Barx2	EpC_Agedup	EpC
6.72E-20	0.4725747	0.648	0.436	1.61E-15	Smim22	EpC_Agedup	EpC
7.11E-25	0.4721772	0.939	0.941	1.71E-20	Rps27l	EpC_Agedup	EpC
7.31E-19	0.4703066	0.713	0.562	1.75E-14	Sap18	EpC_Agedup	EpC
1.06E-18	0.4689009	0.841	0.77	2.53E-14	Ndufb2	EpC_Agedup	EpC
5.62E-11	0.4672602	0.567	0.395	1.35E-06	Anxa3	EpC_Agedup	EpC
5.24E-19	0.4671227	0.793	0.651	1.26E-14	Cycs	EpC_Agedup	EpC
2.08E-25	0.4659402	0.509	0.232	4.98E-21	Gstp1	EpC_Agedup	EpC
2.25E-17	0.4582561	0.991	0.997	5.40E-13	Rpl30	EpC_Agedup	EpC
1.79E-37	0.450675	0.346	0.059	4.28E-33	Csprs	EpC_Agedup	EpC
9.40E-13	0.4478817	0.178	0.052	2.25E-08	Ttc36	EpC_Agedup	EpC
2.64E-17	0.4475694	0.628	0.438	6.32E-13	Cox17	EpC_Agedup	EpC
2.81E-16	0.4443893	0.228	0.064	6.73E-12	Bcat1	EpC_Agedup	EpC
3.22E-16	0.4429428	0.904	0.792	7.73E-12	Nedd4	EpC_Agedup	EpC
9.77E-11	0.4425497	0.204	0.08	2.34E-06	Wfdc15b	EpC_Agedup	EpC
3.66E-17	0.4423293	0.733	0.596	8.78E-13	Dpm3	EpC_Agedup	EpC
1.52E-11	0.4417653	0.959	0.965	3.65E-07	Rpl36a	EpC_Agedup	EpC
1.03E-14	0.4382731	0.641	0.472	2.48E-10	Cdc42ep5	EpC_Agedup	EpC
6.15E-17	0.4376245	0.791	0.711	1.47E-12	Tmem258	EpC_Agedup	EpC
6.43E-14	0.437416	0.602	0.404	1.54E-09	Mmp15	EpC_Agedup	EpC
9.45E-09	0.4353412	0.272	0.146	0.000227	Pdxk	EpC_Agedup	EpC
1.04E-09	0.4340201	0.352	0.208	2.49E-05	Plk2	EpC_Agedup	EpC
1.77E-14	0.4326489	0.383	0.187	4.24E-10	Uaca	EpC_Agedup	EpC
1.27E-20	0.4290444	0.937	0.933	3.05E-16	Uqcr10	EpC_Agedup	EpC
8.44E-11	0.4263246	0.993	0.996	2.02E-06	Rpl39	EpC_Agedup	EpC
1.62E-21	0.4251552	0.972	0.987	3.88E-17	Cox7c	EpC_Agedup	EpC
5.79E-10	0.4238841	0.989	0.991	1.39E-05	Rps2	EpC_Agedup	EpC
1.37E-19	0.4228162	0.876	0.832	3.28E-15	Ndufb7	EpC_Agedup	EpC
2.73E-18	0.4164987	0.839	0.702	6.54E-14	Ndufa5	EpC_Agedup	EpC
2.18E-11	0.4157313	0.637	0.489	5.22E-07	Fam32a	EpC_Agedup	EpC
3.75E-26	0.414816	0.483	0.201	8.99E-22	Grcc10	EpC_Agedup	EpC
2.03E-07	0.4139891	0.754	0.669	0.004865	Txnip	EpC_Agedup	EpC
5.12E-13	0.4139633	0.541	0.343	1.23E-08	Lasp1	EpC_Agedup	EpC
2.39E-08	0.4136133	0.411	0.265	0.000574	Cdc42ep3	EpC_Agedup	EpC
5.25E-21	0.4135101	0.896	0.845	1.26E-16	Ndufa11	EpC_Agedup	EpC
4.88E-22	0.4114681	0.978	0.987	1.17E-17	Oaz1	EpC_Agedup	EpC
8.34E-14	0.4082523	0.789	0.666	2.00E-09	Ifi27	EpC_Agedup	EpC

6.55E-13	0.4075712	0.833	0.748	1.57E-08	Cdh1	EpC_Agedup	EpC
1.14E-07	0.4059704	0.767	0.728	0.002723	Sec11c	EpC_Agedup	EpC
5.06E-11	0.4046777	0.45	0.284	1.21E-06	S100a13	EpC_Agedup	EpC
2.68E-18	0.404156	0.833	0.716	6.42E-14	Ndufv3	EpC_Agedup	EpC
4.62E-16	0.4011266	0.47	0.26	1.11E-11	Npc1	EpC_Agedup	EpC
2.37E-13	0.4000307	0.613	0.446	5.69E-09	Chchd7	EpC_Agedup	EpC
1.58E-08	0.3998472	0.454	0.331	0.000379	Ermp1	EpC_Agedup	EpC
1.18E-14	0.3972239	0.78	0.667	2.83E-10	Tmem256	EpC_Agedup	EpC
9.17E-19	0.3966517	0.639	0.421	2.20E-14	Hypk	EpC_Agedup	EpC
3.39E-22	0.3939275	0.22	0.039	8.13E-18	Dpysl2	EpC_Agedup	EpC
5.26E-11	0.3902847	0.822	0.683	1.26E-06	Ctsb	EpC_Agedup	EpC
1.02E-09	0.3892799	0.385	0.236	2.45E-05	Inhbb	EpC_Agedup	EpC
6.07E-10	0.3889642	0.998	0.996	1.46E-05	mt-Nd5	EpC_Agedup	EpC
4.62E-11	0.387585	0.25	0.101	1.11E-06	Akr1c18	EpC_Agedup	EpC
3.04E-15	0.3875087	0.891	0.822	7.30E-11	Son	EpC_Agedup	EpC
1.06E-14	0.3873227	0.93	0.932	2.55E-10	Uqcrq	EpC_Agedup	EpC
3.00E-25	0.3857012	0.233	0.035	7.18E-21	Osr2	EpC_Agedup	EpC
3.94E-08	0.3842302	0.6	0.447	0.000945	Kctd14	EpC_Agedup	EpC
3.09E-08	0.3831527	0.95	0.961	0.00074	Txn1	EpC_Agedup	EpC
6.46E-13	0.3829437	0.5	0.307	1.55E-08	Acsl3	EpC_Agedup	EpC
2.36E-13	0.3824888	0.52	0.333	5.67E-09	Slc39a1	EpC_Agedup	EpC
5.06E-16	0.3823537	0.83	0.755	1.21E-11	Mrps21	EpC_Agedup	EpC
3.14E-12	0.3821209	0.891	0.858	7.52E-08	Atp5g3	EpC_Agedup	EpC
1.05E-10	0.3807676	0.88	0.822	2.52E-06	Iqgap1	EpC_Agedup	EpC
2.70E-08	0.3766464	0.378	0.246	0.000648	Flrt2	EpC_Agedup	EpC
1.06E-06	0.3760587	0.68	0.603	0.025507	Baiap2	EpC_Agedup	EpC
2.23E-17	0.375055	0.896	0.874	5.35E-13	Ndufa2	EpC_Agedup	EpC
3.86E-18	0.3737673	0.57	0.331	9.26E-14	Gm11808	EpC_Agedup	EpC
1.40E-07	0.3736457	0.317	0.187	0.003355	Sfrp1	EpC_Agedup	EpC
4.07E-16	0.3725745	0.276	0.096	9.76E-12	Ntng1	EpC_Agedup	EpC
5.31E-16	0.3721431	0.87	0.832	1.27E-11	Ndufb8	EpC_Agedup	EpC
4.75E-10	0.3701683	0.452	0.289	1.14E-05	Vat1	EpC_Agedup	EpC
2.46E-14	0.368765	0.343	0.158	5.90E-10	Ptprz1	EpC_Agedup	EpC
1.62E-15	0.3677013	0.97	0.965	3.88E-11	Cox7a2	EpC_Agedup	EpC
1.45E-08	0.3646403	0.593	0.449	0.000347	Srebf2	EpC_Agedup	EpC
1.03E-27	0.3644799	0.235	0.029	2.46E-23	Ifi203	EpC_Agedup	EpC
4.05E-18	0.3638201	0.917	0.919	9.71E-14	Ndufa7	EpC_Agedup	EpC
9.59E-11	0.3629058	0.989	0.991	2.30E-06	Malat1	EpC_Agedup	EpC
6.77E-19	0.3622737	0.926	0.909	1.62E-14	Ndufa13	EpC_Agedup	EpC
6.76E-11	0.3607069	0.607	0.459	1.62E-06	Tuba1b	EpC_Agedup	EpC
2.47E-08	0.359936	0.517	0.357	0.000593	Cyp51	EpC_Agedup	EpC
1.92E-06	0.357536	0.459	0.349	0.046056	Pdlim4	EpC_Agedup	EpC
1.11E-08	0.3572986	0.42	0.278	0.000266	Samhd1	EpC_Agedup	EpC
5.26E-10	0.3561268	0.704	0.605	1.26E-05	Ndufb6	EpC_Agedup	EpC
4.64E-12	0.3554663	0.907	0.868	1.11E-07	Snrpe	EpC_Agedup	EpC
1.25E-15	0.3554379	0.365	0.164	2.99E-11	Fam71a	EpC_Agedup	EpC
6.13E-07	0.3497333	0.789	0.719	0.014695	Ddx3x	EpC_Agedup	EpC
9.14E-11	0.3481817	0.789	0.744	2.19E-06	Fkbp2	EpC_Agedup	EpC
1.99E-16	0.3476247	0.554	0.342	4.77E-12	Cmc1	EpC_Agedup	EpC
1.21E-06	0.3462234	0.459	0.337	0.028998	Rhoc	EpC_Agedup	EpC
4.57E-13	0.3453606	0.724	0.579	1.09E-08	Chchd1	EpC_Agedup	EpC
1.22E-12	0.3429271	0.733	0.564	2.92E-08	Golgb1	EpC_Agedup	EpC
1.13E-12	0.340574	0.88	0.847	2.70E-08	Ndufa6	EpC_Agedup	EpC
4.52E-07	0.3391273	0.72	0.663	0.010831	H2-T23	EpC_Agedup	EpC
1.74E-14	0.3371536	0.93	0.935	4.17E-10	Edf1	EpC_Agedup	EpC
5.40E-11	0.3357046	0.709	0.59	1.29E-06	Churc1	EpC_Agedup	EpC
4.41E-07	0.3356511	0.322	0.207	0.010584	Plbd1	EpC_Agedup	EpC
4.99E-12	0.3351433	0.628	0.443	1.20E-07	Lsm7	EpC_Agedup	EpC
6.57E-12	0.3318401	0.854	0.779	1.57E-07	Arpp19	EpC_Agedup	EpC

1.22E-07	0.3309227	0.711	0.583	0.00293	Clec2d	EpC_Agedup	EpC
5.33E-09	0.330446	0.637	0.488	0.000128	C77080	EpC_Agedup	EpC
1.32E-08	0.3301114	0.441	0.285	0.000317	Cib2	EpC_Agedup	EpC
1.06E-12	0.3274247	0.613	0.433	2.54E-08	Mrps28	EpC_Agedup	EpC
1.02E-08	0.3240959	0.772	0.702	0.000244	Nenf	EpC_Agedup	EpC
1.04E-06	0.3231909	0.635	0.514	0.024865	Celsr2	EpC_Agedup	EpC
7.79E-09	0.322145	0.496	0.339	0.000187	Abhd2	EpC_Agedup	EpC
2.37E-08	0.3190017	0.287	0.162	0.000567	Sox7	EpC_Agedup	EpC
1.54E-06	0.3177238	0.985	0.996	0.036987	Rps7	EpC_Agedup	EpC
1.84E-10	0.3176708	0.998	1	4.41E-06	Rpl18a	EpC_Agedup	EpC
6.21E-07	0.3165103	0.88	0.864	0.014883	Cox6a1	EpC_Agedup	EpC
1.02E-08	0.3158128	0.628	0.492	0.000244	Pin4	EpC_Agedup	EpC
9.39E-10	0.3155684	0.443	0.288	2.25E-05	Cdc42bpb	EpC_Agedup	EpC
5.69E-11	0.3153213	0.928	0.928	1.36E-06	Atp6v1g1	EpC_Agedup	EpC
1.87E-19	0.3152871	0.276	0.078	4.49E-15	Runx3	EpC_Agedup	EpC
7.54E-09	0.3151787	0.974	0.981	0.000181	Ndufa4	EpC_Agedup	EpC
4.94E-07	0.3150071	0.524	0.385	0.011851	Tm4sf1	EpC_Agedup	EpC
2.58E-10	0.3132913	0.989	0.997	6.19E-06	Rpl6	EpC_Agedup	EpC
4.62E-07	0.312588	0.457	0.339	0.011067	Tmem43	EpC_Agedup	EpC
7.13E-09	0.3124551	0.741	0.66	0.000171	Timm8b	EpC_Agedup	EpC
1.82E-10	0.3114691	0.217	0.09	4.37E-06	Cd55	EpC_Agedup	EpC
2.12E-16	0.3108763	0.957	0.981	5.09E-12	Atp5j2	EpC_Agedup	EpC
1.22E-09	0.3107815	0.633	0.498	2.92E-05	Pam16	EpC_Agedup	EpC
9.54E-07	0.3103758	0.667	0.537	0.022883	Diaph1	EpC_Agedup	EpC
6.35E-10	0.3102532	0.913	0.919	1.52E-05	Atp5g1	EpC_Agedup	EpC
4.55E-12	0.3094204	0.454	0.268	1.09E-07	Gpcpd1	EpC_Agedup	EpC
7.41E-11	0.3062306	0.948	0.964	1.78E-06	Atp5g2	EpC_Agedup	EpC
1.47E-07	0.3049084	0.498	0.357	0.003519	Notch2	EpC_Agedup	EpC
2.59E-07	0.3048109	0.726	0.656	0.006201	Mrpl23	EpC_Agedup	EpC
7.00E-15	0.3038814	0.265	0.094	1.68E-10	H2-Q4	EpC_Agedup	EpC
4.03E-07	0.30384	0.385	0.25	0.009656	Arrdc4	EpC_Agedup	EpC
5.21E-08	0.3036254	0.413	0.274	0.001249	Fdft1	EpC_Agedup	EpC
3.62E-18	0.3027661	0.337	0.127	8.69E-14	Snhg9	EpC_Agedup	EpC
1.11E-09	0.3024441	0.887	0.847	2.65E-05	D8Ertd738e	EpC_Agedup	EpC
2.31E-10	0.29969	0.898	0.855	5.54E-06	Scand1	EpC_Agedup	EpC
8.91E-12	0.2990874	0.813	0.724	2.14E-07	Cox14	EpC_Agedup	EpC
2.24E-09	0.2974126	0.463	0.301	5.37E-05	Anpep	EpC_Agedup	EpC
6.78E-08	0.2967048	0.496	0.368	0.001625	Ociad2	EpC_Agedup	EpC
2.50E-12	0.2960352	0.228	0.085	5.99E-08	Lbp	EpC_Agedup	EpC
4.47E-11	0.2948805	0.815	0.705	1.07E-06	Rex1bd	EpC_Agedup	EpC
2.09E-09	0.2942623	0.472	0.311	5.01E-05	Ncaph2	EpC_Agedup	EpC
1.84E-12	0.2939818	0.452	0.265	4.41E-08	Polr2l	EpC_Agedup	EpC
8.38E-07	0.2938768	0.793	0.768	0.020106	Hsbp1	EpC_Agedup	EpC
6.25E-08	0.2935672	0.502	0.357	0.001499	Syne2	EpC_Agedup	EpC
3.74E-11	0.2927918	0.976	0.974	8.96E-07	Atp5j	EpC_Agedup	EpC
5.98E-07	0.2927855	0.711	0.622	0.014332	Rpn1	EpC_Agedup	EpC
7.17E-07	0.2925961	0.454	0.331	0.017204	Dhcr7	EpC_Agedup	EpC
3.26E-10	0.2921993	0.437	0.268	7.81E-06	Irx2	EpC_Agedup	EpC
5.26E-07	0.2912505	0.924	0.938	0.012605	Cox5a	EpC_Agedup	EpC
1.01E-08	0.2908015	0.926	0.954	0.000242	Sec61b	EpC_Agedup	EpC
1.34E-18	0.2898604	0.98	0.994	3.22E-14	Serf2	EpC_Agedup	EpC
1.21E-09	0.2872053	0.846	0.812	2.90E-05	Ndufab1	EpC_Agedup	EpC
3.94E-07	0.2870326	0.867	0.861	0.009451	Ndufc1	EpC_Agedup	EpC
1.95E-11	0.2863129	0.52	0.336	4.68E-07	Gsr	EpC_Agedup	EpC
4.91E-09	0.286199	0.526	0.359	0.000118	Vars	EpC_Agedup	EpC
3.46E-08	0.2855884	0.715	0.614	0.000831	Naa38	EpC_Agedup	EpC
1.93E-08	0.2846099	0.985	0.996	0.000463	Rpl11	EpC_Agedup	EpC
3.23E-07	0.2843849	0.904	0.903	0.007744	Tmbim6	EpC_Agedup	EpC
1.57E-07	0.2833725	0.554	0.41	0.003764	Prr13	EpC_Agedup	EpC

3.15E-07	0.2813792	0.243	0.132	0.007551	Egr2	EpC_Agedup	EpC
5.65E-07	0.2805307	0.72	0.63	0.013549	Ppdpf	EpC_Agedup	EpC
2.79E-08	0.280361	0.452	0.308	0.000669	Prkab1	EpC_Agedup	EpC
1.69E-08	0.280058	0.504	0.347	0.000405	Myof	EpC_Agedup	EpC
5.63E-07	0.2778691	0.993	0.996	0.013505	Rps23	EpC_Agedup	EpC
1.58E-06	0.2773505	0.633	0.52	0.037972	Cib1	EpC_Agedup	EpC
1.75E-07	0.2771272	0.8	0.744	0.004193	Cd63	EpC_Agedup	EpC
1.99E-08	0.2770007	0.502	0.355	0.000476	Ap2b1	EpC_Agedup	EpC
5.42E-11	0.2768813	0.372	0.205	1.30E-06	Apobec1	EpC_Agedup	EpC
1.85E-14	0.2765601	0.237	0.078	4.42E-10	Adgre5	EpC_Agedup	EpC
5.78E-07	0.2758603	0.696	0.627	0.013867	Eny2	EpC_Agedup	EpC
9.73E-09	0.2739405	0.752	0.648	0.000233	Cuta	EpC_Agedup	EpC
7.99E-11	0.2736788	0.374	0.201	1.92E-06	Vamp5	EpC_Agedup	EpC
6.24E-08	0.2734992	0.115	0.035	0.001497	Gprc5a	EpC_Agedup	EpC
4.99E-08	0.2734304	0.835	0.806	0.001197	Ndufb3	EpC_Agedup	EpC
1.43E-08	0.2730275	0.409	0.259	0.000344	Ldlr	EpC_Agedup	EpC
3.70E-08	0.2726933	0.97	0.951	0.000886	Sem1	EpC_Agedup	EpC
1.88E-08	0.2725035	0.6	0.453	0.000452	Rab15	EpC_Agedup	EpC
1.92E-09	0.2698049	0.357	0.201	4.60E-05	Ano1	EpC_Agedup	EpC
9.78E-07	0.2688324	0.522	0.392	0.023459	Sft2d2	EpC_Agedup	EpC
8.75E-07	0.268694	0.95	0.939	0.020979	Eif4g2	EpC_Agedup	EpC
9.62E-07	0.2680587	0.896	0.894	0.023075	Vamp8	EpC_Agedup	EpC
8.84E-07	0.2670039	0.843	0.771	0.021201	Morf4l1	EpC_Agedup	EpC
1.05E-07	0.2664716	0.998	1	0.002509	Eif1	EpC_Agedup	EpC
5.05E-12	0.2650272	0.261	0.11	1.21E-07	Il1r1	EpC_Agedup	EpC
4.77E-07	0.262085	0.776	0.68	0.011442	Eif4g1	EpC_Agedup	EpC
2.62E-14	0.261537	0.25	0.081	6.29E-10	Bbox1	EpC_Agedup	EpC
1.51E-06	0.2611861	0.576	0.452	0.036097	Urah	EpC_Agedup	EpC
9.38E-07	0.2596128	0.383	0.252	0.022499	Sqle	EpC_Agedup	EpC
1.93E-07	0.2595404	0.583	0.437	0.00462	Ctdsp2	EpC_Agedup	EpC
8.79E-07	0.2587711	0.454	0.331	0.021087	Fam102a	EpC_Agedup	EpC
1.25E-25	0.2583883	0.187	0.013	3.00E-21	H2-Q6	EpC_Agedup	EpC
7.45E-07	0.2582387	0.554	0.418	0.01787	Ndufaf8	EpC_Agedup	EpC
3.27E-15	0.2566117	0.35	0.151	7.85E-11	Tomm6	EpC_Agedup	EpC
2.69E-07	0.2560983	0.713	0.609	0.006447	Pdcd5	EpC_Agedup	EpC
4.55E-12	0.2547539	0.317	0.149	1.09E-07	Acp5	EpC_Agedup	EpC
3.67E-07	0.2543965	0.989	0.997	0.008799	Rpl34	EpC_Agedup	EpC
3.49E-07	0.2527724	0.817	0.783	0.008369	Nop10	EpC_Agedup	EpC
1.35E-13	0.2527181	0.322	0.142	3.23E-09	Bloc1s1	EpC_Agedup	EpC
9.01E-14	0.2522401	0.293	0.119	2.16E-09	Gm8186	EpC_Agedup	EpC
9.00E-14	0.2511791	0.133	0.022	2.16E-09	C2cd4b	EpC_Agedup	EpC
4.23E-07	0.2506281	0.72	0.644	0.010136	Trmt112	EpC_Agedup	EpC
5.00E-07	0.2496152	0.852	0.854	0.012	Ndufs5	EpC_Agedup	EpC
1.78E-10	0.2495212	0.307	0.155	4.26E-06	Sh3bp4	EpC_Agedup	EpC
7.69E-14	0.2489664	0.311	0.133	1.84E-09	Vcpkmt	EpC_Agedup	EpC
9.14E-09	0.2470001	0.389	0.229	0.000219	Serpinb6b	EpC_Agedup	EpC
2.25E-07	0.2465627	0.674	0.579	0.005396	Polr2k	EpC_Agedup	EpC
1.27E-08	0.2463012	0.3	0.168	0.000305	Lyn	EpC_Agedup	EpC
1.07E-07	0.2457097	0.578	0.433	0.002575	Slirp	EpC_Agedup	EpC
2.07E-07	0.2450924	0.354	0.219	0.004972	Rflnb	EpC_Agedup	EpC
9.25E-07	0.2445763	0.522	0.398	0.022181	Tmem160	EpC_Agedup	EpC
9.24E-08	0.2445579	0.93	0.958	0.002217	Cox5b	EpC_Agedup	EpC
3.29E-08	0.2437349	0.296	0.162	0.000788	Ifngr1	EpC_Agedup	EpC
2.43E-09	0.2420789	0.396	0.237	5.83E-05	Tpcn1	EpC_Agedup	EpC
4.61E-07	0.2419312	0.398	0.265	0.011054	Srebf1	EpC_Agedup	EpC
5.75E-07	0.2415187	0.474	0.342	0.013779	Erbb2	EpC_Agedup	EpC
6.58E-07	0.2399411	0.322	0.201	0.015779	Acaca	EpC_Agedup	EpC
3.20E-09	0.2396824	0.407	0.239	7.67E-05	Psmg4	EpC_Agedup	EpC
8.22E-11	0.2375432	0.222	0.082	1.97E-06	Lrmp	EpC_Agedup	EpC

7.61E-13	0.2367173	0.333	0.151	1.82E-08	B4galt3	EpC_Agedup	EpC
4.87E-08	0.2366954	0.4	0.253	0.001169	Lurap1l	EpC_Agedup	EpC
2.85E-07	0.2358253	0.415	0.271	0.006834	Sash1	EpC_Agedup	EpC
8.56E-17	0.2352277	0.17	0.03	2.05E-12	E2f2	EpC_Agedup	EpC
8.70E-11	0.2347997	0.35	0.182	2.09E-06	Dvl1	EpC_Agedup	EpC
2.53E-10	0.2347686	0.335	0.179	6.07E-06	Ppp6r1	EpC_Agedup	EpC
2.91E-07	0.2346655	0.659	0.524	0.00697	Ewsr1	EpC_Agedup	EpC
1.11E-08	0.2343966	0.309	0.168	0.000266	Naga	EpC_Agedup	EpC
2.47E-10	0.2324933	0.239	0.106	5.93E-06	Tex15	EpC_Agedup	EpC
2.30E-17	0.2324646	0.1	0	5.51E-13	Gfra2	EpC_Agedup	EpC
9.47E-10	0.2312384	0.974	0.984	2.27E-05	Atp5h	EpC_Agedup	EpC
8.81E-08	0.2308956	0.498	0.346	0.002113	Mrpl41	EpC_Agedup	EpC
4.92E-08	0.2308412	0.226	0.109	0.00118	Bhlhe41	EpC_Agedup	EpC
2.51E-09	0.2299395	0.991	0.999	6.01E-05	Chchd2	EpC_Agedup	EpC
2.10E-07	0.2288366	0.954	0.959	0.005043	Ubl5	EpC_Agedup	EpC
6.92E-08	0.2287108	0.378	0.237	0.001659	Dynlt1f	EpC_Agedup	EpC
1.06E-07	0.2286339	0.824	0.767	0.002532	Taf10	EpC_Agedup	EpC
2.51E-09	0.2283426	0.287	0.151	6.02E-05	Capsl	EpC_Agedup	EpC
4.39E-11	0.2272355	0.341	0.175	1.05E-06	0610010K14Rik	EpC_Agedup	EpC
4.00E-07	0.226551	0.387	0.249	0.009583	Tjp3	EpC_Agedup	EpC
3.06E-12	0.2255755	0.241	0.093	7.33E-08	Psmb9	EpC_Agedup	EpC
2.93E-13	0.2252446	0.333	0.148	7.03E-09	Tap2	EpC_Agedup	EpC
5.22E-21	0.2251379	0.23	0.046	1.25E-16	Gm8797	EpC_Agedup	EpC
1.53E-06	0.2250101	0.472	0.342	0.036716	Ddx54	EpC_Agedup	EpC
3.22E-07	0.2249392	0.313	0.188	0.007722	2010300C02Rik	EpC_Agedup	EpC
6.07E-15	0.224482	0.204	0.056	1.46E-10	Dtx4	EpC_Agedup	EpC
3.70E-07	0.2235993	0.252	0.139	0.008882	Kcnk5	EpC_Agedup	EpC
9.37E-07	0.2229669	0.378	0.249	0.022458	Tmem63a	EpC_Agedup	EpC
6.18E-08	0.2224017	0.341	0.201	0.001482	Lzts2	EpC_Agedup	EpC
1.19E-06	0.2220407	0.391	0.259	0.028599	Rhoq	EpC_Agedup	EpC
1.07E-06	0.2218726	0.802	0.74	0.025774	Sec62	EpC_Agedup	EpC
2.72E-07	0.2213904	0.493	0.344	0.006526	Trp53bp2	EpC_Agedup	EpC
3.62E-09	0.2209625	0.326	0.178	8.68E-05	Slc28a3	EpC_Agedup	EpC
2.83E-09	0.2200134	0.2	0.084	6.78E-05	B3gnt8	EpC_Agedup	EpC
7.02E-07	0.2196943	0.989	0.996	0.016835	Myl6	EpC_Agedup	EpC
1.74E-12	0.2195003	0.25	0.097	4.18E-08	Fhl2	EpC_Agedup	EpC
1.86E-07	0.2189582	0.948	0.971	0.004451	Uqcrb	EpC_Agedup	EpC
5.13E-07	0.2184212	0.893	0.889	0.012313	Lamtor2	EpC_Agedup	EpC
1.27E-17	0.2181352	0.267	0.081	3.05E-13	Adat2	EpC_Agedup	EpC
2.26E-16	0.217802	0.246	0.074	5.41E-12	Gm10036	EpC_Agedup	EpC
1.55E-07	0.217527	0.193	0.088	0.003729	Apol7a	EpC_Agedup	EpC
1.31E-07	0.217444	0.315	0.187	0.003151	Nacc1	EpC_Agedup	EpC
6.12E-07	0.2170812	0.337	0.21	0.014688	Qtrt1	EpC_Agedup	EpC
3.37E-16	0.216763	0.252	0.081	8.09E-12	Ocel1	EpC_Agedup	EpC
4.38E-07	0.2131548	0.641	0.546	0.010503	Jtb	EpC_Agedup	EpC
2.91E-07	0.2131174	0.267	0.149	0.006971	Ptprj	EpC_Agedup	EpC
4.94E-12	0.2119554	0.154	0.039	1.18E-07	Erich3	EpC_Agedup	EpC
5.26E-08	0.2105385	0.326	0.185	0.00126	Bst2	EpC_Agedup	EpC
1.92E-07	0.2077895	0.898	0.906	0.004612	Ndufb11	EpC_Agedup	EpC
4.16E-13	0.2062014	0.296	0.123	9.97E-09	Cox16	EpC_Agedup	EpC
4.11E-09	0.2060642	0.317	0.171	9.85E-05	Plxna1	EpC_Agedup	EpC
3.94E-09	0.2060027	0.317	0.172	9.45E-05	Rnf141	EpC_Agedup	EpC
1.06E-06	0.2042827	0.315	0.194	0.025338	Slc6a9	EpC_Agedup	EpC
1.24E-06	0.2031608	0.848	0.848	0.029805	Srp9	EpC_Agedup	EpC
1.68E-06	0.2028581	0.52	0.385	0.040387	Mrps36	EpC_Agedup	EpC
1.68E-07	0.202276	0.346	0.211	0.004036	Mansc1	EpC_Agedup	EpC
4.18E-16	0.2009883	0.165	0.03	1.00E-11	Trbc2	EpC_Agedup	EpC
1.32E-08	0.2008611	0.391	0.236	0.000316	2300009A05Rik	EpC_Agedup	EpC
1.08E-06	0.2008382	0.53	0.394	0.026007	Dnajc15	EpC_Agedup	EpC

6.67E-09	0.2005421	0.343	0.191	0.00016	Txndc12	EpC_Agedup	EpC
1.88E-06	0.200256	0.209	0.107	0.044992	Ptpru	EpC_Agedup	EpC
5.39E-09	-0.200814	0.172	0.321	0.000129	Hspa13	EpC_Ageddown	EpC
1.60E-06	-0.201515	0.304	0.428	0.038338	Ppp6c	EpC_Ageddown	EpC
1.63E-07	-0.202186	0.25	0.389	0.003918	Lamtor3	EpC_Ageddown	EpC
1.61E-09	-0.202935	0.072	0.2	3.87E-05	Best2	EpC_Ageddown	EpC
1.91E-08	-0.203214	0.065	0.178	0.000458	Cyp2b10	EpC_Ageddown	EpC
6.54E-07	-0.203245	0.117	0.227	0.015672	Dapp1	EpC_Ageddown	EpC
1.02E-10	-0.204921	0.083	0.226	2.46E-06	Slc5a3	EpC_Ageddown	EpC
1.45E-06	-0.205104	0.335	0.446	0.03483	Acaa1a	EpC_Ageddown	EpC
1.92E-06	-0.207885	0.357	0.47	0.046103	Dnajb11	EpC_Ageddown	EpC
3.05E-08	-0.208339	0.983	0.997	0.000732	Rpl10	EpC_Ageddown	EpC
9.13E-09	-0.208756	0.339	0.509	0.000219	Gstm2	EpC_Ageddown	EpC
2.71E-09	-0.210587	0.1	0.236	6.50E-05	Frat2	EpC_Ageddown	EpC
3.00E-10	-0.21112	0.104	0.249	7.20E-06	Lman2l	EpC_Ageddown	EpC
2.02E-06	-0.212646	0.535	0.664	0.048412	Cct8	EpC_Ageddown	EpC
5.84E-11	-0.213368	0.167	0.342	1.40E-06	Ahsa2	EpC_Ageddown	EpC
8.73E-09	-0.213514	0.07	0.187	0.000209	Mslnl	EpC_Ageddown	EpC
7.08E-07	-0.213754	0.77	0.831	0.016988	Cnbp	EpC_Ageddown	EpC
9.68E-12	-0.214786	0.065	0.217	2.32E-07	Gm12446	EpC_Ageddown	EpC
1.40E-11	-0.215034	0.037	0.166	3.36E-07	Cdc42ep2	EpC_Ageddown	EpC
8.40E-07	-0.215785	0.32	0.446	0.020132	Exosc7	EpC_Ageddown	EpC
7.65E-07	-0.216675	0.487	0.593	0.01835	Acp1	EpC_Ageddown	EpC
1.62E-10	-0.217466	0.041	0.165	3.88E-06	Dhrs9	EpC_Ageddown	EpC
6.29E-07	-0.219937	0.513	0.631	0.015095	Cct3	EpC_Ageddown	EpC
1.13E-06	-0.224297	0.346	0.47	0.027003	Gadd45gip1	EpC_Ageddown	EpC
2.36E-07	-0.226485	0.811	0.858	0.005667	Ppp1cb	EpC_Ageddown	EpC
1.56E-06	-0.226598	0.4	0.524	0.037363	Tob2	EpC_Ageddown	EpC
7.52E-08	-0.227199	0.165	0.313	0.001803	Rdh12	EpC_Ageddown	EpC
5.23E-07	-0.228542	0.943	0.971	0.012551	Dstn	EpC_Ageddown	EpC
3.83E-10	-0.229056	0.25	0.421	9.18E-06	Prnp	EpC_Ageddown	EpC
1.69E-06	-0.230348	0.267	0.394	0.040535	Amotl1	EpC_Ageddown	EpC
1.45E-06	-0.230875	0.404	0.538	0.034687	Cldnd1	EpC_Ageddown	EpC
1.73E-07	-0.231434	0.424	0.559	0.004159	Lamtor1	EpC_Ageddown	EpC
3.07E-07	-0.231622	0.972	0.993	0.007361	Cox4i1	EpC_Ageddown	EpC
5.69E-08	-0.232685	0.304	0.443	0.001364	Ppm1l	EpC_Ageddown	EpC
5.63E-07	-0.233763	0.567	0.656	0.013512	Pdia6	EpC_Ageddown	EpC
6.56E-15	-0.233775	0.109	0.305	1.57E-10	Eif2s3y	EpC_Ageddown	EpC
2.52E-08	-0.234011	0.165	0.295	0.000605	Gsta2	EpC_Ageddown	EpC
8.28E-09	-0.23496	0.785	0.865	0.000199	Lamp2	EpC_Ageddown	EpC
2.46E-09	-0.235406	0.204	0.353	5.91E-05	Hdac1	EpC_Ageddown	EpC
5.86E-10	-0.235805	0.513	0.654	1.41E-05	Pura	EpC_Ageddown	EpC
2.53E-10	-0.236232	0.313	0.47	6.06E-06	Mettl23	EpC_Ageddown	EpC
4.68E-09	-0.236259	0.33	0.507	0.000112	Trp63	EpC_Ageddown	EpC
1.68E-06	-0.237105	0.58	0.674	0.04033	U2af1	EpC_Ageddown	EpC
1.31E-06	-0.239173	0.639	0.709	0.031317	Rdx	EpC_Ageddown	EpC
1.79E-07	-0.239385	0.496	0.598	0.004291	Srp72	EpC_Ageddown	EpC
5.96E-07	-0.239828	0.204	0.329	0.014304	Elovl6	EpC_Ageddown	EpC
1.25E-12	-0.240266	0.033	0.168	3.00E-08	Abo	EpC_Ageddown	EpC
2.53E-12	-0.240884	0.113	0.281	6.08E-08	Hlf	EpC_Ageddown	EpC
8.18E-07	-0.241051	0.554	0.661	0.019626	Pgd	EpC_Ageddown	EpC
8.60E-08	-0.242003	0.843	0.91	0.002062	Gpx1	EpC_Ageddown	EpC
1.73E-06	-0.244007	0.652	0.74	0.041511	Arpc5	EpC_Ageddown	EpC
8.72E-10	-0.244389	0.161	0.314	2.09E-05	Rgma	EpC_Ageddown	EpC
2.82E-09	-0.245593	0.137	0.272	6.76E-05	Frmd4a	EpC_Ageddown	EpC
3.79E-07	-0.245896	0.346	0.476	0.009082	Gnaq	EpC_Ageddown	EpC
1.53E-07	-0.247966	0.378	0.504	0.003674	Becn1	EpC_Ageddown	EpC
3.48E-09	-0.249198	0.122	0.256	8.34E-05	Gm20186	EpC_Ageddown	EpC
7.51E-09	-0.249443	0.957	0.986	0.00018	Ddx5	EpC_Ageddown	EpC

2.71E-13	-0.24982	0.098	0.265	6.49E-09	Pla2g12a	EpC_Ageddown	EpC
8.77E-08	-0.250884	0.548	0.667	0.002103	Arl1	EpC_Ageddown	EpC
4.64E-09	-0.251352	0.674	0.808	0.000111	Brk1	EpC_Ageddown	EpC
1.58E-10	-0.252958	0.098	0.242	3.79E-06	Retsat	EpC_Ageddown	EpC
1.09E-06	-0.253776	0.402	0.531	0.026058	Zfhx3	EpC_Ageddown	EpC
1.72E-07	-0.254795	0.985	0.997	0.004121	Fxyd3	EpC_Ageddown	EpC
3.22E-07	-0.255222	0.793	0.858	0.007733	Hnrnpf	EpC_Ageddown	EpC
2.17E-08	-0.260096	0.433	0.567	0.00052	Banf1	EpC_Ageddown	EpC
1.04E-09	-0.261417	0.115	0.253	2.50E-05	Prr15	EpC_Ageddown	EpC
1.69E-06	-0.261881	0.217	0.339	0.040428	Fam117b	EpC_Ageddown	EpC
6.35E-10	-0.26198	0.583	0.734	1.52E-05	Cd164	EpC_Ageddown	EpC
3.06E-10	-0.262763	0.454	0.598	7.33E-06	Yipf4	EpC_Ageddown	EpC
2.08E-08	-0.264029	0.4	0.54	0.000498	Snrnp48	EpC_Ageddown	EpC
5.54E-23	-0.265204	1	1	1.33E-18	mt-Co2	EpC_Ageddown	EpC
1.20E-09	-0.265476	0.917	0.97	2.89E-05	Arf5	EpC_Ageddown	EpC
8.07E-07	-0.266046	0.533	0.635	0.019356	Psmd12	EpC_Ageddown	EpC
6.16E-14	-0.266559	0	0.114	1.48E-09	St6galnac1	EpC_Ageddown	EpC
1.39E-08	-0.266685	0.563	0.689	0.000333	Srsf11	EpC_Ageddown	EpC
6.82E-10	-0.266763	0.783	0.907	1.63E-05	2010111I01Rik	EpC_Ageddown	EpC
1.17E-06	-0.270071	0.73	0.822	0.027948	Rab10	EpC_Ageddown	EpC
9.38E-10	-0.270878	0.58	0.7	2.25E-05	Psmd4	EpC_Ageddown	EpC
1.26E-06	-0.272354	0.207	0.327	0.030222	Vsig10l	EpC_Ageddown	EpC
1.28E-09	-0.272875	0.226	0.382	3.06E-05	Lxn	EpC_Ageddown	EpC
6.18E-07	-0.273047	0.43	0.538	0.014827	Strn3	EpC_Ageddown	EpC
4.80E-10	-0.27307	0.372	0.52	1.15E-05	Ap3s1	EpC_Ageddown	EpC
3.41E-11	-0.274244	0.23	0.397	8.17E-07	Nr1d2	EpC_Ageddown	EpC
5.21E-43	-0.274427	0.822	0.991	1.25E-38	Lipf	EpC_Ageddown	EpC
5.54E-13	-0.274816	0.039	0.181	1.33E-08	Il1r2	EpC_Ageddown	EpC
4.03E-07	-0.275702	0.604	0.695	0.009667	Pitpna	EpC_Ageddown	EpC
1.35E-09	-0.275929	0.854	0.938	3.25E-05	Sumo2	EpC_Ageddown	EpC
1.91E-07	-0.27607	0.202	0.324	0.004577	Cdc25b	EpC_Ageddown	EpC
1.55E-07	-0.278619	0.333	0.456	0.003718	Dnajb9	EpC_Ageddown	EpC
1.58E-10	-0.278622	0.737	0.847	3.78E-06	Tmed9	EpC_Ageddown	EpC
1.45E-07	-0.280405	0.493	0.628	0.003488	Map3k1	EpC_Ageddown	EpC
1.08E-08	-0.282248	0.691	0.784	0.000258	Hnrnpa0	EpC_Ageddown	EpC
4.14E-10	-0.282526	0.663	0.773	9.92E-06	Arf4	EpC_Ageddown	EpC
8.42E-10	-0.282749	0.465	0.619	2.02E-05	Nudc	EpC_Ageddown	EpC
3.33E-09	-0.285706	0.596	0.712	7.97E-05	Cct5	EpC_Ageddown	EpC
2.46E-12	-0.286512	0.887	0.971	5.91E-08	Ppib	EpC_Ageddown	EpC
6.43E-08	-0.287102	0.47	0.592	0.001541	Map4k4	EpC_Ageddown	EpC
3.45E-09	-0.287109	0.248	0.394	8.28E-05	Arl4a	EpC_Ageddown	EpC
4.07E-07	-0.287698	0.67	0.764	0.009755	AY036118	EpC_Ageddown	EpC
2.01E-12	-0.288329	0.276	0.476	4.82E-08	Serinc2	EpC_Ageddown	EpC
1.93E-12	-0.290118	0.667	0.795	4.64E-08	Eif4a2	EpC_Ageddown	EpC
1.03E-17	-0.290899	0.009	0.165	2.47E-13	Cd200r2	EpC_Ageddown	EpC
3.64E-09	-0.292298	0.343	0.502	8.73E-05	Tmem65	EpC_Ageddown	EpC
7.40E-10	-0.297509	0.635	0.764	1.77E-05	Psmd8	EpC_Ageddown	EpC
5.23E-07	-0.29765	0.207	0.321	0.012548	Tgm5	EpC_Ageddown	EpC
4.51E-10	-0.29906	0.778	0.876	1.08E-05	Spcs2	EpC_Ageddown	EpC
7.75E-11	-0.299373	0.554	0.695	1.86E-06	Rbm8a	EpC_Ageddown	EpC
6.43E-12	-0.299802	0.18	0.352	1.54E-07	Apobec3	EpC_Ageddown	EpC
3.84E-14	-0.300084	0.159	0.352	9.20E-10	Rassf9	EpC_Ageddown	EpC
2.25E-08	-0.301487	0.678	0.755	0.000538	Hspd1	EpC_Ageddown	EpC
4.34E-12	-0.302177	0.259	0.456	1.04E-07	Entpd5	EpC_Ageddown	EpC
9.33E-12	-0.310734	0.317	0.499	2.24E-07	Tmem165	EpC_Ageddown	EpC
7.65E-08	-0.310922	0.624	0.75	0.001835	Tst	EpC_Ageddown	EpC
2.23E-11	-0.311847	0.8	0.886	5.35E-07	Ube2d3	EpC_Ageddown	EpC
3.30E-07	-0.3128	0.304	0.424	0.007914	Bag2	EpC_Ageddown	EpC
1.82E-09	-0.313028	0.67	0.784	4.36E-05	Erp29	EpC_Ageddown	EpC

4.91E-08	-0.313672	0.763	0.823	0.001177	Mdh1	EpC_Ageddown	EpC
4.28E-10	-0.31392	0.563	0.687	1.03E-05	Cct4	EpC_Ageddown	EpC
1.06E-06	-0.314539	0.787	0.852	0.025319	Map1lc3a	EpC_Ageddown	EpC
3.02E-08	-0.316133	0.589	0.699	0.000725	Cd47	EpC_Ageddown	EpC
4.03E-11	-0.31643	0.091	0.242	9.66E-07	Ces2f	EpC_Ageddown	EpC
1.24E-07	-0.317558	0.728	0.781	0.00298	Purb	EpC_Ageddown	EpC
3.92E-11	-0.317976	0.707	0.812	9.39E-07	Ptges3	EpC_Ageddown	EpC
1.04E-12	-0.318782	0.309	0.499	2.49E-08	Zkscan3	EpC_Ageddown	EpC
2.20E-13	-0.319657	0.172	0.372	5.28E-09	Serpinh1	EpC_Ageddown	EpC
9.97E-10	-0.320258	0.991	1	2.39E-05	Rpl26	EpC_Ageddown	EpC
1.69E-11	-0.321871	0.433	0.603	4.06E-07	Stip1	EpC_Ageddown	EpC
4.71E-09	-0.321945	0.82	0.88	0.000113	Pcbp2	EpC_Ageddown	EpC
9.04E-15	-0.327268	0.05	0.211	2.17E-10	Aldh1a7	EpC_Ageddown	EpC
1.15E-06	-0.327414	0.439	0.556	0.027687	Eif4e2	EpC_Ageddown	EpC
8.73E-09	-0.329212	0.289	0.427	0.000209	Coq10b	EpC_Ageddown	EpC
1.75E-06	-0.329455	0.435	0.562	0.04188	Tmprss4	EpC_Ageddown	EpC
1.70E-12	-0.330433	0.374	0.564	4.08E-08	Lap3	EpC_Ageddown	EpC
9.00E-10	-0.338427	0.204	0.355	2.16E-05	Cyp2j6	EpC_Ageddown	EpC
4.03E-10	-0.338903	0.637	0.742	9.66E-06	Hnrnpc	EpC_Ageddown	EpC
7.01E-09	-0.339671	0.978	0.996	0.000168	Rpl17	EpC_Ageddown	EpC
1.95E-07	-0.339869	0.411	0.547	0.004686	Tuba4a	EpC_Ageddown	EpC
2.89E-15	-0.341148	0.196	0.41	6.94E-11	Per3	EpC_Ageddown	EpC
7.44E-07	-0.341654	0.783	0.841	0.017833	Srp14	EpC_Ageddown	EpC
1.27E-18	-0.348943	0.067	0.265	3.04E-14	Cd164l2	EpC_Ageddown	EpC
1.42E-12	-0.35253	0.37	0.55	3.41E-08	Smpdl3a	EpC_Ageddown	EpC
2.74E-15	-0.353733	0.315	0.515	6.56E-11	Rassf1	EpC_Ageddown	EpC
4.80E-13	-0.353997	0.167	0.346	1.15E-08	Eid1	EpC_Ageddown	EpC
8.11E-15	-0.354708	0.341	0.528	1.94E-10	Tmem33	EpC_Ageddown	EpC
1.55E-15	-0.355578	0.361	0.59	3.73E-11	Tnrc6c	EpC_Ageddown	EpC
4.55E-21	-0.355879	0.222	0.488	1.09E-16	Tef	EpC_Ageddown	EpC
1.18E-13	-0.357497	0.617	0.768	2.82E-09	Dnaja2	EpC_Ageddown	EpC
5.31E-16	-0.359042	0.839	0.923	1.27E-11	Rtn4	EpC_Ageddown	EpC
3.81E-10	-0.359737	0.196	0.352	9.14E-06	Me1	EpC_Ageddown	EpC
1.03E-11	-0.361208	0.122	0.287	2.47E-07	Prss23	EpC_Ageddown	EpC
1.31E-12	-0.370239	0.276	0.465	3.14E-08	Tnfaip8	EpC_Ageddown	EpC
3.06E-08	-0.372087	0.446	0.583	0.000735	Elovl1	EpC_Ageddown	EpC
1.79E-12	-0.374413	0.089	0.247	4.29E-08	Fam89a	EpC_Ageddown	EpC
3.60E-09	-0.374425	0.454	0.595	8.63E-05	Cbr3	EpC_Ageddown	EpC
1.28E-15	-0.376408	0.337	0.537	3.08E-11	Chordc1	EpC_Ageddown	EpC
2.38E-12	-0.376518	0.089	0.258	5.71E-08	Nrp2	EpC_Ageddown	EpC
7.74E-12	-0.376907	0.202	0.386	1.86E-07	Mme	EpC_Ageddown	EpC
1.07E-20	-0.379608	0.97	0.994	2.56E-16	mt-Nd3	EpC_Ageddown	EpC
8.22E-26	-0.379948	0.996	1	1.97E-21	Tpt1	EpC_Ageddown	EpC
1.63E-09	-0.380373	0.665	0.784	3.90E-05	Cltb	EpC_Ageddown	EpC
4.53E-11	-0.380751	0.267	0.433	1.09E-06	Ammecr1	EpC_Ageddown	EpC
1.26E-13	-0.381572	0.667	0.815	3.02E-09	Atp6v1a	EpC_Ageddown	EpC
2.72E-11	-0.382646	0.198	0.385	6.52E-07	Ces2g	EpC_Ageddown	EpC
2.34E-12	-0.383251	0.485	0.632	5.62E-08	Med21	EpC_Ageddown	EpC
1.22E-14	-0.384943	0.041	0.2	2.93E-10	Gjb6	EpC_Ageddown	EpC
4.66E-08	-0.385167	0.807	0.839	0.001118	Sf3b6	EpC_Ageddown	EpC
4.26E-09	-0.389528	0.748	0.835	0.000102	Hras	EpC_Ageddown	EpC
9.97E-09	-0.389719	0.183	0.331	0.000239	Ces1h	EpC_Ageddown	EpC
7.37E-17	-0.389886	0.72	0.873	1.77E-12	Eif5	EpC_Ageddown	EpC
9.25E-09	-0.390817	0.88	0.928	0.000222	Gapdh	EpC_Ageddown	EpC
8.76E-14	-0.396288	0.561	0.718	2.10E-09	Acadl	EpC_Ageddown	EpC
1.49E-09	-0.397605	0.37	0.533	3.58E-05	Wnt4	EpC_Ageddown	EpC
8.08E-11	-0.39779	0.104	0.252	1.94E-06	Gsdma	EpC_Ageddown	EpC
5.08E-09	-0.398631	0.617	0.724	0.000122	Pgam1	EpC_Ageddown	EpC
1.29E-14	-0.399184	0.102	0.287	3.09E-10	UMuCg	EpC_Ageddown	EpC

2.23E-14	-0.401458	0.652	0.783	5.34E-10	Bcap31	EpC_Ageddown	EpC
4.11E-12	-0.401689	0.809	0.902	9.86E-08	Slc25a5	EpC_Ageddown	EpC
1.36E-16	-0.403035	0.761	0.857	3.26E-12	Pcbp1	EpC_Ageddown	EpC
2.51E-08	-0.40542	0.522	0.645	0.000603	Serpinb5	EpC_Ageddown	EpC
9.66E-13	-0.406171	0.915	0.967	2.32E-08	Calm1	EpC_Ageddown	EpC
1.30E-07	-0.40693	0.378	0.495	0.003125	Rab27b	EpC_Ageddown	EpC
1.31E-07	-0.410756	0.954	0.984	0.003139	Dynll1	EpC_Ageddown	EpC
1.44E-12	-0.41083	0.991	0.994	3.46E-08	Rplp1	EpC_Ageddown	EpC
1.99E-13	-0.412906	0.667	0.806	4.78E-09	Anp32b	EpC_Ageddown	EpC
8.00E-10	-0.413491	0.309	0.457	1.92E-05	F3	EpC_Ageddown	EpC
3.07E-09	-0.415063	0.648	0.76	7.37E-05	Dynlt3	EpC_Ageddown	EpC
4.08E-07	-0.417642	0.237	0.375	0.009787	Ces1f	EpC_Ageddown	EpC
4.23E-33	-0.420232	0.093	0.433	1.01E-28	Ddx3y	EpC_Ageddown	EpC
1.10E-11	-0.422223	0.615	0.748	2.64E-07	Spop	EpC_Ageddown	EpC
2.26E-20	-0.422334	0.85	0.938	5.42E-16	Laptm4a	EpC_Ageddown	EpC
6.94E-12	-0.422534	0.617	0.747	1.67E-07	Uqcrc2	EpC_Ageddown	EpC
6.72E-18	-0.42503	0.233	0.457	1.61E-13	Mrps6	EpC_Ageddown	EpC
2.32E-22	-0.425315	0.089	0.333	5.57E-18	Dbp	EpC_Ageddown	EpC
2.61E-17	-0.428459	0.717	0.877	6.25E-13	Top1	EpC_Ageddown	EpC
4.49E-17	-0.435086	0.891	0.945	1.08E-12	Eif4a1	EpC_Ageddown	EpC
3.29E-21	-0.435116	0.398	0.682	7.88E-17	Amy1	EpC_Ageddown	EpC
1.92E-12	-0.436931	0.428	0.621	4.61E-08	Ablim1	EpC_Ageddown	EpC
6.42E-16	-0.438295	0.67	0.808	1.54E-11	Herpud1	EpC_Ageddown	EpC
1.81E-28	-0.443754	0.996	1	4.35E-24	mt-Nd1	EpC_Ageddown	EpC
3.68E-10	-0.443874	0.896	0.948	8.83E-06	Ubc	EpC_Ageddown	EpC
3.06E-21	-0.444487	0.65	0.821	7.34E-17	Sumo1	EpC_Ageddown	EpC
3.78E-14	-0.444558	0.261	0.465	9.06E-10	Selenbp1	EpC_Ageddown	EpC
1.01E-13	-0.444937	0.833	0.9	2.42E-09	Hspe1	EpC_Ageddown	EpC
1.35E-10	-0.446313	0.448	0.603	3.24E-06	Atp11b	EpC_Ageddown	EpC
2.99E-10	-0.447683	0.226	0.382	7.16E-06	Egln3	EpC_Ageddown	EpC
4.79E-20	-0.453731	0.685	0.834	1.15E-15	P4hb	EpC_Ageddown	EpC
4.08E-07	-0.461316	0.237	0.375	0.009787	Ccnd2	EpC_Ageddown	EpC
3.31E-30	-0.464397	0.983	0.996	7.93E-26	Itm2b	EpC_Ageddown	EpC
1.60E-13	-0.465556	0.233	0.424	3.83E-09	Aldh3b2	EpC_Ageddown	EpC
2.12E-13	-0.465609	0.154	0.34	5.09E-09	Tmprss11g	EpC_Ageddown	EpC
3.51E-14	-0.469839	0.354	0.563	8.42E-10	MuClm	EpC_Ageddown	EpC
3.65E-15	-0.470014	0.68	0.828	8.76E-11	Eif3e	EpC_Ageddown	EpC
1.27E-08	-0.471635	0.424	0.547	0.000303	Abhd17c	EpC_Ageddown	EpC
8.71E-20	-0.471871	0.991	0.999	2.09E-15	Rps14	EpC_Ageddown	EpC
4.54E-10	-0.47232	0.733	0.81	1.09E-05	Esd	EpC_Ageddown	EpC
8.14E-13	-0.473033	0.85	0.903	1.95E-08	Hmgn1	EpC_Ageddown	EpC
6.03E-15	-0.479551	0.561	0.716	1.45E-10	Cnih4	EpC_Ageddown	EpC
7.68E-14	-0.483151	0.452	0.635	1.84E-09	Golim4	EpC_Ageddown	EpC
1.47E-07	-0.483527	0.776	0.839	0.003522	Tubb4b	EpC_Ageddown	EpC
1.33E-15	-0.485779	0.754	0.851	3.19E-11	Arpc2	EpC_Ageddown	EpC
1.72E-09	-0.488338	0.807	0.92	4.11E-05	Cyp2f2	EpC_Ageddown	EpC
2.81E-17	-0.488781	0.474	0.673	6.74E-13	Gstm1	EpC_Ageddown	EpC
9.91E-17	-0.494957	0.58	0.754	2.38E-12	Tcf4	EpC_Ageddown	EpC
4.84E-16	-0.499996	0.613	0.774	1.16E-11	Anxa7	EpC_Ageddown	EpC
9.40E-09	-0.507043	0.678	0.748	0.000226	Mgst3	EpC_Ageddown	EpC
7.89E-20	-0.507846	0.576	0.755	1.89E-15	Psmd7	EpC_Ageddown	EpC
2.13E-18	-0.511631	0.161	0.427	5.10E-14	Krt15	EpC_Ageddown	EpC
1.43E-12	-0.515082	0.313	0.483	3.42E-08	Xpa	EpC_Ageddown	EpC
4.26E-32	-0.515238	0.987	1	1.02E-27	H3f3a	EpC_Ageddown	EpC
2.77E-24	-0.518838	0.624	0.813	6.64E-20	Fkbp4	EpC_Ageddown	EpC
5.35E-08	-0.520833	0.376	0.504	0.001284	Cited2	EpC_Ageddown	EpC
7.34E-20	-0.521918	0.954	0.988	1.76E-15	Rps25	EpC_Ageddown	EpC
6.08E-19	-0.523722	0.707	0.841	1.46E-14	Manf	EpC_Ageddown	EpC
5.76E-20	-0.526588	0.428	0.645	1.38E-15	Cux1	EpC_Ageddown	EpC

3.36E-16	-0.529869	0.813	0.893	8.05E-12	Skp1a	EpC_Ageddown	EpC
1.16E-06	-0.532218	0.467	0.564	0.027807	Odc1	EpC_Ageddown	EpC
1.21E-25	-0.534276	0.476	0.722	2.89E-21	Cacybp	EpC_Ageddown	EpC
7.41E-15	-0.537023	0.943	0.977	1.78E-10	Rps15	EpC_Ageddown	EpC
4.11E-37	-0.549243	0.841	0.991	9.86E-33	Sbpl	EpC_Ageddown	EpC
1.18E-15	-0.549275	0.598	0.745	2.83E-11	Sri	EpC_Ageddown	EpC
2.16E-25	-0.550851	0.978	0.997	5.17E-21	Rpl21	EpC_Ageddown	EpC
2.53E-20	-0.555776	0.846	0.925	6.07E-16	Hspa5	EpC_Ageddown	EpC
2.56E-20	-0.561061	0.507	0.705	6.13E-16	Gm16136	EpC_Ageddown	EpC
1.38E-06	-0.5631	0.691	0.787	0.033071	Ier3	EpC_Ageddown	EpC
2.16E-07	-0.568576	0.12	0.239	0.005188	Bpifa2	EpC_Ageddown	EpC
2.36E-15	-0.568947	0.276	0.488	5.67E-11	Mafb	EpC_Ageddown	EpC
2.51E-13	-0.571682	0.283	0.459	6.01E-09	Ethe1	EpC_Ageddown	EpC
7.77E-13	-0.578955	0.191	0.372	1.86E-08	Cyp3a13	EpC_Ageddown	EpC
3.66E-28	-0.581654	0.82	0.936	8.79E-24	Calr	EpC_Ageddown	EpC
4.27E-13	-0.587305	0.102	0.262	1.02E-08	Mndal	EpC_Ageddown	EpC
1.09E-15	-0.591392	0.311	0.559	2.63E-11	Id1	EpC_Ageddown	EpC
5.73E-15	-0.597133	0.376	0.556	1.37E-10	Arap2	EpC_Ageddown	EpC
5.23E-11	-0.59751	0.728	0.88	1.26E-06	Lgals7	EpC_Ageddown	EpC
9.96E-17	-0.598935	0.626	0.781	2.39E-12	Marcks	EpC_Ageddown	EpC
1.52E-21	-0.599934	0.728	0.844	3.64E-17	Prdx6	EpC_Ageddown	EpC
2.01E-23	-0.601414	0.987	0.999	4.82E-19	Rps8	EpC_Ageddown	EpC
1.69E-08	-0.606723	0.202	0.349	0.000405	Serpinb11	EpC_Ageddown	EpC
1.65E-12	-0.618075	0.272	0.438	3.95E-08	Paqr5	EpC_Ageddown	EpC
1.10E-11	-0.622358	0.152	0.317	2.65E-07	Ephx3	EpC_Ageddown	EpC
2.15E-12	-0.623755	0.035	0.168	5.16E-08	Srgn	EpC_Ageddown	EpC
2.90E-35	-0.627991	0.996	1	6.96E-31	Cd9	EpC_Ageddown	EpC
4.31E-24	-0.634753	0.924	0.975	1.03E-19	Rpl23a	EpC_Ageddown	EpC
1.92E-06	-0.635614	0.933	0.942	0.046079	Gsta4	EpC_Ageddown	EpC
1.05E-13	-0.636185	0.424	0.588	2.52E-09	Tpm2	EpC_Ageddown	EpC
1.67E-10	-0.644887	0.628	0.724	3.99E-06	Ggh	EpC_Ageddown	EpC
3.06E-12	-0.650404	0.822	0.893	7.33E-08	Zfp36l1	EpC_Ageddown	EpC
6.55E-17	-0.653592	0.672	0.848	1.57E-12	Pycard	EpC_Ageddown	EpC
8.84E-15	-0.659503	0.487	0.666	2.12E-10	Gadd45b	EpC_Ageddown	EpC
1.39E-14	-0.661305	0.335	0.535	3.33E-10	Them5	EpC_Ageddown	EpC
2.20E-07	-0.681109	0.765	0.89	0.005276	Krt6a	EpC_Ageddown	EpC
6.17E-15	-0.682929	0.335	0.518	1.48E-10	Htatip2	EpC_Ageddown	EpC
5.39E-11	-0.684027	0.235	0.412	1.29E-06	Csta1	EpC_Ageddown	EpC
1.33E-11	-0.703048	0.341	0.543	3.18E-07	Calm4	EpC_Ageddown	EpC
3.83E-23	-0.704715	0.948	0.987	9.18E-19	Rpl12	EpC_Ageddown	EpC
4.56E-28	-0.706874	0.546	0.78	1.09E-23	Pax1	EpC_Ageddown	EpC
6.38E-14	-0.719155	0.848	0.915	1.53E-09	Gpx2	EpC_Ageddown	EpC
5.21E-20	-0.724249	0.113	0.343	1.25E-15	Aldh1a1	EpC_Ageddown	EpC
2.93E-24	-0.733864	0.085	0.34	7.04E-20	Endou	EpC_Ageddown	EpC
8.01E-62	-0.750154	0.985	1	1.92E-57	Ubb	EpC_Ageddown	EpC
9.85E-56	-0.764923	0.974	1	2.36E-51	Hsp90ab1	EpC_Ageddown	EpC
2.97E-30	-0.765472	0.654	0.857	7.12E-26	Ctsl	EpC_Ageddown	EpC
2.85E-27	-0.779148	0.972	0.994	6.82E-23	Rps12	EpC_Ageddown	EpC
8.01E-32	-0.782081	0.624	0.867	1.92E-27	Socs2	EpC_Ageddown	EpC
1.51E-55	-0.783659	0.952	0.991	3.62E-51	Hspa8	EpC_Ageddown	EpC
3.39E-100	-0.784032	1	1	8.14E-96	mt-Cytb	EpC_Ageddown	EpC
3.09E-15	-0.786042	0.08	0.262	7.40E-11	Il1a	EpC_Ageddown	EpC
2.53E-79	-0.786894	1	1	6.06E-75	mt-Nd2	EpC_Ageddown	EpC
1.08E-16	-0.789991	0.435	0.625	2.60E-12	Anxa8	EpC_Ageddown	EpC
3.77E-08	-0.797667	0.77	0.808	0.000903	Atp6v1e1	EpC_Ageddown	EpC
3.09E-15	-0.80463	0.683	0.841	7.40E-11	Fabp5	EpC_Ageddown	EpC
2.19E-24	-0.805334	0.898	0.952	5.24E-20	Prdx2	EpC_Ageddown	EpC
1.92E-20	-0.820607	0.541	0.703	4.61E-16	Sox2	EpC_Ageddown	EpC
1.88E-10	-0.822528	0.304	0.462	4.50E-06	Pinlyp	EpC_Ageddown	EpC

1.72E-30	-0.827197	0.467	0.773	4.13E-26	Capns2	EpC_Ageddown	EpC
1.64E-06	-0.838012	0.396	0.54	0.039226	Msln	EpC_Ageddown	EpC
1.52E-23	-0.851612	0.15	0.428	3.65E-19	Il33	EpC_Ageddown	EpC
5.20E-114	-0.861198	1	1	1.25E-109	mt-Atp6	EpC_Ageddown	EpC
9.97E-56	-0.886939	0.754	0.944	2.39E-51	Dnaja1	EpC_Ageddown	EpC
1.86E-29	-0.889531	0.161	0.482	4.45E-25	Foxe1	EpC_Ageddown	EpC
1.26E-24	-0.905387	0.18	0.467	3.02E-20	Id3	EpC_Ageddown	EpC
4.69E-10	-0.918232	0.061	0.192	1.12E-05	2310046K23Rik	EpC_Ageddown	EpC
2.02E-19	-0.948314	0.976	0.991	4.83E-15	Anxa1	EpC_Ageddown	EpC
1.21E-16	-0.950295	0.137	0.343	2.91E-12	Fetub	EpC_Ageddown	EpC
1.59E-17	-0.959136	0.274	0.505	3.81E-13	Sptssb	EpC_Ageddown	EpC
9.69E-16	-0.998864	0.178	0.384	2.32E-11	Elovl4	EpC_Ageddown	EpC
6.65E-23	-1.006681	0.067	0.308	1.59E-18	Gm8882	EpC_Ageddown	EpC
1.21E-123	-1.036069	1	1	2.91E-119	mt-Co3	EpC_Ageddown	EpC
5.67E-37	-1.072234	0.485	0.79	1.36E-32	Ces1d	EpC_Ageddown	EpC
5.41E-67	-1.09531	0.285	0.747	1.30E-62	Hsph1	EpC_Ageddown	EpC
2.32E-25	-1.108688	0.578	0.893	5.56E-21	Krt13	EpC_Ageddown	EpC
2.51E-33	-1.174002	0.759	0.949	6.02E-29	Hspb1	EpC_Ageddown	EpC
1.22E-07	-1.184538	0.376	0.485	0.002934	Dmbt1	EpC_Ageddown	EpC
2.01E-18	-1.261665	0.265	0.472	4.82E-14	Cst6	EpC_Ageddown	EpC
4.46E-13	-1.27814	0.246	0.423	1.07E-08	Spink5	EpC_Ageddown	EpC
6.31E-22	-1.282227	0.665	0.828	1.51E-17	Aldh3a1	EpC_Ageddown	EpC
1.03E-108	-1.348782	0.209	0.915	2.47E-104	Tmem59	EpC_Ageddown	EpC
6.40E-07	-1.433502	0.052	0.143	0.015359	Lce3a	EpC_Ageddown	EpC
1.64E-110	-1.493964	0.804	0.99	3.92E-106	Hsp90aa1	EpC_Ageddown	EpC
6.35E-101	-1.671021	0.643	0.949	1.52E-96	Hspa1b	EpC_Ageddown	EpC
1.86E-06	-1.795585	0.191	0.315	0.044644	2300002M23Rik	EpC_Ageddown	EpC
2.63E-43	-1.803909	0.204	0.608	6.32E-39	Adh7	EpC_Ageddown	EpC
1.30E-105	-1.907263	0.391	0.887	3.11E-101	Hspa1a	EpC_Ageddown	EpC
9.38E-08	-1.984383	0.322	0.433	0.002249	Ly6g6c	EpC_Ageddown	EpC
1.31E-34	-2.002548	0.163	0.504	3.15E-30	Tgm3	EpC_Ageddown	EpC
2.78E-35	-2.138431	0.113	0.46	6.68E-31	Krt75	EpC_Ageddown	EpC
2.40E-25	-2.171407	0.109	0.376	5.74E-21	Sprr2a3	EpC_Ageddown	EpC
7.58E-43	-2.505412	0.611	0.915	1.82E-38	Krt17	EpC_Ageddown	EpC
5.18E-79	9.5839385	0.742	0	1.24E-74	Tff2	MuC_Agedup	MuC
6.60E-57	6.504892	0.619	0.038	1.58E-52	Bpifb2	MuC_Agedup	MuC
2.13E-62	6.2715041	0.957	0.956	5.11E-58	Muc5b	MuC_Agedup	MuC
2.81E-27	4.8419379	0.751	0.611	6.73E-23	Agr2	MuC_Agedup	MuC
1.60E-16	3.6969115	0.628	0.604	3.83E-12	Gp2	MuC_Agedup	MuC
4.09E-48	3.5727777	0.768	0.392	9.81E-44	Tmsb10	MuC_Agedup	MuC
5.05E-44	3.2857908	0.963	0.965	1.21E-39	Nupr1	MuC_Agedup	MuC
4.69E-102	2.9584537	1	0.994	1.13E-97	Gm42418	MuC_Agedup	MuC
3.09E-50	2.9031608	0.527	0	7.40E-46	SmMuC	MuC_Agedup	MuC
9.84E-74	2.7931243	0.897	0.598	2.36E-69	Pigr	MuC_Agedup	MuC
6.40E-69	2.6910985	0.877	0.443	1.53E-64	Cst3	MuC_Agedup	MuC
3.09E-42	2.5273937	0.693	0.297	7.42E-38	Pam	MuC_Agedup	MuC
3.95E-42	2.3673532	0.553	0.089	9.47E-38	Lman1l	MuC_Agedup	MuC
1.85E-47	2.3542271	0.527	0.019	4.44E-43	AW112010	MuC_Agedup	MuC
1.14E-30	2.3197998	0.911	0.902	2.74E-26	Wfdc2	MuC_Agedup	MuC
1.25E-60	2.3174074	0.653	0.044	2.99E-56	Tesc	MuC_Agedup	MuC
1.49E-91	2.0497591	0.974	0.949	3.57E-87	Gm10076	MuC_Agedup	MuC
3.69E-69	2.0394166	0.94	0.953	8.85E-65	Ly6e	MuC_Agedup	MuC
5.91E-33	2.0234625	0.656	0.304	1.42E-28	Crip1	MuC_Agedup	MuC
3.29E-55	1.9171587	0.868	0.813	7.90E-51	Gfpt1	MuC_Agedup	MuC
6.05E-67	1.9095035	0.926	0.93	1.45E-62	Gng5	MuC_Agedup	MuC
8.94E-22	1.8680213	0.364	0.066	2.14E-17	Gm26870	MuC_Agedup	MuC
3.29E-52	1.8250439	0.708	0.193	7.89E-48	Galnt6	MuC_Agedup	MuC
3.27E-43	1.8070768	0.559	0.082	7.85E-39	Nkx3-1	MuC_Agedup	MuC
4.40E-93	1.8003969	0.986	0.978	1.06E-88	Pglyrp1	MuC_Agedup	MuC

1.12E-68	1.7865161	0.943	0.832	2.69E-64	mt-Atp8	MuC_Agedup	MuC
5.81E-44	1.7597007	0.556	0.073	1.39E-39	Basp1	MuC_Agedup	MuC
1.34E-60	1.7322478	0.888	0.791	3.22E-56	Ost4	MuC_Agedup	MuC
5.09E-53	1.7313708	0.848	0.604	1.22E-48	Snhg18	MuC_Agedup	MuC
5.88E-44	1.7122333	0.848	0.712	1.41E-39	Tspan13	MuC_Agedup	MuC
1.47E-47	1.6744992	0.837	0.595	3.52E-43	Mfsd4a	MuC_Agedup	MuC
2.31E-30	1.6671529	0.625	0.307	5.53E-26	Cldn2	MuC_Agedup	MuC
2.27E-82	1.663128	1	0.997	5.44E-78	mt-Nd4l	MuC_Agedup	MuC
1.14E-41	1.6615617	0.533	0.07	2.74E-37	Cgref1	MuC_Agedup	MuC
6.28E-79	1.6532488	0.977	0.981	1.51E-74	Rpl38	MuC_Agedup	MuC
8.98E-72	1.612546	0.946	0.937	2.15E-67	H2-K1	MuC_Agedup	MuC
5.27E-33	1.6121397	0.576	0.19	1.26E-28	Lrg1	MuC_Agedup	MuC
1.47E-32	1.5926207	0.819	0.788	3.53E-28	Pdia3	MuC_Agedup	MuC
6.60E-51	1.5827778	0.86	0.842	1.58E-46	Ndufa1	MuC_Agedup	MuC
1.06E-52	1.5825818	0.905	0.899	2.54E-48	Rabac1	MuC_Agedup	MuC
2.58E-37	1.5795721	0.725	0.415	6.19E-33	Tgoln1	MuC_Agedup	MuC
4.42E-38	1.5757369	0.61	0.199	1.06E-33	Hexb	MuC_Agedup	MuC
6.37E-62	1.5532433	0.934	0.946	1.53E-57	Sec61g	MuC_Agedup	MuC
4.51E-60	1.5513565	0.917	0.924	1.08E-55	Rrbp1	MuC_Agedup	MuC
1.08E-36	1.5385846	0.911	0.94	2.58E-32	Tmed3	MuC_Agedup	MuC
2.82E-42	1.5262446	0.542	0.073	6.76E-38	Golm1	MuC_Agedup	MuC
1.15E-44	1.5176784	0.507	0.022	2.76E-40	Oit1	MuC_Agedup	MuC
1.14E-27	1.5174049	0.653	0.354	2.75E-23	Klf4	MuC_Agedup	MuC
4.14E-84	1.5122235	0.991	0.991	9.93E-80	Rps28	MuC_Agedup	MuC
2.46E-35	1.4986226	0.842	0.851	5.91E-31	Cpd	MuC_Agedup	MuC
4.27E-62	1.489462	0.911	0.918	1.02E-57	Uqcr11	MuC_Agedup	MuC
7.78E-25	1.4861289	0.751	0.646	1.86E-20	Guk1	MuC_Agedup	MuC
7.36E-65	1.4852257	0.94	0.975	1.76E-60	Cox8a	MuC_Agedup	MuC
2.41E-41	1.4787809	0.45	0	5.78E-37	Muc16	MuC_Agedup	MuC
7.24E-46	1.4648403	0.49	0	1.74E-41	Papss2	MuC_Agedup	MuC
2.41E-81	1.4552525	0.997	0.994	5.79E-77	Rps29	MuC_Agedup	MuC
6.68E-47	1.4503962	0.888	0.899	1.60E-42	Fkbp2	MuC_Agedup	MuC
2.79E-68	1.4491424	0.977	0.978	6.69E-64	Ftl1	MuC_Agedup	MuC
3.38E-46	1.4440292	0.493	0	8.12E-42	Chst4	MuC_Agedup	MuC
2.14E-73	1.4355102	0.966	0.981	5.13E-69	Serf2	MuC_Agedup	MuC
3.76E-60	1.4331706	0.908	0.93	9.01E-56	Ndufa4	MuC_Agedup	MuC
5.84E-76	1.4234079	0.986	0.994	1.40E-71	Rpl37a	MuC_Agedup	MuC
6.37E-45	1.4189398	0.822	0.665	1.53E-40	Smim22	MuC_Agedup	MuC
1.61E-30	1.4183177	0.805	0.731	3.85E-26	Tmbim4	MuC_Agedup	MuC
1.28E-34	1.4166741	0.874	0.905	3.07E-30	Epcam	MuC_Agedup	MuC
3.66E-53	1.4134019	0.894	0.93	8.78E-49	Spint2	MuC_Agedup	MuC
4.71E-36	1.4015756	0.788	0.608	1.13E-31	Mia3	MuC_Agedup	MuC
9.76E-27	1.3771671	0.705	0.503	2.34E-22	Dnajc10	MuC_Agedup	MuC
2.92E-38	1.3684273	0.94	0.949	7.00E-34	Fth1	MuC_Agedup	MuC
4.83E-45	1.3598236	0.888	0.94	1.16E-40	Reep5	MuC_Agedup	MuC
1.72E-50	1.3564614	0.883	0.823	4.13E-46	Ndufa7	MuC_Agedup	MuC
6.59E-49	1.3502476	0.848	0.75	1.58E-44	Ndufv3	MuC_Agedup	MuC
2.19E-77	1.3487488	1	1	5.25E-73	Rpl41	MuC_Agedup	MuC
3.09E-50	1.3399877	0.527	0	7.40E-46	Xist	MuC_Agedup	MuC
1.23E-63	1.3374135	0.971	0.975	2.95E-59	Rps27	MuC_Agedup	MuC
2.40E-54	1.3311455	0.914	0.937	5.76E-50	Cox7c	MuC_Agedup	MuC
2.08E-53	1.3231575	0.903	0.892	4.98E-49	Cox6b1	MuC_Agedup	MuC
3.96E-69	1.3212736	0.983	0.984	9.50E-65	Rpl37	MuC_Agedup	MuC
3.39E-57	1.3199128	0.897	0.915	8.13E-53	Rpl36al	MuC_Agedup	MuC
5.27E-45	1.3125586	0.9	0.921	1.26E-40	Tmbim6	MuC_Agedup	MuC
4.64E-46	1.2878108	0.828	0.668	1.11E-41	Ndufa3	MuC_Agedup	MuC
6.54E-55	1.2876575	0.903	0.902	1.57E-50	Uqcrq	MuC_Agedup	MuC
2.46E-42	1.2864321	0.476	0.013	5.91E-38	Casc4	MuC_Agedup	MuC
6.32E-31	1.2768497	0.86	0.899	1.52E-26	Cd63	MuC_Agedup	MuC

8.82E-55	1.2751506	0.94	0.956	2.12E-50	Sec61b	MuC_Agedup	MuC
1.53E-39	1.2743383	0.868	0.896	3.68E-35	Tm9sf3	MuC_Agedup	MuC
1.90E-27	1.2732563	0.682	0.449	4.56E-23	Erlec1	MuC_Agedup	MuC
2.93E-31	1.2726707	0.731	0.528	7.03E-27	Mrps18a	MuC_Agedup	MuC
6.64E-58	1.2603408	0.926	0.93	1.59E-53	Atp5e	MuC_Agedup	MuC
1.47E-53	1.2568872	0.908	0.902	3.52E-49	Cox6c	MuC_Agedup	MuC
1.38E-65	1.2538991	0.96	0.943	3.32E-61	Rpl35	MuC_Agedup	MuC
3.14E-24	1.2489834	0.507	0.199	7.53E-20	Mgst2	MuC_Agedup	MuC
4.00E-42	1.2431523	0.871	0.854	9.60E-38	Hdlbp	MuC_Agedup	MuC
7.18E-50	1.2340623	0.891	0.88	1.72E-45	Atp5l	MuC_Agedup	MuC
9.05E-56	1.2340246	0.934	0.965	2.17E-51	Chchd2	MuC_Agedup	MuC
1.93E-11	1.2300592	0.65	0.608	4.63E-07	Gsto1	MuC_Agedup	MuC
5.28E-31	1.2297312	0.811	0.715	1.27E-26	Kdelr2	MuC_Agedup	MuC
4.00E-45	1.2288598	0.883	0.915	9.59E-41	Cox7a2	MuC_Agedup	MuC
1.61E-29	1.2259159	0.797	0.703	3.85E-25	Stard10	MuC_Agedup	MuC
3.96E-23	1.2207024	0.728	0.608	9.50E-19	Galnt7	MuC_Agedup	MuC
3.36E-23	1.2191736	0.716	0.547	8.06E-19	Tob1	MuC_Agedup	MuC
1.90E-36	1.2162438	0.622	0.209	4.56E-32	Scd2	MuC_Agedup	MuC
5.19E-26	1.213999	0.84	0.832	1.24E-21	Tceal9	MuC_Agedup	MuC
4.98E-38	1.2109489	0.862	0.877	1.19E-33	Hsp90b1	MuC_Agedup	MuC
9.78E-36	1.2092545	0.871	0.921	2.35E-31	Smim14	MuC_Agedup	MuC
1.58E-35	1.1993743	0.564	0.149	3.79E-31	S100a13	MuC_Agedup	MuC
2.16E-45	1.1986926	0.86	0.832	5.18E-41	Tomm7	MuC_Agedup	MuC
6.23E-21	1.1981746	0.659	0.465	1.49E-16	Cyba	MuC_Agedup	MuC
4.79E-46	1.1943139	0.874	0.832	1.15E-41	Uba52	MuC_Agedup	MuC
1.67E-39	1.1934986	0.831	0.756	4.01E-35	Isg20	MuC_Agedup	MuC
1.22E-36	1.1886286	0.914	0.943	2.93E-32	Mfge8	MuC_Agedup	MuC
1.17E-25	1.1864663	0.479	0.139	2.81E-21	Sgk1	MuC_Agedup	MuC
1.87E-23	1.1847276	0.742	0.566	4.49E-19	Hes1	MuC_Agedup	MuC
2.85E-31	1.1806168	0.911	0.968	6.84E-27	Ssr4	MuC_Agedup	MuC
1.16E-41	1.1796987	0.453	0	2.79E-37	Chst5	MuC_Agedup	MuC
6.25E-61	1.1775896	0.989	0.994	1.50E-56	Rps20	MuC_Agedup	MuC
1.11E-42	1.1714692	0.871	0.886	2.66E-38	Ubl5	MuC_Agedup	MuC
4.90E-42	1.1663429	0.851	0.848	1.17E-37	Selenok	MuC_Agedup	MuC
1.37E-21	1.1661529	0.754	0.725	3.29E-17	Bag1	MuC_Agedup	MuC
6.34E-29	1.1640258	0.673	0.383	1.52E-24	Atp1b1	MuC_Agedup	MuC
2.25E-34	1.16353	0.811	0.801	5.39E-30	Srpr	MuC_Agedup	MuC
1.34E-31	1.1628001	0.67	0.354	3.20E-27	Galnt4	MuC_Agedup	MuC
3.81E-40	1.1576831	0.642	0.212	9.13E-36	MuCc2	MuC_Agedup	MuC
3.69E-28	1.151629	0.822	0.861	8.84E-24	Ostc	MuC_Agedup	MuC
8.64E-48	1.1495589	0.891	0.953	2.07E-43	Dad1	MuC_Agedup	MuC
6.62E-41	1.1490465	0.883	0.921	1.59E-36	Uqcrh	MuC_Agedup	MuC
1.24E-36	1.1478087	0.421	0.009	2.96E-32	Ceacam10	MuC_Agedup	MuC
4.83E-31	1.1433024	0.759	0.617	1.16E-26	Golgb1	MuC_Agedup	MuC
2.73E-41	1.1426018	0.903	0.943	6.55E-37	Krtcap2	MuC_Agedup	MuC
5.91E-25	1.1370399	0.779	0.671	1.42E-20	Npc2	MuC_Agedup	MuC
1.48E-36	1.1370147	0.842	0.785	3.54E-32	Plet1	MuC_Agedup	MuC
2.55E-29	1.1358146	0.911	0.93	6.11E-25	Pdcd4	MuC_Agedup	MuC
7.54E-25	1.1324779	0.797	0.753	1.81E-20	Lman1	MuC_Agedup	MuC
3.07E-44	1.132363	0.854	0.763	7.37E-40	Romo1	MuC_Agedup	MuC
3.42E-39	1.126514	0.842	0.696	8.19E-35	Atp1a1	MuC_Agedup	MuC
2.90E-41	1.1263997	0.871	0.924	6.95E-37	Smdt1	MuC_Agedup	MuC
1.07E-37	1.1260847	0.891	0.896	2.57E-33	Tmed2	MuC_Agedup	MuC
3.52E-42	1.1222628	0.888	0.924	8.45E-38	Atp5g2	MuC_Agedup	MuC
1.07E-40	1.1222092	0.888	0.934	2.57E-36	Tmed10	MuC_Agedup	MuC
1.86E-27	1.1218665	0.315	0	4.45E-23	Olfm4	MuC_Agedup	MuC
1.56E-28	1.1217156	0.794	0.728	3.75E-24	Tmem258	MuC_Agedup	MuC
3.72E-41	1.1214232	0.831	0.801	8.93E-37	Atp5k	MuC_Agedup	MuC
2.01E-31	1.1197935	0.794	0.829	4.82E-27	Dpm3	MuC_Agedup	MuC

5.42E-32	1.1197333	0.819	0.835	1.30E-27	Sec62	MuC_Agedup	MuC
7.74E-35	1.1177035	0.848	0.832	1.86E-30	Cd164	MuC_Agedup	MuC
7.20E-45	1.1157785	0.88	0.864	1.73E-40	Uqcr10	MuC_Agedup	MuC
3.03E-21	1.1118606	0.96	0.959	7.27E-17	Actb	MuC_Agedup	MuC
8.42E-31	1.1104886	0.711	0.446	2.02E-26	Creb3l1	MuC_Agedup	MuC
2.43E-26	1.108421	0.865	0.899	5.83E-22	App	MuC_Agedup	MuC
3.11E-39	1.1063552	0.888	0.921	7.45E-35	Atp5g1	MuC_Agedup	MuC
2.38E-40	1.1052264	0.842	0.823	5.71E-36	Mrpl52	MuC_Agedup	MuC
1.49E-37	1.1043926	0.862	0.864	3.58E-33	Cox5b	MuC_Agedup	MuC
6.27E-36	1.101681	0.63	0.218	1.50E-31	Bace2	MuC_Agedup	MuC
5.65E-37	1.1002438	0.851	0.883	1.36E-32	Cox5a	MuC_Agedup	MuC
1.40E-40	1.0997175	0.453	0.006	3.36E-36	Scd1	MuC_Agedup	MuC
2.95E-30	1.0955845	0.774	0.652	7.09E-26	Golph3	MuC_Agedup	MuC
1.70E-57	1.0951601	0.937	0.953	4.09E-53	Oaz1	MuC_Agedup	MuC
3.79E-34	1.0945196	0.762	0.528	9.10E-30	Maged1	MuC_Agedup	MuC
2.61E-35	1.0932071	0.799	0.737	6.25E-31	Usmg5	MuC_Agedup	MuC
1.43E-31	1.0884174	0.659	0.326	3.42E-27	Sel1l3	MuC_Agedup	MuC
3.05E-39	1.0879176	0.86	0.826	7.31E-35	Rpl27	MuC_Agedup	MuC
1.81E-23	1.0802627	0.55	0.263	4.34E-19	Pmm1	MuC_Agedup	MuC
7.36E-24	1.0796673	0.67	0.434	1.76E-19	Nucb1	MuC_Agedup	MuC
1.48E-61	1.0763907	0.968	0.978	3.54E-57	Rpl36	MuC_Agedup	MuC
8.58E-31	1.0756076	0.851	0.861	2.06E-26	Lrrc26	MuC_Agedup	MuC
2.11E-20	1.0751686	0.822	0.864	5.07E-16	Slc12a2	MuC_Agedup	MuC
1.00E-39	1.0708577	0.868	0.915	2.40E-35	Atp5h	MuC_Agedup	MuC
4.24E-36	1.0699605	0.722	0.43	1.02E-31	Vkorc1	MuC_Agedup	MuC
1.17E-30	1.0695806	0.536	0.158	2.82E-26	Prss32	MuC_Agedup	MuC
7.59E-22	1.0635402	0.748	0.62	1.82E-17	P2rx4	MuC_Agedup	MuC
5.63E-34	1.059616	0.831	0.842	1.35E-29	Ndufb8	MuC_Agedup	MuC
1.98E-49	1.0543663	0.968	0.994	4.75E-45	Rpl30	MuC_Agedup	MuC
3.28E-29	1.0539506	0.88	0.949	7.86E-25	Ppib	MuC_Agedup	MuC
7.77E-26	1.0441426	0.782	0.737	1.86E-21	Mydgf	MuC_Agedup	MuC
2.36E-48	1.0412972	0.897	0.972	5.66E-44	Sec11c	MuC_Agedup	MuC
2.69E-21	1.0399984	0.768	0.75	6.46E-17	Tmem167	MuC_Agedup	MuC
3.06E-31	1.039957	0.814	0.813	7.34E-27	Npdc1	MuC_Agedup	MuC
1.14E-27	1.0373527	0.774	0.671	2.74E-23	Cmas	MuC_Agedup	MuC
2.54E-32	1.0344659	0.897	0.902	6.10E-28	Rpl22l1	MuC_Agedup	MuC
1.08E-37	1.0336368	0.966	0.981	2.60E-33	Fxyd3	MuC_Agedup	MuC
1.97E-27	1.0330155	0.47	0.127	4.73E-23	Ppic	MuC_Agedup	MuC
7.11E-40	1.0303633	0.458	0.016	1.70E-35	Mgat5	MuC_Agedup	MuC
3.80E-29	1.0246234	0.814	0.753	9.10E-25	Sec61a1	MuC_Agedup	MuC
5.09E-28	1.0232329	0.63	0.332	1.22E-23	Galnt12	MuC_Agedup	MuC
5.10E-45	1.022316	0.954	0.965	1.22E-40	Rps2	MuC_Agedup	MuC
2.26E-39	1.0196513	0.857	0.864	5.43E-35	Ndufa2	MuC_Agedup	MuC
2.11E-31	1.0188144	0.805	0.728	5.06E-27	Morf4l1	MuC_Agedup	MuC
7.92E-33	1.0184547	0.673	0.351	1.90E-28	Tmf1	MuC_Agedup	MuC
2.99E-36	1.0164034	0.862	0.861	7.17E-32	Sem1	MuC_Agedup	MuC
3.94E-40	1.0158057	0.986	0.978	9.44E-36	mt-Nd5	MuC_Agedup	MuC
2.65E-30	1.0129324	0.865	0.889	6.36E-26	Dnajc3	MuC_Agedup	MuC
2.02E-21	1.0102871	0.768	0.715	4.84E-17	Ywhaq	MuC_Agedup	MuC
9.40E-52	1.0101545	0.908	0.921	2.25E-47	Atp5j2	MuC_Agedup	MuC
8.35E-27	1.0098406	0.857	0.877	2.00E-22	Pfn1	MuC_Agedup	MuC
2.91E-22	1.0096547	0.788	0.772	6.98E-18	Txndc17	MuC_Agedup	MuC
8.83E-10	1.0086876	0.702	0.68	2.12E-05	Dusp1	MuC_Agedup	MuC
5.81E-33	1.0042215	0.837	0.804	1.39E-28	2010107E04Rik	MuC_Agedup	MuC
6.73E-23	1.0020769	0.774	0.734	1.61E-18	Mien1	MuC_Agedup	MuC
2.09E-23	1.0015765	0.84	0.87	5.01E-19	Manf	MuC_Agedup	MuC
8.98E-35	0.9996553	0.868	0.886	2.15E-30	Tcn2	MuC_Agedup	MuC
8.31E-39	0.9992628	0.88	0.918	1.99E-34	H2-D1	MuC_Agedup	MuC
9.57E-47	0.9884884	0.983	0.987	2.29E-42	Rpl39	MuC_Agedup	MuC

2.99E-10	0.9834788	0.401	0.222	7.17E-06	Cbr2	MuC_Agedup	MuC
4.60E-14	0.9822831	0.923	0.946	1.10E-09	Malat1	MuC_Agedup	MuC
7.33E-19	0.9779034	0.347	0.076	1.76E-14	Klf2	MuC_Agedup	MuC
2.59E-22	0.9738066	0.814	0.788	6.20E-18	Fkbp11	MuC_Agedup	MuC
2.03E-27	0.9727022	0.834	0.791	4.87E-23	Cox6a1	MuC_Agedup	MuC
2.34E-24	0.9676667	0.851	0.883	5.62E-20	Calr	MuC_Agedup	MuC
4.97E-27	0.9674508	0.842	0.88	1.19E-22	Spcs1	MuC_Agedup	MuC
2.38E-26	0.9669396	0.871	0.908	5.72E-22	Hspa5	MuC_Agedup	MuC
9.79E-39	0.966243	0.868	0.861	2.35E-34	Srp9	MuC_Agedup	MuC
1.32E-29	0.9634181	0.817	0.82	3.16E-25	D8Ertd738e	MuC_Agedup	MuC
3.46E-39	0.9586842	0.877	0.905	8.29E-35	Elob	MuC_Agedup	MuC
1.95E-19	0.9582812	0.705	0.608	4.67E-15	Dap	MuC_Agedup	MuC
4.79E-24	0.9582445	0.699	0.544	1.15E-19	H2-T23	MuC_Agedup	MuC
3.03E-31	0.9555521	0.834	0.848	7.26E-27	Scand1	MuC_Agedup	MuC
1.65E-26	0.9521449	0.834	0.845	3.95E-22	Etv1	MuC_Agedup	MuC
1.30E-24	0.9501572	0.822	0.813	3.12E-20	Atpif1	MuC_Agedup	MuC
2.59E-30	0.9494645	0.765	0.554	6.21E-26	Ctsb	MuC_Agedup	MuC
4.83E-34	0.9488615	0.665	0.313	1.16E-29	Fcgbp	MuC_Agedup	MuC
1.26E-49	0.943531	0.96	0.997	3.02E-45	Rpl11	MuC_Agedup	MuC
4.03E-39	0.9425034	0.688	0.275	9.67E-35	Mgst1	MuC_Agedup	MuC
2.58E-34	0.9419252	0.774	0.614	6.18E-30	Tmem256	MuC_Agedup	MuC
1.08E-30	0.9386012	0.862	0.854	2.59E-26	Ehf	MuC_Agedup	MuC
2.25E-31	0.9384411	0.642	0.282	5.40E-27	Hk2	MuC_Agedup	MuC
4.34E-30	0.9368067	0.825	0.782	1.04E-25	Psap	MuC_Agedup	MuC
1.57E-25	0.9348799	0.797	0.763	3.78E-21	Rpn1	MuC_Agedup	MuC
1.23E-33	0.9327108	0.842	0.87	2.94E-29	Txndc5	MuC_Agedup	MuC
4.78E-30	0.9317096	0.828	0.864	1.15E-25	Lamtor2	MuC_Agedup	MuC
4.81E-30	0.931498	0.802	0.81	1.15E-25	Ndufc1	MuC_Agedup	MuC
4.78E-25	0.9297089	0.822	0.813	1.15E-20	Vamp8	MuC_Agedup	MuC
7.19E-55	0.929439	0.983	0.984	1.72E-50	Rpl34	MuC_Agedup	MuC
2.50E-31	0.9268906	0.782	0.731	6.00E-27	Ndufb2	MuC_Agedup	MuC
4.92E-34	0.9265337	0.862	0.921	1.18E-29	Atp6v1g1	MuC_Agedup	MuC
1.35E-47	0.9239783	0.974	0.994	3.24E-43	Rpl18a	MuC_Agedup	MuC
6.11E-29	0.9220934	0.845	0.918	1.47E-24	Gabarap	MuC_Agedup	MuC
1.46E-14	0.9214584	0.688	0.699	3.51E-10	Arl1	MuC_Agedup	MuC
1.83E-12	0.9169297	0.656	0.62	4.39E-08	Serpinb11	MuC_Agedup	MuC
4.87E-21	0.9118626	0.799	0.835	1.17E-16	Cope	MuC_Agedup	MuC
4.05E-29	0.9097881	0.828	0.835	9.72E-25	Atp5g3	MuC_Agedup	MuC
7.24E-22	0.9086219	0.668	0.465	1.74E-17	Pdia4	MuC_Agedup	MuC
1.10E-23	0.9074359	0.734	0.585	2.63E-19	Rab3d	MuC_Agedup	MuC
5.83E-15	0.9063152	0.605	0.453	1.40E-10	Tsc22d1	MuC_Agedup	MuC
1.26E-32	0.9039389	0.817	0.734	3.03E-28	Mrps21	MuC_Agedup	MuC
4.18E-26	0.8990892	0.842	0.851	1.00E-21	Nme1	MuC_Agedup	MuC
1.07E-20	0.8947112	0.791	0.744	2.58E-16	Rpn2	MuC_Agedup	MuC
2.01E-22	0.8941863	0.731	0.642	4.83E-18	Sar1a	MuC_Agedup	MuC
2.09E-16	0.8914025	0.527	0.297	5.01E-12	Anxa2	MuC_Agedup	MuC
6.34E-26	0.8911684	0.794	0.766	1.52E-21	Psmb6	MuC_Agedup	MuC
1.78E-28	0.8911547	0.808	0.826	4.26E-24	Ndufa11	MuC_Agedup	MuC
4.91E-25	0.8883165	0.719	0.535	1.18E-20	Copb2	MuC_Agedup	MuC
2.76E-19	0.8865488	0.736	0.674	6.61E-15	Atp2a3	MuC_Agedup	MuC
1.22E-15	0.8821436	0.585	0.411	2.93E-11	Spdef	MuC_Agedup	MuC
8.63E-23	0.8817412	0.705	0.576	2.07E-18	2310039H08Rik	MuC_Agedup	MuC
6.25E-31	0.8810559	0.774	0.547	1.50E-26	Itm2c	MuC_Agedup	MuC
7.91E-27	0.8801869	0.504	0.152	1.90E-22	St3gal4	MuC_Agedup	MuC
2.47E-20	0.8799587	0.705	0.57	5.92E-16	Dcxr	MuC_Agedup	MuC
3.17E-42	0.8791923	0.877	0.927	7.60E-38	Ndufb9	MuC_Agedup	MuC
3.74E-27	0.8753267	0.673	0.411	8.96E-23	Shisa5	MuC_Agedup	MuC
1.45E-20	0.872409	0.837	0.873	3.47E-16	Gnas	MuC_Agedup	MuC
5.35E-18	0.8720277	0.777	0.766	1.28E-13	Cmpk1	MuC_Agedup	MuC

1.03E-20	0.8713561	0.708	0.56	2.46E-16	S100a1	MuC_Agedup	MuC
2.93E-26	0.8702125	0.751	0.579	7.02E-22	Cops9	MuC_Agedup	MuC
1.58E-26	0.8690477	0.802	0.772	3.80E-22	Nop10	MuC_Agedup	MuC
2.03E-35	0.8679943	0.92	0.956	4.88E-31	Cd24a	MuC_Agedup	MuC
3.31E-28	0.8666258	0.765	0.633	7.95E-24	Cycs	MuC_Agedup	MuC
1.57E-20	0.8654236	0.768	0.788	3.77E-16	Rab2a	MuC_Agedup	MuC
2.18E-27	0.8643934	0.825	0.854	5.23E-23	Minos1	MuC_Agedup	MuC
2.26E-27	0.8636544	0.868	0.93	5.42E-23	Uqcrb	MuC_Agedup	MuC
8.50E-26	0.8632934	0.751	0.611	2.04E-21	Sec23b	MuC_Agedup	MuC
8.90E-22	0.8627546	0.771	0.759	2.13E-17	Ufm1	MuC_Agedup	MuC
9.68E-20	0.8617108	0.731	0.646	2.32E-15	Higd1a	MuC_Agedup	MuC
2.32E-42	0.8613006	0.989	0.994	5.56E-38	Rps27a	MuC_Agedup	MuC
4.09E-26	0.8602326	0.779	0.684	9.81E-22	Rex1bd	MuC_Agedup	MuC
1.16E-26	0.8600875	0.762	0.649	2.78E-22	Nenf	MuC_Agedup	MuC
2.92E-32	0.8584113	0.364	0	7.01E-28	Osr2	MuC_Agedup	MuC
2.76E-22	0.8579759	0.312	0.028	6.63E-18	1600014C10Rik	MuC_Agedup	MuC
1.56E-25	0.8569182	0.762	0.633	3.74E-21	Surf4	MuC_Agedup	MuC
1.35E-30	0.8549638	0.808	0.851	3.24E-26	Creg1	MuC_Agedup	MuC
3.80E-34	0.8547144	0.854	0.918	9.12E-30	Edf1	MuC_Agedup	MuC
4.12E-20	0.8527396	0.765	0.741	9.88E-16	Ddost	MuC_Agedup	MuC
1.65E-13	0.8517717	0.453	0.218	3.96E-09	Cyr61	MuC_Agedup	MuC
2.99E-20	0.8515747	0.736	0.671	7.18E-16	Rnf7	MuC_Agedup	MuC
2.95E-26	0.8506403	0.828	0.851	7.07E-22	Arf1	MuC_Agedup	MuC
3.01E-21	0.8504961	0.802	0.839	7.21E-17	Lamp1	MuC_Agedup	MuC
5.58E-21	0.8495954	0.788	0.737	1.34E-16	Oat	MuC_Agedup	MuC
2.87E-26	0.8469763	0.711	0.497	6.89E-22	Churc1	MuC_Agedup	MuC
5.51E-25	0.8460562	0.808	0.722	1.32E-20	Copz2	MuC_Agedup	MuC
2.96E-21	0.8430787	0.55	0.291	7.09E-17	Cracr2a	MuC_Agedup	MuC
2.45E-35	0.8415093	0.868	0.915	5.87E-31	Atp5j	MuC_Agedup	MuC
9.10E-16	0.8387473	0.768	0.674	2.18E-11	B2m	MuC_Agedup	MuC
1.22E-38	0.8384879	1	1	2.93E-34	mt-Co1	MuC_Agedup	MuC
9.82E-29	0.8376325	0.645	0.32	2.35E-24	Slc39a11	MuC_Agedup	MuC
2.56E-20	0.837096	0.716	0.592	6.14E-16	Mlph	MuC_Agedup	MuC
6.73E-26	0.8368183	0.854	0.905	1.61E-21	Selenos	MuC_Agedup	MuC
2.55E-24	0.8354692	0.765	0.655	6.11E-20	Ndufa5	MuC_Agedup	MuC
4.86E-29	0.8352571	0.926	0.975	1.17E-24	Ppia	MuC_Agedup	MuC
2.41E-29	0.8346097	0.797	0.712	5.78E-25	Mrpl57	MuC_Agedup	MuC
1.43E-21	0.8343368	0.751	0.652	3.43E-17	Tmem14c	MuC_Agedup	MuC
1.14E-24	0.8338897	0.88	0.937	2.74E-20	Calm1	MuC_Agedup	MuC
2.10E-27	0.8325527	0.774	0.68	5.05E-23	Anapc13	MuC_Agedup	MuC
1.44E-46	0.8299134	0.977	1	3.46E-42	Rps23	MuC_Agedup	MuC
1.60E-19	0.8296963	0.676	0.462	3.84E-15	Tspan1	MuC_Agedup	MuC
8.04E-15	0.8286166	0.814	0.848	1.93E-10	Calm2	MuC_Agedup	MuC
6.96E-27	0.8275816	0.88	0.959	1.67E-22	Itm2b	MuC_Agedup	MuC
5.09E-23	0.8269749	0.817	0.82	1.22E-18	Gpx1	MuC_Agedup	MuC
2.17E-13	0.8265913	0.814	0.848	5.20E-09	Arf4	MuC_Agedup	MuC
2.27E-43	0.8243862	0.51	0.032	5.45E-39	F5	MuC_Agedup	MuC
3.01E-22	0.8238936	0.782	0.734	7.22E-18	1810037I17Rik	MuC_Agedup	MuC
9.22E-26	0.8238795	0.88	0.934	2.21E-21	Dbi	MuC_Agedup	MuC
9.84E-18	0.8196386	0.854	0.927	2.36E-13	Prdx1	MuC_Agedup	MuC
5.11E-35	0.8195846	0.433	0.028	1.23E-30	Atp2c2	MuC_Agedup	MuC
1.38E-21	0.8179934	0.702	0.535	3.31E-17	Cbx3	MuC_Agedup	MuC
2.31E-24	0.817371	0.759	0.674	5.54E-20	Tma7	MuC_Agedup	MuC
5.01E-17	0.8155204	0.794	0.886	1.20E-12	Selenof	MuC_Agedup	MuC
6.99E-24	0.8146583	0.576	0.275	1.68E-19	C1galt1	MuC_Agedup	MuC
2.03E-36	0.8145433	0.951	0.968	4.86E-32	Rps7	MuC_Agedup	MuC
9.57E-26	0.812711	0.774	0.734	2.29E-21	Higd2a	MuC_Agedup	MuC
2.19E-21	0.8121931	0.719	0.56	5.26E-17	Prom1	MuC_Agedup	MuC
2.31E-27	0.8119738	0.814	0.848	5.55E-23	Ndufb7	MuC_Agedup	MuC

2.58E-16	0.8110143	0.84	0.934	6.19E-12	Txn1	MuC_Agedup	MuC
1.01E-24	0.8084817	0.748	0.642	2.42E-20	Ier3ip1	MuC_Agedup	MuC
2.08E-20	0.8067435	0.794	0.826	4.99E-16	Rbx1	MuC_Agedup	MuC
3.39E-18	0.8062772	0.748	0.696	8.14E-14	Tpd52	MuC_Agedup	MuC
1.19E-26	0.8058508	0.739	0.627	2.85E-22	Mrps28	MuC_Agedup	MuC
3.57E-22	0.804154	0.756	0.731	8.56E-18	Dnajc19	MuC_Agedup	MuC
5.07E-19	0.8037597	0.782	0.851	1.22E-14	Atp6v0b	MuC_Agedup	MuC
7.08E-28	0.8003253	0.436	0.082	1.70E-23	Tcea3	MuC_Agedup	MuC
1.79E-19	0.7993429	0.567	0.323	4.30E-15	Foxa1	MuC_Agedup	MuC
3.40E-18	0.7988145	0.874	0.934	8.14E-14	S100a11	MuC_Agedup	MuC
1.16E-10	0.7961039	0.361	0.158	2.77E-06	Ifi27l2a	MuC_Agedup	MuC
1.43E-35	0.7954981	0.957	0.972	3.42E-31	Rpl6	MuC_Agedup	MuC
1.35E-17	0.7947893	0.745	0.725	3.23E-13	Nans	MuC_Agedup	MuC
1.30E-14	0.7944113	0.719	0.56	3.12E-10	Insig1	MuC_Agedup	MuC
1.92E-21	0.7944031	0.702	0.538	4.61E-17	Sec31a	MuC_Agedup	MuC
4.73E-19	0.794076	0.656	0.459	1.14E-14	Gne	MuC_Agedup	MuC
1.25E-39	0.7935905	0.971	0.981	2.99E-35	Rps19	MuC_Agedup	MuC
2.01E-29	0.7935158	0.883	0.93	4.83E-25	Serp1	MuC_Agedup	MuC
2.49E-24	0.7891432	0.814	0.88	5.97E-20	Timm13	MuC_Agedup	MuC
1.27E-17	0.7890385	0.53	0.31	3.05E-13	Slc26a2	MuC_Agedup	MuC
2.50E-29	0.7887767	0.971	0.981	6.00E-25	Tmsb4x	MuC_Agedup	MuC
8.84E-25	0.7877406	0.885	0.94	2.12E-20	Myl6	MuC_Agedup	MuC
3.15E-39	0.7873774	0.974	0.965	7.55E-35	Rps13	MuC_Agedup	MuC
9.90E-19	0.7865599	0.619	0.367	2.37E-14	Txnip	MuC_Agedup	MuC
3.20E-22	0.782469	0.731	0.63	7.67E-18	Chchd1	MuC_Agedup	MuC
3.62E-25	0.7822851	0.731	0.535	8.69E-21	Slc39a7	MuC_Agedup	MuC
5.78E-20	0.781051	0.759	0.639	1.39E-15	Ctnnb1	MuC_Agedup	MuC
4.33E-13	0.7789463	0.768	0.842	1.04E-08	Psma7	MuC_Agedup	MuC
7.08E-40	0.7756894	0.986	0.991	1.70E-35	Rpl9	MuC_Agedup	MuC
6.94E-23	0.7720391	0.894	0.991	1.66E-18	Cldn10	MuC_Agedup	MuC
4.03E-20	0.7710298	0.699	0.566	9.66E-16	Syngr2	MuC_Agedup	MuC
2.33E-22	0.7699874	0.848	0.816	5.60E-18	Cox7b	MuC_Agedup	MuC
7.26E-15	0.7677803	0.175	0	1.74E-10	Gm21718	MuC_Agedup	MuC
2.09E-20	0.7663768	0.616	0.402	5.01E-16	Cib1	MuC_Agedup	MuC
2.36E-27	0.7659382	0.857	0.934	5.65E-23	Hint1	MuC_Agedup	MuC
1.16E-21	0.7621346	0.648	0.427	2.78E-17	Slc44a4	MuC_Agedup	MuC
3.77E-40	0.7616238	0.974	0.997	9.04E-36	Rpl19	MuC_Agedup	MuC
2.66E-18	0.7616175	0.745	0.734	6.37E-14	Eny2	MuC_Agedup	MuC
1.95E-22	0.7607301	0.713	0.516	4.69E-18	Jpt1	MuC_Agedup	MuC
6.88E-13	0.7602152	0.352	0.12	1.65E-08	Thbs1	MuC_Agedup	MuC
1.11E-18	0.7596074	0.716	0.649	2.66E-14	Timm10b	MuC_Agedup	MuC
2.29E-25	0.7586607	0.613	0.282	5.49E-21	Cmtm8	MuC_Agedup	MuC
3.76E-23	0.7574633	0.842	0.797	9.02E-19	Selenow	MuC_Agedup	MuC
1.13E-27	0.7553787	0.556	0.209	2.72E-23	Retreg1	MuC_Agedup	MuC
2.95E-26	0.7549969	0.831	0.927	7.07E-22	Ndufa13	MuC_Agedup	MuC
3.41E-20	0.7549666	0.685	0.554	8.18E-16	Man2a1	MuC_Agedup	MuC
2.86E-17	0.7533943	0.765	0.696	6.85E-13	Kif5b	MuC_Agedup	MuC
7.54E-40	0.7520619	0.968	0.991	1.81E-35	Rpl35a	MuC_Agedup	MuC
7.13E-19	0.7517112	0.754	0.642	1.71E-14	Atp6v0e	MuC_Agedup	MuC
7.40E-27	0.7507372	0.415	0.073	1.77E-22	Pf4	MuC_Agedup	MuC
4.98E-21	0.7502701	0.774	0.709	1.19E-16	Lamtor4	MuC_Agedup	MuC
2.64E-14	0.749674	0.794	0.769	6.33E-10	Ifitm3	MuC_Agedup	MuC
1.98E-24	0.7495086	0.874	0.899	4.74E-20	Slc25a3	MuC_Agedup	MuC
8.14E-20	0.7490394	0.768	0.744	1.95E-15	Srp14	MuC_Agedup	MuC
6.06E-35	0.7480842	0.905	0.962	1.45E-30	H2afj	MuC_Agedup	MuC
1.19E-20	0.7475833	0.751	0.696	2.84E-16	Tcf25	MuC_Agedup	MuC
5.04E-18	0.7461137	0.788	0.82	1.21E-13	H13	MuC_Agedup	MuC
4.60E-22	0.7458301	0.811	0.797	1.10E-17	Aes	MuC_Agedup	MuC
1.74E-23	0.7421317	0.616	0.329	4.17E-19	Chchd7	MuC_Agedup	MuC

6.67E-35	0.7418108	0.39	0	1.60E-30	Bpifb6	MuC_Agedup	MuC
4.13E-18	0.7413124	0.857	0.886	9.90E-14	Dstn	MuC_Agedup	MuC
1.06E-12	0.7411081	0.705	0.655	2.53E-08	S100a16	MuC_Agedup	MuC
8.61E-19	0.7410826	0.831	0.883	2.07E-14	Tmed9	MuC_Agedup	MuC
4.31E-20	0.7407758	0.734	0.646	1.03E-15	Mrpl54	MuC_Agedup	MuC
4.68E-30	0.7406776	0.55	0.174	1.12E-25	Txndc12	MuC_Agedup	MuC
1.27E-27	0.7400723	0.547	0.196	3.05E-23	Gale	MuC_Agedup	MuC
3.83E-21	0.7377658	0.771	0.703	9.19E-17	Taf10	MuC_Agedup	MuC
6.93E-29	0.7374755	0.493	0.127	1.66E-24	Trp53inp2	MuC_Agedup	MuC
2.37E-34	0.7346696	0.951	0.987	5.67E-30	Rps11	MuC_Agedup	MuC
5.37E-21	0.7337803	0.765	0.747	1.29E-16	Ndufb4	MuC_Agedup	MuC
2.60E-31	0.733392	0.596	0.203	6.24E-27	Ccl28	MuC_Agedup	MuC
1.65E-19	0.7322049	0.771	0.766	3.95E-15	Ddrgk1	MuC_Agedup	MuC
2.02E-19	0.7316822	0.527	0.225	4.83E-15	Abhd2	MuC_Agedup	MuC
1.97E-21	0.7315158	0.665	0.449	4.73E-17	Ddb1	MuC_Agedup	MuC
1.08E-19	0.730784	0.702	0.525	2.58E-15	Cd2ap	MuC_Agedup	MuC
6.57E-20	0.7300332	0.802	0.807	1.57E-15	Ssr3	MuC_Agedup	MuC
3.25E-18	0.7296465	0.779	0.741	7.79E-14	Snrpe	MuC_Agedup	MuC
5.89E-21	0.7296461	0.688	0.503	1.41E-16	Tvp23b	MuC_Agedup	MuC
7.55E-27	0.7292759	0.825	0.889	1.81E-22	Ndufb11	MuC_Agedup	MuC
1.06E-15	0.7287094	0.731	0.674	2.53E-11	Psmb4	MuC_Agedup	MuC
1.41E-27	0.7282192	0.659	0.358	3.38E-23	Rab15	MuC_Agedup	MuC
2.66E-19	0.7279671	0.67	0.475	6.39E-15	Rsrp1	MuC_Agedup	MuC
1.83E-18	0.7276826	0.519	0.266	4.39E-14	Ckap4	MuC_Agedup	MuC
7.61E-37	0.7270495	0.974	1	1.83E-32	Fau	MuC_Agedup	MuC
1.98E-18	0.7245431	0.725	0.627	4.75E-14	Trappc6a	MuC_Agedup	MuC
3.17E-14	0.7239473	0.607	0.456	7.60E-10	5330417C22Rik	MuC_Agedup	MuC
5.92E-19	0.7221169	0.67	0.503	1.42E-14	Capns1	MuC_Agedup	MuC
7.16E-19	0.7209533	0.708	0.642	1.72E-14	Coa3	MuC_Agedup	MuC
1.69E-23	0.7207245	0.648	0.415	4.06E-19	Naxe	MuC_Agedup	MuC
8.60E-20	0.7205999	0.742	0.661	2.06E-15	Hsbp1	MuC_Agedup	MuC
6.57E-28	0.7205632	0.934	0.984	1.58E-23	Eif1	MuC_Agedup	MuC
7.78E-20	0.7197915	0.736	0.665	1.87E-15	Mpc1	MuC_Agedup	MuC
7.71E-34	0.718907	0.923	0.962	1.85E-29	Rps27l	MuC_Agedup	MuC
1.15E-20	0.7170642	0.599	0.339	2.77E-16	Plscr1	MuC_Agedup	MuC
1.16E-22	0.7158546	0.711	0.57	2.78E-18	Asph	MuC_Agedup	MuC
3.20E-20	0.7155824	0.693	0.557	7.68E-16	Sap18	MuC_Agedup	MuC
6.08E-22	0.7132225	0.808	0.829	1.46E-17	Ndufs6	MuC_Agedup	MuC
5.01E-18	0.7127385	0.86	0.842	1.20E-13	Dynll1	MuC_Agedup	MuC
3.02E-25	0.7114655	0.63	0.358	7.23E-21	Pofut2	MuC_Agedup	MuC
2.76E-20	0.7112606	0.774	0.75	6.63E-16	Mrps14	MuC_Agedup	MuC
1.22E-19	0.7087916	0.765	0.715	2.92E-15	Nedd4	MuC_Agedup	MuC
1.56E-19	0.7085842	0.573	0.31	3.75E-15	Sim2	MuC_Agedup	MuC
3.34E-20	0.7085264	0.648	0.424	8.01E-16	Itgb1	MuC_Agedup	MuC
9.62E-20	0.7081501	0.779	0.75	2.31E-15	Swi5	MuC_Agedup	MuC
1.10E-17	0.7080606	0.215	0.003	2.63E-13	Edn2	MuC_Agedup	MuC
2.18E-16	0.7070608	0.756	0.728	5.23E-12	Akr1a1	MuC_Agedup	MuC
6.02E-18	0.7062204	0.668	0.487	1.44E-13	Ptp4a2	MuC_Agedup	MuC
7.40E-20	0.7060277	0.785	0.731	1.77E-15	Aldoa	MuC_Agedup	MuC
1.62E-32	0.7058079	0.481	0.082	3.89E-28	Wipi1	MuC_Agedup	MuC
5.33E-31	0.7039322	0.943	0.978	1.28E-26	Rps5	MuC_Agedup	MuC
2.87E-19	0.7036145	0.814	0.804	6.89E-15	Tmem238	MuC_Agedup	MuC
2.38E-20	0.7026269	0.814	0.842	5.70E-16	Ndufb5	MuC_Agedup	MuC
1.11E-20	0.7022401	0.805	0.785	2.65E-16	Pomp	MuC_Agedup	MuC
4.90E-21	0.70169	0.834	0.934	1.18E-16	Pfdn5	MuC_Agedup	MuC
5.77E-24	0.7008628	0.668	0.408	1.38E-19	Twf1	MuC_Agedup	MuC
2.66E-14	0.7001086	0.682	0.535	6.37E-10	Ceacam1	MuC_Agedup	MuC
3.28E-20	0.6992822	0.748	0.68	7.85E-16	Son	MuC_Agedup	MuC
6.06E-21	0.6991204	0.699	0.554	1.45E-16	Naa38	MuC_Agedup	MuC

2.34E-15	0.698681	0.607	0.44	5.62E-11	Ppa1	MuC_Agedup	MuC
1.37E-17	0.6980766	0.765	0.794	3.29E-13	Ndufs7	MuC_Agedup	MuC
1.59E-34	0.6974768	0.401	0.009	3.82E-30	Smoc2	MuC_Agedup	MuC
1.49E-14	0.6965918	0.665	0.525	3.57E-10	Sppl2a	MuC_Agedup	MuC
2.80E-10	0.6963469	0.745	0.778	6.72E-06	Pdia6	MuC_Agedup	MuC
6.21E-21	0.6953345	0.756	0.649	1.49E-16	Cox14	MuC_Agedup	MuC
2.58E-31	0.6943274	0.453	0.07	6.19E-27	Enho	MuC_Agedup	MuC
7.60E-21	0.6941844	0.579	0.297	1.82E-16	Rab27b	MuC_Agedup	MuC
1.02E-32	0.6934593	0.453	0.057	2.45E-28	Lbp	MuC_Agedup	MuC
1.68E-34	0.6929047	0.593	0.18	4.04E-30	Gstp1	MuC_Agedup	MuC
1.11E-19	0.6914454	0.794	0.851	2.67E-15	2410015M20Rik	MuC_Agedup	MuC
6.68E-17	0.6909981	0.576	0.358	1.60E-12	Timp2	MuC_Agedup	MuC
1.33E-19	0.689557	0.716	0.563	3.19E-15	Clstn1	MuC_Agedup	MuC
9.90E-29	0.6893333	0.636	0.291	2.38E-24	Lasp1	MuC_Agedup	MuC
1.97E-26	0.6884488	0.613	0.269	4.73E-22	Dnm2	MuC_Agedup	MuC
1.77E-17	0.6879581	0.699	0.595	4.25E-13	Gorasp2	MuC_Agedup	MuC
2.48E-27	0.6875302	0.619	0.297	5.95E-23	Setd5	MuC_Agedup	MuC
8.80E-14	0.6864963	0.691	0.674	2.11E-09	Gabarapl2	MuC_Agedup	MuC
9.13E-09	0.6846431	0.728	0.665	0.000219	Nr4a1	MuC_Agedup	MuC
1.33E-11	0.6839467	0.751	0.788	3.19E-07	Furin	MuC_Agedup	MuC
5.73E-12	0.6838316	0.779	0.832	1.37E-07	Ssr2	MuC_Agedup	MuC
1.99E-24	0.6832372	0.295	0.006	4.78E-20	Gm10260	MuC_Agedup	MuC
1.17E-17	0.6824326	0.742	0.731	2.82E-13	Mia2	MuC_Agedup	MuC
3.42E-21	0.6822237	0.673	0.481	8.20E-17	Cox17	MuC_Agedup	MuC
2.51E-27	0.6819982	0.923	0.953	6.01E-23	Rpl7a	MuC_Agedup	MuC
1.56E-14	0.681847	0.762	0.725	3.73E-10	Vcp	MuC_Agedup	MuC
7.93E-29	0.6818221	0.533	0.165	1.90E-24	Acsl3	MuC_Agedup	MuC
3.57E-17	0.6790833	0.739	0.674	8.57E-13	Mrpl33	MuC_Agedup	MuC
6.80E-25	0.6780772	0.86	0.937	1.63E-20	Map1lc3b	MuC_Agedup	MuC
8.42E-23	0.6774672	0.616	0.361	2.02E-18	Psme2	MuC_Agedup	MuC
1.54E-17	0.6769332	0.768	0.699	3.69E-13	Rab1a	MuC_Agedup	MuC
7.56E-17	0.6767098	0.725	0.617	1.81E-12	Fis1	MuC_Agedup	MuC
6.98E-20	0.6761882	0.702	0.598	1.67E-15	Mrps33	MuC_Agedup	MuC
2.21E-13	0.6755028	0.716	0.671	5.31E-09	Tm9sf2	MuC_Agedup	MuC
3.78E-24	0.6753819	0.607	0.294	9.07E-20	Akt1	MuC_Agedup	MuC
1.11E-17	0.6744829	0.728	0.693	2.67E-13	Arpp19	MuC_Agedup	MuC
5.05E-20	0.6740087	0.562	0.313	1.21E-15	Trmt1	MuC_Agedup	MuC
8.28E-24	0.6732546	0.862	0.911	1.99E-19	Pabpc1	MuC_Agedup	MuC
1.75E-16	0.6724378	0.716	0.595	4.19E-12	Rbm47	MuC_Agedup	MuC
1.90E-10	0.6722603	0.702	0.709	4.56E-06	Psmb3	MuC_Agedup	MuC
2.46E-19	0.6709873	0.742	0.75	5.90E-15	Tmem160	MuC_Agedup	MuC
1.34E-12	0.6707012	0.696	0.646	3.22E-08	Bcat2	MuC_Agedup	MuC
2.45E-26	0.669294	0.407	0.066	5.88E-22	Anxa3	MuC_Agedup	MuC
2.72E-13	0.6661343	0.619	0.494	6.53E-09	Slc38a1	MuC_Agedup	MuC
2.31E-16	0.6654881	0.705	0.627	5.55E-12	Srp19	MuC_Agedup	MuC
7.40E-21	0.6631247	0.791	0.81	1.77E-16	Ndufb3	MuC_Agedup	MuC
4.31E-19	0.6626602	0.768	0.68	1.03E-14	Zfp706	MuC_Agedup	MuC
3.64E-18	0.662039	0.788	0.81	8.74E-14	1110008F13Rik	MuC_Agedup	MuC
9.43E-17	0.6620287	0.777	0.823	2.26E-12	Ndufs5	MuC_Agedup	MuC
2.31E-15	0.6607569	0.696	0.585	5.53E-11	Yipf3	MuC_Agedup	MuC
1.04E-17	0.6598276	0.751	0.747	2.49E-13	Jtb	MuC_Agedup	MuC
8.88E-19	0.6595148	0.88	0.873	2.13E-14	Rpl31	MuC_Agedup	MuC
1.27E-14	0.6568973	0.754	0.794	3.04E-10	Psmb5	MuC_Agedup	MuC
3.40E-22	0.6568156	0.607	0.335	8.16E-18	Mansc1	MuC_Agedup	MuC
1.02E-16	0.6550158	0.544	0.323	2.45E-12	Kdelr3	MuC_Agedup	MuC
5.24E-23	0.654564	0.679	0.44	1.26E-18	Sdf2	MuC_Agedup	MuC
8.97E-25	0.65412	0.96	0.984	2.15E-20	Rps26	MuC_Agedup	MuC
1.73E-18	0.6535565	0.799	0.82	4.15E-14	Ndufb10	MuC_Agedup	MuC
1.43E-19	0.6517465	0.84	0.902	3.44E-15	Atp5d	MuC_Agedup	MuC

1.45E-20	0.6516082	0.67	0.487	3.47E-16	Clint1	MuC_Agedup	MuC
1.17E-24	0.6513586	0.372	0.054	2.80E-20	Vwf	MuC_Agedup	MuC
3.80E-19	0.6502844	0.585	0.316	9.11E-15	Slc39a1	MuC_Agedup	MuC
9.02E-18	0.6498847	0.814	0.861	2.16E-13	Eif4g2	MuC_Agedup	MuC
1.25E-19	0.649361	0.676	0.475	2.99E-15	Uso1	MuC_Agedup	MuC
3.13E-20	0.648837	0.748	0.636	7.51E-16	Spcs3	MuC_Agedup	MuC
4.91E-17	0.6452498	0.685	0.544	1.18E-12	Mrpl14	MuC_Agedup	MuC
6.21E-21	0.6450428	0.562	0.282	1.49E-16	Rell1	MuC_Agedup	MuC
4.13E-25	0.6449096	0.444	0.111	9.89E-21	Scmh1	MuC_Agedup	MuC
5.69E-09	0.6448395	0.619	0.547	0.000136	Ptprf	MuC_Agedup	MuC
7.58E-13	0.644553	0.811	0.813	1.82E-08	Srrm2	MuC_Agedup	MuC
9.97E-17	0.6440895	0.711	0.611	2.39E-12	Cuta	MuC_Agedup	MuC
8.70E-15	0.6440833	0.699	0.62	2.09E-10	BC031181	MuC_Agedup	MuC
2.15E-23	0.6434599	0.616	0.345	5.15E-19	Erh	MuC_Agedup	MuC
5.16E-14	0.642138	0.642	0.516	1.24E-09	Lrrc59	MuC_Agedup	MuC
1.99E-13	0.6412872	0.665	0.544	4.76E-09	Os9	MuC_Agedup	MuC
1.22E-15	0.6410607	0.751	0.797	2.92E-11	Ndufab1	MuC_Agedup	MuC
7.72E-17	0.640704	0.682	0.522	1.85E-12	Cltc	MuC_Agedup	MuC
4.24E-10	0.6404745	0.765	0.804	1.02E-05	Tmem176b	MuC_Agedup	MuC
6.60E-18	0.6393254	0.934	0.981	1.58E-13	Cd9	MuC_Agedup	MuC
4.17E-23	0.6392421	1	1	9.99E-19	mt-Nd4	MuC_Agedup	MuC
7.59E-13	0.6379456	0.648	0.532	1.82E-08	Copb1	MuC_Agedup	MuC
6.14E-31	0.6379376	0.375	0.013	1.47E-26	Cib3	MuC_Agedup	MuC
1.34E-16	0.637554	0.785	0.81	3.21E-12	Ndufa6	MuC_Agedup	MuC
7.90E-22	0.6349934	0.888	0.93	1.89E-17	Rpl36a	MuC_Agedup	MuC
6.02E-17	0.6348285	0.653	0.491	1.44E-12	Hint3	MuC_Agedup	MuC
9.55E-12	0.6344314	0.639	0.557	2.29E-07	Yipf6	MuC_Agedup	MuC
5.15E-22	0.6328088	0.596	0.329	1.24E-17	Pet100	MuC_Agedup	MuC
1.43E-16	0.6325336	0.782	0.778	3.42E-12	Atp6v1f	MuC_Agedup	MuC
3.89E-16	0.6311994	0.605	0.418	9.33E-12	Sdf4	MuC_Agedup	MuC
3.48E-12	0.631177	0.659	0.566	8.35E-08	Sdhb	MuC_Agedup	MuC
2.75E-27	0.6304526	0.413	0.063	6.59E-23	Crlf1	MuC_Agedup	MuC
7.50E-18	0.6295767	0.734	0.646	1.80E-13	Eif4g1	MuC_Agedup	MuC
1.46E-15	0.6290702	0.759	0.744	3.49E-11	Ndufa8	MuC_Agedup	MuC
3.16E-13	0.6282129	0.774	0.728	7.58E-09	Ifi27	MuC_Agedup	MuC
3.28E-18	0.6280276	0.691	0.528	7.87E-14	Copz1	MuC_Agedup	MuC
1.33E-20	0.6272236	0.519	0.234	3.19E-16	Creb3l4	MuC_Agedup	MuC
3.82E-12	0.6266084	0.716	0.674	9.16E-08	Gde1	MuC_Agedup	MuC
1.21E-09	0.6231641	0.59	0.472	2.90E-05	Tst	MuC_Agedup	MuC
3.25E-11	0.6215578	0.805	0.829	7.80E-07	Slc25a5	MuC_Agedup	MuC
1.66E-22	0.6210388	0.413	0.104	3.98E-18	Slc12a8	MuC_Agedup	MuC
5.67E-13	0.6208155	0.708	0.671	1.36E-08	Uqcrfs1	MuC_Agedup	MuC
4.95E-22	0.6205047	0.656	0.408	1.19E-17	Gns	MuC_Agedup	MuC
6.35E-31	0.6200789	0.367	0.009	1.52E-26	Liph	MuC_Agedup	MuC
3.63E-22	0.619948	0.57	0.275	8.70E-18	Tmem9b	MuC_Agedup	MuC
7.50E-14	0.6197785	0.587	0.443	1.80E-09	Appl2	MuC_Agedup	MuC
6.07E-11	0.618313	0.653	0.598	1.45E-06	Slc35b1	MuC_Agedup	MuC
7.90E-12	0.6179937	0.845	0.93	1.89E-07	P4hb	MuC_Agedup	MuC
1.49E-13	0.617209	0.605	0.396	3.56E-09	Lars2	MuC_Agedup	MuC
2.80E-21	0.6165942	0.799	0.883	6.71E-17	Ndufc2	MuC_Agedup	MuC
4.50E-16	0.6157829	0.685	0.595	1.08E-11	Prelid1	MuC_Agedup	MuC
1.95E-14	0.6150793	0.673	0.5	4.68E-10	Cdh1	MuC_Agedup	MuC
3.99E-19	0.6150683	0.676	0.535	9.57E-15	Timm17b	MuC_Agedup	MuC
3.34E-20	0.6136825	0.854	0.921	8.02E-16	Tram1	MuC_Agedup	MuC
2.12E-11	0.6134579	0.51	0.345	5.08E-07	Ezr	MuC_Agedup	MuC
9.14E-15	0.6116468	0.67	0.519	2.19E-10	Erp44	MuC_Agedup	MuC
3.99E-10	0.6101619	0.636	0.601	9.57E-06	Slc50a1	MuC_Agedup	MuC
2.21E-11	0.6091291	0.754	0.82	5.30E-07	Ifitm2	MuC_Agedup	MuC
3.88E-13	0.6086104	0.593	0.449	9.31E-09	Fndc3b	MuC_Agedup	MuC

1.35E-12	0.6083817	0.682	0.617	3.23E-08	Chmp4b	MuC_Agedup	MuC
1.84E-11	0.6079015	0.731	0.741	4.41E-07	Psma2	MuC_Agedup	MuC
2.67E-16	0.6066207	0.708	0.595	6.40E-12	Ergic1	MuC_Agedup	MuC
3.14E-14	0.6060043	0.59	0.43	7.54E-10	Tspan3	MuC_Agedup	MuC
8.55E-12	0.6045176	0.754	0.737	2.05E-07	Nxf1	MuC_Agedup	MuC
1.37E-15	0.6043781	0.622	0.484	3.29E-11	Iscu	MuC_Agedup	MuC
2.09E-24	0.6041463	0.931	0.959	5.00E-20	Rpl22	MuC_Agedup	MuC
7.00E-13	0.603796	0.722	0.655	1.68E-08	Clptm1l	MuC_Agedup	MuC
6.54E-21	0.6031944	0.596	0.32	1.57E-16	Fam32a	MuC_Agedup	MuC
2.03E-22	0.60257	0.582	0.272	4.87E-18	Por	MuC_Agedup	MuC
1.38E-13	0.6024115	0.619	0.472	3.30E-09	Arpc5	MuC_Agedup	MuC
3.74E-10	0.6018625	0.785	0.775	8.98E-06	Sox9	MuC_Agedup	MuC
1.49E-12	0.6015107	0.754	0.763	3.57E-08	Tmem234	MuC_Agedup	MuC
5.68E-18	0.6013352	0.593	0.377	1.36E-13	Urah	MuC_Agedup	MuC
1.98E-12	0.6010154	0.782	0.813	4.75E-08	Psmb1	MuC_Agedup	MuC
7.17E-19	0.6009207	0.636	0.427	1.72E-14	Slc17a5	MuC_Agedup	MuC
2.94E-15	0.5998373	0.65	0.487	7.04E-11	1810058I24Rik	MuC_Agedup	MuC
9.95E-09	0.5993904	0.722	0.756	0.000239	Psma3	MuC_Agedup	MuC
3.57E-14	0.5988166	0.599	0.421	8.56E-10	Aplp2	MuC_Agedup	MuC
1.20E-24	0.5986794	0.943	0.984	2.87E-20	Rpl24	MuC_Agedup	MuC
7.18E-11	0.5979189	0.682	0.699	1.72E-06	Mrpl17	MuC_Agedup	MuC
1.28E-19	0.5975454	0.585	0.335	3.08E-15	Sec16a	MuC_Agedup	MuC
8.53E-12	0.597444	0.748	0.737	2.05E-07	Canx	MuC_Agedup	MuC
1.12E-15	0.5968424	0.831	0.877	2.68E-11	Atp5a1	MuC_Agedup	MuC
5.20E-12	0.5957775	0.777	0.807	1.25E-07	Atox1	MuC_Agedup	MuC
2.17E-13	0.5950101	0.607	0.459	5.21E-09	Fuca1	MuC_Agedup	MuC
2.09E-15	0.5942026	0.458	0.237	5.01E-11	Slc35a1	MuC_Agedup	MuC
1.52E-14	0.5940771	0.708	0.617	3.64E-10	Ndufb6	MuC_Agedup	MuC
4.04E-10	0.5940322	0.636	0.598	9.68E-06	Psmb2	MuC_Agedup	MuC
4.18E-13	0.5933097	0.693	0.627	1.00E-08	Mcfd2	MuC_Agedup	MuC
6.83E-15	0.5929375	0.61	0.443	1.64E-10	Prr15l	MuC_Agedup	MuC
9.46E-15	0.5927167	0.731	0.665	2.27E-10	Nars	MuC_Agedup	MuC
8.89E-16	0.5914848	0.556	0.339	2.13E-11	Hes6	MuC_Agedup	MuC
8.20E-14	0.5903817	0.668	0.551	1.97E-09	Pmm2	MuC_Agedup	MuC
1.70E-13	0.5901828	0.759	0.788	4.07E-09	Ift20	MuC_Agedup	MuC
1.91E-19	0.5901668	0.983	1	4.58E-15	Eef1a1	MuC_Agedup	MuC
1.37E-12	0.58962	0.748	0.703	3.29E-08	Gdi2	MuC_Agedup	MuC
2.05E-25	0.5879415	0.415	0.076	4.93E-21	Dkk3	MuC_Agedup	MuC
2.53E-13	0.5869858	0.768	0.788	6.06E-09	Mrps24	MuC_Agedup	MuC
1.40E-23	0.5869082	0.55	0.231	3.35E-19	Tmem263	MuC_Agedup	MuC
5.12E-17	0.5842942	0.513	0.272	1.23E-12	Pllp	MuC_Agedup	MuC
2.80E-10	0.58391	0.576	0.434	6.72E-06	Bhlhe40	MuC_Agedup	MuC
9.10E-19	0.5836228	0.716	0.598	2.18E-14	Timm8b	MuC_Agedup	MuC
2.14E-31	0.5824923	0.361	0.003	5.12E-27	Bcas1	MuC_Agedup	MuC
1.10E-14	0.5824263	0.791	0.832	2.64E-10	Gpx4	MuC_Agedup	MuC
2.16E-07	0.5817522	0.115	0.282	0.005174	Dcpp3	MuC_Agedup	MuC
1.84E-19	0.5773497	0.625	0.389	4.41E-15	Hid1	MuC_Agedup	MuC
1.67E-16	0.574491	0.521	0.269	4.01E-12	Klf10	MuC_Agedup	MuC
3.31E-24	0.5744363	0.499	0.165	7.95E-20	Rhoc	MuC_Agedup	MuC
2.26E-17	0.572975	0.533	0.301	5.42E-13	Nt5c	MuC_Agedup	MuC
1.33E-20	0.5720473	0.43	0.136	3.19E-16	Hilpda	MuC_Agedup	MuC
1.88E-12	0.5715345	0.596	0.446	4.51E-08	Atp6ap2	MuC_Agedup	MuC
3.52E-21	0.5709885	0.579	0.304	8.43E-17	Sh3bgrl2	MuC_Agedup	MuC
3.37E-13	0.5700766	0.685	0.573	8.09E-09	Edem2	MuC_Agedup	MuC
1.90E-25	0.5694136	0.954	0.984	4.57E-21	Rps21	MuC_Agedup	MuC
1.36E-19	0.5683065	0.487	0.206	3.25E-15	Tmem9	MuC_Agedup	MuC
1.17E-14	0.5681858	0.668	0.563	2.80E-10	Ndufs4	MuC_Agedup	MuC
1.38E-18	0.565943	0.696	0.582	3.32E-14	Bola2	MuC_Agedup	MuC
2.25E-16	0.5648052	0.636	0.478	5.39E-12	Snrpg	MuC_Agedup	MuC

5.39E-31	0.5631673	0.991	0.994	1.29E-26	Rps24	MuC_Agedup	MuC
5.26E-28	0.5629398	0.401	0.051	1.26E-23	Acp5	MuC_Agedup	MuC
5.20E-28	0.5622756	0.321	0	1.25E-23	Spink4	MuC_Agedup	MuC
6.75E-19	0.560368	0.559	0.297	1.62E-14	Ick	MuC_Agedup	MuC
3.36E-13	0.5590342	0.625	0.468	8.05E-09	Eif4ebp2	MuC_Agedup	MuC
2.87E-13	0.5588522	0.639	0.487	6.87E-09	Pcbd2	MuC_Agedup	MuC
7.84E-15	0.5581946	0.57	0.377	1.88E-10	Galnt3	MuC_Agedup	MuC
9.40E-12	0.5559169	0.57	0.411	2.25E-07	Ganab	MuC_Agedup	MuC
2.02E-13	0.5556124	0.576	0.405	4.84E-09	Tmem30a	MuC_Agedup	MuC
8.55E-12	0.5555838	0.731	0.753	2.05E-07	Mpc2	MuC_Agedup	MuC
3.75E-08	0.5549807	0.616	0.538	0.0009	Runx1	MuC_Agedup	MuC
1.42E-07	0.5534181	0.696	0.699	0.003416	Clic1	MuC_Agedup	MuC
5.60E-13	0.5531865	0.736	0.722	1.34E-08	Cstb	MuC_Agedup	MuC
5.28E-16	0.5528685	0.53	0.282	1.27E-11	Atp9a	MuC_Agedup	MuC
2.94E-16	0.5527787	0.633	0.478	7.05E-12	Med29	MuC_Agedup	MuC
1.87E-14	0.5522936	0.585	0.37	4.47E-10	Impad1	MuC_Agedup	MuC
3.20E-13	0.5517454	0.751	0.81	7.67E-09	Nedd8	MuC_Agedup	MuC
4.49E-08	0.55147	0.725	0.725	0.001076	Klf5	MuC_Agedup	MuC
5.26E-13	0.5500695	0.725	0.665	1.26E-08	Scp2	MuC_Agedup	MuC
3.25E-13	0.5496417	0.63	0.484	7.78E-09	Cant1	MuC_Agedup	MuC
3.89E-19	0.5496277	0.433	0.152	9.33E-15	Glb1	MuC_Agedup	MuC
6.58E-11	0.5488108	0.613	0.472	1.58E-06	Actr2	MuC_Agedup	MuC
2.78E-12	0.5482774	0.736	0.687	6.67E-08	Nsa2	MuC_Agedup	MuC
8.04E-14	0.5478603	0.516	0.316	1.93E-09	Creb3	MuC_Agedup	MuC
9.78E-17	0.5474318	0.628	0.392	2.34E-12	Arhgdia	MuC_Agedup	MuC
4.26E-10	0.5468942	0.67	0.639	1.02E-05	Trappc2l	MuC_Agedup	MuC
5.81E-13	0.5465793	0.659	0.532	1.39E-08	Tmc5	MuC_Agedup	MuC
2.56E-11	0.5464177	0.782	0.81	6.15E-07	Chchd10	MuC_Agedup	MuC
5.49E-15	0.5462873	0.639	0.468	1.32E-10	Hcfc1r1	MuC_Agedup	MuC
1.13E-10	0.5459205	0.708	0.665	2.71E-06	Trir	MuC_Agedup	MuC
2.12E-15	0.5458703	0.676	0.595	5.07E-11	Eloc	MuC_Agedup	MuC
7.10E-09	0.5457399	0.696	0.639	0.00017	Cfl1	MuC_Agedup	MuC
1.00E-18	0.5455915	0.688	0.516	2.41E-14	Sec63	MuC_Agedup	MuC
9.40E-11	0.5453602	0.622	0.513	2.25E-06	Akap9	MuC_Agedup	MuC
2.69E-11	0.5451368	0.65	0.579	6.44E-07	Sra1	MuC_Agedup	MuC
9.04E-17	0.5450026	0.51	0.269	2.17E-12	Hexa	MuC_Agedup	MuC
2.97E-22	0.544042	0.582	0.263	7.12E-18	Eif2ak3	MuC_Agedup	MuC
4.46E-10	0.5438453	0.665	0.604	1.07E-05	Atp6v1e1	MuC_Agedup	MuC
1.73E-10	0.5437667	0.619	0.528	4.15E-06	Sat1	MuC_Agedup	MuC
1.75E-10	0.5417201	0.771	0.788	4.20E-06	Prdx2	MuC_Agedup	MuC
3.04E-17	0.541332	0.539	0.297	7.30E-13	Mbtps1	MuC_Agedup	MuC
7.87E-17	0.5403462	0.45	0.187	1.89E-12	Fbxo32	MuC_Agedup	MuC
1.82E-19	0.5401905	0.928	0.984	4.37E-15	Rpl14	MuC_Agedup	MuC
5.71E-16	0.5387097	0.877	0.946	1.37E-11	Eef2	MuC_Agedup	MuC
2.19E-22	0.5382848	0.991	0.997	5.26E-18	Tpt1	MuC_Agedup	MuC
5.88E-12	0.5377307	0.564	0.418	1.41E-07	Pdxdc1	MuC_Agedup	MuC
1.80E-17	0.5376869	0.55	0.304	4.32E-13	Uggt1	MuC_Agedup	MuC
1.15E-16	0.5373777	0.63	0.462	2.76E-12	Pam16	MuC_Agedup	MuC
1.34E-15	0.5373243	0.607	0.402	3.22E-11	Prkcsh	MuC_Agedup	MuC
1.97E-10	0.5371857	0.725	0.712	4.72E-06	Mdh1	MuC_Agedup	MuC
7.53E-14	0.5370102	0.688	0.604	1.81E-09	Trmt112	MuC_Agedup	MuC
3.47E-23	0.5365082	0.456	0.136	8.31E-19	Pdia5	MuC_Agedup	MuC
3.92E-17	0.5348424	0.59	0.345	9.40E-13	Preb	MuC_Agedup	MuC
1.88E-09	0.5345299	0.688	0.712	4.50E-05	Fndc3a	MuC_Agedup	MuC
1.06E-11	0.5342236	0.668	0.604	2.55E-07	Emc7	MuC_Agedup	MuC
3.50E-08	0.5340041	0.688	0.712	0.00084	Serinc3	MuC_Agedup	MuC
3.80E-23	0.5338311	0.977	0.984	9.12E-19	Rps9	MuC_Agedup	MuC
2.66E-10	0.5332825	0.739	0.763	6.38E-06	Tomm5	MuC_Agedup	MuC
1.18E-13	0.5331993	0.719	0.655	2.84E-09	Mlec	MuC_Agedup	MuC

2.71E-12	0.5327672	0.579	0.421	6.50E-08	Litaf	MuC_Agedup	MuC
2.83E-15	0.5321236	0.599	0.396	6.79E-11	Edem1	MuC_Agedup	MuC
2.53E-11	0.5317894	0.659	0.592	6.07E-07	Arcn1	MuC_Agedup	MuC
3.02E-11	0.5316936	0.756	0.725	7.23E-07	Gmds	MuC_Agedup	MuC
2.46E-17	0.5303209	0.521	0.256	5.89E-13	Tapbp	MuC_Agedup	MuC
1.17E-16	0.5299699	0.461	0.209	2.82E-12	Slc38a10	MuC_Agedup	MuC
3.21E-10	0.5287518	0.771	0.851	7.70E-06	Bsg	MuC_Agedup	MuC
8.75E-16	0.528538	0.579	0.358	2.10E-11	Lrp10	MuC_Agedup	MuC
1.48E-19	0.5275174	0.481	0.193	3.54E-15	Twf2	MuC_Agedup	MuC
5.77E-14	0.5263506	0.564	0.392	1.38E-09	Malsu1	MuC_Agedup	MuC
3.92E-18	0.5262734	0.504	0.228	9.39E-14	Ttc39a	MuC_Agedup	MuC
1.94E-15	0.5261246	0.593	0.386	4.64E-11	Ccnl2	MuC_Agedup	MuC
6.97E-10	0.5247463	0.736	0.744	1.67E-05	Kdelr1	MuC_Agedup	MuC
2.17E-16	0.5246914	0.501	0.256	5.21E-12	Txndc11	MuC_Agedup	MuC
4.71E-09	0.5241317	0.768	0.82	0.000113	Rbm39	MuC_Agedup	MuC
8.35E-09	0.5234428	0.544	0.446	0.0002	Yipf1	MuC_Agedup	MuC
2.23E-09	0.5231743	0.642	0.611	5.35E-05	Ndufs8	MuC_Agedup	MuC
1.75E-13	0.5228769	0.865	0.946	4.20E-09	Spcs2	MuC_Agedup	MuC
1.65E-22	0.522615	0.45	0.136	3.95E-18	Dynlt1f	MuC_Agedup	MuC
2.68E-16	0.5215057	0.648	0.472	6.42E-12	Ppdpf	MuC_Agedup	MuC
9.00E-12	0.5212865	0.639	0.557	2.16E-07	Uqcc3	MuC_Agedup	MuC
2.93E-13	0.5212804	0.61	0.446	7.02E-09	Prrc1	MuC_Agedup	MuC
2.38E-12	0.5211322	0.596	0.459	5.71E-08	Bola1	MuC_Agedup	MuC
7.18E-14	0.5209849	0.576	0.402	1.72E-09	Tmem248	MuC_Agedup	MuC
3.21E-14	0.5201668	0.579	0.38	7.71E-10	Fam174b	MuC_Agedup	MuC
2.68E-13	0.5174062	0.822	0.905	6.42E-09	H2afz	MuC_Agedup	MuC
1.47E-16	0.5173065	0.585	0.364	3.52E-12	Hypk	MuC_Agedup	MuC
1.43E-14	0.5167715	0.65	0.525	3.42E-10	1110065P20Rik	MuC_Agedup	MuC
3.97E-08	0.5165856	0.765	0.785	0.000952	Ybx1	MuC_Agedup	MuC
6.69E-07	0.5164601	0.679	0.668	0.016051	Tpi1	MuC_Agedup	MuC
1.32E-10	0.5162593	0.811	0.848	3.16E-06	Pgls	MuC_Agedup	MuC
2.86E-11	0.5159016	0.59	0.468	6.87E-07	Pigt	MuC_Agedup	MuC
1.56E-14	0.51569	0.693	0.592	3.74E-10	Ss18l2	MuC_Agedup	MuC
2.44E-11	0.5151046	0.605	0.462	5.85E-07	Tmem183a	MuC_Agedup	MuC
6.74E-09	0.5149949	0.794	0.826	0.000162	Polr1d	MuC_Agedup	MuC
3.21E-19	0.5144032	0.928	0.975	7.70E-15	Rpl10a	MuC_Agedup	MuC
1.50E-09	0.5142793	0.722	0.712	3.59E-05	Chmp2a	MuC_Agedup	MuC
6.78E-20	0.512544	0.971	0.994	1.63E-15	Rpl27a	MuC_Agedup	MuC
1.57E-13	0.5122903	0.456	0.222	3.76E-09	Slc6a6	MuC_Agedup	MuC
3.25E-13	0.5117433	0.645	0.484	7.79E-09	Mrpl36	MuC_Agedup	MuC
3.34E-11	0.5113475	0.404	0.206	8.01E-07	Hopx	MuC_Agedup	MuC
7.89E-16	0.5111871	0.607	0.402	1.89E-11	Pin4	MuC_Agedup	MuC
9.12E-09	0.5106388	0.585	0.481	0.000219	Capzb	MuC_Agedup	MuC
2.82E-11	0.5103276	0.699	0.655	6.77E-07	Arpc1b	MuC_Agedup	MuC
6.47E-08	0.5093546	0.61	0.554	0.001552	H1f0	MuC_Agedup	MuC
6.70E-10	0.5090184	0.665	0.57	1.61E-05	Odc1	MuC_Agedup	MuC
3.30E-12	0.508824	0.65	0.497	7.90E-08	Eif3b	MuC_Agedup	MuC
4.27E-13	0.5078352	0.553	0.364	1.02E-08	Bst2	MuC_Agedup	MuC
3.69E-13	0.5069261	0.499	0.294	8.86E-09	Leprotl1	MuC_Agedup	MuC
5.79E-07	0.5044133	0.633	0.57	0.013879	Sh3glb1	MuC_Agedup	MuC
6.98E-13	0.5033607	0.573	0.399	1.67E-08	Sdhd	MuC_Agedup	MuC
8.38E-08	0.503177	0.705	0.794	0.002009	Ufc1	MuC_Agedup	MuC
4.52E-16	0.5026512	0.441	0.19	1.08E-11	Nek7	MuC_Agedup	MuC
3.86E-12	0.5020081	0.61	0.462	9.26E-08	Dazap2	MuC_Agedup	MuC
2.51E-13	0.5019999	0.648	0.506	6.02E-09	Mrpl34	MuC_Agedup	MuC
9.60E-11	0.5012892	0.656	0.566	2.30E-06	Stub1	MuC_Agedup	MuC
2.38E-20	0.5012229	0.504	0.218	5.70E-16	Smim1	MuC_Agedup	MuC
5.08E-12	0.5003479	0.479	0.275	1.22E-07	Rap1gap	MuC_Agedup	MuC
1.34E-08	0.5001397	0.731	0.734	0.000321	Rtraf	MuC_Agedup	MuC

5.31E-09	0.4994988	0.734	0.725	0.000127	Cdc42	MuC_Agedup	MuC
3.19E-13	0.499401	0.628	0.491	7.66E-09	Cisd1	MuC_Agedup	MuC
3.19E-11	0.4993935	0.504	0.358	7.66E-07	Gnpnat1	MuC_Agedup	MuC
5.96E-11	0.4989708	0.648	0.56	1.43E-06	Grpel1	MuC_Agedup	MuC
1.53E-19	0.498195	0.229	0	3.68E-15	Itln1	MuC_Agedup	MuC
1.84E-11	0.4980316	0.659	0.566	4.42E-07	Emc6	MuC_Agedup	MuC
2.55E-20	0.4976771	0.499	0.209	6.11E-16	Krtcap3	MuC_Agedup	MuC
5.00E-15	0.4976348	0.533	0.326	1.20E-10	Gnptg	MuC_Agedup	MuC
2.62E-13	0.4974069	0.857	0.956	6.28E-09	Rpl4	MuC_Agedup	MuC
1.43E-13	0.4974013	0.562	0.386	3.43E-09	Dpm2	MuC_Agedup	MuC
2.92E-32	0.4970191	0.364	0	7.01E-28	BC030870	MuC_Agedup	MuC
7.98E-12	0.4967643	0.642	0.544	1.91E-07	Commd6	MuC_Agedup	MuC
4.90E-10	0.4967639	0.797	0.87	1.18E-05	Atp5f1	MuC_Agedup	MuC
4.78E-09	0.4962833	0.65	0.604	0.000115	Mxd4	MuC_Agedup	MuC
8.79E-15	0.4953575	0.633	0.446	2.11E-10	Tmed7	MuC_Agedup	MuC
5.43E-15	0.4949677	0.55	0.329	1.30E-10	Arl6ip5	MuC_Agedup	MuC
5.51E-13	0.4948821	0.55	0.364	1.32E-08	Rbbp7	MuC_Agedup	MuC
2.29E-27	0.49438	0.321	0.003	5.49E-23	Syt7	MuC_Agedup	MuC
7.34E-10	0.4936727	0.822	0.934	1.76E-05	Arf5	MuC_Agedup	MuC
2.60E-07	0.4933447	0.696	0.696	0.006231	Arpc3	MuC_Agedup	MuC
7.12E-21	0.4933156	0.404	0.104	1.71E-16	Plekhb2	MuC_Agedup	MuC
5.96E-13	0.4930332	0.699	0.582	1.43E-08	Gsta4	MuC_Agedup	MuC
8.24E-11	0.4911675	0.799	0.835	1.98E-06	Pebp1	MuC_Agedup	MuC
4.94E-10	0.4910496	0.645	0.547	1.18E-05	Slc1a5	MuC_Agedup	MuC
4.59E-19	0.4910212	0.298	0.041	1.10E-14	Pmp22	MuC_Agedup	MuC
5.76E-10	0.4908587	0.59	0.468	1.38E-05	Tmem214	MuC_Agedup	MuC
2.12E-09	0.4906864	0.805	0.851	5.08E-05	Gnb2	MuC_Agedup	MuC
1.10E-08	0.4906464	0.696	0.741	0.000263	Tmem208	MuC_Agedup	MuC
8.18E-17	0.490561	0.513	0.209	1.96E-12	Fgl2	MuC_Agedup	MuC
6.85E-10	0.4904284	0.547	0.396	1.64E-05	Ogdh	MuC_Agedup	MuC
2.04E-14	0.4900524	0.894	0.921	4.90E-10	Rpl5	MuC_Agedup	MuC
3.16E-10	0.4898243	0.57	0.43	7.58E-06	Slc5a8	MuC_Agedup	MuC
3.18E-09	0.4892489	0.685	0.623	7.62E-05	Ssr1	MuC_Agedup	MuC
1.15E-12	0.4892426	0.868	0.937	2.76E-08	Ctsd	MuC_Agedup	MuC
3.96E-10	0.4887767	0.619	0.516	9.49E-06	Tmem50a	MuC_Agedup	MuC
1.88E-25	0.4884939	0.312	0.009	4.50E-21	St6galnac2	MuC_Agedup	MuC
1.08E-06	0.4882106	0.659	0.665	0.026009	Tecr	MuC_Agedup	MuC
1.53E-26	0.4873598	0.318	0.006	3.67E-22	Kcnk2	MuC_Agedup	MuC
9.91E-09	0.4872398	0.467	0.332	0.000238	Tceal8	MuC_Agedup	MuC
7.26E-23	0.4864217	0.501	0.171	1.74E-18	Prkab1	MuC_Agedup	MuC
5.65E-10	0.4858305	0.619	0.519	1.36E-05	Ergic3	MuC_Agedup	MuC
4.26E-17	0.4856653	0.564	0.332	1.02E-12	Naga	MuC_Agedup	MuC
2.90E-12	0.4854462	0.513	0.32	6.95E-08	Sdhc	MuC_Agedup	MuC
2.31E-26	0.4853418	0.304	0	5.55E-22	Atp7b	MuC_Agedup	MuC
5.54E-10	0.4846726	0.673	0.608	1.33E-05	Ndufv2	MuC_Agedup	MuC
5.66E-12	0.4843918	0.668	0.601	1.36E-07	Polr2k	MuC_Agedup	MuC
2.44E-12	0.4836709	0.616	0.494	5.84E-08	Glrx2	MuC_Agedup	MuC
7.48E-09	0.483495	0.688	0.722	0.000179	Aurkaip1	MuC_Agedup	MuC
2.64E-15	0.4823172	0.49	0.237	6.33E-11	Tmem30b	MuC_Agedup	MuC
1.30E-09	0.4822489	0.622	0.522	3.12E-05	Sod1	MuC_Agedup	MuC
4.86E-08	0.4821641	0.705	0.687	0.001166	Hnrnpa3	MuC_Agedup	MuC
1.70E-08	0.4820319	0.696	0.693	0.000408	Sf3b5	MuC_Agedup	MuC
1.05E-08	0.4811215	0.622	0.56	0.000251	Dnajc1	MuC_Agedup	MuC
1.45E-28	0.4810874	0.327	0	3.47E-24	Ern2	MuC_Agedup	MuC
4.77E-14	0.4808643	0.564	0.358	1.14E-09	Bola3	MuC_Agedup	MuC
4.70E-09	0.4793695	0.994	1	0.000113	mt-Nd1	MuC_Agedup	MuC
1.32E-10	0.4787772	0.49	0.307	3.16E-06	Acly	MuC_Agedup	MuC
6.28E-23	0.4786331	0.499	0.152	1.51E-18	Prom2	MuC_Agedup	MuC
2.09E-07	0.4786148	0.616	0.573	0.005014	Sar1b	MuC_Agedup	MuC

3.37E-07	0.4785742	0.736	0.75	0.008071	Hnrnpa2b1	MuC_Agedup	MuC
9.64E-11	0.4784558	0.622	0.535	2.31E-06	Fdx1	MuC_Agedup	MuC
1.78E-21	0.4781122	0.911	0.962	4.27E-17	Cox4i1	MuC_Agedup	MuC
1.39E-10	0.4780822	0.842	0.905	3.33E-06	Eef1g	MuC_Agedup	MuC
1.51E-09	0.4778638	0.636	0.532	3.62E-05	Tsta3	MuC_Agedup	MuC
1.30E-14	0.4777698	0.573	0.377	3.12E-10	D17Wsu92e	MuC_Agedup	MuC
1.16E-09	0.4776651	0.785	0.839	2.78E-05	Atp5o	MuC_Agedup	MuC
1.10E-12	0.4776493	0.456	0.244	2.63E-08	Alcam	MuC_Agedup	MuC
1.68E-19	0.4775093	0.436	0.146	4.04E-15	Adam28	MuC_Agedup	MuC
1.29E-09	0.4773228	0.599	0.494	3.08E-05	Sil1	MuC_Agedup	MuC
1.63E-06	0.4767624	0.593	0.519	0.039076	Ywhab	MuC_Agedup	MuC
2.91E-10	0.4764572	0.728	0.684	6.98E-06	Dynlrb1	MuC_Agedup	MuC
3.73E-12	0.4761442	0.607	0.481	8.95E-08	Grn	MuC_Agedup	MuC
1.50E-08	0.4758267	0.642	0.573	0.00036	Uap1	MuC_Agedup	MuC
1.50E-08	0.47456	0.668	0.611	0.00036	Prdx5	MuC_Agedup	MuC
6.95E-13	0.4741262	0.55	0.339	1.67E-08	Pgrmc1	MuC_Agedup	MuC
1.59E-17	0.4740695	0.587	0.342	3.80E-13	Prrc2b	MuC_Agedup	MuC
2.62E-07	0.4725447	0.65	0.57	0.006282	Iqgap1	MuC_Agedup	MuC
8.11E-09	0.4717264	0.648	0.62	0.000195	Tmco1	MuC_Agedup	MuC
8.56E-14	0.4712449	0.923	0.978	2.05E-09	Rpl3	MuC_Agedup	MuC
5.73E-10	0.4710555	0.711	0.725	1.37E-05	Ak3	MuC_Agedup	MuC
4.41E-19	0.4709417	0.963	0.981	1.06E-14	Rps3	MuC_Agedup	MuC
5.74E-10	0.4708872	0.607	0.478	1.38E-05	Mat2a	MuC_Agedup	MuC
3.80E-07	0.4708351	0.685	0.687	0.009108	Rac1	MuC_Agedup	MuC
6.72E-08	0.4698163	0.633	0.573	0.001613	Mgat4a	MuC_Agedup	MuC
8.49E-13	0.4697604	0.542	0.373	2.04E-08	Scamp2	MuC_Agedup	MuC
1.33E-08	0.469682	0.613	0.547	0.000319	Dph3	MuC_Agedup	MuC
3.29E-13	0.4687806	0.533	0.339	7.88E-09	Hook1	MuC_Agedup	MuC
7.16E-09	0.4687225	0.607	0.532	0.000172	Golga4	MuC_Agedup	MuC
6.14E-21	0.4684863	0.977	0.984	1.47E-16	Rps16	MuC_Agedup	MuC
8.27E-10	0.4674552	0.656	0.617	1.98E-05	Mrps34	MuC_Agedup	MuC
1.09E-12	0.4672733	0.616	0.475	2.62E-08	Tm2d1	MuC_Agedup	MuC
4.20E-19	0.4665813	0.914	0.956	1.01E-14	Xbp1	MuC_Agedup	MuC
7.72E-12	0.4663121	0.559	0.38	1.85E-07	Trpc4ap	MuC_Agedup	MuC
7.35E-23	0.4657306	0.401	0.085	1.76E-18	Pcsk7	MuC_Agedup	MuC
3.81E-15	0.4649651	0.501	0.272	9.14E-11	Glg1	MuC_Agedup	MuC
1.15E-10	0.4637069	0.693	0.68	2.77E-06	Uqcc2	MuC_Agedup	MuC
3.08E-10	0.4636102	0.585	0.456	7.39E-06	Ddx6	MuC_Agedup	MuC
2.47E-15	0.4633216	0.45	0.209	5.91E-11	Prr13	MuC_Agedup	MuC
2.49E-10	0.4626586	0.673	0.585	5.97E-06	Gtf2h5	MuC_Agedup	MuC
1.48E-11	0.4625826	0.562	0.367	3.54E-07	Mpzl1	MuC_Agedup	MuC
4.18E-11	0.4623864	0.625	0.509	1.00E-06	Tm2d2	MuC_Agedup	MuC
1.69E-06	0.4620416	0.722	0.737	0.040442	Gapdh	MuC_Agedup	MuC
7.00E-13	0.4609404	0.418	0.209	1.68E-08	Enpp5	MuC_Agedup	MuC
1.33E-06	0.4607989	0.759	0.82	0.031977	Sdcbp	MuC_Agedup	MuC
3.01E-17	0.4606999	0.507	0.247	7.21E-13	Polr2l	MuC_Agedup	MuC
4.14E-10	0.4602736	0.599	0.491	9.92E-06	Chmp3	MuC_Agedup	MuC
1.54E-11	0.4601716	0.516	0.339	3.68E-07	Fkbp1a	MuC_Agedup	MuC
1.74E-07	0.4600681	0.576	0.5	0.00417	Nipal2	MuC_Agedup	MuC
1.93E-07	0.4588435	0.582	0.509	0.004628	Brd2	MuC_Agedup	MuC
5.23E-13	0.4581084	0.573	0.383	1.26E-08	Crb3	MuC_Agedup	MuC
2.29E-12	0.4576376	0.711	0.671	5.49E-08	0610012G03Rik	MuC_Agedup	MuC
3.48E-12	0.4573485	0.484	0.294	8.35E-08	Prorsd1	MuC_Agedup	MuC
1.84E-11	0.457229	0.516	0.342	4.41E-07	Tmed4	MuC_Agedup	MuC
7.67E-13	0.4570994	0.413	0.19	1.84E-08	Serpinb6a	MuC_Agedup	MuC
1.78E-28	0.4570616	0.338	0.006	4.27E-24	Steap2	MuC_Agedup	MuC
1.42E-08	0.4569596	0.573	0.449	0.000339	BC005537	MuC_Agedup	MuC
5.79E-08	0.4567123	0.628	0.551	0.001389	Cd82	MuC_Agedup	MuC
1.09E-07	0.4567066	0.734	0.741	0.002622	Tbca	MuC_Agedup	MuC

5.69E-15	0.4563248	0.45	0.212	1.36E-10	E330009J07Rik	MuC_Agedup	MuC
2.17E-11	0.4556717	0.527	0.358	5.20E-07	Rab8a	MuC_Agedup	MuC
8.42E-07	0.4553405	0.633	0.611	0.020184	Myl12a	MuC_Agedup	MuC
2.88E-09	0.4531568	0.648	0.585	6.90E-05	Sfr1	MuC_Agedup	MuC
7.09E-09	0.4516314	0.542	0.421	0.00017	Ormdl3	MuC_Agedup	MuC
1.23E-14	0.4513144	0.501	0.266	2.96E-10	Cttn	MuC_Agedup	MuC
7.71E-11	0.4506625	0.513	0.351	1.85E-06	Far1	MuC_Agedup	MuC
2.00E-07	0.4505259	0.536	0.434	0.004798	Cgnl1	MuC_Agedup	MuC
4.76E-20	0.4503176	0.367	0.082	1.14E-15	Cthrc1	MuC_Agedup	MuC
1.61E-22	0.4501984	0.41	0.092	3.85E-18	Slc41a2	MuC_Agedup	MuC
8.45E-08	0.4498821	0.556	0.43	0.002025	Birc6	MuC_Agedup	MuC
6.93E-14	0.4495795	0.524	0.313	1.66E-09	Smim26	MuC_Agedup	MuC
2.95E-08	0.4492536	0.702	0.652	0.000707	Cyc1	MuC_Agedup	MuC
5.00E-14	0.4490017	0.539	0.329	1.20E-09	Lsm7	MuC_Agedup	MuC
1.60E-18	0.448693	0.43	0.146	3.84E-14	Psme1	MuC_Agedup	MuC
4.67E-14	0.447879	0.467	0.231	1.12E-09	Syvn1	MuC_Agedup	MuC
7.51E-12	0.4476022	0.499	0.307	1.80E-07	Azi2	MuC_Agedup	MuC
9.72E-13	0.4475185	0.544	0.364	2.33E-08	Vti1b	MuC_Agedup	MuC
5.98E-07	0.4472551	0.438	0.323	0.014334	Prdx4	MuC_Agedup	MuC
7.44E-18	0.4466163	0.951	0.975	1.78E-13	Rps3a1	MuC_Agedup	MuC
8.57E-12	0.4460857	0.582	0.408	2.06E-07	Zfp106	MuC_Agedup	MuC
4.74E-08	0.446074	0.659	0.611	0.001137	Clcn3	MuC_Agedup	MuC
2.48E-09	0.4451304	0.768	0.861	5.94E-05	Park7	MuC_Agedup	MuC
1.85E-07	0.4443547	0.722	0.696	0.004431	Csde1	MuC_Agedup	MuC
1.13E-19	0.4439684	0.954	0.978	2.70E-15	Rpsa	MuC_Agedup	MuC
1.12E-23	0.4425266	0.275	0	2.68E-19	Ildr2	MuC_Agedup	MuC
1.14E-10	0.4421292	0.63	0.525	2.75E-06	Mvb12a	MuC_Agedup	MuC
9.01E-08	0.4420671	0.805	0.883	0.002161	Cnpy2	MuC_Agedup	MuC
3.37E-14	0.4413721	0.49	0.247	8.09E-10	Gtf2i	MuC_Agedup	MuC
3.91E-10	0.4412497	0.504	0.348	9.37E-06	Armcx3	MuC_Agedup	MuC
4.32E-11	0.4409569	0.605	0.456	1.04E-06	Pdcd5	MuC_Agedup	MuC
6.08E-24	0.4405437	0.278	0	1.46E-19	Scgb1b3	MuC_Agedup	MuC
1.26E-18	0.4405417	0.341	0.073	3.02E-14	Ptprs	MuC_Agedup	MuC
1.96E-12	0.4403232	0.914	0.934	4.70E-08	Eef1b2	MuC_Agedup	MuC
1.87E-21	0.439946	0.367	0.07	4.48E-17	Cpq	MuC_Agedup	MuC
1.21E-10	0.4393903	0.59	0.443	2.91E-06	Ndufaf8	MuC_Agedup	MuC
3.14E-09	0.4385697	0.496	0.339	7.52E-05	Igf2r	MuC_Agedup	MuC
5.10E-07	0.4382913	0.619	0.528	0.01222	Vdac1	MuC_Agedup	MuC
3.66E-14	0.438276	0.433	0.196	8.79E-10	Slk	MuC_Agedup	MuC
1.04E-08	0.4378172	0.544	0.399	0.000249	R3hdm4	MuC_Agedup	MuC
7.13E-08	0.4373267	0.754	0.845	0.001711	Atp5c1	MuC_Agedup	MuC
8.83E-08	0.437094	0.519	0.377	0.002118	Vegfa	MuC_Agedup	MuC
2.32E-22	0.4359803	0.332	0.041	5.56E-18	Pls1	MuC_Agedup	MuC
3.38E-08	0.4354972	0.736	0.741	0.000811	Snrpd2	MuC_Agedup	MuC
1.58E-07	0.4351886	0.582	0.484	0.003779	Dag1	MuC_Agedup	MuC
5.47E-07	0.4350706	0.739	0.763	0.013124	Sypl	MuC_Agedup	MuC
1.15E-06	0.4339864	0.705	0.693	0.027691	Cldn7	MuC_Agedup	MuC
9.71E-09	0.4339771	0.605	0.497	0.000233	Cdk2ap2	MuC_Agedup	MuC
5.41E-07	0.4338536	0.645	0.665	0.012975	Atraid	MuC_Agedup	MuC
1.49E-06	0.4332151	0.479	0.364	0.03576	Foxq1	MuC_Agedup	MuC
8.20E-23	0.4331602	0.989	1	1.97E-18	Rpl23	MuC_Agedup	MuC
7.94E-24	0.4328746	0.324	0.025	1.91E-19	Slc37a1	MuC_Agedup	MuC
1.90E-13	0.4321981	0.51	0.301	4.57E-09	Smim27	MuC_Agedup	MuC
5.61E-09	0.4320233	0.685	0.674	0.000135	1110008P14Rik	MuC_Agedup	MuC
1.77E-07	0.4318961	0.937	0.991	0.004254	H3f3b	MuC_Agedup	MuC
1.35E-07	0.4317384	0.57	0.453	0.003232	Prpf4b	MuC_Agedup	MuC
1.95E-11	0.431268	0.53	0.361	4.69E-07	Pttg1ip	MuC_Agedup	MuC
1.91E-08	0.4308706	0.665	0.646	0.000459	Mtdh	MuC_Agedup	MuC
8.69E-09	0.4304159	0.736	0.722	0.000208	Psenen	MuC_Agedup	MuC

1.07E-06	0.4295964	0.734	0.788	0.025563	Crip2	MuC_Agedup	MuC
1.20E-13	0.4294363	0.567	0.367	2.88E-09	Taf6l	MuC_Agedup	MuC
1.06E-12	0.4282766	0.576	0.415	2.54E-08	Copg1	MuC_Agedup	MuC
4.05E-08	0.4282598	0.553	0.427	0.00097	Dnajc15	MuC_Agedup	MuC
7.78E-09	0.4275693	0.559	0.462	0.000187	Glmp	MuC_Agedup	MuC
6.51E-15	0.427334	0.453	0.203	1.56E-10	Spns2	MuC_Agedup	MuC
9.85E-20	0.4263782	0.367	0.085	2.36E-15	Irf8	MuC_Agedup	MuC
6.05E-14	0.4263147	0.507	0.291	1.45E-09	Sec24d	MuC_Agedup	MuC
1.99E-11	0.4258704	0.539	0.367	4.78E-07	Gm15417	MuC_Agedup	MuC
8.40E-13	0.4237292	0.533	0.329	2.01E-08	Gm11808	MuC_Agedup	MuC
9.09E-10	0.4237139	0.607	0.5	2.18E-05	Arfip2	MuC_Agedup	MuC
1.13E-14	0.4230134	0.453	0.209	2.71E-10	Cmtm6	MuC_Agedup	MuC
1.22E-24	0.4228276	0.298	0.006	2.93E-20	Clec14a	MuC_Agedup	MuC
1.84E-07	0.4224971	0.788	0.845	0.004404	Chpt1	MuC_Agedup	MuC
2.07E-15	0.42225	0.427	0.177	4.96E-11	Sft2d1	MuC_Agedup	MuC
5.69E-10	0.4221137	0.556	0.396	1.36E-05	Arfgef1	MuC_Agedup	MuC
2.44E-17	0.4206327	0.438	0.18	5.85E-13	3110040N11Rik	MuC_Agedup	MuC
1.29E-11	0.4202074	0.613	0.481	3.08E-07	Fmc1	MuC_Agedup	MuC
2.71E-13	0.419714	0.447	0.218	6.51E-09	Slc35a2	MuC_Agedup	MuC
2.14E-18	0.4196292	0.355	0.089	5.14E-14	1110008L16Rik	MuC_Agedup	MuC
3.40E-08	0.4181936	0.734	0.778	0.000816	Carhsp1	MuC_Agedup	MuC
8.26E-21	0.4177359	0.971	1	1.98E-16	Rps10	MuC_Agedup	MuC
7.13E-15	0.4167265	0.464	0.209	1.71E-10	Il6st	MuC_Agedup	MuC
9.31E-08	0.4158299	0.476	0.348	0.002233	Gjb2	MuC_Agedup	MuC
9.60E-12	0.4156401	0.436	0.241	2.30E-07	Fam114a1	MuC_Agedup	MuC
1.14E-08	0.4145778	0.582	0.522	0.000274	1810022K09Rik	MuC_Agedup	MuC
1.42E-06	0.4144001	0.628	0.576	0.03394	Cyb561	MuC_Agedup	MuC
9.59E-15	0.4142493	0.364	0.123	2.30E-10	Ppif	MuC_Agedup	MuC
1.22E-16	0.4138945	0.344	0.089	2.91E-12	Ptges	MuC_Agedup	MuC
9.26E-16	0.4137796	0.937	0.962	2.22E-11	Rplp2	MuC_Agedup	MuC
9.17E-07	0.4137435	0.645	0.639	0.02199	Tomm22	MuC_Agedup	MuC
3.06E-14	0.4135649	0.33	0.098	7.33E-10	Bambi	MuC_Agedup	MuC
3.23E-11	0.41353	0.464	0.294	7.74E-07	Tm9sf4	MuC_Agedup	MuC
3.33E-17	0.4130152	0.47	0.19	7.98E-13	Snhg9	MuC_Agedup	MuC
2.14E-07	0.4123406	0.679	0.655	0.005125	Polr2e	MuC_Agedup	MuC
1.49E-06	0.4119283	0.616	0.579	0.035741	Copa	MuC_Agedup	MuC
6.48E-13	0.4113243	0.441	0.218	1.55E-08	Man2b1	MuC_Agedup	MuC
1.34E-13	0.4107368	0.473	0.247	3.22E-09	Fam107b	MuC_Agedup	MuC
1.83E-09	0.4104057	0.57	0.418	4.39E-05	Mindy1	MuC_Agedup	MuC
2.56E-11	0.4102232	0.536	0.354	6.13E-07	Nisch	MuC_Agedup	MuC
3.97E-07	0.4101924	0.599	0.544	0.009527	Tmem205	MuC_Agedup	MuC
8.43E-07	0.4095312	0.358	0.203	0.020214	Cdkn1a	MuC_Agedup	MuC
1.87E-08	0.4091803	0.501	0.329	0.000448	Eif2s3x	MuC_Agedup	MuC
1.56E-10	0.4088886	0.55	0.396	3.75E-06	Ewsr1	MuC_Agedup	MuC
3.54E-09	0.4088866	0.605	0.475	8.48E-05	Pdcd6ip	MuC_Agedup	MuC
3.58E-19	0.4086648	0.427	0.136	8.59E-15	Fam20b	MuC_Agedup	MuC
7.83E-15	0.4084161	0.41	0.165	1.88E-10	Adgrg1	MuC_Agedup	MuC
7.58E-07	0.4083313	0.372	0.231	0.018174	Mfsd1	MuC_Agedup	MuC
7.37E-08	0.4083078	0.728	0.722	0.001767	Srsf5	MuC_Agedup	MuC
7.10E-15	0.4081799	0.438	0.193	1.70E-10	Fam102a	MuC_Agedup	MuC
3.92E-13	0.4080946	0.963	0.991	9.40E-09	Rpl10	MuC_Agedup	MuC
2.18E-08	0.4059181	0.573	0.478	0.000524	Gpi1	MuC_Agedup	MuC
2.31E-09	0.4050716	0.633	0.541	5.54E-05	Lamtor5	MuC_Agedup	MuC
1.69E-06	0.4049523	0.662	0.595	0.040638	Kif21a	MuC_Agedup	MuC
3.76E-16	0.4049186	0.427	0.168	9.02E-12	Phyh	MuC_Agedup	MuC
2.59E-16	0.4047022	0.951	0.997	6.22E-12	Rpl18	MuC_Agedup	MuC
1.58E-07	0.4041748	0.593	0.503	0.003791	Calm3	MuC_Agedup	MuC
9.28E-08	0.4034087	0.51	0.373	0.002226	Vps35	MuC_Agedup	MuC
5.01E-11	0.4027703	0.516	0.316	1.20E-06	Ibtk	MuC_Agedup	MuC

2.75E-09	0.4025227	0.573	0.446	6.58E-05	Ddx24	MuC_Agedup	MuC
7.10E-12	0.4023512	0.51	0.301	1.70E-07	Rab1b	MuC_Agedup	MuC
7.75E-12	0.4022133	0.49	0.301	1.86E-07	Fut8	MuC_Agedup	MuC
3.07E-15	0.4020016	0.966	0.991	7.36E-11	Rplp0	MuC_Agedup	MuC
6.81E-07	0.401993	0.716	0.734	0.016331	Azin1	MuC_Agedup	MuC
1.22E-16	0.4012986	0.55	0.294	2.92E-12	Ufsp2	MuC_Agedup	MuC
1.32E-06	0.4003098	0.533	0.43	0.03168	1500011K16Rik	MuC_Agedup	MuC
8.65E-11	0.3995601	0.544	0.364	2.07E-06	Ddr1	MuC_Agedup	MuC
1.44E-08	0.3995068	0.487	0.335	0.000345	Neu1	MuC_Agedup	MuC
8.01E-13	0.3993984	0.461	0.244	1.92E-08	Slc35a3	MuC_Agedup	MuC
8.19E-09	0.398854	0.496	0.345	0.000196	Anapc5	MuC_Agedup	MuC
9.44E-13	0.3978052	0.481	0.263	2.26E-08	Golt1b	MuC_Agedup	MuC
6.73E-07	0.3974146	0.524	0.421	0.01613	Psmd11	MuC_Agedup	MuC
9.44E-12	0.3971971	0.384	0.184	2.26E-07	Arhgap1	MuC_Agedup	MuC
4.57E-07	0.396905	0.57	0.487	0.01097	Psma6	MuC_Agedup	MuC
1.32E-10	0.3967537	0.473	0.297	3.17E-06	Mrps36	MuC_Agedup	MuC
4.32E-10	0.3964377	0.427	0.237	1.04E-05	Sgsm3	MuC_Agedup	MuC
4.41E-09	0.396048	0.55	0.386	0.000106	Pi4k2b	MuC_Agedup	MuC
2.17E-07	0.3957235	0.496	0.373	0.005199	Tcea1	MuC_Agedup	MuC
1.13E-10	0.3956515	0.567	0.415	2.71E-06	Mrps18c	MuC_Agedup	MuC
3.79E-09	0.3953799	0.53	0.399	9.08E-05	Anxa4	MuC_Agedup	MuC
9.22E-07	0.3953323	0.59	0.491	0.022109	G3bp2	MuC_Agedup	MuC
2.73E-07	0.3951422	0.559	0.462	0.006543	Esrp1	MuC_Agedup	MuC
2.34E-12	0.3950781	0.467	0.269	5.61E-08	Rnf13	MuC_Agedup	MuC
1.49E-13	0.3947861	0.481	0.256	3.57E-09	Gm5617	MuC_Agedup	MuC
5.09E-15	0.394319	0.45	0.203	1.22E-10	Endog	MuC_Agedup	MuC
8.73E-11	0.3938369	0.372	0.168	2.09E-06	Sirpa	MuC_Agedup	MuC
3.23E-09	0.3937229	0.542	0.399	7.74E-05	Ivns1abp	MuC_Agedup	MuC
2.31E-17	0.3933588	0.372	0.104	5.55E-13	Slc17a9	MuC_Agedup	MuC
1.95E-06	0.3922686	0.679	0.642	0.046664	Tsc22d4	MuC_Agedup	MuC
3.04E-09	0.3922262	0.476	0.31	7.30E-05	Ociad1	MuC_Agedup	MuC
6.84E-11	0.3918776	0.521	0.354	1.64E-06	Ddx50	MuC_Agedup	MuC
1.13E-08	0.3914831	0.49	0.326	0.000271	Cirbp	MuC_Agedup	MuC
9.65E-25	0.3912094	0.287	0	2.31E-20	Steap1	MuC_Agedup	MuC
2.13E-09	0.3910946	0.587	0.481	5.10E-05	Vps28	MuC_Agedup	MuC
7.43E-18	0.3906293	0.424	0.146	1.78E-13	Klhdc1	MuC_Agedup	MuC
7.96E-11	0.3905224	0.544	0.38	1.91E-06	Acat1	MuC_Agedup	MuC
4.17E-07	0.3904574	0.656	0.617	0.009999	Dynll2	MuC_Agedup	MuC
2.06E-10	0.3897104	0.539	0.386	4.94E-06	Vma21	MuC_Agedup	MuC
5.71E-11	0.3893753	0.47	0.269	1.37E-06	Pip4k2c	MuC_Agedup	MuC
1.41E-10	0.3892099	0.418	0.234	3.38E-06	Adam10	MuC_Agedup	MuC
3.58E-11	0.3887761	0.476	0.291	8.59E-07	Atf6	MuC_Agedup	MuC
1.02E-10	0.3879046	0.464	0.291	2.46E-06	Iah1	MuC_Agedup	MuC
1.92E-07	0.3866973	0.822	0.908	0.004594	Npm1	MuC_Agedup	MuC
4.54E-15	0.3862392	0.954	0.994	1.09E-10	Rpl15	MuC_Agedup	MuC
1.54E-10	0.3855621	0.447	0.256	3.70E-06	Myo1c	MuC_Agedup	MuC
7.25E-14	0.3853958	0.519	0.285	1.74E-09	Susd6	MuC_Agedup	MuC
1.58E-08	0.3843558	0.61	0.513	0.000379	Mcee	MuC_Agedup	MuC
1.15E-09	0.3839974	0.519	0.373	2.75E-05	Usp16	MuC_Agedup	MuC
2.09E-11	0.3838375	0.9	0.975	5.00E-07	Naca	MuC_Agedup	MuC
1.52E-07	0.3837317	0.587	0.497	0.003639	Mettl26	MuC_Agedup	MuC
1.01E-08	0.3832967	0.358	0.187	0.000243	B4galt3	MuC_Agedup	MuC
2.75E-09	0.3830137	0.436	0.256	6.61E-05	Sec24a	MuC_Agedup	MuC
9.84E-17	0.3829511	0.321	0.07	2.36E-12	Cacnb3	MuC_Agedup	MuC
1.77E-06	0.3828822	0.613	0.541	0.042468	Tpm1	MuC_Agedup	MuC
2.79E-12	0.3817088	0.513	0.323	6.68E-08	2300009A05Rik	MuC_Agedup	MuC
7.88E-09	0.3814933	0.825	0.886	0.000189	Btf3	MuC_Agedup	MuC
6.78E-09	0.3813862	0.564	0.443	0.000162	Gfer	MuC_Agedup	MuC
2.30E-08	0.3810259	0.49	0.348	0.000551	Tns3	MuC_Agedup	MuC

2.34E-09	0.3802034	0.567	0.402	5.61E-05	Mtch1	MuC_Agedup	MuC
1.48E-09	0.3801344	0.458	0.294	3.54E-05	Nfe2l1	MuC_Agedup	MuC
3.76E-07	0.3795523	0.57	0.481	0.009012	Derl1	MuC_Agedup	MuC
1.93E-08	0.3792153	0.378	0.228	0.000463	Pde4d	MuC_Agedup	MuC
7.46E-10	0.3791732	0.476	0.307	1.79E-05	Tex264	MuC_Agedup	MuC
1.76E-10	0.3781672	0.444	0.247	4.21E-06	Edem3	MuC_Agedup	MuC
6.30E-11	0.3779178	0.47	0.282	1.51E-06	Gpbp1l1	MuC_Agedup	MuC
2.09E-11	0.3777359	0.344	0.133	5.01E-07	Ldlr	MuC_Agedup	MuC
1.75E-09	0.3773543	0.438	0.25	4.19E-05	Mfsd11	MuC_Agedup	MuC
2.07E-16	0.3767661	0.335	0.085	4.97E-12	Tox3	MuC_Agedup	MuC
9.54E-07	0.3766452	0.616	0.544	0.022883	Aco2	MuC_Agedup	MuC
6.71E-21	0.3766092	0.37	0.073	1.61E-16	Lgals3bp	MuC_Agedup	MuC
1.50E-10	0.3764922	0.413	0.225	3.60E-06	Tmem38b	MuC_Agedup	MuC
1.50E-07	0.3764603	0.768	0.861	0.003603	Cyb5a	MuC_Agedup	MuC
4.28E-15	0.3760827	0.401	0.152	1.03E-10	Slc45a3	MuC_Agedup	MuC
8.36E-13	0.3743892	0.413	0.187	2.00E-08	Tmem97	MuC_Agedup	MuC
4.39E-09	0.3741554	0.49	0.329	0.000105	Acbd3	MuC_Agedup	MuC
3.17E-15	0.3721555	0.39	0.136	7.60E-11	Pmepa1	MuC_Agedup	MuC
2.09E-11	0.3720939	0.464	0.253	5.01E-07	Usp48	MuC_Agedup	MuC
6.54E-08	0.3713293	0.51	0.392	0.001569	Mmp15	MuC_Agedup	MuC
7.58E-08	0.3709541	0.628	0.582	0.001819	Ube2l3	MuC_Agedup	MuC
3.20E-12	0.3702553	0.553	0.358	7.67E-08	Psme4	MuC_Agedup	MuC
1.14E-06	0.3698237	0.599	0.535	0.02743	Rab11a	MuC_Agedup	MuC
3.91E-10	0.3696742	0.352	0.165	9.37E-06	MuCnt3	MuC_Agedup	MuC
5.85E-10	0.3696021	0.415	0.225	1.40E-05	Fam129b	MuC_Agedup	MuC
2.20E-23	0.3691494	0.298	0.013	5.27E-19	Amhr2	MuC_Agedup	MuC
3.56E-08	0.3691073	0.582	0.481	0.000853	Nus1	MuC_Agedup	MuC
7.32E-08	0.3687128	0.625	0.535	0.001754	Aamp	MuC_Agedup	MuC
1.62E-06	0.3686669	0.673	0.693	0.038964	Mrps12	MuC_Agedup	MuC
3.02E-14	0.3682796	0.436	0.196	7.24E-10	Bcl2l2	MuC_Agedup	MuC
8.73E-20	0.3679738	0.284	0.025	2.09E-15	Nrp2	MuC_Agedup	MuC
2.01E-08	0.3673897	0.527	0.408	0.000481	Ypel3	MuC_Agedup	MuC
1.15E-09	0.3672248	0.602	0.481	2.76E-05	Ppp1r11	MuC_Agedup	MuC
2.13E-08	0.3669866	0.55	0.424	0.000511	Cebpzos	MuC_Agedup	MuC
7.23E-07	0.3660324	0.542	0.459	0.017328	Cggbp1	MuC_Agedup	MuC
3.49E-19	0.3647547	0.332	0.063	8.37E-15	Gm8797	MuC_Agedup	MuC
3.26E-08	0.3647379	0.436	0.263	0.000782	Akap13	MuC_Agedup	MuC
2.05E-06	0.3641643	0.504	0.399	0.049264	Yme1l1	MuC_Agedup	MuC
1.44E-16	0.3637336	0.372	0.111	3.46E-12	Camk2n1	MuC_Agedup	MuC
2.59E-12	0.3627914	0.923	0.978	6.22E-08	Rpl7	MuC_Agedup	MuC
1.45E-13	0.3624489	0.966	0.987	3.48E-09	Rpl28	MuC_Agedup	MuC
8.51E-10	0.3622471	0.504	0.361	2.04E-05	Cmc1	MuC_Agedup	MuC
1.30E-10	0.361695	0.461	0.275	3.11E-06	Clmn	MuC_Agedup	MuC
7.20E-24	0.3616551	0.292	0.006	1.73E-19	Chrm1	MuC_Agedup	MuC
3.64E-07	0.3615708	0.579	0.513	0.008726	Pla2g16	MuC_Agedup	MuC
2.58E-07	0.3613344	0.599	0.541	0.006197	Trip11	MuC_Agedup	MuC
6.90E-07	0.3607651	0.542	0.453	0.016547	Sqstm1	MuC_Agedup	MuC
2.96E-09	0.3606869	0.464	0.297	7.09E-05	Psmd1	MuC_Agedup	MuC
8.68E-15	0.3606865	0.458	0.206	2.08E-10	Txlna	MuC_Agedup	MuC
1.86E-14	0.3606747	0.456	0.206	4.47E-10	Rnf141	MuC_Agedup	MuC
1.79E-07	0.359516	0.59	0.509	0.004297	Plpp5	MuC_Agedup	MuC
5.32E-08	0.3594932	0.891	0.965	0.001276	Rpl29	MuC_Agedup	MuC
3.99E-07	0.3588427	0.418	0.278	0.009563	Scamp1	MuC_Agedup	MuC
8.40E-08	0.358707	0.51	0.37	0.002014	Larp1	MuC_Agedup	MuC
8.74E-11	0.3576032	0.53	0.354	2.10E-06	Slirp	MuC_Agedup	MuC
9.65E-11	0.3574146	0.521	0.323	2.31E-06	Diaph1	MuC_Agedup	MuC
8.92E-10	0.357232	0.499	0.348	2.14E-05	Polr2m	MuC_Agedup	MuC
4.86E-07	0.3572264	0.547	0.465	0.01166	Unc50	MuC_Agedup	MuC
6.63E-08	0.3571464	0.395	0.253	0.001591	Gpr180	MuC_Agedup	MuC

5.88E-08	0.3570051	0.527	0.421	0.001411	Blvrb	MuC_Agedup	MuC
2.06E-09	0.3565846	0.43	0.263	4.94E-05	Pon3	MuC_Agedup	MuC
9.14E-08	0.3557496	0.487	0.342	0.002193	Amfr	MuC_Agedup	MuC
5.29E-07	0.3550996	0.799	0.842	0.012696	Nfib	MuC_Agedup	MuC
3.29E-07	0.3548387	0.499	0.37	0.007887	C77080	MuC_Agedup	MuC
9.21E-08	0.3538929	0.453	0.329	0.002208	Sh3bgr	MuC_Agedup	MuC
1.67E-17	0.3533361	0.407	0.136	4.01E-13	Nme2	MuC_Agedup	MuC
5.92E-07	0.3530607	0.599	0.528	0.014193	Csnk2b	MuC_Agedup	MuC
2.66E-07	0.3528766	0.536	0.424	0.006372	Rnf11	MuC_Agedup	MuC
1.67E-09	0.3528421	0.456	0.278	4.00E-05	Mapre2	MuC_Agedup	MuC
9.42E-08	0.3521248	0.43	0.272	0.002258	Slc43a2	MuC_Agedup	MuC
6.96E-11	0.3520814	0.47	0.263	1.67E-06	9530068E07Rik	MuC_Agedup	MuC
2.05E-09	0.3519559	0.384	0.203	4.92E-05	Bex3	MuC_Agedup	MuC
2.75E-10	0.3519312	0.441	0.25	6.59E-06	Rin2	MuC_Agedup	MuC
9.40E-08	0.3516318	0.464	0.313	0.002253	Mrpl41	MuC_Agedup	MuC
3.19E-07	0.3515253	0.596	0.525	0.007639	Mrpl18	MuC_Agedup	MuC
6.12E-12	0.3510258	0.393	0.18	1.47E-07	Ccdc167	MuC_Agedup	MuC
3.72E-10	0.3509488	0.461	0.278	8.92E-06	Fam3c	MuC_Agedup	MuC
6.90E-09	0.3507085	0.542	0.38	0.000166	2610507B11Rik	MuC_Agedup	MuC
7.23E-10	0.3506359	0.424	0.247	1.73E-05	Fh1	MuC_Agedup	MuC
3.61E-12	0.3503562	0.453	0.244	8.65E-08	Ktn1	MuC_Agedup	MuC
1.58E-06	0.3499698	0.456	0.339	0.037819	Ube2q2	MuC_Agedup	MuC
1.41E-08	0.3495728	0.433	0.272	0.000338	Mier1	MuC_Agedup	MuC
9.20E-10	0.3495622	0.501	0.323	2.21E-05	Fry	MuC_Agedup	MuC
1.39E-11	0.3489774	0.433	0.222	3.33E-07	Gpd1l	MuC_Agedup	MuC
4.63E-10	0.3487271	0.427	0.234	1.11E-05	Pja2	MuC_Agedup	MuC
5.26E-08	0.3477862	0.453	0.329	0.001262	Smim20	MuC_Agedup	MuC
3.16E-12	0.347609	0.43	0.222	7.58E-08	Dhx40	MuC_Agedup	MuC
3.81E-09	0.3471513	0.447	0.263	9.13E-05	Mgea5	MuC_Agedup	MuC
5.38E-08	0.3471135	0.521	0.37	0.001289	Ptbp1	MuC_Agedup	MuC
8.55E-07	0.3465851	0.579	0.456	0.020493	Trim2	MuC_Agedup	MuC
1.53E-06	0.3462003	0.639	0.617	0.036772	Llph	MuC_Agedup	MuC
4.71E-09	0.3461106	0.519	0.348	0.000113	Ano1	MuC_Agedup	MuC
1.40E-08	0.345675	0.441	0.288	0.000335	Mkrn1	MuC_Agedup	MuC
2.24E-07	0.34505	0.481	0.348	0.00537	Sgms1	MuC_Agedup	MuC
2.24E-07	0.3450449	0.481	0.348	0.005383	Rab11b	MuC_Agedup	MuC
7.25E-12	0.3447456	0.37	0.136	1.74E-07	Gsn	MuC_Agedup	MuC
9.90E-10	0.3445321	0.47	0.31	2.37E-05	Cdc34	MuC_Agedup	MuC
3.81E-13	0.3442845	0.407	0.18	9.13E-09	Inafm1	MuC_Agedup	MuC
6.84E-08	0.3442598	0.481	0.345	0.001641	B230118H07Rik	MuC_Agedup	MuC
2.99E-12	0.3436173	0.966	0.987	7.18E-08	Rps15a	MuC_Agedup	MuC
2.94E-07	0.3429111	0.467	0.335	0.007047	Baz2b	MuC_Agedup	MuC
9.53E-07	0.3427205	0.556	0.478	0.022851	Selenoh	MuC_Agedup	MuC
7.47E-15	0.3426534	0.479	0.244	1.79E-10	Cpne8	MuC_Agedup	MuC
1.57E-09	0.3424305	0.547	0.405	3.77E-05	Pigp	MuC_Agedup	MuC
3.52E-09	0.3422024	0.559	0.399	8.44E-05	Knop1	MuC_Agedup	MuC
1.91E-19	0.3419782	0.278	0.025	4.59E-15	Cd59a	MuC_Agedup	MuC
3.47E-11	0.3418735	0.255	0.07	8.31E-07	Utp14b	MuC_Agedup	MuC
1.79E-06	0.3417938	0.622	0.579	0.04301	Arpc5l	MuC_Agedup	MuC
1.01E-06	0.3411103	0.398	0.269	0.024336	Trappc1	MuC_Agedup	MuC
1.41E-06	0.3410124	0.842	0.921	0.03383	Eif5a	MuC_Agedup	MuC
5.02E-08	0.3408464	0.427	0.269	0.001205	Cacfd1	MuC_Agedup	MuC
1.76E-06	0.3408283	0.424	0.285	0.042118	Elf3	MuC_Agedup	MuC
1.91E-09	0.340263	0.352	0.171	4.58E-05	Pigk	MuC_Agedup	MuC
2.16E-07	0.3401918	0.599	0.513	0.005181	Smim11	MuC_Agedup	MuC
6.36E-08	0.3401355	0.473	0.32	0.001525	Slc30a7	MuC_Agedup	MuC
5.67E-10	0.3400637	0.481	0.313	1.36E-05	Svip	MuC_Agedup	MuC
1.52E-10	0.3395722	0.487	0.297	3.64E-06	Gna11	MuC_Agedup	MuC
2.61E-07	0.3389271	0.464	0.316	0.00625	Tmem123	MuC_Agedup	MuC

2.41E-08	0.3383657	0.487	0.329	0.000579	Mpdu1	MuC_Agedup	MuC
5.21E-10	0.3368961	0.473	0.278	1.25E-05	Acox1	MuC_Agedup	MuC
5.54E-14	0.336637	0.284	0.07	1.33E-09	1810055G02Rik	MuC_Agedup	MuC
1.09E-07	0.336329	0.387	0.234	0.002609	Ndel1	MuC_Agedup	MuC
3.73E-16	0.3362027	0.312	0.073	8.95E-12	Inha	MuC_Agedup	MuC
5.14E-15	0.3361649	0.344	0.108	1.23E-10	Hsbp1l1	MuC_Agedup	MuC
2.69E-07	0.3358549	0.47	0.332	0.006451	Pole4	MuC_Agedup	MuC
2.98E-11	0.3358257	0.278	0.089	7.14E-07	Zfp612	MuC_Agedup	MuC
3.71E-10	0.3356533	0.519	0.329	8.90E-06	Tpcn1	MuC_Agedup	MuC
8.31E-07	0.3354319	0.573	0.475	0.019938	2900097C17Rik	MuC_Agedup	MuC
9.73E-07	0.3351445	0.507	0.383	0.023323	Pkn2	MuC_Agedup	MuC
1.50E-10	0.3344267	0.352	0.155	3.59E-06	Slc35e1	MuC_Agedup	MuC
6.08E-21	0.333781	0.278	0.016	1.46E-16	Tbx1	MuC_Agedup	MuC
1.00E-08	0.3331566	0.562	0.405	0.00024	Msi2	MuC_Agedup	MuC
2.78E-15	0.332991	0.272	0.047	6.66E-11	Cdkn2a	MuC_Agedup	MuC
9.93E-07	0.3323052	0.636	0.563	0.02382	Ubxn1	MuC_Agedup	MuC
4.91E-08	0.3311454	0.444	0.294	0.001177	HmMuCl	MuC_Agedup	MuC
2.23E-12	0.3310154	0.427	0.196	5.34E-08	Impdh1	MuC_Agedup	MuC
1.94E-21	0.3306797	0.344	0.051	4.64E-17	1500011B03Rik	MuC_Agedup	MuC
1.05E-06	0.3300679	0.544	0.456	0.025098	Rassf3	MuC_Agedup	MuC
1.90E-10	0.3300389	0.361	0.171	4.55E-06	Stim2	MuC_Agedup	MuC
3.84E-10	0.3297789	0.407	0.218	9.21E-06	Ctsa	MuC_Agedup	MuC
7.32E-07	0.3294862	0.444	0.278	0.017558	Insig2	MuC_Agedup	MuC
1.14E-06	0.3290732	0.487	0.367	0.027359	Riok3	MuC_Agedup	MuC
1.29E-11	0.3287913	0.456	0.253	3.08E-07	Btg3	MuC_Agedup	MuC
6.94E-09	0.3287828	0.516	0.358	0.000166	Vps4b	MuC_Agedup	MuC
1.20E-07	0.3284103	0.616	0.5	0.002878	Mrpl58	MuC_Agedup	MuC
1.25E-10	0.3272559	0.47	0.275	3.00E-06	Gmppa	MuC_Agedup	MuC
3.90E-19	0.3270736	0.361	0.085	9.34E-15	AL590144.2	MuC_Agedup	MuC
6.56E-15	0.3263038	0.338	0.101	1.57E-10	Kit	MuC_Agedup	MuC
8.29E-09	0.3261833	0.501	0.345	0.000199	Trappc6b	MuC_Agedup	MuC
2.51E-10	0.3253556	0.504	0.339	6.02E-06	Acot13	MuC_Agedup	MuC
5.05E-11	0.3251445	0.378	0.168	1.21E-06	Noxo1	MuC_Agedup	MuC
2.07E-15	0.3248411	0.433	0.174	4.96E-11	Grcc10	MuC_Agedup	MuC
1.74E-10	0.3244714	0.387	0.196	4.18E-06	Tusc2	MuC_Agedup	MuC
1.79E-06	0.3242802	0.427	0.31	0.042945	Ppp1r1b	MuC_Agedup	MuC
5.45E-20	0.3236429	0.266	0.016	1.31E-15	Vtcn1	MuC_Agedup	MuC
2.91E-10	0.323426	0.413	0.222	6.97E-06	Pafah1b3	MuC_Agedup	MuC
4.74E-07	0.3232977	0.378	0.225	0.011361	Utrn	MuC_Agedup	MuC
2.01E-11	0.323018	0.41	0.209	4.82E-07	Tmc4	MuC_Agedup	MuC
5.57E-07	0.3229177	0.539	0.424	0.013353	Atp6v0d1	MuC_Agedup	MuC
1.29E-08	0.3223146	0.461	0.301	0.00031	Tmem63b	MuC_Agedup	MuC
1.56E-06	0.3222572	0.487	0.373	0.037423	Chrac1	MuC_Agedup	MuC
2.75E-07	0.3220047	0.507	0.386	0.006598	Pigyl	MuC_Agedup	MuC
1.25E-09	0.3210115	0.43	0.244	3.00E-05	Padi2	MuC_Agedup	MuC
1.58E-07	0.3210073	0.479	0.323	0.00379	Prpf8	MuC_Agedup	MuC
4.00E-09	0.3209082	0.381	0.19	9.58E-05	Srebf1	MuC_Agedup	MuC
9.53E-15	0.3205277	0.372	0.13	2.29E-10	Pgm3	MuC_Agedup	MuC
4.75E-09	0.3199892	0.309	0.142	0.000114	Sav1	MuC_Agedup	MuC
1.16E-06	0.3192147	0.479	0.354	0.02787	Top2b	MuC_Agedup	MuC
7.67E-12	0.3184058	0.361	0.155	1.84E-07	Fam241a	MuC_Agedup	MuC
5.88E-10	0.3182403	0.45	0.25	1.41E-05	Ubr3	MuC_Agedup	MuC
3.24E-07	0.3173713	0.487	0.345	0.007778	Ubap2l	MuC_Agedup	MuC
2.31E-09	0.3169238	0.444	0.25	5.53E-05	Plxnb2	MuC_Agedup	MuC
4.61E-07	0.3168082	0.415	0.272	0.011046	Atxn2l	MuC_Agedup	MuC
1.67E-16	0.3167998	0.344	0.082	4.00E-12	Eya2	MuC_Agedup	MuC
3.94E-08	0.3156324	0.507	0.367	0.000944	Dnlz	MuC_Agedup	MuC
5.46E-09	0.3155082	0.427	0.247	0.000131	Uxt	MuC_Agedup	MuC
1.20E-10	0.3153588	0.358	0.158	2.89E-06	2510039O18Rik	MuC_Agedup	MuC

1.51E-07	0.3152447	0.378	0.228	0.003619	Fkbp9	MuC_Agedup	MuC
1.73E-06	0.3148447	0.527	0.421	0.041375	Ckmt1	MuC_Agedup	MuC
2.81E-07	0.3147837	0.372	0.225	0.006735	Mrps10	MuC_Agedup	MuC
2.57E-15	0.3143143	0.226	0.022	6.16E-11	Ptprz1	MuC_Agedup	MuC
1.46E-06	0.313669	0.539	0.418	0.035028	Ssbp3	MuC_Agedup	MuC
1.07E-07	0.3128422	0.318	0.152	0.002564	Tfcp2l1	MuC_Agedup	MuC
1.42E-09	0.3125639	0.378	0.196	3.39E-05	Tmem60	MuC_Agedup	MuC
7.94E-07	0.3121136	0.539	0.421	0.019041	Ubxn2a	MuC_Agedup	MuC
4.58E-09	0.31175	0.51	0.335	0.00011	Vamp2	MuC_Agedup	MuC
1.72E-06	0.3116769	0.504	0.392	0.041298	Mrps15	MuC_Agedup	MuC
3.48E-10	0.3114287	0.954	0.984	8.36E-06	Rpl26	MuC_Agedup	MuC
1.53E-07	0.3111018	0.464	0.326	0.003681	Cox20	MuC_Agedup	MuC
8.94E-12	0.3110635	0.321	0.117	2.14E-07	Rhoj	MuC_Agedup	MuC
8.83E-07	0.3108665	0.496	0.364	0.021187	Ormdl2	MuC_Agedup	MuC
1.62E-08	0.310771	0.481	0.329	0.000389	Smim4	MuC_Agedup	MuC
9.34E-08	0.3106886	0.55	0.43	0.00224	Suclg2	MuC_Agedup	MuC
8.77E-22	0.3103978	0.261	0.003	2.10E-17	S100b	MuC_Agedup	MuC
7.04E-07	0.3099085	0.415	0.272	0.016886	Tmem109	MuC_Agedup	MuC
2.14E-07	0.3093762	0.401	0.256	0.005135	Mcrip2	MuC_Agedup	MuC
6.22E-09	0.3092142	0.375	0.196	0.000149	Lrp5	MuC_Agedup	MuC
1.89E-06	0.3090222	0.372	0.228	0.045265	B4galt1	MuC_Agedup	MuC
3.61E-07	0.3089692	0.501	0.383	0.008651	Tars	MuC_Agedup	MuC
1.98E-06	0.3088812	0.458	0.326	0.047438	Epn1	MuC_Agedup	MuC
5.01E-07	0.3084958	0.622	0.547	0.012015	Stt3b	MuC_Agedup	MuC
1.31E-06	0.3082852	0.413	0.275	0.031344	Sssca1	MuC_Agedup	MuC
5.76E-12	0.3075143	0.324	0.111	1.38E-07	Agtrap	MuC_Agedup	MuC
4.74E-08	0.3071011	0.404	0.247	0.001138	Tmem218	MuC_Agedup	MuC
3.39E-10	0.306935	0.318	0.13	8.13E-06	Entpd7	MuC_Agedup	MuC
5.53E-07	0.3066428	0.436	0.304	0.013252	Zfp560	MuC_Agedup	MuC
1.63E-06	0.3064492	0.521	0.418	0.038973	Ssbp1	MuC_Agedup	MuC
2.66E-20	0.3062996	0.238	0	6.38E-16	Galnt5	MuC_Agedup	MuC
1.26E-06	0.306062	0.395	0.256	0.030158	DMuCr2	MuC_Agedup	MuC
1.28E-08	0.3060298	0.427	0.259	0.000307	Tlk1	MuC_Agedup	MuC
2.52E-10	0.3056771	0.47	0.288	6.05E-06	Csnk2a1	MuC_Agedup	MuC
3.42E-09	0.3056237	0.393	0.218	8.21E-05	Mesd	MuC_Agedup	MuC
1.83E-20	0.3042349	0.246	0.003	4.38E-16	Slc38a5	MuC_Agedup	MuC
8.07E-07	0.30419	0.493	0.361	0.01935	Gstm5	MuC_Agedup	MuC
1.18E-09	0.3041471	0.398	0.218	2.84E-05	Nfx1	MuC_Agedup	MuC
5.93E-08	0.3038534	0.37	0.209	0.001421	Myadm	MuC_Agedup	MuC
3.30E-14	0.3038039	0.249	0.044	7.92E-10	Gpx8	MuC_Agedup	MuC
1.63E-07	0.3034416	0.456	0.307	0.00392	Grina	MuC_Agedup	MuC
4.66E-09	0.3033192	0.421	0.259	0.000112	Bet1l	MuC_Agedup	MuC
4.82E-07	0.3028882	0.47	0.339	0.011565	Tnrc6b	MuC_Agedup	MuC
1.76E-09	0.302508	0.456	0.272	4.22E-05	Ascc3	MuC_Agedup	MuC
1.63E-12	0.3016357	0.37	0.149	3.92E-08	8-Mar	MuC_Agedup	MuC
4.22E-08	0.3014327	0.318	0.155	0.001013	Enc1	MuC_Agedup	MuC
1.63E-06	0.3010682	0.404	0.282	0.039032	Desi2	MuC_Agedup	MuC
2.72E-09	0.3010014	0.487	0.31	6.52E-05	BC004004	MuC_Agedup	MuC
1.93E-21	0.3005907	0.264	0.006	4.62E-17	Adh1	MuC_Agedup	MuC
2.66E-11	0.3005123	0.324	0.12	6.39E-07	Met	MuC_Agedup	MuC
3.87E-09	0.3003615	0.338	0.165	9.27E-05	Maged2	MuC_Agedup	MuC
1.01E-09	0.2999067	0.98	0.997	2.43E-05	Rpl32	MuC_Agedup	MuC
3.95E-09	0.2996247	0.281	0.111	9.48E-05	Kcne3	MuC_Agedup	MuC
3.75E-12	0.2993922	0.347	0.13	8.98E-08	Slc28a3	MuC_Agedup	MuC
1.04E-08	0.2983721	0.384	0.218	0.000251	Actr1b	MuC_Agedup	MuC
3.29E-07	0.2971625	0.519	0.402	0.007902	Psmd3	MuC_Agedup	MuC
4.21E-11	0.2971197	0.367	0.168	1.01E-06	Parva	MuC_Agedup	MuC
1.05E-07	0.2966135	0.372	0.215	0.002508	Slc29a1	MuC_Agedup	MuC
7.32E-08	0.2963862	0.344	0.18	0.001755	Xpnpep1	MuC_Agedup	MuC

2.97E-07	0.2963307	0.461	0.316	0.007122	Mthfd2l	MuC_Agedup	MuC
2.78E-11	0.2962081	0.378	0.174	6.65E-07	Cox16	MuC_Agedup	MuC
8.86E-08	0.2953158	0.436	0.275	0.002125	Slc48a1	MuC_Agedup	MuC
3.85E-16	0.2949802	0.341	0.089	9.23E-12	2900076A07Rik	MuC_Agedup	MuC
2.81E-16	0.2949245	0.212	0.009	6.73E-12	Bex2	MuC_Agedup	MuC
4.24E-07	0.2945326	0.398	0.237	0.010173	Anxa11	MuC_Agedup	MuC
1.76E-07	0.2941631	0.438	0.282	0.004232	Nap1l4	MuC_Agedup	MuC
2.44E-10	0.2931357	0.407	0.206	5.85E-06	Vat1	MuC_Agedup	MuC
2.00E-06	0.2922584	0.424	0.291	0.048051	Tbc1d20	MuC_Agedup	MuC
4.55E-07	0.2919379	0.458	0.332	0.010903	Znhit1	MuC_Agedup	MuC
7.98E-13	0.291645	0.218	0.032	1.91E-08	Dcn	MuC_Agedup	MuC
1.15E-06	0.2913834	0.461	0.326	0.027671	Jak1	MuC_Agedup	MuC
1.47E-09	0.290865	0.344	0.158	3.52E-05	Ikbip	MuC_Agedup	MuC
2.49E-07	0.290425	0.404	0.241	0.005973	Celsr2	MuC_Agedup	MuC
2.16E-08	0.2901843	0.378	0.209	0.000517	Dhrs3	MuC_Agedup	MuC
1.72E-07	0.2897658	0.415	0.269	0.004133	Adam9	MuC_Agedup	MuC
3.13E-09	0.2896665	0.493	0.323	7.50E-05	Mpv17l2	MuC_Agedup	MuC
2.12E-08	0.2896225	0.347	0.168	0.000507	Ten1	MuC_Agedup	MuC
3.29E-07	0.2894647	0.888	0.962	0.007882	Rack1	MuC_Agedup	MuC
6.28E-20	0.289324	0.272	0.019	1.51E-15	Csprs	MuC_Agedup	MuC
5.53E-09	0.2886617	0.513	0.339	0.000133	Stk11	MuC_Agedup	MuC
1.73E-08	0.2883953	0.335	0.171	0.000414	Arrdc1	MuC_Agedup	MuC
7.08E-07	0.2880081	0.441	0.307	0.016972	Glud1	MuC_Agedup	MuC
5.33E-08	0.2873974	0.476	0.307	0.001279	Pabpc4	MuC_Agedup	MuC
3.85E-09	0.2873023	0.289	0.12	9.23E-05	Prss23	MuC_Agedup	MuC
3.84E-11	0.2872069	0.341	0.139	9.22E-07	Fam8a1	MuC_Agedup	MuC
3.39E-07	0.2867148	0.519	0.377	0.008121	Hspa4	MuC_Agedup	MuC
5.71E-19	0.2852482	0.229	0.003	1.37E-14	Plcb1	MuC_Agedup	MuC
4.44E-08	0.285054	0.47	0.304	0.001066	Psmc1	MuC_Agedup	MuC
6.96E-08	0.28494	0.249	0.095	0.001669	Gm26802	MuC_Agedup	MuC
1.36E-07	0.2848624	0.453	0.297	0.003253	Akr1b3	MuC_Agedup	MuC
7.61E-07	0.2846451	0.467	0.348	0.018254	Adrm1	MuC_Agedup	MuC
3.03E-11	0.2843511	0.378	0.177	7.27E-07	Golga5	MuC_Agedup	MuC
7.97E-11	0.2841214	0.433	0.222	1.91E-06	Phf14	MuC_Agedup	MuC
1.54E-08	0.2840798	0.415	0.234	0.00037	Tmem63a	MuC_Agedup	MuC
1.09E-10	0.2838667	0.427	0.212	2.60E-06	Pbxip1	MuC_Agedup	MuC
1.62E-17	0.2836707	0.252	0.022	3.90E-13	Sema3b	MuC_Agedup	MuC
6.78E-12	0.2835464	0.341	0.133	1.63E-07	Snx4	MuC_Agedup	MuC
4.95E-08	0.2833149	0.332	0.161	0.001186	Lnpep	MuC_Agedup	MuC
8.57E-08	0.2833117	0.332	0.18	0.002055	Naglu	MuC_Agedup	MuC
1.02E-07	0.283063	0.413	0.259	0.002442	Ppp1r37	MuC_Agedup	MuC
1.04E-11	0.2825388	0.381	0.168	2.50E-07	Adipor2	MuC_Agedup	MuC
4.74E-07	0.2825017	0.309	0.158	0.011359	Traf7	MuC_Agedup	MuC
2.00E-07	0.2824023	0.413	0.259	0.004795	Dda1	MuC_Agedup	MuC
1.66E-07	0.2821237	0.479	0.335	0.003987	Pink1	MuC_Agedup	MuC
3.89E-07	0.2817611	0.358	0.209	0.009324	Shoc2	MuC_Agedup	MuC
1.60E-09	0.28158	0.387	0.193	3.83E-05	Aldh3a2	MuC_Agedup	MuC
1.12E-09	0.2815067	0.393	0.206	2.68E-05	Pdzd11	MuC_Agedup	MuC
6.65E-09	0.280742	0.49	0.342	0.000159	Isca2	MuC_Agedup	MuC
4.61E-12	0.2806838	0.358	0.139	1.11E-07	Chchd6	MuC_Agedup	MuC
4.75E-13	0.2806046	0.295	0.082	1.14E-08	Kcnk6	MuC_Agedup	MuC
5.16E-12	0.2804023	0.289	0.085	1.24E-07	Arap1	MuC_Agedup	MuC
2.07E-06	0.2795475	0.318	0.177	0.049613	Usp4	MuC_Agedup	MuC
6.53E-07	0.2792419	0.418	0.282	0.015669	Zbtb8os	MuC_Agedup	MuC
1.43E-07	0.2790815	0.37	0.209	0.003432	Sardh	MuC_Agedup	MuC
1.10E-17	0.2789925	0.241	0.016	2.64E-13	Ephx4	MuC_Agedup	MuC
6.05E-07	0.2789038	0.355	0.212	0.01451	B4galnt1	MuC_Agedup	MuC
5.92E-08	0.2784719	0.304	0.155	0.00142	Rab3ip	MuC_Agedup	MuC
4.84E-07	0.2778174	0.321	0.174	0.011601	Atg13	MuC_Agedup	MuC

5.72E-08	0.2772333	0.395	0.231	0.001372	Cnot7	MuC_Agedup	MuC
4.88E-09	0.2766799	0.304	0.133	0.000117	Akip1	MuC_Agedup	MuC
3.52E-11	0.2762183	0.301	0.104	8.45E-07	Ndrg3	MuC_Agedup	MuC
5.55E-07	0.2760952	0.43	0.278	0.013303	Scyl1	MuC_Agedup	MuC
9.06E-08	0.275094	0.421	0.269	0.002173	Wbp2	MuC_Agedup	MuC
4.25E-07	0.2749925	0.461	0.316	0.010191	Cops7a	MuC_Agedup	MuC
1.48E-08	0.2738901	0.341	0.158	0.000356	Pof1b	MuC_Agedup	MuC
4.13E-18	0.2737053	0.246	0.016	9.91E-14	Hpn	MuC_Agedup	MuC
1.26E-07	0.273627	0.476	0.313	0.003031	Ube2z	MuC_Agedup	MuC
1.28E-06	0.2732201	0.364	0.222	0.030682	Wbp11	MuC_Agedup	MuC
1.60E-06	0.2726093	0.315	0.18	0.03847	Coq7	MuC_Agedup	MuC
2.60E-07	0.2717927	0.361	0.209	0.006224	Atp6v1h	MuC_Agedup	MuC
2.74E-07	0.2713276	0.37	0.218	0.006567	Ncaph2	MuC_Agedup	MuC
1.55E-11	0.2710763	0.275	0.082	3.73E-07	Reep6	MuC_Agedup	MuC
2.68E-12	0.2701201	0.324	0.111	6.42E-08	Slc35c1	MuC_Agedup	MuC
1.82E-11	0.2687866	0.281	0.089	4.36E-07	Stc2	MuC_Agedup	MuC
4.79E-12	0.2686009	0.175	0.016	1.15E-07	C2cd4b	MuC_Agedup	MuC
1.94E-12	0.2685133	0.287	0.082	4.64E-08	Ippk	MuC_Agedup	MuC
2.92E-17	0.2678826	0.272	0.035	7.00E-13	Aox3	MuC_Agedup	MuC
2.97E-07	0.2677938	0.473	0.345	0.007119	Polr3k	MuC_Agedup	MuC
1.53E-08	0.2664749	0.284	0.12	0.000366	Cxxc5	MuC_Agedup	MuC
6.80E-12	0.2658639	0.401	0.18	1.63E-07	Ptpn11	MuC_Agedup	MuC
2.58E-08	0.2653788	0.456	0.304	0.00062	Nudcd3	MuC_Agedup	MuC
9.03E-07	0.2649973	0.395	0.25	0.021649	AC149090.1	MuC_Agedup	MuC
2.47E-07	0.2645308	0.539	0.424	0.005912	Vps29	MuC_Agedup	MuC
4.31E-07	0.2644525	0.433	0.282	0.010326	Wls	MuC_Agedup	MuC
1.79E-06	0.2641875	0.453	0.326	0.042906	Lrpap1	MuC_Agedup	MuC
1.03E-08	0.2639304	0.45	0.288	0.000247	Card19	MuC_Agedup	MuC
8.80E-08	0.263266	0.364	0.209	0.002111	Gabarapl1	MuC_Agedup	MuC
3.59E-07	0.2632202	0.433	0.275	0.008618	Zc3h7a	MuC_Agedup	MuC
1.46E-07	0.2630356	0.41	0.253	0.003497	Spag9	MuC_Agedup	MuC
5.84E-08	0.2620111	0.384	0.222	0.0014	Hpcal1	MuC_Agedup	MuC
2.98E-07	0.2619199	0.301	0.146	0.007148	Slc30a9	MuC_Agedup	MuC
4.40E-11	0.2614967	0.281	0.092	1.05E-06	Ocel1	MuC_Agedup	MuC
9.94E-08	0.2609349	0.467	0.316	0.002384	Bsdc1	MuC_Agedup	MuC
1.90E-18	0.2607907	0.238	0.009	4.56E-14	Igf2bp2	MuC_Agedup	MuC
1.34E-06	0.2607794	0.433	0.288	0.032064	Macf1	MuC_Agedup	MuC
1.83E-06	0.2605883	0.327	0.18	0.043842	Cbx5	MuC_Agedup	MuC
2.87E-09	0.2604846	0.289	0.114	6.89E-05	Map2k7	MuC_Agedup	MuC
1.68E-17	0.2596112	0.241	0.016	4.02E-13	Sdc2	MuC_Agedup	MuC
5.27E-08	0.2595558	0.957	0.984	0.001263	Ptma	MuC_Agedup	MuC
8.52E-07	0.2588544	0.33	0.187	0.020438	Inppl1	MuC_Agedup	MuC
4.94E-07	0.2587933	0.327	0.184	0.011852	Golga3	MuC_Agedup	MuC
3.40E-08	0.2586199	0.246	0.095	0.000815	Dpp7	MuC_Agedup	MuC
5.03E-13	0.2583568	0.352	0.13	1.21E-08	Bloc1s1	MuC_Agedup	MuC
5.14E-09	0.2580842	0.338	0.158	0.000123	Sec23ip	MuC_Agedup	MuC
1.54E-06	0.257882	0.301	0.161	0.036834	Spry2	MuC_Agedup	MuC
7.48E-07	0.2578734	0.321	0.168	0.017936	Vps26b	MuC_Agedup	MuC
5.92E-08	0.2574238	0.284	0.12	0.001421	Ap1b1	MuC_Agedup	MuC
1.74E-13	0.257417	0.261	0.054	4.18E-09	Chpf2	MuC_Agedup	MuC
3.32E-16	0.2572472	0.352	0.092	7.97E-12	Tomm6	MuC_Agedup	MuC
6.02E-07	0.2568138	0.461	0.307	0.014436	Tacc2	MuC_Agedup	MuC
8.39E-17	0.2566459	0.258	0.032	2.01E-12	Triqk	MuC_Agedup	MuC
4.77E-11	0.2551353	0.309	0.108	1.14E-06	Ece1	MuC_Agedup	MuC
1.96E-08	0.2550075	0.413	0.247	0.00047	Ap3m1	MuC_Agedup	MuC
3.98E-08	0.2549327	0.289	0.541	0.000955	Mt2	MuC_Agedup	MuC
1.31E-17	0.254649	0.221	0.006	3.15E-13	Kctd12b	MuC_Agedup	MuC
8.14E-17	0.2538515	0.212	0.006	1.95E-12	Catspere2	MuC_Agedup	MuC
4.46E-07	0.2537346	0.278	0.13	0.010698	Hltf	MuC_Agedup	MuC

1.25E-07	0.2535257	0.355	0.196	0.002989	Gosr2	MuC_Agedup	MuC
8.08E-12	0.2530956	0.327	0.12	1.94E-07	0610010K14Rik	MuC_Agedup	MuC
3.16E-18	0.2527605	0.304	0.047	7.58E-14	Trp53i11	MuC_Agedup	MuC
1.13E-07	0.2527171	0.292	0.139	0.002713	Micu1	MuC_Agedup	MuC
2.58E-07	0.2514039	0.496	0.345	0.006175	Arl6ip1	MuC_Agedup	MuC
3.68E-11	0.2512004	0.249	0.066	8.83E-07	BC017158	MuC_Agedup	MuC
1.01E-07	0.2510614	0.41	0.25	0.002422	Ap1g1	MuC_Agedup	MuC
5.85E-07	0.2506417	0.499	0.383	0.014019	Ildr1	MuC_Agedup	MuC
1.03E-18	0.2502901	0.226	0.003	2.47E-14	Fam83e	MuC_Agedup	MuC
6.96E-08	0.2502246	0.361	0.193	0.001669	Dvl1	MuC_Agedup	MuC
1.75E-16	0.2500339	0.215	0.009	4.19E-12	1700066B19Rik	MuC_Agedup	MuC
2.25E-07	0.2495821	0.367	0.209	0.005399	Zfp395	MuC_Agedup	MuC
3.23E-13	0.248851	0.212	0.028	7.75E-09	Lfng	MuC_Agedup	MuC
2.48E-10	0.2486895	0.246	0.07	5.95E-06	Psmb8	MuC_Agedup	MuC
4.78E-12	0.2483352	0.292	0.089	1.15E-07	Tjp3	MuC_Agedup	MuC
5.43E-18	0.2469247	0.232	0.009	1.30E-13	Hist2h2ac	MuC_Agedup	MuC
1.09E-10	0.2468774	0.258	0.076	2.62E-06	Hebp1	MuC_Agedup	MuC
2.13E-07	0.2465774	0.301	0.149	0.005119	Llgl2	MuC_Agedup	MuC
3.92E-10	0.24572	0.278	0.098	9.40E-06	Phpt1	MuC_Agedup	MuC
1.73E-13	0.245657	0.289	0.073	4.14E-09	Chpf	MuC_Agedup	MuC
1.09E-07	0.2454233	0.41	0.247	0.002621	Ncstn	MuC_Agedup	MuC
6.12E-07	0.2453983	0.295	0.152	0.01467	Ppt1	MuC_Agedup	MuC
6.84E-10	0.2453315	0.281	0.098	1.64E-05	9-Sep	MuC_Agedup	MuC
9.90E-15	0.245208	0.209	0.016	2.37E-10	Ntn4	MuC_Agedup	MuC
1.75E-10	0.2436494	0.367	0.177	4.20E-06	Zfp9	MuC_Agedup	MuC
2.10E-10	0.2434127	0.315	0.123	5.04E-06	Klhdc7a	MuC_Agedup	MuC
1.86E-10	0.2431065	0.226	0.057	4.46E-06	Igdcc4	MuC_Agedup	MuC
1.63E-11	0.2421073	0.404	0.193	3.91E-07	Sdhaf4	MuC_Agedup	MuC
1.90E-06	0.2420232	0.358	0.215	0.04567	Trappc10	MuC_Agedup	MuC
1.59E-10	0.2419336	0.241	0.07	3.82E-06	Acsf3	MuC_Agedup	MuC
6.09E-10	0.240842	0.284	0.108	1.46E-05	Gxylt1	MuC_Agedup	MuC
5.46E-07	0.2407651	0.421	0.263	0.013105	Cep170b	MuC_Agedup	MuC
3.01E-07	0.2407424	0.315	0.158	0.007224	Brap	MuC_Agedup	MuC
3.06E-07	0.2407044	0.355	0.199	0.007349	Mtus1	MuC_Agedup	MuC
4.99E-07	0.2399362	0.39	0.241	0.011965	Gaa	MuC_Agedup	MuC
3.81E-13	0.2397946	0.309	0.085	9.13E-09	Dab2ip	MuC_Agedup	MuC
4.60E-09	0.2393714	0.96	0.994	0.00011	Rps18	MuC_Agedup	MuC
4.76E-17	0.2389429	0.201	0	1.14E-12	Slc41a3	MuC_Agedup	MuC
9.32E-09	0.2389054	0.261	0.098	0.000224	Samd8	MuC_Agedup	MuC
7.26E-09	0.2387343	0.35	0.165	0.000174	Golga2	MuC_Agedup	MuC
1.50E-07	0.2385276	0.332	0.171	0.003592	Vkorc1l1	MuC_Agedup	MuC
5.87E-08	0.2361905	0.281	0.127	0.001407	Wtip	MuC_Agedup	MuC
1.07E-10	0.2360461	0.318	0.12	2.57E-06	Lrrc8d	MuC_Agedup	MuC
4.76E-17	0.2351139	0.201	0	1.14E-12	Hpx	MuC_Agedup	MuC
1.41E-10	0.2338845	0.289	0.098	3.37E-06	Rpl27-ps3	MuC_Agedup	MuC
8.95E-09	0.2326086	0.272	0.108	0.000215	Dlg3	MuC_Agedup	MuC
3.30E-10	0.2324853	0.281	0.098	7.92E-06	Ralb	MuC_Agedup	MuC
3.03E-09	0.2324464	0.295	0.12	7.27E-05	Dctn5	MuC_Agedup	MuC
3.12E-07	0.2324066	0.37	0.215	0.00749	Rsph9	MuC_Agedup	MuC
6.77E-07	0.2323997	0.364	0.206	0.01624	Fcho2	MuC_Agedup	MuC
4.41E-09	0.2322347	0.249	0.082	0.000106	Cyth1	MuC_Agedup	MuC
1.49E-06	0.2321121	0.318	0.18	0.035642	Sidt1	MuC_Agedup	MuC
4.98E-07	0.231573	0.35	0.203	0.011942	Arfip1	MuC_Agedup	MuC
5.54E-13	0.2313226	0.324	0.101	1.33E-08	Ano10	MuC_Agedup	MuC
1.23E-09	0.2303024	0.275	0.092	2.94E-05	Ly6g6e	MuC_Agedup	MuC
5.34E-09	0.2302229	0.261	0.095	0.000128	Vipr1	MuC_Agedup	MuC
2.49E-11	0.2300393	0.266	0.076	5.97E-07	Usf3	MuC_Agedup	MuC
1.04E-08	0.2296649	0.318	0.149	0.000249	Arfgap1	MuC_Agedup	MuC
1.20E-06	0.2294432	0.427	0.285	0.028761	Insr	MuC_Agedup	MuC

2.47E-10	0.2294175	0.307	0.117	5.92E-06	Slc11a2	MuC_Agedup	MuC
4.05E-11	0.2292659	0.332	0.133	9.70E-07	Nbas	MuC_Agedup	MuC
1.03E-11	0.2289916	0.281	0.082	2.48E-07	Unc5a	MuC_Agedup	MuC
7.92E-08	0.228519	0.372	0.212	0.001899	Manbal	MuC_Agedup	MuC
8.68E-08	0.2282561	0.272	0.12	0.002081	Vps13b	MuC_Agedup	MuC
3.06E-10	0.2281645	0.203	0.044	7.34E-06	Emb	MuC_Agedup	MuC
4.41E-07	0.227744	0.378	0.225	0.010565	Mut	MuC_Agedup	MuC
9.39E-12	0.2274431	0.221	0.044	2.25E-07	Bvht	MuC_Agedup	MuC
1.61E-09	0.2269077	0.264	0.089	3.86E-05	Rnf213	MuC_Agedup	MuC
5.55E-09	0.2268239	0.344	0.161	0.000133	Ttc37	MuC_Agedup	MuC
1.75E-07	0.226519	0.321	0.171	0.004193	Jpt2	MuC_Agedup	MuC
7.13E-14	0.2260815	0.203	0.019	1.71E-09	B4galt4	MuC_Agedup	MuC
5.41E-07	0.226054	0.315	0.158	0.012981	Slc35a4	MuC_Agedup	MuC
7.66E-14	0.2258397	0.232	0.035	1.84E-09	Cpn1	MuC_Agedup	MuC
1.72E-06	0.2254917	0.367	0.228	0.041215	Ntmt1	MuC_Agedup	MuC
6.04E-17	0.2251884	0.206	0.003	1.45E-12	Cbfa2t3	MuC_Agedup	MuC
1.19E-07	0.2248985	0.401	0.244	0.002855	Coa6	MuC_Agedup	MuC
2.05E-17	0.2248175	0.212	0.003	4.91E-13	Mcf2l	MuC_Agedup	MuC
2.15E-13	0.2246317	0.203	0.022	5.16E-09	Cdr2l	MuC_Agedup	MuC
4.84E-08	0.2243567	0.266	0.114	0.00116	Crlf2	MuC_Agedup	MuC
3.96E-08	0.2240954	0.318	0.149	0.000949	Celsr1	MuC_Agedup	MuC
4.08E-08	0.2239846	0.278	0.12	0.000978	Smpd1	MuC_Agedup	MuC
9.54E-07	0.2231254	0.375	0.237	0.022868	Cnot4	MuC_Agedup	MuC
1.25E-06	0.2219719	0.289	0.149	0.030042	Apeh	MuC_Agedup	MuC
2.45E-07	0.2219315	0.335	0.184	0.005881	Mphosph6	MuC_Agedup	MuC
7.87E-17	0.2215638	0.206	0.003	1.89E-12	Serpina9	MuC_Agedup	MuC
1.47E-09	0.2214798	0.318	0.133	3.54E-05	Alad	MuC_Agedup	MuC
7.72E-13	0.221459	0.209	0.028	1.85E-08	Galns	MuC_Agedup	MuC
1.71E-07	0.2214453	0.338	0.184	0.0041	Man1b1	MuC_Agedup	MuC
1.24E-07	0.221327	0.275	0.117	0.002976	Tmem107	MuC_Agedup	MuC
4.52E-16	0.2206959	0.189	0	1.08E-11	Sytl2	MuC_Agedup	MuC
5.66E-10	0.2200534	0.246	0.073	1.36E-05	Crebl2	MuC_Agedup	MuC
1.37E-08	0.2198868	0.309	0.139	0.000328	Fsd1l	MuC_Agedup	MuC
2.65E-07	0.2198786	0.338	0.174	0.006357	Limk2	MuC_Agedup	MuC
1.21E-11	0.218845	0.332	0.123	2.90E-07	Rnaset2a	MuC_Agedup	MuC
4.00E-17	0.21836	0.238	0.016	9.60E-13	Podxl	MuC_Agedup	MuC
7.62E-08	0.2177814	0.278	0.12	0.001828	Slc10a7	MuC_Agedup	MuC
2.03E-09	0.2170661	0.252	0.085	4.86E-05	N6amt1	MuC_Agedup	MuC
1.08E-07	0.2169225	0.309	0.149	0.002584	Cep164	MuC_Agedup	MuC
6.49E-08	0.2166605	0.372	0.206	0.001557	Cdk19	MuC_Agedup	MuC
6.67E-07	0.2165503	0.393	0.234	0.015989	2010320M18Rik	MuC_Agedup	MuC
3.09E-07	0.2164014	0.96	0.987	0.007419	Rps4x	MuC_Agedup	MuC
1.69E-06	0.2160926	0.275	0.136	0.040621	Ammecr1	MuC_Agedup	MuC
1.19E-07	0.2159691	0.301	0.133	0.00286	Zmynd8	MuC_Agedup	MuC
1.15E-06	0.2153474	0.341	0.19	0.027619	Rad21	MuC_Agedup	MuC
1.28E-07	0.2150504	0.341	0.184	0.003072	Alg14	MuC_Agedup	MuC
4.77E-11	0.2146002	0.298	0.104	1.14E-06	Tbc1d16	MuC_Agedup	MuC
5.53E-07	0.2138306	0.327	0.184	0.013257	Mrpl10	MuC_Agedup	MuC
9.49E-16	0.2135898	0.215	0.013	2.27E-11	Foxp2	MuC_Agedup	MuC
5.79E-08	0.2135466	0.352	0.18	0.001389	Tmem184a	MuC_Agedup	MuC
5.52E-15	0.2133693	0.235	0.028	1.32E-10	Gm38910	MuC_Agedup	MuC
1.30E-06	0.2127456	0.304	0.155	0.031195	Camta1	MuC_Agedup	MuC
4.83E-09	0.2124929	0.275	0.101	0.000116	Snx18	MuC_Agedup	MuC
9.18E-11	0.2124139	0.986	1	2.20E-06	Rpl13	MuC_Agedup	MuC
1.20E-08	0.211939	0.355	0.171	0.000289	Psmg4	MuC_Agedup	MuC
6.70E-09	0.2101872	0.393	0.215	0.000161	Ptrhd1	MuC_Agedup	MuC
1.91E-06	0.2099323	0.456	0.326	0.045691	Idnk	MuC_Agedup	MuC
3.16E-15	0.2092842	0.951	1	7.57E-11	Wfdc18	MuC_Agedup	MuC
1.88E-07	0.2091421	0.266	0.114	0.004519	Mmp14	MuC_Agedup	MuC

2.33E-08	0.2086333	0.235	0.082	0.000559	Cog2	MuC_Agedup	MuC
1.72E-09	0.2073989	0.195	0.044	4.12E-05	Dennd3	MuC_Agedup	MuC
2.14E-12	0.2073643	0.186	0.019	5.12E-08	B3gnt3	MuC_Agedup	MuC
1.04E-07	0.207314	0.241	0.092	0.002482	Myrip	MuC_Agedup	MuC
4.41E-10	0.2071645	0.264	0.089	1.06E-05	Ldhb	MuC_Agedup	MuC
9.14E-07	0.2071447	0.266	0.123	0.021915	2610528J11Rik	MuC_Agedup	MuC
1.59E-09	0.2069675	0.238	0.073	3.80E-05	Gm34006	MuC_Agedup	MuC
7.33E-10	0.2068448	0.209	0.051	1.76E-05	Pomgnt2	MuC_Agedup	MuC
6.52E-09	0.2064931	0.221	0.066	0.000156	Adcy6	MuC_Agedup	MuC
1.83E-07	0.2062341	0.401	0.237	0.004377	Zc3hav1	MuC_Agedup	MuC
3.63E-07	0.2062111	0.364	0.206	0.008711	Zfp664	MuC_Agedup	MuC
1.14E-09	0.2062038	0.272	0.095	2.75E-05	Cd1d1	MuC_Agedup	MuC
7.29E-09	0.2056221	0.327	0.149	0.000175	Alkbh6	MuC_Agedup	MuC
3.00E-10	0.2051349	0.261	0.082	7.19E-06	Tap2	MuC_Agedup	MuC
2.55E-08	0.2050813	0.338	0.168	0.000612	Asnsd1	MuC_Agedup	MuC
2.71E-08	0.2045917	0.226	0.076	0.00065	Fut2	MuC_Agedup	MuC
3.83E-11	0.2044941	0.198	0.032	9.19E-07	H2-Q7	MuC_Agedup	MuC
1.15E-07	0.2031399	0.281	0.13	0.002762	Zfp511	MuC_Agedup	MuC
4.71E-08	0.2030519	0.209	0.066	0.00113	Ttc7	MuC_Agedup	MuC
5.50E-12	0.2022248	0.232	0.047	1.32E-07	Iqcc	MuC_Agedup	MuC
1.06E-07	0.2021538	0.241	0.092	0.002542	Cog3	MuC_Agedup	MuC
4.36E-08	0.2020334	0.292	0.13	0.001046	Mgrn1	MuC_Agedup	MuC
8.45E-11	0.2010897	0.269	0.082	2.03E-06	H2-Q2	MuC_Agedup	MuC
8.27E-13	0.200302	0.218	0.032	1.98E-08	Tgfb1	MuC_Agedup	MuC
3.90E-10	-0.207249	0.138	0.351	9.36E-06	Gdf5	MuC_Ageddown	MuC
1.15E-12	-0.210885	0.02	0.184	2.76E-08	Lpo	MuC_Ageddown	MuC
5.58E-12	-0.21164	0.026	0.19	1.34E-07	Krt75	MuC_Ageddown	MuC
1.52E-13	-0.218492	0.381	0.753	3.65E-09	Krt4	MuC_Ageddown	MuC
5.73E-15	-0.220333	0.53	0.816	1.37E-10	Fkbp4	MuC_Ageddown	MuC
6.11E-20	-0.220978	0.292	0.699	1.46E-15	4833423E24Rik	MuC_Ageddown	MuC
1.24E-06	-0.221884	0.779	0.934	0.029684	Taldo1	MuC_Ageddown	MuC
2.65E-14	-0.228651	0.269	0.598	6.37E-10	Plin2	MuC_Ageddown	MuC
8.83E-10	-0.235064	0.049	0.206	2.12E-05	Gdf15	MuC_Ageddown	MuC
6.44E-14	-0.239404	0.195	0.475	1.55E-09	Gsta2	MuC_Ageddown	MuC
2.81E-11	-0.240643	0.037	0.203	6.74E-07	Capns2	MuC_Ageddown	MuC
2.77E-10	-0.245417	0.201	0.408	6.65E-06	Hsph1	MuC_Ageddown	MuC
1.25E-16	-0.255951	0.232	0.538	2.99E-12	Psat1	MuC_Ageddown	MuC
1.18E-10	-0.262609	0.04	0.203	2.84E-06	Aqp3	MuC_Ageddown	MuC
2.34E-15	-0.276201	0.112	0.367	5.62E-11	Folr1	MuC_Ageddown	MuC
4.18E-25	-0.281724	0.049	0.37	1.00E-20	Dbp	MuC_Ageddown	MuC
1.20E-07	-0.281846	0.054	0.19	0.002869	Ly6g6c	MuC_Ageddown	MuC
1.28E-24	-0.296305	0.281	0.715	3.07E-20	Elf5	MuC_Ageddown	MuC
2.42E-07	-0.296588	0.077	0.225	0.00581	Tgm3	MuC_Ageddown	MuC
3.82E-37	-0.299699	0.232	0.908	9.16E-33	Tmem59	MuC_Ageddown	MuC
2.51E-17	-0.301545	0.092	0.361	6.01E-13	Chac1	MuC_Ageddown	MuC
8.67E-19	-0.302591	0.413	0.763	2.08E-14	Cald1	MuC_Ageddown	MuC
1.63E-08	-0.305981	0.04	0.177	0.00039	Id3	MuC_Ageddown	MuC
9.39E-15	-0.306986	0.542	0.81	2.25E-10	Socs2	MuC_Ageddown	MuC
1.58E-10	-0.311972	0.183	0.408	3.79E-06	Thrsp	MuC_Ageddown	MuC
3.97E-20	-0.31401	0.255	0.592	9.51E-16	Mast4	MuC_Ageddown	MuC
1.60E-07	-0.318951	0.066	0.209	0.003834	Krt6b	MuC_Ageddown	MuC
2.08E-10	-0.319985	0.281	0.522	4.98E-06	Gadd45b	MuC_Ageddown	MuC
1.84E-20	-0.324794	0.238	0.582	4.42E-16	St3gal6	MuC_Ageddown	MuC
5.09E-14	-0.325424	1	1	1.22E-09	mt-Co3	MuC_Ageddown	MuC
9.21E-18	-0.342217	0.06	0.323	2.21E-13	Stmn1	MuC_Ageddown	MuC
4.26E-07	-0.354321	0.149	0.329	0.010222	Adh7	MuC_Ageddown	MuC
4.35E-07	-0.3545	0.381	0.551	0.010434	Chka	MuC_Ageddown	MuC
2.13E-20	-0.355253	0.223	0.582	5.10E-16	Tcim	MuC_Ageddown	MuC
2.82E-18	-0.369576	0.097	0.415	6.77E-14	Krt15	MuC_Ageddown	MuC

1.10E-12	-0.37463	0.582	0.81	2.63E-08	Dnaja1	MuC_Ageddown	MuC
3.04E-14	-0.380812	0.381	0.763	7.29E-10	Sval2	MuC_Ageddown	MuC
9.07E-08	-0.387675	0.883	0.975	0.002175	Hsp90ab1	MuC_Ageddown	MuC
1.46E-12	-0.389778	1	1	3.51E-08	mt-Atp6	MuC_Ageddown	MuC
7.16E-14	-0.411644	0.077	0.313	1.72E-09	Lgals3	MuC_Ageddown	MuC
1.01E-16	-0.445439	0.129	0.44	2.42E-12	Krt14	MuC_Ageddown	MuC
2.90E-12	-0.457583	0.249	0.557	6.96E-08	Sfn	MuC_Ageddown	MuC
5.49E-12	-0.508189	0.241	0.538	1.32E-07	Dmkn	MuC_Ageddown	MuC
1.49E-09	-0.539997	0.034	0.18	3.57E-05	2300002M23Rik	MuC_Ageddown	MuC
5.55E-11	-0.544371	0.14	0.377	1.33E-06	Calm4	MuC_Ageddown	MuC
2.97E-20	-0.592076	0.53	1	7.13E-16	Dmbt1	MuC_Ageddown	MuC
2.53E-32	-0.596881	0.533	0.943	6.08E-28	Pax1	MuC_Ageddown	MuC
5.09E-20	-0.653759	0.456	0.953	1.22E-15	Prpmp5	MuC_Ageddown	MuC
8.19E-15	-0.654591	0.702	0.88	1.96E-10	Hsp90aa1	MuC_Ageddown	MuC
2.79E-16	-0.658327	0.301	0.677	6.70E-12	Msln	MuC_Ageddown	MuC
1.87E-20	-0.703133	0.59	0.886	4.48E-16	Phlda1	MuC_Ageddown	MuC
4.82E-17	-0.725335	0.355	0.766	1.16E-12	Mt4	MuC_Ageddown	MuC
7.53E-25	-0.818259	0.467	0.93	1.81E-20	Krt13	MuC_Ageddown	MuC
1.94E-23	-0.867167	0.344	0.794	4.65E-19	Fabp5	MuC_Ageddown	MuC
2.37E-21	-0.890161	0.794	1	5.69E-17	Bpifb1	MuC_Ageddown	MuC
2.39E-16	-0.958255	0.272	0.642	5.72E-12	Ly6d	MuC_Ageddown	MuC
1.16E-35	-0.964517	0.453	0.975	2.78E-31	Prb1	MuC_Ageddown	MuC
7.18E-27	-0.999162	0.255	0.731	1.72E-22	Krt6a	MuC_Ageddown	MuC
2.33E-30	-1.063236	0.607	0.987	5.58E-26	2310057J18Rik	MuC_Ageddown	MuC
6.68E-17	-1.066599	0.315	0.706	1.60E-12	Krtdap	MuC_Ageddown	MuC
1.93E-44	-1.109914	0.049	0.535	4.64E-40	Hspa1a	MuC_Ageddown	MuC
3.74E-29	-1.129924	0.309	0.804	8.96E-25	Lgals7	MuC_Ageddown	MuC
3.16E-27	-1.13008	0.361	0.823	7.58E-23	Bpifa2	MuC_Ageddown	MuC
3.22E-36	-1.153921	0.59	1	7.71E-32	Amy1	MuC_Ageddown	MuC
8.10E-37	-1.264773	0.315	0.835	1.94E-32	A630073D07Rik	MuC_Ageddown	MuC
1.62E-48	-1.328893	0.748	0.965	3.87E-44	Ggh	MuC_Ageddown	MuC
7.58E-58	-1.388069	0.095	0.684	1.82E-53	Hspa1b	MuC_Ageddown	MuC
9.56E-39	-1.413344	0.198	0.725	2.29E-34	Hspb1	MuC_Ageddown	MuC
9.33E-66	-1.522051	0.991	1	2.24E-61	Sbpl	MuC_Ageddown	MuC
1.79E-82	-1.684518	0.908	1	4.30E-78	Lipf	MuC_Ageddown	MuC
1.03E-51	-1.848935	0.272	0.892	2.46E-47	Krt17	MuC_Ageddown	MuC
1.92E-93	-4.338075	0.318	0.953	4.61E-89	Gm8882	MuC_Ageddown	MuC
1.00E-10	2.174081	0.841	0.75	2.41E-06	Tmsb10	IC_Agedup	IC
1.40E-09	2.1325204	0.372	0	3.36E-05	Xist	IC_Agedup	IC
1.22E-26	1.9642189	0.97	0.829	2.92E-22	mt-Atp8	IC_Agedup	IC
2.62E-28	1.8861673	1	1	6.28E-24	Gm42418	IC_Agedup	IC
7.72E-11	1.7418546	0.933	0.921	1.85E-06	Crip1	IC_Agedup	IC
4.97E-07	1.7270501	0.61	0.355	0.011913	S100a4	IC_Agedup	IC
6.19E-13	1.6883111	0.713	0.342	1.49E-08	Adgre5	IC_Agedup	IC
8.15E-30	1.6801621	1	0.987	1.95E-25	mt-Nd4l	IC_Agedup	IC
6.43E-12	1.6625021	0.451	0	1.54E-07	Gm10260	IC_Agedup	IC
7.11E-09	1.5730298	0.463	0.105	0.000171	Gm26870	IC_Agedup	IC
2.63E-18	1.2788178	0.994	0.987	6.30E-14	H2-D1	IC_Agedup	IC
1.49E-12	1.2192176	0.97	0.987	3.57E-08	H2-K1	IC_Agedup	IC
1.40E-09	1.0352104	0.372	0	3.36E-05	Tff2	IC_Agedup	IC
4.47E-07	1.0181592	0.64	0.395	0.010713	Arhgap45	IC_Agedup	IC
6.87E-14	1.0095198	0.963	0.961	1.65E-09	Gm10076	IC_Agedup	IC
2.44E-12	0.9781878	1	1	5.86E-08	Malat1	IC_Agedup	IC
3.10E-12	0.9758993	0.909	0.921	7.45E-08	Uba52	IC_Agedup	IC
1.97E-06	0.8367395	0.927	0.947	0.047196	Btg1	IC_Agedup	IC
1.31E-07	0.8288299	1	1	0.003138	Actb	IC_Agedup	IC
1.36E-14	0.7646968	0.976	1	3.26E-10	Rpl38	IC_Agedup	IC
4.79E-17	0.7099573	0.988	1	1.15E-12	Rps28	IC_Agedup	IC
4.27E-09	0.6616191	1	1	0.000102	Rps27	IC_Agedup	IC

2.34E-14	0.6513611	1	1	5.62E-10	Rpl37a	IC_Agedup	IC
8.82E-07	0.6453493	0.909	0.882	0.021147	Rpl27	IC_Agedup	IC
6.77E-14	0.594195	1	1	1.62E-09	Rps29	IC_Agedup	IC
8.07E-08	0.5357801	0.982	1	0.001935	Rpl35	IC_Agedup	IC
1.58E-06	0.5210759	0.994	0.987	0.037871	Pfn1	IC_Agedup	IC
1.42E-06	0.4939276	0.976	0.987	0.034092	Serf2	IC_Agedup	IC
9.79E-07	0.3674052	0.988	1	0.023469	Rpl37	IC_Agedup	IC
8.04E-07	-0.239129	0.012	0.184	0.019273	Bcl7a	IC_Ageddown	IC
1.44E-06	-0.243268	0.018	0.197	0.034639	Id4	IC_Ageddown	IC
1.16E-06	-0.245709	0.018	0.197	0.027791	Rassf9	IC_Ageddown	IC
2.25E-07	-0.306723	0.067	0.329	0.005405	Gtf2f1	IC_Ageddown	IC
1.41E-06	-0.3108	0.146	0.461	0.033911	Eif2a	IC_Ageddown	IC
2.02E-07	-0.315667	0.012	0.197	0.004848	Prr15	IC_Ageddown	IC
4.05E-07	-0.317031	0.146	0.487	0.009717	Ndufa9	IC_Ageddown	IC
6.24E-07	-0.32979	0.128	0.434	0.014954	Gadd45gip1	IC_Ageddown	IC
1.57E-06	-0.3328	0.024	0.211	0.037735	Tmprss4	IC_Ageddown	IC
1.11E-06	-0.33712	0.018	0.197	0.026588	Nqo1	IC_Ageddown	IC
3.55E-07	-0.338731	0.024	0.224	0.008516	Paqr5	IC_Ageddown	IC
5.68E-07	-0.370451	1	1	0.01362	Rps16	IC_Ageddown	IC
1.08E-06	-0.379622	1	1	0.025976	Rpl23	IC_Ageddown	IC
6.29E-07	-0.386025	0.037	0.25	0.015091	Foxe1	IC_Ageddown	IC
1.74E-06	-0.386926	0.976	1	0.041836	Rps4x	IC_Ageddown	IC
2.03E-07	-0.388429	0.098	0.395	0.004861	Wdr89	IC_Ageddown	IC
2.53E-07	-0.389168	0.982	1	0.006059	Rpl32	IC_Ageddown	IC
1.86E-06	-0.399505	0.024	0.211	0.044553	Mertk	IC_Ageddown	IC
3.95E-07	-0.401117	0.024	0.224	0.009481	Kif21a	IC_Ageddown	IC
9.90E-07	-0.402867	0.128	0.434	0.023749	Tmem33	IC_Ageddown	IC
1.00E-06	-0.413376	0.122	0.408	0.024005	Utp3	IC_Ageddown	IC
1.92E-06	-0.433026	0.146	0.434	0.046044	Fuca2	IC_Ageddown	IC
3.81E-07	-0.434556	0.018	0.211	0.009142	Snhg18	IC_Ageddown	IC
7.39E-07	-0.436478	0.073	0.329	0.017722	Tns1	IC_Ageddown	IC
1.67E-07	-0.443581	0.177	0.513	0.004014	Psmc6	IC_Ageddown	IC
1.42E-07	-0.452161	0.232	0.605	0.003406	Sumo3	IC_Ageddown	IC
1.69E-06	-0.458423	0.006	0.158	0.04054	Ces1f	IC_Ageddown	IC
1.08E-07	-0.464084	0.134	0.447	0.002596	Arpc1a	IC_Ageddown	IC
4.71E-09	-0.474722	0.982	1	0.000113	Rpsa	IC_Ageddown	IC
9.90E-07	-0.479985	0.061	0.289	0.023736	Cbr2	IC_Ageddown	IC
4.57E-10	-0.486368	0.024	0.289	1.10E-05	Ccdc86	IC_Ageddown	IC
8.55E-07	-0.487531	0.915	1	0.020506	Rps6	IC_Ageddown	IC
5.73E-07	-0.490376	0.634	0.921	0.013741	Hnrnpf	IC_Ageddown	IC
1.77E-06	-0.491995	0.232	0.553	0.042381	Mtch1	IC_Ageddown	IC
6.13E-07	-0.496893	0.049	0.276	0.014696	Gja1	IC_Ageddown	IC
9.29E-07	-0.49835	0.451	0.789	0.02228	Eif2s2	IC_Ageddown	IC
5.70E-09	-0.499725	0.976	1	0.000137	Rpl13	IC_Ageddown	IC
3.40E-07	-0.512407	0.945	1	0.00815	Ubb	IC_Ageddown	IC
1.68E-08	-0.512423	0.988	1	0.000402	Rpl28	IC_Ageddown	IC
3.51E-08	-0.51343	0.067	0.355	0.000841	Mcub	IC_Ageddown	IC
9.03E-07	-0.520917	0.183	0.487	0.021645	Atp13a3	IC_Ageddown	IC
2.76E-11	-0.5298	0.03	0.342	6.62E-07	Plxdc2	IC_Ageddown	IC
5.09E-08	-0.535648	0.049	0.303	0.00122	Tst	IC_Ageddown	IC
3.73E-09	-0.542919	0.287	0.75	8.94E-05	Eif3m	IC_Ageddown	IC
1.68E-11	-0.545677	1	1	4.02E-07	Tpt1	IC_Ageddown	IC
9.04E-08	-0.547678	0.159	0.487	0.002168	Ube2e3	IC_Ageddown	IC
2.27E-10	-0.549927	0.085	0.447	5.45E-06	Ogfrl1	IC_Ageddown	IC
9.29E-10	-0.550036	1	1	2.23E-05	mt-Nd2	IC_Ageddown	IC
1.61E-06	-0.552805	0.25	0.592	0.038502	Ccni	IC_Ageddown	IC
4.88E-08	-0.554034	0.963	1	0.001171	Rpl7	IC_Ageddown	IC
1.55E-06	-0.562011	0.043	0.25	0.037103	Anxa8	IC_Ageddown	IC
1.38E-06	-0.562477	0.366	0.776	0.03306	Glul	IC_Ageddown	IC

5.60E-08	-0.564345	0.067	0.342	0.001343	Pitx1	IC_Ageddown	IC
5.17E-07	-0.566306	0.207	0.539	0.012403	Krcc1	IC_Ageddown	IC
2.28E-10	-0.566315	0.963	1	5.47E-06	Rps18	IC_Ageddown	IC
1.42E-09	-0.57179	0.945	1	3.40E-05	Rpl10	IC_Ageddown	IC
2.52E-07	-0.578158	0.28	0.645	0.006051	Ybx3	IC_Ageddown	IC
1.71E-11	-0.578878	0.982	1	4.10E-07	Rpl17	IC_Ageddown	IC
9.87E-08	-0.580372	0.152	0.474	0.002367	Rraga	IC_Ageddown	IC
1.18E-08	-0.581124	0.134	0.474	0.000283	Lsm2	IC_Ageddown	IC
2.90E-07	-0.586662	0.256	0.618	0.006963	Comt	IC_Ageddown	IC
1.52E-08	-0.590318	0.256	0.645	0.000365	Gnaq	IC_Ageddown	IC
8.04E-08	-0.592283	0.36	0.737	0.001927	Cct4	IC_Ageddown	IC
8.65E-11	-0.595003	0.97	1	2.07E-06	Rpl15	IC_Ageddown	IC
6.46E-07	-0.595602	0.25	0.592	0.015482	Cacybp	IC_Ageddown	IC
4.13E-12	-0.599863	0.982	1	9.91E-08	Eef1a1	IC_Ageddown	IC
3.91E-07	-0.608696	0.183	0.513	0.009366	Tceal9	IC_Ageddown	IC
4.91E-09	-0.615101	0.244	0.711	0.000118	Fkbp5	IC_Ageddown	IC
1.88E-10	-0.623186	0.915	0.987	4.51E-06	Rack1	IC_Ageddown	IC
1.06E-10	-0.626363	0.055	0.382	2.55E-06	Smagp	IC_Ageddown	IC
4.66E-09	-0.631144	0.348	0.737	0.000112	Cct7	IC_Ageddown	IC
2.06E-09	-0.633911	0.11	0.461	4.95E-05	Slamf7	IC_Ageddown	IC
2.34E-07	-0.634025	0.39	0.763	0.005619	Arf4	IC_Ageddown	IC
8.23E-07	-0.647069	0.128	0.421	0.019735	Ccdc34	IC_Ageddown	IC
9.54E-12	-0.663115	0.915	1	2.29E-07	Rpl29	IC_Ageddown	IC
2.43E-16	-0.667611	0.976	1	5.82E-12	Rplp1	IC_Ageddown	IC
8.74E-07	-0.668816	0.348	0.711	0.020961	Itgb5	IC_Ageddown	IC
2.22E-08	-0.673604	0.055	0.329	0.000532	Ptprf	IC_Ageddown	IC
1.64E-08	-0.674497	0	0.184	0.000393	Gm8882	IC_Ageddown	IC
1.30E-09	-0.674713	0.89	0.974	3.11E-05	Cox4i1	IC_Ageddown	IC
4.48E-19	-0.681493	1	1	1.08E-14	mt-Cytb	IC_Ageddown	IC
9.04E-07	-0.682443	0.543	0.829	0.021672	Calr	IC_Ageddown	IC
1.34E-06	-0.692335	0.165	0.461	0.032057	MuClm	IC_Ageddown	IC
1.40E-07	-0.692711	0.232	0.566	0.003357	Pnp	IC_Ageddown	IC
4.31E-12	-0.700538	0.976	1	1.03E-07	Rplp0	IC_Ageddown	IC
4.65E-11	-0.704375	0.22	0.671	1.11E-06	Fkbp4	IC_Ageddown	IC
2.38E-10	-0.705336	0.104	0.461	5.70E-06	Mndal	IC_Ageddown	IC
4.38E-08	-0.705625	0.585	0.947	0.001049	Erp29	IC_Ageddown	IC
3.45E-07	-0.705836	0.073	0.329	0.008269	Serpinb5	IC_Ageddown	IC
2.01E-07	-0.714821	0.195	0.553	0.004819	Ctsl	IC_Ageddown	IC
7.63E-19	-0.717074	1	1	1.83E-14	mt-Co3	IC_Ageddown	IC
2.39E-09	-0.717568	0.293	0.711	5.73E-05	Mat2a	IC_Ageddown	IC
5.94E-08	-0.722607	0.012	0.211	0.001425	Sptssb	IC_Ageddown	IC
2.15E-17	-0.727337	0.97	1	5.15E-13	Rpl26	IC_Ageddown	IC
6.14E-14	-0.736681	0.915	0.987	1.47E-09	H3f3a	IC_Ageddown	IC
3.01E-10	-0.743835	0.817	0.974	7.21E-06	Rps17	IC_Ageddown	IC
2.37E-09	-0.753202	0.64	0.961	5.69E-05	Pcbp2	IC_Ageddown	IC
1.49E-06	-0.762306	0.555	0.842	0.035818	Marcks	IC_Ageddown	IC
1.38E-06	-0.774886	0.274	0.566	0.033184	Tcf4	IC_Ageddown	IC
4.91E-07	-0.776994	0.28	0.618	0.01178	Cks2	IC_Ageddown	IC
1.97E-06	-0.782648	0.049	0.25	0.047264	Sox2	IC_Ageddown	IC
2.39E-09	-0.785979	0.055	0.355	5.74E-05	Nfib	IC_Ageddown	IC
6.17E-08	-0.788554	0.055	0.316	0.00148	Trp63	IC_Ageddown	IC
7.42E-11	-0.802504	0.28	0.697	1.78E-06	P4hb	IC_Ageddown	IC
2.26E-18	-0.81524	0.945	1	5.41E-14	Rpl21	IC_Ageddown	IC
4.58E-18	-0.821396	1	1	1.10E-13	Rps14	IC_Ageddown	IC
2.53E-27	-0.859095	1	1	6.08E-23	mt-Atp6	IC_Ageddown	IC
5.29E-09	-0.874794	0.671	0.934	0.000127	Hspe1	IC_Ageddown	IC
1.04E-07	-0.879891	0.299	0.632	0.002488	Skp1a	IC_Ageddown	IC
2.46E-09	-0.881172	0.335	0.724	5.91E-05	Hmgn1	IC_Ageddown	IC
4.19E-09	-0.894548	0.067	0.368	0.0001	Id1	IC_Ageddown	IC

3.54E-08	-0.912088	0.098	0.395	0.000848	Klf5	IC_Ageddown	IC
6.74E-08	-0.924463	0.061	0.316	0.001617	Ces1d	IC_Ageddown	IC
1.34E-17	-0.942599	0.201	0.829	3.20E-13	Tmem59	IC_Ageddown	IC
3.38E-23	-0.953911	0.988	1	8.12E-19	Rps8	IC_Ageddown	IC
1.67E-18	-0.976298	0.933	1	4.00E-14	Rps25	IC_Ageddown	IC
1.79E-08	-1.025817	0.03	0.276	0.000429	Tgm3	IC_Ageddown	IC
7.73E-19	-1.040359	0.927	1	1.85E-14	Hspa8	IC_Ageddown	IC
1.55E-14	-1.044342	0.738	0.974	3.71E-10	Rpl23a	IC_Ageddown	IC
9.27E-08	-1.049103	0.476	0.829	0.002222	Cd9	IC_Ageddown	IC
4.27E-19	-1.059313	0.079	0.618	1.02E-14	Hsph1	IC_Ageddown	IC
1.01E-09	-1.061039	0.079	0.408	2.42E-05	Rab25	IC_Ageddown	IC
6.22E-17	-1.09168	0.866	1	1.49E-12	Rps15	IC_Ageddown	IC
9.15E-21	-1.108065	0.909	0.987	2.19E-16	Rpl12	IC_Ageddown	IC
8.69E-22	-1.134822	0.933	1	2.08E-17	Rps12	IC_Ageddown	IC
1.14E-09	-1.158373	0.024	0.289	2.73E-05	Krt75	IC_Ageddown	IC
8.36E-09	-1.17771	0.043	0.303	0.0002	Id3	IC_Ageddown	IC
3.48E-07	-1.204767	0.183	0.5	0.00835	Sfn	IC_Ageddown	IC
6.15E-09	-1.220501	0.256	0.645	0.000147	Hspa1a	IC_Ageddown	IC
1.37E-08	-1.22453	0.238	0.566	0.000329	Tubb4b	IC_Ageddown	IC
1.26E-09	-1.271898	0.085	0.408	3.03E-05	S100a14	IC_Ageddown	IC
3.73E-07	-1.273078	0.409	0.803	0.008953	Krt13	IC_Ageddown	IC
2.04E-09	-1.327347	0.152	0.513	4.90E-05	Fxyd3	IC_Ageddown	IC
1.25E-07	-1.388071	0.201	0.526	0.003001	Gsta4	IC_Ageddown	IC
3.76E-26	-1.423135	0.866	1	9.01E-22	Hsp90ab1	IC_Ageddown	IC
4.00E-08	-1.429358	0.14	0.461	0.000959	Perp	IC_Ageddown	IC
6.34E-08	-1.501114	0.122	0.434	0.00152	Adh7	IC_Ageddown	IC
1.59E-08	-1.505964	0.14	0.487	0.000381	Krt5	IC_Ageddown	IC
6.32E-09	-1.511847	0.104	0.421	0.000151	Dsp	IC_Ageddown	IC
2.77E-19	-1.586374	0.61	0.961	6.64E-15	Hsp90aa1	IC_Ageddown	IC
7.78E-07	-1.617369	0.305	0.592	0.018648	Gsto1	IC_Ageddown	IC
2.11E-08	-1.63931	0.134	0.461	0.000506	Gpx2	IC_Ageddown	IC
1.61E-10	-1.668498	0.22	0.658	3.85E-06	Ly6d	IC_Ageddown	IC
7.53E-28	-1.740287	0.079	0.75	1.81E-23	Hpgd	IC_Ageddown	IC
5.79E-10	-1.750186	0.024	0.289	1.39E-05	Ptn	IC_Ageddown	IC
1.44E-19	-1.859102	0.11	0.645	3.46E-15	Hspa1b	IC_Ageddown	IC
9.18E-08	-1.915994	0.78	0.974	0.002202	Lipf	IC_Ageddown	IC
7.13E-08	-1.937199	0.091	0.382	0.001709	Krt15	IC_Ageddown	IC
2.80E-10	-1.973095	0.774	0.987	6.71E-06	Sbpl	IC_Ageddown	IC
5.45E-09	-2.036539	0.22	0.632	0.000131	Krt6a	IC_Ageddown	IC
1.79E-10	-2.076908	0.305	0.763	4.30E-06	Lgals7	IC_Ageddown	IC
7.00E-14	-2.285314	0.189	0.684	1.68E-09	Hspb1	IC_Ageddown	IC
8.10E-16	-3.287756	0.238	0.763	1.94E-11	Krt17	IC_Ageddown	IC
7.64E-22	3.2275018	1	0.984	1.83E-17	Gm42418	MC_Agedup	MC
1.45E-17	1.8185999	0.986	0.952	3.48E-13	mt-Nd4l	MC_Agedup	MC
5.62E-14	1.5963565	1	0.919	1.35E-09	Gm10076	MC_Agedup	MC
1.42E-07	1.5356449	0.757	0.629	0.003409	mt-Atp8	MC_Agedup	MC
2.08E-11	1.1924554	1	0.984	4.99E-07	Rps28	MC_Agedup	MC
1.41E-11	1.1129734	0.986	0.984	3.37E-07	Rpl38	MC_Agedup	MC
7.57E-14	1.0735466	1	0.984	1.81E-09	Rps29	MC_Agedup	MC
1.22E-08	0.9552812	0.986	0.968	0.000292	Rpl35	MC_Agedup	MC
2.95E-09	0.942566	1	1	7.08E-05	Rpl37a	MC_Agedup	MC
1.00E-06	-0.232886	0.057	0.452	0.02404	Itgb4	MC_Ageddown	MC
1.94E-07	-0.360236	0.057	0.484	0.004644	Bcl11b	MC_Ageddown	MC
1.77E-06	-0.427312	0.043	0.403	0.042525	Ube2f	MC_Ageddown	MC
1.16E-07	-0.499128	1	1	0.00277	mt-Co3	MC_Ageddown	MC
1.47E-06	-0.52995	0.014	0.323	0.035138	Tanc1	MC_Ageddown	MC
1.08E-06	-0.568144	0.114	0.565	0.026014	Spint2	MC_Ageddown	MC
5.22E-10	-0.584971	1	1	1.25E-05	mt-Cytb	MC_Ageddown	MC
1.50E-06	-0.594182	0	0.29	0.035926	Hmgb3	MC_Ageddown	MC

3.71E-07	-0.600438	0.186	0.71	0.008887	Avpi1	MC_Ageddown	MC
6.58E-07	-0.608462	0.014	0.339	0.015778	Arap2	MC_Ageddown	MC
1.44E-06	-0.614686	0.986	1	0.034526	Rps8	MC_Ageddown	MC
1.41E-06	-0.620879	0.143	0.613	0.033793	Sox2	MC_Ageddown	MC
8.14E-07	-0.628953	0.086	0.484	0.019531	Cldnd1	MC_Ageddown	MC
2.39E-07	-0.638856	0.086	0.548	0.005731	Wnt4	MC_Ageddown	MC
2.02E-06	-0.646131	0.114	0.516	0.048477	Nab1	MC_Ageddown	MC
3.38E-07	-0.658864	0.014	0.355	0.008109	Vgll4	MC_Ageddown	MC
6.05E-07	-0.660321	0.1	0.516	0.014498	Tnfaip8	MC_Ageddown	MC
9.96E-07	-0.672999	0.043	0.403	0.023876	Fgfr2	MC_Ageddown	MC
1.07E-06	-0.690253	0.029	0.355	0.025711	Ahsa2	MC_Ageddown	MC
2.83E-07	-0.724613	0.929	0.984	0.006793	Rpl21	MC_Ageddown	MC
1.61E-06	-0.72775	0.029	0.355	0.038709	Emb	MC_Ageddown	MC
1.87E-06	-0.735025	0.129	0.532	0.044942	Nr1d2	MC_Ageddown	MC
8.74E-08	-0.743296	0.071	0.516	0.002095	Slc12a2	MC_Ageddown	MC
4.70E-09	-0.746934	0.029	0.468	0.000113	F11r	MC_Ageddown	MC
1.16E-07	-0.769766	0.814	0.968	0.002781	H3f3a	MC_Ageddown	MC
1.50E-06	-0.771707	0	0.29	0.035926	Cdh13	MC_Ageddown	MC
1.30E-06	-0.781081	0.9	1	0.031091	Rpl12	MC_Ageddown	MC
4.81E-07	-0.801944	0.071	0.5	0.011544	Phgdh	MC_Ageddown	MC
1.92E-08	-0.807878	0.071	0.532	0.000461	Igsf3	MC_Ageddown	MC
7.48E-09	-0.811961	0.043	0.516	0.000179	Dsc3	MC_Ageddown	MC
8.31E-07	-0.81814	0.1	0.532	0.019937	Hmgn3	MC_Ageddown	MC
7.20E-07	-0.84126	0.043	0.419	0.017261	Cp	MC_Ageddown	MC
1.03E-07	-0.841941	0.143	0.613	0.002473	Ptprf	MC_Ageddown	MC
2.41E-08	-0.847181	0.043	0.468	0.000578	Snai2	MC_Ageddown	MC
6.71E-07	-0.854859	0.186	0.661	0.016097	MuClm	MC_Ageddown	MC
3.30E-09	-0.865167	0.029	0.484	7.91E-05	Mgst2	MC_Ageddown	MC
5.73E-07	-0.865205	0.043	0.419	0.013748	Bcam	MC_Ageddown	MC
4.78E-08	-0.871132	0.029	0.435	0.001146	4631405K08Rik	MC_Ageddown	MC
4.35E-08	-0.874812	0.029	0.419	0.001042	Tmem132a	MC_Ageddown	MC
1.74E-06	-0.875066	0.171	0.629	0.041825	Id1	MC_Ageddown	MC
9.35E-07	-0.895586	0.243	0.71	0.022428	2010111I01Rik	MC_Ageddown	MC
5.91E-08	-0.901797	0.071	0.484	0.001417	Nectin1	MC_Ageddown	MC
1.54E-09	-0.904494	0.8	0.968	3.70E-05	Cox4i1	MC_Ageddown	MC
6.23E-08	-0.911882	0.014	0.387	0.001493	Pdgfa	MC_Ageddown	MC
5.62E-08	-0.913546	0.071	0.532	0.001347	Epcam	MC_Ageddown	MC
1.50E-06	-0.945017	0	0.29	0.035926	Ptprz1	MC_Ageddown	MC
5.95E-07	-0.961445	0.214	0.661	0.014276	Psma4	MC_Ageddown	MC
4.67E-20	-0.966774	1	1	1.12E-15	mt-Atp6	MC_Ageddown	MC
1.97E-07	-0.96849	0.229	0.726	0.004715	Atp1b3	MC_Ageddown	MC
3.70E-07	-0.983327	0.114	0.548	0.008873	Sox4	MC_Ageddown	MC
5.64E-08	-0.984897	0.229	0.774	0.001353	Dsp	MC_Ageddown	MC
1.25E-07	-0.98492	0.114	0.565	0.002994	Gadd45g	MC_Ageddown	MC
8.12E-09	-0.989172	0.086	0.548	0.000195	Hsph1	MC_Ageddown	MC
2.49E-07	-0.99002	0.329	0.79	0.005961	Ccnd2	MC_Ageddown	MC
2.02E-09	-1.002225	0.843	0.968	4.84E-05	Hsp90ab1	MC_Ageddown	MC
1.41E-07	-1.003505	0.1	0.532	0.003376	Slc3a2	MC_Ageddown	MC
1.37E-07	-1.047665	0.129	0.613	0.003278	Trp63	MC_Ageddown	MC
3.15E-09	-1.053476	0.743	1	7.56E-05	Ubb	MC_Ageddown	MC
1.54E-06	-1.090746	0.529	0.855	0.036882	Hsp90aa1	MC_Ageddown	MC
1.25E-09	-1.096368	0.1	0.645	3.00E-05	Slc6a6	MC_Ageddown	MC
7.04E-09	-1.149503	0.029	0.468	0.000169	Sox9	MC_Ageddown	MC
2.99E-13	-1.149703	0.743	0.984	7.18E-09	Hspa8	MC_Ageddown	MC
5.46E-09	-1.183096	0	0.403	0.000131	Slc5a3	MC_Ageddown	MC
4.19E-07	-1.215244	0.071	0.468	0.010055	Cited2	MC_Ageddown	MC
1.25E-07	-1.238196	0.1	0.532	0.002987	Ces1d	MC_Ageddown	MC
1.13E-07	-1.256395	0.271	0.694	0.002721	Atp1a1	MC_Ageddown	MC
5.31E-08	-1.258855	0.271	0.806	0.001274	Perp	MC_Ageddown	MC

1.83E-10	-1.294821	0.057	0.613	4.40E-06	Col17a1	MC_Ageddown	MC
7.36E-09	-1.296169	0.371	0.855	0.000177	Fxyd3	MC_Ageddown	MC
8.61E-11	-1.36342	0.029	0.548	2.06E-06	Itga6	MC_Ageddown	MC
5.19E-07	-1.396395	0.086	0.484	0.012452	Wfdc2	MC_Ageddown	MC
7.13E-07	-1.427861	0.314	0.742	0.017106	Tsc22d3	MC_Ageddown	MC
3.37E-09	-1.452454	0.029	0.468	8.07E-05	Stmn1	MC_Ageddown	MC
1.45E-06	-1.494213	0.171	0.581	0.034701	Dusp1	MC_Ageddown	MC
3.91E-07	-1.51599	0.014	0.355	0.009379	Tnc	MC_Ageddown	MC
2.43E-07	-1.568299	0.143	0.597	0.005824	Ier3	MC_Ageddown	MC
7.51E-13	-1.641761	0.586	0.919	1.80E-08	Cd9	MC_Ageddown	MC
1.50E-06	-1.679958	0	0.29	0.035926	Gm8882	MC_Ageddown	MC
9.48E-15	-1.680195	0.043	0.694	2.27E-10	Tmem59	MC_Ageddown	MC
2.58E-12	-1.735491	0.086	0.677	6.19E-08	Nfkbia	MC_Ageddown	MC
1.51E-07	-1.793407	0.086	0.516	0.003631	Igfbp2	MC_Ageddown	MC
8.74E-08	-1.81186	0.129	0.581	0.002095	Zfp36	MC_Ageddown	MC
1.34E-06	-1.932938	0.1	0.484	0.032242	Atf3	MC_Ageddown	MC
8.38E-10	-2.067059	0.029	0.5	2.01E-05	Ptn	MC_Ageddown	MC
1.80E-06	-2.085722	0.243	0.629	0.043142	Egr1	MC_Ageddown	MC
4.30E-08	-2.39786	0.114	0.565	0.001032	Krt15	MC_Ageddown	MC
3.03E-10	-2.744347	0.171	0.694	7.26E-06	Krt14	MC_Ageddown	MC
1.72E-07	-2.766367	0.657	0.984	0.004131	Sbpl	MC_Ageddown	MC
6.41E-10	-2.883511	0.2	0.726	1.54E-05	Fos	MC_Ageddown	MC
4.23E-70	3.139142	0.588	0.002	1.01E-65	Xist	MTC_Agedup	MTC
6.30E-36	2.9238093	0.328	0.004	1.51E-31	Tff2	MTC_Agedup	MTC
4.28E-11	2.2811637	0.378	0.129	1.03E-06	Gng13	MTC_Agedup	MTC
2.61E-17	2.1960768	0.261	0.031	6.27E-13	Muc5b	MTC_Agedup	MTC
2.53E-36	2.0021018	0.966	0.842	6.07E-32	Gm42418	MTC_Agedup	MTC
4.27E-42	1.9311175	0.924	0.565	1.02E-37	mt-Atp8	MTC_Agedup	MTC
5.57E-07	1.5347913	0.244	0.087	0.013357	Plcb2	MTC_Agedup	MTC
3.68E-48	1.4596005	0.992	0.958	8.83E-44	mt-Nd4l	MTC_Agedup	MTC
4.13E-07	1.3595785	0.218	0.069	0.0099	Ifi27l2a	MTC_Agedup	MTC
1.46E-14	1.3109571	0.849	0.707	3.50E-10	Crip1	MTC_Agedup	MTC
2.59E-39	1.2439399	1	0.95	6.22E-35	Gm10076	MTC_Agedup	MTC
4.07E-28	1.1781774	0.277	0.008	9.76E-24	Gm10260	MTC_Agedup	MTC
1.30E-27	1.0485251	0.992	0.881	3.11E-23	Uqcr11	MTC_Agedup	MTC
4.95E-09	1.0287665	0.504	0.254	0.000119	Etv1	MTC_Agedup	MTC
1.27E-16	1.018612	0.748	0.468	3.05E-12	Erh	MTC_Agedup	MTC
8.76E-08	0.9504764	0.412	0.202	0.0021	Itpr3	MTC_Agedup	MTC
6.37E-17	0.9414632	0.319	0.06	1.53E-12	Gm26870	MTC_Agedup	MTC
3.20E-21	0.9301756	0.874	0.565	7.68E-17	Ndufa3	MTC_Agedup	MTC
8.45E-08	0.9177101	0.697	0.518	0.002026	Dusp1	MTC_Agedup	MTC
9.56E-21	0.8909	0.95	0.775	2.29E-16	Usmg5	MTC_Agedup	MTC
7.46E-07	0.8863877	0.597	0.416	0.01789	Cdkn1a	MTC_Agedup	MTC
1.63E-07	0.8861373	0.664	0.484	0.003918	Abhd2	MTC_Agedup	MTC
4.43E-15	0.8739665	0.454	0.146	1.06E-10	Arrdc3	MTC_Agedup	MTC
2.75E-09	0.8308223	0.622	0.358	6.60E-05	Smim22	MTC_Agedup	MTC
8.00E-08	0.823787	0.63	0.422	0.001919	H1f0	MTC_Agedup	MTC
1.09E-06	0.8076025	0.21	0.067	0.026077	Tnfaip2	MTC_Agedup	MTC
9.63E-15	0.7836869	0.916	0.759	2.31E-10	Romo1	MTC_Agedup	MTC
5.26E-11	0.7819164	0.832	0.611	1.26E-06	H2-D1	MTC_Agedup	MTC
1.41E-09	0.7789744	0.765	0.593	3.37E-05	Ndufv3	MTC_Agedup	MTC
1.02E-10	0.7787993	0.412	0.154	2.44E-06	Ceacam1	MTC_Agedup	MTC
7.02E-18	0.7784526	0.294	0.042	1.68E-13	Akap12	MTC_Agedup	MTC
4.56E-14	0.7719141	0.983	0.981	1.09E-09	Malat1	MTC_Agedup	MTC
1.05E-12	0.760333	0.748	0.499	2.52E-08	Cox17	MTC_Agedup	MTC
4.63E-18	0.754327	0.891	0.717	1.11E-13	Ndufa1	MTC_Agedup	MTC
2.17E-16	0.7543163	0.672	0.337	5.20E-12	Pet100	MTC_Agedup	MTC
3.77E-07	0.7485526	0.714	0.557	0.009042	1810058I24Rik	MTC_Agedup	MTC
3.66E-16	0.7471409	1	0.983	8.77E-12	Rps28	MTC_Agedup	MTC

4.11E-11	0.741164	0.622	0.345	9.86E-07	Gstp1	MTC_Agedup	MTC
1.19E-06	0.7315544	0.496	0.279	0.028579	Ociad2	MTC_Agedup	MTC
8.48E-12	0.7307448	0.513	0.233	2.03E-07	Grcc10	MTC_Agedup	MTC
1.22E-14	0.7220553	0.933	0.823	2.93E-10	Uba52	MTC_Agedup	MTC
1.10E-14	0.7182055	0.874	0.717	2.63E-10	Ost4	MTC_Agedup	MTC
3.72E-27	0.714405	0.252	0.004	8.92E-23	Ifi203	MTC_Agedup	MTC
2.63E-12	0.7112165	0.824	0.642	6.30E-08	Ndufb2	MTC_Agedup	MTC
5.80E-08	0.7064844	0.462	0.245	0.001391	Tmem245	MTC_Agedup	MTC
2.38E-29	0.7056014	1	0.988	5.70E-25	Rpl38	MTC_Agedup	MTC
4.08E-15	0.7043815	0.975	0.933	9.78E-11	Rpl35	MTC_Agedup	MTC
1.49E-15	0.7034041	1	0.927	3.57E-11	Uqcr10	MTC_Agedup	MTC
6.62E-08	0.7000729	0.723	0.538	0.001587	Tbx3	MTC_Agedup	MTC
2.20E-19	0.6898026	0.966	0.875	5.27E-15	Tomm7	MTC_Agedup	MTC
8.25E-09	0.6829644	0.706	0.462	0.000198	Sap18	MTC_Agedup	MTC
6.43E-07	0.676021	0.538	0.341	0.015424	Klf10	MTC_Agedup	MTC
1.03E-12	0.6753679	0.891	0.742	2.47E-08	Son	MTC_Agedup	MTC
2.40E-12	0.668196	0.958	0.832	5.76E-08	Ndufa7	MTC_Agedup	MTC
4.24E-12	0.664577	0.807	0.586	1.02E-07	Mrps21	MTC_Agedup	MTC
3.22E-11	0.655161	0.471	0.193	7.71E-07	Sema3f	MTC_Agedup	MTC
7.00E-08	0.6541243	0.126	0.019	0.001679	Gm26829	MTC_Agedup	MTC
2.35E-12	0.6514737	0.807	0.58	5.63E-08	Bola2	MTC_Agedup	MTC
2.31E-08	0.6471923	0.235	0.067	0.000554	Ano10	MTC_Agedup	MTC
1.32E-10	0.6464123	0.924	0.813	3.16E-06	Rpl27	MTC_Agedup	MTC
4.81E-25	0.6414922	1	0.994	1.15E-20	Rps29	MTC_Agedup	MTC
2.77E-10	0.6386459	0.908	0.757	6.63E-06	Atp1a1	MTC_Agedup	MTC
2.72E-18	0.6329197	0.983	0.96	6.51E-14	Cox7c	MTC_Agedup	MTC
1.56E-06	0.6321521	0.95	0.963	0.037527	Tmsb10	MTC_Agedup	MTC
7.74E-11	0.6318044	0.731	0.451	1.86E-06	Ktn1	MTC_Agedup	MTC
4.73E-09	0.6317719	0.756	0.586	0.000113	Itgb1	MTC_Agedup	MTC
6.30E-07	0.6311104	0.655	0.439	0.01511	Txnip	MTC_Agedup	MTC
2.27E-10	0.6286564	0.79	0.576	5.45E-06	Tmem256	MTC_Agedup	MTC
1.36E-08	0.6165002	0.765	0.528	0.000326	Avil	MTC_Agedup	MTC
1.50E-18	0.6061665	0.966	0.95	3.59E-14	Cox6c	MTC_Agedup	MTC
4.47E-09	0.604647	0.79	0.59	0.000107	Snrpg	MTC_Agedup	MTC
1.76E-06	0.6022112	0.395	0.202	0.042275	Snhg20	MTC_Agedup	MTC
4.28E-09	0.5982002	0.538	0.289	0.000103	Sptbn2	MTC_Agedup	MTC
8.90E-11	0.5967073	0.966	0.852	2.13E-06	H2afj	MTC_Agedup	MTC
2.28E-19	0.593083	0.378	0.069	5.46E-15	Snhg9	MTC_Agedup	MTC
1.89E-08	0.583218	0.857	0.782	0.000453	Mrpl52	MTC_Agedup	MTC
9.12E-07	0.5818275	0.546	0.349	0.021869	Setd5	MTC_Agedup	MTC
6.25E-07	0.5811559	0.857	0.713	0.014984	Ndufa6	MTC_Agedup	MTC
1.37E-10	0.5785432	0.882	0.732	3.29E-06	Atp5k	MTC_Agedup	MTC
1.93E-07	0.5773572	0.714	0.489	0.004621	Pam16	MTC_Agedup	MTC
1.85E-07	0.5757719	0.908	0.842	0.004433	1810037I17Rik	MTC_Agedup	MTC
2.71E-08	0.5737821	0.496	0.247	0.000651	Clmn	MTC_Agedup	MTC
6.87E-12	0.5696299	0.966	0.915	1.65E-07	mt-Nd5	MTC_Agedup	MTC
4.75E-12	0.5663198	0.933	0.875	1.14E-07	Spint2	MTC_Agedup	MTC
6.18E-07	0.5623365	0.824	0.694	0.01482	Ctsd	MTC_Agedup	MTC
1.13E-09	0.5542431	0.21	0.046	2.71E-05	Ocel1	MTC_Agedup	MTC
7.54E-07	0.5505861	0.21	0.064	0.018073	Ddit4l	MTC_Agedup	MTC
4.42E-08	0.5390292	0.891	0.751	0.001061	Ndufc1	MTC_Agedup	MTC
3.37E-09	0.5255901	0.798	0.582	8.09E-05	Mrpl54	MTC_Agedup	MTC
6.15E-15	0.5253156	1	0.992	1.47E-10	Rpl37a	MTC_Agedup	MTC
1.12E-08	0.522598	0.58	0.335	0.000268	Stk38	MTC_Agedup	MTC
1.98E-09	0.5188149	0.95	0.859	4.74E-05	Ndufa2	MTC_Agedup	MTC
1.01E-06	0.5138262	0.529	0.32	0.024331	Psmg4	MTC_Agedup	MTC
3.36E-14	0.5126084	1	0.998	8.06E-10	Rpl41	MTC_Agedup	MTC
1.32E-06	0.5086736	0.37	0.187	0.031761	Klhdc7a	MTC_Agedup	MTC
1.62E-12	0.4945299	0.983	0.944	3.88E-08	Atp5l	MTC_Agedup	MTC

5.41E-07	0.4917423	0.235	0.079	0.012979	Fam83c	MTC_Agedup	MTC
2.63E-07	0.4911088	0.412	0.2	0.006297	Rassf3	MTC_Agedup	MTC
2.62E-09	0.490868	0.176	0.033	6.28E-05	Fam71a	MTC_Agedup	MTC
3.28E-10	0.4899654	0.849	0.609	7.86E-06	Mrpl33	MTC_Agedup	MTC
1.11E-09	0.4893409	0.983	0.946	2.65E-05	Atp5e	MTC_Agedup	MTC
1.69E-07	0.4812753	0.353	0.146	0.004059	Grk6	MTC_Agedup	MTC
4.23E-13	0.473432	1	0.996	1.01E-08	Rpl37	MTC_Agedup	MTC
1.34E-09	0.4718701	0.983	0.927	3.21E-05	Atpif1	MTC_Agedup	MTC
6.67E-07	0.4647678	0.84	0.738	0.015997	Ndufb7	MTC_Agedup	MTC
1.02E-08	0.4642811	0.294	0.1	0.000244	Rnaset2a	MTC_Agedup	MTC
4.57E-09	0.4639483	0.891	0.786	0.00011	2010107E04Rik	MTC_Agedup	MTC
4.64E-07	0.4595606	0.529	0.283	0.011127	Fut9	MTC_Agedup	MTC
2.00E-08	0.4551005	0.487	0.237	0.00048	Vkorc1	MTC_Agedup	MTC
7.27E-08	0.4532972	0.345	0.141	0.001744	Sgpl1	MTC_Agedup	MTC
3.70E-12	0.4513265	0.975	0.942	8.87E-08	Cox6b1	MTC_Agedup	MTC
1.42E-06	0.4481725	0.328	0.143	0.034153	Fam193b	MTC_Agedup	MTC
1.62E-07	0.4449495	0.361	0.156	0.003874	Acyp2	MTC_Agedup	MTC
1.28E-13	0.4435197	0.992	0.952	3.07E-09	Ndufa4	MTC_Agedup	MTC
7.04E-08	0.4397995	0.966	0.915	0.001687	Cox7a2	MTC_Agedup	MTC
2.01E-12	0.4391094	0.992	0.973	4.82E-08	Rpl36	MTC_Agedup	MTC
8.80E-09	0.4388963	0.462	0.208	0.000211	Bvht	MTC_Agedup	MTC
3.78E-07	0.438151	0.454	0.235	0.009059	Polr2l	MTC_Agedup	MTC
9.38E-07	0.4374038	0.723	0.511	0.022488	Tmem160	MTC_Agedup	MTC
4.71E-08	0.4359135	0.933	0.894	0.001129	Cox5a	MTC_Agedup	MTC
2.98E-07	0.4331652	0.891	0.85	0.007158	Ndufa13	MTC_Agedup	MTC
1.86E-08	0.4328787	0.95	0.915	0.000446	Elob	MTC_Agedup	MTC
2.25E-11	0.4313131	0.227	0.044	5.38E-07	Vegfa	MTC_Agedup	MTC
1.01E-06	0.4302599	0.958	0.902	0.024273	Sem1	MTC_Agedup	MTC
7.96E-07	0.4299706	0.815	0.63	0.0191	Cox14	MTC_Agedup	MTC
4.67E-08	0.4207419	0.546	0.295	0.001121	C77080	MTC_Agedup	MTC
4.66E-09	0.4187444	0.277	0.085	0.000112	Egln3	MTC_Agedup	MTC
9.29E-08	0.4143593	0.933	0.873	0.002229	Uqcrq	MTC_Agedup	MTC
8.98E-07	0.4140292	0.916	0.894	0.021543	Ubl5	MTC_Agedup	MTC
7.84E-07	0.4102659	0.361	0.166	0.018795	Fam20b	MTC_Agedup	MTC
5.50E-08	0.4041338	0.261	0.085	0.001319	Myo1d	MTC_Agedup	MTC
1.96E-08	0.403624	0.975	0.965	0.000469	Rps27	MTC_Agedup	MTC
5.04E-07	0.3904124	0.941	0.89	0.012083	Atp5j2	MTC_Agedup	MTC
1.45E-07	0.3903962	0.891	0.807	0.003481	Ndufa11	MTC_Agedup	MTC
2.03E-07	0.3886382	0.37	0.158	0.004866	Aldh3a2	MTC_Agedup	MTC
5.94E-07	0.3857953	0.277	0.104	0.014256	Glb1l2	MTC_Agedup	MTC
1.70E-06	0.3831571	0.319	0.141	0.040827	Chmp4c	MTC_Agedup	MTC
1.88E-06	0.3754653	0.782	0.626	0.045068	Ndufa5	MTC_Agedup	MTC
2.47E-09	0.3656209	0.235	0.058	5.92E-05	AI463170	MTC_Agedup	MTC
5.39E-07	0.3610745	0.294	0.11	0.012927	Iffo2	MTC_Agedup	MTC
5.35E-12	0.3594256	0.185	0.023	1.28E-07	Tlx2	MTC_Agedup	MTC
6.65E-07	0.3481134	0.244	0.087	0.015945	Hist2h2aa1	MTC_Agedup	MTC
1.17E-06	0.3454081	0.857	0.742	0.028031	Lamtor2	MTC_Agedup	MTC
1.97E-09	0.3409996	0.118	0.01	4.72E-05	Cdkn2a	MTC_Agedup	MTC
3.27E-08	0.3327842	0.151	0.027	0.000785	Samd9l	MTC_Agedup	MTC
2.50E-09	0.3233584	0.185	0.035	6.00E-05	Gm8797	MTC_Agedup	MTC
3.95E-07	0.319368	0.244	0.081	0.009466	Vsig10	MTC_Agedup	MTC
4.61E-08	0.3175436	0.21	0.054	0.001106	Gm10036	MTC_Agedup	MTC
1.09E-06	0.3004406	0.277	0.104	0.026039	Atg2a	MTC_Agedup	MTC
4.49E-07	0.2980208	0.193	0.054	0.010777	Ctso	MTC_Agedup	MTC
7.61E-07	0.2799124	0.21	0.062	0.018259	Iqcc	MTC_Agedup	MTC
3.34E-09	0.2738218	0.134	0.017	8.01E-05	Hist2h2ac	MTC_Agedup	MTC
1.73E-07	0.2571494	0.143	0.027	0.004141	Gsto2	MTC_Agedup	MTC
1.67E-07	0.2355272	0.143	0.027	0.004004	C920006O11Rik	MTC_Agedup	MTC
6.07E-07	0.2325237	0.143	0.029	0.014565	Ppp1r3b	MTC_Agedup	MTC

5.42E-07	0.2192946	0.151	0.033	0.01299	Gprc5c	MTC_Agedup	MTC
3.69E-08	-0.301342	0.983	0.983	0.000884	mt-Nd1	MTC_Ageddown	MTC
3.89E-07	-0.329022	0.975	0.971	0.009338	Rps10	MTC_Ageddown	MTC
4.49E-08	-0.337839	0.975	0.973	0.001077	Rps21	MTC_Ageddown	MTC
9.90E-08	-0.346542	0.983	0.975	0.002373	Rpl32	MTC_Ageddown	MTC
1.77E-06	-0.349949	0.966	0.954	0.042394	Naca	MTC_Ageddown	MTC
1.21E-06	-0.350387	0.966	0.963	0.028955	Rpl28	MTC_Ageddown	MTC
3.07E-08	-0.354159	0.983	0.981	0.000735	Rpl27a	MTC_Ageddown	MTC
2.55E-08	-0.358765	0.983	0.979	0.000611	Cox4i1	MTC_Ageddown	MTC
1.28E-06	-0.359935	0.983	0.985	0.030794	Ptma	MTC_Ageddown	MTC
1.73E-09	-0.36086	0.983	0.985	4.16E-05	Rpl23	MTC_Ageddown	MTC
4.39E-08	-0.370596	0.95	0.923	0.001054	Rpl10	MTC_Ageddown	MTC
1.22E-07	-0.38017	0.966	0.952	0.002936	Rpl14	MTC_Ageddown	MTC
1.35E-06	-0.402628	0.966	0.956	0.032314	Rps3	MTC_Ageddown	MTC
1.79E-10	-0.416647	0.983	0.977	4.29E-06	Rps3a1	MTC_Ageddown	MTC
1.88E-08	-0.42009	0.992	0.981	0.000452	Rps18	MTC_Ageddown	MTC
1.23E-08	-0.424522	0.983	0.981	0.000295	Rps16	MTC_Ageddown	MTC
3.45E-10	-0.430325	0.975	0.983	8.27E-06	Rpl15	MTC_Ageddown	MTC
7.84E-07	-0.432842	0.908	0.913	0.018804	Rps6	MTC_Ageddown	MTC
9.05E-10	-0.440674	0.958	0.956	2.17E-05	Rpl3	MTC_Ageddown	MTC
1.54E-11	-0.460574	0.992	0.96	3.69E-07	Rpl8	MTC_Ageddown	MTC
5.81E-17	-0.491659	0.992	0.99	1.39E-12	Eef1a1	MTC_Ageddown	MTC
7.83E-07	-0.495659	0.605	0.757	0.018779	Laptm4a	MTC_Ageddown	MTC
1.88E-06	-0.501555	0.529	0.728	0.045121	Sumo1	MTC_Ageddown	MTC
1.80E-06	-0.504422	0.681	0.805	0.043233	Arpc3	MTC_Ageddown	MTC
5.83E-07	-0.522296	0.151	0.395	0.013974	Tmed5	MTC_Ageddown	MTC
6.92E-08	-0.532178	0.723	0.805	0.00166	Pcbp1	MTC_Ageddown	MTC
4.50E-08	-0.533296	0.109	0.376	0.00108	Ddx3y	MTC_Ageddown	MTC
5.39E-07	-0.539771	0.756	0.859	0.012921	Eif5a	MTC_Ageddown	MTC
9.04E-13	-0.547657	0.975	0.988	2.17E-08	Rpl13	MTC_Ageddown	MTC
2.23E-07	-0.548925	0.966	0.956	0.005357	Rpl7	MTC_Ageddown	MTC
4.73E-12	-0.549149	0.958	0.983	1.13E-07	H3f3a	MTC_Ageddown	MTC
2.56E-07	-0.554163	0.798	0.863	0.006128	Eef1g	MTC_Ageddown	MTC
6.84E-10	-0.567585	0.79	0.894	1.64E-05	Sumo2	MTC_Ageddown	MTC
8.56E-07	-0.568905	0.034	0.235	0.020518	Tgfb2	MTC_Ageddown	MTC
3.34E-07	-0.584964	0.824	0.886	0.008012	Hspe1	MTC_Ageddown	MTC
3.76E-07	-0.589414	0.824	0.95	0.009018	Marcks	MTC_Ageddown	MTC
1.06E-12	-0.590945	0.966	0.981	2.55E-08	Rps4x	MTC_Ageddown	MTC
1.33E-17	-0.605516	0.975	0.96	3.20E-13	Rpl26	MTC_Ageddown	MTC
1.08E-14	-0.61544	0.975	0.933	2.59E-10	Rpl17	MTC_Ageddown	MTC
3.90E-12	-0.64046	0.815	0.884	9.34E-08	Ybx1	MTC_Ageddown	MTC
1.14E-13	-0.661079	0.975	0.979	2.74E-09	Cd9	MTC_Ageddown	MTC
3.57E-10	-0.673861	0.798	0.89	8.55E-06	Ppib	MTC_Ageddown	MTC
1.43E-27	-0.682763	0.992	0.979	3.43E-23	Tpt1	MTC_Ageddown	MTC
7.90E-17	-0.700297	0.975	0.988	1.89E-12	Rplp0	MTC_Ageddown	MTC
6.59E-20	-0.706279	1	0.975	1.58E-15	Rps14	MTC_Ageddown	MTC
2.14E-15	-0.710374	0.95	0.971	5.13E-11	Rpsa	MTC_Ageddown	MTC
3.40E-10	-0.723896	0.529	0.726	8.15E-06	Eif3e	MTC_Ageddown	MTC
6.26E-12	-0.725414	0.765	0.873	1.50E-07	Eif4a1	MTC_Ageddown	MTC
8.70E-18	-0.746091	0.983	0.985	2.09E-13	Ubb	MTC_Ageddown	MTC
3.93E-29	-0.752526	0.975	0.985	9.43E-25	mt-Nd2	MTC_Ageddown	MTC
1.30E-38	-0.76534	0.992	1	3.12E-34	mt-Cytb	MTC_Ageddown	MTC
1.77E-14	-0.782892	0.916	0.917	4.23E-10	Rps25	MTC_Ageddown	MTC
1.17E-40	-0.785111	0.992	1	2.80E-36	mt-Atp6	MTC_Ageddown	MTC
2.52E-20	-0.789522	0.924	0.931	6.04E-16	Rpl21	MTC_Ageddown	MTC
1.10E-39	-0.793227	1	1	2.65E-35	mt-Co3	MTC_Ageddown	MTC
6.95E-19	-0.801938	0.95	0.952	1.67E-14	Rplp1	MTC_Ageddown	MTC
1.19E-12	-0.805447	0.874	0.915	2.87E-08	Rps15	MTC_Ageddown	MTC
1.91E-13	-0.820462	0.714	0.875	4.58E-09	Calr	MTC_Ageddown	MTC

6.51E-11	-0.892784	0.664	0.813	1.56E-06	Manf	MTC_Ageddown	MTC
1.24E-12	-0.898727	0.84	0.896	2.97E-08	Hspa5	MTC_Ageddown	MTC
6.07E-23	-0.920449	0.983	0.969	1.45E-18	Rps8	MTC_Ageddown	MTC
5.60E-21	-0.934486	0.832	0.917	1.34E-16	Dnaja1	MTC_Ageddown	MTC
7.41E-09	-0.940477	0.378	0.682	0.000178	Krt13	MTC_Ageddown	MTC
7.17E-12	-0.950836	0.924	0.975	1.72E-07	Hspb1	MTC_Ageddown	MTC
1.94E-13	-0.973055	0.924	0.996	4.66E-09	Prdx1	MTC_Ageddown	MTC
4.16E-14	-0.985325	0.378	0.713	9.97E-10	Hsph1	MTC_Ageddown	MTC
1.21E-08	-1.015582	0.521	0.715	0.000291	Homer2	MTC_Ageddown	MTC
3.70E-07	-1.066076	0.588	0.811	0.008878	Lgals7	MTC_Ageddown	MTC
1.51E-46	-1.082211	1	0.99	3.62E-42	Hsp90ab1	MTC_Ageddown	MTC
2.03E-17	-1.125354	0.849	0.9	4.87E-13	Rpl12	MTC_Ageddown	MTC
7.94E-38	-1.130725	0.933	0.977	1.90E-33	Hspa8	MTC_Ageddown	MTC
5.83E-19	-1.13124	0.815	0.869	1.40E-14	Rpl23a	MTC_Ageddown	MTC
4.64E-07	-1.137645	0.059	0.274	0.011115	Krt14	MTC_Ageddown	MTC
1.15E-23	-1.190004	0.916	0.929	2.76E-19	Rps12	MTC_Ageddown	MTC
3.94E-28	-1.243957	0.891	0.933	9.44E-24	Hsp90aa1	MTC_Ageddown	MTC
1.26E-07	-1.350832	0.622	0.751	0.003031	Ptn	MTC_Ageddown	MTC
2.28E-14	-1.430322	0.109	0.495	5.46E-10	Hspa1a	MTC_Ageddown	MTC
6.59E-08	-1.437335	0.252	0.518	0.00158	Amy1	MTC_Ageddown	MTC
2.35E-17	-1.756204	0.529	0.832	5.64E-13	Bpifb1	MTC_Ageddown	MTC
1.16E-22	-2.04531	0.227	0.694	2.78E-18	Hspa1b	MTC_Ageddown	MTC
1.54E-41	-2.050844	0.067	0.844	3.68E-37	Tmem59	MTC_Ageddown	MTC
2.81E-25	-2.400995	0.697	0.917	6.74E-21	Sbpl	MTC_Ageddown	MTC
1.07E-27	-2.733748	0.588	0.898	2.56E-23	Lipf	MTC_Ageddown	MTC
0	2.879445	1	1	0	Gm42418	SEC_Agedup	SEC
3.18E-249	2.2336904	0.385	0.001	7.63E-245	Xist	SEC_Agedup	SEC
3.85E-278	2.0876801	0.969	0.817	9.24E-274	mt-Atp8	SEC_Agedup	SEC
5.63E-44	1.6267695	0.68	0.521	1.35E-39	Crip1	SEC_Agedup	SEC
4.85E-285	1.6134411	0.999	0.999	1.16E-280	mt-Nd4l	SEC_Agedup	SEC
7.40E-139	1.5288232	0.558	0.143	1.77E-134	Ifi27l2a	SEC_Agedup	SEC
3.02E-128	1.3969911	0.417	0.078	7.23E-124	1600014C10Rik	SEC_Agedup	SEC
2.69E-98	1.3371712	0.744	0.533	6.46E-94	Gstp1	SEC_Agedup	SEC
3.15E-126	1.3301715	0.241	0.01	7.55E-122	Gm10260	SEC_Agedup	SEC
8.99E-232	1.2729263	0.99	0.998	2.16E-227	Gm10076	SEC_Agedup	SEC
6.01E-21	1.033498	0.713	0.723	1.44E-16	Mt2	SEC_Agedup	SEC
1.12E-69	1.0226171	0.732	0.525	2.69E-65	Lars2	SEC_Agedup	SEC
4.56E-249	0.9733731	0.997	1	1.09E-244	Rps28	SEC_Agedup	SEC
9.41E-84	0.9596372	0.336	0.073	2.26E-79	Gm26870	SEC_Agedup	SEC
3.31E-13	0.8898907	0.545	0.462	7.94E-09	Igfbp2	SEC_Agedup	SEC
7.85E-12	0.8804964	0.779	0.804	1.88E-07	S100a6	SEC_Agedup	SEC
1.59E-13	0.8723641	0.747	0.936	3.82E-09	Wfdc18	SEC_Agedup	SEC
1.04E-38	0.8529989	0.573	0.349	2.49E-34	Ly6e	SEC_Agedup	SEC
6.93E-216	0.8326214	0.996	0.999	1.66E-211	Rpl38	SEC_Agedup	SEC
1.72E-60	0.8266857	0.781	0.659	4.12E-56	H2-K1	SEC_Agedup	SEC
8.11E-39	0.8132744	0.63	0.46	1.94E-34	Tppp3	SEC_Agedup	SEC
6.80E-49	0.8024021	0.55	0.309	1.63E-44	B2m	SEC_Agedup	SEC
1.05E-126	0.7928754	0.991	0.999	2.53E-122	Rpl35	SEC_Agedup	SEC
2.73E-21	0.788361	0.883	0.958	6.55E-17	Mt1	SEC_Agedup	SEC
2.60E-199	0.7790446	0.32	0.003	6.23E-195	Tff2	SEC_Agedup	SEC
9.89E-106	0.7371612	0.99	0.995	2.37E-101	mt-Nd5	SEC_Agedup	SEC
7.14E-152	0.7117338	0.999	1	1.71E-147	Rpl37a	SEC_Agedup	SEC
2.41E-50	0.7062745	0.749	0.714	5.79E-46	Snrpg	SEC_Agedup	SEC
1.11E-39	0.7049705	0.719	0.596	2.65E-35	Tmsb10	SEC_Agedup	SEC
3.21E-96	0.6999523	0.933	0.962	7.70E-92	Atp5k	SEC_Agedup	SEC
2.36E-31	0.6796788	0.436	0.247	5.66E-27	Ly6a	SEC_Agedup	SEC
5.09E-41	0.6734072	0.69	0.597	1.22E-36	Erh	SEC_Agedup	SEC
2.31E-82	0.6616525	0.928	0.983	5.53E-78	Rpl27	SEC_Agedup	SEC
2.55E-22	0.6594418	0.494	0.357	6.11E-18	Car12	SEC_Agedup	SEC

9.19E-114	0.6520703	0.959	0.992	2.20E-109	Uqcr11	SEC_Agedup	SEC
3.95E-20	0.649729	0.725	0.603	9.48E-16	Ifitm3	SEC_Agedup	SEC
1.47E-23	0.6370688	0.893	0.756	3.53E-19	Calml3	SEC_Agedup	SEC
2.66E-155	0.633446	0.999	1	6.38E-151	Rps29	SEC_Agedup	SEC
1.24E-63	0.6260587	0.864	0.912	2.98E-59	Mrpl52	SEC_Agedup	SEC
6.97E-76	0.6168358	0.924	0.986	1.67E-71	Uba52	SEC_Agedup	SEC
1.45E-93	0.602447	0.423	0.113	3.47E-89	Snhg9	SEC_Agedup	SEC
4.70E-51	0.6023602	0.822	0.766	1.13E-46	Ndufa3	SEC_Agedup	SEC
2.01E-58	0.6004923	0.836	0.854	4.82E-54	Ndufa1	SEC_Agedup	SEC
3.86E-35	0.5819164	0.57	0.39	9.25E-31	Taf1d	SEC_Agedup	SEC
2.03E-34	0.5766756	0.768	0.723	4.86E-30	Srsf5	SEC_Agedup	SEC
1.38E-12	0.5747011	0.598	0.552	3.32E-08	Hes1	SEC_Agedup	SEC
1.15E-18	0.5740963	0.591	0.488	2.75E-14	Scd2	SEC_Agedup	SEC
5.17E-19	0.5705747	0.545	0.44	1.24E-14	Dhcr24	SEC_Agedup	SEC
6.84E-142	0.5642964	0.999	1	1.64E-137	Rpl41	SEC_Agedup	SEC
3.91E-36	0.562249	0.779	0.778	9.38E-32	Bola2	SEC_Agedup	SEC
1.31E-26	0.5618491	0.732	0.715	3.14E-22	Ktn1	SEC_Agedup	SEC
6.74E-61	0.5611098	0.936	0.971	1.62E-56	Pabpc1	SEC_Agedup	SEC
5.21E-30	0.5579453	0.684	0.59	1.25E-25	Pet100	SEC_Agedup	SEC
3.54E-95	0.5483802	0.996	1	8.48E-91	Rpl37	SEC_Agedup	SEC
9.56E-106	0.5478636	0.99	1	2.29E-101	Rpl36	SEC_Agedup	SEC
9.00E-20	0.534151	0.982	0.995	2.16E-15	Dbi	SEC_Agedup	SEC
4.89E-14	0.5278553	0.794	0.831	1.17E-09	H2-D1	SEC_Agedup	SEC
6.96E-14	0.5123465	0.665	0.591	1.67E-09	4631405K08Rik	SEC_Agedup	SEC
2.56E-32	0.5118328	0.7	0.599	6.14E-28	Lsm7	SEC_Agedup	SEC
3.73E-35	0.5091603	0.472	0.268	8.94E-31	Flna	SEC_Agedup	SEC
6.89E-26	0.5068326	0.588	0.431	1.65E-21	Sod1	SEC_Agedup	SEC
1.35E-35	0.4959579	0.86	0.928	3.23E-31	Usmg5	SEC_Agedup	SEC
8.04E-13	0.4905781	0.673	0.694	1.93E-08	Prom2	SEC_Agedup	SEC
1.52E-30	0.4898977	0.699	0.597	3.64E-26	Ewsr1	SEC_Agedup	SEC
7.67E-25	0.4886375	0.596	0.457	1.84E-20	Gm47283	SEC_Agedup	SEC
2.05E-21	0.4865295	0.715	0.682	4.93E-17	Rsrp1	SEC_Agedup	SEC
1.21E-37	0.4850549	0.849	0.858	2.91E-33	Romo1	SEC_Agedup	SEC
2.77E-25	0.4820417	0.63	0.511	6.65E-21	Larp1	SEC_Agedup	SEC
3.85E-72	0.4798117	0.206	0.025	9.23E-68	Muc5b	SEC_Agedup	SEC
6.65E-08	0.4780973	0.178	0.11	0.001594	Tnfaip2	SEC_Agedup	SEC
2.05E-31	0.47616	0.531	0.342	4.93E-27	Cluh	SEC_Agedup	SEC
3.71E-08	0.4693489	0.93	0.904	0.00089	Krt4	SEC_Agedup	SEC
2.46E-30	0.4640874	0.477	0.289	5.90E-26	Snhg20	SEC_Agedup	SEC
8.48E-13	0.4621576	0.529	0.435	2.03E-08	Ece1	SEC_Agedup	SEC
7.00E-14	0.453931	0.652	0.601	1.68E-09	Notch1	SEC_Agedup	SEC
1.28E-22	0.4515227	0.858	0.911	3.07E-18	Ncl	SEC_Agedup	SEC
2.88E-17	0.4491993	0.713	0.688	6.90E-13	Hnrnpl	SEC_Agedup	SEC
2.23E-22	0.4470015	0.956	0.989	5.35E-18	Ndufa4	SEC_Agedup	SEC
1.59E-23	0.4459168	0.728	0.68	3.81E-19	Ddb1	SEC_Agedup	SEC
1.97E-48	0.4432108	0.984	1	4.72E-44	Rps27	SEC_Agedup	SEC
4.56E-17	0.4399292	0.823	0.881	1.09E-12	Ptms	SEC_Agedup	SEC
7.75E-18	0.4377533	0.69	0.636	1.86E-13	Nop53	SEC_Agedup	SEC
8.83E-11	0.4363598	0.724	0.758	2.12E-06	Cdh1	SEC_Agedup	SEC
1.12E-12	0.4358141	0.749	0.766	2.69E-08	Lad1	SEC_Agedup	SEC
1.44E-37	0.4345921	0.871	0.964	3.44E-33	Tomm7	SEC_Agedup	SEC
7.92E-08	0.432434	0.573	0.527	0.001899	Barx2	SEC_Agedup	SEC
7.44E-21	0.4289828	0.873	0.926	1.78E-16	Cstb	SEC_Agedup	SEC
6.55E-12	0.4249353	0.427	0.333	1.57E-07	Clec2d	SEC_Agedup	SEC
6.34E-14	0.4180982	0.636	0.573	1.52E-09	Ptgfrn	SEC_Agedup	SEC
1.06E-09	0.4133333	0.428	0.346	2.55E-05	Sdc3	SEC_Agedup	SEC
4.35E-18	0.4120738	0.642	0.554	1.04E-13	Rcc2	SEC_Agedup	SEC
3.06E-16	0.405295	0.75	0.778	7.34E-12	Eif4g1	SEC_Agedup	SEC
2.00E-26	0.4020336	0.458	0.28	4.80E-22	Vars	SEC_Agedup	SEC

4.75E-16	0.3989593	0.624	0.545	1.14E-11	Ccnl2	SEC_Agedup	SEC
3.16E-62	0.3971793	0.994	1	7.59E-58	Rps19	SEC_Agedup	SEC
1.48E-13	0.3948119	0.482	0.382	3.54E-09	Tmem259	SEC_Agedup	SEC
8.78E-13	0.3939653	0.569	0.481	2.11E-08	C77080	SEC_Agedup	SEC
1.93E-19	0.3905024	0.784	0.811	4.63E-15	Iqgap1	SEC_Agedup	SEC
5.95E-17	0.389145	0.787	0.855	1.43E-12	Son	SEC_Agedup	SEC
1.94E-10	0.3888085	0.684	0.672	4.65E-06	Eif4b	SEC_Agedup	SEC
1.00E-12	0.3877219	0.627	0.563	2.40E-08	Prrc2b	SEC_Agedup	SEC
7.42E-15	0.3872851	0.569	0.494	1.78E-10	Eif2s3x	SEC_Agedup	SEC
1.27E-14	0.3852826	0.7	0.659	3.05E-10	Eif3b	SEC_Agedup	SEC
1.23E-12	0.3837811	0.535	0.448	2.96E-08	Hr	SEC_Agedup	SEC
1.52E-25	0.382945	0.406	0.237	3.65E-21	Rpl27-ps3	SEC_Agedup	SEC
4.27E-17	0.3815628	0.512	0.38	1.02E-12	Cavin1	SEC_Agedup	SEC
3.81E-34	0.378163	0.943	0.988	9.13E-30	Uqcrq	SEC_Agedup	SEC
1.01E-19	0.3774279	0.921	0.977	2.41E-15	Lmna	SEC_Agedup	SEC
2.79E-20	0.3768015	0.864	0.925	6.69E-16	Spint2	SEC_Agedup	SEC
4.85E-25	0.3717118	0.876	0.958	1.16E-20	2010107E04Rik	SEC_Agedup	SEC
8.00E-16	0.3714865	0.849	0.952	1.92E-11	Sdc1	SEC_Agedup	SEC
9.25E-14	0.3706013	0.658	0.627	2.22E-09	1810022K09Rik	SEC_Agedup	SEC
5.56E-19	0.3684453	0.526	0.382	1.33E-14	Mybbp1a	SEC_Agedup	SEC
4.44E-24	0.3613414	0.399	0.224	1.06E-19	9-Sep	SEC_Agedup	SEC
3.32E-07	0.3584251	0.807	0.9	0.007968	Atp1a1	SEC_Agedup	SEC
1.89E-10	0.3561156	0.504	0.427	4.53E-06	Plxna1	SEC_Agedup	SEC
2.41E-10	0.3527256	0.506	0.444	5.77E-06	AC149090.1	SEC_Agedup	SEC
3.12E-13	0.3525212	0.719	0.728	7.47E-09	Cs	SEC_Agedup	SEC
4.32E-17	0.3522772	0.776	0.826	1.04E-12	Ndufa5	SEC_Agedup	SEC
6.69E-11	0.3505786	0.541	0.447	1.60E-06	Gm26917	SEC_Agedup	SEC
2.71E-20	0.3490314	0.885	0.961	6.50E-16	Rpl36al	SEC_Agedup	SEC
2.24E-09	0.3488511	0.706	0.681	5.38E-05	Hspa9	SEC_Agedup	SEC
1.12E-08	0.348534	0.596	0.547	0.000268	Hnrnpdl	SEC_Agedup	SEC
6.88E-13	0.3474543	0.539	0.455	1.65E-08	Coro1c	SEC_Agedup	SEC
1.67E-07	0.3471878	0.541	0.496	0.004007	Diaph1	SEC_Agedup	SEC
3.51E-11	0.3443159	0.415	0.295	8.41E-07	Ccnd1	SEC_Agedup	SEC
6.62E-20	0.343198	0.867	0.958	1.59E-15	Atp5g3	SEC_Agedup	SEC
4.53E-09	0.342088	0.539	0.492	0.000109	Dhcr7	SEC_Agedup	SEC
9.37E-12	0.3409232	0.985	0.999	2.25E-07	Actb	SEC_Agedup	SEC
5.35E-38	0.340052	0.965	0.998	1.28E-33	Cox6c	SEC_Agedup	SEC
4.99E-16	0.3380597	0.804	0.874	1.20E-11	Ost4	SEC_Agedup	SEC
1.90E-11	0.3344949	0.385	0.271	4.56E-07	Itgb4	SEC_Agedup	SEC
1.37E-10	0.3332596	0.493	0.414	3.29E-06	Tnpo2	SEC_Agedup	SEC
1.20E-10	0.3303437	0.626	0.575	2.88E-06	Aco2	SEC_Agedup	SEC
1.21E-07	0.3300969	0.728	0.757	0.002892	Ddx3x	SEC_Agedup	SEC
7.18E-14	0.3291565	0.405	0.283	1.72E-09	Slc39a1	SEC_Agedup	SEC
7.50E-10	0.3267395	0.788	0.869	1.80E-05	Psap	SEC_Agedup	SEC
1.13E-12	0.3244002	0.374	0.259	2.70E-08	Slc35a4	SEC_Agedup	SEC
3.21E-31	0.3241374	0.991	1	7.70E-27	Rps20	SEC_Agedup	SEC
6.38E-10	0.3228146	0.257	0.161	1.53E-05	Slc16a11	SEC_Agedup	SEC
3.85E-14	0.3226155	0.798	0.882	9.23E-10	Ndufc2	SEC_Agedup	SEC
9.96E-13	0.3224812	0.323	0.213	2.39E-08	Tomm6	SEC_Agedup	SEC
7.08E-09	0.3214048	0.469	0.391	0.00017	Plxnb1	SEC_Agedup	SEC
1.11E-09	0.3203283	0.604	0.549	2.66E-05	Mink1	SEC_Agedup	SEC
6.06E-10	0.3201282	0.291	0.196	1.45E-05	Cald1	SEC_Agedup	SEC
6.00E-12	0.3195401	0.484	0.383	1.44E-07	Fat2	SEC_Agedup	SEC
4.85E-12	0.3153889	0.842	0.937	1.16E-07	Snrpe	SEC_Agedup	SEC
1.75E-08	0.3151512	0.367	0.29	0.00042	Phyhip	SEC_Agedup	SEC
7.16E-10	0.3137877	0.586	0.521	1.72E-05	Hk1	SEC_Agedup	SEC
4.78E-17	0.3131364	0.38	0.24	1.15E-12	Dvl1	SEC_Agedup	SEC
5.67E-08	0.312797	0.611	0.592	0.001359	Wdr1	SEC_Agedup	SEC
8.88E-10	0.3127802	0.595	0.544	2.13E-05	Prrc2a	SEC_Agedup	SEC

4.54E-09	0.3106179	0.81	0.885	0.000109	Ndufb2	SEC_Agedup	SEC
1.97E-09	0.3102843	0.661	0.67	4.72E-05	Got2	SEC_Agedup	SEC
3.04E-10	0.3100618	0.471	0.384	7.29E-06	Bag6	SEC_Agedup	SEC
3.00E-43	0.3100394	0.994	0.999	7.19E-39	Rpl18a	SEC_Agedup	SEC
1.20E-08	0.3090077	0.485	0.414	0.000287	Stat3	SEC_Agedup	SEC
1.83E-09	0.3084042	0.658	0.65	4.38E-05	Trmt112	SEC_Agedup	SEC
3.64E-29	0.3079742	0.969	0.996	8.72E-25	Cox6b1	SEC_Agedup	SEC
1.78E-08	0.3063142	0.693	0.703	0.000427	Tcf25	SEC_Agedup	SEC
2.99E-12	0.3047163	0.453	0.343	7.17E-08	Polr2l	SEC_Agedup	SEC
9.00E-13	0.3040972	0.447	0.335	2.16E-08	Atxn2l	SEC_Agedup	SEC
6.70E-18	0.3037087	0.836	0.914	1.61E-13	Ndufa2	SEC_Agedup	SEC
1.07E-09	0.3015052	0.329	0.232	2.56E-05	Ptprs	SEC_Agedup	SEC
3.62E-08	0.3003417	0.621	0.613	0.000869	Gas6	SEC_Agedup	SEC
8.24E-08	0.2992251	0.699	0.696	0.001975	Hnrnph1	SEC_Agedup	SEC
3.92E-07	0.2987658	0.594	0.592	0.009402	Sap18	SEC_Agedup	SEC
1.11E-14	0.2984621	0.232	0.125	2.67E-10	Gdpd2	SEC_Agedup	SEC
1.39E-33	0.2970083	0.289	0.113	3.33E-29	Gm10036	SEC_Agedup	SEC
5.29E-08	0.2945497	0.835	0.942	0.001269	Ptprf	SEC_Agedup	SEC
1.58E-07	0.2930395	0.782	0.825	0.003789	Ucp2	SEC_Agedup	SEC
4.50E-30	0.2924048	0.958	0.996	1.08E-25	Cox7c	SEC_Agedup	SEC
1.22E-08	0.2921955	0.506	0.447	0.000293	Setd5	SEC_Agedup	SEC
6.82E-09	0.2920963	0.37	0.272	0.000164	Slc6a6	SEC_Agedup	SEC
1.67E-17	0.2916284	0.89	0.963	4.01E-13	Cox6a1	SEC_Agedup	SEC
1.76E-07	0.2912986	0.589	0.565	0.004233	Srebf2	SEC_Agedup	SEC
7.23E-13	0.2908596	0.379	0.267	1.73E-08	Susd6	SEC_Agedup	SEC
5.08E-15	0.2904504	0.923	0.978	1.22E-10	Sec61g	SEC_Agedup	SEC
1.87E-11	0.290257	0.792	0.84	4.49E-07	Ndufv3	SEC_Agedup	SEC
5.38E-07	0.2888417	0.738	0.768	0.012893	Snrnp70	SEC_Agedup	SEC
1.24E-13	0.2882719	0.379	0.265	2.97E-09	Grcc10	SEC_Agedup	SEC
3.84E-20	0.2881753	0.914	0.982	9.21E-16	Uqcr10	SEC_Agedup	SEC
1.20E-09	0.2868621	0.404	0.309	2.87E-05	Atp6v0a1	SEC_Agedup	SEC
3.85E-21	0.2866792	0.963	0.998	9.24E-17	Rpl36a	SEC_Agedup	SEC
1.50E-10	0.2857753	0.534	0.452	3.60E-06	Hypk	SEC_Agedup	SEC
8.77E-09	0.2846166	0.632	0.591	0.00021	Gtf2i	SEC_Agedup	SEC
3.65E-07	0.2845099	0.507	0.45	0.008764	Clk1	SEC_Agedup	SEC
8.63E-15	0.2832648	0.865	0.946	2.07E-10	Minos1	SEC_Agedup	SEC
3.96E-18	0.2828899	0.959	0.992	9.49E-14	Pkp1	SEC_Agedup	SEC
1.09E-06	0.2814117	0.115	0.204	0.026236	Slurp1	SEC_Agedup	SEC
5.55E-12	0.2794416	0.386	0.275	1.33E-07	MuCn1l1	SEC_Agedup	SEC
5.48E-16	0.279117	0.327	0.2	1.31E-11	Zfp692	SEC_Agedup	SEC
1.28E-06	0.2774226	0.424	0.355	0.030749	Ctdsp2	SEC_Agedup	SEC
4.74E-30	0.2755705	0.225	0.079	1.14E-25	Akr1b8	SEC_Agedup	SEC
3.76E-07	0.2755004	0.621	0.604	0.009007	Plbd1	SEC_Agedup	SEC
6.96E-10	0.2750253	0.262	0.409	1.67E-05	Fetub	SEC_Agedup	SEC
3.79E-11	0.274808	0.45	0.346	9.09E-07	Prpf8	SEC_Agedup	SEC
2.01E-08	0.2747758	0.389	0.304	0.000482	Plcb3	SEC_Agedup	SEC
3.16E-08	0.2746309	0.386	0.296	0.000759	Mapkapk2	SEC_Agedup	SEC
2.02E-06	0.2718197	0.563	0.523	0.048325	Eif4ebp2	SEC_Agedup	SEC
4.02E-09	0.2716165	0.069	0.164	9.64E-05	2300002M23Rik	SEC_Agedup	SEC
8.35E-09	0.2714599	0.496	0.425	0.0002	Ddx21	SEC_Agedup	SEC
3.62E-22	0.2707728	0.994	0.999	8.69E-18	Rps2	SEC_Agedup	SEC
3.55E-08	0.2694514	0.428	0.352	0.000851	Pfkl	SEC_Agedup	SEC
1.40E-09	0.2685668	0.896	0.963	3.36E-05	Pkm	SEC_Agedup	SEC
9.79E-07	0.2674805	0.64	0.649	0.023478	Dpm3	SEC_Agedup	SEC
3.00E-11	0.2673185	0.327	0.224	7.20E-07	Zfc3h1	SEC_Agedup	SEC
1.27E-08	0.2671996	0.424	0.334	0.000306	Gpt	SEC_Agedup	SEC
3.94E-07	0.2668206	0.427	0.356	0.009452	Scap	SEC_Agedup	SEC
3.29E-08	0.2667023	0.871	0.927	0.00079	Prelid1	SEC_Agedup	SEC
7.06E-07	0.2663591	0.659	0.663	0.016936	Tardbp	SEC_Agedup	SEC

3.73E-09	0.2659969	0.355	0.261	8.94E-05	Tollip	SEC_Agedup	SEC
1.89E-30	0.2648355	0.993	1	4.54E-26	Rpl30	SEC_Agedup	SEC
1.62E-08	0.2637847	0.379	0.293	0.000389	Rab15	SEC_Agedup	SEC
7.46E-39	0.2635349	0.171	0.037	1.79E-34	Samd9l	SEC_Agedup	SEC
4.30E-08	0.2626057	0.414	0.338	0.001032	Kars	SEC_Agedup	SEC
1.01E-08	0.2618773	0.439	0.363	0.000243	Dag1	SEC_Agedup	SEC
1.04E-09	0.2617991	0.841	0.888	2.48E-05	Arpp19	SEC_Agedup	SEC
3.49E-10	0.259886	0.332	0.238	8.37E-06	Scaf1	SEC_Agedup	SEC
2.46E-08	0.2597399	0.713	0.755	0.00059	Naa38	SEC_Agedup	SEC
1.51E-10	0.2595055	0.308	0.207	3.62E-06	Furin	SEC_Agedup	SEC
5.11E-08	0.2591861	0.557	0.526	0.001226	Dnajc19	SEC_Agedup	SEC
6.30E-08	0.2591024	0.434	0.362	0.00151	Bcl9l	SEC_Agedup	SEC
8.44E-13	0.2588861	0.396	0.279	2.02E-08	Atad3a	SEC_Agedup	SEC
2.20E-19	0.2586826	0.288	0.151	5.28E-15	Trpm4	SEC_Agedup	SEC
3.39E-16	0.2582397	0.329	0.195	8.14E-12	Ptk7	SEC_Agedup	SEC
5.76E-11	0.2573364	0.437	0.331	1.38E-06	Pum3	SEC_Agedup	SEC
1.07E-06	0.2560625	0.354	0.284	0.025714	Akt2	SEC_Agedup	SEC
3.25E-08	0.2559953	0.501	0.427	0.000779	Xrn2	SEC_Agedup	SEC
9.03E-07	0.2542477	0.629	0.622	0.021661	Chd4	SEC_Agedup	SEC
1.40E-14	0.2528925	0.139	0.055	3.36E-10	Pappa	SEC_Agedup	SEC
1.84E-09	0.2525734	0.83	0.883	4.40E-05	Ndufb7	SEC_Agedup	SEC
8.07E-10	0.2523472	0.336	0.24	1.94E-05	Rgl2	SEC_Agedup	SEC
5.47E-08	0.2522492	0.747	0.76	0.001313	Nedd4	SEC_Agedup	SEC
6.84E-23	0.2487558	0.942	0.995	1.64E-18	Atp5l	SEC_Agedup	SEC
7.70E-07	0.2485954	0.667	0.658	0.018455	Hdlbp	SEC_Agedup	SEC
4.56E-07	0.2482395	0.62	0.597	0.010943	Srsf1	SEC_Agedup	SEC
5.43E-07	0.2466454	0.841	0.863	0.013014	Ftl1	SEC_Agedup	SEC
3.20E-07	0.2448712	0.402	0.328	0.007674	Lrp5	SEC_Agedup	SEC
9.87E-15	0.2445512	0.924	0.988	2.37E-10	Elob	SEC_Agedup	SEC
8.07E-07	0.2444179	0.544	0.515	0.019364	Gm11808	SEC_Agedup	SEC
3.07E-09	0.2429801	0.383	0.289	7.37E-05	Acaca	SEC_Agedup	SEC
2.39E-13	0.2405234	0.253	0.145	5.74E-09	Slc6a9	SEC_Agedup	SEC
1.86E-08	0.2401137	0.368	0.279	0.000447	Usp22	SEC_Agedup	SEC
3.74E-08	0.2397535	0.345	0.263	0.000898	Ltb4r1	SEC_Agedup	SEC
1.05E-07	0.23876	0.459	0.379	0.002516	Akt1	SEC_Agedup	SEC
9.37E-08	0.2380289	0.455	0.378	0.002246	Ndufs1	SEC_Agedup	SEC
1.21E-06	0.236974	0.373	0.306	0.029062	Dlst	SEC_Agedup	SEC
1.63E-23	0.235819	0.988	1	3.90E-19	Rpl11	SEC_Agedup	SEC
1.02E-10	0.2347952	0.345	0.243	2.44E-06	Cdcp1	SEC_Agedup	SEC
1.99E-08	0.2346134	0.32	0.23	0.000478	Acvr1b	SEC_Agedup	SEC
2.41E-08	0.2323465	0.259	0.179	0.000578	Arih2	SEC_Agedup	SEC
3.44E-08	0.231973	0.332	0.245	0.000826	Arhgef19	SEC_Agedup	SEC
1.72E-06	0.2317757	0.42	0.344	0.041197	Raf1	SEC_Agedup	SEC
4.06E-08	0.2309542	0.335	0.248	0.000974	Hs6st1	SEC_Agedup	SEC
4.16E-07	0.2304302	0.278	0.203	0.009972	Ppp6r1	SEC_Agedup	SEC
9.07E-07	0.2296967	0.762	0.768	0.021752	Nfib	SEC_Agedup	SEC
2.48E-07	0.2286124	0.301	0.22	0.005946	Anpep	SEC_Agedup	SEC
1.16E-16	0.2280871	0.943	0.997	2.79E-12	Cox5b	SEC_Agedup	SEC
3.82E-11	0.2280747	0.228	0.134	9.16E-07	Apobec1	SEC_Agedup	SEC
2.69E-13	0.2275328	0.314	0.196	6.44E-09	Acsl3	SEC_Agedup	SEC
3.98E-19	0.2271911	0.192	0.081	9.55E-15	Clec16a	SEC_Agedup	SEC
1.86E-07	0.2269245	0.406	0.321	0.004466	Atp2b4	SEC_Agedup	SEC
4.37E-10	0.2268767	0.38	0.279	1.05E-05	Strn4	SEC_Agedup	SEC
1.30E-15	0.2267554	0.215	0.107	3.12E-11	Als2cl	SEC_Agedup	SEC
2.17E-08	0.2257018	0.36	0.273	0.00052	Kmt2d	SEC_Agedup	SEC
1.43E-06	0.2240689	0.313	0.236	0.034338	Ass1	SEC_Agedup	SEC
4.61E-15	0.2240138	0.205	0.1	1.10E-10	Zfp46	SEC_Agedup	SEC
2.45E-07	0.2229304	0.463	0.382	0.005868	Ddx54	SEC_Agedup	SEC
1.52E-22	0.2225844	0.997	1	3.65E-18	Rps26	SEC_Agedup	SEC

1.36E-06	0.2216708	0.292	0.216	0.032688	Lrpprc	SEC_Agedup	SEC
8.41E-08	0.2210751	0.33	0.241	0.002016	Cdca7	SEC_Agedup	SEC
3.08E-17	0.2207691	0.93	0.996	7.39E-13	Atp5g2	SEC_Agedup	SEC
6.25E-19	0.22043	0.178	0.071	1.50E-14	Numbl	SEC_Agedup	SEC
5.29E-10	0.2202843	0.924	0.996	1.27E-05	Atp5e	SEC_Agedup	SEC
1.75E-11	0.2202537	0.246	0.148	4.20E-07	Rhbdf2	SEC_Agedup	SEC
4.52E-07	0.2202106	0.281	0.207	0.010848	Cc2d1b	SEC_Agedup	SEC
9.62E-20	0.2201623	0.259	0.125	2.31E-15	Lamb2	SEC_Agedup	SEC
6.05E-07	0.2192967	0.382	0.309	0.014503	Prkcd	SEC_Agedup	SEC
1.47E-11	0.2189825	0.354	0.239	3.52E-07	Clk4	SEC_Agedup	SEC
5.10E-08	0.2184872	0.82	0.929	0.001222	Ndufa11	SEC_Agedup	SEC
2.55E-07	0.218325	0.291	0.211	0.006107	Opa1	SEC_Agedup	SEC
1.93E-11	0.2182532	0.307	0.205	4.62E-07	Lonp1	SEC_Agedup	SEC
4.68E-07	0.2182181	0.32	0.244	0.011231	Dolpp1	SEC_Agedup	SEC
1.19E-06	0.2166053	0.363	0.291	0.028537	Snrnp200	SEC_Agedup	SEC
1.79E-11	0.2157336	0.379	0.268	4.29E-07	Ddx42	SEC_Agedup	SEC
8.84E-12	0.2157207	0.265	0.159	2.12E-07	Zfp219	SEC_Agedup	SEC
1.20E-07	0.2146319	0.386	0.291	0.002878	Myof	SEC_Agedup	SEC
9.35E-12	0.2138635	0.94	0.994	2.24E-07	Eef2	SEC_Agedup	SEC
1.77E-08	0.2137518	0.335	0.245	0.000424	Ehmt2	SEC_Agedup	SEC
3.69E-07	0.2134848	0.38	0.302	0.008857	Txlna	SEC_Agedup	SEC
3.96E-16	0.2128272	0.262	0.136	9.49E-12	Slc1a5	SEC_Agedup	SEC
1.25E-08	0.2118385	0.376	0.275	0.0003	Ltbp4	SEC_Agedup	SEC
6.92E-08	0.2117857	0.409	0.318	0.00166	Shisa5	SEC_Agedup	SEC
5.49E-08	0.2110087	0.333	0.246	0.001316	Asph	SEC_Agedup	SEC
4.18E-09	0.2048001	0.348	0.251	0.0001	Copg1	SEC_Agedup	SEC
2.36E-10	0.2047063	0.194	0.109	5.65E-06	Gstp2	SEC_Agedup	SEC
1.15E-11	0.2044989	0.183	0.096	2.76E-07	Moxd1	SEC_Agedup	SEC
6.10E-10	0.2042281	0.205	0.121	1.46E-05	Il6st	SEC_Agedup	SEC
1.94E-10	0.2040595	0.275	0.179	4.65E-06	Ints1	SEC_Agedup	SEC
1.43E-06	0.2030863	0.827	0.925	0.034226	Nop10	SEC_Agedup	SEC
1.99E-17	0.2029579	0.996	1	4.77E-13	Rpl39	SEC_Agedup	SEC
2.05E-08	0.2024394	0.314	0.226	0.000493	Map2k7	SEC_Agedup	SEC
1.08E-08	0.2003543	0.251	0.168	0.00026	Aagab	SEC_Agedup	SEC
1.04E-16	-0.200205	0.488	0.682	2.49E-12	Magoh	SEC_Ageddown	SEC
1.15E-17	-0.200764	0.262	0.453	2.75E-13	Rsl24d1	SEC_Ageddown	SEC
1.99E-17	-0.200988	0.558	0.756	4.78E-13	Psma6	SEC_Ageddown	SEC
1.49E-11	-0.20136	0.808	0.956	3.57E-07	Nfe2l2	SEC_Ageddown	SEC
1.28E-16	-0.201381	0.7	0.891	3.06E-12	Aes	SEC_Ageddown	SEC
3.96E-18	-0.201725	0.31	0.51	9.49E-14	Tef	SEC_Ageddown	SEC
6.60E-15	-0.201813	0.294	0.472	1.58E-10	Rassf9	SEC_Ageddown	SEC
4.92E-17	-0.2027	0.265	0.447	1.18E-12	Prelid3b	SEC_Ageddown	SEC
4.98E-21	-0.202709	0.156	0.357	1.19E-16	Eif2s3y	SEC_Ageddown	SEC
1.58E-18	-0.202854	0.754	0.919	3.79E-14	Ptges3	SEC_Ageddown	SEC
4.36E-19	-0.202999	0.317	0.514	1.05E-14	Cebpzos	SEC_Ageddown	SEC
3.03E-14	-0.203299	0.43	0.595	7.27E-10	Strn3	SEC_Ageddown	SEC
4.39E-20	-0.203453	0.174	0.361	1.05E-15	Lipm	SEC_Ageddown	SEC
2.99E-16	-0.203746	0.569	0.743	7.17E-12	Rexo2	SEC_Ageddown	SEC
4.59E-18	-0.204118	0.145	0.32	1.10E-13	Dbp	SEC_Ageddown	SEC
1.12E-18	-0.204221	0.26	0.453	2.67E-14	Med19	SEC_Ageddown	SEC
1.19E-16	-0.204609	0.346	0.546	2.85E-12	Gjb2	SEC_Ageddown	SEC
8.27E-20	-0.204704	0.569	0.77	1.98E-15	Ube2n	SEC_Ageddown	SEC
1.67E-18	-0.206451	0.456	0.675	4.00E-14	Ammecr1	SEC_Ageddown	SEC
2.45E-22	-0.206468	0.311	0.534	5.89E-18	Dnajb11	SEC_Ageddown	SEC
2.41E-16	-0.206542	0.447	0.637	5.78E-12	Pin1	SEC_Ageddown	SEC
4.94E-21	-0.206969	0.349	0.557	1.18E-16	Triap1	SEC_Ageddown	SEC
2.71E-12	-0.207647	0.316	0.473	6.49E-08	Cldn10	SEC_Ageddown	SEC
7.74E-16	-0.207693	0.569	0.772	1.86E-11	Bax	SEC_Ageddown	SEC
4.28E-15	-0.20777	0.088	0.221	1.03E-10	Cmtm8	SEC_Ageddown	SEC

2.76E-19	-0.208828	0.257	0.453	6.63E-15	Tfg	SEC_Ageddown	SEC
4.73E-17	-0.208997	0.414	0.609	1.13E-12	Commd1	SEC_Ageddown	SEC
8.49E-16	-0.209764	0.515	0.695	2.04E-11	MuCat	SEC_Ageddown	SEC
3.26E-21	-0.209842	0.496	0.712	7.83E-17	Zcrb1	SEC_Ageddown	SEC
1.01E-19	-0.210245	0.173	0.356	2.43E-15	Lsm1	SEC_Ageddown	SEC
3.44E-15	-0.210284	0.314	0.475	8.26E-11	Polr3g	SEC_Ageddown	SEC
3.74E-20	-0.210509	0.993	1	8.97E-16	Rps9	SEC_Ageddown	SEC
2.31E-17	-0.210512	0.205	0.398	5.55E-13	Alox12b	SEC_Ageddown	SEC
8.91E-19	-0.210597	0.977	1	2.14E-14	Ppia	SEC_Ageddown	SEC
2.66E-22	-0.21084	0.414	0.646	6.39E-18	Mrpl18	SEC_Ageddown	SEC
1.43E-19	-0.211185	0.256	0.458	3.43E-15	Derl2	SEC_Ageddown	SEC
4.80E-12	-0.211516	0.82	0.944	1.15E-07	Hmgn1	SEC_Ageddown	SEC
1.67E-15	-0.212363	0.727	0.894	4.02E-11	Mrpl20	SEC_Ageddown	SEC
5.34E-14	-0.212611	0.604	0.774	1.28E-09	0610012G03Rik	SEC_Ageddown	SEC
1.92E-20	-0.212884	0.26	0.462	4.60E-16	Bub3	SEC_Ageddown	SEC
3.17E-20	-0.212941	0.61	0.798	7.60E-16	Sypl	SEC_Ageddown	SEC
2.94E-21	-0.213227	0.276	0.488	7.05E-17	Ccdc59	SEC_Ageddown	SEC
1.15E-17	-0.213552	0.393	0.597	2.76E-13	Dnajc3	SEC_Ageddown	SEC
6.39E-20	-0.214076	0.367	0.568	1.53E-15	Sar1b	SEC_Ageddown	SEC
7.16E-17	-0.214544	0.443	0.642	1.72E-12	Rab21	SEC_Ageddown	SEC
1.22E-17	-0.214547	0.187	0.356	2.93E-13	Creld2	SEC_Ageddown	SEC
7.15E-15	-0.214881	0.614	0.791	1.71E-10	Slc38a2	SEC_Ageddown	SEC
1.69E-13	-0.214996	0.849	0.964	4.05E-09	Ralbp1	SEC_Ageddown	SEC
5.88E-19	-0.215225	0.5	0.711	1.41E-14	Mpzl2	SEC_Ageddown	SEC
2.94E-20	-0.216401	0.949	0.996	7.05E-16	Eif5a	SEC_Ageddown	SEC
3.21E-16	-0.216874	0.789	0.955	7.69E-12	Ssr4	SEC_Ageddown	SEC
7.52E-14	-0.216966	0.681	0.86	1.80E-09	Rabac1	SEC_Ageddown	SEC
3.01E-18	-0.217101	0.354	0.562	7.21E-14	Vamp3	SEC_Ageddown	SEC
1.74E-16	-0.217379	0.288	0.469	4.18E-12	Mcrip1	SEC_Ageddown	SEC
1.50E-15	-0.217698	0.509	0.701	3.61E-11	Mmp2	SEC_Ageddown	SEC
2.72E-16	-0.217929	0.402	0.591	6.52E-12	Mtpn	SEC_Ageddown	SEC
4.52E-26	-0.218177	0.288	0.525	1.08E-21	Acaa1a	SEC_Ageddown	SEC
1.05E-20	-0.219286	0.607	0.798	2.53E-16	Ostf1	SEC_Ageddown	SEC
1.36E-18	-0.219358	0.516	0.718	3.26E-14	Psma1	SEC_Ageddown	SEC
1.20E-20	-0.219992	0.281	0.493	2.87E-16	Ppp6c	SEC_Ageddown	SEC
1.26E-16	-0.220154	0.211	0.381	3.03E-12	Sox21	SEC_Ageddown	SEC
6.61E-18	-0.220533	0.44	0.642	1.59E-13	Vdac3	SEC_Ageddown	SEC
9.80E-15	-0.220601	0.803	0.959	2.35E-10	Hnrnpf	SEC_Ageddown	SEC
1.14E-10	-0.220607	0.74	0.9	2.73E-06	Hspd1	SEC_Ageddown	SEC
1.73E-23	-0.220614	0.408	0.636	4.15E-19	Acadl	SEC_Ageddown	SEC
1.48E-27	-0.220636	0.975	1	3.54E-23	Gsto1	SEC_Ageddown	SEC
1.33E-22	-0.220917	0.301	0.527	3.18E-18	Glod4	SEC_Ageddown	SEC
3.49E-18	-0.2211	0.974	0.999	8.37E-14	Rpl3	SEC_Ageddown	SEC
1.75E-15	-0.221299	0.77	0.891	4.20E-11	Gsn	SEC_Ageddown	SEC
4.82E-20	-0.221425	0.26	0.471	1.16E-15	Utp3	SEC_Ageddown	SEC
3.39E-14	-0.221461	0.51	0.696	8.12E-10	Rdx	SEC_Ageddown	SEC
5.82E-23	-0.221792	0.985	0.999	1.40E-18	Rpl7	SEC_Ageddown	SEC
5.44E-19	-0.22186	0.263	0.461	1.30E-14	Zcchc17	SEC_Ageddown	SEC
1.34E-24	-0.221934	0.215	0.43	3.21E-20	Snrnp25	SEC_Ageddown	SEC
7.21E-15	-0.221997	0.406	0.564	1.73E-10	Efna3	SEC_Ageddown	SEC
4.34E-18	-0.222774	0.279	0.472	1.04E-13	Lamtor3	SEC_Ageddown	SEC
1.38E-23	-0.222785	0.047	0.21	3.32E-19	Ang	SEC_Ageddown	SEC
1.79E-26	-0.223196	0.993	1	4.29E-22	Eef1a1	SEC_Ageddown	SEC
1.11E-17	-0.223407	0.37	0.57	2.65E-13	Tuba1a	SEC_Ageddown	SEC
1.50E-19	-0.223489	0.551	0.761	3.59E-15	Pfdn1	SEC_Ageddown	SEC
3.45E-18	-0.223571	0.423	0.615	8.26E-14	Elovl7	SEC_Ageddown	SEC
8.40E-20	-0.223997	0.485	0.712	2.01E-15	Etfa	SEC_Ageddown	SEC
1.89E-22	-0.224177	0.754	0.925	4.53E-18	Cnbp	SEC_Ageddown	SEC
5.91E-17	-0.22428	0.382	0.561	1.42E-12	Creg1	SEC_Ageddown	SEC

5.18E-22	-0.224814	0.215	0.422	1.24E-17	Pycr2	SEC_Ageddown	SEC
3.29E-20	-0.225031	0.427	0.647	7.89E-16	Paqr5	SEC_Ageddown	SEC
5.25E-19	-0.225575	0.446	0.655	1.26E-14	Fam96b	SEC_Ageddown	SEC
2.94E-23	-0.226096	0.575	0.784	7.04E-19	Ppp2ca	SEC_Ageddown	SEC
3.70E-18	-0.226366	0.425	0.627	8.88E-14	Arap2	SEC_Ageddown	SEC
3.03E-22	-0.226662	0.3	0.519	7.28E-18	Spg21	SEC_Ageddown	SEC
5.05E-14	-0.226775	0.415	0.576	1.21E-09	Me1	SEC_Ageddown	SEC
3.97E-20	-0.227117	0.025	0.16	9.52E-16	Cldn17	SEC_Ageddown	SEC
1.04E-23	-0.227574	0.408	0.64	2.49E-19	Eif4e2	SEC_Ageddown	SEC
8.00E-15	-0.228435	0.137	0.279	1.92E-10	Cpn1	SEC_Ageddown	SEC
1.09E-14	-0.228532	0.709	0.882	2.61E-10	Srsf2	SEC_Ageddown	SEC
2.38E-15	-0.228681	0.759	0.915	5.71E-11	Pgam1	SEC_Ageddown	SEC
4.48E-19	-0.228933	0.567	0.782	1.07E-14	Atp6v1d	SEC_Ageddown	SEC
8.35E-22	-0.228965	0.991	0.999	2.00E-17	Rps18	SEC_Ageddown	SEC
4.71E-23	-0.229584	0.136	0.335	1.13E-18	Banp	SEC_Ageddown	SEC
1.68E-18	-0.229682	0.345	0.541	4.03E-14	Hagh	SEC_Ageddown	SEC
2.39E-22	-0.229834	0.028	0.172	5.72E-18	Mndal	SEC_Ageddown	SEC
8.64E-18	-0.230772	0.436	0.642	2.07E-13	Echs1	SEC_Ageddown	SEC
2.17E-22	-0.231334	0.43	0.66	5.19E-18	Psmd13	SEC_Ageddown	SEC
1.92E-15	-0.232472	0.779	0.914	4.60E-11	Pcbp2	SEC_Ageddown	SEC
2.06E-18	-0.233084	0.496	0.715	4.95E-14	Hist1h2bc	SEC_Ageddown	SEC
3.64E-15	-0.233807	0.832	0.941	8.74E-11	Pitx1	SEC_Ageddown	SEC
1.62E-18	-0.233958	0.126	0.291	3.89E-14	UMuCg	SEC_Ageddown	SEC
1.53E-22	-0.234463	0.199	0.399	3.67E-18	Pnp	SEC_Ageddown	SEC
4.67E-23	-0.234759	0.62	0.818	1.12E-18	Psmd8	SEC_Ageddown	SEC
1.17E-24	-0.235382	0.231	0.454	2.81E-20	3830406C13Rik	SEC_Ageddown	SEC
7.71E-24	-0.235432	0.832	0.959	1.85E-19	Gabarap	SEC_Ageddown	SEC
3.40E-18	-0.236788	0.314	0.51	8.15E-14	Ethe1	SEC_Ageddown	SEC
7.00E-22	-0.237687	0.352	0.578	1.68E-17	Mpp7	SEC_Ageddown	SEC
4.78E-18	-0.23769	0.68	0.867	1.15E-13	Tst	SEC_Ageddown	SEC
5.28E-20	-0.238478	0.203	0.398	1.27E-15	Tubb2a	SEC_Ageddown	SEC
4.42E-20	-0.238752	0.991	1	1.06E-15	Rps15a	SEC_Ageddown	SEC
2.76E-21	-0.240203	0.408	0.64	6.62E-17	Rheb	SEC_Ageddown	SEC
7.06E-19	-0.241835	0.566	0.761	1.69E-14	Cct4	SEC_Ageddown	SEC
2.84E-20	-0.242085	0.673	0.851	6.80E-16	Cct5	SEC_Ageddown	SEC
1.31E-17	-0.24241	0.648	0.826	3.15E-13	Vdac2	SEC_Ageddown	SEC
3.18E-19	-0.242494	0.437	0.649	7.63E-15	Pura	SEC_Ageddown	SEC
1.21E-10	-0.243487	0.507	0.647	2.90E-06	Lmo4	SEC_Ageddown	SEC
1.99E-23	-0.244457	0.361	0.584	4.77E-19	Actr10	SEC_Ageddown	SEC
2.76E-13	-0.244755	0.563	0.698	6.61E-09	Degs1	SEC_Ageddown	SEC
1.26E-21	-0.245499	0.924	0.997	3.02E-17	Atp5d	SEC_Ageddown	SEC
1.38E-17	-0.246062	0.813	0.951	3.32E-13	Cyb5a	SEC_Ageddown	SEC
7.38E-23	-0.24668	0.427	0.632	1.77E-18	Ormdl2	SEC_Ageddown	SEC
7.25E-20	-0.246786	0.436	0.646	1.74E-15	Ufc1	SEC_Ageddown	SEC
4.98E-21	-0.247178	0.561	0.76	1.20E-16	Cd47	SEC_Ageddown	SEC
8.19E-07	-0.248258	0.054	0.121	0.019647	Krt76	SEC_Ageddown	SEC
1.88E-29	-0.249329	0.028	0.215	4.50E-25	Prb1	SEC_Ageddown	SEC
4.67E-22	-0.249787	0.146	0.332	1.12E-17	Pawr	SEC_Ageddown	SEC
1.30E-22	-0.250922	0.768	0.933	3.11E-18	Lamp2	SEC_Ageddown	SEC
1.06E-19	-0.251014	0.738	0.927	2.55E-15	Psma3	SEC_Ageddown	SEC
1.05E-22	-0.25112	0.45	0.668	2.53E-18	Eif2s1	SEC_Ageddown	SEC
3.65E-22	-0.251354	0.636	0.87	8.74E-18	Mrps24	SEC_Ageddown	SEC
2.86E-27	-0.251728	0.905	0.992	6.86E-23	Arf5	SEC_Ageddown	SEC
4.13E-19	-0.252375	0.494	0.708	9.91E-15	Mrpl21	SEC_Ageddown	SEC
1.53E-27	-0.253387	0.975	1	3.68E-23	Naca	SEC_Ageddown	SEC
4.35E-21	-0.253666	0.808	0.966	1.04E-16	Eif3k	SEC_Ageddown	SEC
3.75E-23	-0.253794	0.789	0.952	8.99E-19	Eif3i	SEC_Ageddown	SEC
1.39E-20	-0.254502	0.243	0.442	3.34E-16	Nfkbia	SEC_Ageddown	SEC
4.34E-21	-0.255569	0.757	0.945	1.04E-16	Psmb3	SEC_Ageddown	SEC

3.38E-26	-0.255897	0.117	0.319	8.10E-22	Gm9493	SEC_Ageddown	SEC
7.32E-24	-0.25688	0.314	0.528	1.75E-19	Rraga	SEC_Ageddown	SEC
3.71E-24	-0.258282	0.513	0.73	8.89E-20	Arl6ip1	SEC_Ageddown	SEC
8.37E-25	-0.258373	0.034	0.196	2.01E-20	Rnase1	SEC_Ageddown	SEC
1.19E-35	-0.258981	0.962	0.999	2.84E-31	S100a11	SEC_Ageddown	SEC
3.50E-17	-0.259007	0.596	0.799	8.39E-13	Hnrnpc	SEC_Ageddown	SEC
3.10E-29	-0.260174	0.156	0.397	7.44E-25	Ddx3y	SEC_Ageddown	SEC
5.85E-19	-0.262033	0.205	0.386	1.40E-14	Acer1	SEC_Ageddown	SEC
9.44E-18	-0.263072	0.757	0.91	2.26E-13	Gpx1	SEC_Ageddown	SEC
2.16E-24	-0.263551	0.69	0.91	5.19E-20	Psma2	SEC_Ageddown	SEC
7.18E-23	-0.26401	0.497	0.723	1.72E-18	Pop5	SEC_Ageddown	SEC
9.30E-12	-0.264063	0.675	0.84	2.23E-07	Ces1d	SEC_Ageddown	SEC
3.19E-20	-0.264215	0.234	0.425	7.64E-16	Pgap1	SEC_Ageddown	SEC
4.00E-22	-0.264251	0.83	0.969	9.58E-18	Psma7	SEC_Ageddown	SEC
7.10E-20	-0.264275	0.494	0.693	1.70E-15	Ubxn4	SEC_Ageddown	SEC
4.07E-16	-0.264337	0.942	0.996	9.75E-12	Dsp	SEC_Ageddown	SEC
7.69E-22	-0.265168	0.336	0.544	1.84E-17	Atp6ap2	SEC_Ageddown	SEC
2.63E-21	-0.265457	0.539	0.773	6.31E-17	Pdia6	SEC_Ageddown	SEC
1.90E-23	-0.266695	0.503	0.726	4.56E-19	Kctd1	SEC_Ageddown	SEC
3.31E-22	-0.267056	0.319	0.545	7.95E-18	Dsg1b	SEC_Ageddown	SEC
2.22E-26	-0.267607	0.194	0.421	5.31E-22	2510002D24Rik	SEC_Ageddown	SEC
8.53E-17	-0.267867	0.642	0.845	2.05E-12	Phlda3	SEC_Ageddown	SEC
9.96E-21	-0.268101	0.478	0.695	2.39E-16	Sec11a	SEC_Ageddown	SEC
1.65E-25	-0.268576	0.108	0.307	3.95E-21	Gsdmc	SEC_Ageddown	SEC
2.52E-22	-0.268593	0.569	0.782	6.05E-18	Ube2b	SEC_Ageddown	SEC
3.99E-22	-0.268861	0.661	0.874	9.57E-18	Rtraf	SEC_Ageddown	SEC
1.49E-23	-0.269762	0.598	0.812	3.58E-19	Ube2i	SEC_Ageddown	SEC
2.99E-24	-0.26977	0.225	0.442	7.18E-20	Eid1	SEC_Ageddown	SEC
1.09E-31	-0.26984	0.882	0.99	2.62E-27	Fth1	SEC_Ageddown	SEC
1.31E-16	-0.270278	0.465	0.665	3.15E-12	Foxq1	SEC_Ageddown	SEC
5.01E-22	-0.271232	0.526	0.714	1.20E-17	Gng12	SEC_Ageddown	SEC
1.04E-17	-0.271254	0.488	0.683	2.50E-13	Sgk1	SEC_Ageddown	SEC
5.15E-19	-0.273443	0.696	0.891	1.23E-14	Eif5	SEC_Ageddown	SEC
9.99E-24	-0.273667	0.615	0.81	2.40E-19	Elovl1	SEC_Ageddown	SEC
2.80E-22	-0.274354	0.654	0.83	6.72E-18	Actr3	SEC_Ageddown	SEC
7.36E-20	-0.274495	0.722	0.905	1.76E-15	Anp32b	SEC_Ageddown	SEC
1.01E-14	-0.275629	0.759	0.873	2.43E-10	Klf4	SEC_Ageddown	SEC
2.38E-25	-0.275873	0.325	0.56	5.71E-21	Gdpd1	SEC_Ageddown	SEC
1.39E-18	-0.277214	0.792	0.935	3.34E-14	Mdh1	SEC_Ageddown	SEC
9.78E-24	-0.278485	0.355	0.582	2.35E-19	Snrnp48	SEC_Ageddown	SEC
3.03E-21	-0.278781	0.743	0.929	7.26E-17	Rtn4	SEC_Ageddown	SEC
3.08E-26	-0.279183	0.425	0.671	7.39E-22	Rp9	SEC_Ageddown	SEC
1.44E-24	-0.279425	0.482	0.697	3.46E-20	Smap1	SEC_Ageddown	SEC
5.47E-25	-0.280526	0.579	0.802	1.31E-20	Tbcb	SEC_Ageddown	SEC
1.38E-41	-0.281073	0.996	1	3.30E-37	Rplp1	SEC_Ageddown	SEC
1.05E-23	-0.282054	0.348	0.562	2.52E-19	Tnfaip8	SEC_Ageddown	SEC
2.67E-21	-0.282698	0.751	0.905	6.39E-17	Ubc	SEC_Ageddown	SEC
1.15E-25	-0.283255	0.817	0.96	2.75E-21	Taldo1	SEC_Ageddown	SEC
6.39E-17	-0.283976	0.769	0.934	1.53E-12	Ybx1	SEC_Ageddown	SEC
2.94E-29	-0.284276	0.405	0.661	7.04E-25	Pon2	SEC_Ageddown	SEC
3.96E-25	-0.284426	0.306	0.534	9.51E-21	Rab27b	SEC_Ageddown	SEC
2.83E-26	-0.2849	0.545	0.777	6.79E-22	Vapa	SEC_Ageddown	SEC
7.09E-30	-0.285239	0.121	0.347	1.70E-25	Ddit3	SEC_Ageddown	SEC
1.53E-27	-0.287102	0.652	0.875	3.66E-23	Dctn3	SEC_Ageddown	SEC
1.07E-29	-0.287431	0.455	0.699	2.57E-25	Cldnd1	SEC_Ageddown	SEC
1.48E-18	-0.28808	0.731	0.901	3.55E-14	2010111I01Rik	SEC_Ageddown	SEC
6.39E-29	-0.288142	0.412	0.682	1.53E-24	Gsta2	SEC_Ageddown	SEC
1.27E-22	-0.288156	0.458	0.67	3.04E-18	Selenos	SEC_Ageddown	SEC
1.35E-08	-0.2888	0.393	0.508	0.000324	Ctsl	SEC_Ageddown	SEC

4.30E-23	-0.290389	0.686	0.898	1.03E-18	Psmb2	SEC_Ageddown	SEC
2.94E-20	-0.290792	0.785	0.939	7.05E-16	Dsc2	SEC_Ageddown	SEC
3.46E-23	-0.292427	0.469	0.695	8.30E-19	Rdh12	SEC_Ageddown	SEC
3.19E-14	-0.293015	0.614	0.738	7.65E-10	Jpt1	SEC_Ageddown	SEC
6.31E-26	-0.295529	0.798	0.954	1.51E-21	Rab10	SEC_Ageddown	SEC
9.01E-27	-0.295978	0.82	0.968	2.16E-22	Rnh1	SEC_Ageddown	SEC
4.73E-26	-0.296094	0.861	0.962	1.13E-21	Gltp	SEC_Ageddown	SEC
3.61E-22	-0.296752	0.711	0.901	8.66E-18	Top1	SEC_Ageddown	SEC
2.51E-24	-0.297193	0.449	0.68	6.01E-20	Car13	SEC_Ageddown	SEC
1.19E-25	-0.298607	0.333	0.568	2.85E-21	Dnajc21	SEC_Ageddown	SEC
7.22E-22	-0.300693	0.567	0.759	1.73E-17	Atp11b	SEC_Ageddown	SEC
9.70E-27	-0.301315	0.501	0.758	2.33E-22	Imp3	SEC_Ageddown	SEC
7.62E-26	-0.302232	0.564	0.787	1.83E-21	Ywhaq	SEC_Ageddown	SEC
2.11E-27	-0.303974	0.614	0.849	5.05E-23	Srp14	SEC_Ageddown	SEC
9.57E-27	-0.304209	0.557	0.788	2.29E-22	Aldh3b2	SEC_Ageddown	SEC
7.93E-45	-0.304465	0.999	1	1.90E-40	Rplp0	SEC_Ageddown	SEC
1.96E-28	-0.305067	0.827	0.985	4.71E-24	Pfdn5	SEC_Ageddown	SEC
9.21E-31	-0.305637	0.114	0.34	2.21E-26	Gt(ROSA)26Sor	SEC_Ageddown	SEC
1.11E-23	-0.3059	0.855	0.983	2.66E-19	Polr1d	SEC_Ageddown	SEC
8.01E-24	-0.306243	0.772	0.901	1.92E-19	Higd1a	SEC_Ageddown	SEC
1.33E-26	-0.306389	0.151	0.368	3.19E-22	Hspa2	SEC_Ageddown	SEC
5.61E-19	-0.306505	0.857	0.974	1.35E-14	Hspe1	SEC_Ageddown	SEC
3.99E-32	-0.307165	0.107	0.334	9.58E-28	Ahsa2	SEC_Ageddown	SEC
1.57E-32	-0.307327	0.795	0.944	3.77E-28	Cebpb	SEC_Ageddown	SEC
3.36E-30	-0.308935	0.222	0.466	8.07E-26	Mrps6	SEC_Ageddown	SEC
2.24E-27	-0.309053	0.37	0.615	5.36E-23	Tmem165	SEC_Ageddown	SEC
5.64E-28	-0.309404	0.402	0.656	1.35E-23	Bad	SEC_Ageddown	SEC
5.96E-35	-0.309638	0.216	0.482	1.43E-30	Mpst	SEC_Ageddown	SEC
5.24E-33	-0.310032	0.222	0.481	1.26E-28	Cited2	SEC_Ageddown	SEC
1.69E-35	-0.31006	0.982	0.999	4.04E-31	Rpl17	SEC_Ageddown	SEC
3.40E-38	-0.310743	0.958	0.999	8.15E-34	Rpl10	SEC_Ageddown	SEC
3.49E-26	-0.311413	0.858	0.986	8.37E-22	Eef1d	SEC_Ageddown	SEC
4.10E-28	-0.311791	0.753	0.942	9.84E-24	Clta	SEC_Ageddown	SEC
2.34E-32	-0.31305	0.927	0.994	5.62E-28	Calm1	SEC_Ageddown	SEC
5.87E-33	-0.314419	0.104	0.34	1.41E-28	Dnajb9	SEC_Ageddown	SEC
6.56E-24	-0.315195	0.971	0.998	1.57E-19	Rps15	SEC_Ageddown	SEC
4.83E-24	-0.318377	0.456	0.71	1.16E-19	Foxe1	SEC_Ageddown	SEC
5.82E-22	-0.318538	0.687	0.889	1.40E-17	Cox7a2l	SEC_Ageddown	SEC
3.96E-24	-0.318793	0.778	0.954	9.49E-20	Atp5c1	SEC_Ageddown	SEC
1.84E-28	-0.319613	0.899	0.994	4.40E-24	Gapdh	SEC_Ageddown	SEC
5.93E-21	-0.320246	0.816	0.97	1.42E-16	Esd	SEC_Ageddown	SEC
4.46E-53	-0.321236	0.99	0.999	1.07E-48	Rpl8	SEC_Ageddown	SEC
2.07E-28	-0.321404	0.538	0.778	4.96E-24	Rer1	SEC_Ageddown	SEC
5.50E-27	-0.321711	0.553	0.782	1.32E-22	Sri	SEC_Ageddown	SEC
2.47E-32	-0.321743	0.458	0.718	5.93E-28	Trappc4	SEC_Ageddown	SEC
1.93E-30	-0.324056	0.459	0.676	4.63E-26	Dusp22	SEC_Ageddown	SEC
5.28E-17	-0.32459	0.332	0.537	1.27E-12	Elovl4	SEC_Ageddown	SEC
1.28E-24	-0.325375	0.7	0.856	3.08E-20	Rab11a	SEC_Ageddown	SEC
2.71E-28	-0.325829	0.949	0.998	6.49E-24	Tmsb4x	SEC_Ageddown	SEC
6.82E-28	-0.32693	0.251	0.497	1.64E-23	Tcim	SEC_Ageddown	SEC
1.18E-21	-0.327511	0.462	0.665	2.84E-17	Chl1	SEC_Ageddown	SEC
6.12E-32	-0.328059	0.472	0.736	1.47E-27	Mrpl42	SEC_Ageddown	SEC
1.35E-32	-0.328503	0.31	0.557	3.24E-28	Yipf4	SEC_Ageddown	SEC
2.19E-29	-0.329027	0.162	0.391	5.25E-25	Tsc22d3	SEC_Ageddown	SEC
1.16E-22	-0.32904	0.376	0.575	2.78E-18	Hspa4l	SEC_Ageddown	SEC
8.23E-31	-0.329078	0.351	0.614	1.97E-26	Ankrd12	SEC_Ageddown	SEC
6.43E-14	-0.329146	0.057	0.168	1.54E-09	Socs3	SEC_Ageddown	SEC
1.79E-36	-0.329727	0.961	0.999	4.29E-32	Rpl29	SEC_Ageddown	SEC
7.03E-40	-0.330284	0.142	0.422	1.69E-35	2310033P09Rik	SEC_Ageddown	SEC

3.37E-33	-0.33034	0.518	0.761	8.08E-29	Bcas2	SEC_Ageddown	SEC
1.72E-31	-0.332345	0.677	0.881	4.13E-27	Paip2	SEC_Ageddown	SEC
1.95E-27	-0.33236	0.92	0.996	4.68E-23	Fxyd3	SEC_Ageddown	SEC
6.56E-10	-0.333979	0.975	0.99	1.57E-05	Krt13	SEC_Ageddown	SEC
2.04E-28	-0.334505	0.58	0.784	4.90E-24	Tuba4a	SEC_Ageddown	SEC
4.26E-31	-0.335774	0.763	0.95	1.02E-26	Psmb1	SEC_Ageddown	SEC
6.48E-38	-0.3358	0.99	0.999	1.55E-33	Rps3a1	SEC_Ageddown	SEC
8.42E-32	-0.337212	0.611	0.856	2.02E-27	Glrx3	SEC_Ageddown	SEC
3.66E-34	-0.337941	0.472	0.744	8.78E-30	Gm16136	SEC_Ageddown	SEC
5.36E-21	-0.339156	0.544	0.748	1.29E-16	Sox2	SEC_Ageddown	SEC
3.97E-31	-0.339806	0.415	0.66	9.53E-27	Ech1	SEC_Ageddown	SEC
7.43E-23	-0.340177	0.301	0.506	1.78E-18	RMuCc	SEC_Ageddown	SEC
5.35E-31	-0.34051	0.308	0.577	1.28E-26	Pim3	SEC_Ageddown	SEC
1.64E-30	-0.345708	0.576	0.801	3.92E-26	Cnih4	SEC_Ageddown	SEC
3.79E-29	-0.346942	0.143	0.382	9.08E-25	Ier3	SEC_Ageddown	SEC
7.38E-16	-0.348188	0.443	0.604	1.77E-11	Krt32	SEC_Ageddown	SEC
1.93E-27	-0.348755	0.442	0.686	4.62E-23	Cd81	SEC_Ageddown	SEC
4.52E-27	-0.353033	0.731	0.915	1.08E-22	Tacstd2	SEC_Ageddown	SEC
3.84E-35	-0.353423	0.287	0.564	9.20E-31	Smagp	SEC_Ageddown	SEC
2.38E-21	-0.353729	0.934	0.961	5.70E-17	Calm4	SEC_Ageddown	SEC
8.09E-33	-0.357999	0.481	0.736	1.94E-28	Stip1	SEC_Ageddown	SEC
5.72E-41	-0.358799	0.627	0.873	1.37E-36	Scp2	SEC_Ageddown	SEC
2.64E-21	-0.359903	0.645	0.822	6.32E-17	Pdzk1ip1	SEC_Ageddown	SEC
2.48E-46	-0.362018	0.985	0.999	5.94E-42	Rpl15	SEC_Ageddown	SEC
8.00E-46	-0.364256	0.965	0.999	1.92E-41	Ptma	SEC_Ageddown	SEC
7.97E-52	-0.364907	0.981	0.999	1.91E-47	Rps25	SEC_Ageddown	SEC
6.88E-39	-0.365102	0.323	0.613	1.65E-34	Acp1	SEC_Ageddown	SEC
5.69E-38	-0.366418	0.507	0.775	1.36E-33	BC031181	SEC_Ageddown	SEC
1.41E-17	-0.36726	0.213	0.388	3.39E-13	Il1r2	SEC_Ageddown	SEC
2.33E-31	-0.371511	0.588	0.784	5.60E-27	Cd24a	SEC_Ageddown	SEC
1.02E-45	-0.371885	0.789	0.958	2.45E-41	Arpc3	SEC_Ageddown	SEC
6.50E-74	-0.37316	0.993	0.999	1.56E-69	Rps14	SEC_Ageddown	SEC
1.13E-37	-0.374704	0.797	0.96	2.71E-33	Eif3h	SEC_Ageddown	SEC
3.65E-42	-0.374909	0.756	0.934	8.76E-38	Rhoa	SEC_Ageddown	SEC
1.24E-35	-0.376268	0.402	0.674	2.99E-31	Nudc	SEC_Ageddown	SEC
7.00E-37	-0.379194	0.509	0.77	1.68E-32	Rbm8a	SEC_Ageddown	SEC
3.21E-44	-0.380665	0.481	0.772	7.69E-40	Chmp5	SEC_Ageddown	SEC
3.16E-40	-0.381685	0.344	0.647	7.58E-36	Zkscan3	SEC_Ageddown	SEC
1.42E-45	-0.387173	0.433	0.74	3.40E-41	Tprgl	SEC_Ageddown	SEC
1.13E-59	-0.388048	0.99	1	2.71E-55	Rps4x	SEC_Ageddown	SEC
5.53E-39	-0.389599	0.811	0.976	1.33E-34	Ppib	SEC_Ageddown	SEC
3.76E-37	-0.391518	0.276	0.546	9.01E-33	Mettl23	SEC_Ageddown	SEC
2.65E-39	-0.391947	0.694	0.893	6.35E-35	Dynlt3	SEC_Ageddown	SEC
7.02E-23	-0.392172	0.439	0.645	1.68E-18	Lap3	SEC_Ageddown	SEC
6.94E-44	-0.393665	0.741	0.938	1.66E-39	Pcbp1	SEC_Ageddown	SEC
3.51E-41	-0.394591	0.537	0.814	8.42E-37	Eif3m	SEC_Ageddown	SEC
5.54E-34	-0.395494	0.481	0.724	1.33E-29	Pdlim2	SEC_Ageddown	SEC
1.78E-41	-0.395665	0.001	0.224	4.28E-37	Gm8882	SEC_Ageddown	SEC
2.71E-25	-0.396912	0.506	0.721	6.49E-21	Ier2	SEC_Ageddown	SEC
2.15E-63	-0.397689	0.996	1	5.15E-59	Rpl13	SEC_Ageddown	SEC
1.02E-39	-0.397826	0.13	0.398	2.44E-35	Sdf2l1	SEC_Ageddown	SEC
2.08E-29	-0.398091	0.308	0.555	4.98E-25	Arl4a	SEC_Ageddown	SEC
8.42E-28	-0.398114	0.545	0.736	2.02E-23	Blmh	SEC_Ageddown	SEC
6.68E-40	-0.398191	0.87	0.977	1.60E-35	Eif6	SEC_Ageddown	SEC
9.86E-23	-0.398845	0.054	0.218	2.37E-18	Fosb	SEC_Ageddown	SEC
1.51E-32	-0.399077	0.67	0.866	3.62E-28	Wnt4	SEC_Ageddown	SEC
1.67E-27	-0.402198	0.851	0.962	4.00E-23	Them5	SEC_Ageddown	SEC
3.70E-20	-0.402576	0.494	0.686	8.87E-16	Klf6	SEC_Ageddown	SEC
1.29E-34	-0.40479	0.181	0.454	3.10E-30	Clu	SEC_Ageddown	SEC

3.78E-27	-0.412311	0.749	0.926	9.06E-23	Neat1	SEC_Ageddown	SEC
2.29E-40	-0.412792	0.633	0.843	5.48E-36	Ift20	SEC_Ageddown	SEC
1.05E-16	-0.413237	0.868	0.978	2.51E-12	Anxa1	SEC_Ageddown	SEC
7.39E-43	-0.419859	0.509	0.781	1.77E-38	Bcap31	SEC_Ageddown	SEC
3.32E-56	-0.422295	0.928	1	7.95E-52	Cox4i1	SEC_Ageddown	SEC
2.62E-27	-0.425411	0.82	0.936	6.29E-23	Lypd3	SEC_Ageddown	SEC
1.02E-45	-0.426563	0.294	0.6	2.44E-41	Tmem33	SEC_Ageddown	SEC
1.78E-42	-0.427448	0.848	0.987	4.27E-38	Clic1	SEC_Ageddown	SEC
4.97E-69	-0.428357	0.944	0.998	1.19E-64	Btf3	SEC_Ageddown	SEC
2.83E-39	-0.428477	0.553	0.794	6.78E-35	U2af1	SEC_Ageddown	SEC
3.29E-45	-0.428733	0.658	0.909	7.90E-41	Erp29	SEC_Ageddown	SEC
5.14E-47	-0.429402	0.576	0.846	1.23E-42	Spcs2	SEC_Ageddown	SEC
3.71E-25	-0.436518	0.826	0.922	8.90E-21	Tgm3	SEC_Ageddown	SEC
6.93E-49	-0.438252	0.406	0.705	1.66E-44	Med21	SEC_Ageddown	SEC
2.01E-39	-0.442811	0.776	0.948	4.83E-35	Calr	SEC_Ageddown	SEC
6.75E-55	-0.443242	0.317	0.659	1.62E-50	Hspb8	SEC_Ageddown	SEC
4.67E-38	-0.44388	0.314	0.59	1.12E-33	Sdr16c5	SEC_Ageddown	SEC
2.87E-44	-0.444146	0.629	0.859	6.88E-40	Atp6v1a	SEC_Ageddown	SEC
4.50E-26	-0.444869	0.449	0.672	1.08E-21	Serpinb1a	SEC_Ageddown	SEC
1.97E-50	-0.445265	0.583	0.862	4.72E-46	Brk1	SEC_Ageddown	SEC
1.70E-60	-0.445796	0.966	0.996	4.09E-56	S100a14	SEC_Ageddown	SEC
1.43E-53	-0.446841	0.801	0.965	3.44E-49	Arpc2	SEC_Ageddown	SEC
3.03E-61	-0.449637	0.956	0.999	7.26E-57	Prdx1	SEC_Ageddown	SEC
3.99E-38	-0.451733	0.728	0.902	9.57E-34	Anxa5	SEC_Ageddown	SEC
1.07E-52	-0.454474	0.845	0.992	2.56E-48	Slc25a5	SEC_Ageddown	SEC
4.25E-24	-0.455874	0.368	0.61	1.02E-19	Tmprss11d	SEC_Ageddown	SEC
7.48E-57	-0.456215	0.844	0.98	1.79E-52	Hras	SEC_Ageddown	SEC
1.48E-41	-0.457735	0.681	0.911	3.55E-37	Atp6v1e1	SEC_Ageddown	SEC
6.60E-35	-0.466694	0.675	0.87	1.58E-30	Pir	SEC_Ageddown	SEC
1.36E-48	-0.47231	0.558	0.829	3.25E-44	Psma4	SEC_Ageddown	SEC
6.44E-35	-0.474174	0.291	0.567	1.54E-30	2310057J18Rik	SEC_Ageddown	SEC
1.45E-52	-0.47467	0.481	0.779	3.47E-48	Snx3	SEC_Ageddown	SEC
4.66E-55	-0.474852	0.782	0.976	1.12E-50	Atp6v0e	SEC_Ageddown	SEC
1.96E-107	-0.476206	0.993	1	4.71E-103	Rpl26	SEC_Ageddown	SEC
1.37E-71	-0.477002	0.911	0.994	3.29E-67	Eif4a1	SEC_Ageddown	SEC
2.00E-10	-0.480842	0.42	0.545	4.80E-06	Btg2	SEC_Ageddown	SEC
1.61E-48	-0.481204	0.556	0.837	3.87E-44	Sumo1	SEC_Ageddown	SEC
4.00E-59	-0.481278	0.925	0.999	9.60E-55	H3f3b	SEC_Ageddown	SEC
9.86E-50	-0.485992	0.303	0.612	2.36E-45	Chordc1	SEC_Ageddown	SEC
5.45E-51	-0.489279	0.69	0.905	1.31E-46	Eif3e	SEC_Ageddown	SEC
7.23E-12	-0.490484	0.072	0.179	1.73E-07	Krt24	SEC_Ageddown	SEC
3.86E-50	-0.491834	0.246	0.558	9.25E-46	Gadd45b	SEC_Ageddown	SEC
3.77E-33	-0.492255	0.823	0.942	9.03E-29	Prdx5	SEC_Ageddown	SEC
8.30E-46	-0.500696	0.374	0.666	1.99E-41	Herpud1	SEC_Ageddown	SEC
4.95E-69	-0.501033	0.988	0.999	1.19E-64	Lgals7	SEC_Ageddown	SEC
1.53E-45	-0.508368	0.604	0.863	3.66E-41	Mafb	SEC_Ageddown	SEC
1.70E-17	-0.51092	0.991	0.999	4.07E-13	Fabp5	SEC_Ageddown	SEC
2.66E-70	-0.512468	0.985	0.999	6.39E-66	Ly6d	SEC_Ageddown	SEC
3.18E-91	-0.512735	0.999	1	7.63E-87	mt-Nd2	SEC_Ageddown	SEC
9.16E-130	-0.51671	0.999	1	2.20E-125	mt-Cytb	SEC_Ageddown	SEC
3.23E-44	-0.520656	0.77	0.955	7.74E-40	Aqp3	SEC_Ageddown	SEC
3.54E-07	-0.522442	0.262	0.353	0.008485	Egr1	SEC_Ageddown	SEC
3.54E-72	-0.525821	0.892	0.994	8.49E-68	Prdx2	SEC_Ageddown	SEC
1.21E-80	-0.534604	0.966	0.998	2.90E-76	Rpl23a	SEC_Ageddown	SEC
5.06E-83	-0.536089	0.968	0.998	1.21E-78	Perp	SEC_Ageddown	SEC
1.69E-73	-0.536437	0.925	0.994	4.06E-69	Pycard	SEC_Ageddown	SEC
9.13E-68	-0.536824	0.618	0.882	2.19E-63	Psmd7	SEC_Ageddown	SEC
2.01E-63	-0.538338	0.499	0.829	4.82E-59	Fkbp4	SEC_Ageddown	SEC
1.55E-153	-0.542166	0.996	1	3.72E-149	Tpt1	SEC_Ageddown	SEC

7.44E-65	-0.547936	0.696	0.93	1.78E-60	Laptm4a	SEC_Ageddown	SEC
4.59E-53	-0.551298	0.978	0.999	1.10E-48	Gsta4	SEC_Ageddown	SEC
2.03E-23	-0.552766	0.424	0.635	4.86E-19	Id3	SEC_Ageddown	SEC
6.96E-37	-0.554166	0.906	0.993	1.67E-32	Krt6a	SEC_Ageddown	SEC
1.03E-87	-0.556006	0.987	0.999	2.47E-83	Dynll1	SEC_Ageddown	SEC
4.90E-28	-0.568947	0.787	0.922	1.18E-23	Sbsn	SEC_Ageddown	SEC
2.60E-50	-0.57252	0.316	0.645	6.23E-46	Amy1	SEC_Ageddown	SEC
4.90E-27	-0.575315	0.58	0.773	1.17E-22	Selenbp1	SEC_Ageddown	SEC
7.06E-44	-0.578475	0.76	0.911	1.69E-39	Tubb4b	SEC_Ageddown	SEC
3.14E-27	-0.580108	0.206	0.422	7.54E-23	Zfp36	SEC_Ageddown	SEC
2.18E-57	-0.58033	0.757	0.958	5.24E-53	Mgst3	SEC_Ageddown	SEC
2.69E-46	-0.58525	0.295	0.612	6.44E-42	Endou	SEC_Ageddown	SEC
7.38E-62	-0.589115	0.749	0.951	1.77E-57	Cbr3	SEC_Ageddown	SEC
2.09E-21	-0.598339	0.066	0.227	5.00E-17	Atf3	SEC_Ageddown	SEC
1.85E-156	-0.60044	0.991	1	4.43E-152	Rps8	SEC_Ageddown	SEC
8.70E-103	-0.607024	0.962	0.999	2.09E-98	Rpl12	SEC_Ageddown	SEC
7.14E-157	-0.610406	1	1	1.71E-152	mt-Co3	SEC_Ageddown	SEC
1.87E-34	-0.61457	0.817	0.909	4.49E-30	Gm94	SEC_Ageddown	SEC
2.24E-112	-0.618023	0.92	0.999	5.36E-108	Itm2b	SEC_Ageddown	SEC
2.54E-38	-0.620413	0.447	0.697	6.10E-34	Cst6	SEC_Ageddown	SEC
1.01E-49	-0.632817	0.205	0.523	2.43E-45	Gadd45g	SEC_Ageddown	SEC
2.37E-10	-0.633758	0.187	0.315	5.68E-06	Krt16	SEC_Ageddown	SEC
1.42E-76	-0.637592	0.863	0.983	3.40E-72	Serpinb5	SEC_Ageddown	SEC
1.89E-101	-0.647656	0.915	0.995	4.53E-97	Rab25	SEC_Ageddown	SEC
5.77E-67	-0.658534	0.545	0.843	1.38E-62	Tpm2	SEC_Ageddown	SEC
1.16E-92	-0.660138	0.912	0.994	2.78E-88	Lgals3	SEC_Ageddown	SEC
1.36E-85	-0.666915	0.481	0.832	3.27E-81	Cacybp	SEC_Ageddown	SEC
4.73E-46	-0.671012	0.706	0.879	1.13E-41	Sptssb	SEC_Ageddown	SEC
1.41E-98	-0.675305	0.759	0.983	3.39E-94	Sumo2	SEC_Ageddown	SEC
6.94E-205	-0.679955	1	1	1.66E-200	mt-Atp6	SEC_Ageddown	SEC
9.89E-48	-0.688869	0.589	0.828	2.37E-43	Rbp2	SEC_Ageddown	SEC
2.27E-45	-0.69896	0.537	0.779	5.45E-41	Ctnnbip1	SEC_Ageddown	SEC
1.92E-67	-0.710618	0.719	0.935	4.61E-63	Hspa5	SEC_Ageddown	SEC
6.87E-32	-0.712279	0.469	0.699	1.65E-27	Junb	SEC_Ageddown	SEC
2.58E-78	-0.714476	0.406	0.768	6.19E-74	Dnajb1	SEC_Ageddown	SEC
5.59E-66	-0.718689	0.977	0.993	1.34E-61	Dmkn	SEC_Ageddown	SEC
7.10E-81	-0.722131	0.58	0.89	1.70E-76	Manf	SEC_Ageddown	SEC
1.76E-25	-0.72296	0.301	0.524	4.23E-21	Serpinb12	SEC_Ageddown	SEC
3.20E-25	-0.731751	0.181	0.383	7.67E-21	Dusp1	SEC_Ageddown	SEC
2.24E-66	-0.735003	0.26	0.64	5.38E-62	Ifi202b	SEC_Ageddown	SEC
6.15E-31	-0.746939	0.37	0.599	1.47E-26	Dsg1a	SEC_Ageddown	SEC
1.96E-182	-0.747969	0.969	0.999	4.71E-178	Rpl21	SEC_Ageddown	SEC
1.68E-127	-0.753768	0.902	0.999	4.03E-123	Cd9	SEC_Ageddown	SEC
4.53E-43	-0.759885	0.427	0.72	1.09E-38	Spink5	SEC_Ageddown	SEC
4.17E-71	-0.772311	0.854	0.974	1.00E-66	Prdx6	SEC_Ageddown	SEC
1.64E-182	-0.799443	0.952	1	3.93E-178	H3f3a	SEC_Ageddown	SEC
3.87E-148	-0.800742	0.918	0.999	9.29E-144	Hspa8	SEC_Ageddown	SEC
1.45E-171	-0.811225	0.982	0.999	3.47E-167	Rps12	SEC_Ageddown	SEC
2.43E-57	-0.857481	0.839	0.97	5.83E-53	Adh7	SEC_Ageddown	SEC
2.68E-27	-0.86771	0.215	0.456	6.43E-23	Krt75	SEC_Ageddown	SEC
1.32E-104	-0.878745	0.569	0.938	3.17E-100	Bpifb1	SEC_Ageddown	SEC
1.66E-122	-0.905657	0.731	0.97	3.97E-118	Capns2	SEC_Ageddown	SEC
2.07E-210	-0.917391	0.95	0.999	4.97E-206	Hsp90ab1	SEC_Ageddown	SEC
4.43E-26	-0.921676	0.351	0.583	1.06E-21	Krt6b	SEC_Ageddown	SEC
3.07E-15	-0.960565	0.162	0.308	7.37E-11	Serpinb3a	SEC_Ageddown	SEC
9.57E-74	-0.976469	0.418	0.782	2.29E-69	Id1	SEC_Ageddown	SEC
1.36E-127	-0.979138	0.531	0.916	3.26E-123	Anxa8	SEC_Ageddown	SEC
1.93E-190	-1.008155	0.892	0.998	4.62E-186	Ubb	SEC_Ageddown	SEC
6.60E-25	-1.034763	0.265	0.476	1.58E-20	Ly6g6c	SEC_Ageddown	SEC

3.55E-35	-1.123298	0.269	0.506	8.52E-31	Jun	SEC_Ageddown	SEC
1.34E-187	-1.13026	0.186	0.847	3.23E-183	Tmem59	SEC_Ageddown	SEC
4.79E-152	-1.14544	0.537	0.92	1.15E-147	Dnaja1	SEC_Ageddown	SEC
3.07E-41	-1.150501	0.579	0.918	7.36E-37	Krt17	SEC_Ageddown	SEC
1.25E-154	-1.158242	0.352	0.839	3.01E-150	Hsph1	SEC_Ageddown	SEC
3.56E-47	-1.229389	0.135	0.43	8.54E-43	Fos	SEC_Ageddown	SEC
1.38E-172	-1.273053	0.975	0.999	3.30E-168	Hspb1	SEC_Ageddown	SEC
5.20E-138	-1.368087	0.709	0.99	1.25E-133	Lipf	SEC_Ageddown	SEC
4.87E-162	-1.512813	0.703	0.993	1.17E-157	Sbpl	SEC_Ageddown	SEC
1.46E-260	-1.725031	0.727	0.989	3.51E-256	Hsp90aa1	SEC_Ageddown	SEC
1.74E-247	-1.799378	0.696	0.979	4.17E-243	Hspa1b	SEC_Ageddown	SEC
2.27E-223	-1.824881	0.33	0.902	5.45E-219	Hspa1a	SEC_Ageddown	SEC
6.04E-112	2.8842836	0.61	0	1.45E-107	Xist	TPC_Agedup	TPC
1.94E-93	2.3585862	1	1	4.66E-89	Gm42418	TPC_Agedup	TPC
5.26E-87	2.0311205	0.985	0.772	1.26E-82	mt-Atp8	TPC_Agedup	TPC
4.61E-32	1.688011	0.545	0.174	1.11E-27	Ifi27l2a	TPC_Agedup	TPC
2.36E-18	1.6798515	0.84	0.684	5.66E-14	Crip1	TPC_Agedup	TPC
2.64E-47	1.613801	0.295	0.006	6.32E-43	Gm10260	TPC_Agedup	TPC
2.72E-85	1.4437359	1	0.999	6.53E-81	mt-Nd4l	TPC_Agedup	TPC
1.52E-85	1.3310395	1	0.99	3.65E-81	Gm10076	TPC_Agedup	TPC
5.72E-21	1.3177919	0.75	0.498	1.37E-16	H2-K1	TPC_Agedup	TPC
4.75E-14	1.3099769	0.49	0.266	1.14E-09	Sfrp1	TPC_Agedup	TPC
2.82E-32	1.2883284	0.745	0.393	6.76E-28	4631405K08Rik	TPC_Agedup	TPC
2.08E-13	1.2875338	0.79	0.823	4.99E-09	Krtdap	TPC_Agedup	TPC
3.03E-07	1.2651127	0.1	0.022	0.007261	Lyz2	TPC_Agedup	TPC
1.76E-26	1.2336057	0.855	0.673	4.22E-22	H2-D1	TPC_Agedup	TPC
2.04E-21	1.1408944	0.325	0.081	4.89E-17	Col16a1	TPC_Agedup	TPC
2.59E-43	1.1266278	0.42	0.057	6.20E-39	1600014C10Rik	TPC_Agedup	TPC
1.04E-21	1.0755637	0.675	0.379	2.49E-17	B2m	TPC_Agedup	TPC
3.75E-26	1.032704	0.385	0.094	8.99E-22	Gm26870	TPC_Agedup	TPC
1.54E-16	0.9977275	0.58	0.308	3.70E-12	Ly6a	TPC_Agedup	TPC
1.71E-86	0.9744946	1	1	4.09E-82	Rpl38	TPC_Agedup	TPC
1.50E-60	0.9678757	0.345	0	3.60E-56	Tff2	TPC_Agedup	TPC
1.01E-89	0.9518899	1	1	2.41E-85	Rps28	TPC_Agedup	TPC
1.13E-07	0.9429814	0.495	0.348	0.002721	Tmem176a	TPC_Agedup	TPC
4.62E-22	0.9326265	0.82	0.672	1.11E-17	Tmsb10	TPC_Agedup	TPC
1.07E-49	0.8876662	0.98	0.963	2.56E-45	Uba52	TPC_Agedup	TPC
7.90E-08	0.8797775	0.75	0.657	0.001894	Tmem176b	TPC_Agedup	TPC
3.03E-18	0.8744954	0.775	0.53	7.26E-14	Ly6e	TPC_Agedup	TPC
8.83E-19	0.8608541	0.935	0.81	2.12E-14	Ly6d	TPC_Agedup	TPC
9.16E-20	0.8481533	0.725	0.523	2.20E-15	Erh	TPC_Agedup	TPC
2.80E-24	0.8168733	0.86	0.738	6.72E-20	Snrpg	TPC_Agedup	TPC
2.53E-86	0.8028101	1	1	6.08E-82	Rps29	TPC_Agedup	TPC
3.56E-18	0.7952463	0.855	0.823	8.53E-14	Atp5k	TPC_Agedup	TPC
3.84E-86	0.7904513	1	1	9.21E-82	Rpl37a	TPC_Agedup	TPC
9.71E-17	0.7853835	0.725	0.549	2.33E-12	Ndufa3	TPC_Agedup	TPC
3.01E-64	0.7842927	1	0.999	7.22E-60	Rpl35	TPC_Agedup	TPC
1.98E-66	0.7810429	1	1	4.75E-62	Rps27	TPC_Agedup	TPC
1.54E-06	0.7680116	0.57	0.443	0.037002	Cyp2f2	TPC_Agedup	TPC
4.89E-31	0.7652744	0.97	0.935	1.17E-26	Uqcr11	TPC_Agedup	TPC
4.96E-11	0.7404204	0.545	0.361	1.19E-06	Gm47283	TPC_Agedup	TPC
8.02E-18	0.7355763	0.77	0.593	1.92E-13	Ktn1	TPC_Agedup	TPC
2.17E-22	0.7355753	0.835	0.714	5.20E-18	Ndufa1	TPC_Agedup	TPC
5.93E-22	0.716624	0.445	0.143	1.42E-17	Sparc	TPC_Agedup	TPC
2.33E-15	0.7158577	0.815	0.73	5.59E-11	Usmg5	TPC_Agedup	TPC
3.75E-15	0.7146008	0.645	0.396	8.99E-11	Itm2c	TPC_Agedup	TPC
2.73E-26	0.7110447	0.91	0.753	6.55E-22	Spint2	TPC_Agedup	TPC
8.55E-15	0.7006675	0.49	0.258	2.05E-10	Gstp1	TPC_Agedup	TPC
1.48E-08	0.6763175	0.595	0.443	0.000354	Nbl1	TPC_Agedup	TPC

1.28E-17	0.666488	0.19	0.028	3.06E-13	Enho	TPC_Agedup	TPC
1.29E-18	0.6631476	0.965	0.96	3.09E-14	Ptms	TPC_Agedup	TPC
3.50E-63	0.6579065	1	1	8.38E-59	Rpl36	TPC_Agedup	TPC
4.31E-11	0.6571835	0.395	0.193	1.03E-06	Ptpru	TPC_Agedup	TPC
6.38E-15	0.6537239	0.44	0.199	1.53E-10	Snhg20	TPC_Agedup	TPC
5.87E-24	0.6527509	0.92	0.873	1.41E-19	Mrpl52	TPC_Agedup	TPC
2.43E-25	0.6510754	0.97	0.937	5.83E-21	Tomm7	TPC_Agedup	TPC
1.95E-27	0.6487482	0.955	0.959	4.68E-23	Rpl27	TPC_Agedup	TPC
2.60E-11	0.6460185	0.885	0.841	6.23E-07	Epcam	TPC_Agedup	TPC
7.58E-07	0.61873	0.68	0.548	0.018179	Slc6a6	TPC_Agedup	TPC
5.79E-14	0.609518	0.89	0.837	1.39E-09	Son	TPC_Agedup	TPC
9.21E-12	0.6093071	0.7	0.541	2.21E-07	Ewsr1	TPC_Agedup	TPC
4.74E-08	0.6084062	0.315	0.159	0.001137	Gpm6b	TPC_Agedup	TPC
2.10E-30	0.6078352	0.255	0.021	5.03E-26	Muc5b	TPC_Agedup	TPC
6.46E-11	0.606816	0.695	0.542	1.55E-06	Cst3	TPC_Agedup	TPC
1.74E-13	0.6043715	0.27	0.086	4.17E-09	Calml3	TPC_Agedup	TPC
4.24E-10	0.6021499	0.515	0.335	1.02E-05	Nfe2l1	TPC_Agedup	TPC
5.01E-09	0.5942559	0.595	0.433	0.00012	Ifi27	TPC_Agedup	TPC
1.76E-09	0.5905554	0.635	0.476	4.21E-05	Ece1	TPC_Agedup	TPC
1.29E-12	0.5887373	0.115	0.012	3.09E-08	Mia	TPC_Agedup	TPC
1.70E-55	0.5868355	1	1	4.09E-51	Rpl37	TPC_Agedup	TPC
1.59E-07	0.5824305	0.44	0.28	0.003807	Lgr6	TPC_Agedup	TPC
2.78E-08	0.5801595	0.575	0.425	0.000666	H1f0	TPC_Agedup	TPC
4.24E-11	0.5766909	0.755	0.619	1.02E-06	Ptprs	TPC_Agedup	TPC
1.98E-12	0.5764104	0.785	0.641	4.76E-08	Clstn1	TPC_Agedup	TPC
1.66E-11	0.5751211	0.395	0.189	3.98E-07	Car12	TPC_Agedup	TPC
1.80E-13	0.5732762	0.595	0.364	4.32E-09	Lars2	TPC_Agedup	TPC
1.47E-16	0.5644206	0.95	0.964	3.52E-12	Atp1a1	TPC_Agedup	TPC
2.36E-18	0.5594998	0.935	0.941	5.65E-14	Elob	TPC_Agedup	TPC
5.39E-08	0.5561678	0.745	0.716	0.001292	Dsc3	TPC_Agedup	TPC
1.52E-08	0.5537368	0.385	0.218	0.000364	Moxd1	TPC_Agedup	TPC
8.58E-07	0.553567	0.195	0.083	0.020586	Plcb4	TPC_Agedup	TPC
2.17E-16	0.5468791	0.915	0.927	5.21E-12	Sec61g	TPC_Agedup	TPC
1.05E-08	0.5409742	0.65	0.523	0.000251	Ndufv3	TPC_Agedup	TPC
5.06E-07	0.5347394	0.285	0.143	0.012126	Krt8	TPC_Agedup	TPC
1.25E-12	0.533487	0.795	0.691	2.99E-08	Ndufb2	TPC_Agedup	TPC
3.00E-08	0.531805	0.7	0.625	0.000719	Atp6v1g1	TPC_Agedup	TPC
3.72E-07	0.5304375	0.49	0.359	0.008922	Setd5	TPC_Agedup	TPC
3.88E-11	0.5277397	0.705	0.52	9.31E-07	Dpm3	TPC_Agedup	TPC
1.83E-15	0.5255887	0.275	0.077	4.39E-11	Snhg9	TPC_Agedup	TPC
2.34E-16	0.5251445	1	0.996	5.61E-12	mt-Nd5	TPC_Agedup	TPC
3.92E-08	0.5193582	0.58	0.429	0.00094	Plxnb1	TPC_Agedup	TPC
1.37E-07	0.5151571	0.59	0.426	0.003283	1810022K09Rik	TPC_Agedup	TPC
1.58E-11	0.5127113	0.78	0.644	3.79E-07	Iqgap1	TPC_Agedup	TPC
2.22E-09	0.5109919	0.71	0.601	5.33E-05	Tmem256	TPC_Agedup	TPC
1.87E-08	0.51018	0.155	0.044	0.000448	Bgn	TPC_Agedup	TPC
1.13E-10	0.5096499	0.865	0.79	2.71E-06	Ndufa11	TPC_Agedup	TPC
3.70E-09	0.5079326	0.47	0.28	8.88E-05	Scd2	TPC_Agedup	TPC
1.10E-09	0.5068252	0.745	0.6	2.63E-05	Ddx3x	TPC_Agedup	TPC
8.69E-08	0.5036534	0.4	0.236	0.002085	Furin	TPC_Agedup	TPC
1.16E-08	0.5021236	0.83	0.81	0.000279	Dbi	TPC_Agedup	TPC
2.34E-48	0.4971372	1	1	5.61E-44	Rps19	TPC_Agedup	TPC
1.56E-06	0.4965696	0.375	0.225	0.037373	Tmprss4	TPC_Agedup	TPC
1.93E-08	0.4962135	0.425	0.251	0.000464	Sema3f	TPC_Agedup	TPC
5.46E-09	0.49586	0.8	0.731	0.000131	Sdc1	TPC_Agedup	TPC
3.14E-10	0.4952206	0.785	0.648	7.54E-06	Psap	TPC_Agedup	TPC
4.36E-08	0.4873034	0.42	0.248	0.001046	Hs6st1	TPC_Agedup	TPC
3.99E-09	0.4832781	0.21	0.073	9.56E-05	Selenom	TPC_Agedup	TPC
6.16E-09	0.4823332	0.67	0.541	0.000148	Naa38	TPC_Agedup	TPC

4.99E-07	0.4807843	0.475	0.33	0.011962	Scarb2	TPC_Agedup	TPC
4.64E-08	0.4803509	0.37	0.203	0.001113	Slc39a1	TPC_Agedup	TPC
6.03E-34	0.4781481	1	1	1.45E-29	Rpl41	TPC_Agedup	TPC
1.26E-10	0.4757531	0.92	0.943	3.03E-06	Ndufa4	TPC_Agedup	TPC
8.16E-19	0.47529	0.305	0.076	1.96E-14	Gm10036	TPC_Agedup	TPC
6.84E-10	0.4721854	0.75	0.647	1.64E-05	Bola2	TPC_Agedup	TPC
3.17E-12	0.4673407	0.9	0.901	7.61E-08	Minos1	TPC_Agedup	TPC
1.47E-14	0.4647456	0.93	0.966	3.52E-10	Cox6b1	TPC_Agedup	TPC
1.00E-07	0.4637278	0.44	0.272	0.0024	C77080	TPC_Agedup	TPC
8.43E-15	0.4617508	0.985	0.974	2.02E-10	Atp5e	TPC_Agedup	TPC
2.17E-07	0.4614814	0.725	0.604	0.00521	Gtf2i	TPC_Agedup	TPC
1.18E-09	0.4577095	0.44	0.241	2.82E-05	Grcc10	TPC_Agedup	TPC
5.74E-15	0.457629	0.96	0.982	1.38E-10	Cox6c	TPC_Agedup	TPC
3.56E-15	0.454262	0.29	0.084	8.54E-11	Plbd1	TPC_Agedup	TPC
2.68E-08	0.454086	0.925	0.898	0.000644	Nedd4	TPC_Agedup	TPC
1.62E-13	0.4514939	0.91	0.857	3.89E-09	Ndufa2	TPC_Agedup	TPC
1.04E-09	0.449163	0.765	0.648	2.50E-05	Mrpl33	TPC_Agedup	TPC
2.20E-08	0.4480079	0.86	0.87	0.000529	Uqcrq	TPC_Agedup	TPC
9.89E-10	0.4471312	0.985	0.975	2.37E-05	Ifitm3	TPC_Agedup	TPC
1.41E-08	0.4425982	0.48	0.297	0.000338	Pet100	TPC_Agedup	TPC
2.80E-11	0.4408644	0.125	0.019	6.71E-07	Sp100	TPC_Agedup	TPC
3.66E-11	0.4378214	0.88	0.844	8.78E-07	Ndufa7	TPC_Agedup	TPC
2.47E-11	0.4374089	0.945	0.961	5.93E-07	Sem1	TPC_Agedup	TPC
4.21E-09	0.4346793	0.33	0.159	0.000101	Chchd7	TPC_Agedup	TPC
2.49E-07	0.4305657	0.725	0.594	0.005965	Hnrnpl	TPC_Agedup	TPC
5.85E-07	0.4294337	0.76	0.709	0.014021	Ost4	TPC_Agedup	TPC
2.39E-07	0.4255054	0.995	0.996	0.005739	Fxyd3	TPC_Agedup	TPC
3.72E-08	0.4179418	0.14	0.037	0.000892	Sult5a1	TPC_Agedup	TPC
5.83E-09	0.4161058	0.72	0.599	0.00014	Mrps21	TPC_Agedup	TPC
8.13E-08	0.4129991	0.925	0.908	0.001949	Lmna	TPC_Agedup	TPC
5.32E-07	0.4126216	0.21	0.09	0.012751	Plppr4	TPC_Agedup	TPC
5.22E-12	0.411399	0.97	0.954	1.25E-07	Selenow	TPC_Agedup	TPC
5.32E-12	0.4065868	0.175	0.039	1.28E-07	Psmb8	TPC_Agedup	TPC
1.20E-06	0.4047937	0.38	0.23	0.028765	Ggh	TPC_Agedup	TPC
2.81E-07	0.399594	0.79	0.754	0.006736	Ndufb8	TPC_Agedup	TPC
3.42E-09	0.3964931	0.295	0.132	8.20E-05	Tomm6	TPC_Agedup	TPC
5.86E-08	0.3963817	0.86	0.863	0.001406	Sec61b	TPC_Agedup	TPC
2.84E-07	0.3959675	0.355	0.197	0.006812	Rpl27-ps3	TPC_Agedup	TPC
5.53E-07	0.3956079	0.745	0.687	0.013271	Romo1	TPC_Agedup	TPC
9.11E-10	0.3906187	0.96	0.968	2.18E-05	Cox7a2	TPC_Agedup	TPC
2.15E-11	0.3878578	0.955	0.96	5.16E-07	Atp5j2	TPC_Agedup	TPC
1.61E-06	0.3853809	0.765	0.761	0.038523	Ndufc2	TPC_Agedup	TPC
1.27E-06	0.381683	0.645	0.554	0.03045	Lsm7	TPC_Agedup	TPC
4.30E-27	0.3786918	1	1	1.03E-22	Rps20	TPC_Agedup	TPC
3.64E-07	0.3783959	0.355	0.204	0.008734	Scrib	TPC_Agedup	TPC
1.08E-33	0.3765027	1	1	2.58E-29	Rpl39	TPC_Agedup	TPC
1.54E-06	0.376048	0.38	0.234	0.036841	Marf1	TPC_Agedup	TPC
2.92E-07	0.3735129	0.295	0.145	0.006998	Map2k7	TPC_Agedup	TPC
2.42E-07	0.3734277	0.865	0.846	0.005796	Ndufa13	TPC_Agedup	TPC
1.45E-09	0.3728118	0.935	0.938	3.48E-05	Snrpe	TPC_Agedup	TPC
3.33E-13	0.3687235	0.12	0.012	7.98E-09	Islr	TPC_Agedup	TPC
6.40E-07	0.3653626	0.225	0.099	0.015344	Gpc1	TPC_Agedup	TPC
9.50E-07	0.3543571	0.35	0.199	0.022786	Gdi1	TPC_Agedup	TPC
1.92E-15	0.3536619	0.995	0.997	4.60E-11	Rpl36a	TPC_Agedup	TPC
1.49E-08	0.3495789	0.945	0.903	0.000357	Uqcr10	TPC_Agedup	TPC
8.50E-07	0.34669	0.175	0.065	0.020394	Cyba	TPC_Agedup	TPC
1.84E-13	0.3425708	0.13	0.015	4.41E-09	Adgrb2	TPC_Agedup	TPC
6.34E-07	0.3421308	0.93	0.903	0.0152	Ubl5	TPC_Agedup	TPC
3.04E-09	0.3416348	0.125	0.026	7.29E-05	Pld1	TPC_Agedup	TPC

5.14E-08	0.3407762	0.14	0.039	0.001232	Meis2	TPC_Agedup	TPC
1.98E-16	0.3389767	1	1	4.74E-12	Rps2	TPC_Agedup	TPC
1.73E-06	0.336888	0.76	0.739	0.041484	Ndufb7	TPC_Agedup	TPC
7.38E-07	0.3360687	0.265	0.127	0.017707	Cystm1	TPC_Agedup	TPC
1.84E-26	0.3358611	1	1	4.42E-22	Rpl30	TPC_Agedup	TPC
3.10E-07	0.335827	0.81	0.763	0.007439	Ndufa6	TPC_Agedup	TPC
2.91E-10	0.3297483	0.98	0.997	6.98E-06	Oaz1	TPC_Agedup	TPC
1.24E-10	0.327602	0.98	0.983	2.97E-06	Atpif1	TPC_Agedup	TPC
5.75E-09	0.324191	0.945	0.946	0.000138	Ndufb9	TPC_Agedup	TPC
3.14E-08	0.3211165	0.185	0.063	0.000753	Cbarp	TPC_Agedup	TPC
1.36E-10	0.3198746	0.14	0.028	3.26E-06	Samd9l	TPC_Agedup	TPC
2.86E-08	0.3193883	0.97	0.979	0.000686	Atp5l	TPC_Agedup	TPC
5.94E-08	0.3126413	0.97	0.977	0.001425	Cox7c	TPC_Agedup	TPC
7.82E-07	0.304624	0.89	0.914	0.018755	Atp5j	TPC_Agedup	TPC
1.99E-15	0.2913375	0.16	0.021	4.78E-11	Gm8797	TPC_Agedup	TPC
7.72E-11	0.2816513	0.125	0.021	1.85E-06	Psmb9	TPC_Agedup	TPC
1.16E-07	0.280247	0.175	0.059	0.002779	Dhcr24	TPC_Agedup	TPC
3.10E-19	0.2699817	1	1	7.43E-15	Rpl35a	TPC_Agedup	TPC
9.15E-07	0.2699328	0.12	0.034	0.021949	Gm1673	TPC_Agedup	TPC
2.06E-15	0.2449485	1	1	4.94E-11	Rpl11	TPC_Agedup	TPC
2.05E-06	0.2374581	0.175	0.068	0.04909	Angptl2	TPC_Agedup	TPC
1.64E-06	0.2344246	0.105	0.028	0.03929	Inha	TPC_Agedup	TPC
1.07E-08	0.2298422	0.1	0.017	0.000256	Tek	TPC_Agedup	TPC
2.47E-08	0.2285075	0.1	0.018	0.000593	Slfn2	TPC_Agedup	TPC
1.09E-13	0.2198762	0.995	1	2.62E-09	Rpl34	TPC_Agedup	TPC
9.01E-11	0.2093489	0.995	1	2.16E-06	Rps7	TPC_Agedup	TPC
7.55E-10	0.2072605	1	1	1.81E-05	Rps26	TPC_Agedup	TPC
8.86E-10	0.2043569	0.105	0.015	2.12E-05	Stra6	TPC_Agedup	TPC
1.28E-07	-0.210719	0.98	0.999	0.003061	Rpl5	TPC_Ageddown	TPC
1.78E-14	-0.224161	0.995	1	4.27E-10	Rps16	TPC_Ageddown	TPC
5.27E-08	-0.23097	0.985	0.997	0.001264	Btf3	TPC_Ageddown	TPC
1.18E-11	-0.235618	0.995	1	2.82E-07	Rplp2	TPC_Ageddown	TPC
1.72E-06	-0.23565	0.02	0.145	0.041254	Ogfrl1	TPC_Ageddown	TPC
1.20E-06	-0.241491	0.935	0.983	0.028736	Eef1g	TPC_Ageddown	TPC
3.51E-09	-0.245125	0.995	1	8.41E-05	Naca	TPC_Ageddown	TPC
2.85E-18	-0.250226	1	1	6.84E-14	Rpl28	TPC_Ageddown	TPC
1.53E-06	-0.250716	0.05	0.192	0.036584	Slit3	TPC_Ageddown	TPC
6.32E-07	-0.255791	0.915	0.99	0.015156	Dynll1	TPC_Ageddown	TPC
1.40E-12	-0.262495	1	1	3.35E-08	Rpl3	TPC_Ageddown	TPC
8.18E-07	-0.275011	0.56	0.785	0.019611	Taldo1	TPC_Ageddown	TPC
1.48E-06	-0.278971	0.1	0.272	0.035586	Banp	TPC_Ageddown	TPC
2.16E-10	-0.282084	0.33	0.61	5.18E-06	Dnajb1	TPC_Ageddown	TPC
3.78E-15	-0.284404	0.99	1	9.06E-11	Rpl7	TPC_Ageddown	TPC
1.70E-06	-0.286314	0.315	0.521	0.040785	Snrnp48	TPC_Ageddown	TPC
1.95E-06	-0.29006	0.44	0.648	0.046751	Naa50	TPC_Ageddown	TPC
4.80E-07	-0.3067	0.2	0.401	0.011523	Ppa1	TPC_Ageddown	TPC
3.92E-12	-0.307675	0.985	0.997	9.41E-08	Rpl4	TPC_Ageddown	TPC
1.99E-07	-0.309464	0.27	0.466	0.00477	Lamtor1	TPC_Ageddown	TPC
6.01E-16	-0.310494	1	1	1.44E-11	mt-Nd1	TPC_Ageddown	TPC
4.55E-07	-0.31089	0.11	0.291	0.010901	Rsrc1	TPC_Ageddown	TPC
1.21E-06	-0.310944	0.835	0.952	0.02906	Set	TPC_Ageddown	TPC
1.91E-07	-0.311876	0.15	0.346	0.00459	Cks2	TPC_Ageddown	TPC
9.61E-09	-0.31328	0.865	0.96	0.00023	Atp5b	TPC_Ageddown	TPC
1.85E-07	-0.315359	0.7	0.848	0.004444	Laptm4a	TPC_Ageddown	TPC
1.80E-08	-0.316386	0.865	0.96	0.000432	Arf5	TPC_Ageddown	TPC
9.88E-07	-0.316418	0.225	0.432	0.023701	Lsm2	TPC_Ageddown	TPC
2.88E-07	-0.324711	0.345	0.566	0.006903	Mrpl42	TPC_Ageddown	TPC
1.37E-25	-0.327013	1	1	3.28E-21	Rpl8	TPC_Ageddown	TPC
1.51E-07	-0.327059	0.085	0.272	0.00363	Nr1d1	TPC_Ageddown	TPC

7.31E-07	-0.327934	0.78	0.898	0.01753	Ran	TPC_Ageddown	TPC
7.58E-07	-0.32858	0.28	0.494	0.018182	Chordc1	TPC_Ageddown	TPC
8.93E-07	-0.329805	0.295	0.505	0.021405	Elavl1	TPC_Ageddown	TPC
4.56E-07	-0.331348	0.58	0.778	0.010929	Srsf11	TPC_Ageddown	TPC
1.79E-06	-0.332406	0.545	0.703	0.042848	Zfp91	TPC_Ageddown	TPC
5.62E-08	-0.333231	0.365	0.593	0.001347	Rbbp4	TPC_Ageddown	TPC
1.11E-22	-0.334313	0.995	1	2.65E-18	Rps4x	TPC_Ageddown	TPC
2.00E-07	-0.337242	0.345	0.599	0.004801	Egr1	TPC_Ageddown	TPC
2.31E-07	-0.337486	0.45	0.657	0.005551	P4hb	TPC_Ageddown	TPC
5.87E-07	-0.338845	0.11	0.281	0.014085	Mcm4	TPC_Ageddown	TPC
7.58E-12	-0.343293	0.99	0.996	1.82E-07	Rps17	TPC_Ageddown	TPC
4.00E-07	-0.343782	0.46	0.692	0.009604	Ube2s	TPC_Ageddown	TPC
1.15E-06	-0.344325	0.275	0.466	0.027609	Gnl3	TPC_Ageddown	TPC
3.28E-16	-0.344813	1	1	7.87E-12	Ptma	TPC_Ageddown	TPC
1.58E-07	-0.345705	0.2	0.414	0.003798	Eva1c	TPC_Ageddown	TPC
1.61E-06	-0.349178	0.305	0.49	0.038523	Gtpbp4	TPC_Ageddown	TPC
8.10E-07	-0.350022	0.535	0.724	0.019424	Eif6	TPC_Ageddown	TPC
3.78E-07	-0.352041	0.52	0.726	0.009056	Ankrd11	TPC_Ageddown	TPC
1.44E-07	-0.352128	0.19	0.388	0.003454	Cenpv	TPC_Ageddown	TPC
6.51E-07	-0.353751	0.365	0.557	0.015601	Mtpn	TPC_Ageddown	TPC
3.89E-07	-0.356375	0.41	0.611	0.009325	Banf1	TPC_Ageddown	TPC
2.39E-20	-0.357999	0.985	0.999	5.73E-16	Rpl10	TPC_Ageddown	TPC
1.15E-14	-0.360785	0.98	0.999	2.75E-10	Rpl29	TPC_Ageddown	TPC
3.41E-08	-0.362888	0.325	0.586	0.000819	Mrpl21	TPC_Ageddown	TPC
8.22E-07	-0.364359	0.615	0.778	0.019717	Psmb2	TPC_Ageddown	TPC
7.36E-07	-0.365157	0.475	0.647	0.017648	Ndufa10	TPC_Ageddown	TPC
2.26E-09	-0.37013	0.025	0.204	5.41E-05	Aff3	TPC_Ageddown	TPC
2.74E-26	-0.371577	0.995	1	6.57E-22	Rps18	TPC_Ageddown	TPC
6.52E-07	-0.372015	0.38	0.579	0.015625	Cbr3	TPC_Ageddown	TPC
3.47E-12	-0.372297	0.925	0.977	8.32E-08	mt-Nd3	TPC_Ageddown	TPC
4.02E-08	-0.373106	0.42	0.628	0.000964	Tmpo	TPC_Ageddown	TPC
7.98E-08	-0.373815	0.12	0.31	0.001913	Mcm6	TPC_Ageddown	TPC
4.53E-09	-0.373911	0.4	0.628	0.000109	Snx3	TPC_Ageddown	TPC
1.98E-08	-0.374625	0.075	0.259	0.000475	Sdf2l1	TPC_Ageddown	TPC
1.57E-07	-0.375085	0.7	0.877	0.003753	Atp5c1	TPC_Ageddown	TPC
1.15E-07	-0.375092	0.67	0.837	0.00276	Ptges3	TPC_Ageddown	TPC
8.63E-07	-0.378057	0.485	0.683	0.020689	Psmd8	TPC_Ageddown	TPC
5.77E-08	-0.378376	0.59	0.794	0.001384	Rdx	TPC_Ageddown	TPC
3.92E-08	-0.378738	0.39	0.632	0.00094	Tsc22d3	TPC_Ageddown	TPC
5.87E-10	-0.378922	0.82	0.92	1.41E-05	Hnrnpa1	TPC_Ageddown	TPC
4.16E-36	-0.381579	1	1	9.98E-32	Rpl13	TPC_Ageddown	TPC
1.52E-11	-0.381811	0.91	0.983	3.65E-07	Serbp1	TPC_Ageddown	TPC
1.28E-08	-0.382256	0.455	0.674	0.000306	Arf4	TPC_Ageddown	TPC
4.61E-08	-0.382611	0.165	0.378	0.001106	Nr1d2	TPC_Ageddown	TPC
1.67E-08	-0.389621	0.73	0.888	0.000399	Sfr1	TPC_Ageddown	TPC
5.33E-08	-0.390667	0.13	0.328	0.001278	Cav1	TPC_Ageddown	TPC
1.48E-07	-0.391273	0.525	0.742	0.003551	Rab25	TPC_Ageddown	TPC
5.87E-09	-0.392007	0.795	0.946	0.000141	Eif3h	TPC_Ageddown	TPC
2.14E-08	-0.39402	0.485	0.721	0.000512	Tra2a	TPC_Ageddown	TPC
1.50E-08	-0.394131	0.695	0.869	0.000361	Rhoa	TPC_Ageddown	TPC
7.47E-29	-0.394165	1	1	1.79E-24	Rpsa	TPC_Ageddown	TPC
8.31E-10	-0.39486	0.765	0.887	1.99E-05	Hnrnpf	TPC_Ageddown	TPC
1.67E-07	-0.400737	0.64	0.834	0.003993	Mdh1	TPC_Ageddown	TPC
4.25E-09	-0.402045	0.695	0.843	0.000102	Nap1l1	TPC_Ageddown	TPC
3.71E-09	-0.40227	0.11	0.323	8.89E-05	Rassf1	TPC_Ageddown	TPC
2.62E-10	-0.402698	0.06	0.277	6.28E-06	Ddx3y	TPC_Ageddown	TPC
6.64E-08	-0.404066	0.21	0.421	0.001593	Psat1	TPC_Ageddown	TPC
1.38E-09	-0.404445	0.315	0.557	3.31E-05	Lsm12	TPC_Ageddown	TPC
4.61E-09	-0.404966	0.165	0.388	0.00011	Med19	TPC_Ageddown	TPC

9.03E-07	-0.405684	0.24	0.43	0.021664	Them5	TPC_Ageddown	TPC
4.21E-09	-0.405689	0.64	0.828	0.000101	Arpc3	TPC_Ageddown	TPC
1.99E-06	-0.405833	0.195	0.388	0.047735	Id2	TPC_Ageddown	TPC
2.56E-37	-0.406142	1	1	6.15E-33	Rpl26	TPC_Ageddown	TPC
6.05E-08	-0.407622	0.465	0.672	0.001451	Erp29	TPC_Ageddown	TPC
5.40E-29	-0.408959	0.99	1	1.29E-24	Rpl15	TPC_Ageddown	TPC
1.58E-08	-0.409921	0.45	0.657	0.00038	Magoh	TPC_Ageddown	TPC
4.80E-10	-0.41045	0.915	0.977	1.15E-05	Prdx1	TPC_Ageddown	TPC
1.42E-09	-0.412339	0.645	0.81	3.40E-05	Cct2	TPC_Ageddown	TPC
1.37E-31	-0.413223	1	1	3.29E-27	Rplp0	TPC_Ageddown	TPC
1.92E-10	-0.416009	0.77	0.902	4.62E-06	Anxa5	TPC_Ageddown	TPC
9.54E-14	-0.416098	0.43	0.806	2.29E-09	Krt13	TPC_Ageddown	TPC
2.00E-11	-0.419426	0.855	0.961	4.80E-07	Slc25a3	TPC_Ageddown	TPC
3.77E-09	-0.419441	0.09	0.297	9.05E-05	Wee1	TPC_Ageddown	TPC
3.54E-08	-0.419563	0.39	0.606	0.000848	Nudc	TPC_Ageddown	TPC
3.34E-08	-0.421923	0.03	0.192	0.000801	Prb1	TPC_Ageddown	TPC
3.35E-09	-0.423529	0.71	0.854	8.03E-05	Eif3i	TPC_Ageddown	TPC
1.70E-07	-0.428211	0.28	0.479	0.004077	Hlf	TPC_Ageddown	TPC
2.13E-09	-0.429529	0.46	0.69	5.12E-05	Rbm8a	TPC_Ageddown	TPC
4.17E-09	-0.429557	0.225	0.448	9.99E-05	Nasp	TPC_Ageddown	TPC
1.84E-06	-0.431514	0.21	0.407	0.04408	Bdnf	TPC_Ageddown	TPC
5.53E-10	-0.434577	0.335	0.597	1.33E-05	Psma1	TPC_Ageddown	TPC
2.69E-09	-0.434697	0.765	0.913	6.45E-05	Cltb	TPC_Ageddown	TPC
1.43E-10	-0.436272	0.43	0.724	3.43E-06	Serpinh1	TPC_Ageddown	TPC
7.67E-09	-0.43696	0.63	0.837	0.000184	S100a14	TPC_Ageddown	TPC
3.00E-10	-0.437202	0.275	0.524	7.19E-06	Gadd45gip1	TPC_Ageddown	TPC
3.02E-12	-0.442482	0.8	0.939	7.25E-08	Cox7a2l	TPC_Ageddown	TPC
2.01E-09	-0.446069	0.31	0.571	4.83E-05	Capns2	TPC_Ageddown	TPC
2.54E-10	-0.448707	0.225	0.491	6.08E-06	Ech1	TPC_Ageddown	TPC
6.52E-09	-0.449479	0.175	0.392	0.000156	Mphosph8	TPC_Ageddown	TPC
1.05E-07	-0.449622	0.41	0.604	0.002524	Ccnd1	TPC_Ageddown	TPC
1.72E-10	-0.450802	0.605	0.815	4.14E-06	Snu13	TPC_Ageddown	TPC
1.61E-09	-0.451845	0.625	0.819	3.87E-05	Zfp36l2	TPC_Ageddown	TPC
1.26E-10	-0.453056	0.485	0.743	3.02E-06	Cct8	TPC_Ageddown	TPC
1.62E-09	-0.453183	0.515	0.735	3.87E-05	Vdac2	TPC_Ageddown	TPC
4.61E-11	-0.453416	0.27	0.55	1.11E-06	Polr2h	TPC_Ageddown	TPC
2.13E-20	-0.454564	0.98	0.999	5.12E-16	Cox4i1	TPC_Ageddown	TPC
3.38E-11	-0.454708	0.56	0.815	8.09E-07	Eif3m	TPC_Ageddown	TPC
2.63E-33	-0.45635	1	1	6.32E-29	Rplp1	TPC_Ageddown	TPC
3.77E-09	-0.45664	0.115	0.324	9.04E-05	Arl4a	TPC_Ageddown	TPC
5.56E-10	-0.458673	0.675	0.839	1.33E-05	Hspd1	TPC_Ageddown	TPC
8.77E-10	-0.459606	0.69	0.872	2.10E-05	Prdx6	TPC_Ageddown	TPC
7.04E-10	-0.466218	0.435	0.69	1.69E-05	Arid5b	TPC_Ageddown	TPC
2.21E-08	-0.46633	0.56	0.792	0.00053	Cebpb	TPC_Ageddown	TPC
8.68E-10	-0.475554	0.705	0.899	2.08E-05	Hspa5	TPC_Ageddown	TPC
3.63E-31	-0.480777	0.995	1	8.71E-27	Rps6	TPC_Ageddown	TPC
1.97E-08	-0.482241	0.5	0.738	0.000472	Junb	TPC_Ageddown	TPC
6.21E-13	-0.484705	0.76	0.914	1.49E-08	Anp32b	TPC_Ageddown	TPC
5.60E-10	-0.486628	0.52	0.727	1.34E-05	Eif5b	TPC_Ageddown	TPC
4.06E-24	-0.487617	0.995	0.996	9.74E-20	Npm1	TPC_Ageddown	TPC
5.82E-21	-0.487708	0.98	1	1.40E-16	Cd9	TPC_Ageddown	TPC
6.22E-09	-0.487948	0.37	0.662	0.000149	Fos	TPC_Ageddown	TPC
1.48E-10	-0.488367	0.27	0.543	3.54E-06	Mettl23	TPC_Ageddown	TPC
6.61E-12	-0.490158	0.355	0.641	1.59E-07	Vapa	TPC_Ageddown	TPC
7.61E-11	-0.491491	0.41	0.676	1.83E-06	Rp9	TPC_Ageddown	TPC
4.37E-12	-0.497035	0.58	0.79	1.05E-07	Cct4	TPC_Ageddown	TPC
7.79E-12	-0.500895	0.41	0.688	1.87E-07	Prmt1	TPC_Ageddown	TPC
2.88E-07	-0.502822	0.225	0.432	0.006901	Sgk1	TPC_Ageddown	TPC
1.80E-10	-0.504381	0.47	0.701	4.33E-06	Cct3	TPC_Ageddown	TPC

1.43E-10	-0.504815	0.425	0.68	3.43E-06	Wnt4	TPC_Ageddown	TPC
7.66E-13	-0.506981	0.645	0.852	1.84E-08	Eif5	TPC_Ageddown	TPC
1.67E-15	-0.510299	0.765	0.942	4.01E-11	Pcbp2	TPC_Ageddown	TPC
1.94E-45	-0.512595	1	1	4.64E-41	Rpl17	TPC_Ageddown	TPC
1.04E-11	-0.51353	0.43	0.699	2.49E-07	Psmd7	TPC_Ageddown	TPC
2.85E-13	-0.517456	0.68	0.858	6.84E-09	Arpc2	TPC_Ageddown	TPC
4.41E-12	-0.519001	0.64	0.848	1.06E-07	Calr	TPC_Ageddown	TPC
7.88E-11	-0.523154	0.67	0.85	1.89E-06	Sox2	TPC_Ageddown	TPC
8.20E-13	-0.524207	0.645	0.83	1.97E-08	Srsf2	TPC_Ageddown	TPC
1.31E-16	-0.528381	0.88	0.975	3.14E-12	Hspe1	TPC_Ageddown	TPC
1.88E-16	-0.532669	0.82	0.957	4.50E-12	Hmgn1	TPC_Ageddown	TPC
1.90E-51	-0.533189	1	1	4.56E-47	Eef1a1	TPC_Ageddown	TPC
7.35E-17	-0.543909	0.805	0.959	1.76E-12	Eif5a	TPC_Ageddown	TPC
3.70E-15	-0.5558	0.53	0.808	8.88E-11	Pcbp1	TPC_Ageddown	TPC
1.10E-13	-0.561788	0.405	0.681	2.63E-09	Tnfaip8	TPC_Ageddown	TPC
3.53E-13	-0.572259	0.075	0.348	8.46E-09	Dbp	TPC_Ageddown	TPC
2.92E-21	-0.572826	0.84	0.975	7.00E-17	Sumo2	TPC_Ageddown	TPC
3.24E-15	-0.578546	0.53	0.789	7.76E-11	Cct5	TPC_Ageddown	TPC
9.60E-16	-0.581572	0.945	0.978	2.30E-11	Gsta4	TPC_Ageddown	TPC
8.74E-11	-0.581906	0.31	0.618	2.10E-06	Krt75	TPC_Ageddown	TPC
2.77E-20	-0.59535	0.82	0.96	6.63E-16	Slc25a5	TPC_Ageddown	TPC
1.19E-13	-0.599399	0	0.229	2.85E-09	Gm8882	TPC_Ageddown	TPC
2.65E-18	-0.603339	0.81	0.953	6.37E-14	Ybx1	TPC_Ageddown	TPC
1.01E-57	-0.607657	1	1	2.42E-53	Tpt1	TPC_Ageddown	TPC
6.86E-10	-0.614395	0.305	0.542	1.65E-05	Lxn	TPC_Ageddown	TPC
2.37E-17	-0.62185	0.61	0.858	5.68E-13	Hnrnpc	TPC_Ageddown	TPC
2.31E-63	-0.628835	1	0.999	5.55E-59	Rps14	TPC_Ageddown	TPC
1.46E-11	-0.629932	0.085	0.319	3.50E-07	Myc	TPC_Ageddown	TPC
1.52E-15	-0.639669	0.715	0.905	3.64E-11	MuClm	TPC_Ageddown	TPC
7.27E-20	-0.644502	0.645	0.916	1.74E-15	Eif3e	TPC_Ageddown	TPC
1.06E-48	-0.645607	1	0.997	2.54E-44	Rack1	TPC_Ageddown	TPC
5.71E-16	-0.647765	0.815	0.97	1.37E-11	Gsto1	TPC_Ageddown	TPC
3.85E-51	-0.650473	0.985	1	9.23E-47	Rpl21	TPC_Ageddown	TPC
1.08E-14	-0.652602	0.785	0.919	2.59E-10	Ptn	TPC_Ageddown	TPC
1.53E-74	-0.653761	1	1	3.66E-70	Rps8	TPC_Ageddown	TPC
2.83E-17	-0.656297	0.43	0.749	6.78E-13	Fkbp4	TPC_Ageddown	TPC
6.60E-19	-0.656393	0.39	0.716	1.58E-14	Cacybp	TPC_Ageddown	TPC
9.90E-09	-0.657536	0.35	0.579	0.000237	Nfkbia	TPC_Ageddown	TPC
8.83E-11	-0.658831	0.385	0.692	2.12E-06	Jun	TPC_Ageddown	TPC
1.26E-11	-0.659839	0.27	0.545	3.01E-07	Gadd45b	TPC_Ageddown	TPC
4.71E-18	-0.661694	0.48	0.785	1.13E-13	Sumo1	TPC_Ageddown	TPC
1.50E-37	-0.663729	0.985	0.999	3.61E-33	Rps25	TPC_Ageddown	TPC
3.62E-11	-0.694061	0.31	0.57	8.68E-07	2310057J18Rik	TPC_Ageddown	TPC
2.42E-26	-0.697601	0.81	0.966	5.81E-22	Eif4a1	TPC_Ageddown	TPC
3.63E-17	-0.718717	0.195	0.538	8.71E-13	Hspa1a	TPC_Ageddown	TPC
5.84E-83	-0.725065	1	1	1.40E-78	mt-Cytb	TPC_Ageddown	TPC
5.14E-26	-0.727148	0.93	0.999	1.23E-21	Hspb1	TPC_Ageddown	TPC
6.22E-19	-0.728052	0.5	0.752	1.49E-14	Manf	TPC_Ageddown	TPC
5.13E-21	-0.73633	0.595	0.855	1.23E-16	Hspa1b	TPC_Ageddown	TPC
3.97E-17	-0.737475	0.2	0.535	9.51E-13	Anxa8	TPC_Ageddown	TPC
5.74E-25	-0.749294	0.96	0.997	1.38E-20	Lgals7	TPC_Ageddown	TPC
5.25E-24	-0.754415	0.42	0.781	1.26E-19	U2af1	TPC_Ageddown	TPC
4.59E-22	-0.760633	0.435	0.77	1.10E-17	Psma4	TPC_Ageddown	TPC
2.97E-10	-0.762919	0.59	0.847	7.13E-06	Adh7	TPC_Ageddown	TPC
2.92E-15	-0.771304	0.19	0.494	7.00E-11	Efemp1	TPC_Ageddown	TPC
2.75E-14	-0.783571	0.75	0.897	6.59E-10	Neat1	TPC_Ageddown	TPC
8.13E-66	-0.790937	0.995	1	1.95E-61	mt-Nd2	TPC_Ageddown	TPC
5.14E-53	-0.805423	0.975	0.999	1.23E-48	H3f3a	TPC_Ageddown	TPC
6.41E-23	-0.818795	0.81	0.943	1.54E-18	Ccnd2	TPC_Ageddown	TPC

8.67E-12	-0.840778	0.32	0.608	2.08E-07	Amy1	TPC_Ageddown	TPC
3.52E-39	-0.881878	0.985	0.996	8.45E-35	Rps15	TPC_Ageddown	TPC
1.28E-94	-0.896588	1	1	3.08E-90	mt-Atp6	TPC_Ageddown	TPC
8.94E-89	-0.901815	1	1	2.14E-84	mt-Co3	TPC_Ageddown	TPC
2.03E-25	-0.92252	0.07	0.494	4.86E-21	Ifi202b	TPC_Ageddown	TPC
1.06E-24	-0.931468	0.675	0.935	2.54E-20	Id3	TPC_Ageddown	TPC
3.18E-80	-0.995708	1	1	7.62E-76	Rps12	TPC_Ageddown	TPC
2.54E-72	-1.009437	0.99	1	6.10E-68	Hsp90ab1	TPC_Ageddown	TPC
1.79E-78	-1.026679	0.995	1	4.28E-74	Rpl12	TPC_Ageddown	TPC
1.04E-53	-1.057785	0.92	0.992	2.48E-49	Rpl23a	TPC_Ageddown	TPC
5.33E-15	-1.072585	0.27	0.557	1.28E-10	Hpgd	TPC_Ageddown	TPC
3.45E-37	-1.077219	0.65	0.92	8.28E-33	Dnaja1	TPC_Ageddown	TPC
1.21E-64	-1.078549	0.96	1	2.89E-60	Hspa8	TPC_Ageddown	TPC
1.02E-52	-1.079422	0.935	0.999	2.44E-48	Ubb	TPC_Ageddown	TPC
9.11E-34	-1.089439	0.23	0.698	2.18E-29	Hsph1	TPC_Ageddown	TPC
3.29E-29	-1.183528	0.58	0.91	7.88E-25	Bpifb1	TPC_Ageddown	TPC
7.17E-38	-1.194463	0.99	0.999	1.72E-33	Krt17	TPC_Ageddown	TPC
4.55E-32	-1.33156	0.44	0.825	1.09E-27	Id1	TPC_Ageddown	TPC
2.20E-53	-1.378395	0.07	0.757	5.28E-49	Tmem59	TPC_Ageddown	TPC
1.95E-69	-1.551537	0.77	0.99	4.68E-65	Hsp90aa1	TPC_Ageddown	TPC
1.40E-40	-1.825798	0.685	0.974	3.37E-36	Lipf	TPC_Ageddown	TPC
1.36E-49	-2.05758	0.725	0.99	3.26E-45	Sbpl	TPC_Ageddown	TPC
