# Supplementary material for: Single‐cell transcriptomic atlas of taste papilla aging
Source: Aging Cell. 2024 Aug 21;23(12):e14308. doi: 10.1111/acel.14308 (PMC11634696; doi:10.1111/acel.14308)
Supplement: Supplementary file 5 — Table S4. [file ACEL-23-e14308-s005.pdf]

Table S4. DEGs between the aged and young FLP.

p_val	avg_log2FC	pct.1	pct.2	p_val_adj	gene	condition	cluster
3.36E-141	2.09360126	0.767	0.292	8.06E-137	Ifi27l2a	BC_Agedup	BC
1.79E-109	2.07196494	0.28	0.001	4.30E-105	Xist	BC_Agedup	BC
3.97E-117	2.06857748	0.831	0.532	9.53E-113	Krt4	BC_Agedup	BC
2.61E-209	1.90048805	0.818	0.251	6.25E-205	B2m	BC_Agedup	BC
3.84E-201	1.8458737	0.85	0.345	9.21E-197	H2-K1	BC_Agedup	BC
4.58E-114	1.76234246	0.478	0.101	1.10E-109	1600014C10Rik	BC_Agedup	BC
4.12E-185	1.66725458	0.992	0.951	9.89E-181	Crip1	BC_Agedup	BC
1.59E-155	1.61307818	0.879	0.549	3.81E-151	H2-D1	BC_Agedup	BC
4.41E-97	1.59984895	0.838	0.589	1.06E-92	Mt4	BC_Agedup	BC
1.90E-105	1.58657332	0.297	0.01	4.56E-101	Gm10260	BC_Agedup	BC
3.61E-159	1.49177446	0.941	0.717	8.65E-155	Ifitm3	BC_Agedup	BC
2.13E-103	1.47460624	0.535	0.145	5.10E-99	H19	BC_Agedup	BC
2.47E-110	1.3705915	1	0.999	5.93E-106	Gm42418	BC_Agedup	BC
1.90E-152	1.23498532	0.976	0.9	4.56E-148	mt-Atp8	BC_Agedup	BC
1.96E-115	1.20732275	0.994	0.975	4.70E-111	Mt1	BC_Agedup	BC
4.64E-113	1.18354592	0.976	0.889	1.11E-108	Mt2	BC_Agedup	BC
9.48E-12	1.1276975	0.176	0.088	2.27E-07	Hbb-bs	BC_Agedup	BC
9.22E-82	1.11975368	0.769	0.516	2.21E-77	Tppp3	BC_Agedup	BC
4.11E-105	1.07089985	0.949	0.882	9.86E-101	Dbi	BC_Agedup	BC
1.38E-102	1.05480578	0.896	0.722	3.31E-98	Ly6a	BC_Agedup	BC
1.14E-65	0.9615554	0.54	0.229	2.74E-61	Calml3	BC_Agedup	BC
6.92E-206	0.93869256	0.999	0.999	1.66E-201	mt-Nd4l	BC_Agedup	BC
2.07E-66	0.91967094	0.618	0.339	4.97E-62	Ly6e	BC_Agedup	BC
2.47E-82	0.90878278	0.692	0.384	5.91E-78	Csrp1	BC_Agedup	BC
9.45E-122	0.87193397	0.969	0.949	2.27E-117	Ndufa4	BC_Agedup	BC
3.59E-211	0.84866297	0.999	1	8.61E-207	Gm10076	BC_Agedup	BC
4.37E-22	0.84279307	0.957	0.93	1.05E-17	Fabp5	BC_Agedup	BC
1.26E-74	0.83512988	0.903	0.837	3.02E-70	Cstb	BC_Agedup	BC
4.15E-60	0.7715589	0.711	0.485	9.95E-56	Psme2	BC_Agedup	BC
6.87E-40	0.74628082	0.561	0.321	1.65E-35	Fam25c	BC_Agedup	BC
7.45E-61	0.74152704	0.913	0.836	1.79E-56	Cst3	BC_Agedup	BC
1.12E-62	0.71365535	0.216	0.018	2.69E-58	Muc5b	BC_Agedup	BC
1.73E-80	0.69951991	0.289	0.03	4.15E-76	Psmb8	BC_Agedup	BC
7.62E-50	0.68550369	0.802	0.67	1.83E-45	Tmsb10	BC_Agedup	BC
1.67E-43	0.66127492	0.997	0.999	4.02E-39	Actb	BC_Agedup	BC
2.92E-16	0.64714813	0.396	0.263	6.99E-12	Cyp2f2	BC_Agedup	BC
1.34E-44	0.6456671	0.528	0.3	3.20E-40	Gstp1	BC_Agedup	BC
1.07E-54	0.64474058	0.327	0.087	2.57E-50	Dynap	BC_Agedup	BC
1.72E-70	0.63432531	0.253	0.025	4.12E-66	Psmb9	BC_Agedup	BC
7.40E-43	0.61416806	0.515	0.276	1.77E-38	Snhg20	BC_Agedup	BC
9.73E-65	0.60741884	0.991	0.988	2.33E-60	Txn1	BC_Agedup	BC
2.66E-54	0.60204546	0.879	0.787	6.37E-50	Prelid1	BC_Agedup	BC
2.00E-52	0.60189955	0.863	0.798	4.80E-48	Ndufb8	BC_Agedup	BC
2.94E-40	0.59468553	0.675	0.543	7.06E-36	Erh	BC_Agedup	BC
6.37E-67	0.58575488	0.187	0.004	1.53E-62	Tff2	BC_Agedup	BC
4.32E-25	0.58187949	0.175	0.049	1.04E-20	Cenpa	BC_Agedup	BC
3.02E-39	0.57689814	0.326	0.118	7.25E-35	Ly6c1	BC_Agedup	BC
2.61E-42	0.55863745	0.849	0.78	6.26E-38	Snrpg	BC_Agedup	BC
3.15E-31	0.55821854	0.446	0.233	7.56E-27	Igfbp4	BC_Agedup	BC
1.53E-22	0.55719644	0.91	0.924	3.68E-18	H2afz	BC_Agedup	BC
8.78E-26	0.54835197	0.66	0.555	2.11E-21	Selenoh	BC_Agedup	BC
1.00E-20	0.54728015	0.276	0.133	2.41E-16	2610528A11Rik	BC_Agedup	BC
4.04E-22	0.53940558	0.597	0.468	9.68E-18	Tmem176b	BC_Agedup	BC
1.36E-25	0.53799067	0.508	0.32	3.25E-21	Chit1	BC_Agedup	BC
7.22E-61	0.53076852	0.971	0.969	1.73E-56	Tspo	BC_Agedup	BC
1.40E-32	0.52352589	0.918	0.891	3.35E-28	Crip2	BC_Agedup	BC
3.11E-17	0.514468	0.927	0.932	7.47E-13	Sfn	BC_Agedup	BC

1.33E-44	0.51263743	0.895	0.838	3.19E-40	Atp5k	BC_Agedup	BC
7.20E-26	0.50991204	0.642	0.513	1.73E-21	Tmbim6	BC_Agedup	BC
3.38E-38	0.50798934	0.826	0.75	8.10E-34	Ndufc2	BC_Agedup	BC
2.86E-45	0.50736328	0.928	0.893	6.85E-41	Atp5g3	BC_Agedup	BC
2.83E-43	0.50260728	0.335	0.114	6.80E-39	Gm10036	BC_Agedup	BC
7.08E-27	0.50072258	0.743	0.656	1.70E-22	Cavin1	BC_Agedup	BC
8.71E-23	0.50041108	0.442	0.281	2.09E-18	Mgst1	BC_Agedup	BC
7.29E-50	0.49592894	0.96	0.962	1.75E-45	Cox5a	BC_Agedup	BC
8.28E-24	0.49476646	0.756	0.671	1.99E-19	Igfbp7	BC_Agedup	BC
6.31E-27	0.49106484	0.857	0.82	1.51E-22	Tagln2	BC_Agedup	BC
1.15E-22	0.48078129	0.716	0.646	2.77E-18	Ifi27	BC_Agedup	BC
1.47E-38	0.48044109	0.293	0.098	3.52E-34	Cpxm2	BC_Agedup	BC
9.73E-36	0.47949676	0.858	0.796	2.33E-31	Ndufa11	BC_Agedup	BC
1.90E-61	0.47489323	0.981	0.984	4.56E-57	Cox6b1	BC_Agedup	BC
1.60E-22	0.47466062	0.615	0.493	3.85E-18	Ddx3x	BC_Agedup	BC
3.87E-19	0.47243788	0.674	0.627	9.29E-15	Cbx3	BC_Agedup	BC
1.53E-22	0.47243137	0.587	0.439	3.68E-18	Gm47283	BC_Agedup	BC
1.22E-33	0.47178182	0.967	0.958	2.93E-29	Lmna	BC_Agedup	BC
2.55E-44	0.46484369	0.266	0.066	6.11E-40	Arhgdib	BC_Agedup	BC
4.59E-58	0.46443991	0.978	0.985	1.10E-53	Cox5b	BC_Agedup	BC
1.01E-26	0.46254594	0.286	0.123	2.43E-22	Cald1	BC_Agedup	BC
1.14E-08	0.45886088	0.153	0.081	0.000273	Ube2c	BC_Agedup	BC
1.41E-20	0.45858119	0.65	0.539	3.38E-16	Ociad2	BC_Agedup	BC
4.02E-23	0.45249243	0.391	0.228	9.64E-19	Tmem176a	BC_Agedup	BC
4.00E-18	0.44986987	0.277	0.144	9.60E-14	Pttg1	BC_Agedup	BC
6.56E-55	0.44744303	0.99	0.994	1.57E-50	Pfn1	BC_Agedup	BC
7.13E-16	0.44658689	0.739	0.618	1.71E-11	Wfdc2	BC_Agedup	BC
1.25E-53	0.44090361	0.191	0.017	3.00E-49	Lgals9	BC_Agedup	BC
9.70E-18	0.44026744	0.946	0.934	2.33E-13	Igfbp2	BC_Agedup	BC
2.81E-25	0.43844018	0.692	0.569	6.75E-21	Ndufv3	BC_Agedup	BC
6.40E-21	0.43662758	0.696	0.592	1.53E-16	Ddb1	BC_Agedup	BC
1.20E-20	0.43100477	0.6	0.492	2.88E-16	Cenpx	BC_Agedup	BC
3.74E-25	0.42787883	0.77	0.679	8.96E-21	Cyc1	BC_Agedup	BC
9.50E-130	0.42733896	1	1	2.28E-125	mt-Co1	BC_Agedup	BC
6.33E-19	0.42625137	0.564	0.445	1.52E-14	Psme1	BC_Agedup	BC
4.07E-26	0.42231135	0.152	0.035	9.75E-22	Birc5	BC_Agedup	BC
6.02E-42	0.4222257	0.972	0.977	1.44E-37	Uqcr11	BC_Agedup	BC
2.44E-22	0.42172933	0.738	0.664	5.85E-18	Nenf	BC_Agedup	BC
1.38E-41	0.41981905	0.985	0.993	3.32E-37	Ftl1	BC_Agedup	BC
1.40E-22	0.41920034	0.756	0.69	3.35E-18	Ndufb7	BC_Agedup	BC
2.83E-29	0.41259677	0.272	0.101	6.80E-25	Aknad1	BC_Agedup	BC
3.14E-49	0.41189722	0.983	0.991	7.53E-45	Uba52	BC_Agedup	BC
4.81E-24	0.40754242	0.257	0.107	1.15E-19	Snhg9	BC_Agedup	BC
3.84E-19	0.40388537	0.597	0.483	9.21E-15	1810022K09Rik	BC_Agedup	BC
3.62E-17	0.40279309	0.533	0.418	8.69E-13	Dnajc19	BC_Agedup	BC
4.49E-47	0.40245745	0.987	0.998	1.08E-42	Oaz1	BC_Agedup	BC
1.65E-14	0.40170356	0.645	0.567	3.96E-10	Rsrp1	BC_Agedup	BC
1.84E-19	0.40165577	0.537	0.403	4.42E-15	2-Mar	BC_Agedup	BC
4.39E-21	0.40147515	0.798	0.766	1.05E-16	Capg	BC_Agedup	BC
9.66E-16	0.40089128	0.284	0.155	2.32E-11	Tfcp2l1	BC_Agedup	BC
3.83E-14	0.39653823	0.39	0.272	9.19E-10	Eppk1	BC_Agedup	BC
2.97E-20	0.3950856	0.216	0.088	7.13E-16	Idi1	BC_Agedup	BC
4.13E-17	0.39373359	0.607	0.514	9.90E-13	Csnk2b	BC_Agedup	BC
8.25E-12	0.38851754	0.448	0.352	1.98E-07	Flna	BC_Agedup	BC
1.30E-19	0.38773888	0.834	0.796	3.13E-15	Cox6a1	BC_Agedup	BC
1.76E-17	0.38670099	0.905	0.887	4.21E-13	Gpx2	BC_Agedup	BC
2.99E-19	0.38470859	0.339	0.191	7.18E-15	S100a1	BC_Agedup	BC
1.21E-06	0.38020719	0.141	0.081	0.029072	Slpi	BC_Agedup	BC
6.19E-21	0.37976776	0.817	0.785	1.49E-16	Nop10	BC_Agedup	BC

6.22E-14	0.37715289	0.685	0.632	1.49E-09	Elof1	BC_Agedup	BC
4.52E-34	0.37698076	0.946	0.941	1.08E-29	Uqcr10	BC_Agedup	BC
6.38E-54	0.37449464	0.988	0.998	1.53E-49	Atp5g2	BC_Agedup	BC
2.38E-27	0.37424984	0.944	0.925	5.70E-23	Uqcrq	BC_Agedup	BC
8.27E-26	0.37248977	0.917	0.912	1.98E-21	Reep5	BC_Agedup	BC
5.60E-24	0.37218124	0.91	0.898	1.34E-19	Ldha	BC_Agedup	BC
1.27E-19	0.37213792	0.761	0.693	3.05E-15	Higd2a	BC_Agedup	BC
1.13E-11	0.37010844	0.547	0.468	2.71E-07	Acp5	BC_Agedup	BC
3.91E-13	0.3696338	0.552	0.471	9.38E-09	Got2	BC_Agedup	BC
8.46E-12	0.36951801	0.464	0.363	2.03E-07	Jpt1	BC_Agedup	BC
7.98E-16	0.36583489	0.214	0.1	1.91E-11	Peg3	BC_Agedup	BC
8.21E-09	0.36558798	0.391	0.315	0.000197	Klk10	BC_Agedup	BC
1.99E-16	0.36541581	0.648	0.573	4.78E-12	Ndufb6	BC_Agedup	BC
2.48E-08	0.36502341	0.418	0.342	0.000595	Epcam	BC_Agedup	BC
1.49E-14	0.36496645	0.138	0.051	3.58E-10	Acta1	BC_Agedup	BC
1.56E-13	0.36242697	0.637	0.579	3.74E-09	Cycs	BC_Agedup	BC
3.69E-26	0.36166636	0.905	0.887	8.85E-22	Edf1	BC_Agedup	BC
4.28E-23	0.36139716	0.202	0.07	1.03E-18	Lgals1	BC_Agedup	BC
6.22E-15	0.36059653	0.693	0.635	1.49E-10	Naa38	BC_Agedup	BC
8.17E-21	0.35869088	0.841	0.819	1.96E-16	Mdh2	BC_Agedup	BC
9.04E-11	0.35790288	0.537	0.447	2.17E-06	Car12	BC_Agedup	BC
2.02E-71	0.35788031	1	1	4.85E-67	Ppia	BC_Agedup	BC
3.66E-31	0.35730106	0.962	0.972	8.79E-27	Cox7a2	BC_Agedup	BC
1.65E-14	0.3569979	0.51	0.405	3.95E-10	Lsm3	BC_Agedup	BC
1.50E-11	0.35613672	0.271	0.166	3.60E-07	Tnni2	BC_Agedup	BC
4.86E-16	0.35389047	0.781	0.752	1.16E-11	Spint2	BC_Agedup	BC
1.42E-15	0.35258229	0.746	0.688	3.40E-11	Srsf5	BC_Agedup	BC
3.71E-17	0.3525197	0.809	0.788	8.91E-13	Usmg5	BC_Agedup	BC
2.27E-17	0.35243521	0.749	0.727	5.43E-13	Romo1	BC_Agedup	BC
2.16E-23	0.34987555	0.85	0.83	5.18E-19	Ndufab1	BC_Agedup	BC
6.04E-16	0.34883094	0.683	0.604	1.45E-11	Lsm7	BC_Agedup	BC
8.61E-21	0.3482914	0.905	0.888	2.07E-16	Ifitm2	BC_Agedup	BC
3.54E-10	0.34520544	0.224	0.135	8.49E-06	Fdps	BC_Agedup	BC
3.36E-14	0.34457975	0.554	0.464	8.06E-10	Mrps28	BC_Agedup	BC
1.75E-18	0.34443352	0.303	0.165	4.19E-14	Ggh	BC_Agedup	BC
6.29E-13	0.34283103	0.299	0.186	1.51E-08	Crlf1	BC_Agedup	BC
1.16E-14	0.34259583	0.682	0.59	2.79E-10	Tpm2	BC_Agedup	BC
2.11E-12	0.34202877	0.146	0.062	5.06E-08	Cdc20	BC_Agedup	BC
2.04E-12	0.34186666	0.535	0.395	4.90E-08	Crabp1	BC_Agedup	BC
4.98E-16	0.34047763	0.793	0.758	1.19E-11	Aprt	BC_Agedup	BC
1.27E-12	0.33997034	0.496	0.382	3.05E-08	Ehf	BC_Agedup	BC
7.49E-23	0.33773312	0.941	0.952	1.80E-18	Atp5j	BC_Agedup	BC
1.00E-26	0.33723657	0.953	0.969	2.41E-22	Atp5g1	BC_Agedup	BC
7.29E-18	0.33667585	0.735	0.682	1.75E-13	Spcs1	BC_Agedup	BC
3.00E-16	0.33659408	0.809	0.771	7.19E-12	Lsm4	BC_Agedup	BC
8.45E-14	0.3363995	0.726	0.697	2.03E-09	Ndufa5	BC_Agedup	BC
4.71E-19	0.33635731	0.83	0.805	1.13E-14	Swi5	BC_Agedup	BC
3.88E-23	0.33400393	0.21	0.075	9.31E-19	Alad	BC_Agedup	BC
5.65E-10	0.3338149	0.37	0.287	1.35E-05	Wdr1	BC_Agedup	BC
4.18E-16	0.33375679	0.748	0.705	1.00E-11	Mrpl12	BC_Agedup	BC
2.60E-20	0.33307212	0.908	0.918	6.23E-16	Pebp1	BC_Agedup	BC
3.52E-36	0.33258288	0.988	0.997	8.44E-32	Cox6c	BC_Agedup	BC
3.69E-15	0.33234134	0.123	0.039	8.85E-11	Tnnc2	BC_Agedup	BC
3.80E-11	0.33219798	0.639	0.587	9.11E-07	Fkbp2	BC_Agedup	BC
5.10E-18	0.33084713	0.797	0.769	1.22E-13	Taf10	BC_Agedup	BC
1.64E-11	0.33032573	0.592	0.534	3.93E-07	Ewsr1	BC_Agedup	BC
1.74E-09	0.33013437	0.184	0.102	4.18E-05	Mki67	BC_Agedup	BC
7.41E-07	0.32912096	0.605	0.574	0.017772	Sod1	BC_Agedup	BC
3.27E-29	0.32716176	0.123	0.016	7.83E-25	Isg15	BC_Agedup	BC

1.06E-12	0.32655448	0.747	0.717	2.53E-08	Pdlim1	BC_Agedup	BC
3.57E-11	0.32561121	0.688	0.659	8.57E-07	Nhp2	BC_Agedup	BC
1.80E-12	0.32452479	0.391	0.284	4.32E-08	Taf6l	BC_Agedup	BC
2.12E-17	0.32360099	0.868	0.861	5.08E-13	Sec61b	BC_Agedup	BC
3.57E-13	0.32189421	0.841	0.84	8.57E-09	Calm2	BC_Agedup	BC
2.57E-21	0.32189301	0.193	0.068	6.16E-17	Cenpw	BC_Agedup	BC
4.30E-26	0.32184036	0.981	0.994	1.03E-21	Prdx1	BC_Agedup	BC
5.85E-14	0.3217327	0.708	0.655	1.40E-09	Sdhb	BC_Agedup	BC
7.47E-12	0.32027001	0.639	0.585	1.79E-07	Ndufa3	BC_Agedup	BC
3.55E-14	0.3191084	0.781	0.777	8.52E-10	Ndufa1	BC_Agedup	BC
1.11E-12	0.31883545	0.372	0.258	2.66E-08	Coro1c	BC_Agedup	BC
1.99E-14	0.31841032	0.454	0.328	4.77E-10	Bola3	BC_Agedup	BC
1.60E-15	0.31800598	0.776	0.76	3.84E-11	D8Ertd738e	BC_Agedup	BC
3.44E-23	0.31749131	0.941	0.962	8.24E-19	Ndufb9	BC_Agedup	BC
1.56E-16	0.31672559	0.808	0.783	3.73E-12	Trmt112	BC_Agedup	BC
1.44E-29	0.31631091	0.178	0.042	3.46E-25	Dhcr24	BC_Agedup	BC
1.69E-13	0.31611119	0.825	0.835	4.05E-09	Ndufs6	BC_Agedup	BC
3.39E-09	0.31536884	0.696	0.666	8.14E-05	Canx	BC_Agedup	BC
9.58E-18	0.3147991	0.868	0.87	2.30E-13	Pomp	BC_Agedup	BC
7.58E-11	0.31468658	0.362	0.263	1.82E-06	Dcxr	BC_Agedup	BC
6.47E-13	0.31461115	0.627	0.532	1.55E-08	Churc1	BC_Agedup	BC
2.35E-15	0.31420507	0.762	0.725	5.63E-11	Ndufa12	BC_Agedup	BC
3.43E-14	0.3141845	0.794	0.78	8.22E-10	Ndufs7	BC_Agedup	BC
1.05E-11	0.31389414	0.606	0.542	2.52E-07	Eif4g1	BC_Agedup	BC
2.82E-20	0.31383752	0.98	0.978	6.77E-16	Actg1	BC_Agedup	BC
4.39E-33	0.31306973	0.161	0.028	1.05E-28	Clec11a	BC_Agedup	BC
1.59E-12	0.31282008	0.648	0.592	3.81E-08	1810058I24Rik	BC_Agedup	BC
1.20E-18	0.31275559	0.922	0.947	2.88E-14	Sec61g	BC_Agedup	BC
3.03E-26	0.31232393	0.212	0.069	7.28E-22	Adat2	BC_Agedup	BC
2.55E-15	0.31086698	0.249	0.13	6.12E-11	Fbln1	BC_Agedup	BC
5.05E-09	0.30994397	0.606	0.573	0.000121	Hsbp1	BC_Agedup	BC
1.18E-09	0.30882634	0.808	0.777	2.84E-05	Dsc3	BC_Agedup	BC
2.41E-10	0.30858212	0.215	0.124	5.79E-06	Epas1	BC_Agedup	BC
1.45E-09	0.30735462	0.379	0.287	3.49E-05	Acat1	BC_Agedup	BC
7.29E-10	0.3059538	0.657	0.6	1.75E-05	Hspa9	BC_Agedup	BC
5.20E-08	0.30338048	0.371	0.292	0.001248	Fat2	BC_Agedup	BC
1.14E-14	0.30180668	0.788	0.754	2.73E-10	Arpp19	BC_Agedup	BC
6.74E-20	0.29957428	0.936	0.952	1.62E-15	Elob	BC_Agedup	BC
1.09E-10	0.2987845	0.405	0.31	2.61E-06	Fmc1	BC_Agedup	BC
4.50E-11	0.29769154	0.988	0.951	1.08E-06	Krt13	BC_Agedup	BC
1.34E-11	0.2967189	0.473	0.366	3.20E-07	Pigp	BC_Agedup	BC
2.86E-12	0.29629503	0.753	0.731	6.87E-08	Bola2	BC_Agedup	BC
4.18E-15	0.29598027	0.953	0.94	1.00E-10	Anxa2	BC_Agedup	BC
4.18E-13	0.2957094	0.659	0.564	1.00E-08	Pdcd5	BC_Agedup	BC
5.37E-75	0.29565234	0.997	1	1.29E-70	Rpl11	BC_Agedup	BC
1.13E-18	0.29376604	0.214	0.091	2.70E-14	Nudt22	BC_Agedup	BC
8.95E-11	0.29345722	0.657	0.621	2.15E-06	Hcfc1r1	BC_Agedup	BC
3.48E-11	0.29342631	0.368	0.258	8.35E-07	Tmem205	BC_Agedup	BC
2.79E-09	0.29293063	0.407	0.313	6.68E-05	Akr1b3	BC_Agedup	BC
2.35E-11	0.29227281	0.688	0.634	5.64E-07	Phgdh	BC_Agedup	BC
1.35E-09	0.29210022	0.334	0.245	3.23E-05	9-Sep	BC_Agedup	BC
9.25E-10	0.29025128	0.515	0.428	2.22E-05	BC005537	BC_Agedup	BC
4.36E-15	0.2887337	0.17	0.071	1.05E-10	Ckap2l	BC_Agedup	BC
1.32E-11	0.28734491	0.719	0.693	3.17E-07	Atp6v1g1	BC_Agedup	BC
9.18E-26	0.28696517	0.159	0.038	2.20E-21	Tppp	BC_Agedup	BC
4.37E-69	0.28580068	1	1	1.05E-64	Rps19	BC_Agedup	BC
5.90E-07	0.28520577	0.5	0.442	0.014154	Dctpp1	BC_Agedup	BC
2.03E-11	0.28408079	0.847	0.819	4.88E-07	Jund	BC_Agedup	BC
4.76E-15	0.28379786	0.247	0.125	1.14E-10	Pitx2	BC_Agedup	BC

1.19E-11	0.2831511	0.692	0.646	2.85E-07	Mrpl54	BC_Agedup	BC
5.08E-18	0.28311281	0.171	0.063	1.22E-13	Tap1	BC_Agedup	BC
4.36E-16	0.28269319	0.115	0.033	1.05E-11	Anln	BC_Agedup	BC
1.78E-10	0.28028869	0.386	0.282	4.27E-06	Cirbp	BC_Agedup	BC
1.04E-10	0.2798767	0.628	0.583	2.50E-06	Ube2m	BC_Agedup	BC
2.64E-17	0.27939998	0.88	0.874	6.33E-13	Ndufa7	BC_Agedup	BC
1.94E-70	0.27928736	1	1	4.65E-66	Rps28	BC_Agedup	BC
5.56E-32	0.27907661	0.99	0.999	1.33E-27	Cox8a	BC_Agedup	BC
1.14E-09	0.27819698	0.335	0.24	2.73E-05	Tob1	BC_Agedup	BC
7.20E-11	0.27805706	0.28	0.18	1.73E-06	Cdc34	BC_Agedup	BC
1.91E-22	0.27805357	0.968	0.982	4.58E-18	Selenow	BC_Agedup	BC
2.87E-09	0.27796557	0.422	0.329	6.89E-05	Gpt	BC_Agedup	BC
1.78E-11	0.27796058	0.613	0.55	4.27E-07	Ndufs3	BC_Agedup	BC
2.74E-15	0.27740778	0.128	0.042	6.57E-11	Cxcl14	BC_Agedup	BC
2.98E-09	0.27713859	0.34	0.252	7.16E-05	Asph	BC_Agedup	BC
1.40E-06	0.27685322	0.65	0.655	0.033637	Ptprs	BC_Agedup	BC
4.10E-07	0.27478144	0.476	0.417	0.009842	Arpc1b	BC_Agedup	BC
3.24E-08	0.27393708	0.476	0.413	0.000776	Sap18	BC_Agedup	BC
5.66E-13	0.27359784	0.873	0.856	1.36E-08	Nme1	BC_Agedup	BC
3.05E-13	0.27323131	0.894	0.906	7.32E-09	2010107E04Rik	BC_Agedup	BC
1.28E-08	0.27228693	0.744	0.73	0.000308	Ctsd	BC_Agedup	BC
2.98E-59	0.27052432	0.999	1	7.16E-55	Rps2	BC_Agedup	BC
4.10E-08	0.26992993	0.283	0.197	0.000982	Hs6st1	BC_Agedup	BC
5.22E-13	0.26956689	0.84	0.84	1.25E-08	Ssr4	BC_Agedup	BC
6.67E-11	0.26925041	0.28	0.178	1.60E-06	Tmem43	BC_Agedup	BC
3.67E-10	0.26801248	0.111	0.045	8.81E-06	Ada	BC_Agedup	BC
3.89E-36	0.26787667	0.115	0.006	9.33E-32	H2-Q7	BC_Agedup	BC
3.20E-08	0.26762909	0.671	0.658	0.000767	Mrps21	BC_Agedup	BC
1.11E-09	0.26744687	0.274	0.18	2.65E-05	Cks1b	BC_Agedup	BC
1.01E-07	0.26709732	0.583	0.545	0.002425	Coa3	BC_Agedup	BC
1.40E-09	0.26704151	0.693	0.676	3.36E-05	Ndufs8	BC_Agedup	BC
6.59E-08	0.26694805	0.164	0.094	0.001579	Pinlyp	BC_Agedup	BC
1.93E-18	0.26680908	0.955	0.96	4.64E-14	Uqcrb	BC_Agedup	BC
6.02E-13	0.26634013	0.753	0.716	1.44E-08	Snrpd1	BC_Agedup	BC
2.37E-14	0.26541024	0.224	0.114	5.67E-10	Psmb10	BC_Agedup	BC
8.77E-08	0.2653932	0.591	0.542	0.002103	Hnrnpdl	BC_Agedup	BC
5.44E-08	0.26457061	0.404	0.326	0.001304	Degs1	BC_Agedup	BC
1.36E-15	0.26446245	0.12	0.037	3.26E-11	Bst2	BC_Agedup	BC
3.42E-43	0.26438358	1	1	8.21E-39	Rpl38	BC_Agedup	BC
5.43E-14	0.26285352	0.918	0.925	1.30E-09	Mrpl52	BC_Agedup	BC
3.37E-09	0.26007463	0.629	0.592	8.09E-05	Bcl7c	BC_Agedup	BC
1.13E-13	0.25917736	0.124	0.043	2.71E-09	Pclaf	BC_Agedup	BC
2.16E-07	0.25861631	0.545	0.489	0.005182	Gas6	BC_Agedup	BC
2.42E-07	0.25852288	0.332	0.26	0.005805	Cyb5b	BC_Agedup	BC
3.03E-07	0.25791006	0.38	0.308	0.007261	Msrb1	BC_Agedup	BC
3.86E-09	0.25725119	0.636	0.595	9.25E-05	Mrpl57	BC_Agedup	BC
1.21E-09	0.25718024	0.219	0.131	2.90E-05	Dvl1	BC_Agedup	BC
2.02E-10	0.25625572	0.159	0.079	4.85E-06	Msmo1	BC_Agedup	BC
9.84E-10	0.25536594	0.448	0.358	2.36E-05	Pet100	BC_Agedup	BC
3.33E-08	0.25495035	0.336	0.249	0.000799	Iscu	BC_Agedup	BC
8.76E-45	0.25440061	0.999	1	2.10E-40	Rpl35	BC_Agedup	BC
6.59E-10	0.2542814	0.761	0.759	1.58E-05	Ndufa8	BC_Agedup	BC
1.57E-09	0.25314236	0.375	0.278	3.77E-05	2310039H08Rik	BC_Agedup	BC
2.45E-07	0.2530957	0.127	0.067	0.005881	Top2a	BC_Agedup	BC
8.77E-08	0.2529344	0.434	0.361	0.002104	Sssca1	BC_Agedup	BC
1.00E-07	0.25232673	0.619	0.576	0.002402	Eno1	BC_Agedup	BC
1.58E-06	0.25223952	0.297	0.221	0.037997	Ptgr1	BC_Agedup	BC
3.24E-12	0.252065	0.864	0.906	7.77E-08	Minos1	BC_Agedup	BC
4.55E-11	0.25175332	0.706	0.684	1.09E-06	Cox14	BC_Agedup	BC

1.57E-11	0.25087529	0.835	0.839	3.76E-07	Ndufs5	BC_Agedup	BC
4.73E-08	0.25082053	0.674	0.643	0.001135	Fis1	BC_Agedup	BC
5.63E-08	0.25063347	0.889	0.928	0.00135	H2afj	BC_Agedup	BC
5.55E-16	0.25041669	0.114	0.031	1.33E-11	Ccnb2	BC_Agedup	BC
3.45E-07	0.24907883	0.653	0.634	0.008265	Bzw1	BC_Agedup	BC
8.69E-07	0.24734451	0.276	0.204	0.020845	Rab3d	BC_Agedup	BC
4.60E-09	0.24726133	0.114	0.05	0.00011	Mylpf	BC_Agedup	BC
9.49E-07	0.24698605	0.501	0.452	0.022746	Vps28	BC_Agedup	BC
1.16E-10	0.24614438	0.772	0.775	2.79E-06	Dad1	BC_Agedup	BC
1.94E-09	0.2444588	0.169	0.091	4.66E-05	Asprv1	BC_Agedup	BC
1.89E-07	0.24424474	0.316	0.239	0.004538	Slc39a1	BC_Agedup	BC
3.36E-09	0.24388972	0.558	0.484	8.05E-05	Tmem238	BC_Agedup	BC
4.12E-10	0.24371247	0.668	0.622	9.89E-06	Eloc	BC_Agedup	BC
1.35E-08	0.2434066	0.726	0.721	0.000323	Sub1	BC_Agedup	BC
2.91E-08	0.24330873	0.416	0.331	0.000697	Prpf8	BC_Agedup	BC
5.49E-10	0.24281467	0.808	0.827	1.32E-05	Mrpl23	BC_Agedup	BC
1.30E-07	0.24269615	0.214	0.138	0.003112	Slc16a11	BC_Agedup	BC
8.99E-09	0.24241272	0.235	0.151	0.000216	Ciapin1	BC_Agedup	BC
1.36E-06	0.24210199	0.302	0.228	0.032629	Dnph1	BC_Agedup	BC
1.59E-08	0.24185019	0.724	0.696	0.00038	Slc25a4	BC_Agedup	BC
2.14E-07	0.24097377	0.192	0.118	0.00513	Defb1	BC_Agedup	BC
9.62E-13	0.24096525	0.945	0.956	2.31E-08	Pabpc1	BC_Agedup	BC
1.87E-09	0.24075205	0.749	0.739	4.49E-05	Ndufb2	BC_Agedup	BC
6.38E-07	0.24050706	0.29	0.215	0.015299	Tapbp	BC_Agedup	BC
1.77E-11	0.24020412	0.183	0.094	4.25E-07	H2-T22	BC_Agedup	BC
5.80E-17	0.24008348	0.104	0.025	1.39E-12	Crabp2	BC_Agedup	BC
5.94E-07	0.23956911	0.648	0.638	0.014251	Tecr	BC_Agedup	BC
1.59E-08	0.23926707	0.412	0.324	0.000381	Rab24	BC_Agedup	BC
7.12E-08	0.23889632	0.803	0.827	0.001708	Ndufc1	BC_Agedup	BC
2.83E-62	0.23843881	0.999	1	6.80E-58	Rps29	BC_Agedup	BC
1.45E-10	0.23832125	0.841	0.843	3.48E-06	Smdt1	BC_Agedup	BC
1.51E-12	0.23827284	0.93	0.963	3.63E-08	Snrpe	BC_Agedup	BC
6.33E-07	0.23796198	0.5	0.445	0.015191	Rpn1	BC_Agedup	BC
2.61E-11	0.23770958	0.192	0.099	6.25E-07	Hebp2	BC_Agedup	BC
4.08E-15	0.2377086	0.137	0.048	9.78E-11	Cyp51	BC_Agedup	BC
7.31E-16	0.23682686	0.978	0.993	1.75E-11	Hint1	BC_Agedup	BC
7.12E-07	0.23676316	0.641	0.638	0.01708	Krtcap2	BC_Agedup	BC
1.83E-08	0.23553297	0.698	0.691	0.000439	Rex1bd	BC_Agedup	BC
6.36E-10	0.23495609	0.893	0.932	1.53E-05	Ran	BC_Agedup	BC
8.90E-09	0.23423595	0.265	0.174	0.000213	Uxt	BC_Agedup	BC
1.16E-07	0.23364298	0.24	0.165	0.002793	Carhsp1	BC_Agedup	BC
2.59E-10	0.23337905	0.102	0.038	6.22E-06	Ckm	BC_Agedup	BC
1.26E-08	0.23264976	0.801	0.827	0.000302	Psmb6	BC_Agedup	BC
4.21E-07	0.23259061	0.387	0.316	0.010096	Ubl4a	BC_Agedup	BC
2.52E-11	0.23186318	0.142	0.06	6.04E-07	Mt3	BC_Agedup	BC
1.47E-08	0.23046312	0.774	0.787	0.000353	Mrps14	BC_Agedup	BC
5.59E-07	0.23010944	0.723	0.721	0.013396	Mdh1	BC_Agedup	BC
8.97E-09	0.22992835	0.694	0.675	0.000215	Ndufb3	BC_Agedup	BC
4.64E-16	0.22767469	0.977	0.988	1.11E-11	Sem1	BC_Agedup	BC
8.39E-07	0.22737349	0.283	0.205	0.020124	Slc2a1	BC_Agedup	BC
6.86E-12	0.22672637	0.184	0.091	1.64E-07	Gda	BC_Agedup	BC
1.47E-25	0.22646772	0.107	0.014	3.51E-21	Gm11361	BC_Agedup	BC
3.60E-16	0.22632393	0.153	0.056	8.63E-12	Tap2	BC_Agedup	BC
1.63E-12	0.22618924	0.93	0.939	3.91E-08	Pgls	BC_Agedup	BC
6.59E-13	0.22442516	0.247	0.136	1.58E-08	Bcap29	BC_Agedup	BC
2.15E-07	0.22399747	0.267	0.19	0.005159	Vars	BC_Agedup	BC
2.91E-12	0.22334144	0.964	0.969	6.98E-08	Rbm3	BC_Agedup	BC
2.92E-08	0.22131098	0.785	0.801	0.0007	Gpx4	BC_Agedup	BC
1.16E-43	0.22114385	1	1	2.77E-39	Rpl37a	BC_Agedup	BC

1.07E-06	0.2211418	0.272	0.199	0.025624	Tnfaip1	BC_Agedup	BC
2.67E-51	0.22108892	1	1	6.41E-47	Rpl18a	BC_Agedup	BC
1.25E-08	0.21981602	0.747	0.727	0.000299	Mrpl33	BC_Agedup	BC
7.13E-07	0.21615612	0.313	0.231	0.017094	Sparc	BC_Agedup	BC
2.17E-10	0.21613783	0.132	0.058	5.20E-06	Ldlr	BC_Agedup	BC
1.74E-06	0.21586306	0.625	0.616	0.04161	Cuta	BC_Agedup	BC
2.45E-10	0.21521735	0.896	0.912	5.87E-06	Timm13	BC_Agedup	BC
1.95E-11	0.21405132	0.157	0.073	4.67E-07	Tspan17	BC_Agedup	BC
3.56E-07	0.21385219	0.76	0.787	0.008541	Ost4	BC_Agedup	BC
1.57E-06	0.2128966	0.277	0.206	0.037577	Mapkapk2	BC_Agedup	BC
3.06E-07	0.21192942	0.416	0.34	0.007337	Sin3b	BC_Agedup	BC
4.93E-09	0.21186266	0.198	0.115	0.000118	Bak1	BC_Agedup	BC
2.21E-10	0.21106876	0.926	0.936	5.31E-06	Ubl5	BC_Agedup	BC
6.15E-16	0.20946868	0.996	0.999	1.47E-11	Tmsb4x	BC_Agedup	BC
9.75E-13	0.20871716	0.145	0.06	2.34E-08	Akr1b8	BC_Agedup	BC
1.45E-07	0.20862444	0.85	0.897	0.003474	2410015M20Rik	BC_Agedup	BC
3.54E-09	0.20839677	0.852	0.88	8.49E-05	Ndufa2	BC_Agedup	BC
4.53E-07	0.20707354	0.279	0.202	0.010863	Lrrc59	BC_Agedup	BC
1.81E-08	0.2055835	0.905	0.923	0.000434	Nedd8	BC_Agedup	BC
8.30E-12	0.20439208	0.137	0.057	1.99E-07	Gale	BC_Agedup	BC
4.59E-07	0.20380346	0.244	0.169	0.011005	Dynlt1f	BC_Agedup	BC
1.12E-07	0.20213987	0.916	0.919	0.002682	Pkm	BC_Agedup	BC
1.81E-13	0.20029806	0.988	0.998	4.35E-09	Mif	BC_Agedup	BC
3.31E-13	-0.2001644	0.189	0.348	7.95E-09	Kansl1	BC_Ageddown	BC
3.22E-09	-0.2001877	0.32	0.466	7.72E-05	Smim10l1	BC_Ageddown	BC
4.44E-09	-0.2005718	0.371	0.518	0.000106	Cbx1	BC_Ageddown	BC
7.39E-12	-0.2013138	0.22	0.37	1.77E-07	Adk	BC_Ageddown	BC
1.68E-09	-0.2017727	0.783	0.875	4.03E-05	Nfix	BC_Ageddown	BC
8.38E-10	-0.2020389	0.261	0.401	2.01E-05	Utp14a	BC_Ageddown	BC
1.56E-11	-0.2023238	0.162	0.296	3.75E-07	Cops5	BC_Ageddown	BC
2.68E-09	-0.2031843	0.251	0.393	6.43E-05	Ppic	BC_Ageddown	BC
1.09E-10	-0.2036958	0.237	0.386	2.61E-06	Siah1a	BC_Ageddown	BC
7.98E-10	-0.2044478	0.413	0.571	1.91E-05	Ddx46	BC_Ageddown	BC
9.51E-11	-0.2050664	0.141	0.263	2.28E-06	Meis2	BC_Ageddown	BC
5.62E-13	-0.2052508	0.216	0.382	1.35E-08	Zfhx3	BC_Ageddown	BC
4.63E-08	-0.2056004	0.416	0.561	0.001111	Gadd45gip1	BC_Ageddown	BC
3.49E-09	-0.2057654	0.474	0.632	8.37E-05	Tra2a	BC_Ageddown	BC
4.14E-09	-0.205829	0.532	0.714	9.93E-05	Stmn1	BC_Ageddown	BC
3.71E-13	-0.2059237	0.096	0.218	8.90E-09	Rab7b	BC_Ageddown	BC
1.45E-10	-0.2064939	0.485	0.645	3.49E-06	Rbm8a	BC_Ageddown	BC
3.67E-11	-0.207194	0.173	0.306	8.80E-07	Snhg6	BC_Ageddown	BC
9.14E-12	-0.2073932	0.151	0.287	2.19E-07	Ino80b	BC_Ageddown	BC
1.13E-08	-0.2074676	0.632	0.782	0.000271	Paip2	BC_Ageddown	BC
2.55E-11	-0.2076572	0.084	0.192	6.13E-07	Cited2	BC_Ageddown	BC
1.32E-08	-0.2086901	0.61	0.739	0.000315	Atrx	BC_Ageddown	BC
2.41E-38	-0.2089426	0.999	1	5.77E-34	Rpl24	BC_Ageddown	BC
5.56E-11	-0.2091376	0.315	0.476	1.33E-06	Arid4b	BC_Ageddown	BC
8.06E-10	-0.2097244	0.22	0.35	1.93E-05	Fam114a2	BC_Ageddown	BC
4.91E-07	-0.2104892	0.495	0.618	0.011764	Wnt10a	BC_Ageddown	BC
7.86E-12	-0.211298	0.201	0.349	1.89E-07	Tbc1d10a	BC_Ageddown	BC
5.41E-12	-0.2114961	0.182	0.326	1.30E-07	Casz1	BC_Ageddown	BC
3.66E-11	-0.21228	0.799	0.896	8.79E-07	Anxa5	BC_Ageddown	BC
2.39E-11	-0.2124286	0.243	0.397	5.72E-07	Mphosph10	BC_Ageddown	BC
2.46E-11	-0.2145081	0.233	0.386	5.90E-07	Ing4	BC_Ageddown	BC
3.40E-18	-0.2150593	0.031	0.151	8.15E-14	Zcchc11	BC_Ageddown	BC
6.79E-12	-0.2160222	0.831	0.93	1.63E-07	Clta	BC_Ageddown	BC
1.99E-56	-0.2162207	1	1	4.77E-52	Rpl23	BC_Ageddown	BC
1.67E-10	-0.2165852	0.265	0.417	4.00E-06	Tmem165	BC_Ageddown	BC
1.64E-10	-0.2167542	0.513	0.678	3.93E-06	Srrm1	BC_Ageddown	BC

5.71E-10	-0.2185313	0.243	0.384	1.37E-05	Gpc3	BC_Ageddown	BC
7.08E-15	-0.2186585	0.069	0.192	1.70E-10	Foxa1	BC_Ageddown	BC
2.52E-13	-0.2189841	0.137	0.279	6.05E-09	Aff3	BC_Ageddown	BC
2.38E-12	-0.2192742	0.206	0.361	5.72E-08	Tcf7l2	BC_Ageddown	BC
1.72E-08	-0.2198376	0.719	0.829	0.000412	Tax1bp1	BC_Ageddown	BC
7.33E-12	-0.2201749	0.266	0.426	1.76E-07	Atp6v1a	BC_Ageddown	BC
5.43E-12	-0.2213191	0.242	0.4	1.30E-07	Usf2	BC_Ageddown	BC
1.09E-10	-0.2233754	0.564	0.728	2.61E-06	Snrnp70	BC_Ageddown	BC
4.23E-12	-0.2234107	0.308	0.473	1.01E-07	Bnip2	BC_Ageddown	BC
1.73E-10	-0.2240032	0.459	0.621	4.14E-06	Acin1	BC_Ageddown	BC
1.54E-13	-0.224839	0.127	0.267	3.70E-09	Tjap1	BC_Ageddown	BC
1.49E-10	-0.2257135	0.331	0.486	3.58E-06	Slc7a1	BC_Ageddown	BC
2.43E-09	-0.2259046	0.618	0.753	5.84E-05	App	BC_Ageddown	BC
3.23E-09	-0.2268817	0.375	0.521	7.74E-05	Gnai1	BC_Ageddown	BC
2.11E-10	-0.2274688	0.561	0.713	5.06E-06	Dusp11	BC_Ageddown	BC
3.27E-11	-0.229045	0.481	0.639	7.83E-07	Mat2a	BC_Ageddown	BC
1.00E-53	-0.2291812	1	1	2.41E-49	Rps9	BC_Ageddown	BC
1.79E-16	-0.229476	0.047	0.168	4.30E-12	Tchh	BC_Ageddown	BC
6.11E-12	-0.2299564	0.336	0.5	1.46E-07	Gtpbp4	BC_Ageddown	BC
2.99E-11	-0.2304429	0.648	0.801	7.18E-07	Lamp2	BC_Ageddown	BC
1.11E-12	-0.2310724	0.201	0.355	2.67E-08	Cdc42ep3	BC_Ageddown	BC
8.55E-11	-0.2313358	0.391	0.556	2.05E-06	Srp72	BC_Ageddown	BC
9.91E-14	-0.2314134	0.872	0.943	2.38E-09	Snrpf	BC_Ageddown	BC
4.73E-12	-0.2320248	0.431	0.612	1.13E-07	Psmc6	BC_Ageddown	BC
2.37E-10	-0.232117	0.506	0.675	5.68E-06	Gnai2	BC_Ageddown	BC
6.99E-12	-0.2329097	0.234	0.388	1.68E-07	Zc3h13	BC_Ageddown	BC
3.24E-15	-0.2332919	0.146	0.305	7.76E-11	Il6ra	BC_Ageddown	BC
2.37E-13	-0.2335831	0.201	0.365	5.67E-09	Ablim1	BC_Ageddown	BC
4.87E-14	-0.2339258	0.257	0.44	1.17E-09	Rsl24d1	BC_Ageddown	BC
5.78E-15	-0.2345424	0.173	0.338	1.39E-10	Ddx3y	BC_Ageddown	BC
3.10E-14	-0.2348968	0.174	0.329	7.44E-10	Ammecr1	BC_Ageddown	BC
5.00E-14	-0.2358954	0.29	0.474	1.20E-09	Cebpz	BC_Ageddown	BC
2.80E-23	-0.2361404	0.017	0.153	6.73E-19	Ogfrl1	BC_Ageddown	BC
4.11E-13	-0.2361579	0.354	0.537	9.86E-09	Chmp5	BC_Ageddown	BC
8.94E-15	-0.236456	0.15	0.304	2.14E-10	Hk2	BC_Ageddown	BC
2.16E-17	-0.2366614	0.912	0.977	5.17E-13	Eif4a1	BC_Ageddown	BC
1.63E-15	-0.2369376	0.157	0.318	3.91E-11	Cir1	BC_Ageddown	BC
1.10E-11	-0.2372663	0.348	0.517	2.64E-07	N4bp2l2	BC_Ageddown	BC
1.45E-09	-0.2380428	0.45	0.598	3.48E-05	Klf3	BC_Ageddown	BC
1.24E-13	-0.2383851	0.145	0.287	2.96E-09	Zfp397	BC_Ageddown	BC
9.33E-12	-0.2384681	0.368	0.525	2.24E-07	Zmat2	BC_Ageddown	BC
7.30E-09	-0.2394285	0.308	0.439	0.000175	Hlf	BC_Ageddown	BC
3.26E-14	-0.239441	0.174	0.333	7.82E-10	Cox19	BC_Ageddown	BC
2.84E-13	-0.2397806	0.265	0.437	6.82E-09	Cisd2	BC_Ageddown	BC
9.80E-14	-0.2397942	0.121	0.257	2.35E-09	Rbms3	BC_Ageddown	BC
7.49E-18	-0.2402213	0.974	0.996	1.80E-13	Itm2b	BC_Ageddown	BC
6.78E-11	-0.2409082	0.307	0.456	1.63E-06	Knop1	BC_Ageddown	BC
1.71E-11	-0.2412214	0.412	0.58	4.10E-07	Nudc	BC_Ageddown	BC
9.26E-14	-0.2416357	0.335	0.527	2.22E-09	Smap1	BC_Ageddown	BC
5.84E-13	-0.2424341	0.592	0.767	1.40E-08	Psma4	BC_Ageddown	BC
1.01E-10	-0.2433369	0.274	0.429	2.43E-06	Rsbn1l	BC_Ageddown	BC
2.34E-18	-0.2436356	0.093	0.251	5.61E-14	Ahsa2	BC_Ageddown	BC
3.10E-13	-0.2442725	0.234	0.397	7.44E-09	Cdc5l	BC_Ageddown	BC
6.52E-13	-0.2443676	0.194	0.349	1.56E-08	Pnp	BC_Ageddown	BC
1.15E-11	-0.244471	0.623	0.754	2.75E-07	Rbm25	BC_Ageddown	BC
3.76E-13	-0.2451018	0.858	0.948	9.01E-09	Trp63	BC_Ageddown	BC
1.67E-19	-0.2457119	0.877	0.971	4.02E-15	Dmkn	BC_Ageddown	BC
5.22E-16	-0.2457146	0.174	0.347	1.25E-11	Lamtor3	BC_Ageddown	BC
5.06E-12	-0.245785	0.309	0.474	1.21E-07	Tprgl	BC_Ageddown	BC

3.88E-19	-0.2465418	0.052	0.192	9.30E-15	Slit3	BC_Ageddown	BC
1.72E-15	-0.2472235	0.203	0.378	4.12E-11	Stim1	BC_Ageddown	BC
2.38E-12	-0.2474367	0.451	0.639	5.70E-08	Paics	BC_Ageddown	BC
2.23E-12	-0.2476652	0.37	0.54	5.36E-08	Fjx1	BC_Ageddown	BC
9.65E-13	-0.2480876	0.399	0.581	2.31E-08	Dync1i2	BC_Ageddown	BC
1.72E-17	-0.2494459	0.091	0.241	4.12E-13	Gt(ROSA)26Sor	BC_Ageddown	BC
9.94E-12	-0.2501576	0.315	0.479	2.38E-07	Emb	BC_Ageddown	BC
3.20E-15	-0.2506657	0.217	0.395	7.67E-11	Arap2	BC_Ageddown	BC
8.28E-12	-0.2512349	0.556	0.71	1.99E-07	HmMuCs1	BC_Ageddown	BC
3.49E-15	-0.2513308	0.113	0.258	8.36E-11	Cyp2s1	BC_Ageddown	BC
6.63E-14	-0.2514069	0.217	0.382	1.59E-09	Baz1a	BC_Ageddown	BC
4.02E-13	-0.2549871	0.412	0.586	9.64E-09	Arhgef12	BC_Ageddown	BC
2.28E-19	-0.2571923	0.049	0.186	5.47E-15	Robo2	BC_Ageddown	BC
5.08E-14	-0.2579568	0.192	0.345	1.22E-09	Wdr12	BC_Ageddown	BC
1.83E-17	-0.2584007	0.129	0.298	4.39E-13	Desi2	BC_Ageddown	BC
4.82E-15	-0.2591637	0.372	0.568	1.16E-10	Senp6	BC_Ageddown	BC
8.57E-14	-0.2592045	0.809	0.897	2.05E-09	Hnrnpa1	BC_Ageddown	BC
1.47E-09	-0.2595972	0.298	0.433	3.53E-05	Myc	BC_Ageddown	BC
4.76E-10	-0.2598997	0.542	0.69	1.14E-05	Cbr3	BC_Ageddown	BC
4.35E-13	-0.2602895	0.148	0.289	1.04E-08	Tgif1	BC_Ageddown	BC
1.15E-14	-0.2603026	0.192	0.362	2.76E-10	Sox21	BC_Ageddown	BC
2.04E-14	-0.2604905	0.514	0.699	4.89E-10	Ogt	BC_Ageddown	BC
2.32E-29	-0.2612917	0.987	0.999	5.56E-25	Cox4i1	BC_Ageddown	BC
6.09E-13	-0.2618668	0.802	0.912	1.46E-08	Serpinb5	BC_Ageddown	BC
5.24E-14	-0.2623558	0.723	0.864	1.26E-09	Itga6	BC_Ageddown	BC
1.83E-11	-0.2631958	0.139	0.26	4.40E-07	Tshz2	BC_Ageddown	BC
5.05E-12	-0.2636836	0.519	0.677	1.21E-07	Bcl11b	BC_Ageddown	BC
2.81E-16	-0.2640347	0.077	0.212	6.74E-12	Sema3c	BC_Ageddown	BC
4.44E-15	-0.2642231	0.198	0.369	1.06E-10	Crim1	BC_Ageddown	BC
1.64E-16	-0.2646988	0.884	0.969	3.93E-12	Slc25a5	BC_Ageddown	BC
3.39E-11	-0.2651316	0.322	0.487	8.13E-07	Htra1	BC_Ageddown	BC
2.41E-26	-0.2651357	0.992	0.999	5.78E-22	Rps17	BC_Ageddown	BC
2.28E-15	-0.2652722	0.583	0.777	5.47E-11	Metap2	BC_Ageddown	BC
8.52E-14	-0.2665836	0.306	0.484	2.04E-09	Epb41l4aos	BC_Ageddown	BC
2.74E-12	-0.2675203	0.41	0.582	6.58E-08	Fkbp4	BC_Ageddown	BC
1.18E-15	-0.2676474	0.121	0.271	2.84E-11	Fzd1	BC_Ageddown	BC
1.02E-14	-0.2681103	0.332	0.524	2.45E-10	Ajuba	BC_Ageddown	BC
1.60E-22	-0.2681125	0.072	0.244	3.84E-18	Ang	BC_Ageddown	BC
4.10E-14	-0.2687993	0.575	0.744	9.82E-10	Ube2b	BC_Ageddown	BC
6.35E-13	-0.2689224	0.17	0.312	1.52E-08	Zfp503	BC_Ageddown	BC
1.09E-14	-0.269076	0.734	0.862	2.60E-10	Skp1a	BC_Ageddown	BC
4.29E-14	-0.2693872	0.258	0.431	1.03E-09	Sox6	BC_Ageddown	BC
6.50E-19	-0.2707776	0.151	0.334	1.56E-14	Rassf9	BC_Ageddown	BC
3.44E-12	-0.271191	0.425	0.59	8.25E-08	Ift20	BC_Ageddown	BC
8.43E-17	-0.2714013	0.188	0.36	2.02E-12	Mpst	BC_Ageddown	BC
1.71E-16	-0.2718934	0.467	0.671	4.10E-12	Psmd4	BC_Ageddown	BC
2.12E-18	-0.2725024	0.183	0.369	5.08E-14	Eva1c	BC_Ageddown	BC
8.48E-12	-0.2727415	0.578	0.743	2.03E-07	Cebpb	BC_Ageddown	BC
2.71E-15	-0.2736653	0.341	0.541	6.49E-11	Srpk2	BC_Ageddown	BC
1.36E-12	-0.2736746	0.472	0.637	3.27E-08	Pnn	BC_Ageddown	BC
2.45E-12	-0.2737306	0.639	0.793	5.86E-08	Cct5	BC_Ageddown	BC
1.41E-15	-0.2745549	0.459	0.671	3.38E-11	St13	BC_Ageddown	BC
7.64E-24	-0.2747473	0.94	0.99	1.83E-19	Col17a1	BC_Ageddown	BC
1.31E-13	-0.2760859	0.394	0.575	3.14E-09	Nipbl	BC_Ageddown	BC
2.69E-15	-0.2763745	0.336	0.529	6.44E-11	Ubn2	BC_Ageddown	BC
8.60E-09	-0.2767823	0.344	0.474	0.000206	Slc39a6	BC_Ageddown	BC
6.77E-17	-0.277382	0.633	0.816	1.62E-12	Cct2	BC_Ageddown	BC
6.18E-17	-0.2777811	0.706	0.87	1.48E-12	Rhoa	BC_Ageddown	BC
4.68E-13	-0.2777878	0.465	0.63	1.12E-08	Fgfr2	BC_Ageddown	BC

3.29E-10	-0.2807703	0.234	0.368	7.89E-06	Hist1h1c	BC_Ageddown	BC
5.63E-16	-0.2821133	0.586	0.759	1.35E-11	Arglu1	BC_Ageddown	BC
1.81E-15	-0.2821703	0.132	0.286	4.35E-11	Ascl2	BC_Ageddown	BC
2.95E-21	-0.2826936	0.957	0.998	7.06E-17	Ubb	BC_Ageddown	BC
6.05E-17	-0.2828412	0.61	0.783	1.45E-12	Pcbp1	BC_Ageddown	BC
2.55E-09	-0.2829749	0.465	0.603	6.12E-05	Atp6v1e1	BC_Ageddown	BC
2.44E-15	-0.2837691	0.256	0.438	5.86E-11	Wac	BC_Ageddown	BC
1.52E-16	-0.2837753	0.253	0.443	3.66E-12	Tsc22d4	BC_Ageddown	BC
2.86E-15	-0.2842936	0.614	0.785	6.86E-11	Tmem234	BC_Ageddown	BC
3.83E-12	-0.2844232	0.44	0.606	9.18E-08	Kmt2e	BC_Ageddown	BC
1.39E-14	-0.2846707	0.633	0.797	3.34E-10	U2af1	BC_Ageddown	BC
4.41E-15	-0.2850772	0.414	0.608	1.06E-10	Anxa7	BC_Ageddown	BC
4.21E-10	-0.2852665	0.249	0.385	1.01E-05	Ces1d	BC_Ageddown	BC
2.91E-17	-0.2853933	0.257	0.451	6.98E-13	Svil	BC_Ageddown	BC
1.59E-14	-0.2855381	0.267	0.443	3.81E-10	Krit1	BC_Ageddown	BC
2.03E-20	-0.2868585	0.114	0.29	4.86E-16	Arhgap10	BC_Ageddown	BC
3.73E-15	-0.28816	0.71	0.82	8.95E-11	Nap1l1	BC_Ageddown	BC
2.21E-25	-0.2904504	0.909	0.981	5.30E-21	mt-Nd3	BC_Ageddown	BC
7.46E-17	-0.2906912	0.632	0.791	1.79E-12	Sf3b1	BC_Ageddown	BC
2.43E-16	-0.2919043	0.51	0.709	5.82E-12	Fubp1	BC_Ageddown	BC
4.19E-14	-0.2938918	0.568	0.732	1.00E-09	Hspa5	BC_Ageddown	BC
5.31E-19	-0.2945364	0.754	0.891	1.27E-14	Rpl13a	BC_Ageddown	BC
8.31E-14	-0.2949798	0.761	0.867	1.99E-09	MuClm	BC_Ageddown	BC
7.95E-69	-0.2958449	1	1	1.91E-64	mt-Nd4	BC_Ageddown	BC
1.02E-14	-0.297907	0.174	0.331	2.44E-10	Csta1	BC_Ageddown	BC
3.87E-18	-0.2980993	0.113	0.273	9.28E-14	Ifi202b	BC_Ageddown	BC
2.55E-15	-0.2982007	0.496	0.684	6.11E-11	Cacybp	BC_Ageddown	BC
4.43E-20	-0.2987649	0.1	0.268	1.06E-15	Hist3h2a	BC_Ageddown	BC
4.70E-14	-0.2995449	0.476	0.658	1.13E-09	Tns4	BC_Ageddown	BC
1.83E-20	-0.3004291	0.105	0.274	4.38E-16	Ddit4	BC_Ageddown	BC
2.51E-13	-0.301033	0.476	0.642	6.02E-09	Sox2	BC_Ageddown	BC
2.14E-16	-0.3018044	0.317	0.511	5.14E-12	Eif2a	BC_Ageddown	BC
2.54E-18	-0.3029359	0.623	0.815	6.09E-14	Eif3m	BC_Ageddown	BC
8.13E-17	-0.3034868	0.244	0.436	1.95E-12	Mafb	BC_Ageddown	BC
2.86E-12	-0.3036687	0.841	0.92	6.86E-08	Apoe	BC_Ageddown	BC
6.54E-18	-0.3036863	0.728	0.874	1.57E-13	Eif2s2	BC_Ageddown	BC
6.56E-23	-0.3048458	0.164	0.373	1.57E-18	Eif2s3y	BC_Ageddown	BC
9.05E-16	-0.304868	0.483	0.669	2.17E-11	Tmpo	BC_Ageddown	BC
2.17E-15	-0.305204	0.315	0.502	5.20E-11	Mxd4	BC_Ageddown	BC
6.61E-19	-0.3055845	0.83	0.931	1.59E-14	Anp32b	BC_Ageddown	BC
2.26E-16	-0.3056329	0.409	0.612	5.41E-12	Atxn7l3b	BC_Ageddown	BC
8.76E-11	-0.3061166	0.166	0.293	2.10E-06	Ier3	BC_Ageddown	BC
2.48E-19	-0.3062386	0.803	0.909	5.94E-15	Nfib	BC_Ageddown	BC
1.19E-13	-0.3095603	0.656	0.811	2.85E-09	Ralbp1	BC_Ageddown	BC
3.67E-55	-0.3096235	1	1	8.80E-51	Rack1	BC_Ageddown	BC
1.64E-20	-0.3111852	0.18	0.381	3.93E-16	Per3	BC_Ageddown	BC
1.28E-58	-0.3118248	1	1	3.07E-54	Lgals7	BC_Ageddown	BC
5.73E-09	-0.3127951	0.487	0.638	0.000138	Ier2	BC_Ageddown	BC
5.92E-18	-0.3134375	0.317	0.519	1.42E-13	Anapc16	BC_Ageddown	BC
6.34E-11	-0.3140567	0.336	0.477	1.52E-06	Arl4a	BC_Ageddown	BC
7.25E-21	-0.3161122	0.226	0.444	1.74E-16	Utp3	BC_Ageddown	BC
2.59E-18	-0.3162116	0.691	0.859	6.21E-14	Srsf2	BC_Ageddown	BC
1.33E-22	-0.3163066	0.113	0.299	3.18E-18	Id4	BC_Ageddown	BC
4.46E-25	-0.3196369	0.193	0.427	1.07E-20	Chordc1	BC_Ageddown	BC
3.06E-16	-0.3212272	0.572	0.758	7.33E-12	Rtn4	BC_Ageddown	BC
9.23E-19	-0.3215368	0.552	0.732	2.21E-14	Pdap1	BC_Ageddown	BC
7.93E-22	-0.3223142	0.224	0.445	1.90E-17	Nr1d2	BC_Ageddown	BC
1.03E-22	-0.3237192	0.192	0.408	2.47E-18	Wdr89	BC_Ageddown	BC
7.46E-15	-0.3243072	0.432	0.603	1.79E-10	Ythdc1	BC_Ageddown	BC

2.72E-20	-0.324859	0.307	0.534	6.53E-16	Hspb8	BC_Ageddown	BC
5.73E-19	-0.3266089	0.246	0.442	1.37E-14	Dnajc21	BC_Ageddown	BC
1.60E-22	-0.328475	0.364	0.607	3.84E-18	Cd109	BC_Ageddown	BC
1.64E-22	-0.3298238	0.173	0.381	3.93E-18	Med13l	BC_Ageddown	BC
1.80E-23	-0.3316175	0.27	0.51	4.32E-19	Tef	BC_Ageddown	BC
1.27E-07	-0.331984	0.638	0.765	0.003038	Lipf	BC_Ageddown	BC
4.82E-98	-0.3320842	1	1	1.16E-93	mt-Cytb	BC_Ageddown	BC
2.94E-19	-0.3328712	0.398	0.609	7.06E-15	Plek2	BC_Ageddown	BC
7.12E-12	-0.3330639	0.348	0.505	1.71E-07	Gm26917	BC_Ageddown	BC
1.01E-18	-0.3331508	0.266	0.459	2.42E-14	Tcf4	BC_Ageddown	BC
4.21E-21	-0.3334918	0.201	0.405	1.01E-16	Cdh13	BC_Ageddown	BC
1.07E-21	-0.3348394	0.151	0.343	2.57E-17	Gpatch4	BC_Ageddown	BC
5.18E-21	-0.3349435	0.739	0.872	1.24E-16	Srrm2	BC_Ageddown	BC
5.88E-21	-0.3382193	0.283	0.498	1.41E-16	Strn3	BC_Ageddown	BC
1.32E-17	-0.3393083	0.32	0.523	3.16E-13	Kmt2a	BC_Ageddown	BC
4.02E-22	-0.3397726	0.078	0.248	9.65E-18	Lef1	BC_Ageddown	BC
3.04E-19	-0.3397991	0.304	0.514	7.28E-15	Carnmt1	BC_Ageddown	BC
8.73E-21	-0.3399874	0.33	0.561	2.09E-16	Cldnd1	BC_Ageddown	BC
8.29E-24	-0.3415092	0.6	0.814	1.99E-19	Aimp1	BC_Ageddown	BC
5.45E-21	-0.3447292	0.362	0.595	1.31E-16	Serpinh1	BC_Ageddown	BC
1.21E-20	-0.3478626	0.708	0.87	2.90E-16	2010111I01Rik	BC_Ageddown	BC
6.36E-19	-0.3484398	0.508	0.708	1.53E-14	Eif5b	BC_Ageddown	BC
9.52E-21	-0.3493019	0.343	0.558	2.28E-16	Snrnp48	BC_Ageddown	BC
5.41E-24	-0.3528997	0.723	0.879	1.30E-19	Hnrnpa0	BC_Ageddown	BC
7.03E-19	-0.3530858	0.338	0.538	1.69E-14	Slc26a2	BC_Ageddown	BC
1.89E-19	-0.357414	0.334	0.547	4.53E-15	Tst	BC_Ageddown	BC
1.75E-25	-0.3604611	0.192	0.419	4.19E-21	Nr1d1	BC_Ageddown	BC
4.57E-57	-0.3609564	0.996	0.999	1.10E-52	Krt5	BC_Ageddown	BC
2.88E-23	-0.3609638	0.491	0.724	6.91E-19	Ncor1	BC_Ageddown	BC
6.00E-20	-0.3621457	0.368	0.587	1.44E-15	Pnisr	BC_Ageddown	BC
1.11E-19	-0.3627175	0.246	0.442	2.66E-15	Plxna2	BC_Ageddown	BC
4.29E-21	-0.3636353	0.361	0.583	1.03E-16	Ssbp2	BC_Ageddown	BC
5.57E-21	-0.3640334	0.455	0.675	1.33E-16	Spop	BC_Ageddown	BC
7.42E-24	-0.3671664	0.276	0.509	1.78E-19	Auts2	BC_Ageddown	BC
3.58E-94	-0.3674368	0.999	1	8.58E-90	Rpl17	BC_Ageddown	BC
2.08E-22	-0.3680903	0.559	0.763	5.00E-18	Srsf11	BC_Ageddown	BC
4.87E-22	-0.3683015	0.798	0.912	1.17E-17	Ccnd2	BC_Ageddown	BC
9.87E-24	-0.3684663	0.634	0.833	2.37E-19	Eif5	BC_Ageddown	BC
6.46E-30	-0.3694796	0.904	0.974	1.55E-25	Gsto1	BC_Ageddown	BC
2.39E-37	-0.3739908	0.954	0.988	5.72E-33	Serbp1	BC_Ageddown	BC
4.91E-22	-0.3744256	0.157	0.351	1.18E-17	Klf9	BC_Ageddown	BC
1.20E-28	-0.3793058	0.882	0.973	2.89E-24	S100a14	BC_Ageddown	BC
6.40E-31	-0.3810911	0.794	0.933	1.54E-26	Cox7a2l	BC_Ageddown	BC
2.85E-31	-0.3811195	0.825	0.955	6.84E-27	Rbm39	BC_Ageddown	BC
2.44E-128	-0.3813097	1	1	5.86E-124	Eef1a1	BC_Ageddown	BC
1.15E-150	-0.3818242	1	1	2.76E-146	mt-Atp6	BC_Ageddown	BC
5.60E-23	-0.3826628	0.362	0.594	1.34E-18	Sox4	BC_Ageddown	BC
8.97E-23	-0.3832994	0.49	0.706	2.15E-18	Ankrd11	BC_Ageddown	BC
1.39E-24	-0.3850257	0.691	0.855	3.34E-20	Slc38a2	BC_Ageddown	BC
6.21E-82	-0.3903637	0.999	1	1.49E-77	Rpl12	BC_Ageddown	BC
1.24E-22	-0.3926955	0.332	0.563	2.98E-18	Lrig3	BC_Ageddown	BC
2.68E-106	-0.3978202	1	1	6.44E-102	Rplp2	BC_Ageddown	BC
6.96E-26	-0.3981141	0.257	0.503	1.67E-21	Cux1	BC_Ageddown	BC
3.89E-26	-0.401377	0.432	0.666	9.33E-22	Ppig	BC_Ageddown	BC
1.64E-102	-0.4020663	0.997	1	3.93E-98	Rpl21	BC_Ageddown	BC
2.69E-35	-0.4084636	0.074	0.305	6.44E-31	Banp	BC_Ageddown	BC
8.64E-27	-0.412146	0.308	0.56	2.07E-22	2900026A02Rik	BC_Ageddown	BC
6.17E-26	-0.412776	0.367	0.599	1.48E-21	Pura	BC_Ageddown	BC
1.67E-29	-0.4158285	0.678	0.86	4.00E-25	Cct4	BC_Ageddown	BC

4.30E-30	-0.4271093	0.21	0.468	1.03E-25	Rgma	BC_Ageddown	BC
5.49E-26	-0.4279207	0.514	0.74	1.32E-21	Btg1	BC_Ageddown	BC
3.65E-26	-0.4290339	0.215	0.452	8.76E-22	Mylip	BC_Ageddown	BC
1.52E-30	-0.430482	0.503	0.754	3.65E-26	Rp9	BC_Ageddown	BC
1.52E-26	-0.4353445	0.358	0.614	3.63E-22	Dlk2	BC_Ageddown	BC
6.72E-27	-0.435658	0.595	0.81	1.61E-22	Golim4	BC_Ageddown	BC
1.24E-29	-0.4380967	0.532	0.759	2.97E-25	Luc7l3	BC_Ageddown	BC
5.16E-138	-0.4445315	0.997	1	1.24E-133	Rpl26	BC_Ageddown	BC
2.85E-30	-0.4464899	0.584	0.812	6.83E-26	Hnrnpc	BC_Ageddown	BC
7.40E-24	-0.4473658	0.308	0.54	1.77E-19	Lmo4	BC_Ageddown	BC
1.67E-30	-0.4487311	0.317	0.578	4.01E-26	Arid5b	BC_Ageddown	BC
1.63E-145	-0.4489333	1	1	3.91E-141	Rps14	BC_Ageddown	BC
1.96E-133	-0.4584068	1	1	4.71E-129	Rplp1	BC_Ageddown	BC
3.13E-32	-0.4611852	0.262	0.531	7.49E-28	Tnrc6c	BC_Ageddown	BC
2.18E-33	-0.4651022	0.339	0.615	5.23E-29	Mettl23	BC_Ageddown	BC
4.24E-47	-0.4696141	0.896	0.978	1.02E-42	Nsa2	BC_Ageddown	BC
1.03E-13	-0.4698023	0.4	0.551	2.48E-09	Glul	BC_Ageddown	BC
6.97E-32	-0.4735669	0.348	0.604	1.67E-27	Tnfaip8	BC_Ageddown	BC
4.62E-12	-0.481911	0.469	0.592	1.11E-07	Capns2	BC_Ageddown	BC
4.69E-46	-0.4990265	0.821	0.944	1.12E-41	Pcbp2	BC_Ageddown	BC
5.07E-35	-0.5016876	0.211	0.482	1.22E-30	Ankrd12	BC_Ageddown	BC
7.48E-42	-0.5058887	0.604	0.844	1.79E-37	Top1	BC_Ageddown	BC
3.95E-44	-0.5067772	0.199	0.514	9.48E-40	Zkscan3	BC_Ageddown	BC
7.67E-67	-0.5094021	0.971	0.999	1.84E-62	Fxyd3	BC_Ageddown	BC
4.78E-40	-0.5141247	0.045	0.279	1.15E-35	Frem2	BC_Ageddown	BC
1.04E-26	-0.5214328	0.294	0.523	2.51E-22	Efemp1	BC_Ageddown	BC
7.99E-54	-0.5215045	0.794	0.952	1.92E-49	Eif3e	BC_Ageddown	BC
9.12E-23	-0.522558	0.734	0.869	2.19E-18	Adh7	BC_Ageddown	BC
3.57E-205	-0.5282432	1	1	8.57E-201	mt-Co3	BC_Ageddown	BC
1.51E-38	-0.5325471	0.179	0.457	3.63E-34	Pdzd2	BC_Ageddown	BC
2.47E-35	-0.5352887	0.363	0.645	5.92E-31	Tsc22d3	BC_Ageddown	BC
2.74E-170	-0.5358878	1	1	6.57E-166	Rps21	BC_Ageddown	BC
5.17E-35	-0.5657045	0.366	0.636	1.24E-30	Snai2	BC_Ageddown	BC
9.20E-32	-0.5673897	0.214	0.458	2.21E-27	Id2	BC_Ageddown	BC
2.96E-99	-0.5677176	0.994	1	7.10E-95	Rps25	BC_Ageddown	BC
1.85E-204	-0.5756717	1	1	4.43E-200	Tpt1	BC_Ageddown	BC
3.64E-43	-0.5768516	0.701	0.88	8.74E-39	Zfp36l2	BC_Ageddown	BC
4.96E-44	-0.5769806	0.134	0.42	1.19E-39	Hspa1a	BC_Ageddown	BC
5.47E-37	-0.5947833	0.578	0.828	1.31E-32	Sbpl	BC_Ageddown	BC
9.42E-123	-0.5984929	0.991	1	2.26E-118	H3f3a	BC_Ageddown	BC
2.49E-45	-0.6101055	0.139	0.431	5.96E-41	Foxe1	BC_Ageddown	BC
2.20E-19	-0.6261359	0.538	0.725	5.27E-15	Junb	BC_Ageddown	BC
3.78E-16	-0.6270725	0.101	0.237	9.06E-12	Sostdc1	BC_Ageddown	BC
1.36E-177	-0.6399245	1	1	3.26E-173	mt-Nd2	BC_Ageddown	BC
4.47E-79	-0.6433851	0.995	0.999	1.07E-74	Rps15	BC_Ageddown	BC
7.93E-39	-0.6436894	0.948	0.99	1.90E-34	Malat1	BC_Ageddown	BC
6.34E-46	-0.6495949	0.267	0.595	1.52E-41	Dbp	BC_Ageddown	BC
2.18E-41	-0.6535976	0.159	0.45	5.22E-37	Gadd45g	BC_Ageddown	BC
1.79E-115	-0.656347	0.977	0.999	4.29E-111	Hspa8	BC_Ageddown	BC
3.79E-66	-0.6581609	0.368	0.763	9.09E-62	Tmem59	BC_Ageddown	BC
2.97E-56	-0.6901723	0.155	0.492	7.12E-52	Hsph1	BC_Ageddown	BC
1.22E-49	-0.6902271	0.552	0.816	2.93E-45	Ccnd1	BC_Ageddown	BC
9.85E-164	-0.6905077	0.997	0.999	2.36E-159	Hsp90ab1	BC_Ageddown	BC
1.37E-61	-0.6951557	0.27	0.638	3.28E-57	Cpn1	BC_Ageddown	BC
5.38E-38	-0.7048269	0.815	0.93	1.29E-33	Wnt4	BC_Ageddown	BC
5.56E-23	-0.707259	0.171	0.365	1.33E-18	Foxq1	BC_Ageddown	BC
1.01E-54	-0.7226882	0.161	0.497	2.43E-50	Gadd45b	BC_Ageddown	BC
1.14E-27	-0.727205	0.174	0.388	2.73E-23	Il1r2	BC_Ageddown	BC
3.06E-52	-0.7359681	0.6	0.844	7.34E-48	Txnip	BC_Ageddown	BC

7.62E-67	-0.7373376	0.91	0.975	1.83E-62	Dst	BC_Ageddown	BC
4.28E-67	-0.7739901	0.559	0.832	1.03E-62	Dnaja1	BC_Ageddown	BC
1.31E-65	-0.818225	0.414	0.752	3.15E-61	Anxa8	BC_Ageddown	BC
4.13E-122	-0.828396	0.965	0.996	9.89E-118	Rpl23a	BC_Ageddown	BC
0	-0.8517897	1	1	0	Rps8	BC_Ageddown	BC
3.12E-67	-0.8538594	0.199	0.577	7.47E-63	Crlf3	BC_Ageddown	BC
3.73E-23	-0.8592495	0.335	0.537	8.93E-19	Egr1	BC_Ageddown	BC
3.12E-71	-0.8987221	0.73	0.96	7.49E-67	Krt6a	BC_Ageddown	BC
5.07E-61	-0.9109216	0.219	0.576	1.22E-56	Aqp3	BC_Ageddown	BC
6.36E-82	-0.9326351	0.405	0.769	1.52E-77	Hspa1b	BC_Ageddown	BC
1.59E-22	-0.9530087	0.252	0.448	3.80E-18	Dcn	BC_Ageddown	BC
1.92E-44	-0.9609094	0.156	0.434	4.61E-40	Maf	BC_Ageddown	BC
4.27E-259	-0.9633457	0.997	1	1.02E-254	Rps12	BC_Ageddown	BC
4.57E-115	-0.9684248	0.783	0.964	1.10E-110	Hsp90aa1	BC_Ageddown	BC
2.27E-21	-1.0754676	0.517	0.735	5.45E-17	Krt17	BC_Ageddown	BC
4.88E-96	-1.1366761	0.569	0.906	1.17E-91	AY036118	BC_Ageddown	BC
7.69E-113	-1.388543	0.272	0.749	1.84E-108	Id1	BC_Ageddown	BC
7.50E-146	-1.4370159	0.907	0.977	1.80E-141	Krt15	BC_Ageddown	BC
6.72E-126	-1.4614993	0.52	0.898	1.61E-121	Id3	BC_Ageddown	BC
5.14E-65	2.98789112	0.438	0.002	1.23E-60	Xist	CBC_Agedup	CBC
5.96E-55	2.56006543	0.811	0.536	1.43E-50	Mt4	CBC_Agedup	CBC
6.39E-91	2.17401909	0.645	0.067	1.53E-86	1600014C10Rik	CBC_Agedup	CBC
1.20E-106	2.00425641	1	0.998	2.87E-102	Gm42418	CBC_Agedup	CBC
4.72E-61	1.86635999	0.821	0.412	1.13E-56	Ifi27l2a	CBC_Agedup	CBC
3.70E-50	1.79027071	0.823	0.555	8.87E-46	Krt4	CBC_Agedup	CBC
1.91E-62	1.72031963	0.436	0.009	4.58E-58	Gm10260	CBC_Agedup	CBC
1.09E-84	1.62354061	0.987	0.987	2.62E-80	Crip1	CBC_Agedup	CBC
6.90E-72	1.59066569	0.885	0.555	1.65E-67	H2-K1	CBC_Agedup	CBC
2.05E-63	1.44314548	0.94	0.828	4.91E-59	H2-D1	CBC_Agedup	CBC
3.97E-50	1.39135845	0.519	0.105	9.53E-46	H19	CBC_Agedup	CBC
1.56E-69	1.3834304	0.906	0.671	3.75E-65	Tppp3	CBC_Agedup	CBC
5.28E-58	1.35559693	0.826	0.473	1.27E-53	B2m	CBC_Agedup	CBC
8.73E-85	1.33253448	0.977	0.924	2.09E-80	mt-Atp8	CBC_Agedup	CBC
1.05E-50	1.31213381	0.843	0.527	2.51E-46	Igfbp2	CBC_Agedup	CBC
6.83E-51	1.28108242	0.672	0.323	1.64E-46	Pttg1	CBC_Agedup	CBC
1.95E-23	1.1291712	0.966	0.956	4.67E-19	Fabp5	CBC_Agedup	CBC
1.13E-103	1.07374226	1	1	2.72E-99	mt-Nd4l	CBC_Agedup	CBC
6.57E-128	1.07282237	1	0.998	1.58E-123	Gm10076	CBC_Agedup	CBC
4.39E-40	1.06112324	0.955	0.811	1.05E-35	Krt13	CBC_Agedup	CBC
2.23E-54	1.03303285	0.926	0.832	5.36E-50	Cstb	CBC_Agedup	CBC
5.46E-43	1.02364352	0.857	0.58	1.31E-38	Ly6a	CBC_Agedup	CBC
2.57E-53	1.01448347	0.93	0.832	6.16E-49	Cst3	CBC_Agedup	CBC
9.01E-40	1.00379968	0.732	0.451	2.16E-35	Igfbp7	CBC_Agedup	CBC
1.21E-32	0.86341209	0.572	0.24	2.90E-28	Calml3	CBC_Agedup	CBC
2.24E-31	0.82671676	0.966	0.906	5.38E-27	Ifitm3	CBC_Agedup	CBC
1.11E-43	0.81830608	0.981	0.985	2.67E-39	Dbi	CBC_Agedup	CBC
1.13E-22	0.81604821	0.538	0.29	2.71E-18	Fam25c	CBC_Agedup	CBC
6.09E-19	0.79502263	0.683	0.538	1.46E-14	4631405K08Rik	CBC_Agedup	CBC
1.06E-38	0.79489823	0.96	0.926	2.54E-34	Crip2	CBC_Agedup	CBC
5.11E-42	0.78581423	0.411	0.061	1.23E-37	Dynap	CBC_Agedup	CBC
2.36E-28	0.76781445	0.381	0.104	5.66E-24	Peg3	CBC_Agedup	CBC
4.43E-34	0.7675399	0.998	0.994	1.06E-29	Mt1	CBC_Agedup	CBC
7.00E-29	0.74574316	0.749	0.58	1.68E-24	Ociad2	CBC_Agedup	CBC
4.55E-43	0.72920336	0.962	0.941	1.09E-38	Tspo	CBC_Agedup	CBC
7.50E-29	0.71170839	0.987	0.956	1.80E-24	Mt2	CBC_Agedup	CBC
6.54E-32	0.70672257	0.44	0.129	1.57E-27	Psmb9	CBC_Agedup	CBC
8.68E-28	0.68708121	0.63	0.388	2.08E-23	Gstp1	CBC_Agedup	CBC
6.45E-10	0.68417425	0.504	0.379	1.55E-05	Apoc1	CBC_Agedup	CBC
3.85E-13	0.67676073	0.449	0.248	9.23E-09	Ndufa4l2	CBC_Agedup	CBC

2.47E-17	0.67086903	0.636	0.388	5.93E-13	Gm94	CBC_Agedup	CBC
4.39E-33	0.66383012	0.836	0.671	1.05E-28	Psme2	CBC_Agedup	CBC
1.01E-16	0.66138556	0.253	0.068	2.43E-12	Isg15	CBC_Agedup	CBC
2.42E-24	0.65629398	0.828	0.695	5.81E-20	Ifi27	CBC_Agedup	CBC
8.55E-20	0.63792458	0.398	0.159	2.05E-15	Chit1	CBC_Agedup	CBC
8.66E-23	0.63416189	0.383	0.135	2.08E-18	Psmb8	CBC_Agedup	CBC
2.50E-49	0.6290865	0.989	0.993	6.00E-45	Ndufa4	CBC_Agedup	CBC
3.97E-28	0.62215772	0.532	0.24	9.51E-24	Snhg20	CBC_Agedup	CBC
2.85E-29	0.61898831	0.953	0.913	6.83E-25	Fam162a	CBC_Agedup	CBC
8.64E-21	0.61120889	0.702	0.494	2.07E-16	Barx2	CBC_Agedup	CBC
7.69E-29	0.59630255	0.917	0.854	1.84E-24	Tmsb10	CBC_Agedup	CBC
4.20E-23	0.57192532	0.223	0.026	1.01E-18	Muc5b	CBC_Agedup	CBC
1.05E-16	0.56859534	0.8	0.684	2.53E-12	Gja1	CBC_Agedup	CBC
1.87E-31	0.54990423	0.957	0.963	4.49E-27	Reep5	CBC_Agedup	CBC
4.81E-81	0.54664175	1	1	1.15E-76	Rpl11	CBC_Agedup	CBC
1.02E-15	0.54267415	0.481	0.287	2.45E-11	Gas1	CBC_Agedup	CBC
3.07E-43	0.54109178	0.989	0.994	7.36E-39	Uba52	CBC_Agedup	CBC
5.93E-26	0.53616738	0.911	0.893	1.42E-21	Prelid1	CBC_Agedup	CBC
3.25E-38	0.53154926	0.985	0.993	7.80E-34	Cox5a	CBC_Agedup	CBC
2.02E-15	0.52126239	0.372	0.179	4.84E-11	Slc7a5	CBC_Agedup	CBC
5.26E-18	0.51314151	0.598	0.427	1.26E-13	Acp5	CBC_Agedup	CBC
2.85E-16	0.50049255	0.221	0.048	6.83E-12	Mt3	CBC_Agedup	CBC
1.94E-26	0.49988298	0.368	0.104	4.65E-22	Cpxm2	CBC_Agedup	CBC
5.59E-65	0.49750718	1	1	1.34E-60	mt-Co1	CBC_Agedup	CBC
6.16E-40	0.49181553	0.989	0.998	1.48E-35	Cox6b1	CBC_Agedup	CBC
6.94E-13	0.48842181	0.955	0.972	1.66E-08	Sfn	CBC_Agedup	CBC
5.08E-37	0.48451342	0.991	0.989	1.22E-32	Uqcr11	CBC_Agedup	CBC
1.41E-18	0.48437773	0.847	0.83	3.39E-14	Capg	CBC_Agedup	CBC
8.37E-18	0.48318348	0.785	0.701	2.01E-13	Ndufa3	CBC_Agedup	CBC
1.60E-11	0.47789102	0.762	0.673	3.83E-07	Them5	CBC_Agedup	CBC
1.05E-15	0.47609513	0.347	0.148	2.53E-11	Tap1	CBC_Agedup	CBC
7.38E-25	0.47599925	0.351	0.096	1.77E-20	Gm10036	CBC_Agedup	CBC
7.12E-26	0.47287625	0.949	0.937	1.71E-21	Ndufb8	CBC_Agedup	CBC
2.19E-23	0.47007476	0.955	0.95	5.25E-19	Anxa2	CBC_Agedup	CBC
5.40E-08	0.46868119	0.27	0.144	0.001294	2610528A11Rik	CBC_Agedup	CBC
4.73E-11	0.4686174	0.836	0.791	1.13E-06	Lgals3	CBC_Agedup	CBC
2.83E-14	0.46233968	0.494	0.307	6.78E-10	Car12	CBC_Agedup	CBC
2.76E-12	0.46176316	0.574	0.44	6.62E-08	Gas6	CBC_Agedup	CBC
1.41E-11	0.46061184	0.587	0.41	3.38E-07	Fam213a	CBC_Agedup	CBC
1.42E-24	0.45427433	0.372	0.116	3.41E-20	Tnni2	CBC_Agedup	CBC
7.22E-14	0.45241956	0.581	0.427	1.73E-09	Coro1c	CBC_Agedup	CBC
9.19E-26	0.45190131	0.217	0.013	2.20E-21	Tff2	CBC_Agedup	CBC
2.08E-11	0.45155203	0.664	0.593	4.99E-07	Csrp1	CBC_Agedup	CBC
5.90E-14	0.44700086	0.777	0.708	1.41E-09	Pdlim1	CBC_Agedup	CBC
3.06E-53	0.44566616	1	0.998	7.34E-49	Rpl35	CBC_Agedup	CBC
1.29E-15	0.4436706	0.309	0.116	3.08E-11	Ly6c1	CBC_Agedup	CBC
2.64E-13	0.44084478	0.713	0.571	6.33E-09	Aldoa	CBC_Agedup	CBC
5.44E-77	0.44029077	1	1	1.30E-72	Rps28	CBC_Agedup	CBC
3.03E-38	0.43939665	0.996	0.994	7.26E-34	Atp5g2	CBC_Agedup	CBC
2.29E-28	0.43509734	0.253	0.022	5.50E-24	Aknad1	CBC_Agedup	CBC
1.93E-09	0.43429503	0.283	0.146	4.62E-05	Igfbp5	CBC_Agedup	CBC
5.46E-58	0.43082883	1	1	1.31E-53	Rpl36	CBC_Agedup	CBC
2.67E-16	0.42956846	0.847	0.804	6.41E-12	Erh	CBC_Agedup	CBC
6.45E-15	0.4224564	0.847	0.821	1.55E-10	Elof1	CBC_Agedup	CBC
1.07E-09	0.42200922	0.657	0.58	2.57E-05	Gm47283	CBC_Agedup	CBC
7.24E-24	0.41566918	0.998	0.991	1.74E-19	Mif	CBC_Agedup	CBC
1.24E-15	0.41382041	0.881	0.848	2.98E-11	Mrpl23	CBC_Agedup	CBC
1.82E-12	0.41286037	0.826	0.789	4.37E-08	Dsc3	CBC_Agedup	CBC
3.11E-13	0.41227749	0.504	0.327	7.45E-09	Klk10	CBC_Agedup	CBC

8.93E-22	0.41219106	0.951	0.957	2.14E-17	Ndufa11	CBC_Agedup	CBC
7.98E-14	0.41079846	0.836	0.8	1.91E-09	Cycs	CBC_Agedup	CBC
1.58E-17	0.41036732	0.896	0.884	3.80E-13	Ndufs7	CBC_Agedup	CBC
1.63E-24	0.40885054	0.968	0.974	3.90E-20	Atp5g3	CBC_Agedup	CBC
1.16E-19	0.40644877	0.936	0.919	2.77E-15	Ndufs6	CBC_Agedup	CBC
1.91E-22	0.40184425	0.998	1	4.59E-18	Txn1	CBC_Agedup	CBC
1.60E-14	0.40177247	0.921	0.924	3.83E-10	Cox6a1	CBC_Agedup	CBC
5.03E-14	0.40079103	0.372	0.179	1.21E-09	Cald1	CBC_Agedup	CBC
9.92E-22	0.40020584	0.262	0.054	2.38E-17	Clec11a	CBC_Agedup	CBC
1.01E-12	0.39916916	0.23	0.076	2.42E-08	Bst2	CBC_Agedup	CBC
1.08E-18	0.39810155	0.234	0.044	2.60E-14	H2-Q7	CBC_Agedup	CBC
6.61E-26	0.39248341	0.994	0.996	1.58E-21	Cox5b	CBC_Agedup	CBC
2.11E-19	0.39186598	0.994	0.998	5.06E-15	Ftl1	CBC_Agedup	CBC
2.58E-08	0.39107707	0.521	0.403	0.000619	Ptgr1	CBC_Agedup	CBC
3.33E-47	0.39021497	1	1	7.99E-43	Rpl38	CBC_Agedup	CBC
2.88E-12	0.38931966	0.76	0.747	6.91E-08	Ndufv3	CBC_Agedup	CBC
1.33E-06	0.38875407	0.683	0.588	0.031864	Crabp1	CBC_Agedup	CBC
4.83E-07	0.38772607	0.555	0.418	0.01158	Calm4	CBC_Agedup	CBC
1.09E-10	0.38558187	0.643	0.56	2.61E-06	1810022K09Rik	CBC_Agedup	CBC
5.55E-10	0.38364211	0.672	0.562	1.33E-05	Hif1a	CBC_Agedup	CBC
2.38E-14	0.38243541	0.832	0.795	5.70E-10	Mrpl54	CBC_Agedup	CBC
1.00E-12	0.37993287	0.87	0.834	2.40E-08	Ckmt1	CBC_Agedup	CBC
1.32E-53	0.37882748	1	1	3.16E-49	Rpl37a	CBC_Agedup	CBC
2.43E-09	0.37748699	0.611	0.512	5.82E-05	Flna	CBC_Agedup	CBC
1.04E-11	0.37721196	0.468	0.318	2.50E-07	Ids	CBC_Agedup	CBC
3.52E-22	0.37720461	0.979	0.989	8.44E-18	Ndufb9	CBC_Agedup	CBC
2.64E-11	0.3753185	0.485	0.323	6.32E-07	Eppk1	CBC_Agedup	CBC
3.86E-21	0.373972	0.968	0.978	9.25E-17	Uqcrq	CBC_Agedup	CBC
1.48E-12	0.37290756	0.809	0.776	3.54E-08	Aprt	CBC_Agedup	CBC
6.85E-08	0.3712109	0.555	0.481	0.001643	Setd5	CBC_Agedup	CBC
8.03E-17	0.37090634	0.938	0.956	1.92E-12	Mrpl52	CBC_Agedup	CBC
1.05E-09	0.36632238	0.76	0.732	2.52E-05	Ddb1	CBC_Agedup	CBC
3.32E-17	0.3656869	0.947	0.98	7.95E-13	Snrpg	CBC_Agedup	CBC
3.07E-13	0.36480589	0.879	0.848	7.37E-09	Nop10	CBC_Agedup	CBC
2.47E-13	0.36402522	0.887	0.86	5.93E-09	Usmg5	CBC_Agedup	CBC
1.94E-07	0.36384757	0.826	0.806	0.004655	Pkp1	CBC_Agedup	CBC
7.03E-09	0.36375163	0.383	0.248	0.000169	Klk11	CBC_Agedup	CBC
5.09E-08	0.35892661	0.909	0.865	0.001221	Dmkn	CBC_Agedup	CBC
7.84E-39	0.35836475	1	1	1.88E-34	Rps2	CBC_Agedup	CBC
2.09E-10	0.35731344	0.53	0.388	5.01E-06	Ggh	CBC_Agedup	CBC
5.83E-14	0.35695633	0.904	0.908	1.40E-09	Ndufc2	CBC_Agedup	CBC
1.36E-37	0.35570103	0.998	1	3.25E-33	Rpl10a	CBC_Agedup	CBC
2.60E-42	0.35463846	1	1	6.23E-38	Rps20	CBC_Agedup	CBC
2.47E-17	0.35380363	0.983	0.991	5.92E-13	Selenow	CBC_Agedup	CBC
4.99E-47	0.35370656	1	1	1.20E-42	Rps19	CBC_Agedup	CBC
9.32E-08	0.35276782	0.272	0.146	0.002234	Tgm3	CBC_Agedup	CBC
1.36E-08	0.35243779	0.826	0.815	0.000325	Atp6v1g1	CBC_Agedup	CBC
5.14E-14	0.35024622	0.889	0.902	1.23E-09	Ndufa1	CBC_Agedup	CBC
7.30E-11	0.34857384	0.817	0.797	1.75E-06	Ndufb6	CBC_Agedup	CBC
2.33E-07	0.34611325	0.357	0.24	0.005584	Crlf1	CBC_Agedup	CBC
5.27E-08	0.34176818	0.626	0.56	0.001264	2-Mar	CBC_Agedup	CBC
1.00E-08	0.33910602	0.777	0.741	0.00024	Rsrp1	CBC_Agedup	CBC
2.04E-08	0.33869968	0.66	0.577	0.000488	Psme1	CBC_Agedup	CBC
2.84E-08	0.33851571	0.574	0.495	0.000682	Gm10073	CBC_Agedup	CBC
8.88E-09	0.33723143	0.406	0.261	0.000213	Rora	CBC_Agedup	CBC
9.66E-12	0.33645523	0.894	0.891	2.32E-07	Taf10	CBC_Agedup	CBC
1.57E-11	0.33632733	0.77	0.732	3.76E-07	Lsm7	CBC_Agedup	CBC
2.85E-10	0.33589276	0.853	0.86	6.84E-06	Cyc1	CBC_Agedup	CBC
9.01E-09	0.33459632	0.73	0.688	0.000216	Csnk2b	CBC_Agedup	CBC

1.96E-19	0.32982794	0.215	0.033	4.69E-15	Lgals9	CBC_Agedup	CBC
2.48E-14	0.32969647	0.951	0.963	5.94E-10	Edf1	CBC_Agedup	CBC
2.74E-09	0.32937763	0.804	0.808	6.58E-05	Sdhb	CBC_Agedup	CBC
2.52E-07	0.32477934	0.579	0.484	0.006045	Cirbp	CBC_Agedup	CBC
8.03E-14	0.32252175	0.964	0.976	1.92E-09	Ppp1r14b	CBC_Agedup	CBC
4.77E-09	0.32052575	0.477	0.338	0.000114	Car13	CBC_Agedup	CBC
2.06E-23	0.31862839	1	0.998	4.94E-19	Cox6c	CBC_Agedup	CBC
2.42E-09	0.31699679	0.781	0.73	5.79E-05	Phgdh	CBC_Agedup	CBC
1.74E-36	0.3157327	1	1	4.17E-32	Rpl37	CBC_Agedup	CBC
4.10E-15	0.31517129	0.979	0.982	9.84E-11	Uqcr10	CBC_Agedup	CBC
2.06E-14	0.31452745	0.24	0.074	4.94E-10	Dhcr24	CBC_Agedup	CBC
2.81E-12	0.31377333	0.943	0.969	6.73E-08	Atp5k	CBC_Agedup	CBC
2.72E-50	0.3137425	1	1	6.52E-46	Rps29	CBC_Agedup	CBC
3.70E-09	0.31361748	0.932	0.928	8.87E-05	Uqcrc1	CBC_Agedup	CBC
1.01E-06	0.31356236	0.757	0.706	0.024185	Ddx3x	CBC_Agedup	CBC
3.09E-08	0.31232989	0.598	0.518	0.00074	Dnajc15	CBC_Agedup	CBC
2.10E-09	0.31094764	0.596	0.495	5.05E-05	Pet100	CBC_Agedup	CBC
5.49E-11	0.30921102	0.923	0.93	1.32E-06	2410015M20Rik	CBC_Agedup	CBC
1.63E-37	0.3087201	1	1	3.92E-33	Rpl18a	CBC_Agedup	CBC
1.47E-08	0.30706372	0.719	0.675	0.000352	Dnajc19	CBC_Agedup	CBC
8.30E-08	0.30698466	0.715	0.695	0.00199	Hcfc1r1	CBC_Agedup	CBC
6.35E-10	0.30672173	0.757	0.667	1.52E-05	Churc1	CBC_Agedup	CBC
2.71E-07	0.30595521	0.679	0.64	0.0065	Cenpw	CBC_Agedup	CBC
2.73E-08	0.3058953	0.813	0.828	0.000654	Naa38	CBC_Agedup	CBC
3.56E-10	0.30589505	0.889	0.889	8.54E-06	Mrpl33	CBC_Agedup	CBC
5.10E-10	0.30309993	0.796	0.745	1.22E-05	Fis1	CBC_Agedup	CBC
4.84E-07	0.30289338	0.647	0.579	0.011615	Cs	CBC_Agedup	CBC
2.30E-07	0.30234914	0.304	0.19	0.005523	Stox2	CBC_Agedup	CBC
1.23E-08	0.30159401	0.957	0.954	0.000296	S100a16	CBC_Agedup	CBC
2.85E-07	0.2977012	0.423	0.309	0.006832	Npdc1	CBC_Agedup	CBC
4.93E-09	0.29740983	0.906	0.872	0.000118	Nme1	CBC_Agedup	CBC
2.69E-12	0.29683192	0.962	0.957	6.46E-08	Timm13	CBC_Agedup	CBC
1.99E-06	0.29623281	0.445	0.355	0.047791	Rnf141	CBC_Agedup	CBC
1.98E-06	0.29583955	0.781	0.745	0.047532	Cavin1	CBC_Agedup	CBC
6.19E-08	0.29573919	0.506	0.41	0.001486	Svbp	CBC_Agedup	CBC
6.70E-08	0.29566941	0.67	0.632	0.001607	1810058I24Rik	CBC_Agedup	CBC
1.34E-09	0.29551834	0.883	0.893	3.22E-05	Trmt112	CBC_Agedup	CBC
5.48E-09	0.29488682	0.928	0.945	0.000131	H2afj	CBC_Agedup	CBC
4.38E-08	0.29424543	0.821	0.762	0.001052	Srsf5	CBC_Agedup	CBC
1.59E-07	0.29369273	0.753	0.736	0.003801	Higd2a	CBC_Agedup	CBC
8.94E-09	0.29261509	0.294	0.161	0.000214	Emc9	CBC_Agedup	CBC
8.10E-09	0.29252892	0.794	0.756	0.000194	Mrpl57	CBC_Agedup	CBC
4.69E-07	0.29243155	0.377	0.262	0.011257	Il17rc	CBC_Agedup	CBC
2.33E-15	0.29184756	0.251	0.076	5.58E-11	Adat2	CBC_Agedup	CBC
2.41E-08	0.29004642	0.838	0.863	0.000578	Ndufa8	CBC_Agedup	CBC
1.62E-13	0.28923069	0.977	0.991	3.88E-09	Atp5g1	CBC_Agedup	CBC
7.71E-09	0.28900428	0.902	0.93	0.000185	Spint2	CBC_Agedup	CBC
4.61E-11	0.28791861	0.213	0.074	1.11E-06	Cxcl14	CBC_Agedup	CBC
1.20E-07	0.28717354	0.8	0.83	0.002866	Romo1	CBC_Agedup	CBC
8.64E-08	0.28551346	0.774	0.754	0.002073	Uqcrfs1	CBC_Agedup	CBC
3.81E-11	0.28547564	0.951	0.97	9.13E-07	Pgls	CBC_Agedup	CBC
1.86E-08	0.28418938	0.898	0.896	0.000447	D8Ertd738e	CBC_Agedup	CBC
1.41E-06	0.28376914	0.653	0.619	0.03392	Nudt14	CBC_Agedup	CBC
7.17E-07	0.28241759	0.445	0.351	0.017203	Tmem43	CBC_Agedup	CBC
9.69E-11	0.28211067	0.153	0.037	2.32E-06	Rtp4	CBC_Agedup	CBC
2.21E-15	0.2813771	0.219	0.054	5.30E-11	Arhgdib	CBC_Agedup	CBC
9.46E-12	0.28099715	0.206	0.065	2.27E-07	Samd9l	CBC_Agedup	CBC
4.64E-07	0.28099231	0.798	0.769	0.011129	Bcl7c	CBC_Agedup	CBC
1.14E-06	0.28008695	0.598	0.523	0.02727	Ubl4a	CBC_Agedup	CBC

1.11E-07	0.2796365	0.338	0.214	0.002669	S100a1	CBC_Agedup	CBC
1.25E-06	0.27935944	0.809	0.865	0.029877	Ndufs8	CBC_Agedup	CBC
8.01E-09	0.27747749	0.991	0.985	0.000192	Lmna	CBC_Agedup	CBC
9.02E-18	0.27632294	0.996	0.998	2.16E-13	Pfn1	CBC_Agedup	CBC
1.54E-11	0.27630016	0.962	0.963	3.68E-07	Polr1d	CBC_Agedup	CBC
1.39E-08	0.27597752	0.262	0.131	0.000334	Pitx2	CBC_Agedup	CBC
9.71E-28	0.27531818	1	1	2.33E-23	Rpl39	CBC_Agedup	CBC
1.45E-37	0.27460166	1	1	3.48E-33	Rpl41	CBC_Agedup	CBC
1.63E-11	0.27330694	0.966	0.983	3.92E-07	Rpl27	CBC_Agedup	CBC
5.90E-11	0.27039379	0.174	0.05	1.41E-06	Acta1	CBC_Agedup	CBC
1.05E-09	0.26469882	0.232	0.1	2.52E-05	H2-Q4	CBC_Agedup	CBC
2.67E-11	0.26414353	0.962	0.948	6.41E-07	2010107E04Rik	CBC_Agedup	CBC
1.42E-06	0.26348254	0.806	0.813	0.034071	Cox14	CBC_Agedup	CBC
3.27E-08	0.26330045	0.221	0.105	0.000785	Kcnj2	CBC_Agedup	CBC
1.14E-06	0.26296749	0.26	0.15	0.027261	Fam46a	CBC_Agedup	CBC
1.70E-06	0.26243941	0.462	0.355	0.040875	Fat2	CBC_Agedup	CBC
5.67E-08	0.26241534	0.821	0.638	0.001361	Apoe	CBC_Agedup	CBC
2.13E-16	0.26044073	0.162	0.018	5.10E-12	Gm11361	CBC_Agedup	CBC
1.54E-19	0.26039891	0.998	1	3.69E-15	Rpl7a	CBC_Agedup	CBC
1.36E-06	0.26004541	0.17	0.078	0.032558	Tap2	CBC_Agedup	CBC
7.88E-07	0.25774241	0.832	0.823	0.018887	Ndufb2	CBC_Agedup	CBC
1.08E-14	0.25605798	0.115	0.004	2.60E-10	Cdkn2a	CBC_Agedup	CBC
5.68E-07	0.2531104	0.813	0.773	0.013626	Mrps16	CBC_Agedup	CBC
3.84E-08	0.25200391	0.347	0.207	0.000921	Plbd1	CBC_Agedup	CBC
2.20E-14	0.25150017	0.985	0.994	5.27E-10	Elob	CBC_Agedup	CBC
3.34E-07	0.25143324	0.304	0.183	0.008014	Mgmt	CBC_Agedup	CBC
1.71E-06	0.25084704	0.832	0.884	0.041058	Ndufb4	CBC_Agedup	CBC
1.40E-08	0.24990655	0.96	0.952	0.000337	Ldha	CBC_Agedup	CBC
4.14E-08	0.2498482	0.983	0.978	0.000992	Pabpc1	CBC_Agedup	CBC
3.67E-07	0.2493245	0.798	0.808	0.008803	Ndufv2	CBC_Agedup	CBC
5.31E-07	0.24746518	0.309	0.192	0.01274	Slc2a1	CBC_Agedup	CBC
7.45E-23	0.24734432	1	1	1.79E-18	mt-Co2	CBC_Agedup	CBC
1.36E-08	0.24706548	0.168	0.061	0.000327	Bmp3	CBC_Agedup	CBC
1.99E-13	0.24684504	0.236	0.074	4.78E-09	Akr1b8	CBC_Agedup	CBC
8.68E-10	0.24627782	0.998	0.996	2.08E-05	mt-Nd5	CBC_Agedup	CBC
5.15E-07	0.24374837	0.896	0.893	0.012341	Ost4	CBC_Agedup	CBC
3.32E-07	0.24280298	0.864	0.902	0.007964	Spcs1	CBC_Agedup	CBC
8.02E-07	0.24083351	0.215	0.107	0.01923	Pdzk1ip1	CBC_Agedup	CBC
1.19E-12	0.24023398	0.191	0.052	2.86E-08	Insl6	CBC_Agedup	CBC
2.27E-07	0.24001029	0.906	0.917	0.005442	Ndufa5	CBC_Agedup	CBC
3.00E-08	0.23969262	0.934	0.967	0.000719	Ndufb10	CBC_Agedup	CBC
7.45E-07	0.23595844	0.191	0.091	0.01787	Sqle	CBC_Agedup	CBC
1.04E-06	0.23168379	0.881	0.887	0.024952	Zfp706	CBC_Agedup	CBC
2.58E-08	0.22716427	0.172	0.065	0.000619	Trim7	CBC_Agedup	CBC
9.42E-11	0.2250035	0.989	0.998	2.26E-06	Cox7a2	CBC_Agedup	CBC
8.94E-08	0.22451542	0.732	0.612	0.002145	Ly6e	CBC_Agedup	CBC
5.73E-09	0.22421015	0.13	0.033	0.000137	Tnnc2	CBC_Agedup	CBC
1.90E-13	0.22313671	0.123	0.011	4.56E-09	Fmo1	CBC_Agedup	CBC
2.36E-11	0.22202394	0.157	0.037	5.67E-07	Cd59a	CBC_Agedup	CBC
3.79E-07	0.22169557	0.932	0.957	0.009088	Pebp1	CBC_Agedup	CBC
1.74E-06	0.22134964	0.215	0.115	0.041809	Lrrc8b	CBC_Agedup	CBC
3.70E-07	0.21866765	0.989	0.996	0.008883	Atp5j	CBC_Agedup	CBC
1.67E-08	0.21837604	0.16	0.054	0.000401	Xaf1	CBC_Agedup	CBC
1.75E-06	0.2172847	0.894	0.908	0.042018	Ndufb7	CBC_Agedup	CBC
4.17E-18	0.21725551	1	1	1.00E-13	Rps27	CBC_Agedup	CBC
8.45E-10	0.21713485	0.217	0.085	2.03E-05	Ocel1	CBC_Agedup	CBC
1.15E-11	0.21427462	0.164	0.039	2.75E-07	Tppp	CBC_Agedup	CBC
6.43E-19	0.21319328	1	1	1.54E-14	Rps26	CBC_Agedup	CBC
1.10E-11	0.21244321	0.136	0.024	2.63E-07	Irx4	CBC_Agedup	CBC

1.93E-07	0.21233858	0.2	0.092	0.004638	Epas1	CBC_Agedup	CBC
1.78E-06	0.21102153	0.196	0.098	0.042631	Klk13	CBC_Agedup	CBC
3.52E-20	0.2095633	1	1	8.45E-16	Rps13	CBC_Agedup	CBC
1.44E-06	0.20936518	0.285	0.166	0.034481	Lgals1	CBC_Agedup	CBC
7.24E-09	0.20901784	0.268	0.131	0.000174	Gale	CBC_Agedup	CBC
4.65E-08	0.20540885	0.96	0.989	0.001116	Tomm7	CBC_Agedup	CBC
1.85E-14	-0.2003613	1	1	4.44E-10	Rack1	CBC_Ageddown	CBC
4.50E-11	-0.2008306	0.155	0.353	1.08E-06	H2afy2	CBC_Ageddown	CBC
1.06E-06	-0.201769	0.502	0.684	0.025453	Eif2s1	CBC_Ageddown	CBC
8.64E-07	-0.2018967	0.494	0.669	0.020723	Chmp5	CBC_Ageddown	CBC
8.12E-13	-0.2021223	0.064	0.233	1.95E-08	Tspan13	CBC_Ageddown	CBC
7.09E-08	-0.202498	0.453	0.678	0.0017	Lyar	CBC_Ageddown	CBC
1.03E-07	-0.2026822	0.253	0.431	0.002466	Taf1	CBC_Ageddown	CBC
1.68E-08	-0.2028968	0.349	0.549	0.000403	Cuedc2	CBC_Ageddown	CBC
8.66E-08	-0.2032958	0.232	0.41	0.002077	Zbtb20	CBC_Ageddown	CBC
1.46E-06	-0.2036217	0.655	0.799	0.035073	Bcap31	CBC_Ageddown	CBC
2.84E-07	-0.2041277	0.472	0.656	0.006813	Kmt2e	CBC_Ageddown	CBC
1.00E-08	-0.2048437	0.211	0.388	0.00024	Acaa1a	CBC_Ageddown	CBC
8.67E-09	-0.2051122	0.215	0.399	0.000208	Helz	CBC_Ageddown	CBC
1.74E-10	-0.2052258	0.123	0.299	4.17E-06	Pim1	CBC_Ageddown	CBC
2.65E-08	-0.2052456	0.155	0.316	0.000636	Lef1	CBC_Ageddown	CBC
5.58E-10	-0.2053462	0.177	0.359	1.34E-05	D030056L22Rik	CBC_Ageddown	CBC
3.09E-09	-0.2063887	0.402	0.623	7.41E-05	Rnps1	CBC_Ageddown	CBC
1.69E-06	-0.2069	0.591	0.75	0.040528	Tsn	CBC_Ageddown	CBC
3.94E-07	-0.2069101	0.655	0.832	0.009437	Txnl1	CBC_Ageddown	CBC
1.33E-06	-0.2071412	0.553	0.741	0.031897	Cdc37	CBC_Ageddown	CBC
3.65E-12	-0.207507	0.087	0.264	8.75E-08	Gkap1	CBC_Ageddown	CBC
9.15E-07	-0.2076478	0.066	0.168	0.021933	Il1r2	CBC_Ageddown	CBC
1.26E-08	-0.2082854	0.619	0.808	0.000302	Mat2a	CBC_Ageddown	CBC
6.05E-07	-0.2084051	0.281	0.445	0.014498	Xpa	CBC_Ageddown	CBC
1.26E-09	-0.2086658	0.238	0.453	3.03E-05	Aig1	CBC_Ageddown	CBC
5.54E-08	-0.2097956	0.713	0.893	0.001328	Nono	CBC_Ageddown	CBC
4.04E-07	-0.2099695	0.185	0.336	0.009679	Serpinb8	CBC_Ageddown	CBC
6.72E-09	-0.2107547	0.198	0.379	0.000161	Rel	CBC_Ageddown	CBC
5.61E-08	-0.2113764	0.023	0.111	0.001347	Thbs1	CBC_Ageddown	CBC
1.80E-09	-0.211815	0.243	0.44	4.32E-05	Smn1	CBC_Ageddown	CBC
5.38E-10	-0.2119278	0.17	0.355	1.29E-05	Dusp6	CBC_Ageddown	CBC
1.06E-09	-0.2119789	0.091	0.242	2.55E-05	Etv1	CBC_Ageddown	CBC
4.89E-15	-0.212367	0.021	0.172	1.17E-10	Nav2	CBC_Ageddown	CBC
2.63E-07	-0.2123982	0.449	0.651	0.006296	Ppp2r2a	CBC_Ageddown	CBC
4.11E-07	-0.2127836	0.434	0.612	0.009845	Ubxn4	CBC_Ageddown	CBC
4.20E-08	-0.2127855	0.7	0.845	0.001008	Cct8	CBC_Ageddown	CBC
7.40E-08	-0.2128232	0.332	0.525	0.001774	Grhl2	CBC_Ageddown	CBC
2.27E-11	-0.2128401	0.16	0.368	5.45E-07	Zfp704	CBC_Ageddown	CBC
8.45E-07	-0.2129502	0.321	0.49	0.020267	Strbp	CBC_Ageddown	CBC
8.46E-08	-0.2129624	0.413	0.606	0.002028	Gadd45gip1	CBC_Ageddown	CBC
2.41E-07	-0.2133394	0.662	0.813	0.005775	Vapa	CBC_Ageddown	CBC
4.76E-10	-0.2138771	0.155	0.335	1.14E-05	Ppm1l	CBC_Ageddown	CBC
9.89E-07	-0.2146067	0.777	0.887	0.023707	Manf	CBC_Ageddown	CBC
1.37E-09	-0.2149165	0.362	0.573	3.29E-05	Sbds	CBC_Ageddown	CBC
1.14E-09	-0.2149324	0.23	0.427	2.74E-05	Gps2	CBC_Ageddown	CBC
8.61E-09	-0.2151886	0.351	0.569	0.000206	Vbp1	CBC_Ageddown	CBC
1.23E-08	-0.2152945	0.296	0.503	0.000296	Ammecr1	CBC_Ageddown	CBC
2.25E-07	-0.215856	0.387	0.579	0.005395	Polr2a	CBC_Ageddown	CBC
1.82E-06	-0.2160287	0.502	0.656	0.043541	Psmd14	CBC_Ageddown	CBC
2.52E-07	-0.2162572	0.368	0.555	0.006054	Lrig3	CBC_Ageddown	CBC
1.39E-06	-0.216273	0.887	0.969	0.033264	Nsa2	CBC_Ageddown	CBC
4.24E-09	-0.2164585	0.274	0.477	0.000102	Cenpk	CBC_Ageddown	CBC
5.18E-09	-0.2167828	0.274	0.473	0.000124	Larp7	CBC_Ageddown	CBC

1.13E-06	-0.2169616	0.677	0.848	0.027201	Tmed9	CBC_Ageddown	CBC
4.48E-10	-0.2173224	0.189	0.375	1.08E-05	Haus8	CBC_Ageddown	CBC
1.81E-12	-0.2176065	0.117	0.312	4.35E-08	Pknox1	CBC_Ageddown	CBC
6.03E-10	-0.2178569	0.106	0.266	1.45E-05	Dpysl3	CBC_Ageddown	CBC
2.32E-07	-0.2178741	0.477	0.669	0.005569	Cd164	CBC_Ageddown	CBC
1.19E-10	-0.2183472	0.268	0.49	2.86E-06	Khsrp	CBC_Ageddown	CBC
3.42E-07	-0.2183474	0.285	0.46	0.008203	Tmem33	CBC_Ageddown	CBC
3.85E-08	-0.2187471	0.302	0.505	0.000922	Fam111a	CBC_Ageddown	CBC
8.55E-07	-0.2188386	0.432	0.617	0.020494	Anapc16	CBC_Ageddown	CBC
7.85E-09	-0.2189149	0.609	0.789	0.000188	Khdrbs1	CBC_Ageddown	CBC
1.34E-06	-0.2191059	0.4	0.556	0.032194	Ccdc59	CBC_Ageddown	CBC
1.34E-06	-0.2193027	0.351	0.519	0.032061	Slbp	CBC_Ageddown	CBC
1.12E-09	-0.2197858	0.302	0.519	2.67E-05	Smarca2	CBC_Ageddown	CBC
1.87E-16	-0.2205451	1	1	4.49E-12	mt-Nd4	CBC_Ageddown	CBC
2.17E-08	-0.2212073	0.287	0.473	0.00052	Zkscan3	CBC_Ageddown	CBC
4.01E-07	-0.221892	0.4	0.571	0.009613	Tst	CBC_Ageddown	CBC
1.06E-07	-0.2224838	0.285	0.468	0.002537	Rassf1	CBC_Ageddown	CBC
6.66E-07	-0.2225064	0.564	0.76	0.015976	Prdx4	CBC_Ageddown	CBC
1.24E-10	-0.2225747	0.113	0.275	2.98E-06	Tjap1	CBC_Ageddown	CBC
8.03E-07	-0.2227326	0.577	0.76	0.01926	Vps36	CBC_Ageddown	CBC
1.35E-08	-0.2234092	0.757	0.919	0.000325	Sumo1	CBC_Ageddown	CBC
1.27E-11	-0.2234408	0.138	0.336	3.04E-07	Casp3	CBC_Ageddown	CBC
2.27E-08	-0.2237069	0.389	0.586	0.000544	Ak6	CBC_Ageddown	CBC
2.32E-08	-0.2238893	0.381	0.588	0.000556	Tnrc6b	CBC_Ageddown	CBC
5.90E-09	-0.2239913	0.27	0.481	0.000141	Parva	CBC_Ageddown	CBC
5.46E-14	-0.2242682	0.083	0.275	1.31E-09	Epha4	CBC_Ageddown	CBC
1.05E-07	-0.2245213	0.845	0.943	0.002506	Ptprf	CBC_Ageddown	CBC
2.29E-22	-0.2245975	1	1	5.49E-18	mt-Atp6	CBC_Ageddown	CBC
2.41E-09	-0.2255357	0.279	0.492	5.77E-05	Kat6a	CBC_Ageddown	CBC
2.59E-08	-0.2255796	0.515	0.726	0.000622	Kdelr1	CBC_Ageddown	CBC
1.49E-06	-0.2257522	0.715	0.876	0.035688	Nol7	CBC_Ageddown	CBC
3.27E-08	-0.226247	0.389	0.591	0.000784	Atp6v1a	CBC_Ageddown	CBC
1.50E-09	-0.2265674	0.955	0.991	3.60E-05	mt-Nd3	CBC_Ageddown	CBC
1.25E-07	-0.2273399	0.513	0.706	0.002998	Elavl1	CBC_Ageddown	CBC
3.87E-08	-0.227366	0.372	0.582	0.000928	Bzw2	CBC_Ageddown	CBC
4.72E-07	-0.2279148	0.279	0.449	0.011314	Hist1h1d	CBC_Ageddown	CBC
3.61E-10	-0.2287257	0.204	0.401	8.66E-06	Eva1c	CBC_Ageddown	CBC
3.67E-12	-0.2293302	0.074	0.237	8.79E-08	Gt(ROSA)26Sor	CBC_Ageddown	CBC
6.11E-07	-0.229572	0.647	0.797	0.014643	Sh3glb1	CBC_Ageddown	CBC
7.51E-08	-0.2295996	0.477	0.669	0.001802	Dbf4	CBC_Ageddown	CBC
8.14E-10	-0.2299322	0.281	0.481	1.95E-05	Rbmxl1	CBC_Ageddown	CBC
7.69E-08	-0.2299898	0.351	0.54	0.001843	Phf3	CBC_Ageddown	CBC
5.17E-08	-0.2302769	0.557	0.771	0.001239	Rnf187	CBC_Ageddown	CBC
4.18E-09	-0.2305338	0.489	0.688	0.0001	Pum1	CBC_Ageddown	CBC
5.95E-09	-0.231248	0.223	0.403	0.000143	Rassf9	CBC_Ageddown	CBC
3.22E-07	-0.2319933	0.73	0.86	0.007716	Itgb1	CBC_Ageddown	CBC
1.75E-08	-0.2322663	0.904	0.969	0.000421	Gnb2	CBC_Ageddown	CBC
1.84E-09	-0.2322816	0.326	0.536	4.42E-05	Arl3	CBC_Ageddown	CBC
8.37E-11	-0.2323417	0.291	0.518	2.01E-06	Ccdc47	CBC_Ageddown	CBC
4.95E-11	-0.2323554	0.249	0.468	1.19E-06	Kansl1	CBC_Ageddown	CBC
7.73E-10	-0.2336319	0.226	0.438	1.85E-05	Cenph	CBC_Ageddown	CBC
7.81E-09	-0.2343212	0.823	0.946	0.000187	Cox7a2l	CBC_Ageddown	CBC
1.91E-08	-0.2345767	0.591	0.799	0.000459	Prkar1a	CBC_Ageddown	CBC
1.65E-10	-0.2347283	0.17	0.359	3.97E-06	Hk2	CBC_Ageddown	CBC
1.78E-08	-0.2349345	0.464	0.673	0.000427	Larp4b	CBC_Ageddown	CBC
1.70E-06	-0.2352141	0.596	0.789	0.040655	AY036118	CBC_Ageddown	CBC
3.91E-08	-0.2355537	0.409	0.621	0.000939	Knop1	CBC_Ageddown	CBC
6.91E-08	-0.2361522	0.794	0.924	0.001656	Laptm4a	CBC_Ageddown	CBC
4.42E-07	-0.2361802	0.462	0.654	0.010607	Nudcd2	CBC_Ageddown	CBC

1.01E-08	-0.2364233	0.589	0.791	0.000242	Dnaja2	CBC_Ageddown	CBC
3.31E-08	-0.2369088	0.689	0.843	0.000795	Ncor1	CBC_Ageddown	CBC
2.57E-08	-0.2370069	0.632	0.811	0.000617	Thoc7	CBC_Ageddown	CBC
4.70E-08	-0.2371737	0.577	0.76	0.001126	Ssrp1	CBC_Ageddown	CBC
5.46E-11	-0.2371797	0.215	0.431	1.31E-06	Dars	CBC_Ageddown	CBC
7.83E-08	-0.2375561	0.396	0.591	0.001878	Elf2	CBC_Ageddown	CBC
1.77E-09	-0.2378347	0.181	0.366	4.25E-05	Gm12446	CBC_Ageddown	CBC
1.07E-07	-0.2386851	0.581	0.758	0.002576	Psmd4	CBC_Ageddown	CBC
1.67E-12	-0.2400999	0.138	0.34	4.01E-08	Etv5	CBC_Ageddown	CBC
4.10E-10	-0.2404957	0.119	0.285	9.84E-06	Cox6b2	CBC_Ageddown	CBC
1.61E-07	-0.2405162	0.547	0.725	0.003867	Rsrc2	CBC_Ageddown	CBC
1.14E-08	-0.2410469	0.423	0.632	0.000273	N4bp2l2	CBC_Ageddown	CBC
1.98E-08	-0.2410814	0.683	0.85	0.000474	Ube2b	CBC_Ageddown	CBC
1.27E-08	-0.2412277	0.679	0.813	0.000305	Chd4	CBC_Ageddown	CBC
4.47E-09	-0.2415769	0.47	0.678	0.000107	Fxr1	CBC_Ageddown	CBC
5.04E-08	-0.2415795	0.915	0.957	0.001209	Ywhaz	CBC_Ageddown	CBC
1.69E-11	-0.2424087	0.126	0.311	4.06E-07	Coq10b	CBC_Ageddown	CBC
1.08E-08	-0.242636	0.57	0.762	0.000259	Ccar1	CBC_Ageddown	CBC
1.25E-07	-0.2429549	0.753	0.869	0.003006	Actn4	CBC_Ageddown	CBC
1.85E-09	-0.243204	0.921	0.985	4.44E-05	Hsp90b1	CBC_Ageddown	CBC
2.47E-08	-0.2435059	0.777	0.906	0.000592	Slc38a2	CBC_Ageddown	CBC
5.00E-11	-0.2438625	0.277	0.51	1.20E-06	Pnp	CBC_Ageddown	CBC
1.51E-08	-0.2439476	0.619	0.797	0.000362	1110004F10Rik	CBC_Ageddown	CBC
2.38E-08	-0.2441619	0.751	0.872	0.000571	Cct2	CBC_Ageddown	CBC
2.92E-07	-0.2442429	0.543	0.721	0.007007	Dnajc8	CBC_Ageddown	CBC
1.87E-07	-0.2445423	0.306	0.482	0.004474	Kitl	CBC_Ageddown	CBC
5.04E-08	-0.2447239	0.519	0.712	0.00121	Smchd1	CBC_Ageddown	CBC
7.58E-10	-0.2450389	0.374	0.593	1.82E-05	Ube2e3	CBC_Ageddown	CBC
2.85E-10	-0.2451263	0.349	0.591	6.84E-06	Ajuba	CBC_Ageddown	CBC
8.58E-10	-0.245211	0.321	0.538	2.06E-05	Arap2	CBC_Ageddown	CBC
8.98E-10	-0.2457876	0.255	0.455	2.15E-05	Serf1	CBC_Ageddown	CBC
1.11E-07	-0.2466136	0.672	0.841	0.002664	Luc7l3	CBC_Ageddown	CBC
2.09E-10	-0.246971	0.398	0.636	5.02E-06	Pcmt1	CBC_Ageddown	CBC
8.91E-11	-0.2471474	0.451	0.695	2.14E-06	Prpf38b	CBC_Ageddown	CBC
7.79E-08	-0.2472866	0.755	0.902	0.001869	Vdac2	CBC_Ageddown	CBC
1.21E-09	-0.2475075	0.413	0.63	2.91E-05	Ube2e1	CBC_Ageddown	CBC
7.32E-08	-0.2482329	0.626	0.799	0.001755	Fkbp3	CBC_Ageddown	CBC
6.90E-11	-0.2489268	0.234	0.451	1.66E-06	Gsr	CBC_Ageddown	CBC
7.24E-10	-0.2489617	0.396	0.623	1.74E-05	Wac	CBC_Ageddown	CBC
9.72E-09	-0.2495916	0.6	0.78	0.000233	Nsmce4a	CBC_Ageddown	CBC
3.30E-11	-0.2496602	0.074	0.229	7.92E-07	Prr15	CBC_Ageddown	CBC
1.09E-07	-0.249673	0.26	0.431	0.002611	Mrps6	CBC_Ageddown	CBC
2.55E-09	-0.2497068	0.296	0.497	6.11E-05	2610001J05Rik	CBC_Ageddown	CBC
1.00E-06	-0.2497922	0.574	0.725	0.023993	Lockd	CBC_Ageddown	CBC
3.24E-08	-0.2499533	0.579	0.747	0.000777	Pcnp	CBC_Ageddown	CBC
1.67E-07	-0.2503068	0.485	0.645	0.004013	Aurkb	CBC_Ageddown	CBC
6.27E-10	-0.250363	0.379	0.588	1.50E-05	Ubn1	CBC_Ageddown	CBC
1.22E-08	-0.2505359	0.598	0.797	0.000293	Rbbp4	CBC_Ageddown	CBC
5.01E-13	-0.2507045	0.168	0.386	1.20E-08	Zfp871	CBC_Ageddown	CBC
1.14E-11	-0.250966	0.198	0.409	2.74E-07	Rprd1b	CBC_Ageddown	CBC
4.59E-10	-0.2514006	0.67	0.869	1.10E-05	Lsm2	CBC_Ageddown	CBC
2.70E-08	-0.2514752	0.519	0.699	0.000647	Psmd12	CBC_Ageddown	CBC
5.58E-12	-0.251631	0.251	0.482	1.34E-07	Wdr89	CBC_Ageddown	CBC
6.23E-10	-0.251704	0.387	0.606	1.49E-05	Pak1ip1	CBC_Ageddown	CBC
4.09E-14	-0.2518199	0.087	0.274	9.80E-10	Pbx3	CBC_Ageddown	CBC
1.88E-08	-0.2519043	0.413	0.608	0.000452	Wee1	CBC_Ageddown	CBC
5.19E-10	-0.251933	0.266	0.475	1.24E-05	Tcf7l2	CBC_Ageddown	CBC
7.67E-10	-0.2519828	0.938	0.978	1.84E-05	Mrfap1	CBC_Ageddown	CBC
1.44E-07	-0.2520512	0.768	0.874	0.003445	Rad21	CBC_Ageddown	CBC

4.36E-07	-0.2520732	0.785	0.885	0.010464	Smarca5	CBC_Ageddown	CBC
1.63E-09	-0.2524391	0.213	0.397	3.91E-05	Ccdc88a	CBC_Ageddown	CBC
5.67E-09	-0.253318	0.706	0.856	0.000136	Eif3m	CBC_Ageddown	CBC
6.23E-12	-0.2533648	0.1	0.277	1.50E-07	Sertad1	CBC_Ageddown	CBC
1.84E-09	-0.2538412	0.36	0.569	4.41E-05	Atp2b1	CBC_Ageddown	CBC
1.19E-11	-0.2539244	0.26	0.503	2.85E-07	Sms	CBC_Ageddown	CBC
4.62E-08	-0.2539295	0.751	0.887	0.001109	Ssb	CBC_Ageddown	CBC
3.19E-10	-0.2543046	0.67	0.86	7.65E-06	Spcs2	CBC_Ageddown	CBC
1.25E-08	-0.2549991	0.474	0.652	0.000299	Bptf	CBC_Ageddown	CBC
4.96E-08	-0.2550073	0.96	0.98	0.001188	Ddx5	CBC_Ageddown	CBC
1.09E-09	-0.2553728	0.366	0.591	2.60E-05	Dnajc21	CBC_Ageddown	CBC
3.55E-08	-0.256391	0.504	0.684	0.000852	Tns4	CBC_Ageddown	CBC
1.66E-11	-0.2570597	0.4	0.643	3.99E-07	Actr10	CBC_Ageddown	CBC
1.43E-15	-0.2581567	0.051	0.235	3.44E-11	Hist1h2ab	CBC_Ageddown	CBC
2.16E-08	-0.2582664	0.4	0.595	0.000517	Map4k4	CBC_Ageddown	CBC
2.38E-11	-0.2589155	0.296	0.53	5.70E-07	Asf1b	CBC_Ageddown	CBC
1.30E-10	-0.2592861	0.343	0.573	3.11E-06	Shcbp1	CBC_Ageddown	CBC
1.42E-08	-0.2593711	0.621	0.782	0.000342	Morf4l2	CBC_Ageddown	CBC
4.06E-11	-0.259544	0.496	0.75	9.75E-07	Brd2	CBC_Ageddown	CBC
2.46E-11	-0.2608492	0.313	0.54	5.90E-07	Bcl10	CBC_Ageddown	CBC
1.70E-08	-0.2609118	0.63	0.83	0.000408	Prmt1	CBC_Ageddown	CBC
2.85E-08	-0.2610201	0.726	0.872	0.000684	Anp32e	CBC_Ageddown	CBC
5.09E-12	-0.262268	0.921	0.989	1.22E-07	Hnrnpk	CBC_Ageddown	CBC
1.75E-10	-0.2627198	0.398	0.645	4.20E-06	Tmem165	CBC_Ageddown	CBC
1.99E-07	-0.2628934	0.596	0.758	0.004774	Klf13	CBC_Ageddown	CBC
2.90E-08	-0.2634838	0.562	0.741	0.000696	Dazap1	CBC_Ageddown	CBC
8.00E-11	-0.2638489	0.377	0.597	1.92E-06	Rap1a	CBC_Ageddown	CBC
4.09E-09	-0.2648651	0.485	0.688	9.81E-05	Klf3	CBC_Ageddown	CBC
3.39E-16	-0.2654592	0.068	0.268	8.13E-12	Six1	CBC_Ageddown	CBC
7.47E-09	-0.267535	0.328	0.525	0.000179	Irf2bpl	CBC_Ageddown	CBC
1.41E-08	-0.2677974	0.379	0.571	0.000337	Hirip3	CBC_Ageddown	CBC
3.49E-12	-0.2683555	0.209	0.427	8.38E-08	Itpa	CBC_Ageddown	CBC
1.07E-11	-0.2684569	0.221	0.429	2.57E-07	Rad51ap1	CBC_Ageddown	CBC
4.79E-08	-0.2687385	0.632	0.808	0.001149	Aimp1	CBC_Ageddown	CBC
3.44E-10	-0.2690075	0.534	0.741	8.26E-06	Mtpn	CBC_Ageddown	CBC
1.67E-09	-0.2694088	0.596	0.815	4.01E-05	Rer1	CBC_Ageddown	CBC
6.66E-11	-0.2696443	0.409	0.638	1.60E-06	Atxn10	CBC_Ageddown	CBC
3.95E-09	-0.2702955	0.479	0.675	9.47E-05	Ahsa1	CBC_Ageddown	CBC
1.97E-08	-0.2703559	0.315	0.494	0.000472	Pdgfa	CBC_Ageddown	CBC
5.90E-10	-0.2707601	0.038	0.157	1.41E-05	Basp1	CBC_Ageddown	CBC
1.93E-10	-0.2708948	0.404	0.614	4.63E-06	Anxa7	CBC_Ageddown	CBC
1.13E-09	-0.271017	0.796	0.902	2.71E-05	Prrc2c	CBC_Ageddown	CBC
8.64E-24	-0.2711066	0.998	1	2.07E-19	Rpl21	CBC_Ageddown	CBC
4.13E-10	-0.2711124	0.196	0.384	9.92E-06	Rhob	CBC_Ageddown	CBC
6.52E-09	-0.2711501	0.483	0.673	0.000156	Ilkap	CBC_Ageddown	CBC
2.64E-08	-0.2721587	0.279	0.462	0.000634	Aqp3	CBC_Ageddown	CBC
5.89E-13	-0.2723723	0.287	0.54	1.41E-08	Polr2h	CBC_Ageddown	CBC
1.07E-25	-0.2727575	1	1	2.57E-21	mt-Cytb	CBC_Ageddown	CBC
1.51E-09	-0.2730428	0.689	0.856	3.62E-05	Zfp91	CBC_Ageddown	CBC
4.05E-11	-0.2740881	0.911	0.972	9.71E-07	Gnas	CBC_Ageddown	CBC
3.51E-09	-0.2745124	0.611	0.793	8.42E-05	Tra2a	CBC_Ageddown	CBC
3.52E-16	-0.2746295	0.189	0.457	8.43E-12	Arhgap10	CBC_Ageddown	CBC
1.13E-14	-0.2752665	1	1	2.70E-10	H3f3b	CBC_Ageddown	CBC
2.17E-19	-0.2753558	0.994	1	5.19E-15	Cox4i1	CBC_Ageddown	CBC
4.38E-10	-0.2772034	0.6	0.799	1.05E-05	Ppig	CBC_Ageddown	CBC
4.99E-12	-0.2774351	0.302	0.545	1.20E-07	Zcchc17	CBC_Ageddown	CBC
2.93E-10	-0.278308	0.432	0.669	7.04E-06	Hspb8	CBC_Ageddown	CBC
2.11E-12	-0.2789976	0.26	0.495	5.06E-08	Tgif1	CBC_Ageddown	CBC
4.34E-10	-0.2791802	0.662	0.837	1.04E-05	Rwdd1	CBC_Ageddown	CBC

1.89E-10	-0.2805796	0.515	0.725	4.54E-06	Acp1	CBC_Ageddown	CBC
1.94E-11	-0.2806208	0.413	0.645	4.66E-07	Svil	CBC_Ageddown	CBC
2.41E-13	-0.2825106	0.377	0.634	5.78E-09	Slc26a2	CBC_Ageddown	CBC
9.98E-11	-0.2825461	0.221	0.425	2.39E-06	Dlk2	CBC_Ageddown	CBC
3.07E-12	-0.2826447	0.181	0.392	7.37E-08	Frat2	CBC_Ageddown	CBC
7.37E-23	-0.2826875	0.019	0.231	1.77E-18	Ogfrl1	CBC_Ageddown	CBC
9.23E-09	-0.2827488	0.819	0.93	0.000221	Smc2	CBC_Ageddown	CBC
4.47E-13	-0.2829948	0.181	0.405	1.07E-08	Tbx3	CBC_Ageddown	CBC
5.67E-11	-0.283574	0.766	0.919	1.36E-06	2010111I01Rik	CBC_Ageddown	CBC
1.78E-13	-0.2840119	0.174	0.397	4.26E-09	Cyp2s1	CBC_Ageddown	CBC
2.49E-09	-0.284251	0.174	0.349	5.96E-05	Bhlhe40	CBC_Ageddown	CBC
1.21E-10	-0.2844171	0.445	0.664	2.89E-06	Nudt4	CBC_Ageddown	CBC
1.60E-12	-0.2846266	0.285	0.519	3.83E-08	Casz1	CBC_Ageddown	CBC
3.03E-08	-0.2848526	0.985	0.985	0.000728	Tubb5	CBC_Ageddown	CBC
4.79E-08	-0.2853717	0.736	0.854	0.00115	Hp1bp3	CBC_Ageddown	CBC
3.88E-11	-0.286188	0.147	0.331	9.30E-07	Cited2	CBC_Ageddown	CBC
3.12E-14	-0.2869824	0.106	0.311	7.48E-10	Kazald1	CBC_Ageddown	CBC
9.99E-11	-0.2873494	0.743	0.941	2.40E-06	Eif2s2	CBC_Ageddown	CBC
1.03E-13	-0.28736	0.287	0.532	2.46E-09	Rsl24d1	CBC_Ageddown	CBC
5.68E-08	-0.2880411	0.9	0.943	0.001361	Smc4	CBC_Ageddown	CBC
6.69E-17	-0.2880495	0.1	0.327	1.60E-12	Per3	CBC_Ageddown	CBC
4.11E-14	-0.2882285	0.955	0.996	9.87E-10	Srsf3	CBC_Ageddown	CBC
1.76E-10	-0.2883037	0.63	0.8	4.22E-06	Eif5b	CBC_Ageddown	CBC
2.58E-28	-0.2883718	1	1	6.18E-24	Rpl17	CBC_Ageddown	CBC
9.18E-11	-0.2884439	0.632	0.813	2.20E-06	Scp2	CBC_Ageddown	CBC
4.08E-14	-0.2888761	1	1	9.79E-10	H2afz	CBC_Ageddown	CBC
2.73E-10	-0.2893727	0.315	0.521	6.55E-06	Rps6ka3	CBC_Ageddown	CBC
3.41E-13	-0.2898643	0.951	0.991	8.19E-09	Snrpf	CBC_Ageddown	CBC
6.94E-11	-0.2913119	0.823	0.946	1.66E-06	Nedd4	CBC_Ageddown	CBC
4.03E-11	-0.2916127	0.457	0.699	9.67E-07	Senp6	CBC_Ageddown	CBC
5.91E-12	-0.2918447	0.226	0.449	1.42E-07	Mycl	CBC_Ageddown	CBC
3.38E-10	-0.2922596	0.579	0.797	8.11E-06	Psmc6	CBC_Ageddown	CBC
2.82E-13	-0.2926515	0.934	0.989	6.77E-09	Hnrnpf	CBC_Ageddown	CBC
5.53E-15	-0.294048	0.172	0.412	1.33E-10	Tpbg	CBC_Ageddown	CBC
6.42E-17	-0.2945281	0.138	0.381	1.54E-12	Banp	CBC_Ageddown	CBC
1.36E-11	-0.2951227	0.738	0.869	3.26E-07	Pbrm1	CBC_Ageddown	CBC
3.36E-10	-0.2951402	0.579	0.787	8.05E-06	Smc3	CBC_Ageddown	CBC
1.82E-11	-0.2954483	0.283	0.506	4.36E-07	Rab34	CBC_Ageddown	CBC
4.13E-07	-0.2955159	0.677	0.806	0.009902	Tpm1	CBC_Ageddown	CBC
1.42E-15	-0.2955962	0.34	0.619	3.40E-11	Prpf19	CBC_Ageddown	CBC
9.27E-12	-0.2959602	0.796	0.933	2.22E-07	G3bp1	CBC_Ageddown	CBC
6.59E-13	-0.2977569	0.421	0.671	1.58E-08	Cldnd1	CBC_Ageddown	CBC
1.66E-08	-0.2981973	0.702	0.865	0.000398	Dut	CBC_Ageddown	CBC
2.20E-14	-0.2983125	0.262	0.505	5.29E-10	Lamtor3	CBC_Ageddown	CBC
4.41E-09	-0.2991408	0.745	0.871	0.000106	Marcks	CBC_Ageddown	CBC
3.77E-11	-0.2991749	0.757	0.896	9.03E-07	Cct7	CBC_Ageddown	CBC
1.08E-14	-0.2998406	0.253	0.523	2.58E-10	Mpst	CBC_Ageddown	CBC
8.42E-11	-0.2998667	0.319	0.534	2.02E-06	Orc6	CBC_Ageddown	CBC
4.61E-14	-0.3004709	0.221	0.458	1.11E-09	Pdzd2	CBC_Ageddown	CBC
9.04E-12	-0.3004739	0.691	0.876	2.17E-07	Cct5	CBC_Ageddown	CBC
2.28E-10	-0.3007643	0.553	0.756	5.46E-06	Sri	CBC_Ageddown	CBC
2.06E-12	-0.3023653	0.609	0.823	4.94E-08	P4hb	CBC_Ageddown	CBC
3.89E-10	-0.3024978	0.438	0.647	9.32E-06	Bub1b	CBC_Ageddown	CBC
4.94E-11	-0.3027172	0.689	0.863	1.19E-06	Metap2	CBC_Ageddown	CBC
2.34E-11	-0.3029384	0.815	0.928	5.61E-07	Purb	CBC_Ageddown	CBC
1.10E-15	-0.3029636	0.168	0.407	2.64E-11	Atp8b1	CBC_Ageddown	CBC
3.84E-15	-0.3030166	0.966	0.991	9.20E-11	Sumo2	CBC_Ageddown	CBC
4.23E-08	-0.3034702	0.689	0.819	0.001015	Smc6	CBC_Ageddown	CBC
1.35E-09	-0.3036722	0.662	0.823	3.25E-05	Spc24	CBC_Ageddown	CBC

9.45E-14	-0.3051089	0.849	0.941	2.27E-09	Arpc2	CBC_Ageddown	CBC
6.27E-11	-0.3054158	0.823	0.933	1.50E-06	Cnbp	CBC_Ageddown	CBC
6.51E-37	-0.3054647	1	1	1.56E-32	Eef1a1	CBC_Ageddown	CBC
1.48E-12	-0.3067057	0.313	0.556	3.56E-08	Rgma	CBC_Ageddown	CBC
3.14E-12	-0.3077495	0.855	0.956	7.53E-08	Hdgf	CBC_Ageddown	CBC
2.94E-11	-0.3077962	0.496	0.702	7.05E-07	Smap1	CBC_Ageddown	CBC
2.48E-13	-0.3080244	0.451	0.708	5.94E-09	Strap	CBC_Ageddown	CBC
1.70E-08	-0.3113267	0.402	0.582	0.000408	Ncapd3	CBC_Ageddown	CBC
1.11E-10	-0.3126952	0.628	0.804	2.65E-06	Phip	CBC_Ageddown	CBC
1.68E-14	-0.3140349	0.298	0.553	4.02E-10	Dkc1	CBC_Ageddown	CBC
1.24E-12	-0.3152931	0.572	0.784	2.98E-08	Rp9	CBC_Ageddown	CBC
5.62E-17	-0.3156211	0.191	0.455	1.35E-12	Gid4	CBC_Ageddown	CBC
2.38E-12	-0.3162377	0.287	0.519	5.70E-08	Hlf	CBC_Ageddown	CBC
6.42E-10	-0.3165997	0.453	0.638	1.54E-05	Sgo2a	CBC_Ageddown	CBC
6.21E-20	-0.3174876	0.913	0.982	1.49E-15	Rbm39	CBC_Ageddown	CBC
1.07E-06	-0.319182	0.043	0.131	0.025779	Cxcl1	CBC_Ageddown	CBC
3.09E-15	-0.3195425	0.957	0.985	7.42E-11	Slc25a5	CBC_Ageddown	CBC
1.12E-12	-0.3203516	0.445	0.691	2.69E-08	Bnip2	CBC_Ageddown	CBC
1.63E-12	-0.320356	0.602	0.817	3.90E-08	Cct3	CBC_Ageddown	CBC
7.77E-14	-0.3205225	0.145	0.359	1.86E-09	Sema5a	CBC_Ageddown	CBC
3.53E-09	-0.3206979	0.419	0.601	8.45E-05	Glul	CBC_Ageddown	CBC
2.47E-19	-0.3208261	0.034	0.233	5.93E-15	Foxa2	CBC_Ageddown	CBC
2.02E-16	-0.3208942	0.111	0.333	4.84E-12	Ddit4	CBC_Ageddown	CBC
1.43E-41	-0.3210719	1	1	3.44E-37	Rps14	CBC_Ageddown	CBC
5.87E-10	-0.321468	0.477	0.682	1.41E-05	Nasp	CBC_Ageddown	CBC
1.14E-11	-0.3218644	0.477	0.704	2.73E-07	Nptn	CBC_Ageddown	CBC
5.65E-10	-0.3218741	0.564	0.745	1.35E-05	Btg1	CBC_Ageddown	CBC
2.29E-13	-0.3223387	0.287	0.538	5.50E-09	Zfhx3	CBC_Ageddown	CBC
1.26E-13	-0.3233694	0.121	0.323	3.03E-09	Hist1h3e	CBC_Ageddown	CBC
2.27E-10	-0.3239208	0.417	0.617	5.44E-06	Cd24a	CBC_Ageddown	CBC
2.00E-07	-0.3242591	0.277	0.433	0.0048	Hist1h4d	CBC_Ageddown	CBC
9.38E-17	-0.3245415	0.219	0.494	2.25E-12	Gnaq	CBC_Ageddown	CBC
1.02E-11	-0.3246429	0.734	0.896	2.45E-07	Magoh	CBC_Ageddown	CBC
1.34E-09	-0.3247117	0.406	0.619	3.21E-05	Xbp1	CBC_Ageddown	CBC
1.95E-12	-0.3251967	0.736	0.887	4.67E-08	Banf1	CBC_Ageddown	CBC
2.61E-13	-0.3265538	0.289	0.532	6.26E-09	Odc1	CBC_Ageddown	CBC
3.53E-17	-0.3266492	0.302	0.59	8.48E-13	Mettl23	CBC_Ageddown	CBC
4.06E-16	-0.3275494	0.274	0.553	9.74E-12	Mpp6	CBC_Ageddown	CBC
1.49E-09	-0.3292608	0.357	0.553	3.57E-05	Nfat5	CBC_Ageddown	CBC
3.29E-15	-0.3292776	0.449	0.717	7.90E-11	Pura	CBC_Ageddown	CBC
8.28E-13	-0.3299721	0.702	0.867	1.99E-08	Sf3b2	CBC_Ageddown	CBC
6.87E-08	-0.3306823	0.962	0.985	0.001648	Gsta4	CBC_Ageddown	CBC
1.03E-13	-0.3312574	0.364	0.616	2.47E-09	Cdc5l	CBC_Ageddown	CBC
3.23E-13	-0.3316114	0.713	0.878	7.74E-09	Cnih4	CBC_Ageddown	CBC
5.76E-12	-0.3317904	0.315	0.555	1.38E-07	Hat1	CBC_Ageddown	CBC
5.49E-12	-0.3327028	0.815	0.93	1.32E-07	Hspa5	CBC_Ageddown	CBC
8.44E-14	-0.3331404	0.749	0.902	2.02E-09	Sf3b1	CBC_Ageddown	CBC
9.99E-17	-0.3332874	0.138	0.377	2.39E-12	Frmd4a	CBC_Ageddown	CBC
7.15E-20	-0.3341622	0.97	0.991	1.71E-15	Serbp1	CBC_Ageddown	CBC
9.27E-07	-0.3345814	0.943	0.924	0.022237	Top2a	CBC_Ageddown	CBC
5.56E-18	-0.3370134	0.187	0.468	1.33E-13	Nkx2-3	CBC_Ageddown	CBC
2.57E-07	-0.3370725	0.97	0.993	0.006158	Malat1	CBC_Ageddown	CBC
8.52E-15	-0.3397723	0.279	0.543	2.04E-10	Crlf3	CBC_Ageddown	CBC
3.19E-15	-0.3404596	0.24	0.492	7.64E-11	Fbxo5	CBC_Ageddown	CBC
4.15E-17	-0.3404775	0.253	0.534	9.96E-13	Zfp503	CBC_Ageddown	CBC
1.95E-13	-0.3405602	0.56	0.76	4.67E-09	Ythdc1	CBC_Ageddown	CBC
9.06E-11	-0.3420935	0.626	0.811	2.17E-06	Hspa4l	CBC_Ageddown	CBC
9.66E-08	-0.3433841	0.119	0.251	0.002316	Nr4a1	CBC_Ageddown	CBC
2.02E-15	-0.343459	0.115	0.336	4.85E-11	Bdnf	CBC_Ageddown	CBC

7.43E-15	-0.3445179	0.828	0.963	1.78E-10	Sfr1	CBC_Ageddown	CBC
4.15E-14	-0.3454028	0.306	0.56	9.96E-10	Cdh13	CBC_Ageddown	CBC
3.50E-09	-0.3459302	0.585	0.749	8.39E-05	Ier2	CBC_Ageddown	CBC
2.97E-14	-0.3462217	0.568	0.817	7.12E-10	Acin1	CBC_Ageddown	CBC
1.57E-08	-0.3480908	0.455	0.656	0.000376	Esco2	CBC_Ageddown	CBC
6.85E-16	-0.3484974	0.289	0.562	1.64E-11	Ube2t	CBC_Ageddown	CBC
5.40E-14	-0.3488083	0.379	0.616	1.29E-09	Kmt2a	CBC_Ageddown	CBC
2.07E-09	-0.3488417	0.298	0.486	4.96E-05	Zfp36	CBC_Ageddown	CBC
1.05E-19	-0.3505912	0.964	0.989	2.51E-15	Calm1	CBC_Ageddown	CBC
5.70E-18	-0.3505967	0.168	0.44	1.37E-13	Rbms3	CBC_Ageddown	CBC
3.80E-47	-0.3507863	1	1	9.12E-43	Rplp2	CBC_Ageddown	CBC
5.21E-14	-0.3509427	0.685	0.876	1.25E-09	Arglu1	CBC_Ageddown	CBC
3.19E-31	-0.3517807	1	1	7.65E-27	Rpl12	CBC_Ageddown	CBC
1.99E-14	-0.3542182	0.489	0.726	4.76E-10	Aplp2	CBC_Ageddown	CBC
3.28E-19	-0.3556763	0.179	0.451	7.88E-15	Smad7	CBC_Ageddown	CBC
8.74E-12	-0.3557637	0.717	0.882	2.10E-07	Smc1a	CBC_Ageddown	CBC
9.99E-48	-0.3584498	1	1	2.40E-43	Rpl26	CBC_Ageddown	CBC
1.32E-13	-0.3606236	0.566	0.797	3.17E-09	Kmt5a	CBC_Ageddown	CBC
1.01E-08	-0.3607164	0.104	0.246	0.000241	Cebpd	CBC_Ageddown	CBC
1.50E-19	-0.3611573	0.966	0.991	3.60E-15	Set	CBC_Ageddown	CBC
4.75E-13	-0.3624797	0.853	0.954	1.14E-08	Hmgn2	CBC_Ageddown	CBC
2.19E-16	-0.3626147	0.594	0.841	5.26E-12	Vdac3	CBC_Ageddown	CBC
1.15E-12	-0.3627623	0.6	0.795	2.76E-08	Ankrd11	CBC_Ageddown	CBC
3.55E-09	-0.3637944	0.453	0.647	8.52E-05	Pnrc1	CBC_Ageddown	CBC
1.21E-11	-0.3645954	0.777	0.889	2.89E-07	Zfp36l2	CBC_Ageddown	CBC
2.88E-15	-0.3675204	0.345	0.617	6.91E-11	Chordc1	CBC_Ageddown	CBC
2.01E-10	-0.3676763	0.383	0.575	4.83E-06	Pmepa1	CBC_Ageddown	CBC
1.47E-13	-0.3681345	0.519	0.728	3.53E-09	Tmod3	CBC_Ageddown	CBC
5.06E-13	-0.3689793	0.364	0.61	1.21E-08	Chd3	CBC_Ageddown	CBC
9.07E-09	-0.369926	0.894	0.97	0.000218	Anxa1	CBC_Ageddown	CBC
2.68E-15	-0.3699377	0.785	0.932	6.42E-11	Eif3e	CBC_Ageddown	CBC
1.74E-17	-0.3716661	0.67	0.893	4.18E-13	Rbm8a	CBC_Ageddown	CBC
3.21E-09	-0.3719766	0.838	0.932	7.69E-05	Pclaf	CBC_Ageddown	CBC
5.90E-41	-0.3727064	1	1	1.41E-36	Rplp1	CBC_Ageddown	CBC
9.52E-21	-0.3781469	0.928	0.998	2.28E-16	Eif4a1	CBC_Ageddown	CBC
9.96E-21	-0.3788306	0.945	0.993	2.39E-16	Ywhae	CBC_Ageddown	CBC
1.11E-13	-0.3798653	0.402	0.651	2.66E-09	Dnajc9	CBC_Ageddown	CBC
1.09E-16	-0.38168	0.381	0.649	2.62E-12	Wbp4	CBC_Ageddown	CBC
1.30E-13	-0.3821481	0.664	0.865	3.12E-09	Tceal9	CBC_Ageddown	CBC
8.43E-14	-0.3821879	0.423	0.649	2.02E-09	Eid1	CBC_Ageddown	CBC
4.36E-26	-0.3825147	0.126	0.449	1.05E-21	Nr1d2	CBC_Ageddown	CBC
3.57E-12	-0.3834806	0.081	0.244	8.56E-08	Icam1	CBC_Ageddown	CBC
3.05E-15	-0.3843848	0.564	0.787	7.31E-11	Ctcf	CBC_Ageddown	CBC
2.00E-16	-0.3847455	0.738	0.908	4.81E-12	Tra2b	CBC_Ageddown	CBC
1.49E-10	-0.3857073	0.934	0.946	3.58E-06	Cks2	CBC_Ageddown	CBC
7.32E-14	-0.3857454	0.287	0.516	1.76E-09	Litaf	CBC_Ageddown	CBC
4.95E-18	-0.3863215	0.368	0.656	1.19E-13	Snrnp48	CBC_Ageddown	CBC
7.63E-14	-0.3892305	0.132	0.338	1.83E-09	Ppp1r15a	CBC_Ageddown	CBC
4.20E-19	-0.3921775	0.209	0.492	1.01E-14	Ppp1r14c	CBC_Ageddown	CBC
2.34E-15	-0.3958476	0.438	0.695	5.61E-11	Paics	CBC_Ageddown	CBC
5.46E-23	-0.3977585	0.851	0.97	1.31E-18	Rhoa	CBC_Ageddown	CBC
1.23E-18	-0.3982431	0.877	0.967	2.94E-14	Ptges3	CBC_Ageddown	CBC
2.88E-21	-0.3985878	0.343	0.66	6.90E-17	Med19	CBC_Ageddown	CBC
2.57E-16	-0.4003804	0.57	0.802	6.16E-12	Arf4	CBC_Ageddown	CBC
1.52E-20	-0.4005858	0.596	0.854	3.64E-16	Sbpl	CBC_Ageddown	CBC
1.57E-16	-0.4025257	0.168	0.409	3.76E-12	Klf9	CBC_Ageddown	CBC
1.52E-17	-0.4045855	0.315	0.588	3.64E-13	2900026A02Rik	CBC_Ageddown	CBC
7.77E-08	-0.4046766	0.791	0.86	0.001864	Ubc	CBC_Ageddown	CBC
2.15E-18	-0.4048041	0.691	0.9	5.15E-14	Srsf11	CBC_Ageddown	CBC

4.44E-17	-0.4054207	0.419	0.688	1.06E-12	Vezf1	CBC_Ageddown	CBC
9.12E-19	-0.406272	0.115	0.36	2.19E-14	Ifi202b	CBC_Ageddown	CBC
1.40E-16	-0.4072989	0.694	0.891	3.36E-12	Psma4	CBC_Ageddown	CBC
5.87E-17	-0.408139	0.613	0.845	1.41E-12	Pdia6	CBC_Ageddown	CBC
1.02E-19	-0.4082937	0.145	0.42	2.45E-15	Pdpn	CBC_Ageddown	CBC
7.11E-23	-0.4083828	0.751	0.921	1.70E-18	Pcbp1	CBC_Ageddown	CBC
4.27E-17	-0.4141271	0.402	0.682	1.02E-12	Emb	CBC_Ageddown	CBC
1.25E-17	-0.4144074	0.768	0.924	2.99E-13	U2af1	CBC_Ageddown	CBC
4.28E-12	-0.4160077	0.934	0.991	1.03E-07	Gsto1	CBC_Ageddown	CBC
1.93E-19	-0.4174528	0.745	0.917	4.62E-15	Top1	CBC_Ageddown	CBC
1.63E-22	-0.4217372	0.964	0.994	3.90E-18	Hspe1	CBC_Ageddown	CBC
2.95E-23	-0.4235251	0.055	0.303	7.07E-19	Lgr5	CBC_Ageddown	CBC
3.45E-26	-0.4263941	0.138	0.455	8.27E-22	Tef	CBC_Ageddown	CBC
1.13E-19	-0.4272355	0.628	0.867	2.70E-15	Psmd7	CBC_Ageddown	CBC
3.55E-19	-0.4278327	0.749	0.922	8.50E-15	Eif5	CBC_Ageddown	CBC
3.73E-19	-0.4282213	0.66	0.88	8.95E-15	Srrm1	CBC_Ageddown	CBC
5.50E-16	-0.4288679	0.513	0.758	1.32E-11	Hmgn5	CBC_Ageddown	CBC
1.11E-15	-0.4320244	0.702	0.882	2.66E-11	Sdc4	CBC_Ageddown	CBC
5.59E-26	-0.4323356	0.081	0.364	1.34E-21	Nr1d1	CBC_Ageddown	CBC
2.42E-20	-0.4347779	0.577	0.837	5.79E-16	Cbx1	CBC_Ageddown	CBC
6.38E-13	-0.4384921	0.226	0.438	1.53E-08	Lxn	CBC_Ageddown	CBC
3.67E-23	-0.4400433	0.938	0.989	8.80E-19	Anp32b	CBC_Ageddown	CBC
4.09E-25	-0.4426924	0.16	0.477	9.82E-21	Mns1	CBC_Ageddown	CBC
2.94E-20	-0.4428844	0.264	0.555	7.05E-16	Fzd1	CBC_Ageddown	CBC
5.85E-17	-0.444278	0.338	0.606	1.40E-12	Snai2	CBC_Ageddown	CBC
1.73E-28	-0.4447435	0.134	0.468	4.15E-24	Ahsa2	CBC_Ageddown	CBC
1.39E-58	-0.4456954	1	1	3.33E-54	mt-Co3	CBC_Ageddown	CBC
1.55E-17	-0.4469344	0.281	0.547	3.72E-13	Add3	CBC_Ageddown	CBC
3.72E-24	-0.4493155	0.832	0.97	8.92E-20	Srsf2	CBC_Ageddown	CBC
2.50E-23	-0.4506238	0.87	0.965	6.00E-19	Anxa5	CBC_Ageddown	CBC
4.35E-65	-0.4506747	1	1	1.04E-60	Rps21	CBC_Ageddown	CBC
5.18E-16	-0.4546331	0.187	0.429	1.24E-11	Socs3	CBC_Ageddown	CBC
2.43E-18	-0.4547309	0.421	0.706	5.84E-14	Arid5b	CBC_Ageddown	CBC
1.00E-23	-0.4591624	0.674	0.898	2.40E-19	Hnrnpc	CBC_Ageddown	CBC
5.72E-21	-0.4624579	0.674	0.878	1.37E-16	Cct4	CBC_Ageddown	CBC
9.59E-19	-0.4627409	0.43	0.688	2.30E-14	Cux1	CBC_Ageddown	CBC
6.27E-21	-0.4634268	0.794	0.948	1.50E-16	Hspd1	CBC_Ageddown	CBC
9.52E-12	-0.4666505	0.5	0.699	2.28E-07	Btg2	CBC_Ageddown	CBC
3.79E-10	-0.4671464	0.723	0.819	9.09E-06	Cltb	CBC_Ageddown	CBC
5.12E-23	-0.4673994	0.689	0.902	1.23E-18	Gnai2	CBC_Ageddown	CBC
9.74E-19	-0.4682563	0.489	0.774	2.34E-14	Psip1	CBC_Ageddown	CBC
1.44E-20	-0.4711177	0.545	0.815	3.46E-16	Dnajb1	CBC_Ageddown	CBC
6.19E-23	-0.4728996	0.402	0.719	1.48E-18	Auts2	CBC_Ageddown	CBC
3.98E-24	-0.4871185	0.551	0.828	9.54E-20	Stip1	CBC_Ageddown	CBC
3.95E-26	-0.4880025	0.911	0.982	9.47E-22	Hnrnpab	CBC_Ageddown	CBC
9.99E-25	-0.4880808	0.877	0.961	2.40E-20	Nap1l1	CBC_Ageddown	CBC
1.11E-33	-0.490922	0.902	0.982	2.67E-29	Pcbp2	CBC_Ageddown	CBC
7.23E-17	-0.4956811	0.2	0.449	1.73E-12	Foxq1	CBC_Ageddown	CBC
6.65E-24	-0.4982992	0.664	0.871	1.59E-19	St13	CBC_Ageddown	CBC
3.98E-17	-0.499835	0.17	0.427	9.55E-13	Phlda1	CBC_Ageddown	CBC
1.60E-29	-0.5010398	0.985	1	3.84E-25	Fxyd3	CBC_Ageddown	CBC
1.60E-19	-0.5016378	0.902	0.976	3.85E-15	Dek	CBC_Ageddown	CBC
4.56E-27	-0.5016947	0.649	0.895	1.09E-22	Pdap1	CBC_Ageddown	CBC
3.91E-30	-0.501843	0.249	0.617	9.37E-26	Hdac1	CBC_Ageddown	CBC
3.83E-41	-0.5024616	0.996	1	9.19E-37	Hmgb1	CBC_Ageddown	CBC
5.52E-20	-0.5055616	0.794	0.898	1.32E-15	Cdca8	CBC_Ageddown	CBC
2.12E-24	-0.5090542	0.683	0.891	5.09E-20	Lmnb1	CBC_Ageddown	CBC
3.49E-25	-0.5102433	0.911	0.983	8.36E-21	Nfib	CBC_Ageddown	CBC
4.93E-23	-0.5115084	0.879	0.961	1.18E-18	Nfix	CBC_Ageddown	CBC

3.27E-13	-0.5124519	0.226	0.442	7.83E-09	Fosb	CBC_Ageddown	CBC
2.08E-27	-0.5154753	0.117	0.444	4.99E-23	Fbln2	CBC_Ageddown	CBC
3.44E-15	-0.5171234	0.326	0.588	8.24E-11	Htra1	CBC_Ageddown	CBC
1.81E-12	-0.5178316	0.151	0.348	4.35E-08	Cldn10	CBC_Ageddown	CBC
1.30E-20	-0.5188505	0.555	0.787	3.12E-16	Ezh2	CBC_Ageddown	CBC
5.92E-38	-0.5199156	0.981	1	1.42E-33	Cd9	CBC_Ageddown	CBC
1.27E-29	-0.5218738	0.381	0.725	3.04E-25	Tnrc6c	CBC_Ageddown	CBC
1.37E-25	-0.5250718	0.472	0.784	3.29E-21	Tnfaip8	CBC_Ageddown	CBC
7.02E-29	-0.5254253	0.849	0.963	1.68E-24	Hnrnpa1	CBC_Ageddown	CBC
1.94E-26	-0.531703	0.947	0.989	4.64E-22	Nucks1	CBC_Ageddown	CBC
4.07E-08	-0.5325598	0.043	0.146	0.000975	Sult1d1	CBC_Ageddown	CBC
2.96E-52	-0.5335985	1	1	7.10E-48	Rps25	CBC_Ageddown	CBC
6.35E-28	-0.5343599	0.555	0.834	1.52E-23	Spop	CBC_Ageddown	CBC
6.39E-09	-0.5357858	0.14	0.29	0.000153	Ces1f	CBC_Ageddown	CBC
1.25E-32	-0.5413996	0.849	0.978	3.01E-28	Hnrnpa0	CBC_Ageddown	CBC
9.00E-70	-0.5445416	1	1	2.16E-65	Ptma	CBC_Ageddown	CBC
1.42E-07	-0.5471523	0.132	0.261	0.003405	Sostdc1	CBC_Ageddown	CBC
2.50E-17	-0.5497455	0.643	0.848	5.98E-13	Sox2	CBC_Ageddown	CBC
6.34E-28	-0.5536259	0.096	0.41	1.52E-23	Runx1	CBC_Ageddown	CBC
1.02E-24	-0.5573112	0.153	0.462	2.44E-20	Epha7	CBC_Ageddown	CBC
5.73E-19	-0.5580402	0.113	0.353	1.37E-14	Il33	CBC_Ageddown	CBC
4.24E-60	-0.5583678	1	1	1.02E-55	mt-Nd2	CBC_Ageddown	CBC
8.74E-11	-0.5618164	0.468	0.662	2.10E-06	Klf6	CBC_Ageddown	CBC
3.45E-34	-0.5651951	0.998	1	8.27E-30	Rps15	CBC_Ageddown	CBC
2.23E-16	-0.5659855	0.217	0.462	5.35E-12	Ptch1	CBC_Ageddown	CBC
2.51E-23	-0.5691177	0.879	0.948	6.01E-19	MuClm	CBC_Ageddown	CBC
3.73E-32	-0.5726386	0.679	0.935	8.95E-28	Cacybp	CBC_Ageddown	CBC
6.55E-44	-0.5768599	0.966	0.996	1.57E-39	Ybx1	CBC_Ageddown	CBC
4.90E-19	-0.5807555	0.157	0.416	1.18E-14	Socs2	CBC_Ageddown	CBC
7.56E-33	-0.5854558	0.123	0.494	1.81E-28	Dbp	CBC_Ageddown	CBC
8.56E-27	-0.585644	0.16	0.486	2.05E-22	Pax1	CBC_Ageddown	CBC
4.88E-17	-0.5900071	0.336	0.579	1.17E-12	Efemp1	CBC_Ageddown	CBC
8.91E-106	-0.5905945	1	1	2.14E-101	Tpt1	CBC_Ageddown	CBC
1.30E-26	-0.5977142	0.853	0.961	3.11E-22	Skp1a	CBC_Ageddown	CBC
1.69E-28	-0.6001133	0.643	0.887	4.05E-24	Txnip	CBC_Ageddown	CBC
2.09E-40	-0.600592	0.994	1	5.00E-36	Stmn1	CBC_Ageddown	CBC
9.00E-36	-0.602111	0.626	0.908	2.16E-31	Nudc	CBC_Ageddown	CBC
1.33E-23	-0.605402	0.666	0.893	3.18E-19	Cebpb	CBC_Ageddown	CBC
4.61E-26	-0.606499	0.187	0.518	1.11E-21	Fam3c	CBC_Ageddown	CBC
3.32E-30	-0.618551	0.715	0.911	7.95E-26	Rdx	CBC_Ageddown	CBC
4.68E-29	-0.6199391	0.985	0.993	1.12E-24	Ubb	CBC_Ageddown	CBC
1.66E-31	-0.6225245	0.423	0.78	3.98E-27	Serpinh1	CBC_Ageddown	CBC
1.26E-34	-0.6268178	0.172	0.555	3.02E-30	Gadd45b	CBC_Ageddown	CBC
1.72E-26	-0.6315535	0.323	0.656	4.13E-22	Nfkbia	CBC_Ageddown	CBC
1.34E-20	-0.6419089	0.438	0.708	3.22E-16	Sox4	CBC_Ageddown	CBC
4.11E-23	-0.6426865	0.304	0.662	9.86E-19	Rbp1	CBC_Ageddown	CBC
1.83E-31	-0.6502374	0.689	0.904	4.40E-27	Golim4	CBC_Ageddown	CBC
1.35E-28	-0.6545763	0.385	0.71	3.25E-24	Tsc22d3	CBC_Ageddown	CBC
4.92E-35	-0.6546729	0.868	0.959	1.18E-30	Tmpo	CBC_Ageddown	CBC
4.63E-79	-0.6599428	0.989	1	1.11E-74	H3f3a	CBC_Ageddown	CBC
9.12E-40	-0.6667549	0.64	0.908	2.19E-35	Fkbp4	CBC_Ageddown	CBC
3.93E-17	-0.6674459	0.232	0.486	9.43E-13	Ier3	CBC_Ageddown	CBC
2.61E-17	-0.6739496	0.798	0.922	6.27E-13	Epcam	CBC_Ageddown	CBC
6.92E-30	-0.6741702	0.243	0.591	1.66E-25	Tshz2	CBC_Ageddown	CBC
2.32E-27	-0.6836549	0.57	0.845	5.57E-23	H1fx	CBC_Ageddown	CBC
2.88E-25	-0.6842109	0.098	0.384	6.90E-21	Sox9	CBC_Ageddown	CBC
7.30E-26	-0.700145	0.37	0.688	1.75E-21	Id2	CBC_Ageddown	CBC
1.01E-39	-0.7011252	0.83	0.963	2.41E-35	Ccdc34	CBC_Ageddown	CBC
2.11E-38	-0.705436	0.134	0.53	5.06E-34	Alcam	CBC_Ageddown	CBC

2.43E-54	-0.7268921	0.972	0.994	5.82E-50	Rpl23a	CBC_Ageddown	CBC
5.62E-24	-0.7305165	0.677	0.856	1.35E-19	Ccnd2	CBC_Ageddown	CBC
2.15E-37	-0.7610779	0.196	0.595	5.15E-33	Id4	CBC_Ageddown	CBC
3.20E-20	-0.7634141	0.113	0.359	7.67E-16	Fst	CBC_Ageddown	CBC
4.99E-22	-0.7660042	0.113	0.383	1.20E-17	Krt75	CBC_Ageddown	CBC
8.27E-137	-0.766451	1	1	1.98E-132	Rps8	CBC_Ageddown	CBC
6.40E-30	-0.7719094	0.219	0.556	1.53E-25	Gadd45g	CBC_Ageddown	CBC
3.02E-34	-0.7924344	0.296	0.669	7.25E-30	Foxe1	CBC_Ageddown	CBC
8.20E-18	-0.8021933	0.394	0.632	1.97E-13	Ces1d	CBC_Ageddown	CBC
4.68E-99	-0.8125007	1	1	1.12E-94	Rps12	CBC_Ageddown	CBC
2.70E-59	-0.8139151	0.302	0.834	6.47E-55	Tmem59	CBC_Ageddown	CBC
2.96E-21	-0.8204904	0.338	0.604	7.11E-17	Hes6	CBC_Ageddown	CBC
1.93E-57	-0.8346894	1	1	4.63E-53	Hmgb2	CBC_Ageddown	CBC
1.59E-47	-0.8645881	0.964	0.998	3.81E-43	Hspb1	CBC_Ageddown	CBC
9.11E-45	-0.8838674	0.583	0.889	2.18E-40	Anxa8	CBC_Ageddown	CBC
2.83E-53	-0.8848981	0.904	0.989	6.78E-49	Ptn	CBC_Ageddown	CBC
1.94E-18	-0.8993	0.1	0.331	4.65E-14	Wif1	CBC_Ageddown	CBC
1.41E-42	-0.9135754	0.513	0.845	3.38E-38	Lmo4	CBC_Ageddown	CBC
3.43E-113	-1.0394657	0.998	1	8.22E-109	Hspa8	CBC_Ageddown	CBC
6.12E-45	-1.046295	0.485	0.835	1.47E-40	Tcf4	CBC_Ageddown	CBC
2.35E-24	-1.0500728	0.721	0.878	5.63E-20	Adh7	CBC_Ageddown	CBC
1.18E-29	-1.0635621	0.243	0.573	2.83E-25	Serpine2	CBC_Ageddown	CBC
3.09E-134	-1.077655	0.998	1	7.42E-130	Hsp90ab1	CBC_Ageddown	CBC
1.77E-20	-1.0962406	0.409	0.649	4.24E-16	Egr1	CBC_Ageddown	CBC
2.16E-16	-1.1173256	0.411	0.64	5.17E-12	Fos	CBC_Ageddown	CBC
2.88E-82	-1.2432919	0.315	0.85	6.91E-78	Hsph1	CBC_Ageddown	CBC
1.58E-31	-1.2584271	0.498	0.799	3.78E-27	Junb	CBC_Ageddown	CBC
7.38E-26	-1.3572857	0.432	0.695	1.77E-21	Jun	CBC_Ageddown	CBC
1.05E-117	-1.4772463	0.764	0.993	2.52E-113	Dnaja1	CBC_Ageddown	CBC
2.87E-95	-1.483277	0.215	0.826	6.88E-91	Hspa1a	CBC_Ageddown	CBC
2.20E-73	-1.5867252	0.643	0.937	5.28E-69	Id3	CBC_Ageddown	CBC
1.26E-37	-1.628487	0.63	0.906	3.03E-33	Krt17	CBC_Ageddown	CBC
7.07E-83	-1.6423419	0.43	0.891	1.69E-78	Id1	CBC_Ageddown	CBC
1.10E-123	-1.6998142	0.591	0.98	2.63E-119	Hspa1b	CBC_Ageddown	CBC
2.42E-136	-1.7081445	0.96	1	5.80E-132	Hsp90aa1	CBC_Ageddown	CBC
1.87E-07	3.30995206	0.465	0	0.004488	Muc5b	EnC_Agedup	EnC
4.43E-07	2.13102974	0.442	0	0.010634	Xist	EnC_Agedup	EnC
3.37E-07	1.95010811	0.512	0.043	0.008084	1600014C10Rik	EnC_Agedup	EnC
1.11E-06	1.93974121	0.791	0.362	0.026603	Ifi27l2a	EnC_Agedup	EnC
4.84E-07	1.88526379	1	0.979	0.011616	Gm42418	EnC_Agedup	EnC
3.16E-08	1.45315829	0.512	0	0.000758	Gm10260	EnC_Agedup	EnC
3.84E-10	1.23270377	1	0.936	9.21E-06	Gm10076	EnC_Agedup	EnC
1.02E-06	0.97981386	0.837	0.426	0.024529	Ndufa3	EnC_Agedup	EnC
4.64E-07	0.84119847	1	1	0.011136	Rpl11	EnC_Agedup	EnC
6.50E-07	0.76807988	1	1	0.015578	mt-Nd4l	EnC_Agedup	EnC
1.74E-06	0.65257152	1	1	0.041624	Rpl37a	EnC_Agedup	EnC
9.75E-08	0.57393315	1	1	0.002339	Rpl38	EnC_Agedup	EnC
9.05E-07	0.42739614	1	1	0.021704	Rps29	EnC_Agedup	EnC
5.21E-09	-0.6580189	1	1	0.000125	mt-Nd2	EnC_Ageddown	EnC
7.51E-07	-0.7446597	0.977	1	0.018018	Itm2b	EnC_Ageddown	EnC
1.02E-07	-0.8011408	1	1	0.002445	Hspa8	EnC_Ageddown	EnC
1.36E-06	-1.059007	0.907	0.979	0.032602	Ddx5	EnC_Ageddown	EnC
1.02E-09	-1.1114652	1	0.979	2.44E-05	Hsp90ab1	EnC_Ageddown	EnC
3.42E-07	-1.152194	0.814	0.872	0.00821	Hsp90aa1	EnC_Ageddown	EnC
4.64E-07	-1.1738675	0.256	0.83	0.011134	Clic4	EnC_Ageddown	EnC
2.21E-07	-1.2493115	0.186	0.745	0.005289	Hspa1a	EnC_Ageddown	EnC
5.26E-07	-1.2622294	0.907	0.979	0.012609	Hspb1	EnC_Ageddown	EnC
2.28E-08	-1.2759327	0.395	0.83	0.000548	Hspa1b	EnC_Ageddown	EnC
1.46E-07	-1.3289059	0.186	0.723	0.003499	Ddit4	EnC_Ageddown	EnC

1.77E-07	-1.411071	0.674	0.979	0.004241	Vim	EnC_Ageddown	EnC
8.97E-07	-1.4493379	0.047	0.532	0.021522	Scarb1	EnC_Ageddown	EnC
3.76E-10	-1.4706718	0.279	0.872	9.03E-06	Tmem59	EnC_Ageddown	EnC
3.77E-08	-1.5253337	0.907	1	0.000905	Txnip	EnC_Ageddown	EnC
1.26E-07	-1.5401945	0.349	0.83	0.003015	Adgrf5	EnC_Ageddown	EnC
1.03E-06	-1.627627	0.14	0.681	0.024607	Ppp1r15a	EnC_Ageddown	EnC
1.35E-10	-1.6293949	0.698	0.979	3.23E-06	Dnaja1	EnC_Ageddown	EnC
6.47E-08	-1.694941	0.605	0.936	0.001551	Junb	EnC_Ageddown	EnC
2.40E-08	-1.7194303	0.465	0.936	0.000575	Nfkbia	EnC_Ageddown	EnC
9.56E-07	-1.7541947	0.116	0.596	0.022917	Gadd45g	EnC_Ageddown	EnC
7.47E-07	-1.8108734	0.465	0.83	0.017918	Sparcl1	EnC_Ageddown	EnC
3.30E-07	-1.9503161	0.372	0.809	0.007913	Ier3	EnC_Ageddown	EnC
1.09E-08	-2.0166559	0.279	0.851	0.000261	Cd200	EnC_Ageddown	EnC
1.20E-08	-2.0719223	0.558	0.936	0.000287	Tsc22d3	EnC_Ageddown	EnC
2.65E-07	-2.8090587	0.465	0.83	0.00636	Egr1	EnC_Ageddown	EnC
4.67E-50	3.44213798	0.43	0.131	1.12E-45	Lyz2	EpC_Agedup	EpC
1.43E-97	2.27277154	0.798	0.361	3.44E-93	Ifi27l2a	EpC_Agedup	EpC
5.24E-31	2.2100177	0.567	0.362	1.26E-26	Wfdc12	EpC_Agedup	EpC
3.57E-73	2.05289402	0.868	0.623	8.57E-69	Crip1	EpC_Agedup	EpC
1.04E-27	1.98650031	0.26	0.071	2.50E-23	Dcpp1	EpC_Agedup	EpC
1.80E-36	1.97190007	0.255	0.047	4.31E-32	Lcn2	EpC_Agedup	EpC
4.94E-87	1.75017729	0.926	0.798	1.19E-82	H2-K1	EpC_Agedup	EpC
4.96E-40	1.74091801	0.583	0.301	1.19E-35	Slpi	EpC_Agedup	EpC
2.53E-34	1.65691917	0.423	0.162	6.06E-30	Ccl6	EpC_Agedup	EpC
6.96E-69	1.62662171	0.931	0.912	1.67E-64	H2-D1	EpC_Agedup	EpC
1.47E-134	1.5286929	0.499	0.01	3.54E-130	Gm10260	EpC_Agedup	EpC
4.88E-68	1.50964984	0.82	0.607	1.17E-63	B2m	EpC_Agedup	EpC
7.88E-40	1.49148467	0.737	0.529	1.89E-35	Ly6a	EpC_Agedup	EpC
3.16E-28	1.48642815	0.341	0.131	7.59E-24	Gp2	EpC_Agedup	EpC
3.26E-42	1.48445577	0.572	0.27	7.82E-38	Pglyrp1	EpC_Agedup	EpC
1.81E-16	1.39726798	0.211	0.075	4.33E-12	Dcpp3	EpC_Agedup	EpC
3.56E-48	1.32445422	0.4	0.107	8.54E-44	Muc5b	EpC_Agedup	EpC
1.24E-10	1.25257735	0.585	0.534	2.97E-06	Mt4	EpC_Agedup	EpC
6.92E-22	1.23616918	0.747	0.648	1.66E-17	Nupr1	EpC_Agedup	EpC
6.84E-55	1.23222537	1	0.996	1.64E-50	Gm42418	EpC_Agedup	EpC
4.93E-16	1.21717351	0.128	0.028	1.18E-11	Cd74	EpC_Agedup	EpC
5.01E-50	1.21649565	0.862	0.674	1.20E-45	Tmem176b	EpC_Agedup	EpC
1.11E-55	1.2032429	0.926	0.757	2.67E-51	Ifitm3	EpC_Agedup	EpC
1.22E-74	1.17717258	0.948	0.881	2.93E-70	mt-Atp8	EpC_Agedup	EpC
1.14E-49	1.1461855	0.347	0.075	2.73E-45	Pigr	EpC_Agedup	EpC
1.06E-54	1.09230098	0.971	0.907	2.53E-50	Wfdc18	EpC_Agedup	EpC
1.37E-29	1.06027725	0.292	0.086	3.30E-25	Cxcl5	EpC_Agedup	EpC
6.91E-51	1.04659015	0.255	0.021	1.66E-46	Tff2	EpC_Agedup	EpC
6.43E-10	1.03105747	0.106	0.033	1.54E-05	Scgb3a1	EpC_Agedup	EpC
1.21E-34	1.00942238	0.786	0.623	2.90E-30	Tmem176a	EpC_Agedup	EpC
9.53E-53	1.00451424	0.211	0.003	2.29E-48	Xist	EpC_Agedup	EpC
4.19E-22	0.99193159	0.734	0.555	1.00E-17	Wfdc2	EpC_Agedup	EpC
2.49E-34	0.95743011	0.391	0.14	5.96E-30	Isg15	EpC_Agedup	EpC
4.23E-90	0.95742382	1	0.998	1.01E-85	mt-Nd4l	EpC_Agedup	EpC
1.47E-43	0.95680185	0.648	0.369	3.53E-39	S100a1	EpC_Agedup	EpC
9.67E-33	0.95614714	0.575	0.334	2.32E-28	Cyba	EpC_Agedup	EpC
1.27E-30	0.95473424	0.535	0.26	3.05E-26	Cxcl17	EpC_Agedup	EpC
2.86E-54	0.95161654	0.504	0.18	6.86E-50	Psmb8	EpC_Agedup	EpC
1.31E-27	0.922674	0.499	0.282	3.13E-23	Tppp3	EpC_Agedup	EpC
4.49E-27	0.90193049	0.717	0.507	1.08E-22	Cbr2	EpC_Agedup	EpC
5.58E-45	0.88034225	0.902	0.711	1.34E-40	Ly6e	EpC_Agedup	EpC
7.07E-19	0.82537358	0.199	0.061	1.69E-14	Wfdc3	EpC_Agedup	EpC
1.30E-19	0.81761154	0.147	0.029	3.11E-15	AW112010	EpC_Agedup	EpC
1.63E-70	0.80418603	0.995	0.984	3.92E-66	Gm10076	EpC_Agedup	EpC

3.02E-29	0.7709326	0.742	0.468	7.24E-25	Ltf	EpC_Agedup	EpC
8.87E-25	0.76031231	0.45	0.236	2.13E-20	Bst2	EpC_Agedup	EpC
4.34E-33	0.717315	0.739	0.457	1.04E-28	Krt8	EpC_Agedup	EpC
1.31E-16	0.71566945	0.325	0.163	3.15E-12	Aoc1	EpC_Agedup	EpC
1.73E-26	0.69522073	0.755	0.645	4.16E-22	Sec11c	EpC_Agedup	EpC
7.38E-17	0.68267332	0.632	0.521	1.77E-12	Mgst1	EpC_Agedup	EpC
2.78E-32	0.67490019	0.331	0.11	6.67E-28	Lgals3bp	EpC_Agedup	EpC
1.12E-27	0.66763626	0.97	0.952	2.68E-23	Dbi	EpC_Agedup	EpC
1.42E-26	0.65049879	0.887	0.819	3.40E-22	Ftl1	EpC_Agedup	EpC
2.07E-26	0.64933524	0.432	0.204	4.97E-22	Agr2	EpC_Agedup	EpC
4.15E-19	0.64168184	0.332	0.158	9.96E-15	Irf7	EpC_Agedup	EpC
5.02E-17	0.62181897	0.631	0.482	1.20E-12	Mfge8	EpC_Agedup	EpC
7.03E-33	0.62139594	0.73	0.581	1.69E-28	Rsrp1	EpC_Agedup	EpC
1.25E-19	0.61673357	0.627	0.433	3.00E-15	Cldn3	EpC_Agedup	EpC
4.05E-11	0.59496923	0.261	0.148	9.72E-07	Rnase1	EpC_Agedup	EpC
1.02E-16	0.59016429	0.39	0.239	2.44E-12	Fam46a	EpC_Agedup	EpC
2.13E-11	0.58521504	0.143	0.049	5.10E-07	Tmem213	EpC_Agedup	EpC
1.94E-25	0.57742791	0.705	0.612	4.66E-21	Psme2	EpC_Agedup	EpC
5.49E-20	0.57539521	0.454	0.274	1.32E-15	Isg20	EpC_Agedup	EpC
3.48E-52	0.56501727	0.297	0.038	8.34E-48	H2-Q7	EpC_Agedup	EpC
1.42E-24	0.5585347	0.184	0.037	3.40E-20	Wfdc15b	EpC_Agedup	EpC
4.55E-38	0.5562313	0.322	0.088	1.09E-33	Psmb9	EpC_Agedup	EpC
2.66E-13	0.53312729	0.777	0.747	6.39E-09	Tmsb10	EpC_Agedup	EpC
5.36E-25	0.5241204	0.852	0.75	1.28E-20	Psap	EpC_Agedup	EpC
7.99E-22	0.52327677	0.535	0.354	1.92E-17	Prr15l	EpC_Agedup	EpC
2.09E-14	0.51946979	0.17	0.059	5.02E-10	Alox15	EpC_Agedup	EpC
3.59E-26	0.51918694	0.304	0.104	8.61E-22	F5	EpC_Agedup	EpC
4.05E-38	0.51758917	0.973	0.957	9.70E-34	Spint2	EpC_Agedup	EpC
5.37E-19	0.5141635	0.681	0.575	1.29E-14	Psme1	EpC_Agedup	EpC
1.56E-43	0.51283171	0.312	0.065	3.74E-39	Csprs	EpC_Agedup	EpC
6.77E-20	0.51147778	0.543	0.351	1.62E-15	Plet1	EpC_Agedup	EpC
1.16E-19	0.50393535	0.622	0.48	2.78E-15	Shisa5	EpC_Agedup	EpC
8.78E-16	0.50214273	0.536	0.397	2.11E-11	Scd2	EpC_Agedup	EpC
9.65E-11	0.5003356	0.755	0.778	2.31E-06	Atp2a2	EpC_Agedup	EpC
2.57E-10	0.49618842	0.911	0.847	6.17E-06	S100a6	EpC_Agedup	EpC
1.59E-26	0.49190167	0.782	0.732	3.80E-22	Ndufa3	EpC_Agedup	EpC
3.06E-22	0.48930668	0.872	0.756	7.33E-18	Ifitm2	EpC_Agedup	EpC
5.23E-18	0.47906467	0.897	0.831	1.26E-13	Tagln2	EpC_Agedup	EpC
6.23E-26	0.47717635	0.425	0.215	1.49E-21	Gpcpd1	EpC_Agedup	EpC
2.58E-13	0.4622489	0.511	0.384	6.19E-09	Tcn2	EpC_Agedup	EpC
7.87E-16	0.46131844	0.894	0.896	1.89E-11	Crip2	EpC_Agedup	EpC
1.54E-16	0.45978301	0.46	0.294	3.70E-12	Mmp15	EpC_Agedup	EpC
1.54E-51	0.44914955	1	1	3.70E-47	mt-Co1	EpC_Agedup	EpC
3.05E-07	0.44845705	0.236	0.146	0.007313	Klf2	EpC_Agedup	EpC
2.55E-19	0.44617105	0.87	0.847	6.11E-15	Ndufc2	EpC_Agedup	EpC
2.66E-09	0.44286357	0.17	0.075	6.38E-05	Ifitm1	EpC_Agedup	EpC
5.70E-18	0.43864357	0.497	0.29	1.37E-13	Atp1b1	EpC_Agedup	EpC
5.52E-16	0.43538915	0.216	0.086	1.32E-11	Gm32219	EpC_Agedup	EpC
1.68E-18	0.43225865	0.786	0.736	4.02E-14	Ndufv3	EpC_Agedup	EpC
1.81E-06	0.43077289	0.585	0.484	0.043357	Krt7	EpC_Agedup	EpC
1.46E-14	0.42446754	0.336	0.185	3.51E-10	0610040J01Rik	EpC_Agedup	EpC
7.41E-27	0.423144	0.96	0.964	1.78E-22	Sec61g	EpC_Agedup	EpC
3.99E-20	0.42236623	0.304	0.133	9.58E-16	Samd9l	EpC_Agedup	EpC
7.69E-13	0.42231515	0.616	0.458	1.84E-08	Slc12a2	EpC_Agedup	EpC
1.38E-19	0.41508202	0.236	0.085	3.31E-15	Clic6	EpC_Agedup	EpC
3.79E-11	0.41450132	0.175	0.076	9.08E-07	1600014C10Rik	EpC_Agedup	EpC
2.43E-13	0.41262403	0.369	0.223	5.84E-09	Gm26532	EpC_Agedup	EpC
1.63E-16	0.41113867	0.752	0.701	3.90E-12	Nenf	EpC_Agedup	EpC
3.25E-13	0.4074945	0.435	0.299	7.79E-09	Rhoc	EpC_Agedup	EpC

1.30E-11	0.40669589	0.74	0.623	3.13E-07	Cldn7	EpC_Agedup	EpC
3.38E-13	0.40507591	0.477	0.342	8.10E-09	Dcxr	EpC_Agedup	EpC
3.30E-16	0.40196042	0.644	0.538	7.92E-12	1810022K09Rik	EpC_Agedup	EpC
1.16E-14	0.40097685	0.297	0.145	2.77E-10	Enpep	EpC_Agedup	EpC
1.90E-19	0.3996411	0.307	0.135	4.55E-15	Rtp4	EpC_Agedup	EpC
1.77E-19	0.39961259	0.877	0.911	4.25E-15	Ndufa11	EpC_Agedup	EpC
5.37E-16	0.39715332	0.444	0.277	1.29E-11	Pdlim3	EpC_Agedup	EpC
2.16E-17	0.39306769	0.872	0.844	5.18E-13	Tmbim6	EpC_Agedup	EpC
2.16E-15	0.39211759	0.452	0.298	5.18E-11	Tspan13	EpC_Agedup	EpC
7.47E-17	0.38976989	0.739	0.68	1.79E-12	Tmem256	EpC_Agedup	EpC
8.15E-10	0.38802268	0.526	0.43	1.95E-05	Txndc5	EpC_Agedup	EpC
5.35E-13	0.38773463	0.889	0.745	1.28E-08	Ecm1	EpC_Agedup	EpC
2.04E-12	0.38731879	0.231	0.107	4.89E-08	Crabp1	EpC_Agedup	EpC
7.74E-26	0.38621521	0.177	0.031	1.86E-21	Ifi203	EpC_Agedup	EpC
9.44E-15	0.38536596	0.976	0.982	2.26E-10	Fth1	EpC_Agedup	EpC
3.43E-17	0.38524747	0.82	0.829	8.22E-13	Romo1	EpC_Agedup	EpC
2.97E-14	0.38429998	0.769	0.732	7.13E-10	Krtcap2	EpC_Agedup	EpC
5.13E-15	0.38163693	0.718	0.552	1.23E-10	Clec2d	EpC_Agedup	EpC
1.89E-12	0.38104453	0.481	0.32	4.54E-08	Tm4sf1	EpC_Agedup	EpC
4.92E-12	0.38030239	0.327	0.19	1.18E-07	Kcne3	EpC_Agedup	EpC
3.64E-11	0.3765711	0.501	0.402	8.73E-07	Npdc1	EpC_Agedup	EpC
2.59E-18	0.37495353	0.359	0.183	6.22E-14	Apobec1	EpC_Agedup	EpC
5.18E-43	0.37231692	0.199	0.011	1.24E-38	H2-Q6	EpC_Agedup	EpC
4.60E-13	0.37057486	0.503	0.371	1.10E-08	Setd5	EpC_Agedup	EpC
6.97E-11	0.36746709	0.594	0.505	1.67E-06	Arpc1b	EpC_Agedup	EpC
2.77E-14	0.36621789	0.788	0.746	6.63E-10	Cd63	EpC_Agedup	EpC
1.12E-15	0.36512318	0.906	0.907	2.68E-11	Atp5g3	EpC_Agedup	EpC
5.83E-12	0.36200081	0.376	0.239	1.40E-07	Gstp1	EpC_Agedup	EpC
5.41E-11	0.36164298	0.506	0.342	1.30E-06	Msln	EpC_Agedup	EpC
5.61E-13	0.3558061	0.899	0.908	1.34E-08	Gpx4	EpC_Agedup	EpC
5.35E-09	0.3502411	0.59	0.514	0.000128	Lsm7	EpC_Agedup	EpC
2.68E-11	0.34560709	0.482	0.374	6.43E-07	Tmem160	EpC_Agedup	EpC
7.25E-10	0.34248881	0.703	0.677	1.74E-05	Cuta	EpC_Agedup	EpC
4.21E-11	0.34246051	0.636	0.585	1.01E-06	Dnajc19	EpC_Agedup	EpC
2.73E-12	0.34244412	0.325	0.185	6.54E-08	Oasl2	EpC_Agedup	EpC
3.49E-09	0.34233989	0.577	0.48	8.36E-05	Ece1	EpC_Agedup	EpC
1.21E-09	0.34183696	0.298	0.19	2.90E-05	Ano1	EpC_Agedup	EpC
7.58E-22	0.33818376	0.243	0.081	1.82E-17	H2-Q4	EpC_Agedup	EpC
3.10E-17	0.33504496	0.946	0.915	7.44E-13	Atp6v1g1	EpC_Agedup	EpC
8.09E-10	0.33366301	0.624	0.512	1.94E-05	Lrrc26	EpC_Agedup	EpC
6.66E-07	0.33330316	0.631	0.531	0.015964	Cldn10	EpC_Agedup	EpC
1.55E-07	0.33156338	0.594	0.567	0.003712	Hint2	EpC_Agedup	EpC
1.65E-09	0.3299461	0.545	0.463	3.95E-05	Mrps28	EpC_Agedup	EpC
3.27E-11	0.32941501	0.428	0.277	7.84E-07	Plcb4	EpC_Agedup	EpC
2.36E-07	0.32920952	0.562	0.479	0.005649	Elf3	EpC_Agedup	EpC
9.97E-18	0.32406504	0.194	0.062	2.39E-13	Zbp1	EpC_Agedup	EpC
1.63E-10	0.32282051	0.209	0.104	3.90E-06	Shisa2	EpC_Agedup	EpC
6.83E-12	0.32279555	0.855	0.868	1.64E-07	Ndufb8	EpC_Agedup	EpC
2.99E-09	0.32131952	0.43	0.323	7.17E-05	Itm2c	EpC_Agedup	EpC
2.51E-14	0.32092943	0.371	0.216	6.01E-10	Snhg20	EpC_Agedup	EpC
5.17E-08	0.31979009	0.594	0.534	0.001239	Erh	EpC_Agedup	EpC
1.13E-12	0.31915785	0.258	0.129	2.70E-08	Xaf1	EpC_Agedup	EpC
2.67E-08	0.31893819	0.686	0.669	0.000639	Snrpg	EpC_Agedup	EpC
2.03E-24	0.31821997	0.558	0.291	4.86E-20	Krt19	EpC_Agedup	EpC
1.30E-09	0.31764552	0.314	0.2	3.12E-05	Cnn3	EpC_Agedup	EpC
2.50E-13	0.31624908	0.847	0.837	6.00E-09	Ndufb7	EpC_Agedup	EpC
5.86E-09	0.31449089	0.401	0.297	0.000141	Pttg1	EpC_Agedup	EpC
1.12E-09	0.31262455	0.288	0.164	2.68E-05	Fcgbp	EpC_Agedup	EpC
4.58E-08	0.31024599	0.469	0.373	0.001097	Smim22	EpC_Agedup	EpC

1.54E-08	0.31020033	0.607	0.586	0.000369	Sap18	EpC_Agedup	EpC
3.83E-21	0.30921974	0.69	0.417	9.18E-17	Krt18	EpC_Agedup	EpC
1.08E-06	0.3089864	0.514	0.426	0.025948	Oat	EpC_Agedup	EpC
5.23E-11	0.30819988	0.336	0.204	1.26E-06	Mlph	EpC_Agedup	EpC
1.84E-07	0.30644738	0.415	0.32	0.004405	Atp11a	EpC_Agedup	EpC
8.81E-07	0.30618206	0.744	0.705	0.02113	Neat1	EpC_Agedup	EpC
8.30E-10	0.30601623	0.641	0.599	1.99E-05	Chchd1	EpC_Agedup	EpC
1.73E-06	0.30559361	0.663	0.649	0.041394	Ifi27	EpC_Agedup	EpC
6.62E-13	0.30550165	0.98	0.989	1.59E-08	Cox6b1	EpC_Agedup	EpC
6.99E-09	0.30227478	0.516	0.409	0.000168	Krtcap3	EpC_Agedup	EpC
9.66E-10	0.30219898	0.723	0.696	2.32E-05	Ndufs8	EpC_Agedup	EpC
6.01E-09	0.30206274	0.717	0.721	0.000144	Tmem258	EpC_Agedup	EpC
1.22E-09	0.30016345	0.823	0.857	2.93E-05	Ndufa6	EpC_Agedup	EpC
7.70E-14	0.29920453	0.922	0.939	1.85E-09	Selenow	EpC_Agedup	EpC
4.66E-07	0.29543822	0.524	0.462	0.01118	Azin1	EpC_Agedup	EpC
3.11E-12	0.29522121	0.968	0.974	7.47E-08	Atox1	EpC_Agedup	EpC
8.28E-10	0.29363492	0.863	0.883	1.99E-05	Rabac1	EpC_Agedup	EpC
8.01E-14	0.29283749	0.194	0.076	1.92E-09	Adgre5	EpC_Agedup	EpC
5.53E-07	0.29124908	0.403	0.305	0.013253	Flna	EpC_Agedup	EpC
1.26E-07	0.28886239	0.393	0.293	0.003027	Cd82	EpC_Agedup	EpC
5.35E-11	0.28871431	0.27	0.153	1.28E-06	Tap1	EpC_Agedup	EpC
3.39E-09	0.28824054	0.931	0.894	8.13E-05	Atp1a1	EpC_Agedup	EpC
4.14E-10	0.28801534	0.322	0.187	9.93E-06	Prom1	EpC_Agedup	EpC
3.63E-12	0.2868788	0.863	0.896	8.71E-08	Dad1	EpC_Agedup	EpC
9.37E-13	0.2866803	0.936	0.951	2.25E-08	Sec61b	EpC_Agedup	EpC
3.08E-08	0.28653086	0.944	0.96	0.000738	Cox5a	EpC_Agedup	EpC
3.50E-08	0.2858796	0.283	0.182	0.000839	Stat1	EpC_Agedup	EpC
1.64E-13	0.28497347	0.939	0.956	3.94E-09	Atp5g1	EpC_Agedup	EpC
6.68E-07	0.28145993	0.361	0.272	0.016021	Ifi35	EpC_Agedup	EpC
1.66E-15	0.28084823	0.993	0.995	3.99E-11	Rpl38	EpC_Agedup	EpC
1.76E-13	0.27929939	0.958	0.966	4.21E-09	Uqcr11	EpC_Agedup	EpC
1.89E-09	0.27595188	0.764	0.76	4.52E-05	Fkbp2	EpC_Agedup	EpC
5.40E-09	0.27317659	0.669	0.63	0.000129	Eny2	EpC_Agedup	EpC
1.12E-07	0.27292895	0.793	0.814	0.002696	Nop10	EpC_Agedup	EpC
5.05E-07	0.27288988	0.499	0.442	0.012102	Pigyl	EpC_Agedup	EpC
7.90E-08	0.27255319	0.843	0.873	0.001895	Ost4	EpC_Agedup	EpC
1.37E-15	0.27180048	0.987	0.992	3.29E-11	Oaz1	EpC_Agedup	EpC
2.55E-13	0.27088848	0.204	0.086	6.12E-09	Tex15	EpC_Agedup	EpC
2.24E-10	0.26900646	0.86	0.866	5.37E-06	D8Ertd738e	EpC_Agedup	EpC
1.88E-16	0.26853403	0.207	0.074	4.51E-12	Adat2	EpC_Agedup	EpC
4.30E-09	0.26806734	0.806	0.813	0.000103	Ndufa1	EpC_Agedup	EpC
6.32E-09	0.2675646	0.433	0.308	0.000152	Car12	EpC_Agedup	EpC
3.07E-07	0.2675039	0.105	0.042	0.007363	Alox12e	EpC_Agedup	EpC
1.58E-06	0.26572901	0.354	0.266	0.037925	Npc1	EpC_Agedup	EpC
1.05E-10	0.26558371	0.98	0.986	2.53E-06	Pfn1	EpC_Agedup	EpC
2.75E-10	0.26521803	0.268	0.149	6.60E-06	Sp100	EpC_Agedup	EpC
2.31E-11	0.2651113	0.233	0.112	5.53E-07	Cldn2	EpC_Agedup	EpC
1.43E-07	0.26313537	0.683	0.651	0.003422	Sdcbp	EpC_Agedup	EpC
7.11E-09	0.26169863	0.3	0.19	0.00017	Rhoq	EpC_Agedup	EpC
3.80E-09	0.25920342	0.497	0.355	9.10E-05	Kctd14	EpC_Agedup	EpC
4.19E-09	0.2584989	0.445	0.323	0.000101	Sulf2	EpC_Agedup	EpC
2.62E-10	0.2579693	0.921	0.938	6.27E-06	Uqcrq	EpC_Agedup	EpC
6.92E-07	0.2542812	0.874	0.9	0.016603	Atp5k	EpC_Agedup	EpC
9.06E-07	0.25103013	0.642	0.615	0.021734	Churc1	EpC_Agedup	EpC
7.02E-10	0.25078996	0.197	0.097	1.68E-05	Lbp	EpC_Agedup	EpC
1.54E-09	0.25062074	0.273	0.157	3.70E-05	Enpp3	EpC_Agedup	EpC
2.54E-13	0.2498498	0.207	0.088	6.09E-09	Ocel1	EpC_Agedup	EpC
6.15E-11	0.24970989	0.219	0.106	1.47E-06	Parp14	EpC_Agedup	EpC
1.26E-06	0.24830291	0.759	0.761	0.030206	Cope	EpC_Agedup	EpC

5.75E-07	0.24760337	0.823	0.853	0.013793	Swi5	EpC_Agedup	EpC
2.22E-07	0.24714892	0.174	0.09	0.005315	Lgals1	EpC_Agedup	EpC
1.94E-08	0.24690209	0.287	0.176	0.000465	Irgm1	EpC_Agedup	EpC
3.01E-10	0.24533573	0.927	0.957	7.22E-06	Rps27l	EpC_Agedup	EpC
1.61E-09	0.24196953	0.998	0.998	3.86E-05	Rps28	EpC_Agedup	EpC
2.39E-08	0.2407115	0.245	0.144	0.000573	Slc28a3	EpC_Agedup	EpC
9.86E-08	0.23998098	0.836	0.858	0.002364	Usmg5	EpC_Agedup	EpC
5.74E-08	0.23611943	0.309	0.193	0.001378	Myo1b	EpC_Agedup	EpC
1.30E-09	0.23415997	0.971	0.971	3.12E-05	Cox7a2	EpC_Agedup	EpC
1.46E-23	0.23404333	0.18	0.037	3.51E-19	Gm8797	EpC_Agedup	EpC
7.28E-09	0.23311377	0.853	0.868	0.000175	Selenok	EpC_Agedup	EpC
1.63E-06	0.23175243	0.705	0.633	0.039017	Cat	EpC_Agedup	EpC
8.84E-09	0.23172444	0.283	0.17	0.000212	Ppp1r1b	EpC_Agedup	EpC
5.02E-08	0.23042033	0.847	0.878	0.001203	Ndufa2	EpC_Agedup	EpC
4.98E-10	0.22999586	0.966	0.991	1.20E-05	Atp5g2	EpC_Agedup	EpC
3.73E-11	0.22881825	0.199	0.091	8.94E-07	Kcnk5	EpC_Agedup	EpC
1.69E-09	0.22786456	0.909	0.928	4.05E-05	Ndufa7	EpC_Agedup	EpC
1.70E-08	0.22311222	0.953	0.975	0.000409	Elob	EpC_Agedup	EpC
8.53E-08	0.21926802	0.924	0.948	0.002045	Uqcr10	EpC_Agedup	EpC
1.39E-27	0.21748011	0.126	0.007	3.33E-23	AC168977.2	EpC_Agedup	EpC
1.59E-25	0.21320409	0.121	0.007	3.82E-21	Gm11361	EpC_Agedup	EpC
1.15E-06	0.21206253	0.275	0.184	0.027678	Cdc42ep4	EpC_Agedup	EpC
5.59E-08	0.21162132	0.147	0.068	0.00134	Ifi44	EpC_Agedup	EpC
3.55E-08	0.20985405	0.177	0.088	0.000852	Ifit1	EpC_Agedup	EpC
1.18E-06	0.2068406	0.865	0.901	0.028367	2410015M20Rik	EpC_Agedup	EpC
1.33E-07	0.20672848	0.895	0.928	0.003185	Ssr4	EpC_Agedup	EpC
1.15E-06	0.20206632	0.833	0.876	0.027683	Ndufs5	EpC_Agedup	EpC
4.22E-11	0.20155827	0.985	0.998	1.01E-06	Serf2	EpC_Agedup	EpC
8.13E-08	0.20103663	0.899	0.941	0.00195	Ndufa13	EpC_Agedup	EpC
8.84E-10	-0.2004171	0.408	0.601	2.12E-05	Eif3d	EpC_Ageddown	EpC
5.91E-10	-0.2010231	0.331	0.505	1.42E-05	Ccar1	EpC_Ageddown	EpC
2.18E-15	-0.2011702	0.113	0.29	5.23E-11	Cpox	EpC_Ageddown	EpC
1.41E-09	-0.2014692	0.219	0.37	3.38E-05	Mboat1	EpC_Ageddown	EpC
1.08E-09	-0.202172	0.642	0.793	2.58E-05	Gnai2	EpC_Ageddown	EpC
1.02E-09	-0.2023237	0.368	0.556	2.45E-05	Cul1	EpC_Ageddown	EpC
6.65E-07	-0.2029826	0.698	0.802	0.015941	Nfix	EpC_Ageddown	EpC
9.74E-09	-0.2030573	0.553	0.713	0.000234	Glo1	EpC_Ageddown	EpC
1.24E-13	-0.2031794	0.165	0.35	2.97E-09	Rab38	EpC_Ageddown	EpC
7.06E-13	-0.2033018	0.197	0.372	1.69E-08	2310033P09Rik	EpC_Ageddown	EpC
9.33E-10	-0.2041409	0.538	0.734	2.24E-05	Map2k2	EpC_Ageddown	EpC
3.95E-13	-0.2041507	0.273	0.471	9.47E-09	Tmem165	EpC_Ageddown	EpC
2.15E-08	-0.2041781	0.568	0.715	0.000515	Mtdh	EpC_Ageddown	EpC
1.30E-12	-0.2042825	0.302	0.51	3.12E-08	Vamp3	EpC_Ageddown	EpC
2.68E-08	-0.2046443	0.42	0.584	0.000642	Hmgn3	EpC_Ageddown	EpC
6.68E-11	-0.2046828	0.167	0.324	1.60E-06	Eid1	EpC_Ageddown	EpC
3.48E-09	-0.2048096	0.465	0.615	8.34E-05	Arhgef12	EpC_Ageddown	EpC
5.40E-11	-0.2050334	0.098	0.232	1.29E-06	Ces2f	EpC_Ageddown	EpC
3.80E-11	-0.2054226	0.159	0.31	9.11E-07	Plek2	EpC_Ageddown	EpC
1.92E-12	-0.2059898	0.174	0.345	4.59E-08	Sertad1	EpC_Ageddown	EpC
5.43E-09	-0.2061165	0.818	0.908	0.00013	Csnk1a1	EpC_Ageddown	EpC
6.03E-14	-0.2061427	0.187	0.374	1.45E-09	Hsdl2	EpC_Ageddown	EpC
2.20E-08	-0.2063978	0.106	0.213	0.000528	Ddit4	EpC_Ageddown	EpC
2.63E-08	-0.2065753	0.563	0.711	0.000631	Ostf1	EpC_Ageddown	EpC
7.13E-07	-0.2066181	0.627	0.75	0.017109	Hnrnpa1	EpC_Ageddown	EpC
9.18E-12	-0.2079745	0.312	0.512	2.20E-07	Gadd45gip1	EpC_Ageddown	EpC
1.66E-13	-0.2083155	0.177	0.356	3.97E-09	Dnajb2	EpC_Ageddown	EpC
2.15E-11	-0.2083546	0.18	0.338	5.15E-07	Tmem159	EpC_Ageddown	EpC
8.46E-10	-0.2087398	0.476	0.665	2.03E-05	Smim7	EpC_Ageddown	EpC
1.06E-10	-0.2088131	0.245	0.417	2.54E-06	Abhd5	EpC_Ageddown	EpC

5.92E-10	-0.2088753	0.403	0.587	1.42E-05	Nectin1	EpC_Ageddown	EpC
3.31E-12	-0.2090647	0.995	0.997	7.95E-08	Rpl10	EpC_Ageddown	EpC
5.70E-13	-0.2095179	0.184	0.364	1.37E-08	Efnb1	EpC_Ageddown	EpC
3.44E-12	-0.2103495	0.251	0.441	8.26E-08	Epha4	EpC_Ageddown	EpC
1.68E-09	-0.2103729	0.361	0.531	4.04E-05	Comt	EpC_Ageddown	EpC
2.81E-10	-0.2106314	0.265	0.424	6.75E-06	Gid8	EpC_Ageddown	EpC
1.80E-12	-0.2107383	0.094	0.237	4.32E-08	Lipm	EpC_Ageddown	EpC
2.54E-09	-0.2108028	0.83	0.94	6.08E-05	Hnrnpk	EpC_Ageddown	EpC
3.01E-09	-0.2121414	0.479	0.648	7.22E-05	Commd6	EpC_Ageddown	EpC
2.78E-09	-0.2123681	0.059	0.158	6.66E-05	Jag2	EpC_Ageddown	EpC
6.03E-10	-0.212753	0.691	0.845	1.45E-05	Ptprf	EpC_Ageddown	EpC
8.80E-11	-0.2127775	0.535	0.718	2.11E-06	Zcrb1	EpC_Ageddown	EpC
1.87E-06	-0.2128429	0.292	0.417	0.044767	Tsc22d3	EpC_Ageddown	EpC
9.46E-09	-0.2131184	0.349	0.505	0.000227	Ddx46	EpC_Ageddown	EpC
1.75E-11	-0.2133832	0.514	0.721	4.19E-07	Rbm8a	EpC_Ageddown	EpC
2.55E-08	-0.2136503	0.324	0.471	0.000612	Itgb4	EpC_Ageddown	EpC
1.56E-13	-0.2140482	0.191	0.373	3.75E-09	Cdc25b	EpC_Ageddown	EpC
8.30E-12	-0.2146421	0.223	0.397	1.99E-07	Eif2a	EpC_Ageddown	EpC
2.02E-13	-0.2151235	0.179	0.358	4.84E-09	Myo1e	EpC_Ageddown	EpC
4.23E-11	-0.2152693	0.363	0.549	1.01E-06	Slc25a11	EpC_Ageddown	EpC
1.49E-09	-0.2156241	0.556	0.738	3.58E-05	Reep3	EpC_Ageddown	EpC
1.60E-15	-0.2162522	0.18	0.375	3.83E-11	Taf12	EpC_Ageddown	EpC
4.82E-09	-0.2168756	0.489	0.645	0.000116	Gipc1	EpC_Ageddown	EpC
2.01E-10	-0.2170292	0.258	0.426	4.83E-06	Phactr4	EpC_Ageddown	EpC
1.59E-11	-0.2179161	0.302	0.475	3.82E-07	Wac	EpC_Ageddown	EpC
5.96E-10	-0.2180673	0.295	0.447	1.43E-05	Ap3s1	EpC_Ageddown	EpC
2.52E-10	-0.2186197	0.411	0.592	6.05E-06	Banf1	EpC_Ageddown	EpC
1.88E-10	-0.2187446	0.379	0.561	4.51E-06	Prpf40a	EpC_Ageddown	EpC
6.93E-07	-0.2190779	1	0.999	0.016625	Rps10	EpC_Ageddown	EpC
1.68E-11	-0.2197232	0.497	0.692	4.03E-07	Pkp3	EpC_Ageddown	EpC
5.62E-09	-0.2197529	0.669	0.807	0.000135	Foxp1	EpC_Ageddown	EpC
8.25E-09	-0.2202521	0.184	0.316	0.000198	Tns4	EpC_Ageddown	EpC
4.42E-08	-0.220472	0.852	0.903	0.001059	Arpc3	EpC_Ageddown	EpC
4.42E-15	-0.2206367	0.175	0.371	1.06E-10	Rsl24d1	EpC_Ageddown	EpC
5.26E-11	-0.220925	0.346	0.542	1.26E-06	Zdhhc5	EpC_Ageddown	EpC
1.45E-08	-0.2216988	0.046	0.132	0.000347	Wnt10a	EpC_Ageddown	EpC
5.30E-09	-0.2233082	0.518	0.669	0.000127	Pon2	EpC_Ageddown	EpC
1.07E-12	-0.2236472	0.983	0.997	2.55E-08	Naca	EpC_Ageddown	EpC
1.05E-11	-0.2241956	0.476	0.669	2.51E-07	Zc3h15	EpC_Ageddown	EpC
6.53E-14	-0.2242092	0.172	0.351	1.57E-09	Cyp2j6	EpC_Ageddown	EpC
1.32E-10	-0.2247185	0.322	0.493	3.15E-06	Trps1	EpC_Ageddown	EpC
1.86E-11	-0.2250187	0.877	0.959	4.45E-07	Clta	EpC_Ageddown	EpC
5.50E-11	-0.2254429	0.393	0.574	1.32E-06	Mob2	EpC_Ageddown	EpC
3.58E-13	-0.225541	0.474	0.684	8.58E-09	Rtn3	EpC_Ageddown	EpC
1.60E-08	-0.2256301	0.998	0.999	0.000384	Rpl34	EpC_Ageddown	EpC
2.82E-11	-0.2261686	0.583	0.763	6.76E-07	Vapa	EpC_Ageddown	EpC
4.38E-07	-0.2267866	0.592	0.724	0.010492	Arpc5l	EpC_Ageddown	EpC
4.73E-12	-0.2272476	0.425	0.622	1.13E-07	Chic2	EpC_Ageddown	EpC
1.06E-10	-0.227752	0.084	0.209	2.55E-06	Ccdc71l	EpC_Ageddown	EpC
2.20E-07	-0.2277559	0.811	0.904	0.005267	S100a16	EpC_Ageddown	EpC
2.93E-10	-0.2281553	0.695	0.824	7.03E-06	Tmed9	EpC_Ageddown	EpC
2.88E-13	-0.2285824	0.078	0.22	6.89E-09	Frat2	EpC_Ageddown	EpC
2.03E-09	-0.2288023	0.843	0.927	4.87E-05	Ybx1	EpC_Ageddown	EpC
6.96E-09	-0.2294576	0.422	0.587	0.000167	Dek	EpC_Ageddown	EpC
6.68E-11	-0.2294965	0.454	0.62	1.60E-06	Fgfr1op2	EpC_Ageddown	EpC
3.40E-17	-0.2295213	0.128	0.324	8.14E-13	Rab7b	EpC_Ageddown	EpC
8.91E-10	-0.229639	0.707	0.835	2.14E-05	Scp2	EpC_Ageddown	EpC
9.25E-09	-0.2299121	0.056	0.148	0.000222	Icam1	EpC_Ageddown	EpC
5.02E-10	-0.2299469	0.465	0.645	1.20E-05	Rala	EpC_Ageddown	EpC

7.92E-10	-0.2302084	0.516	0.692	1.90E-05	Rp9	EpC_Ageddown	EpC
1.74E-09	-0.2302799	0.791	0.904	4.17E-05	Cdc42	EpC_Ageddown	EpC
1.45E-07	-0.2302819	0.514	0.652	0.003483	Chd4	EpC_Ageddown	EpC
3.01E-15	-0.2312838	0.185	0.378	7.21E-11	Sh3gl1	EpC_Ageddown	EpC
9.31E-10	-0.2314417	0.551	0.725	2.23E-05	Snx3	EpC_Ageddown	EpC
3.29E-10	-0.2314655	0.219	0.376	7.89E-06	Snhg12	EpC_Ageddown	EpC
6.00E-14	-0.2315263	0.14	0.306	1.44E-09	Gpr87	EpC_Ageddown	EpC
9.30E-11	-0.2318657	0.459	0.654	2.23E-06	Cct3	EpC_Ageddown	EpC
2.54E-12	-0.2324979	0.943	0.975	6.08E-08	mt-Nd3	EpC_Ageddown	EpC
1.13E-06	-0.2326797	0.993	0.997	0.027003	Rps3	EpC_Ageddown	EpC
1.28E-11	-0.2327658	0.293	0.479	3.08E-07	Bnip2	EpC_Ageddown	EpC
1.58E-09	-0.2331354	0.914	0.969	3.78E-05	Atp5b	EpC_Ageddown	EpC
6.71E-14	-0.2331368	0.01	0.112	1.61E-09	St6galnac1	EpC_Ageddown	EpC
7.87E-10	-0.2334269	0.666	0.809	1.89E-05	Brk1	EpC_Ageddown	EpC
2.44E-08	-0.2339686	0.118	0.233	0.000585	Prss23	EpC_Ageddown	EpC
7.06E-09	-0.234106	0.594	0.74	0.000169	Cct2	EpC_Ageddown	EpC
5.53E-10	-0.2344708	0.234	0.389	1.33E-05	Bicdl2	EpC_Ageddown	EpC
1.41E-07	-0.2351473	0.106	0.213	0.003377	Nrp2	EpC_Ageddown	EpC
4.86E-09	-0.2351672	0.509	0.672	0.000116	Nxn	EpC_Ageddown	EpC
2.78E-13	-0.2355657	0.304	0.507	6.66E-09	Rtf1	EpC_Ageddown	EpC
1.73E-10	-0.2356835	0.589	0.746	4.16E-06	Ube2i	EpC_Ageddown	EpC
2.69E-13	-0.2357077	0.374	0.581	6.45E-09	Mtpn	EpC_Ageddown	EpC
1.84E-16	-0.2359752	0.113	0.298	4.40E-12	Wnt5a	EpC_Ageddown	EpC
4.72E-12	-0.23651	0.317	0.507	1.13E-07	Pard3	EpC_Ageddown	EpC
5.14E-13	-0.2366838	0.325	0.529	1.23E-08	Zbtb7c	EpC_Ageddown	EpC
1.33E-09	-0.2372948	0.524	0.694	3.20E-05	Cd47	EpC_Ageddown	EpC
2.17E-12	-0.2376486	0.533	0.736	5.20E-08	Cct5	EpC_Ageddown	EpC
1.24E-12	-0.2379358	0.207	0.396	2.96E-08	Ces2g	EpC_Ageddown	EpC
6.96E-15	-0.2385019	0.256	0.471	1.67E-10	Ahsa1	EpC_Ageddown	EpC
4.43E-12	-0.2391567	0.391	0.594	1.06E-07	Ythdc1	EpC_Ageddown	EpC
1.03E-11	-0.2397879	0.831	0.938	2.48E-07	Laptm4a	EpC_Ageddown	EpC
3.83E-09	-0.2398896	0.32	0.467	9.19E-05	Tgm1	EpC_Ageddown	EpC
1.14E-14	-0.2408208	0.253	0.464	2.72E-10	Tef	EpC_Ageddown	EpC
3.41E-12	-0.2413149	0.646	0.817	8.17E-08	Dctn3	EpC_Ageddown	EpC
2.47E-10	-0.2423779	0.43	0.601	5.92E-06	Ube2r2	EpC_Ageddown	EpC
7.71E-08	-0.2427597	0.444	0.591	0.00185	Elovl7	EpC_Ageddown	EpC
9.19E-15	-0.2429306	0.418	0.652	2.20E-10	Smap1	EpC_Ageddown	EpC
6.66E-09	-0.2430318	0.84	0.93	0.00016	Ppp1r14b	EpC_Ageddown	EpC
4.39E-08	-0.2441598	0.403	0.547	0.001053	Smagp	EpC_Ageddown	EpC
4.20E-08	-0.2447321	0.973	0.988	0.001008	Eef1b2	EpC_Ageddown	EpC
6.86E-07	-0.2459504	0.336	0.46	0.016451	Xpa	EpC_Ageddown	EpC
5.25E-09	-0.2469835	0.86	0.919	0.000126	Ahnak	EpC_Ageddown	EpC
7.57E-09	-0.2477566	0.669	0.791	0.000182	Cltb	EpC_Ageddown	EpC
1.34E-10	-0.2482291	0.415	0.588	3.22E-06	Ppp2r2a	EpC_Ageddown	EpC
5.75E-07	-0.2485289	0.998	0.995	0.013781	Rps4x	EpC_Ageddown	EpC
9.72E-13	-0.2487074	0.278	0.47	2.33E-08	Rarg	EpC_Ageddown	EpC
5.88E-08	-0.248892	0.182	0.308	0.001409	Ovol1	EpC_Ageddown	EpC
1.19E-15	-0.2489692	0.393	0.626	2.86E-11	Yipf4	EpC_Ageddown	EpC
2.63E-08	-0.2490678	0.064	0.159	0.000632	Tnc	EpC_Ageddown	EpC
1.86E-13	-0.2490925	0.602	0.803	4.47E-09	Bcap31	EpC_Ageddown	EpC
6.10E-13	-0.2502562	0.56	0.758	1.46E-08	Psmd4	EpC_Ageddown	EpC
1.97E-13	-0.2504299	0.234	0.426	4.72E-09	Pard6g	EpC_Ageddown	EpC
6.58E-12	-0.2505055	0.455	0.655	1.58E-07	Med21	EpC_Ageddown	EpC
5.99E-17	-0.2506699	0.246	0.474	1.44E-12	Ankrd12	EpC_Ageddown	EpC
5.08E-12	-0.251584	0.825	0.926	1.22E-07	Rbm39	EpC_Ageddown	EpC
8.71E-15	-0.2517479	0.234	0.437	2.09E-10	Mpp7	EpC_Ageddown	EpC
2.12E-07	-0.2521635	0.275	0.403	0.005086	Ethe1	EpC_Ageddown	EpC
2.65E-11	-0.2525183	0.491	0.676	6.36E-07	Cdc37	EpC_Ageddown	EpC
6.07E-10	-0.2525582	0.499	0.675	1.46E-05	Rpl13a	EpC_Ageddown	EpC

9.05E-09	-0.2532871	0.46	0.621	0.000217	Cbr3	EpC_Ageddown	EpC
1.61E-15	-0.253812	0.191	0.394	3.85E-11	Amotl1	EpC_Ageddown	EpC
2.75E-14	-0.2542486	0.497	0.699	6.59E-10	Mob4	EpC_Ageddown	EpC
3.01E-10	-0.2546983	0.99	0.998	7.22E-06	Actg1	EpC_Ageddown	EpC
3.00E-10	-0.255263	0.282	0.44	7.20E-06	Trp53	EpC_Ageddown	EpC
4.15E-10	-0.2554435	0.297	0.455	9.95E-06	F3	EpC_Ageddown	EpC
3.63E-09	-0.2555803	0.371	0.535	8.69E-05	Lmo7	EpC_Ageddown	EpC
2.15E-18	-0.2556043	0.143	0.351	5.15E-14	Wdr89	EpC_Ageddown	EpC
2.43E-13	-0.2556045	0.123	0.283	5.82E-09	Fam135a	EpC_Ageddown	EpC
2.70E-12	-0.2557122	0.447	0.642	6.48E-08	Dync1i2	EpC_Ageddown	EpC
2.25E-12	-0.2564575	0.363	0.561	5.39E-08	Myh9	EpC_Ageddown	EpC
1.06E-13	-0.2576212	0.602	0.769	2.53E-09	Srsf9	EpC_Ageddown	EpC
1.40E-13	-0.2581278	0.239	0.428	3.36E-09	Zkscan3	EpC_Ageddown	EpC
1.26E-07	-0.2585345	0.413	0.542	0.00302	Ly6g6e	EpC_Ageddown	EpC
1.07E-17	-0.2589563	0.207	0.433	2.58E-13	3830406C13Rik	EpC_Ageddown	EpC
3.26E-11	-0.2595295	0.681	0.821	7.81E-07	Ube2b	EpC_Ageddown	EpC
1.59E-09	-0.2605491	0.408	0.574	3.81E-05	Dsg3	EpC_Ageddown	EpC
1.73E-12	-0.2607104	0.177	0.343	4.15E-08	Tgm5	EpC_Ageddown	EpC
4.94E-12	-0.2608214	0.268	0.438	1.19E-07	Dnajb9	EpC_Ageddown	EpC
1.38E-14	-0.2614804	0.155	0.336	3.32E-10	Tspan5	EpC_Ageddown	EpC
4.58E-13	-0.2617973	0.465	0.658	1.10E-08	Kmt5a	EpC_Ageddown	EpC
1.60E-16	-0.2623555	0.219	0.43	3.84E-12	Csnk2a2	EpC_Ageddown	EpC
1.84E-07	-0.2623908	0.995	0.998	0.00442	Rpl32	EpC_Ageddown	EpC
2.25E-07	-0.2625874	0.605	0.709	0.005397	Ppp1r2	EpC_Ageddown	EpC
2.37E-10	-0.2630956	0.921	0.978	5.69E-06	Rpl31	EpC_Ageddown	EpC
3.22E-17	-0.2638091	0.28	0.514	7.73E-13	Cops4	EpC_Ageddown	EpC
7.88E-11	-0.2648013	0.45	0.611	1.89E-06	Chmp2b	EpC_Ageddown	EpC
7.85E-15	-0.2652625	0.432	0.657	1.88E-10	Zbtb7a	EpC_Ageddown	EpC
1.63E-06	-0.2653406	0.993	0.994	0.039062	Rps15a	EpC_Ageddown	EpC
2.31E-12	-0.2654705	0.349	0.538	5.53E-08	Sh3d19	EpC_Ageddown	EpC
2.12E-07	-0.2662829	0.049	0.128	0.00509	Cdkn1c	EpC_Ageddown	EpC
2.22E-13	-0.2665423	0.155	0.318	5.33E-09	Elovl6	EpC_Ageddown	EpC
7.61E-09	-0.2686351	0.676	0.79	0.000183	Manf	EpC_Ageddown	EpC
4.15E-13	-0.2688346	0.142	0.306	9.95E-09	Tmprss11g	EpC_Ageddown	EpC
2.95E-12	-0.2688354	0.31	0.493	7.08E-08	Tmem65	EpC_Ageddown	EpC
7.33E-12	-0.2697719	0.494	0.671	1.76E-07	Tra2b	EpC_Ageddown	EpC
4.90E-25	-0.2700974	0.078	0.302	1.18E-20	Banp	EpC_Ageddown	EpC
4.62E-16	-0.2708322	0.064	0.22	1.11E-11	Cav1	EpC_Ageddown	EpC
1.71E-09	-0.2713056	0.565	0.716	4.10E-05	Gm16136	EpC_Ageddown	EpC
3.14E-09	-0.2713394	0.302	0.446	7.53E-05	Rhod	EpC_Ageddown	EpC
6.10E-10	-0.2719757	0.285	0.445	1.46E-05	Adgrl2	EpC_Ageddown	EpC
9.59E-14	-0.2721342	0.6	0.798	2.30E-09	Sf3b1	EpC_Ageddown	EpC
3.00E-12	-0.2723519	0.179	0.345	7.19E-08	Vsig10l	EpC_Ageddown	EpC
2.69E-12	-0.272546	0.457	0.644	6.45E-08	Ppig	EpC_Ageddown	EpC
5.45E-16	-0.2731853	0.425	0.648	1.31E-11	Ubxn4	EpC_Ageddown	EpC
1.25E-13	-0.2733819	0.636	0.815	3.01E-09	Ywhab	EpC_Ageddown	EpC
1.39E-07	-0.2738363	0.983	0.987	0.00333	Rps6	EpC_Ageddown	EpC
2.73E-08	-0.2740922	0.997	0.997	0.000655	Rpl28	EpC_Ageddown	EpC
1.65E-10	-0.2742407	0.037	0.134	3.96E-06	Dlk2	EpC_Ageddown	EpC
6.78E-10	-0.274284	0.998	0.998	1.63E-05	Rpl27a	EpC_Ageddown	EpC
1.25E-10	-0.2743679	0.993	0.996	3.00E-06	Rpl8	EpC_Ageddown	EpC
9.23E-12	-0.2744163	0.504	0.693	2.21E-07	Cct8	EpC_Ageddown	EpC
7.23E-11	-0.2748199	0.201	0.358	1.73E-06	Cav2	EpC_Ageddown	EpC
1.58E-15	-0.2750638	0.499	0.73	3.78E-11	Atxn7l3b	EpC_Ageddown	EpC
4.19E-10	-0.2752625	0.663	0.789	1.01E-05	Ezr	EpC_Ageddown	EpC
1.03E-14	-0.2755368	0.509	0.72	2.47E-10	Prkar1a	EpC_Ageddown	EpC
6.92E-16	-0.2762452	0.25	0.466	1.66E-11	Plxdc2	EpC_Ageddown	EpC
1.80E-10	-0.2768098	0.619	0.768	4.31E-06	Marcks	EpC_Ageddown	EpC
8.33E-13	-0.2768852	0.589	0.756	2.00E-08	Ptbp3	EpC_Ageddown	EpC

5.96E-13	-0.2777403	0.872	0.96	1.43E-08	Ywhae	EpC_Ageddown	EpC
1.31E-14	-0.2781001	0.101	0.264	3.15E-10	Aldh1a7	EpC_Ageddown	EpC
7.07E-12	-0.2786052	0.265	0.441	1.69E-07	Serf1	EpC_Ageddown	EpC
1.30E-15	-0.2788609	0.423	0.652	3.12E-11	Nudc	EpC_Ageddown	EpC
2.60E-26	-0.2791375	1	1	6.23E-22	mt-Cytb	EpC_Ageddown	EpC
1.91E-10	-0.2791793	0.997	0.994	4.59E-06	Rps3a1	EpC_Ageddown	EpC
1.84E-15	-0.2797612	0.374	0.604	4.41E-11	Gspt1	EpC_Ageddown	EpC
8.16E-12	-0.2799494	0.589	0.766	1.96E-07	Ppp2ca	EpC_Ageddown	EpC
8.59E-14	-0.2804408	0.052	0.182	2.06E-09	Snai2	EpC_Ageddown	EpC
5.58E-12	-0.2807417	0.531	0.733	1.34E-07	Psma4	EpC_Ageddown	EpC
1.12E-15	-0.2824762	0.27	0.48	2.69E-11	Necap2	EpC_Ageddown	EpC
1.26E-19	-0.2825279	0.074	0.258	3.02E-15	Ajuba	EpC_Ageddown	EpC
6.61E-16	-0.2830389	0.272	0.492	1.59E-11	Gls	EpC_Ageddown	EpC
4.50E-14	-0.2830776	0.422	0.64	1.08E-09	Klc3	EpC_Ageddown	EpC
3.05E-17	-0.2839265	0.963	0.996	7.31E-13	Btf3	EpC_Ageddown	EpC
2.09E-09	-0.2839735	0.565	0.714	5.02E-05	Atp6v1d	EpC_Ageddown	EpC
4.10E-12	-0.2861611	0.594	0.771	9.84E-08	Pmepa1	EpC_Ageddown	EpC
2.26E-15	-0.2862212	0.071	0.227	5.42E-11	Dsg1b	EpC_Ageddown	EpC
4.11E-12	-0.2866725	0.465	0.65	9.85E-08	Cdv3	EpC_Ageddown	EpC
2.08E-13	-0.2869861	0.454	0.647	4.98E-09	Pcmtd1	EpC_Ageddown	EpC
1.20E-14	-0.2872261	0.223	0.411	2.89E-10	Hspa4l	EpC_Ageddown	EpC
5.69E-13	-0.2872262	0.187	0.358	1.36E-08	Tmem54	EpC_Ageddown	EpC
1.10E-11	-0.2872558	0.322	0.5	2.64E-07	Tjp1	EpC_Ageddown	EpC
1.87E-11	-0.2874035	0.31	0.479	4.48E-07	Becn1	EpC_Ageddown	EpC
3.67E-11	-0.2880739	0.798	0.909	8.79E-07	Klf5	EpC_Ageddown	EpC
3.91E-11	-0.2889894	0.742	0.872	9.37E-07	Pcbp2	EpC_Ageddown	EpC
1.89E-18	-0.2896831	0.993	0.996	4.53E-14	Ubb	EpC_Ageddown	EpC
2.34E-16	-0.2905344	0.199	0.404	5.62E-12	Ammecr1	EpC_Ageddown	EpC
1.09E-16	-0.2911174	0.351	0.579	2.61E-12	Pten	EpC_Ageddown	EpC
4.82E-12	-0.2914174	0.669	0.815	1.16E-07	Eif3a	EpC_Ageddown	EpC
1.40E-16	-0.2919584	0.801	0.922	3.35E-12	Rhoa	EpC_Ageddown	EpC
3.42E-30	-0.2928845	1	1	8.20E-26	mt-Atp6	EpC_Ageddown	EpC
5.64E-14	-0.29308	0.329	0.539	1.35E-09	Trp63	EpC_Ageddown	EpC
1.17E-11	-0.2934353	0.993	0.998	2.80E-07	Rpl15	EpC_Ageddown	EpC
4.20E-24	-0.2947081	0.094	0.319	1.01E-19	Ahsa2	EpC_Ageddown	EpC
6.70E-12	-0.2949657	0.565	0.736	1.61E-07	Eif5b	EpC_Ageddown	EpC
2.81E-10	-0.2949889	0.212	0.371	6.74E-06	Psapl1	EpC_Ageddown	EpC
4.29E-11	-0.2951672	0.998	0.998	1.03E-06	Rpl35a	EpC_Ageddown	EpC
4.54E-16	-0.2958124	0.61	0.804	1.09E-11	Arhgap5	EpC_Ageddown	EpC
1.39E-14	-0.2961186	0.455	0.665	3.33E-10	Ppp3ca	EpC_Ageddown	EpC
1.32E-12	-0.2964679	0.428	0.605	3.16E-08	Mpzl2	EpC_Ageddown	EpC
1.94E-14	-0.2966448	0.585	0.789	4.66E-10	Llph	EpC_Ageddown	EpC
3.14E-12	-0.2967147	0.278	0.46	7.53E-08	Mindy3	EpC_Ageddown	EpC
1.26E-14	-0.2969088	0.246	0.444	3.03E-10	Mrps6	EpC_Ageddown	EpC
3.62E-14	-0.296979	0.26	0.445	8.68E-10	Coq10b	EpC_Ageddown	EpC
3.20E-09	-0.2974515	0.944	0.971	7.68E-05	Rpl4	EpC_Ageddown	EpC
2.56E-13	-0.297686	0.841	0.939	6.13E-09	Hmgb1	EpC_Ageddown	EpC
4.59E-07	-0.2987534	0.239	0.358	0.011003	Sfrp1	EpC_Ageddown	EpC
4.35E-14	-0.2992599	0.147	0.325	1.04E-09	Rdh12	EpC_Ageddown	EpC
3.66E-12	-0.2997279	0.695	0.842	8.77E-08	Ralbp1	EpC_Ageddown	EpC
3.49E-09	-0.299923	0.283	0.438	8.37E-05	Stmn1	EpC_Ageddown	EpC
3.05E-17	-0.3001688	0.315	0.548	7.31E-13	Senp6	EpC_Ageddown	EpC
2.34E-13	-0.3019457	0.782	0.886	5.61E-09	Calr	EpC_Ageddown	EpC
3.36E-12	-0.3020359	0.297	0.476	8.05E-08	Tmpo	EpC_Ageddown	EpC
2.07E-15	-0.3022456	0.487	0.689	4.97E-11	Pitpna	EpC_Ageddown	EpC
1.26E-12	-0.302476	0.487	0.686	3.03E-08	Trim2	EpC_Ageddown	EpC
1.69E-13	-0.3024926	0.13	0.291	4.04E-09	Map3k6	EpC_Ageddown	EpC
6.31E-14	-0.3032007	0.715	0.841	1.51E-09	Ppp1cb	EpC_Ageddown	EpC
5.39E-07	-0.3034998	0.266	0.402	0.012923	Hbegf	EpC_Ageddown	EpC

2.15E-14	-0.3036394	0.464	0.67	5.15E-10	Srsf11	EpC_Ageddown	EpC
2.00E-09	-0.3039729	0.902	0.943	4.80E-05	Calm1	EpC_Ageddown	EpC
1.29E-08	-0.3052072	0.066	0.162	0.000309	Wfdc5	EpC_Ageddown	EpC
2.05E-15	-0.3059944	0.452	0.657	4.91E-11	Arl1	EpC_Ageddown	EpC
1.04E-14	-0.3063986	0.401	0.605	2.48E-10	Kif21a	EpC_Ageddown	EpC
1.11E-14	-0.3097653	0.165	0.337	2.66E-10	Snrnp25	EpC_Ageddown	EpC
7.34E-11	-0.3103296	0.189	0.342	1.76E-06	Cyp3a13	EpC_Ageddown	EpC
9.11E-09	-0.3107864	0.874	0.915	0.000218	Rab25	EpC_Ageddown	EpC
1.51E-13	-0.3115344	0.054	0.187	3.61E-09	Gjb6	EpC_Ageddown	EpC
5.49E-08	-0.3115845	0.509	0.64	0.001316	Pls3	EpC_Ageddown	EpC
6.57E-16	-0.3117312	0.246	0.448	1.57E-11	Sox21	EpC_Ageddown	EpC
1.60E-10	-0.3118076	0.877	0.934	3.85E-06	Eef1g	EpC_Ageddown	EpC
2.54E-18	-0.3120219	0.489	0.736	6.10E-14	U2af1	EpC_Ageddown	EpC
3.43E-13	-0.3133327	0.349	0.536	8.22E-09	Nebl	EpC_Ageddown	EpC
1.89E-15	-0.3140622	0.078	0.235	4.54E-11	Tprg	EpC_Ageddown	EpC
2.57E-16	-0.314439	0.648	0.844	6.17E-12	Sumo1	EpC_Ageddown	EpC
1.27E-09	-0.3156142	0.702	0.818	3.04E-05	Prdx6	EpC_Ageddown	EpC
1.59E-14	-0.3161561	0.292	0.49	3.82E-10	Kctd1	EpC_Ageddown	EpC
1.26E-19	-0.3166145	0.332	0.586	3.03E-15	Acp1	EpC_Ageddown	EpC
2.44E-16	-0.3174842	0.604	0.788	5.85E-12	Rab14	EpC_Ageddown	EpC
1.22E-18	-0.3178922	0.319	0.559	2.93E-14	Tnrc6c	EpC_Ageddown	EpC
1.60E-14	-0.317994	0.073	0.219	3.83E-10	Fbln2	EpC_Ageddown	EpC
3.21E-08	-0.3193501	0.467	0.618	0.00077	Evpl	EpC_Ageddown	EpC
4.73E-14	-0.3196824	0.059	0.196	1.13E-09	Pou3f1	EpC_Ageddown	EpC
2.04E-19	-0.3222742	0.391	0.636	4.90E-15	Ankrd11	EpC_Ageddown	EpC
7.82E-18	-0.3227456	0.57	0.778	1.87E-13	Dnaja2	EpC_Ageddown	EpC
1.74E-17	-0.3229873	0.695	0.875	4.16E-13	Vdac1	EpC_Ageddown	EpC
5.23E-16	-0.3244444	0.245	0.447	1.25E-11	Bag2	EpC_Ageddown	EpC
6.42E-17	-0.324678	0.501	0.714	1.54E-12	Rdx	EpC_Ageddown	EpC
3.93E-16	-0.32545	0.646	0.805	9.43E-12	Srsf2	EpC_Ageddown	EpC
5.40E-14	-0.326086	0.423	0.618	1.29E-09	Eif4e2	EpC_Ageddown	EpC
5.68E-11	-0.3271902	0.261	0.427	1.36E-06	Flrt2	EpC_Ageddown	EpC
1.29E-09	-0.3274552	0.988	0.989	3.09E-05	Rpl3	EpC_Ageddown	EpC
3.92E-19	-0.3282504	0.138	0.345	9.41E-15	Dbp	EpC_Ageddown	EpC
3.79E-15	-0.3304017	0.605	0.786	9.08E-11	Sh3glb1	EpC_Ageddown	EpC
1.66E-11	-0.3309667	0.13	0.273	3.98E-07	Gsdma	EpC_Ageddown	EpC
1.04E-07	-0.3310979	0.432	0.552	0.0025	Wnt4	EpC_Ageddown	EpC
1.25E-18	-0.3326733	0.278	0.502	2.99E-14	Zfp326	EpC_Ageddown	EpC
2.41E-12	-0.33332	0.639	0.791	5.79E-08	Cast	EpC_Ageddown	EpC
1.86E-23	-0.3342078	0.096	0.317	4.45E-19	Cd164l2	EpC_Ageddown	EpC
1.58E-13	-0.334474	0.388	0.574	3.79E-09	Palld	EpC_Ageddown	EpC
1.68E-18	-0.3349228	0.425	0.658	4.02E-14	Casz1	EpC_Ageddown	EpC
5.15E-09	-0.3350746	0.361	0.51	0.000124	MuClm	EpC_Ageddown	EpC
1.47E-20	-0.3353883	0.327	0.576	3.52E-16	Snrnp48	EpC_Ageddown	EpC
8.94E-19	-0.3354156	0.75	0.911	2.14E-14	Hnrnpf	EpC_Ageddown	EpC
8.62E-16	-0.3360519	0.509	0.714	2.07E-11	Arglu1	EpC_Ageddown	EpC
1.08E-17	-0.3373766	0.337	0.555	2.58E-13	Strn3	EpC_Ageddown	EpC
1.16E-11	-0.3381814	0.285	0.448	2.77E-07	Dusp22	EpC_Ageddown	EpC
8.75E-14	-0.3385409	0.619	0.763	2.10E-09	Psmd8	EpC_Ageddown	EpC
2.15E-13	-0.3388504	0.103	0.253	5.15E-09	Trex2	EpC_Ageddown	EpC
6.88E-25	-0.3399593	0.191	0.442	1.65E-20	Lamtor3	EpC_Ageddown	EpC
5.44E-16	-0.3402171	0.251	0.46	1.31E-11	Mafb	EpC_Ageddown	EpC
1.88E-10	-0.3402852	0.696	0.831	4.51E-06	Srp14	EpC_Ageddown	EpC
1.13E-10	-0.3406429	0.853	0.941	2.70E-06	Ubc	EpC_Ageddown	EpC
6.81E-16	-0.3411277	0.487	0.703	1.63E-11	Slc38a2	EpC_Ageddown	EpC
1.51E-13	-0.3411913	0.717	0.857	3.63E-09	Esd	EpC_Ageddown	EpC
1.24E-15	-0.3413685	0.75	0.874	2.97E-11	Paip2	EpC_Ageddown	EpC
2.06E-12	-0.3423727	0.632	0.796	4.94E-08	Mgst3	EpC_Ageddown	EpC
1.90E-13	-0.3445199	0.41	0.615	4.56E-09	Mgst2	EpC_Ageddown	EpC

6.28E-20	-0.3453283	0.676	0.851	1.51E-15	Ptges3	EpC_Ageddown	EpC
6.91E-23	-0.3455426	0.995	0.999	1.66E-18	Fxyd3	EpC_Ageddown	EpC
5.92E-15	-0.3460193	0.449	0.633	1.42E-10	Rexo2	EpC_Ageddown	EpC
4.50E-14	-0.3465282	0.6	0.785	1.08E-09	Nap1l1	EpC_Ageddown	EpC
1.42E-08	-0.3466889	0.518	0.67	0.00034	Plec	EpC_Ageddown	EpC
5.73E-17	-0.3493705	0.093	0.264	1.37E-12	UMuCg	EpC_Ageddown	EpC
2.83E-17	-0.3546731	0.617	0.832	6.79E-13	Sfr1	EpC_Ageddown	EpC
1.79E-10	-0.3556834	0.386	0.547	4.29E-06	Gadd45b	EpC_Ageddown	EpC
9.69E-08	-0.3560678	0.384	0.533	0.002325	Dapl1	EpC_Ageddown	EpC
3.73E-17	-0.3593364	0.26	0.468	8.95E-13	Rassf1	EpC_Ageddown	EpC
5.98E-10	-0.36175	0.293	0.439	1.43E-05	Shroom3	EpC_Ageddown	EpC
2.05E-17	-0.3617527	0.963	0.99	4.93E-13	Rpl29	EpC_Ageddown	EpC
2.53E-23	-0.3620236	0.3	0.562	6.07E-19	Stip1	EpC_Ageddown	EpC
2.63E-13	-0.3626524	0.742	0.86	6.31E-09	Set	EpC_Ageddown	EpC
6.15E-12	-0.362846	0.998	0.998	1.47E-07	Rpl13	EpC_Ageddown	EpC
2.31E-18	-0.3633592	0.735	0.883	5.53E-14	Cox7a2l	EpC_Ageddown	EpC
4.07E-14	-0.3633946	0.838	0.924	9.77E-10	Hmgn1	EpC_Ageddown	EpC
3.67E-15	-0.3638072	0.354	0.554	8.81E-11	Mbnl1	EpC_Ageddown	EpC
1.44E-19	-0.3640751	0.548	0.759	3.45E-15	Pdap1	EpC_Ageddown	EpC
5.63E-18	-0.3658989	0.45	0.673	1.35E-13	Tprgl	EpC_Ageddown	EpC
3.84E-18	-0.3672512	0.214	0.432	9.20E-14	Bcl11b	EpC_Ageddown	EpC
4.50E-20	-0.3676837	0.541	0.774	1.08E-15	Metap2	EpC_Ageddown	EpC
9.69E-25	-0.369347	0.268	0.54	2.32E-20	Mettl23	EpC_Ageddown	EpC
1.62E-19	-0.3700765	0.105	0.303	3.87E-15	Vsnl1	EpC_Ageddown	EpC
3.86E-19	-0.3721446	1	0.999	9.25E-15	Rplp0	EpC_Ageddown	EpC
1.58E-13	-0.3732787	0.995	0.998	3.79E-09	Rps16	EpC_Ageddown	EpC
1.17E-16	-0.3767182	0.221	0.407	2.81E-12	Homer2	EpC_Ageddown	EpC
1.00E-07	-0.3777365	0.675	0.756	0.002402	Dsc2	EpC_Ageddown	EpC
4.74E-18	-0.3777779	0.518	0.727	1.14E-13	Uqcrc2	EpC_Ageddown	EpC
3.29E-10	-0.3778314	0.914	0.965	7.89E-06	Npm1	EpC_Ageddown	EpC
1.71E-15	-0.378018	0.968	0.98	4.09E-11	Rps17	EpC_Ageddown	EpC
3.87E-12	-0.3780601	0.607	0.75	9.28E-08	Sri	EpC_Ageddown	EpC
1.75E-10	-0.3799229	0.393	0.559	4.19E-06	Sult2b1	EpC_Ageddown	EpC
8.22E-11	-0.3806778	0.779	0.863	1.97E-06	Eif6	EpC_Ageddown	EpC
2.09E-23	-0.3819075	0.376	0.647	5.02E-19	Pura	EpC_Ageddown	EpC
1.96E-29	-0.3844441	0.216	0.507	4.71E-25	Chordc1	EpC_Ageddown	EpC
2.21E-21	-0.3844477	0.482	0.723	5.31E-17	Cct4	EpC_Ageddown	EpC
2.34E-07	-0.3862655	0.84	0.882	0.005604	Prdx5	EpC_Ageddown	EpC
1.41E-12	-0.386466	0.189	0.345	3.38E-08	Me1	EpC_Ageddown	EpC
1.04E-14	-0.3884729	0.607	0.774	2.50E-10	Rbp1	EpC_Ageddown	EpC
2.26E-17	-0.3896167	0.685	0.846	5.43E-13	Rab10	EpC_Ageddown	EpC
2.44E-22	-0.3899753	0.798	0.928	5.84E-18	Ywhaz	EpC_Ageddown	EpC
4.54E-23	-0.3905547	0.11	0.328	1.09E-18	Serpinh1	EpC_Ageddown	EpC
3.65E-22	-0.3908944	0.99	0.998	8.75E-18	Cd9	EpC_Ageddown	EpC
9.85E-18	-0.3914731	0.605	0.775	2.36E-13	Atp6v1a	EpC_Ageddown	EpC
4.36E-19	-0.3918656	0.622	0.837	1.05E-14	Trim29	EpC_Ageddown	EpC
1.36E-08	-0.3918739	0.764	0.829	0.000327	Tubb4b	EpC_Ageddown	EpC
7.73E-17	-0.3930527	0.651	0.846	1.85E-12	Hmgb2	EpC_Ageddown	EpC
1.74E-12	-0.3931809	0.727	0.831	4.18E-08	Tacstd2	EpC_Ageddown	EpC
9.40E-21	-0.395194	0.494	0.723	2.25E-16	Hnrnpc	EpC_Ageddown	EpC
5.76E-20	-0.3958033	0.998	0.999	1.38E-15	Rpl23	EpC_Ageddown	EpC
1.81E-11	-0.3967754	0.212	0.367	4.34E-07	Ptprs	EpC_Ageddown	EpC
3.98E-09	-0.3980306	0.265	0.423	9.54E-05	Klk8	EpC_Ageddown	EpC
8.49E-08	-0.3981632	0.759	0.873	0.002037	Junb	EpC_Ageddown	EpC
4.94E-17	-0.4001361	0.556	0.729	1.18E-12	Ube2n	EpC_Ageddown	EpC
1.65E-21	-0.4028495	0.998	0.999	3.95E-17	Eef1a1	EpC_Ageddown	EpC
9.81E-15	-0.403796	0.295	0.473	2.35E-10	Rab27b	EpC_Ageddown	EpC
1.21E-25	-0.4048537	0.562	0.794	2.91E-21	Psmd7	EpC_Ageddown	EpC
2.15E-07	-0.4078677	0.506	0.645	0.005147	Ppl	EpC_Ageddown	EpC

6.79E-17	-0.4078727	0.545	0.734	1.63E-12	Hspd1	EpC_Ageddown	EpC
6.70E-23	-0.4080078	0.629	0.845	1.61E-18	Eif3e	EpC_Ageddown	EpC
3.84E-16	-0.4133323	0.54	0.749	9.20E-12	Serpinb5	EpC_Ageddown	EpC
2.69E-24	-0.4136118	0.749	0.907	6.44E-20	Hnrnpab	EpC_Ageddown	EpC
4.03E-08	-0.4160077	0.03	0.106	0.000966	S100a7a	EpC_Ageddown	EpC
7.38E-24	-0.4160618	0.653	0.858	1.77E-19	Eif2s2	EpC_Ageddown	EpC
3.45E-15	-0.4172859	0.064	0.212	8.28E-11	Alox12b	EpC_Ageddown	EpC
9.61E-22	-0.4193831	0.381	0.642	2.31E-17	Atp11b	EpC_Ageddown	EpC
6.92E-27	-0.4217248	0.476	0.738	1.66E-22	St13	EpC_Ageddown	EpC
7.99E-13	-0.4217342	0.965	0.98	1.92E-08	Rack1	EpC_Ageddown	EpC
2.67E-12	-0.4230679	0.105	0.24	6.40E-08	Lmo1	EpC_Ageddown	EpC
3.00E-14	-0.424232	0.449	0.628	7.18E-10	Elovl1	EpC_Ageddown	EpC
2.96E-07	-0.4260752	0.696	0.759	0.007093	Rab11a	EpC_Ageddown	EpC
2.38E-19	-0.4269926	0.604	0.776	5.70E-15	Herpud1	EpC_Ageddown	EpC
7.81E-09	-0.4290663	0.194	0.326	0.000187	Ptn	EpC_Ageddown	EpC
9.99E-19	-0.430025	0.352	0.58	2.40E-14	Abhd17c	EpC_Ageddown	EpC
4.69E-49	-0.4312362	1	1	1.13E-44	Tpt1	EpC_Ageddown	EpC
9.26E-21	-0.4318481	0.575	0.761	2.22E-16	Anxa7	EpC_Ageddown	EpC
5.81E-22	-0.4341078	0.511	0.741	1.39E-17	Actr3	EpC_Ageddown	EpC
4.19E-21	-0.4359734	0.766	0.914	1.00E-16	2010111I01Rik	EpC_Ageddown	EpC
8.75E-29	-0.4368814	0.53	0.783	2.10E-24	Fkbp4	EpC_Ageddown	EpC
8.79E-21	-0.4385088	0.494	0.702	2.11E-16	Chmp5	EpC_Ageddown	EpC
2.09E-08	-0.4393187	0.916	0.963	0.000501	Gsto1	EpC_Ageddown	EpC
6.89E-22	-0.4397887	0.521	0.736	1.65E-17	Cnih4	EpC_Ageddown	EpC
3.51E-12	-0.4408854	0.135	0.276	8.42E-08	Mndal	EpC_Ageddown	EpC
4.21E-16	-0.4432803	0.462	0.659	1.01E-11	Tcf4	EpC_Ageddown	EpC
6.90E-27	-0.4488855	0.816	0.953	1.65E-22	Slc25a5	EpC_Ageddown	EpC
1.26E-12	-0.45021	0.727	0.857	3.03E-08	Map1lc3a	EpC_Ageddown	EpC
1.68E-23	-0.4516982	0.592	0.817	4.02E-19	Anp32b	EpC_Ageddown	EpC
1.06E-26	-0.4528239	0.642	0.861	2.53E-22	Eif5	EpC_Ageddown	EpC
2.81E-21	-0.4535189	0.185	0.414	6.73E-17	Efna3	EpC_Ageddown	EpC
6.04E-29	-0.4538349	0.477	0.757	1.45E-24	Dusp11	EpC_Ageddown	EpC
5.91E-16	-0.4548779	0.788	0.885	1.42E-11	Jup	EpC_Ageddown	EpC
4.69E-16	-0.4572846	0.725	0.853	1.12E-11	Nsa2	EpC_Ageddown	EpC
5.51E-24	-0.463358	0.772	0.927	1.32E-19	Hras	EpC_Ageddown	EpC
3.26E-16	-0.4640556	0.99	0.993	7.82E-12	Rps18	EpC_Ageddown	EpC
2.49E-22	-0.4649417	0.096	0.301	5.98E-18	Fam89a	EpC_Ageddown	EpC
1.04E-58	-0.4658504	1	1	2.48E-54	mt-Co3	EpC_Ageddown	EpC
3.08E-14	-0.4666112	0.612	0.774	7.39E-10	Foxq1	EpC_Ageddown	EpC
1.09E-19	-0.4697386	0.336	0.572	2.61E-15	Tuba4a	EpC_Ageddown	EpC
2.42E-12	-0.4697688	0.298	0.466	5.81E-08	Zfp36l2	EpC_Ageddown	EpC
1.90E-24	-0.4713228	0.452	0.712	4.56E-20	Nfe2l2	EpC_Ageddown	EpC
6.68E-27	-0.4714964	0.573	0.797	1.60E-22	Spop	EpC_Ageddown	EpC
3.61E-18	-0.4716269	0.794	0.881	8.65E-14	Hspa5	EpC_Ageddown	EpC
3.51E-15	-0.4739239	0.337	0.528	8.41E-11	Id4	EpC_Ageddown	EpC
5.89E-18	-0.4799018	0.791	0.88	1.41E-13	Hspe1	EpC_Ageddown	EpC
4.86E-14	-0.4803284	0.304	0.507	1.16E-09	Sptssb	EpC_Ageddown	EpC
1.56E-07	-0.4805092	0.614	0.658	0.00375	Sox9	EpC_Ageddown	EpC
1.71E-16	-0.4809923	0.828	0.913	4.10E-12	S100a10	EpC_Ageddown	EpC
5.71E-20	-0.483877	0.518	0.727	1.37E-15	Phlda3	EpC_Ageddown	EpC
2.50E-10	-0.4852996	0.088	0.207	5.99E-06	Ctgf	EpC_Ageddown	EpC
2.91E-19	-0.4869157	0.113	0.312	6.97E-15	Endou	EpC_Ageddown	EpC
5.62E-47	-0.4881224	1	1	1.35E-42	mt-Nd2	EpC_Ageddown	EpC
4.80E-21	-0.4894203	0.218	0.452	1.15E-16	Aldh3b2	EpC_Ageddown	EpC
2.65E-15	-0.4895821	0.28	0.467	6.35E-11	Paqr5	EpC_Ageddown	EpC
1.48E-10	-0.492774	0.211	0.358	3.54E-06	Htra1	EpC_Ageddown	EpC
2.04E-30	-0.4932223	0.346	0.639	4.90E-26	Cldnd1	EpC_Ageddown	EpC
6.31E-30	-0.4946137	0.998	1	1.51E-25	Rps14	EpC_Ageddown	EpC
2.47E-34	-0.5000701	0.207	0.512	5.93E-30	Tnfaip8	EpC_Ageddown	EpC

2.02E-28	-0.5011683	0.987	0.993	4.86E-24	Rplp2	EpC_Ageddown	EpC
5.84E-09	-0.5013163	0.486	0.595	0.00014	Pof1b	EpC_Ageddown	EpC
1.40E-34	-0.5022577	0.658	0.876	3.35E-30	Pcbp1	EpC_Ageddown	EpC
9.75E-29	-0.5072835	0.912	0.966	2.34E-24	Dstn	EpC_Ageddown	EpC
1.11E-12	-0.5086004	0.447	0.608	2.65E-08	Lypd3	EpC_Ageddown	EpC
9.51E-39	-0.5087976	0.852	0.967	2.28E-34	Eif4a1	EpC_Ageddown	EpC
1.89E-29	-0.5091767	0.583	0.812	4.54E-25	Purb	EpC_Ageddown	EpC
1.80E-34	-0.5098871	0.612	0.859	4.31E-30	Top1	EpC_Ageddown	EpC
4.83E-21	-0.5131898	0.72	0.896	1.16E-16	Pycard	EpC_Ageddown	EpC
7.07E-07	-0.5162329	0.59	0.696	0.016958	Egr1	EpC_Ageddown	EpC
1.14E-16	-0.5164891	0.314	0.516	2.72E-12	Htatip2	EpC_Ageddown	EpC
9.28E-14	-0.5198668	0.492	0.658	2.22E-09	Sox2	EpC_Ageddown	EpC
8.88E-30	-0.5203728	0.992	0.993	2.13E-25	Rpl21	EpC_Ageddown	EpC
6.31E-23	-0.522146	0.346	0.581	1.51E-18	Cux1	EpC_Ageddown	EpC
9.21E-32	-0.5272139	0.874	0.96	2.21E-27	Serbp1	EpC_Ageddown	EpC
3.41E-18	-0.5304579	0.245	0.452	8.18E-14	Tubb2a	EpC_Ageddown	EpC
3.34E-29	-0.5310389	0.57	0.814	8.00E-25	Hnrnpa0	EpC_Ageddown	EpC
2.56E-39	-0.5361502	0.428	0.736	6.15E-35	Cacybp	EpC_Ageddown	EpC
1.59E-06	-0.5363225	0.681	0.787	0.038184	Jun	EpC_Ageddown	EpC
3.70E-27	-0.5375927	0.379	0.664	8.88E-23	Ablim1	EpC_Ageddown	EpC
1.90E-35	-0.5398162	0.998	0.998	4.56E-31	Rpl26	EpC_Ageddown	EpC
1.31E-23	-0.5403681	0.897	0.957	3.15E-19	Prdx2	EpC_Ageddown	EpC
1.98E-21	-0.5415662	0.41	0.639	4.75E-17	Golim4	EpC_Ageddown	EpC
6.21E-12	-0.546205	0.405	0.549	1.49E-07	Them5	EpC_Ageddown	EpC
1.33E-08	-0.5510362	0.605	0.69	0.000318	Ggh	EpC_Ageddown	EpC
9.97E-25	-0.5532081	0.3	0.555	2.39E-20	Pdlim2	EpC_Ageddown	EpC
6.29E-33	-0.5540506	0.707	0.882	1.51E-28	Arpc2	EpC_Ageddown	EpC
2.07E-28	-0.5619294	0.594	0.825	4.96E-24	Capns2	EpC_Ageddown	EpC
6.82E-26	-0.5642085	0.987	0.995	1.63E-21	Rpl17	EpC_Ageddown	EpC
1.14E-16	-0.5651498	0.847	0.928	2.73E-12	Dsp	EpC_Ageddown	EpC
1.00E-24	-0.5670104	0.309	0.56	2.40E-20	Arap2	EpC_Ageddown	EpC
6.81E-08	-0.5700175	0.103	0.208	0.001632	Slurp1	EpC_Ageddown	EpC
1.79E-24	-0.5764038	0.642	0.83	4.30E-20	Actn4	EpC_Ageddown	EpC
1.22E-20	-0.5773941	0.953	0.991	2.91E-16	Dynll1	EpC_Ageddown	EpC
6.47E-22	-0.5789408	0.627	0.821	1.55E-17	Gltp	EpC_Ageddown	EpC
1.65E-07	-0.5810771	0.28	0.408	0.003952	Crabp2	EpC_Ageddown	EpC
3.67E-19	-0.5825814	0.324	0.54	8.79E-15	Cited2	EpC_Ageddown	EpC
1.75E-13	-0.5857622	0.202	0.375	4.20E-09	Il33	EpC_Ageddown	EpC
6.89E-09	-0.5857967	0.373	0.495	0.000165	Pinlyp	EpC_Ageddown	EpC
1.18E-12	-0.5906614	0.556	0.708	2.84E-08	Fam25c	EpC_Ageddown	EpC
1.23E-22	-0.5917502	0.954	0.985	2.95E-18	Perp	EpC_Ageddown	EpC
2.29E-13	-0.5927167	0.185	0.342	5.50E-09	Ephx3	EpC_Ageddown	EpC
1.93E-22	-0.5929432	0.317	0.569	4.62E-18	Mall	EpC_Ageddown	EpC
1.35E-15	-0.5933455	0.971	0.981	3.25E-11	Gsta4	EpC_Ageddown	EpC
1.08E-66	-0.595527	0.987	0.998	2.60E-62	H3f3a	EpC_Ageddown	EpC
5.05E-31	-0.6027926	0.75	0.91	1.21E-26	Skp1a	EpC_Ageddown	EpC
1.55E-11	-0.6052894	0.111	0.246	3.71E-07	Il1a	EpC_Ageddown	EpC
5.25E-09	-0.6199656	0.359	0.487	0.000126	Rbp2	EpC_Ageddown	EpC
2.00E-38	-0.6242561	0.445	0.745	4.79E-34	Acadl	EpC_Ageddown	EpC
2.56E-10	-0.6254687	0.145	0.278	6.14E-06	Fst	EpC_Ageddown	EpC
5.10E-21	-0.6310577	0.18	0.412	1.22E-16	Foxe1	EpC_Ageddown	EpC
2.10E-31	-0.6331549	0.578	0.792	5.04E-27	Dynlt3	EpC_Ageddown	EpC
1.55E-20	-0.6404673	0.68	0.837	3.72E-16	Klf4	EpC_Ageddown	EpC
1.16E-48	-0.6527831	0.992	0.994	2.78E-44	Rps21	EpC_Ageddown	EpC
3.24E-28	-0.6628959	0.755	0.877	7.78E-24	Ybx3	EpC_Ageddown	EpC
4.22E-30	-0.6670181	0.927	0.978	1.01E-25	Rps15	EpC_Ageddown	EpC
4.44E-09	-0.6699787	0.374	0.481	0.000106	Ifi202b	EpC_Ageddown	EpC
9.39E-25	-0.6707388	0.336	0.61	2.25E-20	Id1	EpC_Ageddown	EpC
1.50E-32	-0.6731726	0.393	0.676	3.60E-28	Tpm2	EpC_Ageddown	EpC

1.15E-19	-0.676795	0.26	0.492	2.77E-15	Id3	EpC_Ageddown	EpC
3.41E-10	-0.6793817	0.594	0.71	8.17E-06	Krt5	EpC_Ageddown	EpC
5.22E-19	-0.6793951	0.798	0.92	1.25E-14	Sfn	EpC_Ageddown	EpC
1.88E-29	-0.6795125	0.965	0.981	4.50E-25	Rpl12	EpC_Ageddown	EpC
3.43E-15	-0.6909786	0.823	0.913	8.22E-11	Lgals7	EpC_Ageddown	EpC
1.40E-10	-0.6924514	0.169	0.303	3.35E-06	Fetub	EpC_Ageddown	EpC
1.15E-14	-0.6928989	0.199	0.364	2.75E-10	Serpinb2	EpC_Ageddown	EpC
2.02E-09	-0.6987399	0.46	0.567	4.85E-05	Krt80	EpC_Ageddown	EpC
3.95E-12	-0.6994127	0.228	0.372	9.47E-08	Ccnd2	EpC_Ageddown	EpC
2.94E-13	-0.7000342	0.42	0.574	7.05E-09	Serpinb1a	EpC_Ageddown	EpC
3.57E-07	-0.7310214	0.148	0.264	0.008563	Psca	EpC_Ageddown	EpC
2.65E-16	-0.7520726	0.201	0.379	6.35E-12	Serpinb11	EpC_Ageddown	EpC
2.60E-22	-0.7583961	0.283	0.525	6.24E-18	Odc1	EpC_Ageddown	EpC
2.58E-53	-0.7608099	0.958	0.989	6.20E-49	Rps25	EpC_Ageddown	EpC
1.56E-17	-0.8007981	0.256	0.455	3.75E-13	Col17a1	EpC_Ageddown	EpC
2.08E-18	-0.8017703	0.204	0.402	4.98E-14	Elovl4	EpC_Ageddown	EpC
1.31E-28	-0.8067699	0.671	0.847	3.14E-24	S100a14	EpC_Ageddown	EpC
3.29E-18	-0.8080329	0.255	0.458	7.89E-14	Dst	EpC_Ageddown	EpC
6.09E-51	-0.8227383	0.998	0.997	1.46E-46	Rplp1	EpC_Ageddown	EpC
4.71E-18	-0.8358612	0.204	0.405	1.13E-13	Cysrt1	EpC_Ageddown	EpC
5.82E-61	-0.8661976	0.874	0.977	1.40E-56	Rpl23a	EpC_Ageddown	EpC
7.79E-24	-0.8733631	0.491	0.669	1.87E-19	Ctnnbip1	EpC_Ageddown	EpC
1.16E-26	-0.8769133	0.358	0.638	2.79E-22	Aqp3	EpC_Ageddown	EpC
1.34E-21	-0.8874107	0.292	0.54	3.22E-17	Adh7	EpC_Ageddown	EpC
8.67E-18	-0.8901192	0.838	0.897	2.08E-13	Krt6a	EpC_Ageddown	EpC
7.38E-70	-0.8919299	0.202	0.639	1.77E-65	Hsph1	EpC_Ageddown	EpC
3.46E-16	-0.8963751	0.234	0.424	8.30E-12	Csta1	EpC_Ageddown	EpC
3.45E-23	-0.9036072	0.589	0.796	8.28E-19	Emp1	EpC_Ageddown	EpC
5.53E-89	-0.9141402	0.376	0.912	1.33E-84	Tmem59	EpC_Ageddown	EpC
3.84E-12	-0.9230294	0.076	0.203	9.21E-08	Nccrp1	EpC_Ageddown	EpC
2.04E-96	-0.9240362	0.929	0.995	4.88E-92	Hspa8	EpC_Ageddown	EpC
3.26E-14	-0.9320658	0.804	0.866	7.83E-10	Aldh3a1	EpC_Ageddown	EpC
2.03E-15	-0.9816094	0.111	0.267	4.87E-11	Sostdc1	EpC_Ageddown	EpC
6.02E-43	-1.0180356	0.825	0.971	1.44E-38	Hspb1	EpC_Ageddown	EpC
5.34E-16	-1.0383333	0.413	0.545	1.28E-11	Tmprss11d	EpC_Ageddown	EpC
3.28E-79	-1.0415157	0.988	0.996	7.87E-75	Rps8	EpC_Ageddown	EpC
8.38E-135	-1.0449867	0.985	0.997	2.01E-130	Hsp90ab1	EpC_Ageddown	EpC
2.40E-30	-1.051459	0.693	0.84	5.76E-26	Atp6v1e1	EpC_Ageddown	EpC
1.55E-67	-1.0518208	0.371	0.827	3.72E-63	AY036118	EpC_Ageddown	EpC
2.97E-11	-1.0539937	0.648	0.791	7.13E-07	Krt14	EpC_Ageddown	EpC
3.84E-57	-1.073087	0.444	0.775	9.21E-53	Anxa8	EpC_Ageddown	EpC
1.64E-11	-1.090269	0.366	0.516	3.92E-07	Cnfn	EpC_Ageddown	EpC
6.21E-106	-1.1058091	0.621	0.928	1.49E-101	Dnaja1	EpC_Ageddown	EpC
2.78E-11	-1.1173939	0.3	0.427	6.68E-07	Timp3	EpC_Ageddown	EpC
2.69E-19	-1.1602467	0.287	0.466	6.45E-15	Cst6	EpC_Ageddown	EpC
9.15E-76	-1.1775153	0.96	0.996	2.19E-71	Rps12	EpC_Ageddown	EpC
1.62E-132	-1.3488537	0.705	0.966	3.88E-128	Hsp90aa1	EpC_Ageddown	EpC
4.87E-07	-1.3989556	0.245	0.356	0.011676	Krt16	EpC_Ageddown	EpC
1.16E-21	-1.4089341	0.288	0.476	2.79E-17	Spink5	EpC_Ageddown	EpC
4.26E-07	-1.4463882	0.298	0.405	0.010208	2300002M23Rik	EpC_Ageddown	EpC
6.19E-39	-1.6187913	0.632	0.892	1.49E-34	Krt17	EpC_Ageddown	EpC
1.13E-08	-1.6639895	0.199	0.304	0.000272	Krt75	EpC_Ageddown	EpC
2.29E-119	-1.6770468	0.319	0.831	5.50E-115	Hspa1a	EpC_Ageddown	EpC
1.56E-133	-1.714615	0.541	0.918	3.75E-129	Hspa1b	EpC_Ageddown	EpC
1.72E-12	-2.0711647	0.356	0.466	4.14E-08	Ly6g6c	EpC_Ageddown	EpC
1.51E-12	3.80003135	0.546	0.291	3.61E-08	Tff2	MuC_Agedup	MuC
3.50E-11	3.34821296	0.681	0.571	8.40E-07	Agr2	MuC_Agedup	MuC
5.63E-19	3.22290391	0.38	0.083	1.35E-14	SmMuC	MuC_Agedup	MuC
1.27E-17	2.89663509	0.902	0.812	3.04E-13	Muc5b	MuC_Agedup	MuC

8.39E-09	2.64290454	0.423	0.238	0.000201	Bpifb2	MuC_Agedup	MuC
5.03E-20	2.39073557	0.436	0.114	1.21E-15	AW112010	MuC_Agedup	MuC
1.37E-21	2.30097221	0.963	0.936	3.29E-17	Nupr1	MuC_Agedup	MuC
1.41E-06	2.18125712	0.742	0.776	0.033726	Tmsb10	MuC_Agedup	MuC
4.65E-49	1.80123075	0.982	0.881	1.11E-44	Pglyrp1	MuC_Agedup	MuC
4.28E-39	1.78440347	0.853	0.429	1.03E-34	Pigr	MuC_Agedup	MuC
2.08E-20	1.63361346	0.515	0.158	5.00E-16	Tesc	MuC_Agedup	MuC
1.55E-21	1.52641332	0.785	0.529	3.71E-17	Gfpt1	MuC_Agedup	MuC
2.22E-18	1.5174309	0.417	0.111	5.33E-14	Cgref1	MuC_Agedup	MuC
8.15E-20	1.49888809	0.883	0.856	1.96E-15	Tmed3	MuC_Agedup	MuC
2.31E-24	1.44760686	0.509	0.13	5.53E-20	Galnt6	MuC_Agedup	MuC
1.96E-43	1.43970529	0.963	0.942	4.69E-39	H2-K1	MuC_Agedup	MuC
5.43E-17	1.43395281	0.969	0.931	1.30E-12	Wfdc2	MuC_Agedup	MuC
1.51E-15	1.40995962	0.387	0.108	3.62E-11	Lman1l	MuC_Agedup	MuC
1.06E-32	1.35356897	0.975	0.914	2.55E-28	Ly6e	MuC_Agedup	MuC
1.35E-20	1.34885376	0.804	0.579	3.24E-16	Tspan13	MuC_Agedup	MuC
5.95E-33	1.33044722	0.975	0.939	1.43E-28	H2-D1	MuC_Agedup	MuC
2.10E-27	1.31029678	0.969	0.958	5.04E-23	Ssr4	MuC_Agedup	MuC
5.20E-27	1.2969834	0.975	0.875	1.25E-22	mt-Atp8	MuC_Agedup	MuC
2.23E-15	1.27626498	0.166	0	5.34E-11	Itln1	MuC_Agedup	MuC
6.94E-20	1.27080386	0.748	0.49	1.66E-15	Fkbp11	MuC_Agedup	MuC
1.79E-35	1.25001203	0.871	0.612	4.29E-31	Isg20	MuC_Agedup	MuC
8.95E-23	1.23676785	0.926	0.886	2.15E-18	Rrbp1	MuC_Agedup	MuC
2.13E-32	1.22592248	0.939	0.903	5.11E-28	Sec11c	MuC_Agedup	MuC
1.84E-18	1.2153854	0.81	0.668	4.42E-14	Cpd	MuC_Agedup	MuC
9.52E-16	1.189575	0.663	0.413	2.28E-11	Tgoln1	MuC_Agedup	MuC
5.63E-26	1.18917135	0.914	0.837	1.35E-21	Fkbp2	MuC_Agedup	MuC
3.97E-14	1.18875684	0.595	0.38	9.52E-10	Dnajc10	MuC_Agedup	MuC
9.53E-37	1.18106932	1	0.997	2.28E-32	mt-Nd4l	MuC_Agedup	MuC
1.89E-14	1.17767218	0.38	0.119	4.53E-10	Oit1	MuC_Agedup	MuC
4.83E-17	1.17690327	0.73	0.571	1.16E-12	Snhg18	MuC_Agedup	MuC
4.04E-21	1.17504502	0.908	0.828	9.68E-17	B2m	MuC_Agedup	MuC
2.35E-11	1.11675034	0.822	0.59	5.64E-07	Ifi27l2a	MuC_Agedup	MuC
5.21E-20	1.11123816	0.38	0.069	1.25E-15	Golm1	MuC_Agedup	MuC
6.38E-15	1.10262091	0.387	0.114	1.53E-10	Nkx3-1	MuC_Agedup	MuC
1.30E-52	1.09883322	0.564	0.008	3.11E-48	Gm10260	MuC_Agedup	MuC
2.15E-20	1.09086755	1	0.989	5.15E-16	Gm42418	MuC_Agedup	MuC
1.27E-20	1.08022186	0.73	0.457	3.05E-16	Mfsd4a	MuC_Agedup	MuC
4.17E-28	1.07173531	0.969	0.972	1.00E-23	Ftl1	MuC_Agedup	MuC
5.60E-18	1.05681324	0.902	0.873	1.34E-13	Smim14	MuC_Agedup	MuC
6.91E-13	1.05529814	0.761	0.679	1.66E-08	Tmbim4	MuC_Agedup	MuC
1.59E-18	1.05076355	0.883	0.85	3.82E-14	Cd63	MuC_Agedup	MuC
3.61E-20	1.03938844	0.957	0.95	8.66E-16	Reep5	MuC_Agedup	MuC
1.59E-13	1.02648182	0.914	0.748	3.81E-09	Cst3	MuC_Agedup	MuC
2.39E-24	1.02281479	0.865	0.673	5.74E-20	Npdc1	MuC_Agedup	MuC
4.38E-20	1.01089121	0.908	0.834	1.05E-15	Tm9sf3	MuC_Agedup	MuC
6.43E-24	1.01086673	0.933	0.892	1.54E-19	Krtcap2	MuC_Agedup	MuC
1.05E-17	1.00769003	0.908	0.906	2.53E-13	Rabac1	MuC_Agedup	MuC
3.83E-15	0.99803465	0.706	0.518	9.18E-11	Dap	MuC_Agedup	MuC
1.08E-20	0.98061323	0.859	0.742	2.60E-16	Hdlbp	MuC_Agedup	MuC
1.27E-15	0.96521273	0.853	0.737	3.05E-11	Lrrc26	MuC_Agedup	MuC
7.14E-19	0.95766639	0.742	0.479	1.71E-14	Copz2	MuC_Agedup	MuC
5.96E-26	0.95260275	0.957	0.978	1.43E-21	Sec61g	MuC_Agedup	MuC
1.25E-16	0.94914973	0.595	0.319	2.99E-12	Erlec1	MuC_Agedup	MuC
3.02E-12	0.94470823	0.73	0.582	7.24E-08	Etv1	MuC_Agedup	MuC
6.25E-13	0.92950751	0.607	0.393	1.50E-08	P2rx4	MuC_Agedup	MuC
1.16E-13	0.91926525	0.534	0.285	2.79E-09	Creb3l1	MuC_Agedup	MuC
7.20E-20	0.91781132	0.951	0.975	1.73E-15	Sec61b	MuC_Agedup	MuC
3.63E-21	0.91688022	0.834	0.69	8.69E-17	Txndc5	MuC_Agedup	MuC

2.88E-17	0.91664927	0.546	0.23	6.90E-13	Cldn2	MuC_Agedup	MuC
1.72E-19	0.91269171	0.871	0.726	4.13E-15	Tram1	MuC_Agedup	MuC
8.36E-18	0.90657025	0.933	0.956	2.00E-13	Cd24a	MuC_Agedup	MuC
1.27E-17	0.90566881	0.81	0.657	3.04E-13	Srpr	MuC_Agedup	MuC
9.47E-30	0.89774715	0.969	0.942	2.27E-25	Dad1	MuC_Agedup	MuC
2.00E-19	0.89706376	0.337	0.047	4.79E-15	Chst4	MuC_Agedup	MuC
2.07E-07	0.89534621	0.822	0.834	0.004965	Pdia3	MuC_Agedup	MuC
4.81E-17	0.89248877	0.902	0.898	1.15E-12	Tmbim6	MuC_Agedup	MuC
1.22E-12	0.89058491	0.914	0.875	2.93E-08	Mfge8	MuC_Agedup	MuC
2.25E-10	0.88981231	0.736	0.618	5.39E-06	Lman1	MuC_Agedup	MuC
7.32E-21	0.88911583	0.344	0.044	1.76E-16	Papss2	MuC_Agedup	MuC
4.86E-15	0.88825593	0.822	0.715	1.17E-10	Cd164	MuC_Agedup	MuC
9.96E-16	0.88298756	0.632	0.357	2.39E-11	Scd2	MuC_Agedup	MuC
1.09E-13	0.87987428	0.337	0.094	2.61E-09	Casc4	MuC_Agedup	MuC
1.99E-19	0.87619437	0.798	0.551	4.77E-15	Plet1	MuC_Agedup	MuC
1.54E-16	0.8612339	0.736	0.526	3.69E-12	Smim22	MuC_Agedup	MuC
8.27E-13	0.86047875	0.227	0.03	1.98E-08	Olfm4	MuC_Agedup	MuC
1.26E-20	0.85557436	0.933	0.914	3.01E-16	Tmed10	MuC_Agedup	MuC
7.28E-16	0.85235205	0.828	0.729	1.75E-11	Mydgf	MuC_Agedup	MuC
1.14E-14	0.85113687	0.951	0.97	2.74E-10	Gng5	MuC_Agedup	MuC
4.48E-22	0.84937038	0.982	0.986	1.07E-17	Gm10076	MuC_Agedup	MuC
1.56E-12	0.84187548	0.779	0.734	3.74E-08	Dnajc3	MuC_Agedup	MuC
1.19E-06	0.84186779	0.785	0.806	0.028498	Bag1	MuC_Agedup	MuC
2.82E-13	0.83537064	0.687	0.499	6.75E-09	S100a1	MuC_Agedup	MuC
1.14E-09	0.83442841	0.865	0.873	2.74E-05	Ost4	MuC_Agedup	MuC
5.15E-14	0.83363654	0.896	0.839	1.23E-09	Ostc	MuC_Agedup	MuC
1.53E-11	0.82128578	0.712	0.582	3.67E-07	Golph3	MuC_Agedup	MuC
3.32E-09	0.81904301	0.748	0.748	7.97E-05	Tmem167	MuC_Agedup	MuC
1.79E-10	0.8086255	0.65	0.507	4.30E-06	Mia3	MuC_Agedup	MuC
1.73E-16	0.79566722	0.963	0.92	4.14E-12	Cldn10	MuC_Agedup	MuC
6.32E-12	0.7919772	0.945	0.942	1.51E-07	Epcam	MuC_Agedup	MuC
5.25E-20	0.782073	0.564	0.227	1.26E-15	Galnt4	MuC_Agedup	MuC
4.21E-15	0.78139236	0.896	0.886	1.01E-10	Ndufa1	MuC_Agedup	MuC
5.20E-09	0.78055955	0.779	0.748	0.000125	Tceal9	MuC_Agedup	MuC
1.53E-19	0.77436718	0.264	0.017	3.68E-15	Ceacam10	MuC_Agedup	MuC
4.64E-11	0.77059193	0.779	0.681	1.11E-06	Stard10	MuC_Agedup	MuC
6.22E-13	0.77031293	0.638	0.416	1.49E-08	Atp2a3	MuC_Agedup	MuC
2.83E-13	0.76797433	0.791	0.665	6.79E-09	Rpn2	MuC_Agedup	MuC
4.31E-13	0.76633153	0.712	0.524	1.03E-08	Tspan1	MuC_Agedup	MuC
2.17E-09	0.76298323	0.834	0.845	5.21E-05	App	MuC_Agedup	MuC
1.73E-09	0.75744723	0.699	0.648	4.14E-05	Rpn1	MuC_Agedup	MuC
1.09E-06	0.75658406	0.423	0.283	0.026237	Hexb	MuC_Agedup	MuC
5.24E-16	0.75530809	0.988	0.994	1.26E-11	Serf2	MuC_Agedup	MuC
4.72E-18	0.75267768	0.933	0.881	1.13E-13	Serp1	MuC_Agedup	MuC
3.23E-15	0.74820504	0.613	0.321	7.73E-11	Bace2	MuC_Agedup	MuC
2.45E-15	0.73922113	0.816	0.726	5.87E-11	Creg1	MuC_Agedup	MuC
2.71E-13	0.7356856	0.791	0.654	6.51E-09	Sec61a1	MuC_Agedup	MuC
5.48E-13	0.73056408	0.853	0.767	1.31E-08	Slc12a2	MuC_Agedup	MuC
6.09E-14	0.73045309	0.828	0.679	1.46E-09	Tcn2	MuC_Agedup	MuC
2.65E-11	0.72113751	0.773	0.659	6.35E-07	Cyba	MuC_Agedup	MuC
1.90E-07	0.71980709	0.472	0.321	0.004556	Spdef	MuC_Agedup	MuC
2.05E-21	0.71978535	0.975	0.964	4.93E-17	Spint2	MuC_Agedup	MuC
4.80E-12	0.71894469	0.834	0.798	1.15E-07	Chchd10	MuC_Agedup	MuC
9.84E-19	0.71710876	0.313	0.042	2.36E-14	Scd1	MuC_Agedup	MuC
9.74E-07	0.71651232	0.632	0.54	0.023367	Insig1	MuC_Agedup	MuC
1.81E-09	0.71633563	0.914	0.909	4.34E-05	Hsp90b1	MuC_Agedup	MuC
5.37E-14	0.70999965	0.963	0.909	1.29E-09	Smdt1	MuC_Agedup	MuC
4.65E-14	0.70624753	0.718	0.507	1.11E-09	Gmds	MuC_Agedup	MuC
1.59E-11	0.70503225	0.834	0.792	3.81E-07	Atp2a2	MuC_Agedup	MuC

8.89E-09	0.70215483	0.399	0.208	0.000213	Cracr2a	MuC_Agedup	MuC
8.58E-12	0.70028281	0.515	0.28	2.06E-07	MuCc2	MuC_Agedup	MuC
1.14E-06	0.69908454	0.644	0.587	0.027439	2310039H08Rik	MuC_Agedup	MuC
8.43E-12	0.6969151	0.853	0.801	2.02E-07	Selenos	MuC_Agedup	MuC
3.94E-14	0.6939973	0.828	0.809	9.45E-10	Ifi27	MuC_Agedup	MuC
9.38E-07	0.68711751	0.528	0.36	0.022494	Basp1	MuC_Agedup	MuC
1.85E-07	0.68248989	0.571	0.454	0.004437	Maged1	MuC_Agedup	MuC
5.17E-16	0.6814799	0.503	0.202	1.24E-11	Fcgbp	MuC_Agedup	MuC
1.62E-14	0.681289	0.84	0.684	3.89E-10	Ssr3	MuC_Agedup	MuC
8.72E-12	0.68045715	0.81	0.762	2.09E-07	Dpm3	MuC_Agedup	MuC
1.22E-07	0.68035099	0.564	0.443	0.002937	Tspan3	MuC_Agedup	MuC
1.43E-11	0.67706901	0.644	0.468	3.42E-07	Tmf1	MuC_Agedup	MuC
2.49E-12	0.67263422	0.411	0.166	5.96E-08	Galnt12	MuC_Agedup	MuC
1.48E-14	0.67257526	0.362	0.102	3.54E-10	Crlf1	MuC_Agedup	MuC
8.43E-12	0.67182145	0.466	0.235	2.02E-07	Sel1l3	MuC_Agedup	MuC
3.85E-09	0.67153624	0.613	0.443	9.22E-05	Atp1b1	MuC_Agedup	MuC
3.06E-14	0.6686499	0.73	0.59	7.33E-10	Carhsp1	MuC_Agedup	MuC
4.94E-09	0.66581285	0.963	0.898	0.000118	Ifitm3	MuC_Agedup	MuC
1.01E-10	0.66478218	0.675	0.512	2.42E-06	Slc50a1	MuC_Agedup	MuC
4.50E-13	0.66344924	0.865	0.795	1.08E-08	Ssr2	MuC_Agedup	MuC
1.37E-14	0.66209265	0.706	0.479	3.29E-10	Bst2	MuC_Agedup	MuC
6.49E-23	0.66144582	0.368	0.047	1.56E-18	1600014C10Rik	MuC_Agedup	MuC
3.75E-12	0.65437533	0.816	0.726	8.98E-08	Chpt1	MuC_Agedup	MuC
6.03E-09	0.65226091	0.945	0.953	0.000145	Ppib	MuC_Agedup	MuC
5.87E-17	0.64859313	1	1	1.41E-12	mt-Co1	MuC_Agedup	MuC
1.17E-13	0.64760951	0.834	0.806	2.82E-09	Tmem258	MuC_Agedup	MuC
7.73E-12	0.64699286	0.626	0.421	1.85E-07	Mlph	MuC_Agedup	MuC
3.37E-11	0.64619582	0.89	0.884	8.08E-07	Selenof	MuC_Agedup	MuC
1.10E-12	0.64276872	0.693	0.518	2.64E-08	Surf4	MuC_Agedup	MuC
4.12E-11	0.64058784	0.742	0.695	9.88E-07	H13	MuC_Agedup	MuC
4.53E-14	0.64050915	0.926	0.878	1.09E-09	Selenok	MuC_Agedup	MuC
4.57E-11	0.63858418	0.613	0.418	1.10E-06	Yipf6	MuC_Agedup	MuC
3.05E-12	0.63397696	0.859	0.867	7.31E-08	Ehf	MuC_Agedup	MuC
1.68E-16	0.6297156	0.313	0.055	4.03E-12	Atp2c2	MuC_Agedup	MuC
4.26E-12	0.62907229	0.362	0.122	1.02E-07	Mgat5	MuC_Agedup	MuC
2.18E-11	0.62804385	0.822	0.731	5.22E-07	Ddost	MuC_Agedup	MuC
1.68E-10	0.62718821	0.724	0.582	4.03E-06	Golgb1	MuC_Agedup	MuC
6.47E-12	0.62283273	0.859	0.834	1.55E-07	Ndufv3	MuC_Agedup	MuC
5.40E-15	0.62123776	0.577	0.296	1.29E-10	Rab15	MuC_Agedup	MuC
9.31E-13	0.61916482	1	0.992	2.23E-08	mt-Nd5	MuC_Agedup	MuC
3.83E-11	0.61180119	0.601	0.388	9.18E-07	Slc39a7	MuC_Agedup	MuC
1.80E-09	0.61162374	0.429	0.222	4.30E-05	Acsl3	MuC_Agedup	MuC
1.52E-23	0.60925723	0.472	0.105	3.65E-19	Lgals3bp	MuC_Agedup	MuC
9.85E-14	0.60480876	0.791	0.673	2.36E-09	Ddrgk1	MuC_Agedup	MuC
8.53E-11	0.60315731	0.644	0.452	2.05E-06	Shisa5	MuC_Agedup	MuC
1.16E-08	0.60050035	0.564	0.416	0.000278	Sppl2a	MuC_Agedup	MuC
1.08E-23	0.59980363	0.258	0	2.59E-19	Xist	MuC_Agedup	MuC
2.39E-12	0.59688409	0.755	0.643	5.73E-08	Mrps28	MuC_Agedup	MuC
2.00E-09	0.59635404	0.699	0.565	4.80E-05	Furin	MuC_Agedup	MuC
1.40E-09	0.59556885	0.865	0.87	3.36E-05	Manf	MuC_Agedup	MuC
7.54E-10	0.59522114	0.699	0.557	1.81E-05	Kcnn4	MuC_Agedup	MuC
1.65E-11	0.59176124	0.73	0.609	3.96E-07	Azin1	MuC_Agedup	MuC
6.87E-07	0.58808958	0.871	0.906	0.016485	Spcs1	MuC_Agedup	MuC
9.08E-10	0.57182187	0.577	0.416	2.18E-05	Ergic1	MuC_Agedup	MuC
5.82E-12	0.56890989	0.534	0.288	1.40E-07	Gale	MuC_Agedup	MuC
1.46E-06	0.56799966	0.834	0.87	0.03494	Cope	MuC_Agedup	MuC
1.26E-09	0.56752845	0.945	0.928	3.03E-05	Uqcrq	MuC_Agedup	MuC
5.23E-09	0.56670406	0.466	0.291	0.000125	Galnt3	MuC_Agedup	MuC
1.57E-07	0.56630711	0.724	0.651	0.003774	Cmas	MuC_Agedup	MuC

6.59E-09	0.56548132	0.153	0.022	0.000158	Edn2	MuC_Agedup	MuC
1.01E-13	0.56271228	0.546	0.258	2.42E-09	Rhoc	MuC_Agedup	MuC
3.75E-08	0.56253684	0.761	0.717	0.000899	Oat	MuC_Agedup	MuC
7.36E-10	0.56110945	0.436	0.227	1.76E-05	Retreg1	MuC_Agedup	MuC
2.45E-08	0.55204816	0.62	0.485	0.000588	Vkorc1	MuC_Agedup	MuC
4.92E-11	0.54879303	0.503	0.291	1.18E-06	Slc38a1	MuC_Agedup	MuC
3.33E-10	0.54515514	0.656	0.499	7.97E-06	Spcs3	MuC_Agedup	MuC
3.02E-08	0.54444637	0.613	0.482	0.000724	Sec31a	MuC_Agedup	MuC
3.21E-09	0.54139356	0.908	0.884	7.69E-05	Nme1	MuC_Agedup	MuC
1.65E-16	0.53875154	0.497	0.205	3.96E-12	Mansc1	MuC_Agedup	MuC
8.37E-07	0.53541207	0.853	0.859	0.02007	Cnpy2	MuC_Agedup	MuC
1.12E-10	0.53356632	0.509	0.294	2.68E-06	Gne	MuC_Agedup	MuC
1.51E-08	0.53223589	0.589	0.421	0.000362	Asph	MuC_Agedup	MuC
1.80E-10	0.53093699	0.442	0.202	4.31E-06	Prss32	MuC_Agedup	MuC
1.63E-09	0.52891387	0.65	0.515	3.91E-05	Sec23b	MuC_Agedup	MuC
5.41E-09	0.52247191	0.448	0.255	0.00013	Txndc12	MuC_Agedup	MuC
3.23E-09	0.51986102	0.822	0.765	7.75E-05	Nenf	MuC_Agedup	MuC
1.77E-11	0.51591933	0.742	0.64	4.24E-07	Msrb1	MuC_Agedup	MuC
3.99E-10	0.51311676	0.613	0.432	9.58E-06	Pmm2	MuC_Agedup	MuC
4.95E-09	0.5116548	0.816	0.756	0.000119	Ufm1	MuC_Agedup	MuC
1.63E-12	0.510016	0.27	0.058	3.91E-08	Stc2	MuC_Agedup	MuC
5.80E-07	0.506889	0.822	0.781	0.013917	Tmem176a	MuC_Agedup	MuC
4.32E-08	0.50397482	0.632	0.504	0.001035	Itm2c	MuC_Agedup	MuC
9.01E-10	0.49761471	0.908	0.873	2.16E-05	Ndufb7	MuC_Agedup	MuC
3.68E-10	0.49301051	0.601	0.391	8.83E-06	Timp2	MuC_Agedup	MuC
2.48E-09	0.49258384	0.822	0.776	5.95E-05	Ndufa3	MuC_Agedup	MuC
2.19E-10	0.49033912	0.466	0.244	5.26E-06	Slc39a11	MuC_Agedup	MuC
2.95E-07	0.49027038	0.926	0.964	0.007066	Atp5g1	MuC_Agedup	MuC
1.68E-12	0.4897836	0.822	0.737	4.02E-08	Dnajc19	MuC_Agedup	MuC
1.06E-09	0.48334805	0.589	0.418	2.55E-05	Edem2	MuC_Agedup	MuC
2.56E-08	0.47978958	0.595	0.44	0.000615	Prom1	MuC_Agedup	MuC
8.63E-12	0.47966558	0.926	0.931	2.07E-07	Atp6v1g1	MuC_Agedup	MuC
1.47E-09	0.47793921	0.92	0.889	3.54E-05	Ndufb8	MuC_Agedup	MuC
7.79E-15	0.47284172	0.227	0.022	1.87E-10	Muc16	MuC_Agedup	MuC
1.45E-17	0.47258498	0.442	0.133	3.47E-13	Ccl28	MuC_Agedup	MuC
1.62E-09	0.47042928	0.485	0.296	3.89E-05	Arfgef3	MuC_Agedup	MuC
1.55E-09	0.46976313	0.503	0.299	3.73E-05	Slc17a5	MuC_Agedup	MuC
1.80E-09	0.46953148	0.472	0.271	4.31E-05	Slc44a4	MuC_Agedup	MuC
1.23E-08	0.46872222	0.663	0.507	0.000295	Tvp23b	MuC_Agedup	MuC
1.71E-07	0.46736092	0.325	0.15	0.004104	Tcea3	MuC_Agedup	MuC
3.57E-07	0.4650115	0.736	0.626	0.008563	Alg5	MuC_Agedup	MuC
1.31E-08	0.4601327	0.374	0.183	0.000314	Creb3l4	MuC_Agedup	MuC
1.19E-07	0.46011555	0.589	0.465	0.002864	Mcfd2	MuC_Agedup	MuC
1.91E-06	0.4597324	0.656	0.568	0.045711	Tmem205	MuC_Agedup	MuC
2.16E-15	0.45863253	0.307	0.058	5.18E-11	Slc12a8	MuC_Agedup	MuC
5.57E-11	0.45832039	0.583	0.357	1.34E-06	Setd5	MuC_Agedup	MuC
6.18E-11	0.45706674	0.515	0.285	1.48E-06	Tmc5	MuC_Agedup	MuC
1.90E-06	0.45692264	0.405	0.255	0.045473	Foxa1	MuC_Agedup	MuC
1.34E-06	0.45681675	0.497	0.343	0.032123	Cmtm8	MuC_Agedup	MuC
3.72E-07	0.45478459	0.638	0.543	0.008912	Nucb2	MuC_Agedup	MuC
1.20E-07	0.45232962	0.448	0.285	0.002877	Hook1	MuC_Agedup	MuC
2.45E-07	0.45221377	0.939	0.934	0.005883	Gabarap	MuC_Agedup	MuC
7.03E-08	0.45211021	0.656	0.573	0.001685	Vmp1	MuC_Agedup	MuC
1.42E-09	0.45010607	0.491	0.28	3.40E-05	Appl2	MuC_Agedup	MuC
2.45E-08	0.44958309	0.411	0.219	0.000587	Hexa	MuC_Agedup	MuC
5.77E-11	0.44912113	0.258	0.058	1.38E-06	Chst5	MuC_Agedup	MuC
1.34E-08	0.44796947	0.613	0.457	0.000322	Stt3b	MuC_Agedup	MuC
1.64E-08	0.44728057	0.515	0.33	0.000393	Fndc3b	MuC_Agedup	MuC
2.59E-08	0.44711264	0.577	0.432	0.00062	Man2a1	MuC_Agedup	MuC

1.00E-06	0.44548411	0.571	0.435	0.023971	Tmem30a	MuC_Agedup	MuC
1.11E-06	0.44470407	0.669	0.557	0.026505	Asns	MuC_Agedup	MuC
8.82E-08	0.44445574	0.791	0.742	0.002114	Sdcbp	MuC_Agedup	MuC
2.13E-07	0.44378572	0.761	0.676	0.005115	Arpc1b	MuC_Agedup	MuC
5.17E-08	0.44362176	0.859	0.892	0.001239	Ndufa2	MuC_Agedup	MuC
1.12E-07	0.44127472	0.926	0.9	0.002698	Ifitm2	MuC_Agedup	MuC
1.14E-06	0.44076405	0.81	0.765	0.027264	Cldn3	MuC_Agedup	MuC
8.74E-09	0.43870802	0.785	0.72	0.00021	Jtb	MuC_Agedup	MuC
5.59E-09	0.43765294	0.454	0.244	0.000134	Cgnl1	MuC_Agedup	MuC
9.06E-13	0.43729481	0.393	0.133	2.17E-08	Dkk3	MuC_Agedup	MuC
1.91E-08	0.43471822	0.571	0.368	0.000458	Tmem9b	MuC_Agedup	MuC
1.19E-06	0.43191386	0.393	0.238	0.028508	Kdelr3	MuC_Agedup	MuC
1.29E-06	0.4318875	0.84	0.842	0.030871	Romo1	MuC_Agedup	MuC
1.62E-08	0.43149486	0.466	0.285	0.000389	Sil1	MuC_Agedup	MuC
3.68E-09	0.42884454	0.282	0.097	8.83E-05	Lrg1	MuC_Agedup	MuC
1.45E-10	0.42837813	0.399	0.177	3.47E-06	Twf2	MuC_Agedup	MuC
7.93E-10	0.42698202	0.926	0.956	1.90E-05	Rpl36al	MuC_Agedup	MuC
1.45E-08	0.4246894	1	0.994	0.000348	Rpl38	MuC_Agedup	MuC
5.98E-07	0.42299951	0.485	0.324	0.014352	Lasp1	MuC_Agedup	MuC
8.83E-07	0.42228926	0.466	0.332	0.02118	Pofut2	MuC_Agedup	MuC
1.05E-06	0.41922234	0.595	0.463	0.025234	Kcnk1	MuC_Agedup	MuC
2.32E-07	0.41412847	0.939	0.917	0.005555	Ndufa13	MuC_Agedup	MuC
1.53E-07	0.41353189	0.613	0.468	0.003665	Prr15l	MuC_Agedup	MuC
6.40E-16	0.41287901	0.172	0	1.54E-11	Gm21718	MuC_Agedup	MuC
2.06E-06	0.41046996	0.816	0.767	0.049386	Rex1bd	MuC_Agedup	MuC
2.89E-07	0.40906514	0.84	0.778	0.006923	Tmem238	MuC_Agedup	MuC
9.43E-07	0.4063606	0.503	0.36	0.022622	Edem1	MuC_Agedup	MuC
9.68E-08	0.40304693	0.865	0.906	0.002321	Srp9	MuC_Agedup	MuC
7.38E-08	0.40172151	0.699	0.587	0.001771	Tmem160	MuC_Agedup	MuC
6.21E-14	0.40024254	0.27	0.047	1.49E-09	Vwf	MuC_Agedup	MuC
7.15E-07	0.39877041	0.607	0.501	0.017154	Tm2d1	MuC_Agedup	MuC
8.63E-08	0.3984737	0.313	0.136	0.00207	Enho	MuC_Agedup	MuC
3.62E-07	0.39641963	0.902	0.878	0.008692	Pebp1	MuC_Agedup	MuC
1.53E-14	0.39622741	0.252	0.033	3.66E-10	Bcas1	MuC_Agedup	MuC
1.08E-11	0.39467087	0.466	0.222	2.60E-07	Hid1	MuC_Agedup	MuC
5.72E-07	0.39247163	0.393	0.241	0.013721	Tmem263	MuC_Agedup	MuC
9.96E-07	0.39004648	0.344	0.183	0.023886	Slc38a10	MuC_Agedup	MuC
3.29E-09	0.38949748	0.982	0.942	7.89E-05	Ndufb9	MuC_Agedup	MuC
2.99E-15	0.38695388	0.258	0.033	7.18E-11	Bpifb6	MuC_Agedup	MuC
2.45E-08	0.38459931	0.442	0.249	0.000587	Sec16a	MuC_Agedup	MuC
2.09E-09	0.38403111	0.54	0.332	5.02E-05	Mmp15	MuC_Agedup	MuC
1.51E-07	0.38268878	0.914	0.936	0.003612	Spcs2	MuC_Agedup	MuC
4.60E-08	0.38080996	0.577	0.393	0.001102	Cd82	MuC_Agedup	MuC
5.66E-08	0.37937162	0.466	0.288	0.001358	Gns	MuC_Agedup	MuC
7.81E-08	0.37648467	0.595	0.443	0.001874	Tmed7	MuC_Agedup	MuC
2.65E-08	0.37499591	0.479	0.305	0.000635	Plpp5	MuC_Agedup	MuC
1.72E-07	0.37375208	0.908	0.889	0.004115	Lamtor2	MuC_Agedup	MuC
1.69E-06	0.37103284	0.798	0.759	0.040588	Eny2	MuC_Agedup	MuC
5.24E-07	0.36729771	0.92	0.909	0.012566	Xbp1	MuC_Agedup	MuC
9.67E-07	0.36714282	0.847	0.892	0.02319	Ndufa11	MuC_Agedup	MuC
2.29E-07	0.36711177	0.405	0.211	0.005493	Psmb8	MuC_Agedup	MuC
1.83E-16	0.36425672	0.178	0	4.40E-12	Scgb1b3	MuC_Agedup	MuC
9.84E-07	0.3638684	0.491	0.324	0.023601	Atp13a2	MuC_Agedup	MuC
4.65E-08	0.36147212	0.945	0.936	0.001116	Ndufa7	MuC_Agedup	MuC
2.27E-07	0.35715804	0.521	0.349	0.005434	D17Wsu92e	MuC_Agedup	MuC
1.93E-09	0.35687422	0.368	0.166	4.63E-05	Ttc39a	MuC_Agedup	MuC
1.55E-11	0.35642934	0.442	0.202	3.72E-07	Smim1	MuC_Agedup	MuC
6.03E-13	0.35506691	0.331	0.094	1.45E-08	Pdia5	MuC_Agedup	MuC
4.00E-07	0.34828601	0.239	0.086	0.009588	Pf4	MuC_Agedup	MuC

3.51E-14	0.33843345	0.227	0.025	8.41E-10	Clec14a	MuC_Agedup	MuC
2.74E-07	0.33795963	0.515	0.346	0.006564	Tmem248	MuC_Agedup	MuC
9.24E-12	0.33666162	0.19	0.022	2.21E-07	Slc38a5	MuC_Agedup	MuC
1.98E-15	0.3360145	0.276	0.042	4.74E-11	H2-Q7	MuC_Agedup	MuC
4.14E-07	0.3357233	0.583	0.438	0.009924	Malsu1	MuC_Agedup	MuC
1.43E-06	0.33389998	0.374	0.213	0.03426	Inafm1	MuC_Agedup	MuC
2.58E-15	0.3308196	0.252	0.03	6.19E-11	Steap1	MuC_Agedup	MuC
4.57E-07	0.3280765	0.509	0.341	0.010953	Slc5a8	MuC_Agedup	MuC
1.83E-07	0.32627174	0.387	0.219	0.004387	Aldh18a1	MuC_Agedup	MuC
2.10E-08	0.3226585	0.374	0.188	0.000503	Ogfod3	MuC_Agedup	MuC
1.02E-06	0.32237695	0.393	0.224	0.024572	3110040N11Rik	MuC_Agedup	MuC
2.33E-13	0.31786241	0.233	0.03	5.59E-09	Syt7	MuC_Agedup	MuC
2.38E-17	0.31088864	0.233	0.014	5.71E-13	BC030870	MuC_Agedup	MuC
2.56E-10	0.31023962	0.301	0.094	6.13E-06	Cpq	MuC_Agedup	MuC
2.19E-09	0.30998138	0.331	0.122	5.26E-05	F5	MuC_Agedup	MuC
5.32E-12	0.30703801	0.227	0.039	1.28E-07	Irf8	MuC_Agedup	MuC
3.02E-11	0.30607298	0.153	0.011	7.25E-07	Pla2g10	MuC_Agedup	MuC
1.77E-06	0.29716444	0.374	0.219	0.042472	Slc35a3	MuC_Agedup	MuC
1.77E-07	0.29512163	0.963	0.958	0.004243	Krt18	MuC_Agedup	MuC
4.45E-07	0.29240292	0.307	0.144	0.010674	Izumo4	MuC_Agedup	MuC
1.10E-07	0.29222464	0.227	0.075	0.00264	Padi2	MuC_Agedup	MuC
2.95E-07	0.28210642	0.411	0.244	0.007066	Ica1	MuC_Agedup	MuC
1.80E-06	0.27806572	0.215	0.075	0.043184	Liph	MuC_Agedup	MuC
2.22E-07	0.27533657	0.178	0.044	0.005314	Osr2	MuC_Agedup	MuC
1.89E-08	0.26542202	0.245	0.08	0.000454	Gpd1	MuC_Agedup	MuC
8.28E-07	0.25629174	0.245	0.094	0.019846	Fsd1l	MuC_Agedup	MuC
1.76E-14	0.2524954	0.215	0.019	4.23E-10	Csprs	MuC_Agedup	MuC
5.62E-08	0.24750055	0.344	0.155	0.001347	Snhg9	MuC_Agedup	MuC
1.92E-08	0.23834051	0.135	0.017	0.000461	2210404E10Rik	MuC_Agedup	MuC
5.19E-12	0.23363675	0.166	0.011	1.24E-07	Galnt5	MuC_Agedup	MuC
5.70E-11	0.22972497	0.16	0.014	1.37E-06	Atp7b	MuC_Agedup	MuC
1.54E-06	0.22867931	0.301	0.141	0.036875	Enpp5	MuC_Agedup	MuC
1.14E-12	0.22743441	0.153	0.006	2.72E-08	Cdkn2a	MuC_Agedup	MuC
1.81E-08	0.22649774	0.233	0.069	0.000433	Gm8797	MuC_Agedup	MuC
3.86E-10	0.22621746	0.209	0.042	9.25E-06	Slc41a2	MuC_Agedup	MuC
2.83E-07	0.22567473	0.147	0.371	0.006796	Lor	MuC_Agedup	MuC
1.90E-06	0.22281142	0.245	0.1	0.045676	1500011B03Rik	MuC_Agedup	MuC
4.34E-10	0.2225837	0.147	0.014	1.04E-05	Ildr2	MuC_Agedup	MuC
4.05E-07	0.21988376	0.264	0.105	0.009714	2900076A07Rik	MuC_Agedup	MuC
1.70E-12	0.21965162	0.19	0.019	4.08E-08	1700066B19Rik	MuC_Agedup	MuC
3.61E-10	0.2194866	0.141	0.011	8.65E-06	Tmem213	MuC_Agedup	MuC
7.69E-09	0.21576557	0.215	0.053	0.000185	Cd59a	MuC_Agedup	MuC
1.02E-08	0.21324975	0.19	0.042	0.000246	H2-Q2	MuC_Agedup	MuC
3.94E-07	0.21265117	0.221	0.072	0.009449	Ephx4	MuC_Agedup	MuC
1.83E-07	0.20454447	0.184	0.047	0.004388	Steap2	MuC_Agedup	MuC
6.30E-09	0.20089938	0.141	0.017	0.000151	Lox	MuC_Agedup	MuC
3.62E-07	-0.2016933	0.141	0.371	0.008687	Gar1	MuC_Ageddown	MuC
4.96E-07	-0.205565	0.202	0.454	0.011884	Tnrc6c	MuC_Ageddown	MuC
2.69E-07	-0.2096429	0.08	0.288	0.006439	Jag1	MuC_Ageddown	MuC
3.35E-08	-0.2103999	0.055	0.263	0.000802	Hlf	MuC_Ageddown	MuC
3.70E-08	-0.2111448	0.172	0.446	0.000887	Tef	MuC_Ageddown	MuC
4.77E-09	-0.2127797	0.025	0.23	0.000114	Ppp1r14c	MuC_Ageddown	MuC
1.17E-06	-0.2136173	0.11	0.316	0.028148	Bcam	MuC_Ageddown	MuC
2.98E-07	-0.2155004	0.104	0.319	0.007156	Pard6g	MuC_Ageddown	MuC
7.82E-07	-0.2192679	0.19	0.413	0.018761	Ethe1	MuC_Ageddown	MuC
8.46E-07	-0.2218951	0.067	0.258	0.020281	Cdc25b	MuC_Ageddown	MuC
3.07E-07	-0.227973	0.025	0.188	0.007365	Tprg	MuC_Ageddown	MuC
5.96E-08	-0.2372239	0.196	0.457	0.00143	Mphosph10	MuC_Ageddown	MuC
1.81E-10	-0.2374183	0.055	0.316	4.35E-06	Banp	MuC_Ageddown	MuC

2.75E-09	-0.2377631	0.098	0.357	6.59E-05	Rrs1	MuC_Ageddown	MuC
1.88E-06	-0.237837	0.067	0.241	0.045171	Ephx3	MuC_Ageddown	MuC
5.59E-07	-0.2412137	0.282	0.554	0.013403	Actr10	MuC_Ageddown	MuC
1.94E-06	-0.2414103	0.037	0.194	0.046607	Gm3776	MuC_Ageddown	MuC
5.29E-07	-0.2422957	0.11	0.316	0.012697	Ltbp4	MuC_Ageddown	MuC
2.04E-08	-0.2424979	0.178	0.457	0.000488	Lamtor3	MuC_Ageddown	MuC
1.55E-06	-0.2433788	0.147	0.355	0.037078	Rsl24d1	MuC_Ageddown	MuC
5.24E-08	-0.2484862	0.135	0.368	0.001258	Bag2	MuC_Ageddown	MuC
1.83E-11	-0.2495991	0.086	0.385	4.38E-07	Sertad1	MuC_Ageddown	MuC
2.18E-08	-0.251059	0.092	0.327	0.000523	Csrp2	MuC_Ageddown	MuC
3.97E-10	-0.2523528	0	0.208	9.52E-06	Cyp3a13	MuC_Ageddown	MuC
2.11E-07	-0.2559317	0.08	0.291	0.005068	Dsg3	MuC_Ageddown	MuC
4.37E-08	-0.2578708	0.141	0.388	0.001048	Polr2h	MuC_Ageddown	MuC
1.40E-07	-0.2603812	0.067	0.277	0.003363	Cavin3	MuC_Ageddown	MuC
9.15E-08	-0.2604505	0.061	0.26	0.002194	Cd109	MuC_Ageddown	MuC
2.63E-07	-0.2630567	0.129	0.355	0.006318	Xpa	MuC_Ageddown	MuC
1.15E-08	-0.2659987	0.037	0.252	0.000276	Vim	MuC_Ageddown	MuC
1.48E-10	-0.2663748	0.117	0.421	3.55E-06	Nr1d1	MuC_Ageddown	MuC
1.88E-07	-0.2665311	0.16	0.404	0.004507	Notch1	MuC_Ageddown	MuC
3.68E-08	-0.268003	0.123	0.352	0.000882	Mrps6	MuC_Ageddown	MuC
8.52E-08	-0.2688155	0.061	0.269	0.002044	Plxdc2	MuC_Ageddown	MuC
1.71E-09	-0.2721634	0.025	0.238	4.11E-05	Snai2	MuC_Ageddown	MuC
1.29E-07	-0.2735424	0.067	0.28	0.003102	Alox12b	MuC_Ageddown	MuC
1.49E-06	-0.2739541	0.288	0.512	0.035844	Tsc22d3	MuC_Ageddown	MuC
2.80E-08	-0.2758054	0.442	0.767	0.000671	U2af1	MuC_Ageddown	MuC
1.32E-08	-0.2776233	0.239	0.535	0.000317	Snw1	MuC_Ageddown	MuC
4.77E-09	-0.2784889	0.086	0.324	0.000114	Serpinh1	MuC_Ageddown	MuC
3.08E-08	-0.2792792	0.166	0.413	0.000739	Tbc1d10a	MuC_Ageddown	MuC
6.39E-08	-0.2805212	0.521	0.812	0.001532	Pax1	MuC_Ageddown	MuC
1.44E-08	-0.2816357	0.141	0.391	0.000344	Marcksl1	MuC_Ageddown	MuC
6.54E-07	-0.2820036	0.08	0.271	0.015694	Nebl	MuC_Ageddown	MuC
3.12E-07	-0.2822222	0.331	0.593	0.007475	Tshz2	MuC_Ageddown	MuC
5.52E-07	-0.2857176	0.178	0.407	0.013226	Map3k20	MuC_Ageddown	MuC
1.82E-07	-0.2879077	0.129	0.371	0.004374	2610528A11Rik	MuC_Ageddown	MuC
7.44E-08	-0.2896625	0.006	0.172	0.001785	Sostdc1	MuC_Ageddown	MuC
1.58E-06	-0.2924771	0.006	0.141	0.037933	S100a8	MuC_Ageddown	MuC
2.04E-06	-0.294983	0.006	0.141	0.049034	Cxcl2	MuC_Ageddown	MuC
7.94E-09	-0.298469	0.012	0.205	0.00019	Il1a	MuC_Ageddown	MuC
5.93E-09	-0.2988985	0.055	0.269	0.000142	Crlf3	MuC_Ageddown	MuC
9.69E-08	-0.2991492	0.074	0.277	0.002325	Efna3	MuC_Ageddown	MuC
7.14E-07	-0.3039334	0.399	0.623	0.017113	Luc7l3	MuC_Ageddown	MuC
1.68E-06	-0.3057107	0.552	0.778	0.040279	Top1	MuC_Ageddown	MuC
8.31E-08	-0.3057655	0.08	0.291	0.001992	Maf	MuC_Ageddown	MuC
3.71E-07	-0.3072231	0.11	0.319	0.008885	Tubb2a	MuC_Ageddown	MuC
9.11E-07	-0.3082135	0.497	0.745	0.021839	Cct5	MuC_Ageddown	MuC
5.80E-10	-0.30919	0.11	0.388	1.39E-05	Dapl1	MuC_Ageddown	MuC
5.91E-07	-0.3104928	0.049	0.222	0.014163	Sparc	MuC_Ageddown	MuC
1.78E-07	-0.3119037	0.239	0.482	0.004269	Cldnd1	MuC_Ageddown	MuC
5.17E-07	-0.3141773	0.393	0.64	0.012394	Acadl	MuC_Ageddown	MuC
1.32E-07	-0.3222538	0.123	0.352	0.003156	Bcl11b	MuC_Ageddown	MuC
5.58E-07	-0.325945	0.276	0.521	0.013372	Sox4	MuC_Ageddown	MuC
5.87E-11	-0.331449	0.135	0.435	1.41E-06	Chordc1	MuC_Ageddown	MuC
1.54E-06	-0.3335418	0.11	0.313	0.03695	Tgm1	MuC_Ageddown	MuC
3.26E-07	-0.334229	0.552	0.806	0.007808	Eif3e	MuC_Ageddown	MuC
6.05E-09	-0.3349914	0	0.183	0.000145	Col3a1	MuC_Ageddown	MuC
1.41E-07	-0.3386003	0.067	0.269	0.003381	Rdh12	MuC_Ageddown	MuC
9.23E-11	-0.3399947	0.221	0.543	2.21E-06	Mettl23	MuC_Ageddown	MuC
9.70E-08	-0.3414778	0.08	0.296	0.002325	Ces1f	MuC_Ageddown	MuC
1.30E-10	-0.3485525	0.184	0.476	3.11E-06	Apoe	MuC_Ageddown	MuC

3.79E-10	-0.3491481	0.092	0.357	9.09E-06	RMuCc	MuC_Ageddown	MuC
5.21E-08	-0.3494127	0.258	0.51	0.001251	Stip1	MuC_Ageddown	MuC
2.17E-10	-0.3504115	0.221	0.554	5.21E-06	Stmn1	MuC_Ageddown	MuC
2.96E-09	-0.3504243	0.104	0.366	7.09E-05	Aldh3b2	MuC_Ageddown	MuC
6.32E-08	-0.3511317	0.405	0.687	0.001516	Ech1	MuC_Ageddown	MuC
3.08E-07	-0.3515405	0.724	0.87	0.007381	Anxa5	MuC_Ageddown	MuC
4.02E-08	-0.352991	0.012	0.191	0.000965	Fst	MuC_Ageddown	MuC
4.09E-07	-0.3557078	0.239	0.46	0.00981	Klc3	MuC_Ageddown	MuC
6.70E-10	-0.3557943	0.067	0.313	1.61E-05	Arap2	MuC_Ageddown	MuC
1.58E-07	-0.3588401	0.638	0.884	0.003778	Phlda1	MuC_Ageddown	MuC
9.78E-12	-0.3621704	0.08	0.374	2.35E-07	Serpine2	MuC_Ageddown	MuC
2.43E-09	-0.3652822	0.104	0.355	5.84E-05	Tnfaip8	MuC_Ageddown	MuC
1.04E-08	-0.3662924	0.307	0.598	0.000251	Cct3	MuC_Ageddown	MuC
7.69E-07	-0.3675194	0.055	0.233	0.018441	Timp3	MuC_Ageddown	MuC
1.94E-08	-0.3706696	0.448	0.723	0.000464	Cct4	MuC_Ageddown	MuC
5.32E-08	-0.3725207	0.067	0.277	0.001276	Cldn4	MuC_Ageddown	MuC
1.50E-06	-0.3782316	0.871	0.978	0.036074	Taldo1	MuC_Ageddown	MuC
1.79E-08	-0.3868071	0.123	0.38	0.00043	Plb1	MuC_Ageddown	MuC
9.75E-08	-0.3880131	0.196	0.454	0.002337	Dsc3	MuC_Ageddown	MuC
6.29E-07	-0.3884459	0	0.139	0.015089	S100a9	MuC_Ageddown	MuC
4.79E-09	-0.3885324	0.11	0.363	0.000115	Car13	MuC_Ageddown	MuC
1.93E-08	-0.3916177	0.006	0.183	0.000463	Upk3a	MuC_Ageddown	MuC
2.17E-10	-0.3954195	0.129	0.416	5.20E-06	Klk11	MuC_Ageddown	MuC
2.52E-09	-0.3976368	0.117	0.374	6.04E-05	Serpinb3a	MuC_Ageddown	MuC
3.34E-08	-0.3995902	0.436	0.704	0.000802	Mt2	MuC_Ageddown	MuC
1.55E-09	-0.4014378	0.178	0.443	3.72E-05	Hebp2	MuC_Ageddown	MuC
5.09E-11	-0.4022492	0.061	0.332	1.22E-06	Il33	MuC_Ageddown	MuC
4.49E-07	-0.40446	0.975	0.989	0.010774	Actg1	MuC_Ageddown	MuC
3.70E-07	-0.406038	0.166	0.393	0.008871	Asprv1	MuC_Ageddown	MuC
1.25E-09	-0.4075847	0.35	0.657	3.00E-05	Lmo4	MuC_Ageddown	MuC
5.71E-07	-0.4097026	0.423	0.643	0.013683	Sox2	MuC_Ageddown	MuC
2.49E-09	-0.4120794	0.031	0.249	5.97E-05	Gng13	MuC_Ageddown	MuC
8.95E-10	-0.4157245	0.288	0.596	2.15E-05	Ces1d	MuC_Ageddown	MuC
2.15E-08	-0.4157987	0.092	0.321	0.000516	Dsg1a	MuC_Ageddown	MuC
1.61E-16	-0.4186781	0.178	0.576	3.86E-12	Gsta2	MuC_Ageddown	MuC
6.74E-07	-0.4199307	0.166	0.391	0.016164	Clic3	MuC_Ageddown	MuC
2.85E-12	-0.4203895	0.08	0.374	6.85E-08	Foxe1	MuC_Ageddown	MuC
1.49E-07	-0.4221375	0.834	0.992	0.003565	Lipf	MuC_Ageddown	MuC
2.47E-08	-0.4269086	0.166	0.418	0.000592	Sult2b1	MuC_Ageddown	MuC
8.50E-11	-0.4294705	0.043	0.302	2.04E-06	Fetub	MuC_Ageddown	MuC
1.12E-10	-0.430002	0.264	0.615	2.68E-06	Capg	MuC_Ageddown	MuC
8.53E-10	-0.4443795	0.08	0.33	2.04E-05	Endou	MuC_Ageddown	MuC
5.57E-07	-0.444428	0.663	0.839	0.013357	Ralbp1	MuC_Ageddown	MuC
2.70E-07	-0.4477958	0.184	0.402	0.00647	Pir	MuC_Ageddown	MuC
8.90E-11	-0.4514191	0.092	0.377	2.13E-06	Krt32	MuC_Ageddown	MuC
2.89E-10	-0.4527352	0.429	0.717	6.93E-06	MuClm	MuC_Ageddown	MuC
6.15E-07	-0.4534958	0.847	0.945	0.014756	Hspe1	MuC_Ageddown	MuC
5.40E-09	-0.4597082	0.466	0.72	0.000129	Hnrnpa1	MuC_Ageddown	MuC
2.03E-07	-0.4672815	0.479	0.701	0.004874	Avpi1	MuC_Ageddown	MuC
1.48E-07	-0.4678745	1	0.992	0.003557	Rps14	MuC_Ageddown	MuC
1.38E-07	-0.4680872	0.994	0.994	0.003307	Rps21	MuC_Ageddown	MuC
2.17E-15	-0.4687491	0.282	0.734	5.20E-11	AY036118	MuC_Ageddown	MuC
1.33E-10	-0.4700309	0.074	0.332	3.19E-06	Mall	MuC_Ageddown	MuC
4.83E-09	-0.4750462	0.135	0.38	0.000116	Pim1	MuC_Ageddown	MuC
3.59E-08	-0.4755899	0.552	0.776	0.000861	2010111I01Rik	MuC_Ageddown	MuC
4.26E-11	-0.4788801	0.067	0.327	1.02E-06	Ifi202b	MuC_Ageddown	MuC
1.85E-14	-0.4795138	0.313	0.704	4.44E-10	Gadd45b	MuC_Ageddown	MuC
2.94E-08	-0.4823539	0.356	0.598	0.000705	Nfe2l2	MuC_Ageddown	MuC
5.71E-11	-0.4914982	0.294	0.612	1.37E-06	Cnih4	MuC_Ageddown	MuC

5.26E-08	-0.4918883	0.209	0.44	0.001261	Tpm2	MuC_Ageddown	MuC
1.79E-10	-0.4948475	0.123	0.399	4.29E-06	Mafb	MuC_Ageddown	MuC
8.20E-07	-0.4964975	0.632	0.789	0.019667	Map1lc3a	MuC_Ageddown	MuC
5.13E-08	-0.4999301	1	0.994	0.001231	Rplp1	MuC_Ageddown	MuC
1.28E-09	-0.502545	0.153	0.438	3.07E-05	Evpl	MuC_Ageddown	MuC
5.24E-08	-0.5028249	0.184	0.41	0.001257	2200002D01Rik	MuC_Ageddown	MuC
7.02E-11	-0.5037431	0.104	0.385	1.68E-06	Pdlim2	MuC_Ageddown	MuC
1.79E-12	-0.5073978	0.417	0.737	4.30E-08	Hnrnpa0	MuC_Ageddown	MuC
2.24E-13	-0.507793	0.061	0.371	5.38E-09	Rpgrip1	MuC_Ageddown	MuC
1.37E-06	-0.5098114	0.957	0.967	0.032792	Rpl12	MuC_Ageddown	MuC
1.01E-10	-0.5116094	0.123	0.407	2.41E-06	Cysrt1	MuC_Ageddown	MuC
1.24E-09	-0.5157825	0.11	0.366	2.96E-05	Dcn	MuC_Ageddown	MuC
3.62E-08	-0.5158782	0.184	0.41	0.000867	Tmprss4	MuC_Ageddown	MuC
3.28E-09	-0.5194912	0.184	0.463	7.86E-05	Trp63	MuC_Ageddown	MuC
6.77E-09	-0.5250871	0.19	0.446	0.000162	Ppl	MuC_Ageddown	MuC
1.27E-09	-0.5290543	0.178	0.435	3.05E-05	Tuba1a	MuC_Ageddown	MuC
1.87E-10	-0.5333645	0.675	0.895	4.47E-06	Cebpb	MuC_Ageddown	MuC
2.50E-09	-0.5335006	0.399	0.64	5.99E-05	Cacybp	MuC_Ageddown	MuC
5.53E-12	-0.5368713	0.141	0.44	1.33E-07	Pls3	MuC_Ageddown	MuC
3.80E-22	-0.5419791	0.098	0.557	9.11E-18	Dbp	MuC_Ageddown	MuC
1.00E-08	-0.5477841	0.147	0.385	0.00024	Serpinb1a	MuC_Ageddown	MuC
2.97E-10	-0.5485654	0.926	0.967	7.13E-06	Rps25	MuC_Ageddown	MuC
1.00E-08	-0.5533024	0.282	0.579	0.000241	Pkp1	MuC_Ageddown	MuC
1.44E-07	-0.5539769	0.417	0.645	0.003453	Selenbp1	MuC_Ageddown	MuC
4.01E-07	-0.5543217	0.245	0.443	0.009612	Ccnd2	MuC_Ageddown	MuC
5.57E-12	-0.5545242	0.086	0.382	1.34E-07	Klk8	MuC_Ageddown	MuC
1.57E-06	-0.5571971	0.638	0.798	0.037662	Hras	MuC_Ageddown	MuC
2.70E-09	-0.5583349	0.166	0.44	6.48E-05	Ces1h	MuC_Ageddown	MuC
7.47E-10	-0.5620968	0.184	0.465	1.79E-05	Tmprss11a	MuC_Ageddown	MuC
7.67E-09	-0.5638429	0.423	0.681	0.000184	Hmgb2	MuC_Ageddown	MuC
1.60E-07	-0.5645286	0.804	0.922	0.003827	Serbp1	MuC_Ageddown	MuC
1.00E-08	-0.5671048	0.27	0.524	0.00024	Sdc1	MuC_Ageddown	MuC
5.54E-13	-0.5677624	0.184	0.529	1.33E-08	Phlda3	MuC_Ageddown	MuC
3.60E-08	-0.567916	0.969	0.981	0.000863	Rplp2	MuC_Ageddown	MuC
8.46E-11	-0.5679939	0.798	0.939	2.03E-06	Eif4a1	MuC_Ageddown	MuC
2.38E-09	-0.5819412	0.172	0.449	5.70E-05	Ndufa4l2	MuC_Ageddown	MuC
4.85E-14	-0.5823297	0.055	0.371	1.16E-09	Elovl4	MuC_Ageddown	MuC
6.39E-10	-0.5864493	0.092	0.349	1.53E-05	Klk13	MuC_Ageddown	MuC
1.24E-10	-0.5885897	0.411	0.706	2.97E-06	Gstm1	MuC_Ageddown	MuC
9.96E-09	-0.6008119	0.307	0.576	0.000239	Dsc2	MuC_Ageddown	MuC
1.44E-08	-0.6009766	0.018	0.211	0.000345	Nccrp1	MuC_Ageddown	MuC
5.10E-09	-0.6016178	0.276	0.554	0.000122	Trim29	MuC_Ageddown	MuC
1.67E-12	-0.6033677	0.172	0.49	4.01E-08	Krt80	MuC_Ageddown	MuC
7.32E-10	-0.6089437	0.552	0.795	1.75E-05	Mgst3	MuC_Ageddown	MuC
2.06E-10	-0.6124935	0.221	0.512	4.95E-06	Wnt4	MuC_Ageddown	MuC
1.90E-12	-0.6200724	0.166	0.488	4.55E-08	Hmgn3	MuC_Ageddown	MuC
2.25E-23	-0.6237881	0.337	0.895	5.40E-19	Tmem59	MuC_Ageddown	MuC
1.32E-11	-0.6364936	0.27	0.562	3.18E-07	Ctnnbip1	MuC_Ageddown	MuC
1.33E-09	-0.6403766	0.27	0.548	3.19E-05	Cryab	MuC_Ageddown	MuC
3.55E-14	-0.6417447	0.11	0.435	8.50E-10	Defb4	MuC_Ageddown	MuC
5.50E-08	-0.6455371	0.252	0.463	0.001319	Lgalsl	MuC_Ageddown	MuC
4.48E-10	-0.6475375	0.209	0.488	1.07E-05	Dst	MuC_Ageddown	MuC
2.83E-10	-0.6547211	0.184	0.468	6.80E-06	Pinlyp	MuC_Ageddown	MuC
1.18E-08	-0.658109	0.969	0.992	0.000284	Rpl21	MuC_Ageddown	MuC
1.67E-11	-0.673953	0.117	0.404	4.01E-07	Ptn	MuC_Ageddown	MuC
2.82E-09	-0.6775486	0.564	0.773	6.76E-05	Gltp	MuC_Ageddown	MuC
2.95E-13	-0.69408	0.785	0.928	7.08E-09	Rpl23a	MuC_Ageddown	MuC
1.22E-06	-0.6944198	0.411	0.598	0.029272	Pdzk1ip1	MuC_Ageddown	MuC
7.91E-17	-0.6964265	0.16	0.535	1.90E-12	Hsph1	MuC_Ageddown	MuC

5.47E-10	-0.7145536	0.16	0.454	1.31E-05	Crabp2	MuC_Ageddown	MuC
1.74E-06	-0.7158875	0.061	0.235	0.041641	S100g	MuC_Ageddown	MuC
6.02E-07	-0.7205864	0.558	0.726	0.014434	Cyp2f2	MuC_Ageddown	MuC
9.16E-15	-0.7215905	0.141	0.501	2.20E-10	Cst6	MuC_Ageddown	MuC
8.70E-08	-0.7280119	0.485	0.825	0.002085	Prb1	MuC_Ageddown	MuC
1.33E-09	-0.7531892	0.294	0.537	3.18E-05	Serpinb5	MuC_Ageddown	MuC
8.90E-13	-0.7576023	0.196	0.518	2.13E-08	Col17a1	MuC_Ageddown	MuC
1.74E-14	-0.7702922	0.086	0.41	4.17E-10	Csta1	MuC_Ageddown	MuC
6.02E-10	-0.7745519	0.325	0.579	1.44E-05	Them5	MuC_Ageddown	MuC
3.47E-17	-0.7905018	0.644	0.873	8.33E-13	Dnaja1	MuC_Ageddown	MuC
1.12E-10	-0.7931604	0.485	0.737	2.69E-06	Jup	MuC_Ageddown	MuC
2.38E-08	-0.8060629	0.994	0.994	0.000571	Rps18	MuC_Ageddown	MuC
2.35E-10	-0.8183003	0.141	0.413	5.64E-06	Serpinb12	MuC_Ageddown	MuC
2.12E-17	-0.8193643	0.172	0.59	5.08E-13	Capns2	MuC_Ageddown	MuC
3.94E-10	-0.827845	0.92	0.994	9.46E-06	Sbpl	MuC_Ageddown	MuC
2.07E-12	-0.8412682	0.19	0.507	4.96E-08	Lypd3	MuC_Ageddown	MuC
1.71E-08	-0.8413652	0.436	0.654	0.000409	Fam25c	MuC_Ageddown	MuC
4.59E-13	-0.8422989	0.202	0.521	1.10E-08	Tacstd2	MuC_Ageddown	MuC
1.46E-08	-0.8640034	0.135	0.374	0.00035	Crct1	MuC_Ageddown	MuC
1.36E-17	-0.8720676	0.135	0.521	3.26E-13	Id1	MuC_Ageddown	MuC
1.09E-10	-0.900102	0.166	0.454	2.61E-06	Krt16	MuC_Ageddown	MuC
7.55E-11	-0.9041748	0.951	0.986	1.81E-06	H3f3a	MuC_Ageddown	MuC
9.25E-17	-0.9068538	0.135	0.526	2.22E-12	Rbp1	MuC_Ageddown	MuC
3.93E-15	-0.9095567	0.135	0.476	9.42E-11	Sptssb	MuC_Ageddown	MuC
5.31E-11	-0.9346148	0.117	0.41	1.27E-06	Klk14	MuC_Ageddown	MuC
8.73E-21	-0.9361036	0.89	0.972	2.09E-16	Hspa8	MuC_Ageddown	MuC
3.28E-17	-0.9436559	0.08	0.454	7.87E-13	Tmprss11d	MuC_Ageddown	MuC
4.44E-07	-0.9677795	0.638	0.839	0.010645	Mt4	MuC_Ageddown	MuC
2.05E-13	-0.9731912	0.202	0.551	4.91E-09	Ecm1	MuC_Ageddown	MuC
1.84E-19	-0.9773436	0.969	0.983	4.42E-15	Rps12	MuC_Ageddown	MuC
1.86E-08	-0.9932424	0.503	0.679	0.000445	Aldh3a1	MuC_Ageddown	MuC
2.34E-09	-1.0020936	0.356	0.601	5.62E-05	Calml3	MuC_Ageddown	MuC
5.35E-21	-1.029309	1	1	1.28E-16	Rps8	MuC_Ageddown	MuC
1.07E-11	-1.0388484	0.307	0.576	2.57E-07	Calm4	MuC_Ageddown	MuC
7.84E-13	-1.0576698	0.11	0.421	1.88E-08	Plac8	MuC_Ageddown	MuC
1.54E-19	-1.0733366	0.73	0.92	3.69E-15	Hsp90aa1	MuC_Ageddown	MuC
1.60E-11	-1.0888697	0.54	0.776	3.84E-07	Pycard	MuC_Ageddown	MuC
4.88E-18	-1.0970588	0.117	0.504	1.17E-13	Spink5	MuC_Ageddown	MuC
1.40E-15	-1.1045385	0.196	0.554	3.36E-11	Emp1	MuC_Ageddown	MuC
1.73E-20	-1.1074989	0.129	0.54	4.15E-16	Anxa8	MuC_Ageddown	MuC
9.97E-11	-1.1255737	0.417	0.645	2.39E-06	Hopx	MuC_Ageddown	MuC
2.08E-20	-1.1318628	0.755	0.947	4.99E-16	Ggh	MuC_Ageddown	MuC
5.32E-21	-1.1337628	0.945	0.994	1.27E-16	Hsp90ab1	MuC_Ageddown	MuC
2.97E-16	-1.1418418	0.589	0.95	7.12E-12	Amy1	MuC_Ageddown	MuC
1.17E-06	-1.1581463	0.613	0.695	0.02808	S100a10	MuC_Ageddown	MuC
1.66E-10	-1.1729814	0.479	0.723	3.99E-06	Dmkn	MuC_Ageddown	MuC
8.65E-15	-1.1730653	0.35	0.751	2.07E-10	Bpifa2	MuC_Ageddown	MuC
4.26E-10	-1.1999158	0.08	0.332	1.02E-05	2310046K23Rik	MuC_Ageddown	MuC
5.39E-11	-1.2053537	0.258	0.524	1.29E-06	Tgm3	MuC_Ageddown	MuC
1.30E-11	-1.2185872	0.245	0.532	3.11E-07	Gm94	MuC_Ageddown	MuC
6.53E-08	-1.2343721	0.748	0.859	0.001566	Perp	MuC_Ageddown	MuC
1.40E-19	-1.2351974	0.11	0.512	3.36E-15	Aqp3	MuC_Ageddown	MuC
5.55E-18	-1.2367054	0.123	0.51	1.33E-13	Rbp2	MuC_Ageddown	MuC
2.03E-22	-1.238433	0.135	0.571	4.88E-18	Id3	MuC_Ageddown	MuC
4.28E-20	-1.2455582	0.141	0.562	1.03E-15	Mal	MuC_Ageddown	MuC
3.24E-10	-1.2578339	0.479	0.662	7.78E-06	S100a14	MuC_Ageddown	MuC
2.76E-13	-1.268025	0.319	0.626	6.63E-09	Sbsn	MuC_Ageddown	MuC
5.68E-12	-1.2695824	0.356	0.62	1.36E-07	Lgals3	MuC_Ageddown	MuC
1.56E-30	-1.2834382	0.117	0.662	3.74E-26	Hspa1a	MuC_Ageddown	MuC

1.17E-13	-1.2944274	0.135	0.449	2.80E-09	Klk10	MuC_Ageddown	MuC
1.21E-13	-1.3096266	0.27	0.571	2.89E-09	Adh7	MuC_Ageddown	MuC
1.96E-18	-1.3662794	0.08	0.465	4.70E-14	Krt75	MuC_Ageddown	MuC
3.62E-11	-1.3966759	0.497	0.72	8.67E-07	Sfn	MuC_Ageddown	MuC
5.03E-14	-1.4160708	0.374	0.687	1.21E-09	Krt5	MuC_Ageddown	MuC
6.30E-16	-1.4370203	0.288	0.668	1.51E-11	Krt15	MuC_Ageddown	MuC
3.72E-18	-1.4837473	0.067	0.443	8.91E-14	Sprr2a3	MuC_Ageddown	MuC
6.85E-09	-1.5551561	0.583	0.776	0.000164	Krtdap	MuC_Ageddown	MuC
1.23E-11	-1.751318	0.693	0.87	2.94E-07	Lgals7	MuC_Ageddown	MuC
3.52E-14	-1.8389381	0.552	0.812	8.45E-10	Ly6d	MuC_Ageddown	MuC
2.40E-13	-1.8582759	0.521	0.762	5.76E-09	Krt6a	MuC_Ageddown	MuC
2.39E-36	-1.8749976	0.178	0.753	5.72E-32	Hspa1b	MuC_Ageddown	MuC
3.68E-21	-1.9359664	0.147	0.562	8.83E-17	Ly6g6c	MuC_Ageddown	MuC
8.59E-15	-1.9796858	0.037	0.357	2.06E-10	Psca	MuC_Ageddown	MuC
3.45E-17	-2.0029078	0.202	0.576	8.28E-13	Krt6b	MuC_Ageddown	MuC
5.90E-23	-2.1574736	0.117	0.59	1.41E-18	2300002M23Rik	MuC_Ageddown	MuC
5.78E-21	-2.2298697	0.442	0.809	1.38E-16	Hspb1	MuC_Ageddown	MuC
2.38E-18	-2.2780962	0.221	0.62	5.70E-14	Cnfn	MuC_Ageddown	MuC
7.53E-18	-2.4060862	0.411	0.792	1.81E-13	Krt14	MuC_Ageddown	MuC
4.09E-23	-2.6099165	0.411	0.845	9.82E-19	Krt17	MuC_Ageddown	MuC
7.56E-19	-2.8713944	0.307	0.67	1.81E-14	Sprr1a	MuC_Ageddown	MuC
2.08E-28	-2.8962725	0.325	0.803	4.98E-24	Gm8882	MuC_Ageddown	MuC
8.68E-08	1.49247899	0.308	0	0.002081	Xist	IC_Agedup	IC
9.60E-10	1.45110753	0.383	0	2.30E-05	Gm10260	IC_Agedup	IC
4.42E-07	1.41548696	0.608	0.273	0.010594	Ly6a	IC_Agedup	IC
1.24E-12	1.11147571	0.958	0.857	2.97E-08	mt-Atp8	IC_Agedup	IC
1.00E-07	1.03051298	0.983	0.896	0.002406	Crip1	IC_Agedup	IC
5.67E-13	1.02625215	1	0.987	1.36E-08	Gm42418	IC_Agedup	IC
6.06E-08	1.0242334	0.867	0.714	0.001454	Lmna	IC_Agedup	IC
8.29E-07	1.01310162	0.583	0.26	0.01987	Tppp3	IC_Agedup	IC
1.62E-06	0.99410774	0.95	0.818	0.038904	S100a10	IC_Agedup	IC
1.48E-07	0.96705923	0.842	0.597	0.003552	Mif	IC_Agedup	IC
3.44E-12	0.92659799	0.967	0.974	8.25E-08	Gm10076	IC_Agedup	IC
1.17E-09	0.8133011	0.917	0.805	2.79E-05	Cox5a	IC_Agedup	IC
1.12E-13	0.80608008	1	1	2.69E-09	mt-Nd4l	IC_Agedup	IC
8.39E-12	0.7799452	1	1	2.01E-07	Ppia	IC_Agedup	IC
5.38E-07	0.74960986	0.775	0.494	0.012892	Nme1	IC_Agedup	IC
3.06E-07	0.74346158	0.917	0.831	0.007334	Txn1	IC_Agedup	IC
9.20E-07	0.70168324	0.833	0.766	0.022065	Ldha	IC_Agedup	IC
9.77E-07	0.68814978	0.717	0.429	0.023432	Ndufab1	IC_Agedup	IC
1.62E-07	0.65489341	0.942	0.805	0.003883	Cox5b	IC_Agedup	IC
1.04E-06	0.65168033	0.758	0.519	0.02487	Ndufs5	IC_Agedup	IC
1.91E-06	0.65112853	0.9	0.779	0.045916	Uqcrb	IC_Agedup	IC
1.55E-06	0.58881058	0.908	0.844	0.037195	Elob	IC_Agedup	IC
1.23E-06	0.43277791	0.992	0.987	0.029507	Oaz1	IC_Agedup	IC
1.14E-11	0.4293605	1	1	2.72E-07	mt-Co1	IC_Agedup	IC
2.35E-07	-0.261567	1	1	0.005628	mt-Atp6	IC_Ageddown	IC
5.45E-08	-0.3136215	1	1	0.001308	mt-Cytb	IC_Ageddown	IC
1.94E-06	-0.3583277	1	1	0.046482	mt-Nd4	IC_Ageddown	IC
1.66E-07	-0.3992328	1	1	0.003988	Rpl23	IC_Ageddown	IC
1.12E-06	-0.4060309	0.992	1	0.026778	Rpl26	IC_Ageddown	IC
7.95E-07	-0.4255952	0.992	1	0.019077	Rplp2	IC_Ageddown	IC
4.52E-08	-0.5088396	0.958	1	0.001084	Rack1	IC_Ageddown	IC
1.03E-08	-0.5230708	1	1	0.000248	Rps14	IC_Ageddown	IC
7.51E-09	-0.5438855	1	1	0.00018	Rplp1	IC_Ageddown	IC
5.87E-10	-0.5507192	1	1	1.41E-05	Tpt1	IC_Ageddown	IC
9.86E-07	-0.5513526	0.942	0.987	0.02365	Rpl12	IC_Ageddown	IC
1.20E-11	-0.5827692	1	1	2.87E-07	mt-Nd2	IC_Ageddown	IC
1.35E-07	-0.591525	0.658	0.922	0.003236	Pcbp2	IC_Ageddown	IC

1.16E-19	-0.6036839	1	1	2.79E-15	mt-Co3	IC_Ageddown	IC
2.80E-09	-0.6106205	0.992	1	6.71E-05	Ubb	IC_Ageddown	IC
3.57E-07	-0.6116994	0.317	0.662	0.008569	Mat2a	IC_Ageddown	IC
9.36E-08	-0.7024868	0.108	0.429	0.002245	Tec	IC_Ageddown	IC
1.16E-06	-0.7107204	0.967	1	0.027837	Cd52	IC_Ageddown	IC
3.66E-08	-0.7116944	0.85	0.948	0.000879	Hspe1	IC_Ageddown	IC
5.13E-17	-0.7287392	0.983	1	1.23E-12	Rps8	IC_Ageddown	IC
5.77E-08	-0.7337626	0.95	1	0.001384	Sh3bgrl3	IC_Ageddown	IC
4.39E-15	-0.7375561	1	1	1.05E-10	Rps21	IC_Ageddown	IC
8.16E-07	-0.7712854	0.967	0.974	0.019577	Junb	IC_Ageddown	IC
1.85E-08	-0.7827324	0.267	0.688	0.000444	Fkbp5	IC_Ageddown	IC
3.89E-07	-0.7889179	0.075	0.364	0.009327	Sertad1	IC_Ageddown	IC
1.09E-06	-0.789016	0.95	0.974	0.026072	Ubc	IC_Ageddown	IC
1.41E-07	-0.7902581	0.358	0.714	0.003383	Tmem59	IC_Ageddown	IC
1.26E-12	-0.7939956	0.942	1	3.03E-08	Rps25	IC_Ageddown	IC
1.45E-08	-0.8373949	0.583	0.87	0.000347	Ccnl1	IC_Ageddown	IC
5.31E-07	-0.8835007	0.317	0.662	0.012736	Icam1	IC_Ageddown	IC
1.13E-08	-0.8857576	0.875	0.987	0.000271	Srgn	IC_Ageddown	IC
2.38E-14	-0.900059	0.983	1	5.71E-10	Rps12	IC_Ageddown	IC
2.44E-16	-0.930999	0.958	1	5.86E-12	Hsp90ab1	IC_Ageddown	IC
8.54E-09	-0.9590006	0.833	0.922	0.000205	Hspa5	IC_Ageddown	IC
2.18E-09	-0.9593953	0.125	0.494	5.23E-05	Hsph1	IC_Ageddown	IC
1.77E-08	-0.9642839	0.783	0.948	0.000425	Nfkbia	IC_Ageddown	IC
3.37E-10	-0.9650437	0.117	0.532	8.09E-06	Mndal	IC_Ageddown	IC
5.53E-11	-0.9815436	0.783	0.987	1.33E-06	Rpl23a	IC_Ageddown	IC
5.51E-18	-1.1277375	0.967	1	1.32E-13	Hspa8	IC_Ageddown	IC
5.80E-09	-1.1627983	0.175	0.545	0.000139	Hspa1b	IC_Ageddown	IC
5.72E-19	-1.2621494	0.683	0.974	1.37E-14	Hsp90aa1	IC_Ageddown	IC
8.96E-17	-1.3165537	0.6	0.961	2.15E-12	Dnaja1	IC_Ageddown	IC
1.89E-15	-1.3353811	0.325	0.831	4.54E-11	AY036118	IC_Ageddown	IC
1.88E-11	-1.3414734	0.167	0.61	4.50E-07	Hpgd	IC_Ageddown	IC
6.14E-09	-1.551754	0.408	0.727	0.000147	Gadd45b	IC_Ageddown	IC
5.35E-19	2.88095088	0.906	0.505	1.28E-14	Ly6a	MC_Agedup	MC
3.05E-18	2.48327349	0.718	0.133	7.31E-14	Ly6c1	MC_Agedup	MC
9.80E-14	2.39119789	0.671	0.171	2.35E-09	Prg4	MC_Agedup	MC
1.17E-20	2.37657532	0.953	0.41	2.81E-16	Ifi27l2a	MC_Agedup	MC
3.35E-12	2.23957837	0.635	0.2	8.03E-08	Uap1	MC_Agedup	MC
2.83E-11	2.15914815	0.529	0.105	6.80E-07	Cd55	MC_Agedup	MC
1.72E-07	2.0467835	0.235	0	0.004117	Tff2	MC_Agedup	MC
4.41E-10	2.01588666	0.612	0.19	1.06E-05	C3	MC_Agedup	MC
4.56E-16	2.00829967	0.565	0.038	1.09E-11	Gfpt2	MC_Agedup	MC
1.93E-16	1.97452389	0.918	0.571	4.64E-12	H2-D1	MC_Agedup	MC
6.56E-11	1.93267776	0.682	0.267	1.57E-06	Ebf1	MC_Agedup	MC
3.19E-21	1.87285546	1	0.933	7.66E-17	Gm42418	MC_Agedup	MC
1.20E-18	1.83264378	0.988	0.81	2.88E-14	Crip1	MC_Agedup	MC
1.62E-10	1.75564432	0.635	0.2	3.88E-06	Fbn1	MC_Agedup	MC
6.60E-09	1.71909866	0.729	0.4	0.000158	Emp3	MC_Agedup	MC
2.92E-11	1.66818462	0.388	0.01	7.00E-07	Xist	MC_Agedup	MC
3.57E-10	1.64053972	0.788	0.467	8.56E-06	Lrp1	MC_Agedup	MC
1.05E-09	1.63353339	0.6	0.219	2.53E-05	Ackr3	MC_Agedup	MC
1.05E-08	1.61567285	0.576	0.219	0.000251	Ugdh	MC_Agedup	MC
1.58E-08	1.59514712	0.271	0	0.000379	C4b	MC_Agedup	MC
4.67E-12	1.59382271	0.8	0.476	1.12E-07	Tppp3	MC_Agedup	MC
3.68E-09	1.58175834	0.553	0.162	8.82E-05	Mustn1	MC_Agedup	MC
7.66E-15	1.53247125	0.871	0.438	1.84E-10	H2-K1	MC_Agedup	MC
7.14E-11	1.4961316	0.576	0.171	1.71E-06	Tmem100	MC_Agedup	MC
9.98E-10	1.48638096	0.682	0.267	2.39E-05	Clec3b	MC_Agedup	MC
1.54E-09	1.48528754	0.494	0.114	3.70E-05	Htra3	MC_Agedup	MC
2.27E-13	1.48431675	0.424	0	5.44E-09	Gm10260	MC_Agedup	MC

1.37E-08	1.46495878	0.694	0.333	0.000328	Tnxb	MC_Agedup	MC
7.69E-09	1.45226899	0.518	0.152	0.000185	Cd248	MC_Agedup	MC
7.96E-11	1.43991786	0.506	0.105	1.91E-06	Heg1	MC_Agedup	MC
5.41E-07	1.3828209	0.694	0.429	0.01298	Fstl1	MC_Agedup	MC
9.58E-12	1.38150778	0.4	0.01	2.30E-07	Cadm3	MC_Agedup	MC
2.13E-07	1.36660781	0.706	0.419	0.005099	Mfap5	MC_Agedup	MC
1.28E-07	1.32842737	0.671	0.305	0.003075	Cfh	MC_Agedup	MC
1.67E-14	1.32426123	0.988	0.819	4.01E-10	Ifitm3	MC_Agedup	MC
2.33E-09	1.31425291	0.318	0.01	5.59E-05	Has2	MC_Agedup	MC
5.78E-11	1.30711105	0.882	0.581	1.39E-06	S100a13	MC_Agedup	MC
2.41E-09	1.3023468	0.565	0.181	5.78E-05	Fhl1	MC_Agedup	MC
1.07E-15	1.29542079	0.929	0.676	2.57E-11	mt-Atp8	MC_Agedup	MC
1.28E-08	1.27099281	0.506	0.143	0.000307	Medag	MC_Agedup	MC
2.36E-08	1.26350397	0.659	0.314	0.000565	Fbln2	MC_Agedup	MC
2.32E-11	1.24752755	0.824	0.505	5.56E-07	B2m	MC_Agedup	MC
2.83E-09	1.24115636	0.6	0.219	6.79E-05	Ugp2	MC_Agedup	MC
6.50E-09	1.24055066	0.306	0.01	0.000156	Dio2	MC_Agedup	MC
1.00E-06	1.23545606	0.671	0.448	0.024022	Axl	MC_Agedup	MC
7.44E-10	1.23396485	0.518	0.095	1.79E-05	Pcolce2	MC_Agedup	MC
3.60E-09	1.22232857	0.518	0.124	8.63E-05	Scara5	MC_Agedup	MC
2.67E-07	1.20284606	0.6	0.276	0.006405	Rhoc	MC_Agedup	MC
4.36E-07	1.19939899	0.741	0.533	0.010452	Rnase4	MC_Agedup	MC
1.67E-07	1.18216148	0.482	0.162	0.004001	Bst2	MC_Agedup	MC
4.08E-09	1.16969322	0.482	0.095	9.78E-05	Loxl1	MC_Agedup	MC
1.08E-07	1.15002637	0.824	0.571	0.002594	Serpinb6a	MC_Agedup	MC
3.60E-08	1.09825696	0.388	0.067	0.000863	Gngt2	MC_Agedup	MC
1.21E-06	1.09039906	0.541	0.229	0.02895	Tgfbr2	MC_Agedup	MC
6.20E-19	1.0848733	1	0.933	1.49E-14	mt-Nd4l	MC_Agedup	MC
1.88E-07	1.08062367	0.671	0.39	0.004519	Neat1	MC_Agedup	MC
2.13E-07	1.07038781	0.647	0.39	0.005111	Psme2	MC_Agedup	MC
8.09E-08	1.06608043	0.4	0.076	0.001939	Ckb	MC_Agedup	MC
3.25E-07	1.06341675	0.518	0.19	0.007789	Abca8b	MC_Agedup	MC
8.15E-07	1.04699516	0.671	0.314	0.019535	Pi16	MC_Agedup	MC
3.07E-08	1.02431709	0.565	0.171	0.000735	Ccl11	MC_Agedup	MC
1.39E-09	1.02170754	0.788	0.438	3.33E-05	Ly6e	MC_Agedup	MC
1.94E-10	1.02090587	0.941	0.695	4.65E-06	Tmsb10	MC_Agedup	MC
6.76E-08	1.01422327	0.553	0.219	0.001621	Lsp1	MC_Agedup	MC
2.00E-07	1.00384247	0.706	0.429	0.004787	Jpt1	MC_Agedup	MC
1.90E-08	0.99732153	0.482	0.124	0.000455	Ddr2	MC_Agedup	MC
4.20E-08	0.9953012	0.353	0.048	0.001007	Dbn1	MC_Agedup	MC
5.96E-07	0.9661102	0.412	0.105	0.01429	Lrrn4cl	MC_Agedup	MC
5.83E-08	0.95537143	0.494	0.143	0.001399	Nav1	MC_Agedup	MC
4.07E-07	0.94132487	0.471	0.143	0.009754	Hmcn2	MC_Agedup	MC
4.79E-08	0.92804734	0.906	0.743	0.00115	Tspo	MC_Agedup	MC
2.56E-07	0.92511111	0.271	0.019	0.006134	Adgrd1	MC_Agedup	MC
6.38E-08	0.89630514	1	0.886	0.001529	S100a6	MC_Agedup	MC
6.67E-07	0.8942377	0.6	0.305	0.015988	Rock2	MC_Agedup	MC
1.18E-08	0.87917228	0.424	0.067	0.000282	Psmb8	MC_Agedup	MC
7.45E-08	0.85137688	0.941	0.781	0.001786	Anxa2	MC_Agedup	MC
1.71E-06	0.84285059	0.412	0.124	0.041017	Emilin2	MC_Agedup	MC
4.83E-08	0.8358653	0.388	0.067	0.001158	Ndrg1	MC_Agedup	MC
1.87E-06	0.83379785	0.341	0.076	0.044825	Tnfaip2	MC_Agedup	MC
8.13E-07	0.80805832	0.212	0	0.019495	Efhd1	MC_Agedup	MC
1.25E-06	0.75654142	0.459	0.152	0.030041	Metrnl	MC_Agedup	MC
1.60E-07	0.7520196	0.259	0.01	0.003843	Ifi205	MC_Agedup	MC
1.68E-06	0.7421302	0.706	0.448	0.040267	Cyba	MC_Agedup	MC
5.07E-07	0.73818806	0.247	0.01	0.012162	Stmn4	MC_Agedup	MC
1.72E-06	0.71181728	0.459	0.152	0.041176	Il33	MC_Agedup	MC
2.02E-21	0.70924793	1	1	4.85E-17	mt-Co1	MC_Agedup	MC

1.43E-07	0.7051303	0.824	0.543	0.003432	Rsrp1	MC_Agedup	MC
3.46E-08	0.69718835	0.976	0.933	0.00083	Ftl1	MC_Agedup	MC
1.76E-06	0.66917197	0.306	0.048	0.042165	Fam111a	MC_Agedup	MC
5.75E-07	0.54128598	0.988	0.886	0.01379	mt-Nd5	MC_Agedup	MC
9.66E-08	0.33730318	1	0.99	0.002318	mt-Co2	MC_Agedup	MC
2.64E-07	-0.3418769	1	1	0.00634	mt-Co3	MC_Ageddown	MC
1.20E-07	-0.5257259	1	1	0.002883	Rpl23	MC_Ageddown	MC
9.89E-07	-0.548951	0.988	0.99	0.023715	Rps16	MC_Ageddown	MC
8.15E-09	-0.6313244	1	0.971	0.000195	Ptma	MC_Ageddown	MC
1.90E-08	-0.6477661	1	0.962	0.000456	Rps21	MC_Ageddown	MC
9.61E-10	-0.7040635	0.988	0.962	2.31E-05	Rpl21	MC_Ageddown	MC
5.17E-10	-0.7333743	1	0.981	1.24E-05	Rpl17	MC_Ageddown	MC
3.20E-08	-0.7360683	0.941	0.933	0.000768	H3f3a	MC_Ageddown	MC
5.39E-10	-0.7504675	1	0.952	1.29E-05	Rplp1	MC_Ageddown	MC
2.21E-08	-0.7546617	0.953	0.981	0.000531	Rpl12	MC_Ageddown	MC
5.72E-17	-0.791385	0.988	1	1.37E-12	Tpt1	MC_Ageddown	MC
9.46E-10	-0.7974168	1	0.981	2.27E-05	Rps14	MC_Ageddown	MC
8.75E-13	-0.9170812	0.918	0.943	2.10E-08	Hspa8	MC_Ageddown	MC
1.45E-10	-0.9227165	0.953	0.981	3.48E-06	Rps12	MC_Ageddown	MC
1.50E-06	-0.9377694	0.518	0.743	0.035887	Dnaja1	MC_Ageddown	MC
6.14E-12	-0.9688455	0.965	0.933	1.47E-07	Hsp90ab1	MC_Ageddown	MC
3.49E-19	-1.101963	1	0.99	8.38E-15	Rps8	MC_Ageddown	MC
1.30E-11	-1.2655789	0.212	0.695	3.11E-07	Tmem59	MC_Ageddown	MC
3.49E-07	-1.2710986	0.494	0.714	0.008373	Fxyd3	MC_Ageddown	MC
4.58E-07	-1.2767877	0.012	0.286	0.010972	C1qtnf3	MC_Ageddown	MC
1.72E-07	-1.4168309	0.118	0.448	0.004122	Dkk3	MC_Ageddown	MC
3.68E-11	-1.4648577	0.176	0.657	8.81E-07	Dbp	MC_Ageddown	MC
4.95E-07	-1.5591713	0.188	0.524	0.011867	Ier3	MC_Ageddown	MC
1.69E-11	-1.7201042	0.294	0.695	4.05E-07	Hspa1b	MC_Ageddown	MC
1.89E-11	-1.8671605	0.176	0.619	4.52E-07	Id1	MC_Ageddown	MC
1.64E-11	-1.9047572	0.435	0.8	3.93E-07	Id3	MC_Ageddown	MC
1.59E-06	-1.9419597	0.294	0.6	0.038224	Egr1	MC_Ageddown	MC
1.47E-08	-3.1721975	0.494	0.829	0.000353	Sbpl	MC_Ageddown	MC
4.31E-33	2.63475298	0.58	0.182	1.03E-28	Ifi27l2a	MTC_Agedup	MTC
3.74E-14	2.59711839	0.31	0.099	8.96E-10	Lyz2	MTC_Agedup	MTC
1.45E-22	1.71319863	0.204	0.008	3.47E-18	Tff2	MTC_Agedup	MTC
1.79E-06	1.71178944	0.263	0.134	0.043036	S100g	MTC_Agedup	MTC
4.61E-10	1.58018952	0.212	0.067	1.10E-05	Hbb-bs	MTC_Agedup	MTC
3.37E-28	1.50048627	0.906	0.751	8.08E-24	Gm42418	MTC_Agedup	MTC
1.49E-06	1.49265661	0.478	0.358	0.035689	Krt4	MTC_Agedup	MTC
2.16E-23	1.37309349	0.235	0.018	5.18E-19	Muc5b	MTC_Agedup	MTC
1.22E-17	1.26388491	0.157	0.006	2.93E-13	Xist	MTC_Agedup	MTC
2.81E-23	1.25595097	0.843	0.644	6.75E-19	H2-D1	MTC_Agedup	MTC
5.72E-64	1.22703181	0.996	0.941	1.37E-59	mt-Nd4l	MTC_Agedup	MTC
3.38E-25	1.22623518	0.765	0.498	8.10E-21	Dusp1	MTC_Agedup	MTC
6.77E-22	1.12324601	0.788	0.559	1.62E-17	H2-K1	MTC_Agedup	MTC
1.09E-20	1.11478956	0.627	0.34	2.62E-16	B2m	MTC_Agedup	MTC
8.54E-41	1.11108152	0.318	0	2.05E-36	Gm10260	MTC_Agedup	MTC
2.06E-28	1.10842739	0.824	0.642	4.94E-24	mt-Atp8	MTC_Agedup	MTC
1.90E-25	1.02590794	0.937	0.82	4.57E-21	Crip1	MTC_Agedup	MTC
1.05E-47	1.00729477	1	0.937	2.51E-43	Gm10076	MTC_Agedup	MTC
7.52E-16	0.93310295	0.796	0.648	1.80E-11	Zfp36	MTC_Agedup	MTC
1.70E-06	0.90584625	0.624	0.528	0.04085	Krt13	MTC_Agedup	MTC
6.17E-09	0.9017116	0.212	0.073	0.000148	Ly6a	MTC_Agedup	MTC
2.07E-07	0.85426347	0.482	0.35	0.004968	Socs3	MTC_Agedup	MTC
5.69E-12	0.82561336	0.576	0.387	1.36E-07	Txnip	MTC_Agedup	MTC
4.76E-16	0.74078751	0.639	0.374	1.14E-11	Ifi27	MTC_Agedup	MTC
4.72E-15	0.71385807	0.851	0.735	1.13E-10	Atp1a1	MTC_Agedup	MTC
9.79E-09	0.69529668	0.349	0.186	0.000235	Arc	MTC_Agedup	MTC

9.03E-08	0.67974234	0.62	0.458	0.002166	Klf2	MTC_Agedup	MTC
1.89E-09	0.66099646	0.761	0.64	4.54E-05	Hist1h2bc	MTC_Agedup	MTC
2.32E-14	0.65598033	0.188	0.03	5.56E-10	Psmb8	MTC_Agedup	MTC
7.41E-07	0.65162999	0.376	0.227	0.017776	Wfdc2	MTC_Agedup	MTC
1.12E-06	0.61655694	0.4	0.253	0.026746	Egr2	MTC_Agedup	MTC
7.91E-12	0.61441849	0.353	0.152	1.90E-07	Tppp3	MTC_Agedup	MTC
9.54E-11	0.60548728	0.91	0.864	2.29E-06	Btg2	MTC_Agedup	MTC
4.73E-14	0.60327645	0.753	0.589	1.14E-09	Mrpl54	MTC_Agedup	MTC
5.48E-11	0.59717398	0.325	0.142	1.31E-06	Arrdc3	MTC_Agedup	MTC
2.15E-12	0.58982133	0.816	0.664	5.15E-08	Tsc22d1	MTC_Agedup	MTC
5.60E-11	0.568111	0.91	0.87	1.34E-06	Ier2	MTC_Agedup	MTC
9.68E-08	0.56352901	0.353	0.196	0.002322	Slc16a11	MTC_Agedup	MTC
1.15E-08	0.55725069	0.549	0.37	0.000276	S100a1	MTC_Agedup	MTC
4.93E-07	0.5514891	0.6	0.488	0.011814	Sdc1	MTC_Agedup	MTC
8.26E-42	0.54496801	1	1	1.98E-37	mt-Co1	MTC_Agedup	MTC
3.48E-09	0.54004011	0.435	0.269	8.35E-05	Grcc10	MTC_Agedup	MTC
4.03E-12	0.53793595	0.133	0.014	9.66E-08	Lgals3bp	MTC_Agedup	MTC
1.43E-09	0.52913375	0.906	0.808	3.42E-05	Mif	MTC_Agedup	MTC
1.18E-07	0.52424218	0.475	0.322	0.002826	Tob1	MTC_Agedup	MTC
1.05E-07	0.52360105	0.384	0.233	0.002523	Snhg20	MTC_Agedup	MTC
2.22E-07	0.521706	0.176	0.061	0.005315	Rtp4	MTC_Agedup	MTC
7.72E-07	0.52068251	0.682	0.591	0.01852	Ly6e	MTC_Agedup	MTC
8.28E-07	0.51328479	0.31	0.168	0.019849	Gm26532	MTC_Agedup	MTC
1.38E-18	0.50161013	0.984	0.879	3.31E-14	Uqcr11	MTC_Agedup	MTC
8.78E-07	0.48374032	0.161	0.055	0.021063	Tnfaip2	MTC_Agedup	MTC
1.53E-06	0.48166247	0.514	0.377	0.03664	S100a13	MTC_Agedup	MTC
9.57E-08	0.47889348	0.318	0.172	0.002296	Scrib	MTC_Agedup	MTC
8.13E-15	0.47270111	0.122	0.002	1.95E-10	Ifi203	MTC_Agedup	MTC
2.07E-07	0.47021319	0.471	0.324	0.004969	Prr15l	MTC_Agedup	MTC
2.85E-09	0.44835367	0.812	0.727	6.84E-05	Ndufb7	MTC_Agedup	MTC
6.73E-10	0.44675816	0.831	0.743	1.61E-05	Romo1	MTC_Agedup	MTC
3.22E-13	0.44103483	0.137	0.012	7.72E-09	Samd9l	MTC_Agedup	MTC
1.99E-07	0.43872959	0.384	0.217	0.004777	Rhoc	MTC_Agedup	MTC
3.62E-10	0.43361095	0.718	0.587	8.67E-06	Cuta	MTC_Agedup	MTC
4.10E-15	0.42802823	0.941	0.885	9.83E-11	Spint2	MTC_Agedup	MTC
1.13E-12	0.41488934	0.933	0.84	2.70E-08	H2afj	MTC_Agedup	MTC
1.76E-07	0.40671427	0.725	0.646	0.004231	Tspo	MTC_Agedup	MTC
4.46E-07	0.40509068	0.69	0.555	0.010703	Cirbp	MTC_Agedup	MTC
9.05E-08	0.39844017	0.827	0.802	0.002171	Cst3	MTC_Agedup	MTC
4.86E-07	0.39792306	0.694	0.587	0.011663	1810058I24Rik	MTC_Agedup	MTC
5.79E-07	0.39232487	0.216	0.089	0.01388	Tppp	MTC_Agedup	MTC
1.29E-06	0.39215697	0.725	0.642	0.030984	Mrpl57	MTC_Agedup	MTC
2.65E-07	0.38565803	0.706	0.623	0.006345	Prelid1	MTC_Agedup	MTC
5.91E-07	0.37707265	0.867	0.792	0.014177	Uba52	MTC_Agedup	MTC
1.63E-10	0.36537682	0.165	0.036	3.90E-06	Gm8797	MTC_Agedup	MTC
1.93E-07	0.36395987	0.118	0.026	0.004624	Nefl	MTC_Agedup	MTC
3.45E-09	0.36356743	0.541	0.362	8.27E-05	Gstp1	MTC_Agedup	MTC
1.56E-08	0.36165409	0.784	0.678	0.000374	Ndufs6	MTC_Agedup	MTC
1.21E-06	0.35932601	0.125	0.036	0.029086	1600014C10Rik	MTC_Agedup	MTC
1.76E-06	0.35808357	0.133	0.042	0.042241	Ppp1r3b	MTC_Agedup	MTC
3.09E-11	0.35287061	0.976	0.943	7.40E-07	Cox6b1	MTC_Agedup	MTC
1.29E-12	0.34844459	0.996	0.958	3.09E-08	Ftl1	MTC_Agedup	MTC
7.21E-11	0.34382355	0.773	0.646	1.73E-06	Ndufa3	MTC_Agedup	MTC
2.64E-07	0.33533518	0.863	0.789	0.006328	Scand1	MTC_Agedup	MTC
1.97E-08	0.33142901	0.831	0.729	0.000472	Atp5k	MTC_Agedup	MTC
1.25E-07	0.32545081	0.808	0.713	0.002989	Swi5	MTC_Agedup	MTC
1.06E-08	0.32180068	0.145	0.034	0.000255	Gm10036	MTC_Agedup	MTC
2.92E-08	0.3144905	0.969	0.911	0.0007	Uqcr10	MTC_Agedup	MTC
9.83E-11	0.31347674	1	0.994	2.36E-06	Rpl11	MTC_Agedup	MTC

2.08E-06	0.3065469	0.125	0.038	0.049838	Acta1	MTC_Agedup	MTC
7.23E-07	0.29788528	0.565	0.466	0.017345	Psme2	MTC_Agedup	MTC
8.43E-08	0.29430206	0.941	0.874	0.002023	Atp5g1	MTC_Agedup	MTC
4.58E-07	0.28974739	0.11	0.024	0.010975	Fam71a	MTC_Agedup	MTC
1.48E-06	0.28154046	0.878	0.723	0.035556	Dsp	MTC_Agedup	MTC
1.64E-06	0.27050533	0.557	0.435	0.03943	1810022K09Rik	MTC_Agedup	MTC
5.99E-08	0.26872841	0.902	0.826	0.001436	Ndufc2	MTC_Agedup	MTC
1.46E-14	0.26501455	1	0.998	3.49E-10	mt-Co2	MTC_Agedup	MTC
5.71E-07	0.26073058	1	0.968	0.013699	Rpl36	MTC_Agedup	MTC
9.01E-07	0.25300033	0.922	0.891	0.021617	Fos	MTC_Agedup	MTC
2.94E-07	0.25183978	0.886	0.812	0.007051	Ndufa2	MTC_Agedup	MTC
1.08E-06	0.24130549	0.851	0.796	0.025877	Ndufa11	MTC_Agedup	MTC
2.57E-07	0.20830155	0.973	0.97	0.006174	Ndufa4	MTC_Agedup	MTC
8.24E-07	-0.2328339	0.996	0.951	0.019769	Rpl15	MTC_Ageddown	MTC
3.29E-08	-0.2456484	0.035	0.182	0.000788	Hspa13	MTC_Ageddown	MTC
1.17E-06	-0.2466163	0.11	0.277	0.028071	Rsl24d1	MTC_Ageddown	MTC
9.35E-07	-0.24908	0.996	0.949	0.022418	Rpl27a	MTC_Ageddown	MTC
1.13E-06	-0.2698148	0.031	0.15	0.027186	Ogfrl1	MTC_Ageddown	MTC
5.48E-07	-0.2814188	0.173	0.366	0.013151	Rnf6	MTC_Ageddown	MTC
5.30E-07	-0.2848022	0.91	0.911	0.012715	Rpl29	MTC_Ageddown	MTC
1.59E-08	-0.2862712	0.992	0.976	0.000381	Rps3a1	MTC_Ageddown	MTC
2.51E-15	-0.2914944	1	1	6.01E-11	mt-Atp6	MTC_Ageddown	MTC
5.37E-18	-0.2930841	1	1	1.29E-13	mt-Co3	MTC_Ageddown	MTC
3.42E-07	-0.3015086	0.949	0.953	0.008213	Naca	MTC_Ageddown	MTC
7.61E-07	-0.301743	0.976	0.955	0.018251	Rps18	MTC_Ageddown	MTC
3.99E-11	-0.3133537	0.992	0.982	9.57E-07	Rpl23	MTC_Ageddown	MTC
2.82E-13	-0.3158113	0.996	0.968	6.77E-09	Eef1a1	MTC_Ageddown	MTC
4.68E-07	-0.3167347	0.141	0.324	0.011216	Tes	MTC_Ageddown	MTC
7.68E-07	-0.3185559	0.169	0.356	0.018423	Tmem165	MTC_Ageddown	MTC
1.34E-09	-0.3220527	0.988	0.968	3.21E-05	Rps14	MTC_Ageddown	MTC
1.80E-08	-0.3222882	0.969	0.939	0.000432	Rplp2	MTC_Ageddown	MTC
1.53E-06	-0.3264516	0.165	0.346	0.036627	Tfg	MTC_Ageddown	MTC
5.53E-10	-0.34041	0.039	0.217	1.33E-05	Banp	MTC_Ageddown	MTC
1.76E-07	-0.3569703	0.165	0.366	0.004221	Desi2	MTC_Ageddown	MTC
8.45E-09	-0.3586043	0.914	0.905	0.000203	Btf3	MTC_Ageddown	MTC
4.68E-12	-0.3649031	0.988	0.98	1.12E-07	Ptma	MTC_Ageddown	MTC
1.39E-07	-0.3666349	0.122	0.294	0.003342	Snhg6	MTC_Ageddown	MTC
9.25E-08	-0.3733986	0.184	0.387	0.002218	Mettl23	MTC_Ageddown	MTC
1.68E-06	-0.3751341	0.4	0.591	0.040322	Nudc	MTC_Ageddown	MTC
2.91E-08	-0.3756477	0.153	0.36	0.000699	Cfl2	MTC_Ageddown	MTC
2.34E-10	-0.3861662	0.996	0.986	5.60E-06	Actg1	MTC_Ageddown	MTC
1.31E-06	-0.393383	0.247	0.439	0.031336	Akirin1	MTC_Ageddown	MTC
7.13E-07	-0.3941572	0.388	0.591	0.017099	Rbm8a	MTC_Ageddown	MTC
1.19E-06	-0.3950355	0.671	0.794	0.028531	Skp1a	MTC_Ageddown	MTC
2.21E-07	-0.4017426	0.314	0.536	0.005296	Dnajb11	MTC_Ageddown	MTC
6.32E-14	-0.4031896	0.973	0.949	1.52E-09	Rpl26	MTC_Ageddown	MTC
1.98E-07	-0.4071818	0.212	0.411	0.004758	Zkscan3	MTC_Ageddown	MTC
7.81E-07	-0.4162082	0.329	0.526	0.018729	Pdia4	MTC_Ageddown	MTC
9.02E-07	-0.4178175	0.388	0.573	0.02164	Psmd7	MTC_Ageddown	MTC
1.28E-06	-0.418149	0.294	0.502	0.030712	Ahsa1	MTC_Ageddown	MTC
1.27E-07	-0.4222026	0.373	0.593	0.003052	Cct3	MTC_Ageddown	MTC
5.60E-09	-0.4236432	0.796	0.893	0.000134	Ppib	MTC_Ageddown	MTC
4.94E-08	-0.4253978	0.278	0.508	0.001184	Map4k4	MTC_Ageddown	MTC
3.61E-07	-0.426567	0.42	0.607	0.008668	Cct4	MTC_Ageddown	MTC
5.24E-08	-0.4268969	0.337	0.557	0.001256	Psmc6	MTC_Ageddown	MTC
6.05E-08	-0.4329663	0.251	0.468	0.001451	Chordc1	MTC_Ageddown	MTC
4.14E-08	-0.4399578	0.396	0.615	0.000992	Prmt1	MTC_Ageddown	MTC
2.00E-10	-0.4420835	0.953	0.879	4.79E-06	Rpl17	MTC_Ageddown	MTC
1.60E-08	-0.4473666	0.31	0.542	0.000384	Casz1	MTC_Ageddown	MTC

6.77E-08	-0.4484393	0.29	0.512	0.001624	Rraga	MTC_Ageddown	MTC
1.98E-29	-0.4490705	1	1	4.75E-25	mt-Cytb	MTC_Ageddown	MTC
3.23E-07	-0.4590932	0.051	0.188	0.007748	Anxa8	MTC_Ageddown	MTC
2.22E-09	-0.4631356	0.078	0.265	5.33E-05	Gm26735	MTC_Ageddown	MTC
3.25E-09	-0.4653844	0.761	0.798	7.79E-05	Ybx1	MTC_Ageddown	MTC
8.02E-09	-0.4669857	0.22	0.447	0.000192	Ahsa2	MTC_Ageddown	MTC
1.51E-08	-0.4688781	0.157	0.366	0.000362	Sdf2l1	MTC_Ageddown	MTC
9.97E-11	-0.470828	0.231	0.494	2.39E-06	Eif1a	MTC_Ageddown	MTC
3.13E-07	-0.471375	0.443	0.634	0.007516	Psma4	MTC_Ageddown	MTC
1.49E-06	-0.4760564	0.388	0.585	0.035747	Eif5b	MTC_Ageddown	MTC
2.15E-08	-0.4802465	0.522	0.688	0.000516	Srsf2	MTC_Ageddown	MTC
3.29E-08	-0.4858457	0.616	0.769	0.000789	Pcbp2	MTC_Ageddown	MTC
2.41E-12	-0.4924736	0.886	0.87	5.77E-08	Rpl21	MTC_Ageddown	MTC
2.00E-06	-0.4934583	0.369	0.557	0.048035	Ythdc1	MTC_Ageddown	MTC
5.14E-12	-0.4956721	0.949	0.966	1.23E-07	Cd9	MTC_Ageddown	MTC
1.70E-10	-0.4994953	0.741	0.828	4.08E-06	Ywhaz	MTC_Ageddown	MTC
1.64E-08	-0.501797	0.557	0.715	0.000393	Hnrnpa0	MTC_Ageddown	MTC
7.14E-10	-0.5026305	0.714	0.808	1.71E-05	Slc25a5	MTC_Ageddown	MTC
1.09E-08	-0.5035464	0.49	0.668	0.000262	Ptges3	MTC_Ageddown	MTC
1.60E-10	-0.5138955	0.157	0.387	3.83E-06	Bnip2	MTC_Ageddown	MTC
2.47E-09	-0.5144363	0.482	0.672	5.92E-05	Eif3e	MTC_Ageddown	MTC
7.01E-08	-0.5151008	0.937	0.937	0.001681	Krt18	MTC_Ageddown	MTC
8.44E-10	-0.5180493	0.212	0.455	2.02E-05	Serpinh1	MTC_Ageddown	MTC
3.16E-08	-0.523661	0.212	0.423	0.000758	Tshz1	MTC_Ageddown	MTC
6.48E-14	-0.5237218	0.945	0.909	1.55E-09	Rplp1	MTC_Ageddown	MTC
1.77E-28	-0.5278024	0.988	0.976	4.24E-24	Tpt1	MTC_Ageddown	MTC
1.69E-08	-0.5346437	0.584	0.719	0.000404	Top1	MTC_Ageddown	MTC
3.68E-10	-0.5432249	0.173	0.411	8.82E-06	AY036118	MTC_Ageddown	MTC
2.26E-12	-0.549215	0.016	0.204	5.42E-08	Tgfb2	MTC_Ageddown	MTC
2.26E-12	-0.5606096	0.706	0.798	5.43E-08	Serbp1	MTC_Ageddown	MTC
1.08E-06	-0.5619105	0.231	0.409	0.025819	Krt20	MTC_Ageddown	MTC
6.02E-30	-0.5671405	0.973	0.978	1.44E-25	mt-Nd2	MTC_Ageddown	MTC
2.62E-19	-0.5717691	0.984	0.988	6.28E-15	Ubb	MTC_Ageddown	MTC
1.22E-10	-0.5746653	0.69	0.792	2.92E-06	Eif4a1	MTC_Ageddown	MTC
2.81E-23	-0.5763727	0.945	0.962	6.75E-19	H3f3a	MTC_Ageddown	MTC
9.54E-09	-0.5792505	0.671	0.743	0.000229	Hnrnpab	MTC_Ageddown	MTC
3.40E-11	-0.5801763	0.604	0.783	8.16E-07	Pcbp1	MTC_Ageddown	MTC
1.55E-10	-0.5955942	0.118	0.336	3.72E-06	Gt(ROSA)26Sor	MTC_Ageddown	MTC
1.95E-10	-0.6237722	0.812	0.824	4.68E-06	Rpl12	MTC_Ageddown	MTC
1.31E-10	-0.6272313	0.678	0.794	3.14E-06	Calr	MTC_Ageddown	MTC
1.31E-14	-0.6671031	0.408	0.684	3.15E-10	Cacybp	MTC_Ageddown	MTC
3.24E-15	-0.6829534	0.847	0.858	7.76E-11	Rps25	MTC_Ageddown	MTC
3.70E-09	-0.6935979	0.498	0.656	8.88E-05	Hnrnpa1	MTC_Ageddown	MTC
2.89E-12	-0.7001081	0.369	0.632	6.92E-08	Homer2	MTC_Ageddown	MTC
4.05E-14	-0.7150754	0.776	0.883	9.72E-10	Hspe1	MTC_Ageddown	MTC
9.38E-09	-0.7561544	0.827	0.899	0.000225	Cldn7	MTC_Ageddown	MTC
4.65E-13	-0.769543	0.678	0.767	1.11E-08	Rpl23a	MTC_Ageddown	MTC
2.58E-33	-0.7807457	0.961	0.937	6.19E-29	Rps21	MTC_Ageddown	MTC
2.19E-15	-0.8952894	0.451	0.735	5.25E-11	Sbpl	MTC_Ageddown	MTC
6.83E-15	-0.9208561	0.925	0.968	1.64E-10	Hspb1	MTC_Ageddown	MTC
1.87E-25	-0.9902519	0.412	0.773	4.48E-21	Tmem59	MTC_Ageddown	MTC
1.75E-20	-0.9973081	0.553	0.785	4.19E-16	Manf	MTC_Ageddown	MTC
2.74E-23	-1.0096714	0.749	0.854	6.58E-19	Hspa5	MTC_Ageddown	MTC
8.86E-32	-1.0332264	0.976	0.915	2.12E-27	Rps8	MTC_Ageddown	MTC
2.59E-53	-1.1131022	0.922	0.958	6.20E-49	Hspa8	MTC_Ageddown	MTC
2.21E-74	-1.116009	0.984	0.99	5.31E-70	Hsp90ab1	MTC_Ageddown	MTC
2.94E-28	-1.1168129	0.251	0.654	7.04E-24	Hsph1	MTC_Ageddown	MTC
1.00E-28	-1.1618743	0.8	0.85	2.40E-24	Rps12	MTC_Ageddown	MTC
3.95E-41	-1.1950159	0.694	0.874	9.48E-37	Dnaja1	MTC_Ageddown	MTC

4.04E-52	-1.4177959	0.812	0.903	9.70E-48	Hsp90aa1	MTC_Ageddown	MTC
5.66E-25	-1.5696537	0.094	0.466	1.36E-20	Hspa1a	MTC_Ageddown	MTC
8.67E-37	-2.1142103	0.149	0.615	2.08E-32	Hspa1b	MTC_Ageddown	MTC
8.79E-267	1.73396095	1	1	2.11E-262	Gm42418	SEC_Agedup	SEC
2.70E-199	1.66421269	0.801	0.357	6.47E-195	Ifi27l2a	SEC_Agedup	SEC
2.44E-134	1.61816819	0.239	0.001	5.85E-130	Xist	SEC_Agedup	SEC
4.73E-152	1.54643419	0.422	0.088	1.13E-147	1600014C10Rik	SEC_Agedup	SEC
3.63E-195	1.53882566	0.353	0.009	8.70E-191	Gm10260	SEC_Agedup	SEC
2.78E-162	1.3950648	0.759	0.468	6.66E-158	Ly6a	SEC_Agedup	SEC
2.98E-93	1.36492813	0.839	0.741	7.14E-89	Crip1	SEC_Agedup	SEC
1.51E-229	1.25434383	0.965	0.906	3.61E-225	mt-Atp8	SEC_Agedup	SEC
2.70E-50	1.20440486	0.456	0.255	6.47E-46	Ada	SEC_Agedup	SEC
4.07E-168	1.16877954	0.759	0.39	9.76E-164	B2m	SEC_Agedup	SEC
1.33E-56	1.07731319	0.961	0.908	3.19E-52	Krt4	SEC_Agedup	SEC
4.33E-167	1.05701799	0.889	0.725	1.04E-162	H2-K1	SEC_Agedup	SEC
2.42E-271	1.0313714	1	1	5.81E-267	mt-Nd4l	SEC_Agedup	SEC
2.81E-133	1.01237741	0.915	0.864	6.74E-129	H2-D1	SEC_Agedup	SEC
4.21E-101	0.92784441	0.764	0.607	1.01E-96	Tppp3	SEC_Agedup	SEC
6.05E-127	0.90963248	0.997	0.993	1.45E-122	Dbi	SEC_Agedup	SEC
4.50E-101	0.90523291	0.739	0.48	1.08E-96	Ly6e	SEC_Agedup	SEC
6.91E-110	0.9028287	0.748	0.578	1.66E-105	Gstp1	SEC_Agedup	SEC
1.22E-72	0.87922926	0.848	0.683	2.92E-68	Ifitm3	SEC_Agedup	SEC
8.71E-299	0.8470044	0.997	0.997	2.09E-294	Gm10076	SEC_Agedup	SEC
2.11E-105	0.79751373	0.225	0.013	5.07E-101	Tff2	SEC_Agedup	SEC
7.74E-108	0.6902912	0.263	0.028	1.86E-103	Muc5b	SEC_Agedup	SEC
7.85E-39	0.66056038	0.864	0.857	1.88E-34	Gja1	SEC_Agedup	SEC
1.18E-41	0.65540553	0.555	0.35	2.83E-37	Crabp2	SEC_Agedup	SEC
1.17E-35	0.64294461	0.615	0.469	2.80E-31	2610528A11Rik	SEC_Agedup	SEC
4.24E-44	0.63952533	0.541	0.414	1.02E-39	Dhcr24	SEC_Agedup	SEC
3.95E-59	0.63800671	0.362	0.168	9.47E-55	Alad	SEC_Agedup	SEC
5.52E-52	0.63144008	0.74	0.676	1.32E-47	Tmsb10	SEC_Agedup	SEC
3.88E-22	0.63025147	0.195	0.093	9.30E-18	Hbb-bs	SEC_Agedup	SEC
5.59E-83	0.60294418	0.94	0.951	1.34E-78	Cstb	SEC_Agedup	SEC
4.96E-37	0.59745511	0.274	0.114	1.19E-32	Isg15	SEC_Agedup	SEC
3.92E-57	0.58115605	0.94	0.829	9.40E-53	Calml3	SEC_Agedup	SEC
1.07E-63	0.58057675	0.742	0.672	2.57E-59	Psme2	SEC_Agedup	SEC
2.38E-35	0.5409367	0.541	0.407	5.70E-31	Dynap	SEC_Agedup	SEC
7.15E-24	0.53420607	0.663	0.553	1.71E-19	Igfbp2	SEC_Agedup	SEC
2.70E-31	0.51773091	0.578	0.485	6.47E-27	Scd2	SEC_Agedup	SEC
4.16E-71	0.51311415	0.324	0.107	9.99E-67	Pitx2	SEC_Agedup	SEC
1.26E-31	0.51288546	0.999	0.999	3.03E-27	Actb	SEC_Agedup	SEC
3.25E-15	0.51146142	0.27	0.172	7.79E-11	Sprr2a3	SEC_Agedup	SEC
7.24E-28	0.50555804	0.999	0.996	1.74E-23	Fabp5	SEC_Agedup	SEC
2.93E-174	0.49721719	1	1	7.03E-170	mt-Co1	SEC_Agedup	SEC
7.98E-29	0.49203526	0.891	0.838	1.91E-24	Mt4	SEC_Agedup	SEC
4.35E-52	0.47946153	0.775	0.727	1.04E-47	Srsf5	SEC_Agedup	SEC
6.03E-13	0.47467726	0.921	0.888	1.45E-08	Fam25c	SEC_Agedup	SEC
1.08E-75	0.47401604	0.938	0.927	2.59E-71	Prelid1	SEC_Agedup	SEC
1.95E-42	0.47168541	0.73	0.692	4.68E-38	Rsrp1	SEC_Agedup	SEC
5.06E-76	0.47020346	0.249	0.049	1.21E-71	Ly6c1	SEC_Agedup	SEC
7.15E-28	0.46698641	0.713	0.653	1.71E-23	Chit1	SEC_Agedup	SEC
1.64E-23	0.46558042	0.376	0.242	3.93E-19	Crabp1	SEC_Agedup	SEC
3.54E-21	0.44113828	0.945	0.945	8.49E-17	Mt1	SEC_Agedup	SEC
2.41E-29	0.43720264	0.562	0.46	5.79E-25	Gm47283	SEC_Agedup	SEC
1.90E-40	0.43141053	0.229	0.081	4.55E-36	Psmb8	SEC_Agedup	SEC
7.62E-56	0.43043662	0.989	0.994	1.83E-51	Txn1	SEC_Agedup	SEC
3.59E-47	0.42247115	0.958	0.955	8.60E-43	Crip2	SEC_Agedup	SEC
2.78E-33	0.42235953	0.904	0.862	6.67E-29	Cst3	SEC_Agedup	SEC
1.58E-40	0.42179232	0.385	0.22	3.78E-36	Cpxm2	SEC_Agedup	SEC

1.49E-24	0.41771793	0.714	0.637	3.58E-20	Fdps	SEC_Agedup	SEC
4.89E-21	0.41562665	0.893	0.87	1.17E-16	S100a6	SEC_Agedup	SEC
3.53E-26	0.41240904	0.693	0.686	8.46E-22	Erh	SEC_Agedup	SEC
1.13E-46	0.41054172	0.366	0.166	2.72E-42	Bst2	SEC_Agedup	SEC
4.87E-21	0.40831554	0.823	0.795	1.17E-16	Cdkn1a	SEC_Agedup	SEC
2.94E-53	0.40469588	0.258	0.078	7.05E-49	Psmb9	SEC_Agedup	SEC
2.39E-31	0.39779708	0.789	0.809	5.73E-27	Snrpg	SEC_Agedup	SEC
4.58E-39	0.39466627	0.793	0.8	1.10E-34	Ndufa3	SEC_Agedup	SEC
3.50E-23	0.39315237	0.71	0.652	8.40E-19	Barx2	SEC_Agedup	SEC
2.19E-29	0.39073652	0.703	0.678	5.25E-25	Ddb1	SEC_Agedup	SEC
2.95E-27	0.38866208	0.786	0.773	7.07E-23	Csnk2b	SEC_Agedup	SEC
5.61E-14	0.3867525	0.784	0.772	1.34E-09	Mt2	SEC_Agedup	SEC
1.77E-53	0.38671441	0.328	0.136	4.23E-49	Gm10036	SEC_Agedup	SEC
5.79E-21	0.38428394	0.839	0.874	1.39E-16	Tspo	SEC_Agedup	SEC
1.51E-16	0.38252198	0.467	0.402	3.62E-12	Fdft1	SEC_Agedup	SEC
9.48E-27	0.38140876	0.732	0.697	2.27E-22	Rab24	SEC_Agedup	SEC
8.97E-20	0.38124666	0.602	0.504	2.15E-15	Clic3	SEC_Agedup	SEC
7.56E-32	0.37973576	0.722	0.684	1.81E-27	1810022K09Rik	SEC_Agedup	SEC
2.23E-31	0.37792367	0.906	0.94	5.34E-27	Sdc1	SEC_Agedup	SEC
1.34E-13	0.37489684	0.646	0.665	3.20E-09	Fam129b	SEC_Agedup	SEC
4.98E-22	0.37353201	0.43	0.332	1.19E-17	Snhg20	SEC_Agedup	SEC
1.97E-30	0.36772059	0.734	0.711	4.72E-26	Lsm7	SEC_Agedup	SEC
4.72E-15	0.36321605	0.444	0.359	1.13E-10	Tmem176b	SEC_Agedup	SEC
6.75E-12	0.35849899	0.277	0.196	1.62E-07	Lor	SEC_Agedup	SEC
1.32E-16	0.35817317	0.484	0.42	3.17E-12	Acp5	SEC_Agedup	SEC
7.91E-27	0.35684371	0.74	0.757	1.90E-22	1810058I24Rik	SEC_Agedup	SEC
1.25E-70	0.35400094	0.988	0.997	3.00E-66	Cox6b1	SEC_Agedup	SEC
2.03E-66	0.35386384	0.238	0.054	4.88E-62	Clec11a	SEC_Agedup	SEC
1.83E-48	0.35281934	0.915	0.931	4.40E-44	Ndufa11	SEC_Agedup	SEC
1.97E-18	0.34868686	0.523	0.471	4.73E-14	Nsun2	SEC_Agedup	SEC
1.12E-123	0.34264627	1	1	2.70E-119	Rps28	SEC_Agedup	SEC
4.52E-16	0.3407216	0.885	0.922	1.08E-11	Gstm1	SEC_Agedup	SEC
1.88E-18	0.33844662	0.495	0.428	4.50E-14	Coro1c	SEC_Agedup	SEC
4.35E-30	0.33837382	0.871	0.922	1.04E-25	Ndufb8	SEC_Agedup	SEC
1.05E-10	0.33531796	0.493	0.464	2.51E-06	Cyp51	SEC_Agedup	SEC
4.88E-32	0.33438099	0.869	0.874	1.17E-27	Romo1	SEC_Agedup	SEC
1.34E-09	0.33422675	0.502	0.476	3.20E-05	Lars2	SEC_Agedup	SEC
6.45E-23	0.33415215	0.728	0.72	1.55E-18	Ddx3x	SEC_Agedup	SEC
1.71E-46	0.33395657	0.97	0.989	4.09E-42	Ndufa4	SEC_Agedup	SEC
5.70E-18	0.3336026	0.332	0.234	1.37E-13	Atf5	SEC_Agedup	SEC
7.09E-39	0.33269381	0.234	0.086	1.70E-34	Samd9l	SEC_Agedup	SEC
1.18E-06	0.33152731	0.506	0.484	0.028204	Fam57a	SEC_Agedup	SEC
2.50E-115	0.32677236	0.999	1	6.00E-111	Rpl38	SEC_Agedup	SEC
1.41E-14	0.32545019	0.487	0.438	3.37E-10	Tmem43	SEC_Agedup	SEC
1.75E-33	0.32510321	0.958	0.97	4.19E-29	Ldha	SEC_Agedup	SEC
9.74E-12	0.32294604	0.404	0.344	2.34E-07	Csrp1	SEC_Agedup	SEC
5.53E-36	0.31945277	0.924	0.962	1.33E-31	Atp5g3	SEC_Agedup	SEC
1.69E-27	0.31904689	0.92	0.923	4.05E-23	Tagln2	SEC_Agedup	SEC
4.79E-12	0.31528788	0.909	0.915	1.15E-07	Ftl1	SEC_Agedup	SEC
7.34E-17	0.3145199	0.653	0.665	1.76E-12	Eif3b	SEC_Agedup	SEC
1.81E-19	0.31393327	0.406	0.318	4.33E-15	Gale	SEC_Agedup	SEC
6.49E-15	0.31055534	0.466	0.416	1.56E-10	Scrib	SEC_Agedup	SEC
1.40E-07	0.30954348	0.601	0.644	0.003349	Prom2	SEC_Agedup	SEC
2.59E-40	0.30853113	0.955	0.977	6.20E-36	Atp5k	SEC_Agedup	SEC
1.81E-34	0.30683015	0.914	0.948	4.33E-30	Pebp1	SEC_Agedup	SEC
1.77E-44	0.3059761	0.972	0.99	4.24E-40	Selenow	SEC_Agedup	SEC
4.36E-55	0.30356899	0.981	0.995	1.05E-50	Uqcr11	SEC_Agedup	SEC
1.95E-17	0.30294889	0.739	0.763	4.68E-13	Eif4g1	SEC_Agedup	SEC
2.09E-25	0.30275618	0.292	0.169	5.00E-21	Snhg9	SEC_Agedup	SEC

3.47E-15	0.30237339	0.488	0.439	8.33E-11	Ppp4c	SEC_Agedup	SEC
6.23E-13	0.30198744	0.615	0.624	1.50E-08	Ociad2	SEC_Agedup	SEC
6.45E-17	0.30131209	0.435	0.361	1.55E-12	Scap	SEC_Agedup	SEC
3.22E-29	0.29877939	0.274	0.143	7.73E-25	Insl6	SEC_Agedup	SEC
7.42E-29	0.2964781	0.948	0.977	1.78E-24	Pkm	SEC_Agedup	SEC
1.34E-19	0.29564039	0.895	0.927	3.22E-15	Atp6v1g1	SEC_Agedup	SEC
1.12E-11	0.29532365	0.572	0.568	2.69E-07	Psme1	SEC_Agedup	SEC
5.16E-28	0.29370246	0.91	0.945	1.24E-23	Capg	SEC_Agedup	SEC
8.27E-55	0.29207549	0.995	0.999	1.98E-50	Rpl35	SEC_Agedup	SEC
3.59E-25	0.29194882	0.949	0.965	8.60E-21	Aldoa	SEC_Agedup	SEC
1.06E-16	0.29123474	0.834	0.902	2.55E-12	Ndufc2	SEC_Agedup	SEC
5.94E-15	0.28979024	0.73	0.746	1.42E-10	Elof1	SEC_Agedup	SEC
3.70E-18	0.28889766	0.78	0.803	8.88E-14	Hcfc1r1	SEC_Agedup	SEC
4.81E-21	0.28862913	0.828	0.861	1.15E-16	Ndufv3	SEC_Agedup	SEC
1.06E-35	0.28856092	0.963	0.986	2.53E-31	Uba52	SEC_Agedup	SEC
2.07E-30	0.28816604	0.993	0.999	4.96E-26	mt-Nd5	SEC_Agedup	SEC
2.15E-09	0.28763948	0.486	0.451	5.16E-05	Pmvk	SEC_Agedup	SEC
1.59E-11	0.28758209	0.567	0.564	3.82E-07	Eif4ebp1	SEC_Agedup	SEC
4.49E-07	0.28695023	0.388	0.365	0.010757	Tmem259	SEC_Agedup	SEC
1.12E-08	0.28688883	0.535	0.552	0.000269	Srebf2	SEC_Agedup	SEC
4.59E-26	0.286862	0.895	0.932	1.10E-21	Nop10	SEC_Agedup	SEC
1.90E-21	0.28648903	0.945	0.982	4.55E-17	Lmna	SEC_Agedup	SEC
5.00E-17	0.28613892	0.774	0.789	1.20E-12	Tmbim6	SEC_Agedup	SEC
2.45E-22	0.28517357	0.878	0.922	5.88E-18	Nhp2	SEC_Agedup	SEC
1.19E-24	0.28416334	0.109	0.029	2.86E-20	H19	SEC_Agedup	SEC
6.39E-19	0.28402664	0.441	0.35	1.53E-14	Kars	SEC_Agedup	SEC
1.20E-21	0.28299883	0.913	0.94	2.88E-17	Spint2	SEC_Agedup	SEC
1.10E-08	0.28257931	0.523	0.537	0.000264	Gm10073	SEC_Agedup	SEC
1.63E-16	0.28230006	0.687	0.694	3.91E-12	Got2	SEC_Agedup	SEC
1.21E-13	0.28210174	0.545	0.521	2.91E-09	Prr13	SEC_Agedup	SEC
3.49E-16	0.28162823	0.905	0.928	8.37E-12	Ctsd	SEC_Agedup	SEC
1.16E-16	0.28100614	0.725	0.769	2.79E-12	Pdcd5	SEC_Agedup	SEC
1.09E-08	0.27976031	0.344	0.298	0.000262	Pla2g4f	SEC_Agedup	SEC
1.69E-10	0.27954356	0.588	0.597	4.05E-06	Guk1	SEC_Agedup	SEC
6.29E-11	0.27932691	0.45	0.409	1.51E-06	Actr1a	SEC_Agedup	SEC
4.40E-17	0.27686187	0.276	0.184	1.06E-12	Crybg2	SEC_Agedup	SEC
1.01E-09	0.27621819	0.441	0.408	2.43E-05	Cdc34	SEC_Agedup	SEC
1.90E-12	0.27514295	0.59	0.574	4.55E-08	Dnajc19	SEC_Agedup	SEC
2.19E-18	0.27443187	0.895	0.928	5.26E-14	Reep5	SEC_Agedup	SEC
1.70E-32	0.27306373	0.956	0.985	4.07E-28	Rpl27	SEC_Agedup	SEC
5.74E-10	0.27213476	0.359	0.309	1.38E-05	Usp4	SEC_Agedup	SEC
4.78E-14	0.27185556	0.392	0.319	1.15E-09	Vars	SEC_Agedup	SEC
6.02E-12	0.2702088	0.483	0.451	1.44E-07	Fasn	SEC_Agedup	SEC
1.19E-52	0.26976388	0.999	1	2.86E-48	Rpl11	SEC_Agedup	SEC
7.18E-17	0.26853989	0.33	0.238	1.72E-12	Uck2	SEC_Agedup	SEC
4.30E-11	0.26797386	0.563	0.561	1.03E-06	Wdr1	SEC_Agedup	SEC
5.79E-25	0.26710793	0.965	0.986	1.39E-20	Cox5a	SEC_Agedup	SEC
1.46E-07	0.26624498	0.215	0.157	0.003498	Fosb	SEC_Agedup	SEC
2.17E-09	0.26480214	0.428	0.393	5.22E-05	DMuCr2	SEC_Agedup	SEC
4.36E-13	0.26455457	0.384	0.307	1.04E-08	Flna	SEC_Agedup	SEC
2.58E-10	0.26318409	0.216	0.144	6.20E-06	Igfbp7	SEC_Agedup	SEC
9.57E-38	0.26296867	0.984	0.995	2.29E-33	Pfn1	SEC_Agedup	SEC
6.44E-16	0.26269624	0.201	0.113	1.54E-11	Igfbp4	SEC_Agedup	SEC
3.59E-08	0.2625284	0.559	0.524	0.000862	Car12	SEC_Agedup	SEC
5.61E-15	0.26068413	0.701	0.695	1.35E-10	2-Mar	SEC_Agedup	SEC
1.75E-07	0.25842662	0.535	0.546	0.004201	Ptgfrn	SEC_Agedup	SEC
5.63E-09	0.25834278	0.341	0.294	0.000135	Rps6ka4	SEC_Agedup	SEC
6.65E-09	0.25678327	0.641	0.682	0.00016	Baiap2	SEC_Agedup	SEC
5.06E-10	0.25621854	0.466	0.436	1.21E-05	Kpnb1	SEC_Agedup	SEC

4.13E-08	0.25582576	0.384	0.344	0.00099	Ldlr	SEC_Agedup	SEC
9.66E-09	0.25567752	0.554	0.548	0.000232	Pdlim1	SEC_Agedup	SEC
1.95E-08	0.25389012	0.593	0.61	0.000467	Atf4	SEC_Agedup	SEC
1.55E-36	0.25305808	0.981	0.996	3.71E-32	Atp5g2	SEC_Agedup	SEC
1.07E-09	0.25045629	0.488	0.456	2.56E-05	Tnfrsf1a	SEC_Agedup	SEC
1.35E-08	0.24946591	0.434	0.39	0.000325	Shisa5	SEC_Agedup	SEC
8.35E-12	0.24915114	0.626	0.606	2.00E-07	Cd63	SEC_Agedup	SEC
1.56E-23	0.24885012	0.935	0.974	3.75E-19	Pabpc1	SEC_Agedup	SEC
6.99E-09	0.24781977	0.536	0.529	0.000168	Larp1	SEC_Agedup	SEC
7.54E-09	0.24545447	0.328	0.276	0.000181	Aacs	SEC_Agedup	SEC
8.77E-11	0.2453369	0.659	0.689	2.10E-06	Cs	SEC_Agedup	SEC
6.11E-15	0.24459561	0.325	0.234	1.47E-10	S100a1	SEC_Agedup	SEC
9.79E-15	0.24381323	0.795	0.819	2.35E-10	Fis1	SEC_Agedup	SEC
1.78E-15	0.2426112	0.27	0.176	4.26E-11	Tap1	SEC_Agedup	SEC
8.89E-44	0.242487	0.999	1	2.13E-39	Rpl18a	SEC_Agedup	SEC
5.84E-07	0.24234883	0.69	0.734	0.013996	Serp1	SEC_Agedup	SEC
3.33E-18	0.24227194	0.871	0.918	7.97E-14	Ost4	SEC_Agedup	SEC
1.02E-10	0.24208084	0.426	0.364	2.45E-06	Cluh	SEC_Agedup	SEC
1.46E-06	0.24139583	0.438	0.402	0.03508	Cavin1	SEC_Agedup	SEC
3.23E-22	0.24122876	0.132	0.048	7.76E-18	Cxcl14	SEC_Agedup	SEC
8.70E-10	0.23917136	0.703	0.734	2.09E-05	Sdhb	SEC_Agedup	SEC
6.87E-17	0.23813351	0.88	0.922	1.65E-12	Swi5	SEC_Agedup	SEC
2.84E-13	0.23795891	0.818	0.878	6.81E-09	Cyc1	SEC_Agedup	SEC
4.34E-07	0.23785378	0.673	0.726	0.010416	Hnrnpl	SEC_Agedup	SEC
8.77E-09	0.23726044	0.352	0.299	0.00021	Cpe	SEC_Agedup	SEC
2.38E-09	0.23598845	0.413	0.37	5.71E-05	Ncaph2	SEC_Agedup	SEC
5.27E-29	0.2359372	0.994	0.997	1.26E-24	Mif	SEC_Agedup	SEC
4.45E-12	0.23590162	0.197	0.121	1.07E-07	Crlf1	SEC_Agedup	SEC
9.61E-07	0.23570243	0.586	0.616	0.023052	Gas6	SEC_Agedup	SEC
4.28E-09	0.23559714	0.651	0.684	0.000103	Hdlbp	SEC_Agedup	SEC
1.01E-10	0.2336253	0.696	0.738	2.43E-06	Nenf	SEC_Agedup	SEC
2.34E-07	0.23334183	0.441	0.419	0.005603	Fosl2	SEC_Agedup	SEC
5.26E-07	0.23243104	0.493	0.465	0.012614	Sod1	SEC_Agedup	SEC
4.25E-39	0.2312961	0.999	0.999	1.02E-34	Rps19	SEC_Agedup	SEC
8.17E-12	0.23128282	0.24	0.16	1.96E-07	Cald1	SEC_Agedup	SEC
1.62E-17	0.23026598	0.911	0.958	3.89E-13	Snrpe	SEC_Agedup	SEC
4.50E-10	0.22981447	0.841	0.867	1.08E-05	Ifitm2	SEC_Agedup	SEC
2.12E-07	0.22967222	0.405	0.364	0.005081	Clec2d	SEC_Agedup	SEC
2.47E-08	0.22764638	0.935	0.953	0.000592	Fam162a	SEC_Agedup	SEC
9.05E-23	0.22758097	0.922	0.96	2.17E-18	Ndufs6	SEC_Agedup	SEC
2.37E-08	0.22709921	0.644	0.665	0.000569	Mrps34	SEC_Agedup	SEC
1.07E-14	0.22672325	0.848	0.876	2.56E-10	Akr1a1	SEC_Agedup	SEC
7.36E-10	0.22589057	0.759	0.812	1.76E-05	Aprt	SEC_Agedup	SEC
1.31E-22	0.22505896	0.189	0.087	3.13E-18	Akr1b8	SEC_Agedup	SEC
3.10E-19	0.22449346	0.965	0.98	7.44E-15	Pkp1	SEC_Agedup	SEC
1.87E-11	0.22395332	0.295	0.219	4.48E-07	Dcxr	SEC_Agedup	SEC
5.01E-08	0.2239464	0.59	0.604	0.001202	Mrps28	SEC_Agedup	SEC
1.91E-43	0.22350244	0.997	1	4.57E-39	Rpl37a	SEC_Agedup	SEC
2.26E-07	0.22195045	0.428	0.398	0.005421	Stat3	SEC_Agedup	SEC
4.10E-34	0.22151412	0.161	0.046	9.83E-30	Arhgdib	SEC_Agedup	SEC
9.71E-09	0.22132283	0.695	0.758	0.000233	Bcl7c	SEC_Agedup	SEC
9.68E-09	0.22050566	0.698	0.761	0.000232	Cuta	SEC_Agedup	SEC
5.53E-10	0.22041135	0.757	0.821	1.33E-05	Ndufb6	SEC_Agedup	SEC
6.21E-08	0.22015037	0.301	0.242	0.001489	Tmem176a	SEC_Agedup	SEC
7.95E-08	0.21970743	0.629	0.658	0.001907	Pet100	SEC_Agedup	SEC
1.36E-24	0.21921845	0.19	0.083	3.26E-20	Adat2	SEC_Agedup	SEC
3.51E-09	0.21919156	0.764	0.827	8.42E-05	Psap	SEC_Agedup	SEC
1.30E-06	0.21898385	0.75	0.787	0.031175	Hadh	SEC_Agedup	SEC
2.53E-15	0.21822862	0.13	0.059	6.06E-11	Peg3	SEC_Agedup	SEC

1.54E-14	0.21809806	0.227	0.14	3.68E-10	Ahnak2	SEC_Agedup	SEC
1.08E-11	0.21802797	0.841	0.904	2.59E-07	Uqcrc1	SEC_Agedup	SEC
3.03E-09	0.21765322	0.244	0.182	7.27E-05	Mvd	SEC_Agedup	SEC
1.80E-29	0.21733547	0.172	0.061	4.32E-25	Aknad1	SEC_Agedup	SEC
6.92E-42	0.21562793	0.114	0.014	1.66E-37	Gm11361	SEC_Agedup	SEC
4.65E-07	0.21478905	0.497	0.485	0.011162	Eif2s3x	SEC_Agedup	SEC
9.98E-10	0.21459928	0.257	0.19	2.39E-05	Psmb10	SEC_Agedup	SEC
4.65E-23	0.214158	0.168	0.069	1.11E-18	Tnni2	SEC_Agedup	SEC
2.19E-08	0.21356031	0.755	0.82	0.000526	Bzw1	SEC_Agedup	SEC
1.55E-15	0.21294962	0.891	0.948	3.71E-11	Mrpl52	SEC_Agedup	SEC
1.32E-13	0.21278206	0.845	0.863	3.17E-09	Ucp2	SEC_Agedup	SEC
7.32E-21	0.20917117	0.97	0.987	1.76E-16	Uqcrq	SEC_Agedup	SEC
1.20E-18	0.20779645	0.96	0.971	2.87E-14	Pgls	SEC_Agedup	SEC
4.04E-09	0.20640694	0.213	0.153	9.69E-05	Sp6	SEC_Agedup	SEC
8.85E-17	0.20549468	0.116	0.046	2.12E-12	Tnnc2	SEC_Agedup	SEC
9.37E-07	0.20479302	0.667	0.72	0.022476	Tmem147	SEC_Agedup	SEC
1.06E-33	0.20370646	0.998	0.999	2.55E-29	Rpl36	SEC_Agedup	SEC
1.90E-07	0.20331173	0.315	0.269	0.004548	Acaca	SEC_Agedup	SEC
5.90E-09	0.20294814	0.284	0.208	0.000142	Crct1	SEC_Agedup	SEC
3.76E-10	0.20191616	0.832	0.87	9.01E-06	Tecr	SEC_Agedup	SEC
1.53E-11	0.20109906	0.841	0.905	3.66E-07	Ndufb7	SEC_Agedup	SEC
1.97E-11	0.20010856	0.266	0.19	4.72E-07	Ppp6r1	SEC_Agedup	SEC
1.01E-07	0.20004458	0.746	0.818	0.002424	Cycs	SEC_Agedup	SEC
1.39E-25	-0.2000917	0.329	0.53	3.34E-21	Mxd4	SEC_Ageddown	SEC
6.80E-13	-0.200464	0.236	0.363	1.63E-08	Ier3	SEC_Ageddown	SEC
1.12E-12	-0.2008213	0.71	0.845	2.69E-08	Wnt4	SEC_Ageddown	SEC
3.94E-21	-0.2009788	0.319	0.5	9.45E-17	Pnisr	SEC_Ageddown	SEC
1.04E-23	-0.2016409	0.466	0.675	2.48E-19	Smap1	SEC_Ageddown	SEC
1.73E-28	-0.2021523	0.347	0.564	4.15E-24	Ahsa1	SEC_Ageddown	SEC
7.18E-22	-0.2022349	0.476	0.672	1.72E-17	Nktr	SEC_Ageddown	SEC
1.38E-17	-0.2028125	0.905	0.981	3.32E-13	Ybx3	SEC_Ageddown	SEC
1.90E-21	-0.2029998	0.852	0.951	4.57E-17	Hnrnpab	SEC_Ageddown	SEC
1.15E-27	-0.203015	0.357	0.584	2.76E-23	Tbc1d10a	SEC_Ageddown	SEC
8.63E-16	-0.2037188	0.684	0.841	2.07E-11	Hnrnpa1	SEC_Ageddown	SEC
1.99E-26	-0.2038255	0.258	0.454	4.77E-22	Ssbp2	SEC_Ageddown	SEC
9.32E-50	-0.2041357	0.041	0.222	2.23E-45	Lman2l	SEC_Ageddown	SEC
4.45E-26	-0.2042589	0.222	0.407	1.07E-21	2900026A02Rik	SEC_Ageddown	SEC
1.05E-28	-0.2045104	0.388	0.611	2.52E-24	Acp1	SEC_Ageddown	SEC
5.18E-28	-0.2046109	0.308	0.525	1.24E-23	Wbp4	SEC_Ageddown	SEC
2.11E-22	-0.2046408	0.383	0.583	5.07E-18	Senp6	SEC_Ageddown	SEC
1.41E-23	-0.2047926	0.881	0.971	3.38E-19	Ywhae	SEC_Ageddown	SEC
1.03E-21	-0.2049432	0.684	0.846	2.47E-17	Psmd8	SEC_Ageddown	SEC
6.38E-16	-0.2055594	0.833	0.937	1.53E-11	Set	SEC_Ageddown	SEC
5.50E-36	-0.2058951	0.12	0.303	1.32E-31	Pawr	SEC_Ageddown	SEC
4.07E-09	-0.2083382	0.358	0.473	9.75E-05	Gm26917	SEC_Ageddown	SEC
5.27E-10	-0.2083759	0.316	0.428	1.26E-05	Glul	SEC_Ageddown	SEC
3.39E-23	-0.2084988	0.578	0.777	8.12E-19	Pdia6	SEC_Ageddown	SEC
1.75E-31	-0.2088949	0.283	0.505	4.20E-27	Ccdc59	SEC_Ageddown	SEC
6.45E-23	-0.2089722	0.419	0.624	1.55E-18	Srpk2	SEC_Ageddown	SEC
1.36E-20	-0.2098203	0.996	1	3.27E-16	Dynll1	SEC_Ageddown	SEC
1.56E-49	-0.2110713	0.999	1	3.74E-45	Rpl23	SEC_Ageddown	SEC
1.32E-24	-0.2116903	0.487	0.709	3.17E-20	Dync1i2	SEC_Ageddown	SEC
1.84E-24	-0.2117793	0.541	0.754	4.41E-20	Klf3	SEC_Ageddown	SEC
9.81E-31	-0.2118969	0.992	0.999	2.35E-26	S100a11	SEC_Ageddown	SEC
3.04E-18	-0.2120342	0.163	0.293	7.28E-14	Sox4	SEC_Ageddown	SEC
1.64E-28	-0.2122166	0.247	0.445	3.94E-24	Baz1a	SEC_Ageddown	SEC
2.27E-22	-0.2123257	0.832	0.946	5.45E-18	Cebpb	SEC_Ageddown	SEC
1.63E-16	-0.2124631	0.387	0.543	3.92E-12	RMuCc	SEC_Ageddown	SEC
1.57E-20	-0.2124848	0.429	0.61	3.77E-16	Acin1	SEC_Ageddown	SEC

1.91E-36	-0.2132522	0.195	0.405	4.58E-32	Sdf2l1	SEC_Ageddown	SEC
6.30E-23	-0.2134886	0.739	0.899	1.51E-18	Erp29	SEC_Ageddown	SEC
2.45E-22	-0.213991	0.602	0.801	5.87E-18	Arhgap5	SEC_Ageddown	SEC
3.12E-24	-0.214191	0.953	0.98	7.48E-20	Calm1	SEC_Ageddown	SEC
2.83E-24	-0.2145567	0.852	0.963	6.79E-20	Hnrnpk	SEC_Ageddown	SEC
3.66E-25	-0.2150444	0.593	0.786	8.77E-21	Bcap31	SEC_Ageddown	SEC
2.09E-29	-0.2154668	0.397	0.631	5.01E-25	Gadd45gip1	SEC_Ageddown	SEC
1.10E-23	-0.2157947	0.599	0.812	2.64E-19	Hmgb2	SEC_Ageddown	SEC
7.32E-25	-0.2163582	0.541	0.761	1.76E-20	BC031181	SEC_Ageddown	SEC
2.14E-33	-0.2172421	0.318	0.553	5.14E-29	Cisd2	SEC_Ageddown	SEC
8.53E-38	-0.2173394	0.999	0.996	2.05E-33	Lgals7	SEC_Ageddown	SEC
3.25E-25	-0.2174959	0.564	0.774	7.78E-21	Srsf11	SEC_Ageddown	SEC
3.11E-36	-0.218432	0.241	0.471	7.45E-32	Med19	SEC_Ageddown	SEC
4.18E-33	-0.2186153	0.248	0.464	1.00E-28	Tef	SEC_Ageddown	SEC
1.36E-24	-0.2193795	0.176	0.336	3.27E-20	Odc1	SEC_Ageddown	SEC
1.99E-20	-0.2193957	0.696	0.866	4.77E-16	Srrm2	SEC_Ageddown	SEC
8.16E-36	-0.220633	0.999	1	1.96E-31	Rpl13	SEC_Ageddown	SEC
9.69E-25	-0.2216258	0.507	0.714	2.32E-20	Foxp1	SEC_Ageddown	SEC
5.96E-30	-0.2218676	0.229	0.43	1.43E-25	Eid1	SEC_Ageddown	SEC
5.65E-25	-0.2223182	0.158	0.313	1.35E-20	Hlf	SEC_Ageddown	SEC
9.42E-34	-0.2226981	0.262	0.48	2.26E-29	Mrps6	SEC_Ageddown	SEC
8.25E-24	-0.2240329	0.462	0.664	1.98E-19	Fubp1	SEC_Ageddown	SEC
8.32E-23	-0.2241108	0.687	0.879	2.00E-18	Cct2	SEC_Ageddown	SEC
9.65E-23	-0.2261368	0.606	0.798	2.32E-18	Ube2b	SEC_Ageddown	SEC
7.84E-21	-0.2265223	0.602	0.788	1.88E-16	Nfib	SEC_Ageddown	SEC
9.74E-34	-0.2265678	0.302	0.536	2.34E-29	Ppid	SEC_Ageddown	SEC
3.59E-23	-0.2266643	0.117	0.255	8.61E-19	Epha7	SEC_Ageddown	SEC
1.01E-15	-0.2270604	0.922	0.973	2.41E-11	Serpinb5	SEC_Ageddown	SEC
1.98E-06	-0.2272854	0.538	0.634	0.047595	Serpinb1a	SEC_Ageddown	SEC
6.89E-30	-0.2275702	0.217	0.414	1.65E-25	Mylip	SEC_Ageddown	SEC
1.67E-30	-0.2276579	0.665	0.867	4.01E-26	Brk1	SEC_Ageddown	SEC
1.17E-29	-0.2280506	0.916	0.98	2.81E-25	Hras	SEC_Ageddown	SEC
2.01E-22	-0.228392	0.233	0.395	4.83E-18	Trim2	SEC_Ageddown	SEC
4.04E-22	-0.2285173	0.647	0.833	9.69E-18	Sh3glb1	SEC_Ageddown	SEC
9.72E-27	-0.2285808	0.286	0.487	2.33E-22	Arl4a	SEC_Ageddown	SEC
2.25E-24	-0.2290158	0.412	0.613	5.41E-20	Acadl	SEC_Ageddown	SEC
2.52E-24	-0.2293457	0.495	0.699	6.03E-20	Sltm	SEC_Ageddown	SEC
1.12E-18	-0.2304363	0.718	0.869	2.68E-14	Sfr1	SEC_Ageddown	SEC
1.41E-22	-0.2306288	0.827	0.942	3.38E-18	Snrpf	SEC_Ageddown	SEC
1.13E-31	-0.2310336	0.125	0.298	2.71E-27	Crlf3	SEC_Ageddown	SEC
5.42E-37	-0.2315437	0.112	0.294	1.30E-32	Dnajb9	SEC_Ageddown	SEC
7.92E-27	-0.2316401	0.615	0.807	1.90E-22	Psmd4	SEC_Ageddown	SEC
3.72E-08	-0.2316536	0.375	0.465	0.000891	Maf	SEC_Ageddown	SEC
1.57E-29	-0.2317802	0.57	0.786	3.75E-25	Snx3	SEC_Ageddown	SEC
9.23E-37	-0.2318497	0.972	0.992	2.21E-32	Dstn	SEC_Ageddown	SEC
4.06E-26	-0.2323634	0.412	0.623	9.73E-22	Sgk1	SEC_Ageddown	SEC
1.11E-31	-0.2325687	0.247	0.456	2.65E-27	Ppm1l	SEC_Ageddown	SEC
6.02E-18	-0.2328712	0.474	0.646	1.44E-13	Paqr5	SEC_Ageddown	SEC
4.59E-28	-0.2337173	0.525	0.745	1.10E-23	Atxn7l3b	SEC_Ageddown	SEC
3.90E-31	-0.234675	0.291	0.504	9.35E-27	Mpp7	SEC_Ageddown	SEC
1.58E-22	-0.2348458	0.581	0.777	3.78E-18	Rbm25	SEC_Ageddown	SEC
4.05E-11	-0.2355749	0.387	0.509	9.70E-07	Col17a1	SEC_Ageddown	SEC
7.01E-31	-0.2358209	0.357	0.573	1.68E-26	Casz1	SEC_Ageddown	SEC
3.11E-23	-0.237422	0.249	0.421	7.46E-19	Tsc22d3	SEC_Ageddown	SEC
4.63E-26	-0.2382195	0.714	0.885	1.11E-21	Purb	SEC_Ageddown	SEC
2.21E-08	-0.2388173	0.468	0.621	0.00053	Bpifb1	SEC_Ageddown	SEC
1.69E-31	-0.2397933	0.495	0.724	4.05E-27	Psmc6	SEC_Ageddown	SEC
7.94E-07	-0.2415021	0.114	0.172	0.019031	Serpinb11	SEC_Ageddown	SEC
5.28E-25	-0.2416814	0.426	0.627	1.27E-20	Pnn	SEC_Ageddown	SEC

4.80E-23	-0.241686	0.703	0.869	1.15E-18	Phlda3	SEC_Ageddown	SEC
1.44E-38	-0.2417169	0.997	1	3.46E-34	Rps3a1	SEC_Ageddown	SEC
2.52E-18	-0.2420561	0.353	0.498	6.03E-14	Sdr16c5	SEC_Ageddown	SEC
3.38E-31	-0.2429142	0.437	0.673	8.10E-27	Vps36	SEC_Ageddown	SEC
2.31E-13	-0.2435695	0.365	0.499	5.54E-09	Kcnq1ot1	SEC_Ageddown	SEC
1.65E-37	-0.2437697	0.355	0.604	3.96E-33	Yipf4	SEC_Ageddown	SEC
4.40E-30	-0.2445138	0.7	0.886	1.06E-25	Scp2	SEC_Ageddown	SEC
5.14E-08	-0.244577	0.053	0.105	0.001233	BC100530	SEC_Ageddown	SEC
3.27E-19	-0.2446567	0.512	0.673	7.84E-15	Pdlim2	SEC_Ageddown	SEC
8.65E-24	-0.2451186	0.586	0.79	2.07E-19	Hnrnpc	SEC_Ageddown	SEC
1.96E-27	-0.2455052	0.922	0.984	4.70E-23	Klf5	SEC_Ageddown	SEC
7.95E-37	-0.2459374	0.161	0.365	1.91E-32	Id4	SEC_Ageddown	SEC
4.34E-29	-0.2469448	0.497	0.696	1.04E-24	Tprgl	SEC_Ageddown	SEC
2.43E-24	-0.24813	0.731	0.892	5.82E-20	Cox7a2l	SEC_Ageddown	SEC
3.60E-37	-0.2481578	0.331	0.572	8.64E-33	Strn3	SEC_Ageddown	SEC
1.13E-34	-0.248327	0.362	0.603	2.72E-30	Ammecr1	SEC_Ageddown	SEC
1.19E-29	-0.2496635	0.642	0.845	2.84E-25	Anxa7	SEC_Ageddown	SEC
1.58E-39	-0.2499789	0.222	0.451	3.78E-35	2510002D24Rik	SEC_Ageddown	SEC
3.03E-34	-0.2500917	0.493	0.74	7.27E-30	Ablim1	SEC_Ageddown	SEC
1.83E-33	-0.2507776	0.469	0.705	4.39E-29	Cldnd1	SEC_Ageddown	SEC
9.79E-10	-0.2509864	0.292	0.42	2.35E-05	Krt10	SEC_Ageddown	SEC
1.79E-36	-0.2510354	0.416	0.676	4.30E-32	Zmat2	SEC_Ageddown	SEC
4.42E-46	-0.2511337	0.242	0.511	1.06E-41	Rsl24d1	SEC_Ageddown	SEC
6.29E-42	-0.2513045	0.211	0.447	1.51E-37	Dnajb2	SEC_Ageddown	SEC
2.41E-31	-0.2514617	0.457	0.683	5.78E-27	Rp9	SEC_Ageddown	SEC
3.28E-33	-0.2519695	0.097	0.262	7.86E-29	Serpinh1	SEC_Ageddown	SEC
5.34E-46	-0.2524256	0.279	0.547	1.28E-41	Acaa1a	SEC_Ageddown	SEC
4.55E-10	-0.2525433	0.488	0.616	1.09E-05	Lgalsl	SEC_Ageddown	SEC
2.25E-27	-0.2535884	0.76	0.918	5.41E-23	Ptges3	SEC_Ageddown	SEC
1.35E-30	-0.2537016	0.593	0.811	3.23E-26	Dusp11	SEC_Ageddown	SEC
5.32E-33	-0.2541533	0.296	0.522	1.28E-28	Lap3	SEC_Ageddown	SEC
1.29E-21	-0.2552422	0.436	0.595	3.09E-17	Gsta2	SEC_Ageddown	SEC
1.80E-28	-0.2571301	0.726	0.891	4.32E-24	Eif5	SEC_Ageddown	SEC
6.11E-25	-0.2582299	0.742	0.9	1.46E-20	Pcbp2	SEC_Ageddown	SEC
1.15E-34	-0.2593904	0.189	0.393	2.76E-30	Rgma	SEC_Ageddown	SEC
1.86E-45	-0.2594802	0.189	0.426	4.47E-41	2310033P09Rik	SEC_Ageddown	SEC
1.15E-51	-0.2598554	0.107	0.332	2.77E-47	Gt(ROSA)26Sor	SEC_Ageddown	SEC
1.21E-64	-0.2605289	0.999	1	2.90E-60	Eef1a1	SEC_Ageddown	SEC
2.02E-34	-0.2617927	0.897	0.983	4.84E-30	mt-Nd3	SEC_Ageddown	SEC
1.34E-24	-0.2626827	0.66	0.844	3.22E-20	Nap1l1	SEC_Ageddown	SEC
3.07E-38	-0.2637098	0.34	0.585	7.37E-34	Dnajc21	SEC_Ageddown	SEC
1.25E-31	-0.2643024	0.971	0.989	2.99E-27	Lgals3	SEC_Ageddown	SEC
3.50E-30	-0.2648291	0.674	0.86	8.39E-26	Srp14	SEC_Ageddown	SEC
1.37E-32	-0.2655679	0.216	0.432	3.29E-28	Cldn10	SEC_Ageddown	SEC
1.37E-25	-0.2666916	0.791	0.92	3.28E-21	Calr	SEC_Ageddown	SEC
4.30E-27	-0.2669618	0.521	0.731	1.03E-22	Ncor1	SEC_Ageddown	SEC
4.21E-26	-0.267646	0.803	0.941	1.01E-21	Nsa2	SEC_Ageddown	SEC
2.75E-33	-0.2676604	0.362	0.582	6.59E-29	Carnmt1	SEC_Ageddown	SEC
3.42E-33	-0.2678128	0.353	0.577	8.19E-29	Hspa4l	SEC_Ageddown	SEC
2.50E-32	-0.2683586	0.862	0.948	6.00E-28	Pitx1	SEC_Ageddown	SEC
1.04E-30	-0.2687238	0.588	0.8	2.50E-26	Sri	SEC_Ageddown	SEC
1.13E-36	-0.269253	0.5	0.74	2.72E-32	Med21	SEC_Ageddown	SEC
2.77E-30	-0.2697812	0.642	0.853	6.65E-26	Psma4	SEC_Ageddown	SEC
3.04E-33	-0.2699738	0.599	0.821	7.29E-29	Sf3b1	SEC_Ageddown	SEC
6.40E-57	-0.2709755	1	1	1.53E-52	mt-Cytb	SEC_Ageddown	SEC
5.40E-31	-0.2729999	0.586	0.801	1.29E-26	Arglu1	SEC_Ageddown	SEC
1.28E-54	-0.2730791	0.994	0.999	3.07E-50	Rpl17	SEC_Ageddown	SEC
1.24E-15	-0.2733449	0.386	0.531	2.98E-11	Zfp36l2	SEC_Ageddown	SEC
5.92E-38	-0.2736347	0.233	0.454	1.42E-33	Cited2	SEC_Ageddown	SEC

1.40E-33	-0.2765864	0.657	0.843	3.35E-29	Spcs2	SEC_Ageddown	SEC
1.64E-35	-0.277135	0.38	0.613	3.92E-31	Arap2	SEC_Ageddown	SEC
2.00E-42	-0.277919	0.333	0.589	4.80E-38	Tmem33	SEC_Ageddown	SEC
4.43E-46	-0.2793587	0.973	0.997	1.06E-41	Cox4i1	SEC_Ageddown	SEC
2.21E-34	-0.2802408	0.598	0.815	5.30E-30	Aimp1	SEC_Ageddown	SEC
5.01E-39	-0.2810922	0.345	0.585	1.20E-34	Pon2	SEC_Ageddown	SEC
5.21E-71	-0.2812663	0.999	1	1.25E-66	Rps14	SEC_Ageddown	SEC
1.15E-06	-0.2813195	0.523	0.465	0.027656	Klk10	SEC_Ageddown	SEC
8.81E-33	-0.2827308	0.456	0.686	2.11E-28	Ppig	SEC_Ageddown	SEC
1.54E-29	-0.2829394	0.829	0.957	3.69E-25	Ybx1	SEC_Ageddown	SEC
5.50E-28	-0.2832282	0.216	0.411	1.32E-23	Alcam	SEC_Ageddown	SEC
4.28E-53	-0.2836569	0.126	0.365	1.03E-48	Nr1d1	SEC_Ageddown	SEC
2.90E-41	-0.283925	0.197	0.427	6.95E-37	Rassf9	SEC_Ageddown	SEC
3.13E-31	-0.2844991	0.595	0.788	7.50E-27	Cnih4	SEC_Ageddown	SEC
1.10E-36	-0.2845961	0.46	0.705	2.64E-32	Ubxn4	SEC_Ageddown	SEC
4.01E-35	-0.2855925	0.582	0.813	9.61E-31	Cct4	SEC_Ageddown	SEC
1.94E-33	-0.2868296	0.597	0.809	4.65E-29	Eif5b	SEC_Ageddown	SEC
3.92E-39	-0.287073	0.514	0.764	9.40E-35	Rbm8a	SEC_Ageddown	SEC
7.44E-42	-0.2882517	0.466	0.731	1.78E-37	Nudc	SEC_Ageddown	SEC
4.74E-18	-0.2885966	0.416	0.587	1.14E-13	Pof1b	SEC_Ageddown	SEC
5.66E-51	-0.2888303	0.24	0.509	1.36E-46	Lamtor3	SEC_Ageddown	SEC
1.72E-31	-0.2890402	0.721	0.889	4.12E-27	Paip2	SEC_Ageddown	SEC
5.66E-27	-0.2892101	0.85	0.954	1.36E-22	Ralbp1	SEC_Ageddown	SEC
1.09E-49	-0.2922474	0.389	0.675	2.62E-45	Hspb8	SEC_Ageddown	SEC
1.07E-07	-0.293639	0.774	0.85	0.002576	S100a10	SEC_Ageddown	SEC
2.58E-33	-0.2939461	0.228	0.432	6.19E-29	Gdpd1	SEC_Ageddown	SEC
2.71E-35	-0.2960493	0.623	0.841	6.51E-31	U2af1	SEC_Ageddown	SEC
1.76E-34	-0.2991199	0.459	0.693	4.21E-30	Rdx	SEC_Ageddown	SEC
3.08E-36	-0.301256	0.074	0.237	7.39E-32	Il1r2	SEC_Ageddown	SEC
2.06E-30	-0.3026231	0.499	0.717	4.94E-26	Btg1	SEC_Ageddown	SEC
1.16E-33	-0.3031344	0.145	0.325	2.78E-29	Id2	SEC_Ageddown	SEC
6.16E-27	-0.3041431	0.746	0.902	1.48E-22	Hspd1	SEC_Ageddown	SEC
2.00E-34	-0.306492	0.57	0.78	4.80E-30	Aldh3b2	SEC_Ageddown	SEC
2.31E-36	-0.3070431	0.552	0.776	5.55E-32	Atp6v1a	SEC_Ageddown	SEC
2.67E-41	-0.307686	0.53	0.757	6.40E-37	Chmp5	SEC_Ageddown	SEC
1.04E-22	-0.3076956	0.734	0.878	2.50E-18	Tacstd2	SEC_Ageddown	SEC
6.24E-33	-0.3082641	0.733	0.901	1.50E-28	Srsf2	SEC_Ageddown	SEC
1.64E-21	-0.308307	0.333	0.496	3.93E-17	Endou	SEC_Ageddown	SEC
6.56E-12	-0.3092291	0.523	0.622	1.57E-07	Cst6	SEC_Ageddown	SEC
1.44E-27	-0.3093202	0.474	0.686	3.46E-23	Blmh	SEC_Ageddown	SEC
9.97E-69	-0.3097246	0.076	0.328	2.39E-64	Ahsa2	SEC_Ageddown	SEC
2.75E-38	-0.3100812	0.64	0.825	6.60E-34	Ift20	SEC_Ageddown	SEC
2.02E-37	-0.3105545	0.801	0.954	4.84E-33	Hmgb1	SEC_Ageddown	SEC
7.64E-20	-0.3114535	0.753	0.84	1.83E-15	Ces1h	SEC_Ageddown	SEC
1.81E-34	-0.3119459	0.901	0.971	4.35E-30	Hspe1	SEC_Ageddown	SEC
2.03E-45	-0.3121722	0.794	0.934	4.87E-41	Rhoa	SEC_Ageddown	SEC
1.55E-33	-0.3132135	0.416	0.647	3.71E-29	Mmp2	SEC_Ageddown	SEC
4.69E-36	-0.3134678	0.48	0.717	1.12E-31	Luc7l3	SEC_Ageddown	SEC
8.82E-11	-0.3150047	0.441	0.558	2.12E-06	Klk8	SEC_Ageddown	SEC
2.81E-20	-0.315657	0.649	0.811	6.73E-16	Ces1d	SEC_Ageddown	SEC
2.33E-32	-0.3162664	0.838	0.929	5.60E-28	Capns2	SEC_Ageddown	SEC
3.31E-27	-0.3169986	0.552	0.747	7.95E-23	MuClm	SEC_Ageddown	SEC
8.62E-42	-0.3174117	0.192	0.415	2.07E-37	Dbp	SEC_Ageddown	SEC
2.02E-89	-0.317868	1	1	4.84E-85	mt-Atp6	SEC_Ageddown	SEC
8.46E-14	-0.3180518	0.552	0.628	2.03E-09	Krt32	SEC_Ageddown	SEC
1.06E-43	-0.3194542	0.843	0.946	2.55E-39	Arpc2	SEC_Ageddown	SEC
3.48E-35	-0.3198867	0.727	0.884	8.34E-31	Dynlt3	SEC_Ageddown	SEC
2.78E-40	-0.3211095	0.987	0.997	6.67E-36	Rps15	SEC_Ageddown	SEC
5.18E-45	-0.3227834	0.436	0.699	1.24E-40	Stip1	SEC_Ageddown	SEC

3.52E-76	-0.3229645	0.079	0.353	8.44E-72	Banp	SEC_Ageddown	SEC
5.54E-59	-0.3235189	0.994	0.999	1.33E-54	Rpl12	SEC_Ageddown	SEC
3.68E-38	-0.3256288	0.538	0.747	8.82E-34	Dnajb1	SEC_Ageddown	SEC
1.40E-45	-0.3257824	0.995	1	3.35E-41	Perp	SEC_Ageddown	SEC
6.38E-22	-0.3263356	0.21	0.369	1.53E-17	Socs2	SEC_Ageddown	SEC
1.54E-49	-0.3264418	0.899	0.99	3.69E-45	Slc25a5	SEC_Ageddown	SEC
9.25E-41	-0.3267444	0.17	0.379	2.22E-36	Cpn1	SEC_Ageddown	SEC
9.90E-76	-0.3272416	0.987	0.998	2.37E-71	Itm2b	SEC_Ageddown	SEC
1.55E-40	-0.3282278	0.54	0.777	3.72E-36	St13	SEC_Ageddown	SEC
1.18E-16	-0.3286319	0.642	0.775	2.82E-12	Klf4	SEC_Ageddown	SEC
8.01E-51	-0.3287665	0.234	0.491	1.92E-46	Tnrc6c	SEC_Ageddown	SEC
6.15E-35	-0.3316422	0.31	0.524	1.47E-30	Cux1	SEC_Ageddown	SEC
7.37E-53	-0.3326668	0.317	0.599	1.77E-48	Tnfaip8	SEC_Ageddown	SEC
1.17E-17	-0.3332667	0.556	0.722	2.82E-13	Sox2	SEC_Ageddown	SEC
1.45E-35	-0.3348579	0.462	0.695	3.47E-31	Ankrd11	SEC_Ageddown	SEC
8.95E-38	-0.3383374	0.84	0.951	2.15E-33	Gltp	SEC_Ageddown	SEC
6.03E-108	-0.3400042	0.998	1	1.45E-103	Rplp2	SEC_Ageddown	SEC
1.99E-74	-0.3410956	0.995	0.999	4.76E-70	Ptma	SEC_Ageddown	SEC
1.27E-50	-0.3447134	0.295	0.569	3.05E-46	Ankrd12	SEC_Ageddown	SEC
1.76E-56	-0.345685	0.338	0.64	4.23E-52	Pura	SEC_Ageddown	SEC
1.87E-44	-0.3461665	0.658	0.874	4.49E-40	Hnrnpa0	SEC_Ageddown	SEC
7.16E-39	-0.3463383	0.557	0.779	1.72E-34	Dapl1	SEC_Ageddown	SEC
1.63E-69	-0.3470015	0.949	0.997	3.92E-65	Eif4a1	SEC_Ageddown	SEC
2.27E-42	-0.3476194	0.701	0.889	5.44E-38	Metap2	SEC_Ageddown	SEC
6.87E-44	-0.3478491	0.179	0.419	1.65E-39	Pax1	SEC_Ageddown	SEC
1.16E-49	-0.3528905	0.649	0.882	2.79E-45	Psmd7	SEC_Ageddown	SEC
9.54E-10	-0.3560011	0.409	0.508	2.29E-05	Dsg1a	SEC_Ageddown	SEC
4.52E-61	-0.3612083	0.279	0.581	1.08E-56	Snrnp48	SEC_Ageddown	SEC
2.42E-44	-0.3612811	0.554	0.798	5.80E-40	Slc38a2	SEC_Ageddown	SEC
5.67E-61	-0.3627897	0.942	0.994	1.36E-56	Serbp1	SEC_Ageddown	SEC
1.48E-13	-0.3655404	0.611	0.723	3.54E-09	Selenbp1	SEC_Ageddown	SEC
2.00E-108	-0.3674407	1	1	4.80E-104	mt-Co3	SEC_Ageddown	SEC
1.07E-47	-0.3705085	0.558	0.812	2.56E-43	Spop	SEC_Ageddown	SEC
5.98E-51	-0.3709974	0.724	0.923	1.44E-46	Eif3e	SEC_Ageddown	SEC
5.98E-60	-0.3710259	0.72	0.922	1.43E-55	Pcbp1	SEC_Ageddown	SEC
1.62E-08	-0.3712135	0.733	0.802	0.000389	Krt5	SEC_Ageddown	SEC
7.64E-31	-0.3734375	0.536	0.724	1.83E-26	Golim4	SEC_Ageddown	SEC
3.01E-131	-0.3748503	0.998	1	7.22E-127	Rpl26	SEC_Ageddown	SEC
2.21E-33	-0.3785637	0.332	0.54	5.31E-29	Foxq1	SEC_Ageddown	SEC
2.18E-21	-0.378894	0.579	0.72	5.22E-17	Ctnnbip1	SEC_Ageddown	SEC
7.23E-69	-0.3806338	0.296	0.62	1.73E-64	Mettl23	SEC_Ageddown	SEC
5.14E-106	-0.3832665	0.999	1	1.23E-101	Rplp1	SEC_Ageddown	SEC
3.71E-45	-0.3833143	0.164	0.384	8.91E-41	Clu	SEC_Ageddown	SEC
1.46E-19	-0.3839743	0.604	0.771	3.49E-15	Junb	SEC_Ageddown	SEC
2.01E-51	-0.3840937	0.784	0.953	4.83E-47	Rbm39	SEC_Ageddown	SEC
5.08E-79	-0.3884627	0.974	0.996	1.22E-74	Ubb	SEC_Ageddown	SEC
3.23E-17	-0.3887899	0.502	0.646	7.75E-13	Csta1	SEC_Ageddown	SEC
9.33E-30	-0.3899134	0.979	0.998	2.24E-25	Gsto1	SEC_Ageddown	SEC
1.56E-38	-0.3960981	0.597	0.828	3.73E-34	Map1lc3a	SEC_Ageddown	SEC
3.26E-38	-0.3980517	0.387	0.614	7.81E-34	Txnip	SEC_Ageddown	SEC
2.35E-54	-0.3992849	0.678	0.901	5.64E-50	2010111I01Rik	SEC_Ageddown	SEC
8.40E-57	-0.399768	0.624	0.858	2.02E-52	Pdap1	SEC_Ageddown	SEC
1.14E-50	-0.405943	0.432	0.684	2.73E-46	Herpud1	SEC_Ageddown	SEC
1.49E-54	-0.4134313	0.67	0.886	3.57E-50	Top1	SEC_Ageddown	SEC
1.39E-27	-0.4143992	0.742	0.863	3.33E-23	Atp6v1e1	SEC_Ageddown	SEC
1.09E-48	-0.4171212	0.724	0.902	2.61E-44	Anp32b	SEC_Ageddown	SEC
1.32E-46	-0.4193562	0.317	0.585	3.16E-42	Tcf4	SEC_Ageddown	SEC
2.07E-47	-0.4197021	0.674	0.859	4.97E-43	Manf	SEC_Ageddown	SEC
2.64E-53	-0.4197286	0.818	0.952	6.33E-49	Skp1a	SEC_Ageddown	SEC

7.73E-103	-0.4223912	0.991	0.998	1.85E-98	Rps25	SEC_Ageddown	SEC
2.58E-122	-0.4239645	0.995	1	6.19E-118	Rpl21	SEC_Ageddown	SEC
8.34E-19	-0.4284631	0.224	0.358	2.00E-14	Il33	SEC_Ageddown	SEC
4.24E-53	-0.4294208	0.989	0.998	1.02E-48	Gsta4	SEC_Ageddown	SEC
1.43E-21	-0.438706	0.534	0.677	3.42E-17	Rbp2	SEC_Ageddown	SEC
1.81E-27	-0.4433692	0.88	0.986	4.35E-23	Malat1	SEC_Ageddown	SEC
4.09E-82	-0.4460546	0.27	0.607	9.81E-78	Zkscan3	SEC_Ageddown	SEC
1.43E-66	-0.4471607	0.733	0.927	3.44E-62	Rtn4	SEC_Ageddown	SEC
4.51E-110	-0.4499799	0.988	0.999	1.08E-105	Cd9	SEC_Ageddown	SEC
2.73E-21	-0.4538689	0.315	0.469	6.55E-17	Egr1	SEC_Ageddown	SEC
2.64E-205	-0.4551391	1	1	6.33E-201	Tpt1	SEC_Ageddown	SEC
1.38E-50	-0.4586164	0.721	0.893	3.30E-46	Hspa5	SEC_Ageddown	SEC
2.09E-49	-0.4675159	0.562	0.778	5.01E-45	Sptssb	SEC_Ageddown	SEC
1.46E-65	-0.4681157	0.759	0.925	3.49E-61	Nfe2l2	SEC_Ageddown	SEC
7.70E-19	-0.4762969	0.229	0.37	1.85E-14	Phlda1	SEC_Ageddown	SEC
3.67E-73	-0.4846975	0.275	0.588	8.79E-69	Gadd45b	SEC_Ageddown	SEC
1.04E-52	-0.4851016	0.344	0.616	2.50E-48	Lmo4	SEC_Ageddown	SEC
4.16E-187	-0.4989408	0.995	1	9.97E-183	Rps21	SEC_Ageddown	SEC
2.48E-102	-0.4997696	0.249	0.624	5.95E-98	Chordc1	SEC_Ageddown	SEC
1.20E-26	-0.5001602	0.429	0.612	2.88E-22	Dst	SEC_Ageddown	SEC
1.15E-63	-0.5067356	0.329	0.635	2.75E-59	Foxe1	SEC_Ageddown	SEC
7.87E-85	-0.5132735	0.538	0.831	1.89E-80	Fkbp4	SEC_Ageddown	SEC
4.59E-46	-0.5164527	0.995	0.998	1.10E-41	Hspb1	SEC_Ageddown	SEC
1.26E-124	-0.5186911	0.998	1	3.01E-120	mt-Nd2	SEC_Ageddown	SEC
4.47E-83	-0.5324172	0.778	0.934	1.07E-78	Anxa5	SEC_Ageddown	SEC
1.34E-71	-0.5375505	0.552	0.806	3.21E-67	Mafb	SEC_Ageddown	SEC
7.22E-80	-0.5383372	0.951	0.995	1.73E-75	Fxyd3	SEC_Ageddown	SEC
4.42E-23	-0.5409157	0.647	0.841	1.06E-18	Lipf	SEC_Ageddown	SEC
6.80E-88	-0.5489686	0.752	0.937	1.63E-83	Cbr3	SEC_Ageddown	SEC
3.13E-15	-0.5499055	0.418	0.527	7.51E-11	Tmprss11d	SEC_Ageddown	SEC
5.57E-97	-0.5567507	0.545	0.835	1.34E-92	Cacybp	SEC_Ageddown	SEC
8.44E-52	-0.5570967	0.367	0.614	2.02E-47	Ifi202b	SEC_Ageddown	SEC
2.51E-173	-0.563781	0.982	0.999	6.02E-169	H3f3a	SEC_Ageddown	SEC
2.01E-141	-0.608	0.97	0.997	4.82E-137	Rpl23a	SEC_Ageddown	SEC
6.28E-25	-0.6101181	0.461	0.626	1.51E-20	Spink5	SEC_Ageddown	SEC
1.28E-158	-0.6883998	0.385	0.848	3.06E-154	Tmem59	SEC_Ageddown	SEC
7.41E-98	-0.6901177	0.217	0.578	1.78E-93	Gadd45g	SEC_Ageddown	SEC
2.04E-215	-0.7341601	0.968	0.999	4.89E-211	Hspa8	SEC_Ageddown	SEC
6.66E-95	-0.7479191	0.729	0.915	1.60E-90	Aqp3	SEC_Ageddown	SEC
8.76E-15	-0.7699109	0.177	0.288	2.10E-10	Krt75	SEC_Ageddown	SEC
1.15E-282	-0.7897241	0.989	1	2.75E-278	Hsp90ab1	SEC_Ageddown	SEC
0	-0.7998455	0.998	1	0	Rps8	SEC_Ageddown	SEC
3.00E-78	-0.8487463	0.362	0.667	7.18E-74	Id3	SEC_Ageddown	SEC
3.57E-286	-0.8768776	0.996	0.999	8.57E-282	Rps12	SEC_Ageddown	SEC
3.10E-124	-0.8778258	0.608	0.9	7.44E-120	AY036118	SEC_Ageddown	SEC
7.90E-09	-0.9077465	0.059	0.117	0.000189	Psca	SEC_Ageddown	SEC
1.74E-125	-0.9195833	0.398	0.773	4.17E-121	Id1	SEC_Ageddown	SEC
1.29E-88	-0.9710105	0.886	0.972	3.10E-84	Krt6a	SEC_Ageddown	SEC
8.16E-205	-0.9922622	0.538	0.917	1.96E-200	Anxa8	SEC_Ageddown	SEC
1.76E-237	-1.0780924	0.274	0.784	4.21E-233	Hsph1	SEC_Ageddown	SEC
2.76E-81	-1.0966429	0.599	0.897	6.63E-77	Sbpl	SEC_Ageddown	SEC
1.18E-48	-1.0973965	0.529	0.802	2.84E-44	Krt17	SEC_Ageddown	SEC
6.13E-160	-1.1819219	0.823	0.95	1.47E-155	Adh7	SEC_Ageddown	SEC
1.17E-264	-1.1912741	0.482	0.93	2.81E-260	Dnaja1	SEC_Ageddown	SEC
8.49E-09	-1.3891766	0.128	0.204	0.000204	2300002M23Rik	SEC_Ageddown	SEC
0	-1.4848023	0.726	0.977	0	Hsp90aa1	SEC_Ageddown	SEC
2.48E-306	-1.6381348	0.315	0.853	5.95E-302	Hspa1a	SEC_Ageddown	SEC
0	-1.6695654	0.641	0.96	0	Hspa1b	SEC_Ageddown	SEC
5.16E-59	2.11289428	1	0.987	1.24E-54	Gm42418	TPC_Agedup	TPC

8.74E-23	1.79893375	0.66	0.308	2.10E-18	Ifi27l2a	TPC_Agedup	TPC
4.02E-78	1.79307401	0.438	0.01	9.65E-74	Gm10260	TPC_Agedup	TPC
2.93E-09	1.50170883	0.667	0.495	7.02E-05	Krt4	TPC_Agedup	TPC
3.29E-37	1.43655687	0.181	0	7.88E-33	Xist	TPC_Agedup	TPC
1.81E-42	1.37730927	0.965	0.825	4.33E-38	mt-Atp8	TPC_Agedup	TPC
9.07E-11	1.30232279	0.715	0.585	2.17E-06	Krt13	TPC_Agedup	TPC
1.25E-23	1.24349893	0.924	0.738	2.99E-19	Krtdap	TPC_Agedup	TPC
1.08E-06	1.20360796	0.833	0.869	0.025999	Crip1	TPC_Agedup	TPC
2.78E-09	0.98971144	0.785	0.757	6.68E-05	H2-D1	TPC_Agedup	TPC
9.68E-53	0.98942703	1	0.991	2.32E-48	Gm10076	TPC_Agedup	TPC
2.97E-51	0.97967532	1	1	7.12E-47	mt-Nd4l	TPC_Agedup	TPC
2.93E-27	0.97671654	0.285	0.039	7.03E-23	1600014C10Rik	TPC_Agedup	TPC
8.64E-20	0.97646229	0.382	0.107	2.07E-15	Calml3	TPC_Agedup	TPC
2.55E-17	0.90819043	0.924	0.723	6.11E-13	Fabp5	TPC_Agedup	TPC
1.72E-19	0.90120592	0.896	0.77	4.12E-15	Dbi	TPC_Agedup	TPC
5.07E-07	0.87688303	0.299	0.151	0.012154	Calm4	TPC_Agedup	TPC
9.32E-10	0.85074667	0.646	0.458	2.23E-05	B2m	TPC_Agedup	TPC
2.39E-15	0.84592479	0.667	0.402	5.72E-11	4631405K08Rik	TPC_Agedup	TPC
6.28E-14	0.83624803	0.701	0.511	1.51E-09	Cst3	TPC_Agedup	TPC
3.62E-16	0.81468131	0.806	0.646	8.69E-12	Prelid1	TPC_Agedup	TPC
2.59E-16	0.805653	0.819	0.657	6.22E-12	Fam162a	TPC_Agedup	TPC
1.59E-20	0.77683224	0.951	0.879	3.82E-16	Cox5a	TPC_Agedup	TPC
1.86E-26	0.75543281	0.993	0.923	4.46E-22	Cox5b	TPC_Agedup	TPC
8.89E-17	0.75059948	0.993	0.933	2.13E-12	Gpx2	TPC_Agedup	TPC
1.60E-09	0.74833934	0.389	0.196	3.84E-05	Tppp3	TPC_Agedup	TPC
7.34E-26	0.74090375	0.222	0.021	1.76E-21	Muc5b	TPC_Agedup	TPC
9.07E-18	0.72554225	0.438	0.164	2.17E-13	Snhg20	TPC_Agedup	TPC
5.07E-09	0.71082838	0.188	0.055	0.000121	Cenpa	TPC_Agedup	TPC
3.11E-17	0.69971899	0.896	0.729	7.45E-13	Ndufb8	TPC_Agedup	TPC
1.13E-06	0.69621469	0.535	0.358	0.027062	Sptssb	TPC_Agedup	TPC
3.04E-12	0.69137214	0.903	0.805	7.28E-08	Rbp1	TPC_Agedup	TPC
4.51E-14	0.68540547	0.465	0.226	1.08E-09	Gstp1	TPC_Agedup	TPC
3.77E-16	0.68202515	0.938	0.79	9.04E-12	Atp5k	TPC_Agedup	TPC
1.75E-09	0.67538005	0.958	0.951	4.19E-05	H2afz	TPC_Agedup	TPC
1.54E-17	0.67137499	0.931	0.82	3.69E-13	Uqcrq	TPC_Agedup	TPC
5.66E-11	0.6519842	0.708	0.535	1.36E-06	Mgst3	TPC_Agedup	TPC
6.76E-15	0.64863401	0.979	0.932	1.62E-10	Txn1	TPC_Agedup	TPC
3.04E-07	0.64621819	0.333	0.179	0.007283	Igfbp7	TPC_Agedup	TPC
2.54E-09	0.64593729	0.653	0.499	6.10E-05	Erh	TPC_Agedup	TPC
1.24E-11	0.63724966	0.632	0.445	2.97E-07	Psme2	TPC_Agedup	TPC
9.35E-13	0.62579786	0.757	0.557	2.24E-08	Pkp1	TPC_Agedup	TPC
9.49E-08	0.62081372	0.736	0.579	0.002276	H2-K1	TPC_Agedup	TPC
1.87E-08	0.61194596	0.639	0.485	0.000448	Ndufv3	TPC_Agedup	TPC
1.19E-06	0.61178057	0.215	0.088	0.028519	Mki67	TPC_Agedup	TPC
1.95E-11	0.61043375	0.847	0.712	4.68E-07	Cstb	TPC_Agedup	TPC
1.04E-18	0.60172686	0.979	0.886	2.51E-14	Uqcr10	TPC_Agedup	TPC
3.67E-09	0.60072811	0.688	0.54	8.81E-05	Selenoh	TPC_Agedup	TPC
2.03E-07	0.59527677	0.778	0.729	0.004864	Crip2	TPC_Agedup	TPC
9.17E-16	0.5949812	0.944	0.9	2.20E-11	Ndufa4	TPC_Agedup	TPC
5.49E-12	0.59273559	0.819	0.707	1.32E-07	Ndufs6	TPC_Agedup	TPC
7.18E-12	0.59144069	0.833	0.74	1.72E-07	Reep5	TPC_Agedup	TPC
1.07E-10	0.59030708	0.875	0.808	2.58E-06	Spint2	TPC_Agedup	TPC
1.82E-06	0.58081072	0.528	0.335	0.043562	Ces1f	TPC_Agedup	TPC
1.61E-26	0.579217	0.181	0.009	3.85E-22	Tff2	TPC_Agedup	TPC
3.34E-07	0.57783393	0.139	0.038	0.008017	Ccl20	TPC_Agedup	TPC
6.39E-26	0.57607089	1	1	1.53E-21	Ppia	TPC_Agedup	TPC
4.64E-18	0.57522005	0.986	0.977	1.11E-13	Pfn1	TPC_Agedup	TPC
1.84E-06	0.57269289	0.59	0.458	0.044135	Fgfbp1	TPC_Agedup	TPC
4.88E-12	0.57051036	0.417	0.187	1.17E-07	Tmprss4	TPC_Agedup	TPC

6.34E-10	0.56997599	0.674	0.521	1.52E-05	Ndufa3	TPC_Agedup	TPC
7.96E-14	0.56147435	0.938	0.893	1.91E-09	Ppp1r14b	TPC_Agedup	TPC
1.98E-10	0.55366543	0.84	0.738	4.76E-06	Ost4	TPC_Agedup	TPC
4.47E-16	0.55349367	0.944	0.886	1.07E-11	Atp5g1	TPC_Agedup	TPC
4.87E-10	0.54630557	0.84	0.768	1.17E-05	Snrpg	TPC_Agedup	TPC
8.39E-14	0.54247103	0.264	0.071	2.01E-09	Gm10036	TPC_Agedup	TPC
1.71E-11	0.53978355	0.889	0.839	4.09E-07	Edf1	TPC_Agedup	TPC
1.83E-07	0.53546559	0.368	0.2	0.004385	Ggh	TPC_Agedup	TPC
9.68E-17	0.53267762	0.986	0.944	2.32E-12	Cox6b1	TPC_Agedup	TPC
2.69E-07	0.52815196	0.646	0.534	0.006445	Fkbp2	TPC_Agedup	TPC
2.08E-09	0.5281326	0.771	0.653	4.99E-05	Ndufa1	TPC_Agedup	TPC
3.06E-08	0.52800261	0.785	0.67	0.000733	Atp6v1g1	TPC_Agedup	TPC
3.29E-08	0.52669262	0.854	0.772	0.00079	S100a16	TPC_Agedup	TPC
4.13E-10	0.52650013	0.243	0.082	9.90E-06	Msmo1	TPC_Agedup	TPC
4.12E-07	0.52575511	0.472	0.306	0.009869	Setd5	TPC_Agedup	TPC
7.43E-15	0.52469112	0.958	0.923	1.78E-10	Uqcr11	TPC_Agedup	TPC
1.20E-13	0.52155433	0.965	0.945	2.87E-09	Elob	TPC_Agedup	TPC
8.69E-08	0.51786844	0.778	0.655	0.002085	Romo1	TPC_Agedup	TPC
4.45E-14	0.51472008	0.993	0.961	1.07E-09	Cox6c	TPC_Agedup	TPC
1.39E-06	0.51468258	0.188	0.071	0.03339	Slpi	TPC_Agedup	TPC
5.25E-11	0.51096076	0.153	0.03	1.26E-06	Birc5	TPC_Agedup	TPC
2.17E-07	0.50645545	0.639	0.532	0.005201	Lsm7	TPC_Agedup	TPC
1.49E-10	0.50205565	0.896	0.818	3.57E-06	Atp5g3	TPC_Agedup	TPC
1.01E-06	0.50185592	0.757	0.67	0.024231	Aldoa	TPC_Agedup	TPC
2.94E-11	0.50050691	0.958	0.903	7.04E-07	Atp5j	TPC_Agedup	TPC
1.77E-46	0.49711575	1	1	4.24E-42	mt-Co1	TPC_Agedup	TPC
2.15E-12	0.49626762	1	0.998	5.15E-08	Ftl1	TPC_Agedup	TPC
1.06E-08	0.49420581	0.778	0.654	0.000255	Zfp706	TPC_Agedup	TPC
1.51E-06	0.49409281	0.604	0.468	0.036117	Ddb1	TPC_Agedup	TPC
1.21E-06	0.49350964	0.535	0.379	0.029046	H1f0	TPC_Agedup	TPC
1.40E-06	0.49075644	0.896	0.834	0.033454	Epcam	TPC_Agedup	TPC
3.21E-07	0.4902649	0.736	0.632	0.007706	Mrpl33	TPC_Agedup	TPC
4.35E-09	0.49025561	0.833	0.767	0.000104	Psmb6	TPC_Agedup	TPC
9.00E-14	0.48849874	0.979	0.962	2.16E-09	Atp5j2	TPC_Agedup	TPC
2.19E-08	0.48029569	0.694	0.557	0.000525	Naa38	TPC_Agedup	TPC
6.42E-12	0.4774183	0.965	0.921	1.54E-07	Cox7a2	TPC_Agedup	TPC
2.89E-10	0.47286906	0.931	0.887	6.93E-06	Ndufb9	TPC_Agedup	TPC
1.21E-15	0.46826423	0.986	0.985	2.91E-11	Atp5g2	TPC_Agedup	TPC
1.09E-06	0.4658565	0.736	0.65	0.02603	Ndufb2	TPC_Agedup	TPC
7.94E-10	0.4652324	0.861	0.795	1.90E-05	Ndufa11	TPC_Agedup	TPC
6.08E-07	0.46465439	0.639	0.49	0.014585	Csnk2b	TPC_Agedup	TPC
7.15E-07	0.46408949	0.75	0.65	0.017153	Ndufa12	TPC_Agedup	TPC
1.56E-09	0.46346492	0.91	0.845	3.74E-05	Sec61b	TPC_Agedup	TPC
3.75E-10	0.46331657	0.972	0.946	8.98E-06	Sem1	TPC_Agedup	TPC
5.96E-09	0.46104831	0.785	0.689	0.000143	Taf10	TPC_Agedup	TPC
1.43E-07	0.45696227	0.868	0.845	0.003421	Clic1	TPC_Agedup	TPC
2.84E-07	0.45422097	0.812	0.73	0.006821	D8Ertd738e	TPC_Agedup	TPC
5.36E-08	0.45363545	0.833	0.732	0.001285	Mdh2	TPC_Agedup	TPC
2.91E-14	0.44952626	1	0.993	6.99E-10	Oaz1	TPC_Agedup	TPC
3.40E-07	0.44874756	0.792	0.72	0.008156	Trmt112	TPC_Agedup	TPC
9.50E-09	0.4438541	0.806	0.718	0.000228	Usmg5	TPC_Agedup	TPC
4.98E-07	0.44215523	0.924	0.867	0.011931	Sfn	TPC_Agedup	TPC
2.16E-11	0.44093745	0.979	0.97	5.17E-07	Prdx1	TPC_Agedup	TPC
1.64E-08	0.43775461	0.486	0.293	0.000393	Bola3	TPC_Agedup	TPC
7.75E-11	0.43683838	0.16	0.034	1.86E-06	Psmb9	TPC_Agedup	TPC
3.45E-07	0.43170495	0.66	0.514	0.008266	Mrpl57	TPC_Agedup	TPC
4.01E-07	0.42974923	0.417	0.245	0.009627	Lasp1	TPC_Agedup	TPC
1.83E-07	0.42821093	0.792	0.674	0.00439	Avpi1	TPC_Agedup	TPC
1.86E-06	0.42335444	0.75	0.645	0.044613	Ndufa5	TPC_Agedup	TPC

2.24E-07	0.42276868	0.743	0.612	0.005364	Nhp2	TPC_Agedup	TPC
1.32E-06	0.42177503	0.951	0.915	0.031583	Sec61g	TPC_Agedup	TPC
2.98E-08	0.42149729	0.875	0.761	0.000714	Nme1	TPC_Agedup	TPC
6.12E-09	0.42101617	0.938	0.928	0.000147	Uqcrb	TPC_Agedup	TPC
1.77E-06	0.42087279	0.882	0.849	0.042428	Ran	TPC_Agedup	TPC
3.77E-12	0.41927247	0.979	0.961	9.03E-08	Uba52	TPC_Agedup	TPC
9.90E-07	0.41420074	0.806	0.708	0.023745	Cox6a1	TPC_Agedup	TPC
1.61E-06	0.41419583	0.688	0.562	0.038586	Mrpl54	TPC_Agedup	TPC
5.66E-11	0.41375752	0.979	0.972	1.36E-06	Hint1	TPC_Agedup	TPC
8.12E-07	0.40766568	0.757	0.65	0.019461	Ndufb3	TPC_Agedup	TPC
3.42E-11	0.40763435	0.16	0.032	8.19E-07	Samd9l	TPC_Agedup	TPC
6.68E-07	0.40594119	0.778	0.695	0.016018	Spcs1	TPC_Agedup	TPC
2.86E-07	0.3989581	0.917	0.891	0.00687	Mrpl52	TPC_Agedup	TPC
1.10E-06	0.39640236	0.812	0.772	0.026393	Dad1	TPC_Agedup	TPC
8.77E-12	0.39631487	0.201	0.049	2.10E-07	Plbd1	TPC_Agedup	TPC
1.84E-07	0.38824391	0.861	0.839	0.004416	2010107E04Rik	TPC_Agedup	TPC
4.12E-08	0.37868127	0.986	0.961	0.000988	Selenow	TPC_Agedup	TPC
2.23E-08	0.37350143	0.965	0.948	0.000534	Mif	TPC_Agedup	TPC
1.46E-09	0.37255163	0.194	0.056	3.50E-05	Cenpw	TPC_Agedup	TPC
4.30E-07	0.37240382	0.917	0.794	0.010317	Psmb5	TPC_Agedup	TPC
7.55E-07	0.37000929	0.868	0.858	0.018115	Nedd8	TPC_Agedup	TPC
3.31E-11	0.36361008	0.146	0.026	7.95E-07	Ndufa4l2	TPC_Agedup	TPC
1.02E-06	0.36068386	0.632	0.476	0.024404	Higd1a	TPC_Agedup	TPC
2.35E-07	0.3551455	0.965	0.968	0.005637	Serf2	TPC_Agedup	TPC
1.42E-25	0.35291928	1	1	3.42E-21	Rpl11	TPC_Agedup	TPC
4.24E-08	0.34584971	0.979	0.967	0.001016	Atp5e	TPC_Agedup	TPC
1.15E-07	0.34041097	0.965	0.935	0.00275	Atp5d	TPC_Agedup	TPC
1.61E-08	0.32135367	0.118	0.024	0.000385	Dhcr24	TPC_Agedup	TPC
4.41E-08	0.31885763	0.979	0.974	0.001058	Atp5h	TPC_Agedup	TPC
1.36E-16	0.31409272	1	1	3.26E-12	Rps2	TPC_Agedup	TPC
2.15E-10	0.30247641	0.125	0.021	5.15E-06	Slfn2	TPC_Agedup	TPC
1.07E-08	0.30229979	0.125	0.026	0.000256	Slc6a11	TPC_Agedup	TPC
6.39E-08	0.30204304	0.181	0.056	0.001534	Adat2	TPC_Agedup	TPC
7.71E-08	0.29810643	0.958	0.95	0.00185	Snrpe	TPC_Agedup	TPC
3.13E-07	0.29500146	0.993	0.984	0.007502	Atpif1	TPC_Agedup	TPC
6.77E-09	0.29090321	0.979	0.994	0.000162	Cox8a	TPC_Agedup	TPC
5.26E-18	0.2791851	1	0.999	1.26E-13	Rps28	TPC_Agedup	TPC
8.86E-08	0.27601509	0.139	0.035	0.002124	Tnnc2	TPC_Agedup	TPC
8.61E-17	0.27162222	1	1	2.07E-12	Rps29	TPC_Agedup	TPC
3.55E-16	0.25996274	1	1	8.50E-12	Rps19	TPC_Agedup	TPC
1.00E-11	0.25381183	1	1	2.40E-07	Rpl37a	TPC_Agedup	TPC
1.10E-06	0.25257399	0.104	0.024	0.026402	Cdkn3	TPC_Agedup	TPC
3.11E-10	0.23593748	1	1	7.45E-06	Rpl38	TPC_Agedup	TPC
3.96E-07	0.22085048	1	1	0.009494	Rpl35	TPC_Agedup	TPC
2.04E-18	0.21989791	1	1	4.89E-14	mt-Co2	TPC_Agedup	TPC
1.69E-10	0.21089542	1	1	4.06E-06	Rps20	TPC_Agedup	TPC
1.79E-09	-0.2025981	1	0.999	4.28E-05	Rpl24	TPC_Ageddown	TPC
4.07E-11	-0.2027664	1	1	9.76E-07	Rpl13	TPC_Ageddown	TPC
1.91E-09	-0.2393734	0.993	1	4.59E-05	Rps18	TPC_Ageddown	TPC
2.47E-07	-0.2624635	0.993	0.997	0.005917	Npm1	TPC_Ageddown	TPC
2.47E-16	-0.2721229	1	1	5.92E-12	mt-Nd4	TPC_Ageddown	TPC
9.06E-08	-0.2746403	0.986	0.999	0.002173	H3f3a	TPC_Ageddown	TPC
1.07E-11	-0.2771488	0.993	1	2.56E-07	Rpl10	TPC_Ageddown	TPC
1.93E-19	-0.2783609	1	1	4.63E-15	mt-Cytb	TPC_Ageddown	TPC
4.58E-21	-0.291337	1	1	1.10E-16	Rpl23	TPC_Ageddown	TPC
1.57E-30	-0.2971654	1	1	3.77E-26	mt-Atp6	TPC_Ageddown	TPC
5.11E-07	-0.3088623	0.917	0.974	0.012255	Eef1d	TPC_Ageddown	TPC
1.67E-07	-0.3198456	0.889	0.981	0.003995	mt-Nd3	TPC_Ageddown	TPC
1.04E-17	-0.3460755	1	1	2.49E-13	Rpl21	TPC_Ageddown	TPC

1.39E-07	-0.3729543	0.722	0.891	0.003336	Hnrnpa0	TPC_Ageddown	TPC
3.48E-21	-0.3740521	1	1	8.35E-17	Rplp2	TPC_Ageddown	TPC
9.47E-08	-0.3919137	0.493	0.765	0.002272	Purb	TPC_Ageddown	TPC
1.46E-06	-0.393133	0.507	0.722	0.035091	Psma4	TPC_Ageddown	TPC
4.49E-09	-0.4045091	0.91	0.958	0.000108	Hspe1	TPC_Ageddown	TPC
2.08E-06	-0.4046298	0.438	0.651	0.049977	Tra2a	TPC_Ageddown	TPC
9.69E-08	-0.4151983	0.75	0.909	0.002325	Hnrnpa1	TPC_Ageddown	TPC
4.37E-10	-0.4165064	0.951	0.961	1.05E-05	Serbp1	TPC_Ageddown	TPC
2.19E-07	-0.4190787	0.444	0.686	0.005258	Rp9	TPC_Ageddown	TPC
1.34E-06	-0.4240639	0.146	0.359	0.032234	Dbp	TPC_Ageddown	TPC
9.36E-31	-0.4296354	1	1	2.24E-26	Rpl26	TPC_Ageddown	TPC
3.85E-07	-0.4322058	0.361	0.622	0.009239	Fam3c	TPC_Ageddown	TPC
2.40E-07	-0.4386122	0.639	0.842	0.005765	Rhoa	TPC_Ageddown	TPC
1.40E-07	-0.4395351	0.264	0.533	0.003362	Mettl23	TPC_Ageddown	TPC
6.66E-07	-0.4416361	0.132	0.345	0.015964	Mllt3	TPC_Ageddown	TPC
7.83E-07	-0.4522591	0.389	0.63	0.018778	Tsc22d3	TPC_Ageddown	TPC
3.11E-07	-0.4524813	0.465	0.704	0.007468	Ncor1	TPC_Ageddown	TPC
6.52E-07	-0.4604839	0.111	0.31	0.015642	Actn1	TPC_Ageddown	TPC
2.84E-35	-0.463219	1	0.999	6.82E-31	Rps14	TPC_Ageddown	TPC
9.32E-34	-0.4695074	1	1	2.23E-29	Rpl17	TPC_Ageddown	TPC
2.39E-08	-0.4710063	0.771	0.903	0.000574	Nfix	TPC_Ageddown	TPC
5.90E-48	-0.4882507	1	1	1.41E-43	mt-Co3	TPC_Ageddown	TPC
9.79E-07	-0.4912402	0.826	0.9	0.023469	Ptn	TPC_Ageddown	TPC
8.98E-07	-0.4976926	0.424	0.663	0.02154	Arid5b	TPC_Ageddown	TPC
1.52E-06	-0.4985027	0.181	0.395	0.036399	Fjx1	TPC_Ageddown	TPC
2.15E-39	-0.4989771	1	1	5.15E-35	Eef1a1	TPC_Ageddown	TPC
4.99E-10	-0.5029238	0.965	0.999	1.20E-05	Hspb1	TPC_Ageddown	TPC
1.18E-25	-0.5068271	1	0.998	2.84E-21	Rack1	TPC_Ageddown	TPC
3.58E-07	-0.5073158	0.604	0.786	0.008582	Lmo4	TPC_Ageddown	TPC
3.02E-07	-0.5188647	0.236	0.478	0.007236	Herpud1	TPC_Ageddown	TPC
2.28E-11	-0.5197747	0.701	0.878	5.48E-07	Eif3e	TPC_Ageddown	TPC
5.56E-09	-0.525042	0.382	0.634	0.000133	Nudc	TPC_Ageddown	TPC
2.82E-13	-0.5290167	0.972	0.995	6.76E-09	Ubb	TPC_Ageddown	TPC
1.11E-27	-0.5300047	1	0.998	2.65E-23	Rpl12	TPC_Ageddown	TPC
4.03E-34	-0.5399827	1	1	9.67E-30	mt-Nd2	TPC_Ageddown	TPC
2.77E-40	-0.5421519	1	1	6.64E-36	Rps21	TPC_Ageddown	TPC
2.95E-07	-0.5528216	0.208	0.44	0.007068	Zfp503	TPC_Ageddown	TPC
1.79E-08	-0.5554117	0.514	0.755	0.00043	Tra2b	TPC_Ageddown	TPC
1.65E-07	-0.5606476	0.292	0.528	0.003957	Snai2	TPC_Ageddown	TPC
3.30E-09	-0.5627355	0.312	0.585	7.91E-05	Kmt2a	TPC_Ageddown	TPC
8.14E-11	-0.5849263	0.319	0.64	1.95E-06	Tnrc6c	TPC_Ageddown	TPC
1.53E-09	-0.5941594	0.722	0.878	3.67E-05	Txnip	TPC_Ageddown	TPC
2.78E-11	-0.598142	0.583	0.774	6.67E-07	Cct4	TPC_Ageddown	TPC
1.54E-14	-0.5992717	0.736	0.931	3.70E-10	Nsa2	TPC_Ageddown	TPC
4.90E-11	-0.5997963	0.84	0.969	1.18E-06	Ddx5	TPC_Ageddown	TPC
1.83E-10	-0.5999606	0.16	0.457	4.39E-06	Ifi202b	TPC_Ageddown	TPC
1.11E-07	-0.6080868	0.229	0.468	0.002673	Dusp6	TPC_Ageddown	TPC
2.10E-25	-0.6107276	0.986	0.995	5.04E-21	Rps25	TPC_Ageddown	TPC
2.39E-09	-0.6447077	0.076	0.317	5.73E-05	Nr1d1	TPC_Ageddown	TPC
3.08E-50	-0.6535675	1	1	7.39E-46	Tpt1	TPC_Ageddown	TPC
9.48E-08	-0.6623556	0.146	0.37	0.002274	Klf9	TPC_Ageddown	TPC
1.40E-06	-0.6671661	0.285	0.508	0.033657	Runx1	TPC_Ageddown	TPC
1.03E-08	-0.6700054	0.618	0.851	0.000247	Cebpb	TPC_Ageddown	TPC
1.36E-15	-0.6854495	0.826	0.961	3.27E-11	Nfib	TPC_Ageddown	TPC
1.68E-48	-0.7030696	1	0.999	4.03E-44	Rplp1	TPC_Ageddown	TPC
2.97E-19	-0.729235	0.681	0.917	7.13E-15	Pcbp2	TPC_Ageddown	TPC
1.21E-20	-0.7321875	0.993	0.993	2.91E-16	Rps15	TPC_Ageddown	TPC
6.38E-08	-0.7342212	0.319	0.592	0.001531	Nfkbia	TPC_Ageddown	TPC
1.00E-16	-0.7429881	0.382	0.778	2.40E-12	Tmem59	TPC_Ageddown	TPC

7.02E-12	-0.7441056	0.576	0.805	1.68E-07	Zfp36l2	TPC_Ageddown	TPC
1.79E-10	-0.7472729	0.375	0.666	4.30E-06	Nrip1	TPC_Ageddown	TPC
4.13E-12	-0.7585253	0.972	0.994	9.90E-08	Malat1	TPC_Ageddown	TPC
1.09E-07	-0.7611904	0.333	0.576	0.002621	Hpgd	TPC_Ageddown	TPC
9.16E-25	-0.7708874	0.924	0.986	2.20E-20	Rpl23a	TPC_Ageddown	TPC
1.62E-10	-0.7713824	0.549	0.77	3.89E-06	Sox4	TPC_Ageddown	TPC
1.00E-33	-0.7730237	0.986	0.997	2.40E-29	Hspa8	TPC_Ageddown	TPC
9.97E-08	-0.7834724	0.479	0.706	0.002391	Zfp36l1	TPC_Ageddown	TPC
1.07E-06	-0.7908989	0.194	0.396	0.025609	Nfkbiz	TPC_Ageddown	TPC
1.70E-06	-0.7980312	0.472	0.654	0.04067	Htra1	TPC_Ageddown	TPC
1.26E-17	-0.8005684	0.715	0.932	3.03E-13	Tcf4	TPC_Ageddown	TPC
2.72E-09	-0.8007576	0.236	0.51	6.53E-05	Sox9	TPC_Ageddown	TPC
1.07E-11	-0.806738	0.201	0.522	2.57E-07	Id2	TPC_Ageddown	TPC
2.32E-16	-0.818225	0.757	0.909	5.55E-12	MuClm	TPC_Ageddown	TPC
5.50E-08	-0.820297	0.458	0.677	0.001319	Ier2	TPC_Ageddown	TPC
1.73E-18	-0.8508407	0.785	0.927	4.16E-14	Ccnd2	TPC_Ageddown	TPC
7.72E-07	-0.8555098	0.306	0.524	0.018501	Gadd45g	TPC_Ageddown	TPC
1.99E-73	-0.8640358	1	1	4.77E-69	Rps8	TPC_Ageddown	TPC
2.03E-09	-0.8730866	0.132	0.391	4.88E-05	Ppp1r15a	TPC_Ageddown	TPC
2.60E-56	-0.8996999	0.993	1	6.24E-52	Hsp90ab1	TPC_Ageddown	TPC
1.75E-11	-0.9159048	0.583	0.811	4.20E-07	Ubc	TPC_Ageddown	TPC
1.30E-16	-0.9312592	0.535	0.844	3.11E-12	Id1	TPC_Ageddown	TPC
1.76E-07	-0.9426576	0.514	0.721	0.004225	Btg2	TPC_Ageddown	TPC
3.65E-60	-0.9834716	1	1	8.76E-56	Rps12	TPC_Ageddown	TPC
1.06E-09	-0.9862686	0.59	0.821	2.54E-05	Sbpl	TPC_Ageddown	TPC
4.94E-23	-1.0317962	0.139	0.607	1.19E-18	Hsph1	TPC_Ageddown	TPC
8.22E-24	-1.0493253	0.778	0.965	1.97E-19	Id3	TPC_Ageddown	TPC
1.59E-09	-1.0558315	0.285	0.536	3.81E-05	Dusp1	TPC_Ageddown	TPC
6.41E-18	-1.0868414	0.188	0.574	1.54E-13	Hspa1a	TPC_Ageddown	TPC
3.52E-32	-1.095085	0.854	0.968	8.45E-28	Hsp90aa1	TPC_Ageddown	TPC
9.40E-10	-1.1084843	0.215	0.484	2.26E-05	Zfp36	TPC_Ageddown	TPC
2.91E-11	-1.1240144	0.104	0.377	6.98E-07	Icam1	TPC_Ageddown	TPC
4.88E-10	-1.1273492	0.25	0.502	1.17E-05	Dcn	TPC_Ageddown	TPC
3.21E-32	-1.1493211	0.583	0.908	7.69E-28	Dnaja1	TPC_Ageddown	TPC
5.09E-07	-1.1775735	0.326	0.543	0.012197	Klf6	TPC_Ageddown	TPC
2.33E-12	-1.1830771	0.271	0.585	5.60E-08	Ier3	TPC_Ageddown	TPC
3.87E-07	-1.2116007	0.174	0.391	0.009273	Atf3	TPC_Ageddown	TPC
8.62E-30	-1.2237664	0.431	0.853	2.07E-25	Hspa1b	TPC_Ageddown	TPC
8.85E-14	-1.3153987	0.215	0.55	2.12E-09	Socs3	TPC_Ageddown	TPC
3.96E-11	-1.5478887	0.174	0.475	9.48E-07	Fosb	TPC_Ageddown	TPC
3.96E-08	-1.5956444	0.368	0.558	0.000949	Egr1	TPC_Ageddown	TPC
2.46E-10	-1.7352612	0.188	0.454	5.89E-06	Cebpd	TPC_Ageddown	TPC
4.70E-15	-1.9528483	0.306	0.636	1.13E-10	Fos	TPC_Ageddown	TPC
7.69E-20	-1.9651926	0.299	0.697	1.85E-15	Jun	TPC_Ageddown	TPC
1.54E-19	-2.0206056	0.479	0.8	3.70E-15	Junb	TPC_Ageddown	TPC
